# Neuropsychological differential diagnosis of Alzheimer’s disease and vascular dementia: a systematic review with meta-regressions

**DOI:** 10.3389/fnagi.2023.1267434

**Published:** 2023-11-06

**Authors:** Leo Sokolovič, Markus J. Hofmann, Nadia Mohammad, Juraj Kukolja

**Affiliations:** ^1^Department of Neurology and Clinical Neurophysiology, Helios University Hospital Wuppertal, Wuppertal, Germany; ^2^Faculty of Health, Witten/Herdecke University, Witten, Germany; ^3^Department of General and Biological Psychology, University of Wuppertal, Wuppertal, Germany

**Keywords:** Alzheimer’s disease dementia, vascular dementia, neuropsychology, differential diagnosis, meta-analysis

## Abstract

**Introduction:**

Diagnostic classification systems and guidelines posit distinguishing patterns of impairment in Alzheimer’s (AD) and vascular dementia (VaD). In our study, we aim to identify which diagnostic instruments distinguish them.

**Methods:**

We searched PubMed and PsychInfo for empirical studies published until December 2020, which investigated differences in cognitive, behavioral, psychiatric, and functional measures in patients older than 64 years and reported information on VaD subtype, age, education, dementia severity, and proportion of women. We systematically reviewed these studies and conducted Bayesian hierarchical meta-regressions to quantify the evidence for differences using the Bayes factor (BF). The risk of bias was assessed using the Newcastle-Ottawa-Scale and funnel plots.

**Results:**

We identified 122 studies with 17,850 AD and 5,247 VaD patients. Methodological limitations of the included studies are low comparability of patient groups and an untransparent patient selection process. In the digit span backward task, AD patients were nine times more probable (BF = 9.38) to outperform VaD patients ( $\beta_{g}$ = 0.33, 95% *ETI* = 0.12, 0.52). In the phonemic fluency task, AD patients outperformed subcortical VaD (sVaD) patients ( $\beta_{g}$ = 0.51, 95% *ETI* = 0.22, 0.77, BF = 42.36). VaD patients, in contrast, outperformed AD patients in verbal ( $\beta_{g}$ = −0.61, 95% *ETI* = −0.97, −0.26, BF = 22.71) and visual ( $\beta_{g}$ = −0.85, 95% *ETI* = −1.29, −0.32, BF = 13.67) delayed recall. We found the greatest difference in verbal memory, showing that sVaD patients outperform AD patients ( $\beta_{g}$ = −0.64, 95% *ETI* = −0.88, −0.36, BF = 72.97). Finally, AD patients performed worse than sVaD patients in recognition memory tasks ( $\beta_{g}$ = −0.76, 95% *ETI* = −1.26, −0.26, BF = 11.50).

**Conclusion:**

Our findings show inferior performance of AD in episodic memory and superior performance in working memory. We found little support for other differences proposed by diagnostic systems and diagnostic guidelines. The utility of cognitive, behavioral, psychiatric, and functional measures in differential diagnosis is limited and should be complemented by other information. Finally, we identify research areas and avenues, which could significantly improve the diagnostic value of cognitive measures.

## 1. Introduction

Alzheimer’s disease and cerebrovascular pathologies are the most common causes of primary dementia (Lobo et al., 2000; Plassman et al., 2007; Brunnström et al., 2009). They can present either as pure Alzheimer’s dementia (AD), pure vascular dementia (VaD), or as mixed dementia, i.e., AD with VaD (Sachdev et al., 2014; Custodio et al., 2017; Alber et al., 2019). Each of these three forms are thought to have different patterns of pathology, disease progression, and cognitive impairment (Bowler et al., 1997; Kramer et al., 2004; but see also van de Pol et al., 2011; Smits et al., 2015; Bischof et al., 2016; Dronse et al., 2016; Custodio et al., 2017; Ramirez-Gomez et al., 2017; Richter et al., 2020). In this systematic review and meta-analysis, we focus on the abilities of cognitive, functional, and measures of behavioral and psychological symptoms in dementia (BPSD; Shah et al., 2005; Perri et al., 2014; Deardorff and Grossberg, 2019) to distinguish AD and VaD. Cognitive impairment represents a core diagnostic criterion of both diseases (Looi and Sachdev, 1999; World Health Organization, 2004, 2022, 11; American Psychiatric Association, 2013). While AD patients have memory impairments, which initially overshadow other cognitive deficits, VaD patients have been described as presenting heterogenous cognitive profiles with common impairments of executive, attentional, and visuo-constructional abilities (Baldy-Moulinier et al., 1986; Almkvist, 1994; Looi and Sachdev, 1999; Hachinski et al., 2006; Skrobot et al., 2018; Sachdev et al., 2019; Smith et al., 2020). Accordingly, cognitive tests should have a substantial role in the differential diagnosis of dementia, especially in the early stages of the diseases (Hachinski et al., 2006; Sachdev et al., 2014, 2019; Smits et al., 2015; Skrobot et al., 2018). An accurate differential diagnosis is important for the choice of treatment (Hoffmann, 2013; Perng et al., 2018) and has implication for the disease prognosis (Bowler et al., 1997; Gill et al., 2013). Moreover, it enables caregivers to make informed decisions regarding home care or applying for a nursing home. Finally, a correct diagnosis has broader economic implications with higher costs in case of misdiagnoses (Hunter et al., 2015; Happich et al., 2016).

### 1.1. Neuropathology and cognitive deficits in AD and VaD

While our focus lies on the pattern of cognitive and functional impairments in AD and VaD, the research on the differentiation of both dementias is inextricably connected with the question of distinct and overlapping patterns of neuropathology in both dementias. Here, we review the central aspects of brain pathology and their putative behavioral and functional correlates.

Alzheimer’s dementia is the result of Alzheimer’s disease characterized by the presence of amyloid β-plaques and phosphorylated tau, which affect neurons and glial cells (Scheltens et al., 2021). Risk factors include advanced age, carrying at least one APOE ε4 allele, female sex, and unhealthy lifestyle (Scheltens et al., 2021). Amyloid β-plaques preferentially affect dendrites and typically first appear in the basal portions of the frontal, temporal and occipital neocortex from where they spread toward primary sensory cortices (Braak and Braak, 1991; Thal et al., 2002, 2008). This is also reflected in cortical atrophy, which in very mild and mild AD follows the distributions of amyloid β-plaques in the temporal and frontal lobes but is also present in parietal lobes (Dickerson et al., 2009). While amyloid β-plaques initially trigger the accumulation of neurofibrillary tangles, cognitive decline is thought to be driven by pathological tau depositions (Biel et al., 2021). Braak and Braak (1991) reported that neurofibrillary tangles and neuropil threads have a characteristic temporo-spatial evolution, which they divided into six stages (transentorhinal, limbic, isocortical, each further divided into mild and severe). In typical AD, the progression of AD pathology from the entorhinal, to limbic and neocortical areas is reflected in the progression of cognitive impairment from memory specific (at transentorhinal and limbic stages) to global impairment (in the isocortical stages; Grober et al., 1999; Therriault et al., 2022). Ten Kate et al. (2018) further investigated atrophy patterns in AD and their relationship with cognitive performance. They identified the following four different clusters of cortical atrophy and cognitive performance: (a) a medial-temporal atrophy cluster characterized by greatest impairments of memory and language; (b) a parieto-occipital atrophy cluster with the greatest visuo-spatial, executive, and attentional deficits; (c) a mild atrophy cluster with the least impaired cognition; and (d) a diffuse cortical atrophy cluster with temporal and frontal atrophy as well as intermediate cognitive impairments. Additionally, there exist biomarker defined AD variants (Graff-Radford et al., 2021), which primarily affect language (left temporo-parietal atrophy), visuo-spatial processing (posterior cortical atrophy), executive functioning (frontoparietal atrophy), motor functioning (corticobasal syndrome), and behavior (temporal atrophy).

Vascular dementia is an even more heterogenous group of brain pathologies of cerebrovascular origin comprising, among others, hypoperfusion dementia, strategic infarct dementia, poststroke dementia, multi-infarct dementia, and subcortical ischemic vascular dementia (Iadecola, 2013; Rincon and Wright, 2013). The latter is often a comorbidity in other primary dementias and plays a central role in vascular cognitive impairment (Iadecola, 2013; Dichgans and Leys, 2017). There are numerous risk factors for these cerebrovascular pathologies, which include advanced age, hypertension, diabetes, smoking, atrial fibrillation, hyperhomocysteinemia, dyslipidemia, and insulin resistance (Iadecola, 2013; Rincon and Wright, 2013; Dichgans and Leys, 2017). Cognitive impairment in VaD results from primary lesions and secondary, remote atrophy of both gray and white matter (Iadecola, 2013; Biesbroek et al., 2017; Dichgans and Leys, 2017). Depending on the exact cause of vascular injury, the deficits can appear immediately (e.g., in strategic infarct dementia) or—like in AD—develop over time (e.g., in cerebral small vessel disease; Iadecola, 2013; Dichgans and Leys, 2017). Studies examining small vessel disease and subcortical vascular dementia for example, have shown that not the total volume of, e.g., matter lesions but their location leads to specific cognitive deficits (Biesbroek et al., 2017). For example, lesions involving fronto-striatal networks are expected to cause deficits of verbal working memory, verbal fluency, and cognitive flexibility (Alexander et al., 1986; Frank et al., 2001; Hazy et al., 2007). Indeed, Camerino et al. (2021) found that lesions in bilateral thalamic radiations, caudate nuclei, and forceps minor lead to language and executive deficits. However, a meta-analysis by Hamilton et al. (2021) found that contrary to current consensus cognitive impairment resulting from sporadic cerebral small vessel disease is not limited to executive functioning and processing speed but that it affects all major domains of cognitive functioning. In fact, Tuladhar et al. (2015) demonstrated that the effects of white matter hyperintensities on cognition were completely moderated by cortical thickness.

Crucially, a mixture of AD and VaD pathologies is very common in autopsy samples and might be the most common cause of dementia, with pure VaD and AD present in as few as 10% of patients (Iadecola, 2013; Rizzi et al., 2014; O’Brien and Thomas, 2015; Dichgans and Leys, 2017; Boyle et al., 2018). Indeed, AD and VaD pathologies share common risk factors such as advanced age, hypertension, insulin resistance, diabetes, obesity, hyperhomocysteinemia, dyslipidemia, low levels of physical activity, cardiovascular disease, and genetic factors (Fahlander et al., 2002; Tsuno et al., 2004; Mathias and Burke, 2009; Iadecola, 2013; O’Brien and Thomas, 2015; Claus et al., 2016; Dichgans and Leys, 2017; Jørgensen et al., 2020; Koton et al., 2022; Lee et al., 2022; Eisenmenger et al., 2023). Mixed AD-VaD pathologies are characterized by amyloid β-plaques and neurofibrillary tangles as well as cerebrovascular lesions including the atherosclerosis of major brain vessels, white matter lesions and lacunar infarcts, microbleed, microinfarcts and cerebral amyloid angiopathy (Iadecola, 2013). Moreover, AD pathology has been shown to cause vascular pathology (Behl et al., 2007; Thal et al., 2008) and the mechanisms leading to VaD cause brain atrophy (Iadecola, 2013; Dichgans and Leys, 2017). Furthermore, a meta-analysis of prospective studies has shown that the presence of white matter hyperintensities, a sign of small vessel disease, increases the risk for AD by 25% and the risk for VaD by 73% (Hu et al., 2021).

The presence and interactions of both pathologies in mixed dementia has important implication for hypotheses about differences in cognitive performance between AD and VaD and has prompted research into the relative contributions of AD and VaD pathology to cognitive profiles of mixed dementia patients. In a series of studies, Price et al. (2005, 2012, 2015) showed that the degree of executive deficits correlates with the volume of periventricular and deep white matter lesions and that memory performance correlates with hippocampal volume. While the effects of both pathologies on cognition and BPSD are most likely additive (Attems and Jellinger, 2014; Chui and Ramirez-Gomez, 2015; Lam et al., 2021), it has been suggested that AD pathology starts to dominate the clinical presentation and with time overwhelms the effects of cerebrovascular disease (Chui et al., 2006). Regardless of the underlying pathology as AD, VaD and mixed dementia progress cognitive abilities decline to a degree of impairment, which precludes the use of complex cognitive assessment (Bowler et al., 1997; Wentzel et al., 2001; Laukka et al., 2012; Smits et al., 2015; Smith, 2017). Despite the complexities of the relationship between brain pathology and cognitive and functional impairment as well as BPSD, there exist many studies on the differences in cognitive and functional profiles between AD and VaD. We next review the most important findings from several domains reported in the literature.

### 1.2. Motor functioning and apraxia

Dementia leads to impairments of motor abilities and praxis. Whereas these deficits appear later in typical AD, cerebrovascular etiology of VaD can lead to motor impairments and apraxia early in the course of the disease (Lezak, 2012). Studies examining differences between AD and VaD in motor functioning have shown that patients with mild to moderate VaD present with more motor symptoms than AD patients (Starkstein et al., 1996; Aitken et al., 1999; Simpson et al., 1999). Differences have also been reported in motor speed. For example, Almkvist et al. (1993) compared patients matched for dementia severity and found better performance of AD patients in simple reaction time and finger-tapping tasks and concluded that motor speed shows promise in distinguishing VaD from AD. Thus, whereas studies failed to find clear correlations between cognitive impairment and brain lesions in VaD (Lafosse et al., 1997; Jones et al., 2004), the relationship between vascular lesions and motor performance appears to be more straightforward. Apraxia, on the other hand, is common in AD and VaD as well as many other dementias (Nagahama et al., 2015).

### 1.3. Processing speed and attention

In the domains of attention and processing speed, Mendez et al. (1997) investigated differences between subcortical VaD (sVaD) and AD on measures of information processing speed, which included (a) a simple reaction time task, which required a key press, when the letter A was presented, (b) a stimulus categorization task, in which letters other than A were also presented but required no response, (c) a response selection task, in which participants had to react to the letter A with one and to the letter B with another key, and (d) finally a continuous performance task, which was a longer version of the stimulus categorization task. They conclude that slower reaction times of sVaD patients in three runs of the continuous performance task cannot be explained by motor or mental slowing, but that they reflect a failure of response criterion adaptation (see also Lamar et al., 2002) or impaired tonic arousal. However, two other studies found no differences in measures of processing speed (Padovani et al., 1995; Heyanka et al., 2010).

Villardita (1993) matched AD and VaD patients on dementia severity and found superior performance of AD patients in selective attention. The relative advantages of AD patients in the study might however also stem from greater motor impairments of VaD patients. Further, deficits of executive attention have been found to be common already in early AD (Smits et al., 2015; McDonough et al., 2019). Thus, there seems to be no clear distinction between AD and VaD in respect to deficits of attention.

### 1.4. Executive functioning and reasoning

The terms executive functioning denotes a multifactorial concept (Testa et al., 2012; Diamond, 2013) which describes a broad collection of cognitive as well as affective and motivational abilities, required for goal-directed behavior (Li et al., 2018). Testa et al. (2012) tested their participants using 19 common measures of executive functioning. Using principal component analysis, they identified the following six areas of executive functioning: prospective working memory, set-shifting and interference management, task analysis, response inhibition, strategy generation and regulation as well as self-monitoring and set-maintenance. Notably missing from this list, is the energizing factor of executive functioning, which governs drive and motivation (Stuss, 2011; Diamond, 2013) and takes on a prominent role in psychiatric and neurodegenerative diseases. Numerous studies have been able to verify the clinical impression that VaD and AD patients differ in their performances on measures of executive functioning. For example, Starkstein et al. (1996), investigating a sample of mildly impaired patients, found more impaired cognitive flexibility in VaD compared to AD. Since perseverations in different cognitive domains, i.e., memory vs. executive functioning, may result from different neurocognitive processes, it has been suggested that perseverations in AD result from semantic dedifferentiation and that perseverations in VaD reflect problems in executive abilities of task-switching and terminating tasks (Carew et al., 1997; Lamar et al., 1997; Mendez et al., 1997; Graham et al., 2004). Lamar et al. (2002) further reported differences in task-set maintenance. Whereas AD only displayed difficulties in learning a mental set, VaD patients showed impaired set-attainment and maintenance. In general, VaD patients have greater impairment of response inhibition, conceptualization, set maintenance, planning as well as structuring and manipulating information in working memory already in the mild stage of dementia (Mendez and Ashla-Mendez, 1991; Padovani et al., 1995; Kandiah et al., 2009). However, Baillon et al. (2003), after controlling for depression, age, sex, pre-morbid intelligence, and dementia severity, reported that patients with mild AD committed more errors on the Trail Making Test B, which suggests a greater impairment of set-shifting in AD compared to VaD. There are also reports of no differences in executive functioning between mild to moderate AD and VaD (Bayles and Tomoeda, 1983; McGuinness et al., 2009, 2010).

In abstract reasoning, Shuttleworth and Huber (1989) report impairments of AD patients on the Pictures Absurdities Test. Further, Gainotti et al. (1992) found that mild to moderate AD patients produced more odd and globalistic answers on Raven’s Colored Matrices than participants with multi-infarct dementia (MID). Almkvist et al. (1993), however, reported better performance of AD patients in the Wechsler Intelligence Scale (WAIS) Similarities subtest in a study comparing severity matched patients. Thus, the existing studies discern no clear pattern of differences between AD and VaD on measures of abstract reasoning and executive functioning.

### 1.5. Language

During the progression of AD and VaD, the difference between performance on language measures increases and shows superior performance of VaD (Smits et al., 2015). There are numerous studies reporting greater language deficits in AD patients (Barr et al., 1992; Engel et al., 1993; Villardita, 1993; Kertesz and Clydesdale, 1994; Padovani et al., 1995). Although Padovani et al. (1995) found worse performance of MID patients on the Controlled Word Association Test, the general pattern of findings regarding semantic and phonemic fluency is mixed. Multiple studies reported no differences in semantic fluency between mild and moderate AD and VaD (Barr and Brandt, 1996; Crossley et al., 1997; Vanderploeg et al., 2001). Others found that patients with mild AD outperform patients with mild VaD (Starkstein et al., 1996; Tierney et al., 2001). Giovannetti et al. (2008) also showed that patients with greater vascular pathology also display greater difficulties in syntactic comprehension.

### 1.6. Memory

Deficits of visual and verbal semantic memory are common and are observed early in the course of AD (Ricker et al., 1994; Zimmer et al., 1994; Libon et al., 1996; Laine et al., 1997; Baillon et al., 2003; Clague et al., 2005; Braaten et al., 2006). Carew et al. (1997) showed that these deficits of AD patients might stem from a loss of subordinate defining features. Thus for AD patients, category-related and unrelated words are equally activated in a semantic fluency task (Lukatela et al., 1998; Giovannetti et al., 2001; Braaten et al., 2006). This might also be the reason why AD patients more often accept new words as old in verbal recognition memory (Gainotti et al., 2001). The dedifferentiation or loss of organization of memory and of its neural bases in AD might further explain the general recall deficits also observed on episodic, semantic as well as memory measures combining semantic and episodic memory (Batchelder et al., 1997; Cannatà et al., 2002; Davis et al., 2002; Hampstead et al., 2010; Laukka et al., 2012; Kwok et al., 2015). However, Bentham et al. (1997) found no differences in semantic memory between mild to moderate AD and VaD. Similarly, in a study of well-matched but small samples, Vuorinen et al. (2000) reported slightly greater deficits in semantics in AD but also found similar semantic deficits in both diseases.

There is a consensus that AD patients display greater impairment of verbal and visual episodic memory (Mendez and Ashla-Mendez, 1991; Padovani et al., 1995; Baillon et al., 2003; Graham et al., 2004; Hildebrandt et al., 2009; Hampstead et al., 2010), which is prominent already in the early stages of the disease (Smits et al., 2015). However, no differences between AD, VaD and depressed patients in delayed recall have also been reported (Taylor and Gilleard, 1990). As mentioned, the deficits in semantic memory have been suggested to also play an important role in learning and delayed recall of verbal material. For example, semantic impairment has been reported to correlate with recall intrusions in mild AD (Loewenstein et al., 1991). Further, Bernard et al. (1992) reported that patients with mild to moderate AD failed to use semantic categories based on typicality to improve their recall. Del Re et al. (1993) compared age and education matched, mildly to moderately impaired AD and MID groups and found inefficient encoding and recall in AD patients. They suggested that in contrast to MID patients, the poor, non-strategic encoding of AD patients leads to an inefficient and random recall (Barr et al., 1992). AD patients were also found to show flatter and slower learning curves than VaD patients, which tended to have a normal learning curve (Barr et al., 1992). Gainotti et al. (1989) suggested that impaired learning unlike the rate of forgetting (Gainotti et al., 1998) should therefore distinguish AD and MID in mild to moderate stages of dementia. Batchelder et al. (1997) also reported that storage and delayed retrieval of new information on a time scale of a few minutes are the earliest deficits in AD (see also Hassing and Bäckman, 1997; Vanderploeg et al., 2001). These deficits most likely reflect the spatial patterns of neuronal loss, senile plaques and neurofibrillary tangles deposits, which impair structure and function of the CA1, subiculum, parasubiculum, and the entorhinal cortex and in turn lead to functional isolation of the hippocampus (Carlesimo et al., 1993). These hypotheses however have not yet been examined in a joint model of cognitive and neuroimaging data (Batchelder et al., 1997; Hofmann and Jacobs, 2014; Palestro et al., 2018; Roelke and Hofmann, 2020). Corroborating data for these anatomic hypotheses and their potential in distinguishing primary dementia forms has however been reported by Della Sala et al. (2012), who used a conjunctive binding test to successfully identify AD. They suggested that the discriminative power of the test is based on its taxing of the perirhinal and entorhinal cortices, but not the hippocampus. It thereby identifies deficits present in AD but not in other dementias.

In delayed verbal recognition memory, AD patients also perform worse. For example, Doddy et al. (1998) reported that mild to moderate VaD outperformed moderate AD in verbal recognition memory (Tierney et al., 2001; Traykov et al., 2002; Hildebrandt et al., 2009). A study with well-matched groups of AD and VaD patients with moderate dementia showed that in recognition memory tests AD patients have a liberal response bias, i.e., a tendency to respond “old” to new items (Snodgrass and Corwin, 1988; Stanislaw and Todorov, 1999) which is stronger when presented with semantically related words (Barr et al., 1992). In contrast to the dedifferentiation hypothesis, the authors suggested that this most likely reflects an executive and not a semantic deficit. Worse performance of AD patients on recognition memory tests has also been suggested to reflect faulty criterion setting (Yuspeh et al., 2002) i.e., choosing a too low threshold for the strength of memory signal at which to respond “old” (Snodgrass and Corwin, 1988; Stanislaw and Todorov, 1999). Finally, no differences between patient groups were found in repetition priming (Carlesimo et al., 1995; Beatty et al., 1998).

### 1.7. Visuo-spatial processing

Kandiah et al. (2009) reported greater visuo-spatial impairment in sVaD on drawing tasks and the WAIS Block Design subtest. However, the authors suggested that these deficits are more reflective of executive deficits than a true impairment of visuo-spatial processing (see also Freeman et al., 2000). Reduced left sided visual exploration and symptoms of hemispatial inattention have also been reported in AD patients (Fischer et al., 1990a; Fitten et al., 1995; Cherrier et al., 1999). Arnaoutoglou et al. (2017) also reported that color perception differentiated AD from VaD, with AD patients displaying greater impairment. Other studies however reported mixed findings and allow no clear conclusions about differences in impairments of visuo-spatial processing (Hier et al., 1989; DeBettignies et al., 1993; Villardita, 1993; Kertesz and Clydesdale, 1994; Padovani et al., 1995; Starkstein et al., 1996; Yamashita et al., 1997; Matsuda et al., 1998; Fahlander et al., 2002; Loewenstein et al., 2006; Nordlund et al., 2007; Heyanka et al., 2010).

### 1.8. Other domains

Differences in unawareness of deficit have also been reported with AD showing more pronounced anosognosia (Wagner et al., 1997), which could also be explained by more severe dementia of AD patients in that study. In contrast, Starkstein et al. (1996) reported lesser anosognosia in AD patients and Zanetti et al. (1999) found no differences between dementia types. In respect to BPSD, Fischer et al. (1990b) reported that patients with mild AD were more depressed than patients with mild VaD. On the other hand, Sultzer and colleagues found that, when matched for dementia severity, VaD patients had more and more pronounced behavioral symptoms, anxiety, and depression than AD patients (Sultzer et al., 1993; Aitken et al., 1999; Kandiah et al., 2009; Anor et al., 2017). There are however also reports of no differences between moderate to severe AD and mild to moderate MID in the frequency of delusions (Flynn et al., 1991), psychiatric and behavioral symptoms (Sultzer et al., 1992). Emotion recognition has also been reported to be more impaired in AD patients with moderate dementia (Shimokawa et al., 2000, 2003). Finally, there are heterogenous findings regarding impairment of olfactory perception (Knupfer and Spiegel, 1986; Gray et al., 2001) and of basic and instrumental activities of daily living (Gorelick et al., 1994; Zimmer et al., 1994; Zanetti et al., 1999; Isik et al., 2007; Brodaty et al., 2011; Gill et al., 2013; Giebel et al., 2016; Tisato et al., 2016; Wei et al., 2018).

In summary, the existent literature suggests superior performance of AD in areas such as verbal fluency, cognitive flexibility, visuo-spatial processing, and digit span. Tasks in these domains require abilities such as cognitive control in recalling, maintaining, or updating information in visual or verbal working memory. They also depend on cognitive flexibility, task set maintenance, and speed of processing. In contrast, AD patients are suggested to show greater impairment in tasks involving semantic and episodic memory, where encoding, storage, recall and recognition of learned verbal or visual material are all impaired.

### 1.9. Issues in research and clinical praxis

Despite these findings, cognitive, functional and BPSD measures only have a limited role in distinguishing AD from VaD (Almkvist, 1994; Erker et al., 1995; Voss and Bullock, 2004; Hayden et al., 2005; Oosterman and Scherder, 2006; Mathias and Burke, 2009; Dutilh et al., 2019). This has multiple reasons. For example, misdiagnoses in original studies (Wetterling et al., 1996; Sachdev et al., 2014; Hunter et al., 2015; Happich et al., 2016) and considerable rates of mixed dementia (Groves et al., 2000; Zekry et al., 2002, 2003; De Jager et al., 2003; Brunnström et al., 2009; Heyanka et al., 2010; Sachdev et al., 2014; Claus et al., 2016).

Reflecting common neuropathological changes, AD and VaD can present with similar patterns of cognitive impairment (Almkvist et al., 1993, 1999; Fahlander et al., 2002; Mathias and Burke, 2009). The differences in cognitive impairment between AD and VaD are further attenuated by the variable patterns of brain pathology within each diagnostic group (Matsuda et al., 1998; Murray et al., 2011; Iadecola, 2013; Rincon and Wright, 2013; Noh et al., 2014; Sachdev et al., 2014; Dong et al., 2016; Dronse et al., 2016; Dichgans and Leys, 2017; Richter et al., 2017; ten Kate et al., 2018; Alber et al., 2019). While studies published in the last decades began to consider the various VaD subtypes (Libon et al., 1997; e.g., Cannatà et al., 2002; Chui et al., 2006; Ramirez-Gomez et al., 2017), AD continues to be treated as a homogenous entity (Moretti et al., 2002). A further complication in the diagnostic and research processes is the blurring of cognitive differences due to the cortical vs. subcortical pathology, which cuts across the AD-VaD division and has been shown to lead to different patterns of cognitive impairment (Vanderploeg et al., 2001; Price et al., 2005, 2012; Behl et al., 2007; Libon et al., 2008; Lam et al., 2021). Using a mixture of sVaD and VaD patients in a study necessarily leads to reduced observed differences between AD and VaD groups.

The utility of established cognitive, functional and BPSD measures in differential diagnostics is also limited by their design. Most measures were developed to identify impaired cognition and not to differentiate between causes of cognitive impairment (Baldy-Moulinier et al., 1986; Cannatà et al., 2002; Lezak, 2012; Sudo et al., 2019). They are thus underspecified, meaning that impaired performance can result from an impairment of one or multiple affective, perceptual, and cognitive processes involved. Consequently, most neuropsychological tests lack specificity in respect to functional and structural neuroanatomy. Finally, unlike for semantic dementia (Rogers et al., 2004; Dilkina et al., 2008; Hoffman et al., 2018), mechanistic models of AD and VaD, which would inform the construction of more sensitive tests, new scoring procedures and advance understanding of the connections between pathology and behavior, are also scarce (Batchelder et al., 1997; Chosak Reiter, 2000; Becker and Lim, 2003; Pooley et al., 2011).

The etiological, psychometric, conceptual, and other limitations lead to a modest contribution of cognitive measures in distinguishing between forms of dementia, especially when compared to a combination of brain imaging and cerebrospinal fluid markers (Boutoleau-Bretonnière et al., 2012; Bruun et al., 2018). The promising advances of imaging and biomarker studies nevertheless do not render cognitive testing, functional and BPSD measures obsolete or irrelevant, as biomarkers do not provide information about the current cognitive and everyday functioning levels of the patient, disease awareness or affective state. Such information is crucial for patient care and case specific treatment planning, treatment evaluation as well as assessment of disease coping and caregiver stress (Baldy-Moulinier et al., 1986; Lezak, 2012), thus underlining the need for an updated review of available cognitive, functional and BPSD measures.

### 1.10. Previous reviews and meta-analyses

Before presenting the aims of the present study, we shortly review the existing syntheses of the subject matter. Almkvist (1994) published the first review on the topic, in which he aimed to provide a clinically applicable description of the distinguishing patterns of neuropsychological and sensory-motor impairments in AD and VaD. He reviewed measures of intelligence, executive functioning, verbal ability, visuospatial functioning, attention, working, episodic, and semantic memory as well as sensory and motor functions. He considered the type of participant population (clinic vs. community), sampling method (inclusion criteria vs. consecutive cases), variations in the diagnostic procedure and criteria, differences in age, sex, educational background, and the degree of dementia severity as moderating factors. He found that VaD patients perform worse than AD patients in executive functions, fluency, attention, and motor functions. Conversely, he argued that AD patients show greater impairment in naming and produce more memory intrusions. However, since the differences were small, he concluded that they do not warrant the use of neuropsychological tests for differentiation purposes.

Next, Looi and Sachdev (1999) using a more structured approach to search and selection of studies investigated differences in intelligence, language, attention/immediate memory, verbal learning and memory, nonverbal memory, conceptual function, arithmetic, frontal executive function, constructional abilities, working memory and concentration, motor speed, orientation as well as visual and tactile perception. They concluded that VaD patients have a relatively preserved long-term memory and a greater impairment of frontal executive functioning. These differences, they argued, follow from mesiotemporal pathology in AD and lesions of frontal-subcortical circuits in VaD. The authors also noted that many studies neither accounted for different VaD subtypes nor controlled for the influence of demographic variables in their analyses.

The first meta-analysis on the topic was published by Oosterman and Scherder (2006) and focused on the WAIS. They argued that, despite contradictory findings in the literature, WAIS subtests can differentiate between AD and VaD and are especially sensitive to the distinction between sVaD and AD. For their analyses they chose studies, which matched the participants on age and dementia severity but not education, which is surprising since education strongly influences the performance on the WAIS (Strauss et al., 2006; Lezak, 2012). Compared to AD, VaD tended to perform better on the Object assembly and Digit Span backward subtests and worse on the Information subtest. As hypothesized, they also found that sVaD patients outperformed AD on Block Design, Digit Span backward, Object Assembly, Picture Arrangement, and Picture Completion subtests. This demonstrates that VaD subtypes influence the differences in cognitive deficits between AD and VaD.

Finally, Mathias and Burke (2009) published a meta-analysis covering a broad range of cognitive measures encompassing Orientation and Attention, Perception, Memory, Verbal ability, Construction, Concept formation and reasoning as well as Motor performance, executive and general functioning. They found that the differences between AD and VaD did not correlate with differences in age, education and MMSE scores. The meta-analyses also identified no test, on which the difference between VaD and AD was great enough to be relevant for single case diagnostics. Importantly, in contrast to previous reviews and meta-analyses they found no differences in executive functioning between VaD and AD. Nonetheless they propose that a test of emotion recognition and a delayed story recall test could potentially, in conjunction with other information, contribute to differential diagnosis.

### 1.11. The present study

In our meta-analyses, we build on previous reviews on the topic (Almkvist, 1994; Looi and Sachdev, 1999; Oosterman and Scherder, 2006; Mathias and Burke, 2009). Each of them increased methodological rigorousness in providing an overview of the diagnostic possibilities and challenges as well as guidance for clinicians. In this vein, we also focus on effect sizes to determine clinical relevance of reported differences. Additionally, we conduct our analyses in the Bayesian framework to be able to quantify the evidence (see Table 1) for or against a potential difference between AD and VaD, and to account explicitly for the uncertainty of reported effect sizes in our syntheses. Further, we synthesize the research not only on the contribution of cognitive, but also functional and BPSD measures to differential diagnostic of AD and VaD, thus expanding our focus on other areas affected by dementia pathologies. Prior to performing statistical analyses, we organized the measures into most representative domains (Strauss et al., 2006; Lezak, 2012). In our statistical analyses, we further compared AD patients to the following subtypes of vascular dementia: vascular dementia (VaD), subcortical vascular dementia (sVaD), multi-infarct dementia (MID), and vascular mild cognitive impairment (VCI) as a counterpart to mild cognitive impairment (MCI; Erkinjuntti et al., 1986; Dichgans and Leys, 2017; Rundek et al., 2022). Due to the evolution of the construct of vascular dementia, the VaD group is a placeholder term for all the studies which did not specify a VaD subtype or included a heterogenous patient group with multiple VaD subtypes. To be able to include research on MID we also expanded the search beyond 1989.

**Table 1:** Heuristic for interpretation of bayes factor BF_10_ adapted from Schönbrodt and Wagenmakers (2018).

Bayes factor	Evidence strength
>100	Extreme evidence for *H_1_*
30–100	Very strong evidence for *H_1_*
10–30	Strong evidence for *H_1_*
3–10	Moderate evidence for *H_1_*
1–3	Anecdotal evidence for *H_1_*
1	No evidence for either *H_1_* or *H_0_*
1/3–1	Anecdotal evidence for *H_0_*
1/10–1/3	Moderate evidence for *H_0_*
1/30–1/10	Strong evidence for *H_0_*
1/100–1/30	Very strong evidence for *H_0_*
< 1/100	Extreme evidence for *H_0_*

H1 stands for the alternative hypothesis.

We assessed the risk of bias using the Newcastle-Ottawa-Scale, a commonly used scale for non-randomized studies (Farrah et al., 2019), which rates the selection of participants, case definitions, the comparability of studied groups as well as the description and comparability of experimental treatment in studied groups.

We report the maximum-a-posteriori estimate with its 95% equal-tailed credible intervals (ETI) of the posterior distribution. In contrast to confidence intervals, credible intervals can be interpreted as the certainty of the result, i.e., there is a 95% probability that the estimated effect lies in the interval. Further, we also report Bayes Factor (BF) as a measure of the quantity of evidence for the alternative hypothesis, which in our analyses states that there is a non-zero difference between AD and VaD subtype, with positive effect sizes indicating that AD performed betterr, i.e.,


$$M_{AD}-M_{VaD}=0$$

The interpretation of the Bayes Factors can be seen in Table 1.

Since we want to formulate suggestions for clinical practice, we also define the region of practical equivalence (Kruschke, 2018) as the interval between −1.7 and 1.7 Hedges’ *g*. For normally distributed mean differences this interval corresponds to an overlap of test score distributions of two groups of about 25% (Zakzanis, 2001). Such an effect size would ensure the correct differential diagnosis in three out of four patients. To interpret the results, one needs to consider the maximum-a-posteriori estimate as the most probable value of the effect size, its credible interval as a measure of certainty in the effect size magnitude and the Bayes Factor as a measure of evidence for the alternative hypothesis compared to the *a priori* probability. Further, if the estimate of the effect size lies in the region of practical equivalence, then its contribution to distinguishing between AD and VaD in clinical praxis is very limited. In Bayesian analysis, the researcher must choose prior distributions for effects to be estimated. Since the choice of prior can influence the magnitude and variance of effect estimates and the Bayes factor (Gelman et al., 2013; Kruschke, 2015), we conducted sensitivity analyses regarding the choice of prior distributions to investigate the influence of the prior distribution on the parameter estimates.

The objectives of this systematic review and meta-analysis were to assess the differences between AD and VaD in available domains; to determine their clinical relevance; and to suggest possible avenues of research and recommendations for clinical practice. Our results show small group differences consistent with the DSM V and ICD-11 classification systems with better average performance of VaD patients in episodic and semantic memory tasks and better average performance of AD patients in phonemic fluency and digit span backward tasks. For other cognitive impairments thought to contribute to differential diagnosis according to the DSM V and ICD-11, we however find no support or even strong evidence for no difference between AD and VaD. For example, we find little support for differences between AD and VaD in complex attention and processing speed, which are described as domains of prominent deficits in VaD by DSM V and ICD-11 (see Supplementary Material 4). Contrary to suggestions in guidelines for differential diagnosis (Hachinski et al., 2006), we also found no support for differences in cognitive flexibility. In the discussion we consider the implications of these findings for the specific cognitive processes involved, clinical praxis, and future research.

## 2. Methods

### 2.1. Eligibility criteria

We searched for quantitative studies:

1. Comparing AD to VaD, sVaD, VETI, or MID. We did not consider studies reporting early onset AD, i.e., AD with onset before age of 64.
2. Reporting measures of cognition, psychiatric symptoms, or activities of daily living (ADL). Studies reporting only the Mini Mental Status Examination (MMSE), or the Montreal Cognitive Assessment scores were excluded.
3. Reporting descriptive statistics or effect sizes for outcomes of interest.
4. Investigating community and hospital-based populations.
5. Published in English or German language.
6. Reporting mean age, a quantitative measure of dementia severity, mean years of education, and number or proportion of women in each group.

Studies which failed to report this information were excluded.

### 2.2. Information sources, search strategy and selection process

In December 2020, we conducted the following search on PubMed and PsychInfo databases:

*Vascular dementia* AND *alzheimer* AND cognit* NOT *frailty* NOT *plasma* NOT *iron* NOT *clusterin* NOT *cancer* NOT *atherosclerosis* NOT *pollution* NOT *post-mortem* NOT *astroglial* NOT *hypotension* NOT *olfactory* NOT *diabetes*

In addition, we used the following filters: English and German language, adults 65+ years. Search results were screened independently by LS and NM. Disagreements were resolved by consensus. Due to the number of identified studies and non-responses to inquiries in the previous meta-analysis (Mathias and Burke, 2009), no attempt was made to contact study authors to obtain missing information.

**Figure 1:**
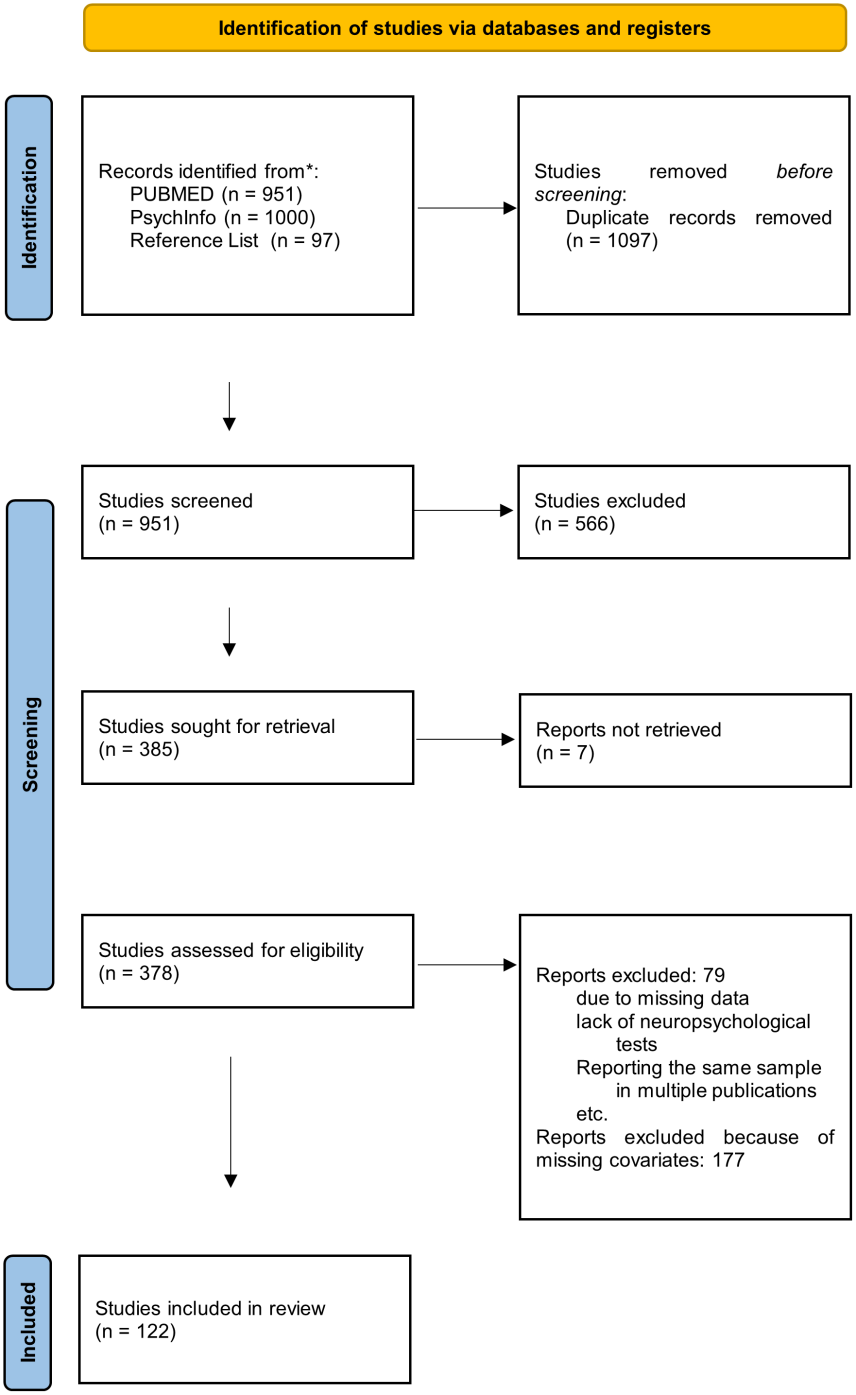
Prisma flowchart of the study selection process.

We first screened the titles of the search results to identify relevant studies and exclude duplicates. In the second step, study abstracts were screened using the eligibility criteria to identify studies, which would be considered for a full text review. To qualify for the full review stage, the abstract had to indicate that cognitive functions were assessed in AD and VaD. We identified 385 relevant studies, of which we could not retrieve seven studies. The 378 obtained studies were read, and their reference lists checked for relevant studies not found in the database searches. We also inspected the references from Mathias and Burke (2009) to ensure, that we identified all studies included in their meta-analysis. After applying our inclusion criteria to all thus identified studies, 122 studies which fulfilled all inclusion criteria were retained for the meta-analyses. They are reported in Supplementary Table 22. All excluded studies failed to meet at least one of the inclusion criteria (see also Figure 1).

### 2.3. Data collection process and data items

Data were extracted independently by LS and a research assistant. All disagreements were resolved by consensus. We checked for studies reporting the same or an incremented sample to only use unique data provided by each study. In case of longitudinal studies, we only included the baseline. If a study reported scores divided according to dementia severity, i.e., mild, moderate, severe AD, and VaD, these scores were combined to obtain a single, pooled score. If studies reported multiple VaD subtypes these were treated as separate studies in the analyses. This introduces dependencies between data, which were addressed by using hierarchical regression models (see Section 3.6).

We extracted all cognitive, behavioral, psychiatric and ADL scores, VaD subtype, average age, dementia severity, years of education, and number or proportion of women. To structure the analyses and results, the reported measures were then sorted into most representative domains based on established classifications (Strauss et al., 2006; Lezak, 2012), previous reviews on the topic as well as criterion and construct validity of tests reported in the literature. For example, following a paper on the harmonization of neuropsychological assessment in neurodegenerative dementias in Europe (Costa et al., 2017) we grouped apraxia and motor symptoms into a single domain. As in previous reviews (Almkvist, 1994; Looi and Sachdev, 1999) and to reflect the fact that most tests of attention are speed-based (Strauss et al., 2006; Lezak, 2012), we also jointly discuss attention and psychomotor speed. In accordance with recent studies, we categorized verbal fluency tests as language tasks with prominent executive components (Unsworth et al., 2011; Whiteside et al., 2016; Aita et al., 2019; Pires et al., 2019). The domain Visuo-Spatial Processing was defined to comprise visual perception and constructional praxis as the latter critically depends both on visual input and spatial processing. For example, the Judgment of Line Orientation Test and Block Design Tests have been shown to correlate highly (*r* = 68, Strauss et al., 2006). Further, Almkvist (1994) and Ricker et al. (1994) both viewed the tests as belonging to a single cognitive domain. We consider our classification to be useful in structuring the review of impairments and the presentation of our results. However, we do not propose a new taxonomy of cognitive functioning. Also, VaD subtype was determined according to the description of patients’ lesions. If data on multiple dementia subgroups were reported then the two most comparable ones were used, e.g., if a study reported results for MCI, mild AD, and VCI, we only compared the MCI and VCI groups. In the final sample there were no missing data, thus no imputation was needed.

Overall, we analyzed the following domains: language production and comprehension, apraxia, motor functioning, perception, visuo-spatial processing, attention and processing speed, executive functioning, memory, global functioning, orientation to time and space, activities of daily living, disease awareness, depression, anxiety, and other affective symptoms, neuropsychiatric symptoms, intelligence measures (see also Oosterman and Scherder, 2006), and reading ability.

Since studies measured dementia severity with different measures these scores were remapped to the range 0–100, with 0 denoting maximal cognitive impairment and 100 normal functioning. The formula used was:


$$\begin{array}{l} transformedscore=old\;score_{\min}\\ \;\;\;\;\;\;\;\;\;\;\;\;\;+\frac{transformedscore_{\max}-transformedscore_{\min}}{old\;score_{\max}-old\;score_{\min}}\\ \;\;\;\;\;\;\;\;\;\;\;\;\;*\left(originalscore-old\;score_{\min}\right) \end{array}$$

for the MMSE this simplifies to:


$$transformedscore=\frac{100}{30}*originalscore$$

For studies reporting standard scores (*z* or T), an approximate range spanning four standard deviations above and below the mean of the control group was used (these studies were: Carlesimo et al., 1993, 1994, 1995; Erker et al., 1995). Differences in reported scores, average age, years of education, proportion of women, and severity of dementia between AD and VaD subtypes were also calculated as the difference between the average of the AD group minus the average of the VaD subtype group.

### 2.4. Study risk of bias assessment

Risk of bias assessment was conducted independently by the first and third author for each of the 122 identified studies by funnel plot inspection and the Newcastle-Ottawa Scale for case control studies,^1^ which assesses the studies according to selection of eligible participants, comparability of study groups and identical exposure to experimental or study procedure. It thus rates the risk of bias in the selection of participants, their comparability, and potential differences in study execution in each group. The Newcastle-Ottawa Scale does not provide an overall risk assessment; thus, no overall risk of bias judgment was produced. Disagreements between assessors were resolved by discussion until a consensus rating was reached. For individual domains, Table 2 shows the respective median ratings on the Selection (0–4 stars), Comparability (0–2 stars), and Exposure (0–2 stars) scales of the Newcastle-Ottawa-Scale. The Selection items evaluate the case definition and representativeness as well as the selection and definition of control subjects. Comparability items assess how the study ensured comparability of case and control subjects by assessing the study design and controlling for nuisance variables in statistical analyses. Finally, Exposure items assess the bases for case or control group assignment, blinding of investigators with respect to group membership of study participants and the description of non-response rate.

**Table 2:** Median scores on the NOS scales for studied domains.

Domain	Selection	Comparability	Exposure
Apraxia	3	1	1
Motor functioning	4	1	2
Attention	3	1	1
Processing speed	3	0	1
Intelligence measures	3	0	1
Executive functioning	3	1	1
Reasoning	4	0	1
Language comprehension	3	0	1
Language production	3	1	1
Reading	2	1	1
Memory	3	1	1
Perception	3	0	1
Visuo-spatial processing	3	1	1
Activities of daily living	3	0	1
Global functioning	3	0	1
Affective symptoms	3	1	1
Neuropsychiatric symptoms	4	1	1
Disease awareness	4	1	1
Orientation to time and space	3	0	1

Ratings of individual studies are available in Supplementary Table 22 in Supplementary Material 1.

### 2.5. Effect measures

If needed descriptive statistics were transformed to means and standard deviations according to Wan et al. (2014). Then, Cohen’s *d* was calculated as $d=\frac{{\bar{X}}_{AD}-{\bar{X}}_{VD}}{s}$ ; with $s=\sqrt{\frac{\left(n_{AD}-1\right)SD_{AD}^{2}+\left(n_{VD}-1\right)SD_{VD}^{2}\;}{n_{AD}+n_{VD}-2}}$ . Other effect sizes were transformed to Cohen’s d using the online tools at http://www.psychometrica.de/effect_size.html. Finally, we transformed Cohen’s *d* to Hedges’ *g* values to obtain an effect size measure corrected for small sample bias (Cooper et al., 2009). All Hedges’ *g* values are calculated so that a negative effect size reflects worse and a positive effect size better performance of AD in comparison to the VaD subtype.

### 2.6. Synthesis methods

We performed the analyses in R 4.2.0 with brms 2.17.0 (Buerkner, 2017; Bürkner, 2018). All models were fitted using the No-U-Turn-Sampler as implemented in brms. After fitting, MCMC chains were checked for convergence and mixing by inspection of trace plots, $\hat{R}$ values (all ≤1.01) and effective sample sizes (Gelman et al., 2013). Varying intercept random effects models were fitted as they are recommended for psychological studies (Field and Gillett, 2010; Röver et al., 2021). The effect sizes were weighted by their standard error (Harrer, 2022). The *τ* statistic—the standard deviation of the random effects—was estimated for each level of the random factor to estimate the heterogeneity between studies ( $\tau_{Study}$ ) and between outcomes within studies ( $\tau_{Study/ES}$ ) when a nested model was fitted. Prior distributions for intercepts ( $\beta_{0}$ ) and predictors ( $\beta_{i}$ ) were set to:


$$\beta_{0},\beta_{i}\sim Cauchy\;\left(0,1\right)$$

which reflects our expectation, that the effect sizes will be in the interval from −5 to 5 g with a probability of 87%. Such a prior also allows for some very large effects thus making the model less sensitive to outliers. The priors for the variability of random effects (*t*) were:


$$\tau_{Study},\tau_{Study/ES}\sim Half-Cauchy\;\left(0,0.5\right)$$

These are moderately informative priors, which also allow for large between-study heterogeneity (Williams et al., 2018; Röver et al., 2021; Harrer, 2022). All other parameters had default brms priors.

Multilevel models with cell means parametrization were used when a categorical predictor was included in the model (Park and Beretvas, 2019). A minimum of two studies per test or subdomain were required to calculate a meta-regression. In considering the moderator variables, a minimum of five studies per moderator variable were required (Borenstein, 2009; Harrer, 2022). Continuous moderators were not mean centered and scaled. VaD subtypes were compared using *post hoc* comparisons and the hypothesis function of the brms package. We included VaD subtype as a predictor variable, if at least two studies per VaD subtype were available. With increasing number of studies, difference in dementia severity, education, age, and percentage of women between the AD and VaD subtype were further predictors included into the analysis. If needed, the covariates were excluded if the variance inflation factor was greater than 5. We present the results of all analyses in tables and forest plots.

### 2.7. Sensitivity analyses of prior distribution choice

In the sensitivity analyses on the choice of the prior distribution for regression coefficients, we used a Student-*t* prior with three degrees of freedom:


$$\beta_{0},\beta_{i}\sim St\;\left(0,1,3\right)$$

A standard normal prior:


$$\beta_{0},\beta_{i}\sim N\;\left(0,1\right)$$

and a uniform prior a broad effect size range:


$$\beta_{0},\beta_{i}\sim U\;\left(-10,10\right)$$

For the τ parameters, we used:


$$\tau_{Study},\tau_{Study/ES}\sim Half-Cauchy\;\left(0,0.5\right)$$

$$\tau_{Study},\tau_{Study/ES}\sim \exp\;\left(1\right)$$

$$\tau_{Study},\tau_{Study/ES}\sim InverseGamma\;\left(0.501,0.501\right)$$

For each student-*t*, standard normal and the uniform prior models. The inverse gamma prior is a uniform prior on a large range of τ values. The results of these sensitivity analyses are presented in Supplementary Material 3.

### 2.8. Study quality sensitivity analyses

Sensitivity analyses pertaining to study quality were conducted for each domain by rerunning the analyses using only studies rated at least three points on the Selection, at least one point on the Comparability and at two points on the Exposure Scale of the NOS. It was not possible to conduct sensitivity analyses for apraxia, processing speed, reasoning measures, global functioning, disease awareness, measures of intelligence, language comprehension, reading, motor functioning, orientation to time and space, perception, and social functioning. While these analyses investigate the impact of study quality on effect sizes, they also resulted in a drastic reduction in statistical power. The results of these sensitivity analyses are presented in Supplementary Materials 1, 2.

### 2.9. Certainty assessment

The advantage of the Bayesian approach is the direct expression of uncertainty in the posterior distribution of the parameter estimates. Thus, synthesizing studies reporting large effect sizes with high variability will lead to a large estimated effect size with a wide posterior distribution. The width of the credibility interval obtained from the posterior distribution is then a quantitative measure of the uncertainty about the effect size. Further, the Bayes Factor expresses the ratio between the evidence for the existence of the effect compared to the *a priori* expected differences. The magnitude of this ratio is the degree of evidence for the existence of a difference between the groups. The certainty of evidence can then be judged based on the magnitude of the Bayes Factor and the width of the credible interval (Gelman et al., 2013; Gelman and Carlin, 2014; Kruschke, 2015), with higher Bayes Factors and narrower credible intervals denoting higher degree of certainty.

## 3. Results

To be concise, we presented only the results from language production, apraxia, motor functioning, perception, visuo-spatial processing, attention, executive functioning, memory domains, and risk of bias assessment in the main article. All other information including data analysis files is available in the Supplementary Material 1.

### 3.1. Risk of bias assessment: median Newcastle-Ottawa scale scores

In the table below, we present median scores on the three NOS scales. While selection scores for most domains show a high patient selection transparency, the scores for the Comparability and Exposure scales indicate incomplete reporting of information on comparability of patient and control groups, blinding and experimental treatments.

### 3.2. Apraxia and motor functioning

Based on three studies with 189 patients, we found anecdotal evidence for lower apraxia scores in AD patients compared to VaD patients ( $\beta_{g}$ = 1.11, 95% *ETI* [−0.35, 2.18], BF = 2.02). For facial and ideomotor apraxia, no differences were found. Based on three studies with 171 subjects, we found anecdotal evidence for better motor functioning of AD patients ( $\beta_{g}$ = 0.66, 95% *ETI* [−0.03, 1.42], BF = 2.66).

### 3.3. Attention

In the attention domain, results from two studies involving 232 patients provided anecdotal evidence for better performance of AD patients as compared to sVaD patients on the Symbol Digit Modalities Test ( $\beta_{g}$ = 0.46, 95% *ETI* [−0.17, 1.02], BF = 1.26). No other differences were found. In fact, we found evidence against differences in selective attention, sustained attention, and visual attention measures (all BF < 0.27; Table 3).

**Table 3:** Results in domains showing the greatest differences between AD patients and VaD subtypes.

VaD Subtype	Domain	*k*	*n*	$\beta_{g}$ [95% *ETI*]	Bayes factor	$\tau_{Study}$	Newcastle-Ottawa scale		
							S	C	E
VaD	Digit span backwards ^S, E,^	7	225	**0.33 [0.12, 0.52]**	9.38	0.05	3	1	1
	Delayed recall: Word list ^S, E^	4	235	−0.61 [−0.97, −0.26]	22.71	0.18	3	0	1
	Wechsler memory scale verbal recognition	2	85	−1.12 [−1.75, 0.04]	4.72	0.42	3	1	1
	CERAD: Word list recognition	4	228	−0.92 [−1.38, −0.43]	19.21	0.27	3	1	1
	Other measures of visual memory^S^: Delayed recall	5	177	−0.85 [−1.29, −0.32]	13.67	0.18	3	1	1
	Immediate visual associative memory	4	354	−1.01 [−1.49, −0.43]	20.02	0.25	3	1	1
sVaD	Phonemic fluency ^S, E, A, G^	17	1,460	**0.51 [0.22, 0.77]**	42.36	0.40	3	1	1
	Basic ADL^S, E, A, G^	3	290	−1.73 [−3.63, 0.01]	4.14	1.92	3	0	1
	Graphical sequence test	2	152	**0.82 [0.02, 1.47]**	3.11	0.36	3	0	1
	Delayed recall: Prose	4	670	−0.70 [−1.12, −0.27]	13.11	0.30	3	1	1
	Delayed recall: Word list ^S, E^	7	805	−0.64 [−0.88, −0.36]	72.97	0.18	3	1	1
	Discriminability in verbal memory	4	489	−0.76 [−1.26, −0.26]	11.50	0.28	3	0	1

Positive effect sizes denote better performance of AD groups and are marked in bold. VaD, Vascular dementia; sVaD, Subcortical vascular dementia. Domain superscripts denote moderators included in the analyses: differences in average dementia severity (S), years of education (E), age (A) and proportion of women (G). k, Number of studies; *n*, Number of patients; 95% ETI, 95% equal-tailed credible interval. Newcastle-Ottawa-Scale Subscales medians: Selection (S), Comparability (C), Exposure (E). ADL, Activities of daily living. $\tau_{\mathrm{Study}},$ Standard deviation of random effects between studies. CERAD, Consortium to Establish a Registry for Alzheimer’s disease Test Battery.

### 3.4. Executive functioning

Five studies reported Mental Control and Accuracy indices from the Wechsler Memory Scale (WMS). The synthesis of the results across 247 patients shows anecdotal evidence for better performances of AD patients ( $\beta_{g}$ = 0.48, 95% *ETI* [0.07, 0.82], BF = 2.68). We also found anecdotal evidence for better performance of AD on the digit span backwards measure from the WMS ( $\beta_{g}$ = 0.44, 95% *ETI* [0.07, 0.83], BF = 2.51). For other digit span backwards measures, we found a moderating effect of the VaD subtype. While the performances of AD, sVaD ( $\beta_{g}$ = 0.14, 95% *ETI* [−0.02, 0.33], BF = 0.298) and MID patients ( $\beta_{g}$ = 0.42, 95% *ETI* [−0.07, 0.89], BF = 0.819) were undistinguishable, AD patients outperformed VaD patients ( $\beta_{g}$ = 0.33, 95% *ETI* [0.12, 0.52], BF = 9.38). There was also moderate evidence from two studies with 152 patients for better performance of AD compared to sVaD on the Graphical Sequence Test ( $\beta_{g}$ = 0.82, 95% *ETI* [0.02, 1.47], BF = 3.11). Three studies with 413 patients using other measures of verbal working memory also provided anecdotal evidence for better performance of AD patients ( $\beta_{g}$ = 0.46, 95% *ETI* [0.04, 0.80], BF = 2.66).

### 3.5. Visuo-spatial processing

**Figure 2:**
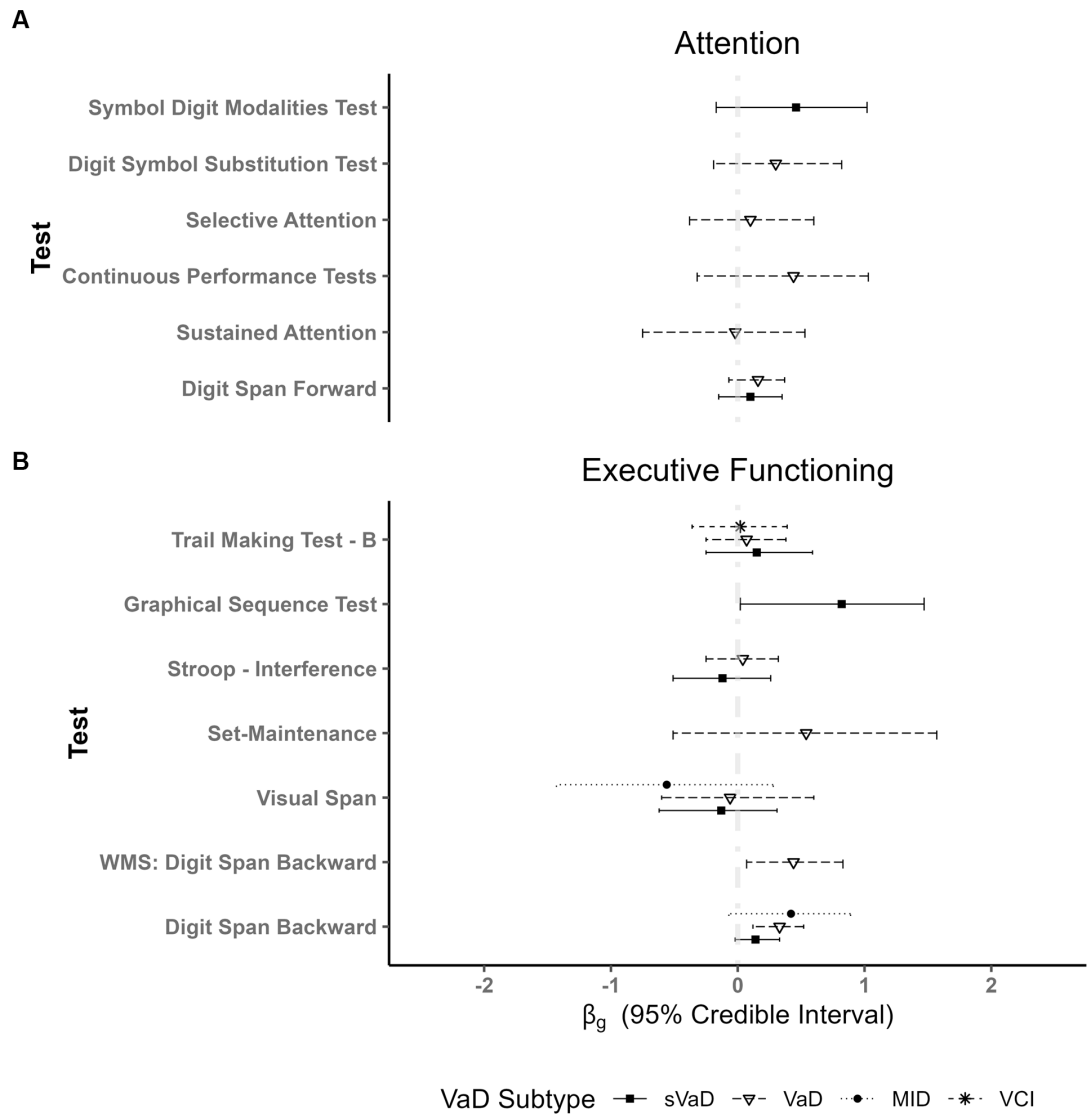
Plot showing fixed effects coefficients with their respective 95% *ETI* for different types of vascular dementias as compared to AD for different tests of: **(A)** Attention and **(B)** Executive Functioning. AD patient groups performed better than VaD patients only in digit span tasks (WMS and other digit span tests). Importantly, there are no differences in respect to other VaD subgroups. WMS = Wechsler Memory Scale.

We only found anecdotal evidence for worse performance of AD patients compared to VaD patients on the Judgment of Line Orientation Test ( $\beta_{g}$ = −0.96, 95% *ETI* [−1.83, 0.36], BF = 1.94). In fact, we found evidence against performance differences on the Rey Osterrieth Complex Figure Test (RCFT) – Copy (sVaD BF = 0.117, VaD BF = 0.086), WAIS: Block Design (sVaD BF = 0.241, VaD BF = 0.114), and the Clock Drawing Test (BF = 0.188; Figure 2).

### 3.6. Language production

**Figure 3:**
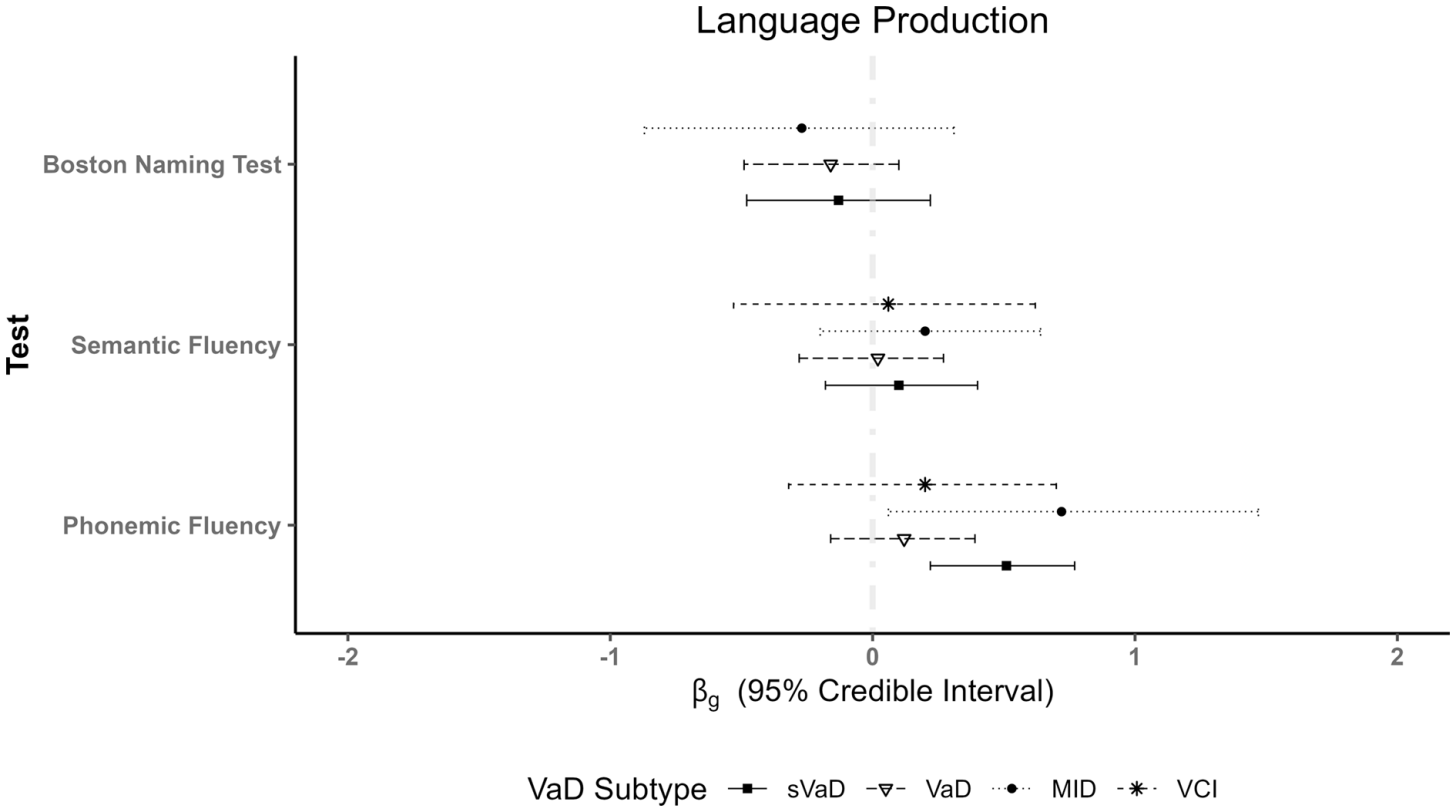
Plot showing fixed effects coefficients with their respective 95% *ETI* for language production measures. Whereas there are no differences in performances on semantic fluency measures, we see a clear advantage of AD patients in phonemic fluency when compared to multi-infarct dementia (MID) and subcortical vascular dementia (sVaD).

For phonemic fluency, we found better performances of AD patients compared to MID ( $\beta_{g}$ = 0.72, 95% *ETI* [0.06, 1.47], BF = 2.66) and sVaD patients ( $\beta_{g}$ = 0.51, 95% *ETI* [0.22, 0.77], BF = 42.36). Additionally, VaD patients outperformed sVaD patients (*M*_Difference_ = 0.38, SD = 0.18, see Figure 3). For semantic fluency, the Boston Naming Test, and other measures of language production we found no differences between AD and VaD subtypes (all BFs < 0.5).

### 3.7. Memory

**Figure 4:**
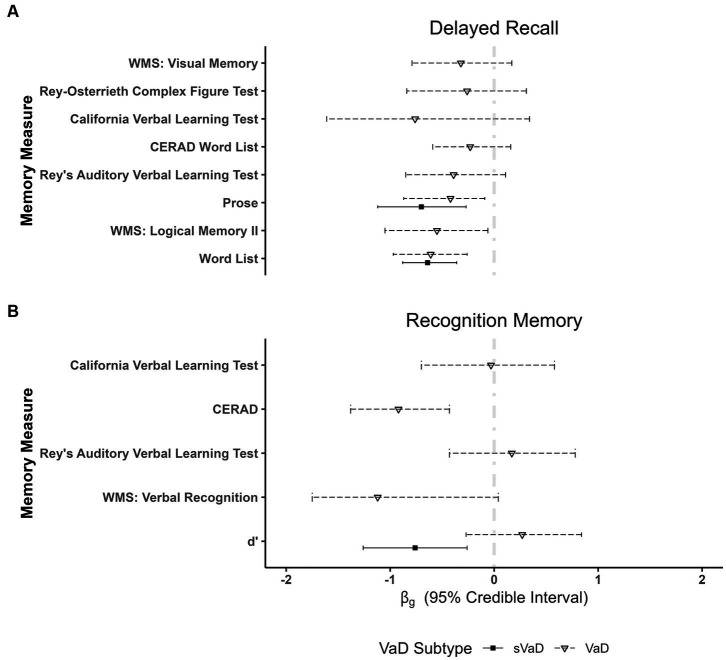
Plots showing fixed effects coefficients with their respective 95% *ETI* for **(A)** delayed recall of verbal and visual material as well as for **(B)** recognition memory. *d’* measures imply a cortical–subcortical division between dementia forms, with similarly impaired performances of VaD and AD and significantly better performances of subcortical VaD (sVaD) patients in verbal recognition memory tasks. WMS, Wechsler Memory Scale; CERAD, Consortium to Establish a Registry for Alzheimer’s disease.

Key results from the memory domain are represented in Figure 4, with full results available in Supplementary Table 21 of Supplementary Material 1. In verbal episodic memory, we found strong evidence for better performances of sVaD patients in delayed recall of prose ( $\beta_{g}$ = −0.70, 95% *ETI* [−1.12, −0.27], BF = 13.11). There was also anecdotal evidence for VaD patients outperforming AD patients on these tasks ( $\beta_{g}$ = −0.42, 95% *ETI* [−0.87, −0.09], BF = 2.47). Similarly, sVaD ( $\beta_{g}$ = −0.64, 95% *ETI* [−0.88, −0.36], BF = 72.97) and VaD ( $\beta_{g}$ = −0.61, 95% *ETI* [−0.97, −0.26], BF = 22.71) patients have been shown to outperform AD patients in delayed recall of word lists. In delayed recall of word lists, we also found strong evidence for a moderating effect of the difference in dementia severity ( $\beta_{g}$ = 0.36, 95% *ETI* [0.17, 0.56], BF = 11.48). In verbal recognition memory, sVaD patients have higher *d’* ( $\beta_{g}$ = −0.76, 95% *ETI* [−1.26, −0.26], BF = 11.50). VaD patients also outperformed AD patients in verbal recognition memory measures from the CERAD test battery ( $\beta_{g}$ = −0.92, 95% *ETI* [−1.38, −0.43], BF = 19.21).

In meta-regressions on recall and recognition in visual episodic memory, we found strong evidence for worse performance of AD compared to VaD patients in immediate recall in visual associative memory ( $\beta_{g}$ = −1.01, 95% *ETI* [−1.49, −0.43], BF = 20.02) and in delayed recall of visual information ( $\beta_{g}$ = −0.85, 95% *ETI* [−1.29, −0.32], BF = 13.67). We found no differences between AD and vascular dementia groups for delayed recall on two standard tests of visual episodic memory, the Rey-Osterrieth Complex Figure Test ( $\beta_{g}$ = −0.26, 95% *ETI* [−0.84, 0.31], BF = 0.369) and the Wechsler Memory Scale: ( $\beta_{g}$ = −0.32, 95% *ETI* [−0.79, 0.17], BF = 0.552).

Finally, we also found moderate evidence for better performance of VaD patients in semantic memory measures including tasks such as a famous faces test, word-picture matching, word definitions, a picture sorting test, and a stem completion priming task (four studies, n = 179, $\beta_{g}$ = −0.79, 95% *ETI* [−1.47, −0.09], BF = 3.85).

### 3.8. Other domains

No differences were found in measures of global functioning, orientation to time and space, disease awareness, depression, state and trait anxiety, other BPSD, intelligence measures, processing speed, language comprehension, reading, reasoning abilities, and ADL.

### 3.9. Results of individual studies and risk of bias assessment results

Overall, the risk of bias assessment shows that most studies used adequate case definitions. Most studies failed to match the dementia groups in the study design on relevant characteristics or to include these confounders as moderators in the analyses. Furthermore, most studies did not report the non-response rate or have not described the non-responders. The greatest limitations of the existing studies thus are the comparability of dementia groups especially on dementia severity, but also premorbid intelligence, education, age, and gender ratios. More information regarding the exact selection process of participants should also have been reported. The Newcastle-Ottawa-Scale ratings and the inspection of funnel plots however indicated no strong selective reporting tendencies.

### 3.10. Sensitivity analyses of prior distribution choice

The results of these sensitivity analyses are presented graphically in Supplementary Material 3. While most prior settings had no meaningful influence on the estimated regression coefficients and dispersions, the inverse gamma priors did allow for higher variability estimates. These were however not significantly different from τ estimates based on other priors, as there was still considerable overlap between confidence intervals. All in all, the results and their interpretations remain unchanged regardless of the prior choice.

### 3.11. Sensitivity analyses to quality of studies

Excluding studies with low NOS ratings did not change the patterns of results for ADL (Supplementary Table 3 in Supplementary Material 1), Depression and Anxiety severity (Supplementary Table 6 in Supplementary Material 1), severity of Neuropsychiatric Symptoms (Supplementary Table 5, p. 11 in Supplementary Material 1), Constructional Praxis (Supplementary Table 7 in Supplementary Material 1), Digit Span Forward (Supplementary Material 1, p. 20), Digit Span Backward, Visual Working Memory or Cognitive Flexibility (Supplementary Table 16 in Supplementary Material 1). The analysis for delayed recall of verbal material showed no difference between AD and VaD groups ( $\beta_{g}$ = −0.01, 95% ETI [−0.66, 0.67], BF = 0.421), which does differ from the results obtained in the main analyses. This is however most likely due to reduced statistical power since the sensitivity analysis included only two studies and thus did not allow controlling for confounding effects of differences in dementia severity and years of education as in the main analyses.

## 4. Discussion

We performed a systematic review and a quantitative synthesis of the findings on the utility of cognitive, functional and BPSD diagnostic instruments in the process of differential diagnostics of AD and VaD. We quantified the strength of evidence for individual tests and domains using Bayesian meta-regressions. In extending the existing reviews and meta-analyses, we not only included new studies, but also compared AD to several VaD subtypes in an attempt to draw a more differentiated picture of diagnostic issues in research and clinical practice (Almkvist, 1994; Looi and Sachdev, 1999; Oosterman and Scherder, 2006; Mathias and Burke, 2009). Our findings from the systematic review and meta-regressions identify similar patterns of cognitive impairment and suggest worse performance of AD patients on measures of episodic and semantic memory. On the other hand, the narrative review implies greater impairment of executive functioning in VaD, such as working memory, set-maintenance, response inhibition, conceptualization, and planning. Yet, our meta-regressions only support worse performances of VaD patients in verbal working memory and letter fluency. Importantly, we also found evidence for differences between VaD subtypes, which underlines the importance of accounting for the etiology of cerebrovascular pathology in clinical praxis and research.

### 4.1. AD patients are superior in verbal working memory and other executive functions

We found that AD outperform VaD patients in verbal working memory tasks such as the WAIS digit span backward task. AD patients also produced more words beginning with a certain letter in phonematic fluency tasks, e.g., the Controlled Word Association Test, when compared to MID (BF = 2.66) and sVaD (BF = 42.36) patients. However, we found moderate evidence for no difference between AD and VaD (BF = 0,109).

These findings imply the relative preservation of information maintenance and simultaneous application or manipulation of the contents of the working memory in AD, when compared to MID and sVaD. Our results are also in line with previous reports, that the acquisition of a task set is difficult for AD patients, but its maintenance and application are relatively preserved so that AD patients can produce more words or are able to reverse the order of a longer number sequence than VaD patients (Lamar et al., 2002). Indeed, phonemic fluency can be seen as requiring a number of executive processes such as initiating and maintaining a set, short-term memory, organizational strategies and cognitive flexibility as well as the inhibition of incorrect responses (Poore et al., 2006). On the level of neural processing, these results suggest greater impairment of frontal-striatal-thalamic circuits in MID and sVaD (Alexander et al., 1986; Frank et al., 2001; O’Reilly and Frank, 2006; Hazy et al., 2007).

### 4.2. AD patients display greater impairment of memory function

On the contrary VaD patients outperformed AD patients in verbal episodic memory tests, in which patients had to recall a list of words (e.g., AVLT) or a paragraph of text (e.g., WMS: Logical Memory). AD also displayed increased forgetting of word lists and paragraphs. Likewise, sVaD patients outperformed AD patients in recall of word lists. VaD and sVaD groups also outperformed AD patients in visual associative memory measures such as a location memory test, WMS: visual paired association, a placing test, the visual association memory test, and a location learning test. sVaD patients also outperformed AD patients in the delayed recall of visual information [e.g., RCFT, The Doors and People Test, Test of Classification and Recall of Pictures, WMS-III, NEUROPSI (Ostrosky et al., 1999—Figure Recall]. In verbal recognition memory, sVaD patients also outperform AD patients on a broad set of tests (such as the California Verbal Learning Test, Ray’s Auditory Verbal Learning Test, CERAD cognitive test battery, Free and Cued Selective Reminding Test, WMS, and The Doors and People Test). In sum, we found moderate to very strong evidence for better performance of sVaD and VaD patients in episodic memory tests.

The difference in episodic memory impairment between AD and vascular dementias also grows larger in the course of the disease (Smits et al., 2015). Deficits on these memory measures point to deficits in encoding (Stamate et al., 2020), recall and retention of verbal and visual information (Larrabee et al., 1993; Kramer et al., 2004; Hildebrandt et al., 2009; Peña-Casanova et al., 2012). These deficits also imply impairments of the processes of pattern separation and completion in the hippocampus (McClelland et al., 1995; O’Reilly and Rudy, 2000; Healey et al., 2016; Rolls, 2016, 2021) and are in line with the known pattern of cerebral pathology in AD reviewed in Section 1.1 (Braak and Braak, 1991; Carlesimo et al., 1993). For example, it has been suggested that the observed liberal bias in recognition memory tasks in AD patients results from the accumulation of amyloid β-plaques in the limbic system, which lead to lower specificity of neural responses to external stimuli when encoding new information (Hildebrandt et al., 2009; Biesbroek et al., 2015). Criterion adaptation in recognition memory on the other hand involves the striatum and frontal regions (Kuchinke et al., 2011; Biesbroek et al., 2015), both of which are also affected by Alzheimer’s pathology (Thal et al., 2002).

### 4.3. Influence of VaD and AD subtypes

The differences in recognition accuracy in verbal recognition memory and to some extent letter fluency (see above) also divide cortical from subcortical pathologies (Vanderploeg et al., 2001). This would suggest that pathologies involving the cortex lead to impaired memory representations (Rolls, 2018), whereas sVaD patients have recall deficits, presumably due to white matter lesions affecting white matter tracts subserving “frontal” functioning, including memory recall (Squire, 1992; Ward, 2003; Jeneson and Squire, 2012; Biesbroek et al., 2017; Richter et al., 2017; Alber et al., 2019). Compared to free recall, recognition tasks reduce the demands on recall and in turn lead to an improved performance. On the contrary, due to the degradation of the to-be-remembered stimuli in cortical pathologies even the repeated presentation of the stimulus does not enable the successful discrimination of learned from new items (Weigard et al., 2020). The cortical vs. subcortical division of dementias, is an important issue for future research, as it suggests that brain areas affected by cortical atrophy, altered metabolism and brain lesions might be more relevant than the etiology of dementia in determining the cognitive profile and course of the disease (Sachdev et al., 2014; Dronse et al., 2016; ten Kate et al., 2018). Indeed, a direct comparison of subcortical and cortical pathologies, based on the data from Vanderploeg et al. (2001) for example, offers anecdotal evidence for inferior performance of patients with cortical VaD (*n* = 12) compared to patients with sVaD (*n* = 16) on the discriminability measure from the CERAD recognition memory test [*t*(17) = −2.23, BF = 2.08, *d* = −0.66 95% *CI* [−1.45, 0.04]]. That is, VaD patients have a potentially lesser ability to discriminate between old and new items due to a smaller difference between the means of the memory and noise distributions (Snodgrass and Corwin, 1988; Stanislaw and Todorov, 1999). These results underscore the importance of accounting for the studied VaD subtypes and also stress the need for future studies to account for AD phenotypes—such as posterior cortical atrophy or fronto-temporal variant of AD (Murray et al., 2011; Noh et al., 2014; Dong et al., 2016; ten Kate et al., 2018)—when investigating differences between AD and VaD.

### 4.4. Visuo-spatial processing is less impaired in VaD

Vascular dementia patients also outperformed AD patients in visuo-spatial processing including tasks such as the Visuo-Spatial Perception Battery, Line Bisection, Judgment of Line Orientation, Hooper’s Test, Overlapping Figures, and WAIS: Picture Completion and the Battery for Visuospatial Abilities. In sum, problems with spatial representation and transformation provide anecdotal to moderate evidence for the presence of AD and the parietal hypometabolism and parietal pathologies present in AD (Engel et al., 1993; Almkvist, 1994; Looi and Sachdev, 1999; Bischof et al., 2016; Dronse et al., 2016; ten Kate et al., 2018).

### 4.5. No support for differences in other domains and measures

In partial disagreement with DSM V and ICD 10/11 and guidelines for differential diagnoses (Hachinski et al., 2006; Skrobot et al., 2018), we found anecdotal to moderate evidence for comparable performances of AD and VaD on measures of processing speed, reasoning, simple and complex attention as well as on BPSD measures. Our findings thus call for caution when these measures are used to distinguish the two dementia forms as their discriminatory ability appears to be not as strong as previously believed. The high uncertainty of the results for the measures of basic ADL precludes any conclusions about the differences regarding the ability to master basic ADL.

### 4.6. Our findings in the context of previous reviews

Like previous reviews (Looi and Sachdev, 1999; Oosterman and Scherder, 2006; Mathias and Burke, 2009, but see Almkvist, 1994), we identified tests which can help neuropsychologists make informed contributions to the process of differential diagnosis of AD and VaD. While we find support for differences in aspects of executive functioning (digit span backward, phonemic fluency) and episodic memory, we depart from the specific tests proposed by Looi and Sachdev (1999) and Oosterman and Scherder (2006). By conducting meta-regressions and including moderator variables in our analyses, we even found evidence for no difference in tests such as the Wisconsin Card Sorting Test. Among memory tests, we found evidence for differences not only in delayed recall and recognition of verbal material, but also evidence for worse performance of AD patients on tests of associative and visual memory. Our results thus expand the toolbox of tests which may help neuropsychologists distinguish AD and VaD. We also showed that sVaD and VaD display distinct differences when compared with AD on cognitive measures (*cf.* Oosterman and Schreder, 2006). Whereas VaD patients performed worse on the digit span backward test, a measure of verbal working memory, sVaD patients performed worse on measures of cognitive flexibility and phonemic fluency. Moreover, by including demographic variables and dementia severity as predictors in many meta-regressions, we were able to statistically control for their influence on the estimated differences between the dementia subtypes. While we included demographic and clinical variables such as gender, age, education and severity of dementia, factors such as time since diagnosis or diagnostic criteria were not considered in the analyses. The effects of diagnostic criteria and disease duration on differences in cognitive and functional impairment therefore remain questions for future research.

### 4.7. Implications for the clinical praxis

To obtain a cognitive profile with differential diagnostic utility, Hayden et al. (2005) for example suggested that neuropsychologists should use tasks requiring flexible behavior, response inhibition and sensorimotor integration to assess executive functioning and tasks with high encoding (prose recall) and consolidation (delayed recall) demands. Further, they suggest including a recognition memory task, because VaD patients perform better in recognition memory than in free recall due to reduced recall demands. Given the fact that recognition memory also includes executive processes, tests that allow to disentangle criterion setting, episodic memory, and stored long-term memory information should be helpful (Hofmann et al., 2011; Kuchinke et al., 2011; Hofmann and Jacobs, 2014). Results of our meta-regressions also advocate the inclusion of visual memory and associative memory tests. In the assessment of executive functioning, phonemic fluency and digit span appear to be superior to tasks involving motor responses, cognitive flexibility, and response inhibition. An example examination could thus include the following: WAIS Digit Span, RAVLT, RCFT, COWAT, immediate recall from a visual associative memory test. Of course, other tests should be included according to the demands of the idiosyncrasy of individual patient’s cognitive complaints.

## 5. Outlook

Our findings suggest the ability of established cognitive measures to differentiate AD from VaD on a group level, yet their utility in distinguishing between AD and VaD subtypes in individual patients remains very limited as all effect sizes’ magnitude was inside the region of practical equivalence. These are nonetheless promising results which should motivate future work by drawing the focus on specific cognitive processes involved in tasks such as digit span backward, phonemic fluency, delayed recall, or associative and recognition memory. Our findings underscore the need for the development of new diagnostic instruments and new scoring systems, which better measure specific cognitive processes (Batchelder et al., 1997; Chosak Reiter, 2000; Davis et al., 2002; Todorova et al., 2020). For example, detailed scoring methods deepened the understanding of impaired performances on the Boston Naming Test (Bayles and Tomoeda, 1983; Laine et al., 1997; Lukatela et al., 1998; Chosak Reiter, 2000) and word fluency tasks (Clark et al., 2014a,b, 2016; Pakhomov et al., 2016). The resulting improved validity and reduced measurement error could thus lead to the identification of cognitive processes, which are differentially impaired in VaD and AD. Finally, they should lead to a consolidation of the verbal theoretical models of cognitive function in both dementias. Furthermore, set acquisition and maintenance are two abilities, which need to be investigated more thoroughly and more often in differential diagnoses. Measures of semantic dedifferentiation which are as free as possible of cultural and educational bias are also needed. Finally, the information provided by such measures would also have important implications for patients and their caregivers. Due to a more complete description of each patient’s strengths and weaknesses they could better inform treatment strategies, potential therapies and coping strategies of patients and caregivers.

Further, formulating and transferring mechanistic models of memory functioning (Hofmann and Jacobs, 2014; Cox and Shiffrin, 2017; Kahana, 2020) or decision-making (Ratcliff and Smith, 2004; Tillman et al., 2020; Weigard et al., 2020) into clinical praxis would address the lack of such models in the research on AD and VaD, which we identified in the introduction. Such models are especially promising, since they can assess patients’ performance at the level of cognitive constructs of interest such as speed of processing, quality of neural input, contributions of semantic and episodic memory to free recall, and criterion setting in recognition memory and attentional or perceptual task. For instance, response bias may be manipulated by constructing recognition memory tasks consisting of sub-blocks containing 80 vs. 60 vs. 40% “old” items to examine conditions inducing a liberal vs. conservative response bias. At the level of cognitive constructs, cognitive architectures such as ACT-R (Ritter et al., 2019) offer an as of yet unexhausted potential for explicit modeling of impaired cognitive processes and their interactions as observed in VaD and AD patients.

The second, yet not necessarily independent, avenue is using knowledge of functional and structural neuroanatomy as well as brain inspired computational principles in the development of new explanatory models (Shuttleworth and Huber, 1989; O’Reilly and McClelland, 1994; O’Reilly and Rudy, 2000; Rolls, 2016, 2021), clinical measures, scoring techniques and even individualized assessments (Schroeter et al., 2020) which account for the heterogeneity of both AD and VaD subtypes. Incorporating these information sources would thus address the underspecified nature of existing measures. Development of these new approaches can rely on databases such as Neurosynth^2^ or NeuroVault.^3^ The importance of firm neuroanatomical basis of cognitive measures is exemplified by reports suggesting that memory tests recommended for the diagnosis of AD (Costa et al., 2017) may actually lack specificity for the disease as they may be more sensitive to memory dysfunctions associated with late hippocampal stages rather than the earliest, stages of the disease, when neurofibrillary tangles are present in the entorhinal cortex, the CA1, basal forebrain, and the antero-dorsal thalamic nucleus (Braak and Braak, 1991; Thal et al., 2002, 2008; Jonin et al., 2019; Richter et al., 2020). A relatively new cognitive measure putatively relying on these brain structures is the conjunctive binding task (Della Sala et al., 2012) which shows promise in discriminating AD from other primary dementias. Other tasks, for example testing the theory that A*β*-plaques lead to reduced quality of neural representations, which lead to a positive bias in recognition memory (Hildebrandt et al., 2009) by assessing response bias for example by manipulating semantic associations between words or visual similarity of items etc., should also be developed (e.g., Hofmann and Jacobs, 2014). With deterministic simulations of episodic memory signals, response criterion setting may even be directly estimated from the data (Hofmann et al., 2011; Kuchinke et al., 2011; Pooley et al., 2011; Starns et al., 2013). For this endeavor, tasks manipulating criterion adaptation in (executive and) memory tasks are needed, to establish domain specific biases in cognitive processing (Mendez et al., 1997; Yuspeh et al., 2002). Moreover, the interactivity of different levels of representation can be directly tested in functional connectivity analyses (Roelke and Hofmann, 2020). Informing and enriching the development of such tasks by neuroanatomical knowledge will only lead to a higher quality of diagnostic instruments (Palestro et al., 2018).

On the other hand, there are studies showing the utility of reading the brain-behavior correlation in reverse (Price et al., 2005, 2012, 2015). Authors following this approach first use a statistical grouping technique (e.g., *k*-means clustering) to define groups based on their performance on neuropsychological tests or composite measures. Then, group differences on neuroradiological measures are investigated. In this manner, a connection between neuroradiological characteristics and cognitive phenotype can be established. Using such an approach in a mixed AD and VaD sample, Price et al. (2015) identified three phenotypes of cognitive impairment: amnestic (34 AD and 7 VaD patients), multi-domain (14 AD and 12 VaD patients) and dysexecutive (5 AD and 21 VaD patients). The comparison of these groups on neuroradiological measures showed higher white matter lesion volumes in the dysexecutive and multi-domain phenotypes and lower hippocampal volume in multi-domain and amnestic groups, when compared to the dysexecutive phenotype. The implication of this research is that AD and VaD should be thought of as two entities from a clinical and pathological spectrum (Emrani et al., 2020).

Both approaches, neuroanatomy first and cognition first, can be brought together in the framework of joint modeling of neural and cognitive processes (Forstmann and Wagenmakers, 2015; Palestro et al., 2018) that we mentioned already in the introduction. Future studies in differential diagnosis of AD and VaD, as all studies aspiring to elucidate brain-behavior relations in neurocognitive disorders, should try to formulate a neurocognitive model, which simultaneously and interactively models the relationship between neuroanatomy and cognitive performance.

## 6. Limitations

The limitations of our meta-analyses can be divided in at least two categories. First, we only included studies which provided a minimal, satisfactory description of included patients. This led to the exclusion of many studies conducted in this field and led to a large variation in the number of studies per domain—ranging from two studies (e.g., disease awareness) to 36 studies in verbal fluency. Therefore, the power to detect a meaningful difference varied across domains thereby limiting the confidence in our conclusions of no reliable differences between AD and VaD. Regretfully, most included studies still failed to assure comparability of their cohorts. Thus, as shown by our sensitivity analyses, the results of meta-regressions should be interpreted with caution, whenever we could not control for the differences in dementia severity, average age, years of education, and proportion of women. We could also not consider moderators such as disease duration, depression scores, premorbid intelligence, measures of brain atrophy, or white matter lesions volume since they were too rarely reported. Further, we could not assess the influence of variables such as sampling method, diagnostic procedure, and population type, all of which are important and relevant variables when investigating differences in patterns of cognitive impairment between AD and VaD. A referral bias—with patients presenting mostly because of memory complaints–is also an issue since it reduces the ability of studies to characterize VaD specific cognitive impairments (Corey-Bloom et al., 1993). Lastly, the unknown locations and spatial distributions of stroke lesions in VaD and MID groups as well as the likely inclusion of mixed dementia patients in both the AD and VaD groups can also have a significant impact on the profile of cognitive impairments and thus on the magnitude and variance of the observed effect size.

Other limitations stem from our decision on data extraction and synthesis. First, the pooling of cognitive performances in studies which divided the sample according to disease severity can distort the differences in cognitive measures. Second, a severe limitation of our approach is that we could only calculate the utility of single cognitive measures to differentiate AD from VaD subtypes, whereas clinical decisions are made based on medical information and a full cognitive profile with relative levels of impairment within a given patient. Since only one study shared the single case data, no analyses investigating individual cognitive profiles were possible. Further, new or rarely used tests had to be analyzed as representing a certain domain or a group of tests denoted as “other.” This inevitably leads to the apples and oranges problem, i.e., comparing tests which might not assess the same aspects of the selected domain. Finally, there are also other important differential diagnoses such as cognitive impairment in affective and psychiatric disorders as well as other causes of dementia, which we did not consider here.

## 7. Conclusion

We found only partial support for the typical cognitive impairments as described in the DSM-V, the ICD-11 and in the guidelines on the differential diagnoses. Our results show strong support for better performance of patients with vascular dementia on episodic and semantic memory tests as well as evidence for better performance of AD patients on two measures of executive functioning: digit span backward and phonemic fluency. On the contrary, we found no or only weak support for differences in attention, processing speed, reasoning, apraxia, or motor impairment. In clinical, praxis cognitive testing should continue to be complemented by brain imaging, CSF markers, and caregiver reports to reach a differential diagnosis. On the other hand, neuropsychological research must develop new theoretical and mechanistic, computational models of dementia subtypes, which are based on neuroanatomical findings and are able to reproduce qualitative and quantitative patterns of impaired cognitive processes in various forms of dementia as well as of normal functioning in older adults.

## Data availability statement

The original contributions presented in the study are included in the article/Supplementary material, further inquiries can be directed to the corresponding author.

## Author contributions

LS: Writing – original draft, Writing – review & editing, Conceptualization, Formal analysis, Methodology, Visualization, Investigation. MH: Writing – review & editing, Supervision. NM: Data curation, Investigation, Validation, Writing – review & editing. JK: Writing – review & editing, Supervision.

## Footnotes

^1^ http://www.ohri.ca/programs/clinical_epidemiology/oxford.asp

^2^ https://neurosynth.org/

^3^ https://neurovault.org/
