# Supplementary material for: Neuropsychological differential diagnosis of Alzheimer’s disease and vascular dementia: a systematic review with meta-regressions
Source: Front Aging Neurosci. 2023 Nov 6;15:1267434. doi: 10.3389/fnagi.2023.1267434 (PMC10657839; doi:10.3389/fnagi.2023.1267434)
Supplement: Supplementary file 1 [file Table_1.DOCX]

**Neuropsychological Differential Diagnosis of Alzheimer’s Disease and Vascular Dementia: A Systematic Review with Meta-Regressions**

**Supplementary Information**

Leo Sokolovic^1,2,3,*^, Markus J. Hofmann^3^, Nadia Mohammad^3^, Juraj Kukolja^1,2^

1 Department of Neurology and Clinical Neurophysiology, Helios University Hospital Wuppertal, Wuppertal, Germany

2 Faculty of Health, Witten/Herdecke University, Witten, Germany

3 Department of General and Biological Psychology, University of Wuppertal, Wuppertal, Germany

* Corresponding author: leo.sokolovic@outlook.com

ORCID

Leo Sokolovic: 0000-0002-7449-925X

Nadia Mohammad: 0000-0001-7566-9516

Juraj Kukolja: 0000-0003-1569-3287

Markus J. Hofmann: 0000-0001-9812-4121

# Supplementary Materials 1: Meta-Regression Analyses Tables

Table 1. Results of Meta-Regressions for Global Functioning Measures 3

Table 2. Results of the Meta-Regression for Orientation to Time and Space 4

Table 3. Results of the Meta-Regression for the Basic and Instrumental Activities of Daily Living 5

Table 3. Results of the Meta-Regression for the Basic and Instrumental Activities of Daily Living 6

Table 6. Results of the Meta-Regression for Measures of Affective Symptoms 7

Table 6. Results of the Meta-Regression for Measures of Affective Symptoms 8

Table 5. Results of the Meta-Regressions for the Measures of Neuropsychiatric Symptoms 9

Table 5. Results of the Meta-Regressions for the Measures of Neuropsychiatric Symptoms 10

Table 6. Results of Meta-Regressions for Apraxias 11

Table 7. Results of Meta-Regressions for the Measures of Visuo-Spatial Processing 12

Table 7. Results of Meta-Regressions for Constructional Praxis 16

Table 10. Results of the Meta-Regression for Intelligence Measures 17

Table 11. Results of the Meta-Regressions for Measures of Attention 18

Table 12. Results of the Meta-Regressions for the Measures of Processing Speed 21

Table 13. Results of the Meta-Regressions for Measures of Language Production 22

Table 13. Results of the Meta-Regressions for Measures of Language Production 24

Table 14. Results of the Meta-Regressions for the Measures of Language Production 25

Table 15. Results of the Meta-Regressions for the Measures of Reasoning 26

Table 16. Results of the Meta-Regressions for the Measures of Executive Functioning 28

Table 16. Results of the Meta-Regressions for the Measures of Executive Functioning 32

Table 21. Results of the Meta-Regressions for Measures of Verbal Episodic and Semantic Memory 34

Table 22. Table of included Studies and their Nottingham-Ottawa Scale Ratings. S: Selectivity, C: Comparability, E: Exposure 40

Global Functioning

The measures of global functioning included the Global Deterioration Scale, Clinical Dementia Rating Scale, Dementia Rating Scale. On the Clinical Dementia Rating Scale, the sVaD group tends to perform worse than the VaD group.

Table 1. Results of Meta-Regressions for Global Functioning Measures

| Test | VaD Subtype | N_studies_ | N_participants_ | $\beta_{g}$ | 95% ETI | | Bayes Factor | Study references |
| --- | --- | --- | --- | --- | --- | --- | --- | --- |
|  |  |  |  |  | LB | UB |  |  |
| CDR |  |  |  |  |  |  |  | CDR: 13, 20, 31, 32, 33, 37, 66, 70, 71, 76, 90, 99, 104, 111, 118  DRS: 97, 98  GDS: 10, 11, 56, 122 |
|  | sVaD^E,A,G^ | 6 | 579 | -0.22 | -0.68 | 0.25 | **0.326** |  |
|  | VaD^E,A,G^ | 11 | 1903 | 0.19 | -0.07 | 0.44 | 0.385 |  |
| DRS |  |  |  |  |  |  |  |  |
|  | VaD^E,A,G^ | 2 | 133 | -0.68 | -1.45 | -0.05 | 2.87 |  |
| GDS |  |  |  |  |  |  |  |  |
|  | VaD^E,A,G^ | 4 | 299 | -0.31 | -0.86 | 0.27 | 0.438 |  |
|  | $\tau_{Study}$= 0.24 [0.02, 0.50]  $\tau_{Study/ES}$= 0.20 [0.01, 0.45] | | | | | | |  |
| **Notes:**  ETI stands for 95% equal-tailed credibility interval.  For τ parameters 95% confidence intervals are reported.  ^E,A,G^ – denote considered moderators   - E = difference in years of education between AD and VaD; - A = difference in average age between AD and VaD; - G = difference in average proportion of women between AD and VaD.   CDR: Clinical Dementia Rating Scale, GDS: Global Deterioration Scale, DRS: Dementia Rating Scale | | | | | | | | |

Orientation to Time and Space

Table 2. Results of the Meta-Regression for Orientation to Time and Space

| Test | VaD Subtype | N_studies_ | N_participants_ | $\beta_{g}$ | 95% ETI | | Bayes Factor | Study references |
| --- | --- | --- | --- | --- | --- | --- | --- | --- |
|  |  |  |  |  | LB | UB |  |  |
| Orientation |  |  |  |  |  | |  | 1, 35, 49, 56, 62, 70, 80, 85, 114 |
|  | MID | 2 | 111 | 0.13 | -0.68 | 1.12 | 0.355 |  |
|  | sVaD | 3 | 154 | 0.01 | -0.70 | 0.76 | **0.257** |  |
|  | VaD | 4 | 598 | -0.21 | -0.86 | 0.37 | **0.293** |  |
|  | $\tau_{Study}$= 0.48 [0.03, 1.09]  $\tau_{Study/ES}$= 0.32 [0.01, 0.88] | | | | | | |  |
| **Notes:**  ETI stands for 95% equal-tailed credibility interval.  For τ parameters 95% confidence intervals are reported. | | | | | | | | |

Activities of Daily Living

Table 3. Results of the Meta-Regression for the Basic and Instrumental Activities of Daily Living

| Test | VaD Subtype | N_studies_ | N_participants_ | $\beta_{g}$ | 95% ETI | | Bayes Factor | Study references |
| --- | --- | --- | --- | --- | --- | --- | --- | --- |
|  |  |  |  |  | LB | UB |  |  |
| Basic Activities of Daily Living |  |  |  |  |  | |  | 10, 11, 27, 31, 33, 42, 44, 45, 46, 51, 58, 69, 71, 88, 101, 103, 106, 108, 109, 112, 115 (2 samples), 121 |
|  | MID^S,E,A,G^ | 2 | 191 | -0.57 | -3.06 | 1.36 | 0.768 |  |
|  | sVaD^S,E,A,G^ | 3 | 290 | -1.73 | -3.63 | 0.01 | 4.14 |  |
|  | VaD^S,E,A,G^ | 15 | 3777 | -0.52 | -1.46 | 0.38 | 0.611 |  |
| Instrumental Activities of Daily Living |  |  |  |  |  | |  |  |
|  | sVaD^S,E,A,G^ | 2 | 173 | 0.92 | -0.70 | 2.91 | 1.05 |  |
|  | VaD^S,E,A,G^ | 9 | 6610 | -0.71 | -1.71 | 0.18 | 1.22 |  |
|  | $\tau_{Study}$= 1.92 [1.34, 2.73]  $\tau_{Study/ES}$= 0.23 [0.04, 0.44] | | | | | | |  |
| **Notes:**  ETI stands for 95% equal-tailed credibility interval.  For τ parameters 95% confidence intervals are reported.  ^A,G,S,E^ – denote considered moderators   - A = age difference between AD and VaD; - G = difference in proportion of women between AD and VaD; - S = difference in dementia severity between AD and VaD; - E = difference in years of education between AD and VaD. | | | | | | | | |

### Activities of Daily Living: Quality Sensitivity Analysis

Table 3. Results of the Meta-Regression for the Basic and Instrumental Activities of Daily Living

| Test | VaD Subtype | N_studies_ | N_participants_ | $\beta_{g}$ | 95% ETI | | Bayes Factor | Study references |
| --- | --- | --- | --- | --- | --- | --- | --- | --- |
|  |  |  |  |  | LB | UB |  |  |
| Basic Activities of Daily Living |  |  |  |  |  | |  |  |
|  | VaD | 3 | 385 | 0.12 | -0.28 | 0.52 | **0.180** | 44, 88, 106, 109 |
| Instrumental Activities of Daily Living |  |  |  |  |  | |  |  |
|  | VaD | 3 | 402 | -0.13 | -0.53 | 0.21 | **0.214** |  |
|  | $\tau_{Study}$= .17 [0.01, 0. 62]  $\tau_{Study/ES}$= 0.17 [0.01, 0.51] | | | | | | |  |
| **Notes:**  ETI stands for 95% equal-tailed credibility interval.  For τ parameters 95% confidence intervals are reported. | | | | | | | | |

Disease Awareness

Zanetti et al. 1999 and Starkstein et al. 1996 reported insight measures. The studies included 129 subjects. The analysis showed an overall mean effect of $\beta_{g}$ = 0.25 (95% ETI [-0.48, 1.12], BF = 0.396). The heterogeneity parameters are $\tau_{Study}$= 0.41 [0.01, 1.69] and $\tau_{Study/ES}$= 0.28 [0.01, 1.04]. For τ parameters 95% confidence intervals are reported.

Affective Symptoms

Table 6. Results of the Meta-Regression for Measures of Affective Symptoms

| Test | VaD Subtype | N_studies_ | N_participants_ | $\beta_{g}$ | 95% ETI | | Bayes Factor | Study references |
| --- | --- | --- | --- | --- | --- | --- | --- | --- |
|  |  |  |  |  | LB | UB |  |  |
| GDS |  |  |  |  |  |  |  | 10, 25, 31, 33, 42, 48, 49, 58, 63, 68, 69, 70, 71, 88, 102, 104, 112, 122 |
|  | sVaD^A,S,G,E^ | 7 | 793 | -0.05 | -0.44 | 0.33 | **0.154** |  |
|  | VaD^A,S,G,E^ | 9 | 1052 | -0.06 | -0.39 | 0.29 | **0.131** |  |
|  | VCI^A,S,G,E^ | 2 | 141 | 0.12 | -0.54 | 0.93 | **0.318** |  |
| HDRS |  |  |  |  |  |  |  | 14, 32, 106 |
|  | VaD^A,S,G,E^ | 3 | 606 | 0.05 | -0.38 | 0.47 | **0.170** |  |
| NPI-Anxiety |  |  |  |  |  |  |  | 15, 38, 64, 96, 111 |
|  | VaD^A,S,G,E^ | 5 | 2347 | -0.06 | -0.47 | 0.33 | **0.171** |  |
| NPI-Depression |  |  |  |  |  |  |  | 15, 38, 64, 111 |
|  | VaD^A,S,G,E^ | 4 | 2189 | -0.13 | -0.55 | 0.28 | **0.202** |  |
| STAIX-State |  |  |  |  |  |  |  | 92, 93 |
|  | VaD^A,S,G,E^ | 2 | 65 | 0.25 | -0.46 | 0.92 | **0.310** |  |
| STAIX-Trait |  |  |  |  |  |  |  | 92, 93 |
|  | VaD^A,S,G,E^ | 2 | 65 | 0.07 | -0.63 | 0.77 | **0.293** |  |
| Depression |  |  |  |  |  |  |  | 51, 92, 93, 94, 98, 99 |
|  | VaD^A,S,G,E^ | 6 | 1740 | -0.01 | -0.35 | 0.33 | **0.142** |  |
| Anxiety |  |  |  |  |  |  |  | 51, 106 |
|  | VaD^A,S,G,E^ | 2 | 1405 | -0.13 | -0.66 | 0.37 | **0.243** |  |
|  | $\tau_{Study}$= 0.25 [0.11, 0.42]  $\tau_{Study/ES}$= 0.25 [0.13, 0.40] | | | | | | |  |
| **Notes:**  ETI stands for 95% equal-tailed credibility interval.  For τ parameters 95% confidence intervals are reported.  ^A,G,S,E^ – denote considered moderators   - A = age difference between AD and VaD; - G = difference in proportion of women between AD and VaD; - S = difference in dementia severity between AD and VaD; - E = difference in years of education between AD and VaD.   GDS: Geriatric Depression Scale, NPI: Neuropsychiatric Inventory, HDRS: Hamilton Depression Rating Scale, STAIX: State trait anxiety inventory. Depression measures included: a questionnaire on Depressed Mood, the Self-Rating Depression Scale, the Rome Depression Inventory, the Korean Consortium to Establish a Registry for Alzheimer's Disease (CERAD) measure of the Number of Depressive Symptoms, the Cornell Scale for Depression in Dementia and the Beck’s Depression Inventory. Anxiety measures included: a questionnaire on Symptoms of Anxiety and the Hamilton Anxiety Scale. | | | | | | | | |

### Affective Symptoms: Quality Sensitivity Analysis

Table 6. Results of the Meta-Regression for Measures of Affective Symptoms

| Test | VaD Subtype | N_studies_ | N_participants_ | $\beta_{g}$ | 95% ETI | | Bayes Factor | Study references |
| --- | --- | --- | --- | --- | --- | --- | --- | --- |
|  |  |  |  |  | LB | UB |  |  |
| Depression |  |  |  |  |  |  |  | 15, 38, 88,106 |
|  | VaD | 4 | 1040 | 0.09 | -0.30 | 0.51 | **0.164** |  |
| Anxiety |  |  |  |  |  |  |  | 15, 38, 106 |
|  | VaD | 3 | 946 | -0.02 | -0.41 | 0.47 | **0.155** |  |
|  | $\tau_{Study}$= 0.25 [0.10, 0.75]  $\tau_{Study/ES}$= 0.19 [0.01, 0.57] | | | | | | |  |
| **Notes:**  ETI stands for 95% equal-tailed credibility interval.  For τ parameters 95% confidence intervals are reported. | | | | | | | | |

Neuropsychiatric Symptoms

Other global score measures of neuropsychiatric symptoms included: the Frontal Systems Behavior Scale and the Behavioral Pathology in Alzheimer's Disease Rating Scale, the Cohen-Mansfield Agitation Inventory, the Frontal Behavioural Inventory, the Dementia Psychosis Scale, and the Behavior Problems Check List.

The domain data are based on NPI subscales and the Scale for the Assessment of Negative Symptoms, the Positive and Negative Syndrome Scale for Schizophrenia, the Psychogeriatric Dependency Rating Scale, Frontal Systems Behavior Scale and the Behavioral Pathology in Alzheimer's Disease Rating Scale subscales, the Cohen-Mansfield Agitation Inventory, the Behavioral Pathology in Alzheimer's Disease Rating Scale, the Pathological Laughing and Crying Scale, the Neurobehavioral Rating Scale, the Revised Memory and Behavior Problems Check List and the Blessed Dementia Rating Scale.

Table 5. Results of the Meta-Regressions for the Measures of Neuropsychiatric Symptoms

| Test | VaD Subtype | N_studies_ | N_participants_ | $\beta_{g}$ | 95% ETI | | Bayes Factor | Study references |
| --- | --- | --- | --- | --- | --- | --- | --- | --- |
|  |  |  |  |  | LB | UB |  |  |
| Domains |  |  |  |  |  | |  |  |
| - Aberrant Motor Behavior | VaD^S^ | 4 | 2189 | -0.11 | -0.42 | 0.25 | **0.141** | 15, 38, 51, 64, 101, 106, 111 |
| - Agitation/Aggression | VaD^S^ | 5 | 2509 | 0.08 | -0.26 | 0.41 | **0.143** |  |
| - Apathy | VaD^S^ | 5 | 3534 | 0.18 | -0.13 | 0.53 | **0.322** |  |
| - Appetite Change | VaD^S^ | 4 | 2189 | 0.06 | -0.34 | 0.35 | **0.147** |  |
| - Delusions | VaD^S^ | 5 | 3534 | 0.07 | -0.27 | 0.37 | **0.131** |  |
| - Disinhibition | VaD^S^ | 4 | 2189 | 0.01 | -0.31 | 0.35 | **0.127** |  |
| - Diurnal Rhythm Disturbances | VaD^S^ | 4 | 2189 | -0.09 | -0.44 | 0.24 | **0.161** |  |
| - Euphoria | VaD^S^ | 3 | 2133 | 0.07 | -0.28 | 0.42 | **0.132** |  |
| - Hallucinations | VaD^S^ | 5 | 3534 | 0.16 | -0.16 | 0.46 | **0.227** |  |
| - Irritability | VaD^S^ | 4 | 2189 | 0.12 | -0.27 | 0.43 | **0.148** |  |
|  | $\tau_{Study}$= 0.30 [0.13, 0.65]  $\tau_{Study/ES}$= 0.16 [0.10, 0.25] | | | | | | |  |
| **Notes:**  ETI stands for 95% equal-tailed credibility interval.  For τ parameters 95% confidence intervals are reported.  ^S,E^ – denote considered moderators   - S = difference in dementia severity between AD and VaD; | | | | | | | | |

### Neuropsychiatric Symptoms - Domains: Quality Sensitivity Analysis

Table 5. Results of the Meta-Regressions for the Measures of Neuropsychiatric Symptoms

| Test | VaD Subtype | N_studies_ | N_participants_ | $\beta_{g}$ | 95% ETI | | Bayes Factor | Study references |
| --- | --- | --- | --- | --- | --- | --- | --- | --- |
|  |  |  |  |  | LB | UB |  |  |
| Domains |  |  |  |  |  | |  |  |
| - Aberrant Motor Behavior | VaD | 2 | 886 | -0.32 | -0.74 | 0.14 | 0.713 | 15, 38 |
| - Agitation/Aggression | VaD | 2 | 886 | -0.14 | -0.51 | 0.39 | **0.231** |  |
| - Apathy | VaD | 2 | 886 | -0.14 | -0.53 | 0.34 | **0.217** |  |
| - Appetite Change | VaD | 2 | 886 | -0.16 | -0.59 | 0.32 | **0.258** |  |
| - Delusions | VaD | 2 | 886 | -0.16 | -0.53 | 0.36 | **0.248** |  |
| - Disinhibition | VaD | 2 | 886 | 0.01 | -0.44 | 0.46 | **0.169** |  |
| - Diurnal Rhythm Disturbances | VaD | 2 | 886 | -0.18 | -0.62 | 0.29 | **0.270** |  |
| - Euphoria | VaD | 2 | 886 | 0.00 | -0.42 | 0.47 | **0.170** |  |
| - Hallucinations | VaD | 2 | 886 | -0.09 | -0.44 | 0.46 | **0.194** |  |
| - Irritability | VaD | 2 | 886 | -0.13 | -0.49 | 0.40 | **0.216** |  |
|  | $\tau_{Study}$= 0.25 [0.01, 1.14]  $\tau_{Study/ES}$= 0.16 [0.01, 0.37] | | | | | | |  |
| **Notes:**  ETI stands for 95% equal-tailed credibility interval.  For τ parameters 95% confidence intervals are reported.  The results are from a model using the Student-t prior for the regression coefficients and an exponential prior on for τ parameters. None of the other models converged. | | | | | | | | |

### Neuropsychiatric Symptoms – Global Score: Quality Sensitivity Analysis

The final sample contained three studies [15, 38, 107] and 946 subjects. Measures used were the NPI total score and the total score on the Dementia Psychosis Scale. The analysis showed an overall mean effect of $\beta_{g}$ = -0.22 (95% ETI [-0.64, 0.20], BF = 0.443) and the heterogeneity parameter $\tau_{Study}$= 0.25 [0.01, 0.93]. For τ parameters 95% confidence intervals are reported.

Apraxia

The Apraxia tests included a test of tool use, Motor performance on the Dementia Rating Scale and the Boston Apraxia Examination. The measures of Facial Apraxia included tasks on buccofacial and oral praxis. Finally, Ideomotor Apraxia included tasks described as ideomotor praxis.

Table 6. Results of Meta-Regressions for Apraxias

| Test | VaD Subtype | N_studies_ | N_participants_ | $\beta_{g}$ | 95% ETI | | Bayes Factor | Study references |
| --- | --- | --- | --- | --- | --- | --- | --- | --- |
|  |  |  |  |  | LB | UB |  |  |
| Apraxia |  |  |  |  |  |  |  |  |
|  | VaD | 3 | 189 | 1.11 | -0.35 | 2.18 | 2.02 | 30, 65, 91 |
|  | $\tau_{Study}$= 0.53 [0.02, 1.98]  $\tau_{Study/ES}$= 1.01 [0.41, 2.29] | | | | | | |  |
| Facial Apraxia |  |  |  |  |  |  |  |  |
|  | VaD | 2 | 139 | 0.03 | -0.58 | 0.64 | **0.190** | 87, 112 |
|  | $\tau_{Study}$= 0.32 [0.01, 1.35] | | | | | | |  |
| Ideomotor Apraxia |  |  |  |  |  |  |  |  |
|  | VaD | 4 | 284 | 0.00 | -0.33 | 0.33 | **0.119** | 14, 87, 112, 118 |
|  | $\tau_{Study}$= 0.19 [0.01, 0.65] | | | | | | |  |
| **Notes:**  ETI stands for 95% equal-tailed credibility interval.  For τ parameters 95% confidence intervals are reported. | | | | | | | | |

Motor Functioning

The final sample contained the Doddy et al. 1998, Starkstein et al. 1996 and Villardita et al. 1993 studies with a total of 171 subjects. The measures included were the Unified Parkinson’s Disease Rating Scale, Finger Tapping, Gibson spiral and a motor programming task. The analysis showed an overall effect of $\beta_{g}$ = 0.66 (95% ETI [-0.03, 1.42], BF = 2.66). The heterogeneity parameters were $\tau_{Study}$= 0.45 [0.02, 1.60] and $\tau_{Study/ES}$= 0.41 [0.19, 0.71]. For τ parameters 95% confidence intervals are reported.

Visuo-Spatial Processing

Other measures of figure copy included the Wechsler Memory Scale – Revised Visual Reproduction, Mini Mental State Examination pentagons copy, NEUROPSI-Test Battery Figure Copy, and other tasks of figure copy. Other measures of constructional praxis included the Developmental Test of Visuo-Motor Integration, the Battery for Visuospatial Abilities, Neurobehavioral Cognitive Status Examination – Constructions, Arizona Battery for Communication Disorders of Dementia –Visuospatial Construction Total, the Stick Design Test, the Lezak Tinker Toy Test, the Boston Three-Dimensional Block Construction Test, Koch’s Baum Test, and other visuo-constructional tasks. Other Measures of Spatial Processing included a map test and a spatial rotation task. Other Measures of Visuo-Spatial Processing included overlapping figures, a double barrage task, the Battery for Visuospatial Abilities – Complex Figure Identification and Point position identification, Incomplete Drawings, Incomplete Letters, visual errors from the Boston Naming Test.

Table 7. Results of Meta-Regressions for the Measures of Visuo-Spatial Processing

| Test | VaD Subtype | | N_studies_ | N_participants_ | | $\beta_{g}$ | | 95% ETI | | | | Bayes Factor | | Study references |
| --- | --- | --- | --- | --- | --- | --- | --- | --- | --- | --- | --- | --- | --- | --- |
|  |  | |  |  | |  | | LB | | UB | |  | |  |
| WAIS |  | |  |  | |  | |  | | | |  | |  |
| a) Block Design | MID^S,E^ | | 2 | 127 | | -0.15 | | -0.72 | | 0.42 | | 0.526 | | 13, 37, 55, 56, 63, 65, 77, 81, 89, 93, 98, 106, 114, 117 |
|  | VaD^S,E^ | | 13 | 1724 | | 0.08 | | -0.16 | | 0.30 | | **0.256** | |  |
| b) Object Assembly | VaD^S,E^ | | 3 | 370 | | 0.26 | | -0.19 | | 0.71 | | 0.784 | | 65, 81, 117 |
| c) Picture Completion | VaD^S,E^ | | 2 | 288 | | 0.04 | | -0.48 | | 0.55 | | 0.411 | | 81, 117 |
|  | $\tau_{Study}$= 0.23 [0.02, 0.51]  $\tau_{Study/ES}$= 0.16 [0.01, 0.41] | | | | | | | | | | | | |  |
| Clock Drawing Test |  | |  |  | |  | |  | |  | |  | |  |
| 1. Copy | VaD^S,E^ | | 2 | 108 | | 0.00 | | -0-44 | | 0.49 | | **0.181** | | 6, 8 |
| 1. Drawing | sVaD^S,E^ | | 4 | 335 | | 0.24 | | -0.11 | | 0.59 | | 0.356 | | 6, 8, 26, 31, 34, 40, 42, 44, 53, 54, 58, 63, 79, 82, 85, 100, 115 |
|  | VaD^S,E^ | | 14 | 2219 | | -0.03 | | -0.20 | | 0.17 | | **0.078** | |  |
| 1. Clock Reading | VaD^S,E^ | | 2 | 142 | | 0.26 | | -0.20 | | 0.72 | | 0.375 | | 37, 44 |
| - Severity |  | |  |  | | 0.24 | | 0.04 | | 0.40 | | 1.49 | |  |
|  | $\tau_{Study}$= 0.26 [0.12, 0.46]  $\tau_{Study/ES}$= 0.06 [0.00, 0.20] | | | | | | | | | | | | |  |
| Rey Osterrieth Complex Figure Test |  | |  |  | |  | |  | |  | |  | |  |
| a) Copy | sVaD | | 3 | 519 | | 0.02 | | -0.27 | | 0.37 | | **0.117** | | 20, 28, 55, 87, 89, 93, 110, 114, 118 |
|  | VaD | | 6 | 848 | | 0.02 | | -0.22 | | 0.27 | | **0.086** | |  |
|  | $\tau_{Study}$= 0.20 [0.02, 0.47] | | | | | | | | | | | | |  |
| VOSP |  | |  |  | |  | |  | |  | |  | |  |
|  | VaD | | 2 | 329 | | -0.13 | | -0.86 | | 0.62 | | **0.247** | | 89, 104 |
|  | $\tau_{Study}$= 0.37 [0.01, 1.44]  $\tau_{Study/ES}$= 0.25 [0.01, 0.80] | | | | | | | | | | | | |  |
| CERAD |  | |  |  | |  | |  | |  | |  | |  |
| a) Constructional Praxis | VaD | | 2 | 323 | | -0.39 | | -1.06 | | 0.60 | | 0.526 | | 13, 57 |
|  | $\tau_{Study}$= 0.50 [0.02, 1.81] | | | | | | | | | | | | |  |
| ACE |  | |  |  | |  | |  | |  | |  | |  |
| a) Visuo-Spatial Processing | VaD | | 2 | 148 | | 0.17 | | -0.50 | | 0.81 | | **0.264** | | 1, 70 |
|  | $\tau_{Study}$= 0.40 [0.01, 1.56] | | | | | | | | | | | | |  |
| Judgment of Line Orientation |  |  | | |  | |  | |  | |  | |  |  |
|  | VaD | | 3 | 217 | | -0.96 | | -1.83 | | 0.36 | | 1.94 | | 14, 28, 98 |
|  | $\tau_{Study}$= 0.92 [0.31, 2.48] | | | | | | | | | | | | |  |
| Hooper’s Test |  | |  |  | |  | |  | |  | |  | |  |
|  | VaD | | 3 | 154 | | -0.03 | | -0.48 | | 0.39 | | **0.156** | | 93, 98, 114 |
|  | $\tau_{Study}$= 0.23 [0.01, 0.83] | | | | | | | | | | | | |  |
| Line Bisection |  | |  |  | |  | |  | |  | |  | |  |
|  | VaD | | 2 | 152 | | -0.45 | | -1.32 | | 0.54 | | 0.736 | | 3, 26 |
|  | $\tau_{Study}$= 0.52 [0.02, 1.95]  $\tau_{Study/ES}$= 0.35 [0.01, 1.15] | | | | | | | | | | | | |  |
| Other Measures of Figure Copy |  | |  |  | |  | |  | |  | |  | |  |
|  | sVaD^S^ | | 8 | 894 | | 0.23 | | 0.01 | | 0.50 | | 0.858 | | 19, 27, 28, 44, 62, 63, 80, 81, 87, 95, 110, 112 |
|  | VaD^S^ | | 4 | 477 | | 0.03 | | -0.30 | | 0.37 | | **0.126** | |  |
|  | $\tau_{Study}$= 0.15 [0.01, 0.40]  $\tau_{Study/ES}$= 0.14 [0.01, 0.39] | | | | | | | | | | | | |  |
| Other Measures of Constructional Praxis |  | |  |  | |  | |  | |  | |  | |  |
|  | MID^S^ | | 3 | 85 | | 0.18 | | -0.73 | | 1.01 | | 0.399 | | 6, 14, 20, 22, 27, 28, 35, 41, 42, 83, 105 |
|  | sVaD^S^ | | 3 | 230 | | -0.10 | | -0.85 | | 0.82 | | **0.296** | |  |
|  | VaD^S^ | | 5 | 458 | | -0.05 | | -0.54 | | 0.60 | | **0.196** | |  |
|  | $\tau_{Study}$= 0.39 [0.04, 0.91]  $\tau_{Study/ES}$= 0.64 [0.44, 0.91] | | | | | | | | | | | | |  |
| Other Measures |  | |  |  | |  | |  | |  | |  | |  |
| a) Visuo-Spatial Processing | VaD^S^ | | 4 | 339 | | -0.20 | | -0.68 | | 0.25 | | **0.260** | | 3, 26, 28, 87 |
| b) Visual Perception | VaD^S^ | | 4 | 282 | | -0.31 | | -0.83 | | 0.15 | | 0.577 | | 28, 42, 78, 114 |
|  | $\tau_{Study}$= 0.33 [0.02, 0.92]  $\tau_{Study/ES}$= 0.21 [0.01, 0.61] | | | | | | | | | | | | |  |
| **Notes:**  ETI stands for 95% equal-tailed credibility interval.  For τ parameters 95% confidence intervals are reported.  ^S,E^ – denote considered moderators   - S = difference in dementia severity between AD and VaD; - E = difference in years of education between AD and VaD.   WAIS: Wechsler Adult Intelligence Scale, VOSP: Visual Object and Space Perception Battery, CERAD: Consortium to Establish a Registry for Alzheimer's Disease cognitive test battery, ACE: Addenbrook’s Cognitive Examination. | | | | | | | | | | | | | | |

### Visuo-Spatial Processing: Quality Sensitivity Analysis

Table 7. Results of Meta-Regressions for Constructional Praxis

| Test | VaD Subtype | N_studies_ | N_participants_ | $\beta_{g}$ | 95% ETI | | Bayes Factor | Study references |
| --- | --- | --- | --- | --- | --- | --- | --- | --- |
|  |  |  |  |  | LB | UB |  |  |
| Constructional Praxis |  |  |  |  |  |  |  |  |
|  | VaD^S^ | 6 | 471 | 0.03 | -0.48 | 0.63 | **0.180** | 40, 44, 53, 77, 88, 106 |
|  | $\tau_{Study}$= 0.60 [0.23, 1.30]  $\tau_{Study/ES}$= 0.46 [0.31, 0.67] | | | | | | |  |
| **Notes:**  ETI stands for 95% equal-tailed credibility interval.  For τ parameters 95% confidence intervals are reported.  ^S^ = difference in dementia severity between AD and VaD; | | | | | | | | |

Intelligence Measures

Table 10. Results of the Meta-Regression for Intelligence Measures

| Test | VaD Subtype | N_studies_ | N_participants_ | $\beta_{g}$ | 95% ETI | | Bayes Factor | Study references |
| --- | --- | --- | --- | --- | --- | --- | --- | --- |
|  |  |  |  |  | LB | UB |  |  |
| WAIS |  |  |  |  |  | |  |  |
| - Full Scale IQ | VaD | 3 | 391 | 0.10 | -0.46 | 0.67 | **0.236** | 36, 68, 98, 117 |
| - Fluid IQ | VaD | 2 | 347 | 0.15 | -0.48 | 0.79 | **0.265** |  |
| - Crystallized IQ | VaD | 3 | 405 | 0.08 | -0.59 | 0.78 | **0.255** |  |
|  | $\tau_{Study}$= 0.43 [0.03, 1.13]  $\tau_{Study/ES}$= 0.21 [0.01, 0.67] | | | | | | |  |
| **Notes:**  ETI stands for 95% equal-tailed credibility interval.  For τ parameters 95% confidence intervals are reported.  WAIS: Wechsler Adult Intelligence Scale. | | | | | | | | |

Attention

Other Measures of Selective Attention include the Digit Cancellation Test, the WAIS-R freedom from distractibility index, the test of everyday attention, the correct number of named colors and read words from the Stroop task, the RBANS coding task, the line cancellation task, the Kana-Hiroi task, the Adult Memory and Information Processing Battery – Information Processing Scores and the accuracy of a choice reaction time test.

Other Measures of Sustained Attention include the Letter Cancellation Task, Test of everyday attention, digit cancellation task, seashore rhythm task, NEUROPSI – Attention/Concentration, Digit Vifilance Task, reaction time variability, Figure Cancellation Test, PASAT, Kendrick Digit Copying Test.

Measures of Visual Attention include the RCFT left side figure score, map search, test drive, lines cancellation, multiple features targets cancellation, NAI-Zahlenverbindungstest, NEUROPSI – visual detection, Color Trails – A, a visual search task.

Divided Attention Tasks included the Della Sala dual task and another dual task.

Table 11. Results of the Meta-Regressions for Measures of Attention

| Test | VaD Subtype | N_studies_ | N_participants_ | $\beta_{g}$ | 95% ETI | | Bayes Factor | Study references |
| --- | --- | --- | --- | --- | --- | --- | --- | --- |
|  |  |  |  |  | LB | UB |  |  |
| Trail Making Test – A |  |  |  |  |  | |  |  |
|  | sVaD^S^ | 4 | 665 | 0.00 | -0.38 | 0.42 | **0.146** | 6, 42, 55, 84, 89, 93, 104, 110, 115, 120 |
|  | VaD^S^ | 5 | 804 | 0.00 | -0.47 | 0.42 | **0.158** |  |
|  | VCI^S^ | 2 | 695 | 0.22 | -0.28 | 0.72 | **0.325** |  |
|  | $\tau_{Study}$= 0.24 [0.02, 0.59] | | | | | | |  |
| Digit Span Forward |  |  |  |  |  |  |  |  |
|  | sVaD^S, E^ | 6 | 676 | 0.10 | -0.15 | 0.35 | **0.147** | 2, 22, 42, 57, 63, 81, 87, 88, 89, 93, 97, 104, 106, 114, 118 |
|  | VaD^S,E^ | 9 | 530 | 0.16 | -0.07 | 0.37 | **0.220** |  |
|  | $\tau_{Study}$= 0.13 [0.01, 0.35] | | | | | | |  |
| Digit Symbol Substitution Test |  |  |  |  |  |  |  |  |
|  | sVaD | 2 | 83 | 0.30 | -0.19 | 0.82 | 0.414 | 68, 81, 84, 89, 93, 117 |
|  | VaD | 4 | 475 | 0.22 | -0.11 | 0.52 | 0.401 |  |
|  | $\tau_{Study}$= 0.17 [0.01, 0.54] | | | | | | |  |
| Symbol Digit Modalities Test |  |  |  |  |  |  |  |  |
|  | sVaD | 2 | 232 | 0.46 | -0.17 | 1.02 | 1.26 | 63, 120 |
|  | $\tau_{Study}$= 0.31 [0.01, 1.31] | | | | | | |  |
| Choice Reaction Time |  |  |  |  |  |  |  |  |
|  | VaD | 4 | 276 | -0.19 | -0.67 | 0.30 | **0.262** | 14, 82, 84, 87 |
|  | $\tau_{Study}$= 0.29 [0.01, 0.93]  $\tau_{Study/ES}$= 0.24 [0.01, 0.74] | | | | | | |  |
| Other Measures of Selective Attention |  |  |  |  |  |  |  |  |
|  | VaD^S^ | 5 | 275 | 0.10 | -0.38 | 0.60 | **0.186** | 22, 30, 87, 92, 118 |
|  | $\tau_{Study}$= 0.52 [0.13, 1.45]  $\tau_{Study/ES}$= 0.40 [0.11, 1.09] | | | | | | |  |
| Continuous Performance Tests |  |  |  |  |  |  |  |  |
|  | VaD | 3 | 232 | 0.44 | -0.32 | 1.03 | 0.665 | 84, 88, 114 |
|  | $\tau_{Study}$= 0.40 [0.03, 1.36]  $\tau_{Study/ES}$= 0.39 [0.03, 1.02] | | | | | | |  |
| Other Measures of Sustained Attention |  |  |  |  |  |  |  |  |
|  | VaD | 4 | 296 | -0.02 | -0.75 | 0.53 | **0.226** | 26, 63, 83, 93 |
|  | $\tau_{Study}$= 0.34 [0.01, 1.12]  $\tau_{Study/ES}$= 0.50 [0.06, 1.26] | | | | | | |  |
| Visual Attention Measures |  |  |  |  |  |  |  |  |
|  | VaD^S^ | 5 | 257 | 0.00 | -0.69 | 0.82 | **0.264** | 26, 40, 57, 80, 112 |
|  | $\tau_{Study}$= 0.46 [0.02, 1.43]  $\tau_{Study/ES}$= 0.50 [0.06, 1.26] | | | | | | |  |
| Other Measures of Attention |  |  |  |  |  |  |  |  |
|  | VaD | 4 | 237 | 0.40 | -0.64 | 1.43 | 0.488 | 1, 35, 40, 89 |
|  | $\tau_{Study}$= 1.17 [0.51, 2.65] | | | | | | |  |
| **Notes:**  ETI stands for 95% equal-tailed credibility interval.  For τ parameters 95% confidence intervals are reported.  ^S^ – denotes the moderator difference in average dementia severity between AD and VaD;  ^E^ – denotes the moderator difference in average years of education between AD and VaD. | | | | | | | | |

### Attention: Quality Sensitivity Analysis

The final sample contained two studies [88, 106] and 154 subjects using a digit span forward task. The analysis showed an overall mean effect of $\beta_{g}$ = 0.07 (95% ETI [-0.59, 0.82], BF = **0.239**) and the heterogeneity parameter $\tau_{Study}$= 0.39 [0.01, 1.54]. For τ parameters 95% confidence intervals are reported.

Processing Speed

The analyses will be split into the Stroop task, reaction time measures and Other measures of processing speed, which included letter, feature and line cancelation tests, letter and pattern comparison as well as motor speed.

Table 12. Results of the Meta-Regressions for the Measures of Processing Speed

| Test | VaD Subtype | N_studies_ | N_participants_ | $\beta_{g}$ | 95% ETI | | Bayes Factor | Study references |
| --- | --- | --- | --- | --- | --- | --- | --- | --- |
|  |  |  |  |  | LB | UB |  |  |
| Stroop |  |  |  |  |  | |  |  |
| - Word Reading & Color Naming | VaD | 2 | 463 | -0.04 | -0.58 | 0.66 | **0.171** | 55, 93 |
|  | $\tau_{Study}$= 0.34 [0.01, 1.31]  $\tau_{Study/ES}$= 0.20 [0.00, 0.73] | | | | | | |  |
| Reaction Time |  |  |  |  |  | |  |  |
|  | sVaD | 2 | 100 | 0.16 | -0.49 | 0.79 | **0.242** | 14, 84 |
|  | $\tau_{Study}$= 0.34 [0.01, 1.39] | | | | | | |  |
| Other Measures of Processing Speed |  |  |  |  |  | |  |  |
|  | VaD | 2 | 126 | -0.29 | -0.94 | 0.45 | 0.460 | 26, 87 |
|  | $\tau_{Study}$= 0.37 [0.01, 1.45]  $\tau_{Study/ES}$= 0.21 [0.01, 0.67] | | | | | | |  |
| **Notes:**  ETI stands for 95% equal-tailed credibility interval.  For τ parameters 95% confidence intervals are reported. | | | | | | | | |

Language Production

Global Measures of verbal Fluency were measures averaging over phonemic and semantic word fluency. Other Naming Measures included: the Test of Classification and Recall of Pictures, Mini Mental State Examination – Naming tasks, the Visual Association Test, the Arizona Battery for Communication Disorders of Dementia – Naming, NEUROPSI-Test Battery – Naming, the Brief Cognitive Screening Battery-Edu – Naming, Neurobehavioral Cognitive Status Examination – Naming, and other naming measures. Writing measures included the Mini Mental State Examination – Writing task, the Western Aphasia Battery – Writing and NEUROPSI-Test Battery – Writing. Finally, Other Measures of Language Production included the Boston Diagnostic Aphasia Examination – Cookie Theft Picture task and the total score on the Linguistic Expression scale of the Arizona Battery for Communication Disorders of Dementia.

Table 13. Results of the Meta-Regressions for Measures of Language Production

| Test | VaD Subtype | N_studies_ | N_participants_ | $\beta_{g}$ | 95% ETI | | Bayes Factor | Study references |
| --- | --- | --- | --- | --- | --- | --- | --- | --- |
|  |  |  |  |  | LB | UB |  |  |
| Global Measures of verbal Fluency |  |  |  |  |  | |  |  |
|  | sVaD^S,E,A,G^ | 2 | 100 | 0.51 | -0.15 | 1.19 | 0.846 | 70, 80 |
| Phonemic Fluency |  |  |  |  |  |  |  |  |
|  | MID^S,E,A,G^ | 2 | 68 | 0.72 | 0.06 | 1.47 | 2.66 | 3, 6, 12, 14, 19, 28, 30, 37, 46, 55, 57, 67, 72, 73, 78, 77, 79, 80, 82, 83, 84, 87, 89, 92, 93, 95, 97, 104, 106, 110, 112, 113, 114, 118, 120 |
|  | sVaD^S,E,A,G^ | 17 | 1460 | 0.51 | 0.22 | 0.77 | **42.36** |  |
|  | VaD^S,E,A,G^ | 14 | 1369 | 0.12 | -0.16 | 0.39 | **0.157** |  |
|  | VCI^S,E,A,G^ | 3 | 265 | 0.20 | -0.32 | 0.70 | **0.277** |  |
|  | sVaD – VaD |  |  | 0.39 | 0.02 | 0.73 |  |  |
| Semantic Fluency |  |  |  |  |  |  |  |  |
|  | MID^S,E,A,G^ | 6 | 230 | 0.20 | -0.20 | 0.64 | **0.264** | 1, 6, 9, 14, 18, 19, 22, 26, 35, 39, 41, 42, 46, 49, 50, 56, 57, 71, 77, 80, 82, 84, 85, 87, 92, 93, 97, 104, 110, 112, 113, 114, 118, 120 |
|  | sVaD^S,E,A,G^ | 13 | 1288 | 0.10 | -0.18 | 0.40 | **0.157** |  |
|  | VaD^S,E,A,G^ | 15 | 1409 | 0.02 | -0.28 | 0.27 | **0.109** |  |
|  | VCI^S,E,A,G^ | 2 | 145 | 0.06 | -0.53 | 0.62 | **0.221** |  |
|  | $\tau_{Study}$= 0.40 [0.27, 0.54]  $\tau_{Study/ES}$= 0.12 [0.01, 0.29] | | | | | | |  |
| Boston Naming Test |  |  |  |  |  |  |  |  |
| 1. Total Score | MID | 3 | 101 | -0.27 | -0.87 | 0.31 | **0.353** | 6, 13, 21, 26, 41, 46, 55, 57, 72, 78, 83, 84, 89, 93, 100, 106, 112, 113, 114, 118 |
|  | sVaD | 8 | 721 | 0.13 | -0.48 | 0.22 | 0.163 |  |
|  | VaD | 10 | 1261 | -0.16 | -0.49 | 0.10 | **0.277** |  |
| 1. Errors | sVaD | 2 | 336 | -0.03 | -0.64 | 0.59 | **0.159** |  |
|  | $\tau_{Study}$= 0.36 [0.02, 1.33]  $\tau_{Study/ES}$= 0.27 [0.09, 0.55] | | | | | | |  |
| Other Naming Measures |  |  |  |  |  |  |  |  |
|  | MID | 2 | 111 | 0.40 | -0.44 | 1.20 | 0.718 | 9, 14, 35, 42, 56, 62, 80, 104 |
|  | sVaD | 3 | 277 | -0.08 | -0.66 | 0.42 | **0.200** |  |
|  | VaD | 3 | 268 | -0.08 | -0.66 | 0.54 | **0.221** |  |
|  | $\tau_{Study}$= 0.35 [0.03, 0.86]  $\tau_{Study/ES}$= 0.31 [0.05, 0.60] | | | | | | |  |
| ACE |  |  |  |  |  |  |  |  |
| 1. Language | VaD | 2 | 148 | 0.16 | -0.62 | 0.80 | **0.259** | 1, 70 |
|  | $\tau_{Study}$= 0.41 [0.05, 1.59] | | | | | | |  |
| Writing |  |  |  |  |  |  |  |  |
|  | VaD | 3 | 325 | 0.33 | -0.26 | 0.92 | 0.552 | 62, 65, 80 |
|  | $\tau_{Study}$= 0.38 [0.01, 1.27] | | | | | | |  |
| Other Measures of Language Production |  |  |  |  |  |  |  |  |
|  | VaD | 3 | 157 | 0.16 | -0.88 | 1.24 | 0.396 | 42, 83, 114 |
|  | $\tau_{Study}$= 1.01 [0.35, 2.60] | | | | | | |  |
| **Notes:**  ETI stands for 95% equal-tailed credibility interval.  For τ parameters 95% confidence intervals are reported.  ^A,G,S,E^ – denote considered moderators   1. A = age difference between AD and VaD; 2. G = difference in proportion of women between AD and VaD; 3. S = difference in dementia severity between AD and VaD; 4. E = difference in years of education between AD and VaD.   ACE: Addenbrook’s Cognitive Examination | | | | | | | | |

### Language Production: Quality Sensitivity Analysis

Table 13. Results of the Meta-Regressions for Measures of Language Production

| Test | VaD Subtype | N_studies_ | N_participants_ | $\beta_{g}$ | 95% ETI | | Bayes Factor | Study references |
| --- | --- | --- | --- | --- | --- | --- | --- | --- |
|  |  |  |  |  | LB | UB |  |  |
| Boston Naming Test |  |  |  |  |  |  |  |  |
|  | VaD | 2 | 123 | 0.12 | -0.52 | 0.73 | 0.476 | 72, 106 |
|  | $\tau_{Study}$= 0.32 [0.01, 1.30] | | | | | | |  |
| Phonemic Fluency |  |  |  |  |  |  |  |  |
|  | VaD | 3 | 180 | 0.33 | -0.13 | 0.77 | 2.10 | 72, 77, 106 |
|  | $\tau_{Study}$= 0.25 [0.01, 0.91] | | | | | | |  |
| **Notes:**  ETI stands for 95% equal-tailed credibility interval.  For τ parameters 95% confidence intervals are reported. | | | | | | | | |

Language Comprehension

Aphasia inventories included: the Western Aphasia Battery: Segmental Commands, the Assessment of Subtle Language Deficits Logical Grammar subtest, and the Arizona Battery for Communication Disorders of Dementia. Other Measures of Language Comprehension included: the Neurobehavior Cognitive Status Examination – Comprehension subtest, the Wiig-Semel Logico-Grammatical Comprehension Test, the Quick Test of receptive vocabulary, the following commands task from the Mini Mental State Examination, the NEUROPSI-Test battery Comprehension subtest.

Table 14. Results of the Meta-Regressions for the Measures of Language Production

| Test | VaD Subtype | N_studies_ | N_participants_ | $\beta_{g}$ | 95% ETI | | Bayes Factor | Study references |
| --- | --- | --- | --- | --- | --- | --- | --- | --- |
|  |  |  |  |  | LB | UB |  |  |
| Token Test |  |  |  |  |  | |  |  |
|  | sVaD | 4 | 582 | -0.06 | -0.44 | 0.32 | **0.141** | 14, 19, 26, 89, 93, 106, 110, 112, 114 |
|  | VaD | 5 | 375 | 0.06 | -0.33 | 0.40 | **0.138** |  |
|  | $\tau_{Study}$= 0.31 [0.08, 0.65] | | | | | | |  |
| Aphasia Inventories |  |  |  |  |  |  |  |  |
|  | VaD | 3 | 196 | -0.02 | -0.94 | 0.67 | **0.267** | 30, 42, 89 |
|  | $\tau_{Study}$= 0.70 [0.17, 1.97] | | | | | | |  |
| Other Measures of Language Comprehension |  |  |  |  |  |  |  |  |
|  | VaD | 4 | 344 | 0.27 | -0.20 | 0.76 | 0.475 | 35, 56, 62, 80 |
|  | $\tau_{Study}$= 0.27 [0.01, 0.89]  $\tau_{Study/ES}$= 0.22 [0.01, 0.73] | | | | | | |  |
| **Notes:**  ETI stands for 95% equal-tailed credibility interval.  For τ parameters 95% confidence intervals are reported. | | | | | | | | |

Reading

The final sample contained four studies [42, 62, 68, 80], 334 subjects. The tasks included the NEUROPSI-Test Battery Reading subtest, the reading task from the Mini Mental State Examination, reading of words and sentences from the Arizona Battery for Communication Disorders of Dementia and the Wide Range Achievement Test – Reading. The analysis showed an overall mean effect of $\beta_{g}$ = 0.21 (95% ETI [-0.43, 0.78], BF = **0.287**). The heterogeneity parameters were $\tau_{Study}$= 0.40 [0.01, 1.24] and $\tau_{Study/ES}$= 0.30 [0.01, 0.99]. For τ parameters 95% confidence intervals are reported.

Reasoning

Abstraction Measures include Neurobehavior Cognitive Status Examination – Similarities, Mattis Dementia Rating Scale – Identities and Oddities, Dementia Rating Scale – Conceptualization, NEUROPSI – Similarities, and an analogies task.

Other Reasoning Measures encompassed the Test of Classification and Recall of Pictures, the Neurobehavior Cognitive Status Examination Judgment task and the Weigel’s Test.

Table 15. Results of the Meta-Regressions for the Measures of Reasoning

| Test | VaD Subtype | N_studies_ | N_participants_ | $\beta_{g}$ | 95% ETI | | Bayes Factor | Study references |
| --- | --- | --- | --- | --- | --- | --- | --- | --- |
|  |  |  |  |  | LB | UB |  |  |
| WAIS |  |  |  |  |  | |  |  |
| 1. Similarities | VaD^S^ | 3 | 413 | 0.08 | -0.52 | 0.62 | **0.210** | 89, 100, 117 |
| 1. Picture Arrangement | VaD^S^ | 4 | 455 | 0.34 | -0.32 | 0.96 | 0.408 | 36, 65, 81, 117 |
| 1. Comprehension | VaD^S^ | 3 | 370 | -0.02 | -0.63 | 0.67 | **0.233** | 36, 81, 117 |
|  | $\tau_{Study}$= 0.31 [0.02, 0.82]  $\tau_{Study/ES}$= 0.19 [0.01, 0.56] | | | | | | |  |
| Wisconsin Card Sorting Test: Categories |  |  |  |  |  |  |  |  |
|  | VaD^S^ | 5 | 338 | 0.08 | -0.60 | 0.70 | **0.231** | 67, 89, 93, 95, 106 |
|  | $\tau_{Study}$= 0.40 [0.03, 1.05] | | | | | | |  |
| Raven’s Progressive Matrices |  |  |  |  |  |  |  |  |
|  | VaD^S^ | 6 | 617 | 0.00 | -0.26 | 0.32 | **0.091** | 18, 87, 106, 110, 112, 114 |
|  | $\tau_{Study}$= 0.19 [0.01, 0.60] | | | | | | |  |
| Raven’s Colored Progressive Matrices |  |  |  |  |  |  |  |  |
|  | sVaD | 2 | 194 | 0.19 | -0.39 | 0.72 | **0.263** | 19, 42, 92, 93, 95 |
|  | VaD | 3 | 108 | 0.08 | -0.42 | 0.66 | **0.209** |  |
|  | $\tau_{Study}$= 0.28 [0.01, 0.91] | | | | | | |  |
| Attentional Matrices |  |  |  |  |  |  |  |  |
|  | sVaD | 3 | 510 | 0.00 | -0.47 | 0.62 | **0.153** | 14, 19, 110 |
|  | $\tau_{Study}$= 0.39 [0.05, 1.17] | | | | | | |  |
| Frontal Assessment Battery: Abstraction |  |  |  |  |  |  |  |  |
|  | VaD | 3 | 365 | -0.15 | -0.69 | 0.55 | **0.261** | 12, 31, 67 |
|  | $\tau_{Study}$= 0.49 [0.08, 1.39] | | | | | | |  |
| Other Abstraction Measures |  |  |  |  |  |  |  |  |
|  | VaD | 4 | 137 | -0.23 | -0.76 | 0.39 | **0.304** | 35, 41, 80, 106 |
|  | $\tau_{Study}$= 0.43 [0.02, 1.28] | | | | | | |  |
| Other Reasoning Measures |  |  |  |  |  |  |  |  |
|  | VaD | 3 | 134 | 0.19 | -0.61 | 0.98 | **0.307** | 14, 35, 114 |
|  | $\tau_{Study}$= 0.37 [0.01, 1.34]  $\tau_{Study/ES}$= 0.61 [0.13, 1.44] | | | | | | |  |
| **Notes:**  ETI stands for 95% equal-tailed credibility interval.  For τ parameters 95% confidence intervals are reported.  ^S^ – denotes the moderator difference in dementia severity between AD and VaD  WAIS: Wechsler Adult Intelligence Scale | | | | | | | | |

Executive Functioning

Arithmetic tasks included calculation tasks from the NEUROPSI-Test battery and the Neurobehavior Cognitive Status Examination as well as a serial sevens task. Cognitive control in verbal fluency were a difference score between phonemic and the fluency benefit due to category cueing on a semantic fluency task. Cognitive control in memory included measures of clustering in free recall. Flexibility measures included measures of perseverative errors on the Wisconsin Card Sorting Test, on the Test of Classification and Recall of Pictures as well as perseverative errors in verbal fluency and word definition tasks. For cognitive estimation a Cognitive Estimates Test and a temporal rules induction task were included. The Executive Interview (EXIT25) and a mean measure of executive function were analysed in the category of Global Measures of Executive Functioning. Other Measures of Verbal Working Memory included the attention task from the Mini Mental State Examination, an auditory detection test and the working memory index from the Wechsler Memory Scale – III. Other Visual Working Memory measures included a Sternberg task and the Knox cubes. Finally, Sequencing tasks included NEUROPSI-Test Battery Sequence task and the Wechsler Memory Scale III Letter-Number Sequencing.

Table 16. Results of the Meta-Regressions for the Measures of Executive Functioning

| Test | VaD Subtype | N_studies_ | N_participants_ | $\beta_{g}$ | 95% ETI | | Bayes Factor | | Study references |
| --- | --- | --- | --- | --- | --- | --- | --- | --- | --- |
|  |  |  |  |  | LB | UB |  | |  |
| WAIS |  |  |  |  |  | |  | |  |
| 1. Arithmetic & Total Digits |  |  |  |  |  |  |  | | 56, 117 |
|  | VaD | 2 | 360 | 0.30 | -0.38 | 0.87 | 0.527 | |  |
|  | $\tau_{Study}$= 0.36 [0.02, 1.37] | | | | | | | |  |
| Trail Making Test - B |  |  |  |  |  |  |  | |  |
|  | sVaD^S^ | 3 | 345 | 0.15 | -0.25 | 0.59 | **0.220** | | 6, 42, 55, 77, 83, 84, 89, 104, 106, 120 |
|  | VaD^S^ | 6 | 868 | 0.07 | -0.25 | 0.38 | **0.125** | |  |
|  | VCI^S^ | 3 | 752 | 0.02 | -0.36 | 0.39 | **0.136** | |  |
|  | $\tau_{Study}$= 0.18 [0.01, 0.45]  $\tau_{Study/ES}$= 0.14 [0.01, 0.40] | | | | | | | |  |
| Frontal Assessment Battery |  |  |  |  |  |  |  | |  |
|  | sVaD | 5 | 606 | -0.26 | -0.88 | 0.43 | **0.312** | | 12, 27, 28, 31, 44, 63, 67, 104 |
|  | VaD | 3 | 430 | -0.30 | -1.02 | 0.52 | 0.408 | |  |
|  | $\tau_{Study}$= 0.72 [0.39, 1.33] | | | | | | | |  |
| Stroop – Interference |  |  |  |  |  |  |  | |  |
|  | sVaD | 2 | 181 | -0.12 | -0.51 | 0.26 | **0.185** | | 55, 87, 89, 118, 120 |
|  | VaD | 3 | 605 | 0.04 | -0.25 | 0.32 | **0.094** | |  |
|  | $\tau_{Study}$= 0.14 [0.00, 0.50] | | | | | | | |  |
| Wechsler Memory Scale |  |  |  |  |  |  |  | |  |
| 1. Executive Functioning (Mental Control and Accuracy Indices) | | | | |  |  |  | |  |
|  | VaD^S^ | 5 | 247 | 0.48 | 0.07 | 0.82 | 2.68 | | 46, 48, 73, 81, 100 |
| 1. Verbal Working Memory (Digit Span Backwards) | | | |  |  |  |  | |  |
|  | VaD^S^ | 5 | 267 | 0.44 | 0.07 | 0.83 | 2.51 | | 48, 57, 81, 106, 112 |
| 1. Visual Working Memory | | | |  |  |  |  | |  |
|  | VaD^S^ | 3 | 443 | 0.22 | -0.12 | 0.60 | 0.371 | | 48, 81, 116 |
|  | $\tau_{Study}$= 0.18 [0.01, 0.51]  $\tau_{Study/ES}$= 0.15 [0.01, 0.41] | | | | | | | |  |
| Digit Span Backwards |  |  |  |  |  |  |  | |  |
|  | MID^S,E^ | 2 | 68 | 0.42 | -0.07 | 0.89 | 0.819 | | 2, 14, 19, 22, 42, 63, 80, 83, 87, 88, 89, 93, 97, 104, 110, 114, 117, 118 |
|  | sVaD^S,E^ | 9 | 1151 | 0.14 | -0.02 | 0.33 | **0.298** | |  |
|  | VaD^S,E^ | 7 | 225 | 0.33 | 0.12 | 0.52 | **9.38** | |  |
|  | $\tau_{Study}$=0.05 [0.00, 0.20] | | | | | | | |  |
| Visual Span |  |  |  |  |  |  |  | |  |
|  | MID^S^ | 2 | 68 | -0.56 | -1.43 | 0.28 | 0.868 | | 14, 17, 19, 27, 28, 63, 87, 88, 93, 97, 112, 114 |
|  | sVaD^S^ | 7 | 945 | -0.13 | -0.62 | 0.31 | **0.207** | |  |
|  | VaD^S^ | 4 | 350 | -0.06 | -0.60 | 0.60 | **0.211** | |  |
|  | $\tau_{Study}$= 0.52 [0.11, 0.94]  $\tau_{Study/ES}$= 0.16 [0.01, 0.54] | | | | | | | |  |
| Maze Tasks |  |  |  |  |  |  |  |  |  |
|  | VaD | 3 | 274 | 0.74 | -0.39 | 1.74 | 1.57 | | 3, 63, 114 |
|  | $\tau_{Study}$= 0.88 [0.25, 2.49] | | | | | | | |  |
| Graphical Sequence Test |  |  |  |  |  |  |  | |  |
|  | sVaD | 2 | 152 | 0.82 | 0.02 | 1.47 | **3.11** | | 25, 46 |
|  | $\tau_{Study}$= 0.36 [0.01, 1.51] | | | | | | | |  |
| Benton Visual Retention Test | |  |  |  |  |  |  | |  |
|  | VaD | 3 | 116 | 0.34 | -0.36 | 0.84 | 0.465 | | 42, 106, 122 |
|  | $\tau_{Study}$= 0.33 [0.01, 1.21] | | | | | | | |  |
| Repetition of Words and Sentences | |  |  |  |  |  |  | |  |
|  | VaD^S^ | 5 | 401 | -0.40 | -0.95 | 0.21 | 0.790 | | 35, 42, 62, 65, 80 |
| 1. Severity |  |  |  | 0.29 | -0.16 | 0.72 | 0.517 | |  |
|  | $\tau_{Study}$= 0.33 [0.01, 1.10]  $\tau_{Study/ES}$= 0.33 [0.01, 1.04] | | | | | | | |  |
| Arithmetic |  |  |  |  |  |  |  | |  |
|  | VaD | 4 | 181 | -0.01 | -0.55 | 0.55 | **0.176** | | 35, 80, 93, 118 |
|  | $\tau_{Study}$= 0.30 [0.01, 1.02]  $\tau_{Study/ES}$= 0.26 [0.01, 0.86] | | | | | | | |  |
| Cognitive Control – Memory | |  |  |  |  |  |  | |  |
| 1. Verbal Fluency |  |  |  |  |  |  |  | |  |
|  | VaD^S^ | 3 | 194 | 0.38 | -0.18 | 0.96 | 0.644 | | 97, 113 |
| 1. Clustering in Free Recall | |  |  |  |  |  |  | |  |
|  | VaD^S^ | 2 | 150 | -0.02 | -0.55 | 0.47 | **0.163** | | 2, 25 |
|  | $\tau_{Study}$= 0.20 [0.01, 0.68]  $\tau_{Study/ES}$= 0.31 [0.06, 0.70] | | | | | | | |  |
| Flexibility |  |  |  |  |  |  |  | |  |
|  | VaD | 4 | 174 | 0.01 | -0.50 | 0.77 | **0.218** | | 9, 14, 67, 93 |
|  | $\tau_{Study}$= 0.36 [0.01, 1.12]  $\tau_{Study/ES}$= 0.51 [0.05, 1.24] | | | | | | | |  |
| Cognitive Estimation |  |  |  |  |  |  |  | |  |
|  | VaD | 2 | 174 | -0.01 | -1.27 | 1.11 | **0.305** | | 87, 89 |
|  | $\tau_{Study}$= 0.97 [0.09, 3.43] | | | | | | | |  |
| Global Measures of Executive Functioning | |  |  |  |  |  |  | |  |
|  | VaD | 3 | 241 | -0.14 | -0.54 | 0.33 | **0.189** | | 79, 82, 97 |
|  | $\tau_{Study}$= 0.24 [0.01, 0.89] | | | | | | | |  |
| Set Maintenance |  |  |  |  |  |  |  | |  |
|  | VaD | 3 | 214 | 0.54 | -0.51 | 1.57 | 0.716 | | 14, 73, 114 |
|  | $\tau_{Study}$= 0.90 [0.11, 2.59]  $\tau_{Study/ES}$= 0.25 [0.01, 0.88] | | | | | | | |  |
| Other Measures of Verbal Working Memory | |  |  |  |  |  |  | |  |
|  | VaD | 3 | 413 | 0.46 | 0.04 | 0.80 | 2.66 | | 48, 62, 63 |
|  | $\tau_{Study}$= 0.20 [0.01, 0.79] | | | | | | | |  |
| Other Measures of Visual Working Memory | |  |  |  |  |  |  | |  |
|  | VaD | 2 | 61 | -0.19 | -0.97 | 0.63 | **0.287** | | 40, 83 |
|  | $\tau_{Study}$= 0.29 [0.01, 0.96] | | | | | | | |  |
| Sequencing |  |  |  |  |  |  |  | |  |
|  | sVaD | 2 | 80 | 0.20 | -0.54 | 0.84 | **0.281** | | 48, 80 |
|  | $\tau_{Study}$= 0.42 [0.01, 0.63] | | | | | | | |  |
| **Notes:**  ETI stands for 95% equal-tailed credibility interval.  For τ parameters 95% confidence intervals are reported.  ^S^ – denotes the moderator difference in dementia severity between AD and VaD  WAIS: Wechsler Adult Intelligence Scale | | | | | | | | | |

### Executive Functioning: Quality Sensitivity Analysis

Table 16. Results of the Meta-Regressions for the Measures of Executive Functioning

| Test | VaD Subtype | N_studies_ | N_participants_ | $\beta_{g}$ | 95% ETI | | Bayes Factor | Study references |
| --- | --- | --- | --- | --- | --- | --- | --- | --- |
|  |  |  |  |  | LB | UB |  |  |
| Digit Span Backwards |  |  |  |  |  |  |  |  |
|  | VaD | 2 | 154 | 0.21 | -0.56 | 0.91 | **0.298** | 88, 106 |
|  | $\tau_{Study}$ = 0.44 [0.02, 1.63] | | | | | | |  |
| Flexibility |  |  |  |  |  |  |  |  |
|  | VaD | 2 | 117 | 0.08 | -0.84 | 0.97 | 0.584 | 77, 106 |
|  | $\tau_{Study}$= 0.67 [0.05, 2.21] | | | | | | |  |
| Visual Working Memory | |  |  |  |  |  |  |  |
|  | VaD | 3 | 179 | -0.11 | -0.71 | 0.55 | **0.219** | 40, 88, 106 |
|  | $\tau_{Study}$= 0.34 [0.01, 1.23]  $\tau_{Study/ES}$= 0.25 [0.01, 0.86] | | | | | | |  |
| **Notes:**  ETI stands for 95% equal-tailed credibility interval.  For τ parameters 95% confidence intervals are reported. | | | | | | | | |

Memory

General Measures of Verbal Memory included the Neurobehavior Cognitive Status Examination – Memory scale, the Arizona Battery for Communication Disorders of Dementia – Verbal Memory scores and a general verbal memory measure.

Other Measures of Verbal Learning included learning and registration measures, the Arizona Battery for Communication Disorders of Dementia – Word Learning measure, the Brief Cognitive Screening Battery-Edu – Learning score, the Mini Mental State Examination – Immediate Recall, the Memory Assessment Scales – Mean total Recall and the California Verbal Learning Test – Total recall – i.e., learning over trials.

Other Measures of Episodic Memory: Prose included the Babcock Story Recall, the Prose Memory Test, the Arizona Battery for Communication Disorders of Dementia – Story Retelling and other measures of story, paragraph, and prose recall.

Other Measures of Episodic Memory: Word Lists included the Arizona Battery for Communication Disorders of Dementia – Word Learning Free Recall measure, the Brief Cognitive Screening Battery-Edu, the Free Cued Selective Reminding Test, the Memory Assessment Scales – List Learning, the Mini Mental State Examination – Delayed Memory, the NEUROPSI-Test Battery – free recall measures, the Seoul Verbal Learning Test and other list learning tasks.

Other Measures of Cued Recall included the California Verbal Learning Test – Cued Recall, the Shopping List Test, the Memory Assessment Scales – Cued Recall, the NEUROPSI-Test battery cued recall and a cued recall task.

Global Measures of Associative Memory included a word-pair learning task and the global Verbal Paired Association score from the Wechsler Memory Scale – Revised.

The Global Measures of Memory included the Brief Cognitive Screening Battery-Edu – Incidental Recall measures, the Immediate Memory Index from the Wechsler Memory Scale – III and the Pursuit Rotor Learning Task.

The Visual Associative Memory measures included: the Visual Association Memory Test, the Placing Test, a visual association test and the Visual Paired Association from the Wechsler Memory Scale – Revised.

Other Measure of Visual Memory Included the Test of Classification and Recall of Pictures, the Corsi Supraspan, the Placing Test, the Consortium to Establish a Registry for Alzheimer's Disease cognitive test battery – visual memory, the Seven Minute Screen, the NEUROPSI-Test battery – Figure Recall, the 7/24 Spatial Recall Test, the Graphemic Cued Recall and a delayed recall task.

Other Measures of Visual Recognition Memory Included the Faces Scores from the Wechsler Memory Scale – III, the Rey Osterrieth Complex Figure Recognition and recognition memory for faces tasks.

Other Measures of Semantic Memory included: a Famous Faces Test, Wechsler Adult Intelligence Scale: Information Test, a Sorting Test, a Word-Picture Matching Test, Word Definitions and a Stem Completion Priming Task.

Finally, Other Measures of Verbal Recognition Memory included the Arizona Battery for Communication Disorders of Dementia – Recognition of learned words task, the NEUROPSI-Test battery – recognition task, the Seoul Verbal Learning Test – Recognition and a recognition task.

Table 21. Results of the Meta-Regressions for Measures of Verbal Episodic and Semantic Memory

| Test | VaD Subtype | N_studies_ | N_participants_ | $\beta_{g}$ | 95% ETI | | Bayes Factor | | Study references | |  |
| --- | --- | --- | --- | --- | --- | --- | --- | --- | --- | --- | --- |
|  |  |  |  |  | LB | UB |  | |  | |  |
| Wechsler Memory Scale |  |  |  |  |  | |  | |  | |  |
| 1. Verbal Memory | VaD^S^ | 2 | 118 | -0.12 | -1.10 | 0.75 | 0.354 | | 56, 81 | |  |
| 1. Paired Associates I | VaD^S^ | 3 | 515 | -0.31 | -0.88 | 0.25 | 0.453 | | 48, 55, 93 | |  |
| 1. Paired Associates II | VaD^S^ | 2 | 483 | -0.37 | -0.95 | 0.28 | 0.525 | | 48, 55 | |  |
| 1. Logical Memory I | VaD^S^ | 5 | 701 | -0.30 | -0.78 | 0.24 | 0.391 | | 30, 48, 55, 77, 89, 114 | |  |
| 1. Logical Memory II | VaD^S^ | 6 | 771 | -0.55 | -1.05 | -0.06 | 2.56 | | 47, 48, 55, 77, 114 | |  |
|  | $\tau_{Study}$= 0.53 [0.13, 1.00]  $\tau_{Study/ES}$= 0.24 [0.01, 0.63] | | | | | | | |  | |  |
| 1. Visual Memory | VaD | 2 | 77 | 0.35 | -0.29 | 0.98 | 0.503 | | 81, 87 | |  |
| 1. Visual Memory Immediate Recall | VaD | 3 | 162 | -0.51 | -0.97 | 0.02 | 1.81 | | 48, 93, 114 | |  |
| 1. Visual Memory Delayed Recall | VaD | 3 | 162 | -0.32 | -0.79 | 0.17 | 0.552 | | 48, 93, 114 | |  |
|  | $\tau_{Study}$= 0.20 [0.01, 0.65]  $\tau_{Study/ES}$= 0.16 [0.01, 0.51] | | | | | | | |  | |  |
| Ray’s Auditory Verbal Learning Test | |  |  |  |  |  |  | |  | |  |
| 1. Learning | VaD^S^ | 6 | 634 | -0.18 | -0.62 | 0.34 | **0.277** | | 28, 87, 89, 92, 104, 112 | |  |
| 1. Delayed Recall | VaD^S^ | 6 | 634 | -0.39 | -0.85 | 0.11 | 0.806 | |  |  |  |
|  | $\tau_{Study}$= 0.40 [0.07, 0.92]  $\tau_{Study/ES}$= 0.17 [0.01, 0.52] | | | | | | | |  | |  |
| California Verbal Learning Test | |  |  |  |  |  |  | |  | |  |
| 1. Immediate Recall | VaD | 2 | 96 | -0.52 | -1.63 | 0.49 | 0.858 | | 83, 84 | |  |
| 1. Delayed Recall | VaD | 3 | 128 | -0.76 | -1.61 | 0.34 | 1.45 | | 83, 84, 93 | |  |
|  | $\tau_{Study}$= 0.71 [0.05, 2.14]  $\tau_{Study/ES}$= 0.38 [0.01, 1.26] | | | | | | | |  | |  |
| CERAD Word List |  |  |  |  |  |  |  | |  | |  |
| 1. Learning | VaD | 4 | 228 | -0.08 | -0.44 | 0.23 | **0.158** | | 57, 113, 120 | |  |
| 1. Forgetting | VaD | 3 | 152 | -0.46 | -0.85 | -0.03 | 1.40 | | 57, 113 | |  |
| 1. Immediate Recall | VaD | 3 | 181 | 0.09 | -0.28 | 0.51 | **0.190** | | 113, 120 | |  |
| 1. Delayed Recall | VaD | 4 | 228 | -0.23 | -0.59 | 0.16 | **0.327** | | 57, 113, 120 | |  |
|  | $\tau_{Study}$= 0.17 [0.01, 0.57]  $\tau_{Study/ES}$= 0.10 [0.00, 0.30] | | | | | | | |  | |  |
| Hopkins Verbal Learning Test | |  |  |  |  |  |  | |  | |  |
| 1. Learning | VaD | 3 | 198 | -0.38 | -1.06 | 0.33 | 0.616 | | 6, 26, 68 | |  |
|  | $\tau_{Study}$= 0.33 [0.01, 1.18]  $\tau_{Study/ES}$= 0.45 [0.04, 1.18] | | | | | | | |  | |  |
| Addenbrook’s Cognitive Examination | |  |  |  |  |  |  | |  | |  |
| 1. Memory | VaD | 3 | 202 | -0.67 | -3.15 | 1.20 | 1.09 | | 1, 49, 70 | |  |
|  | $\tau_{Study}$= 2.62 [1.07, 6.47] | | | | | | | |  | |  |
| Fuld Object Memory Examination | |  |  |  |  |  |  | |  | |  |
| 1. Learning | VaD | 2 | 98 | -0.15 | -1.11 | 0.65 | **0.298** | | 52, 77 | |  |
|  | $\tau_{Study}$= 0.48 [0.02, 1.85]  $\tau_{Study/ES}$= 0.34 [0.01, 1.19] | | | | | | | |  | |  |
| General Measures of Verbal Memory | |  |  |  |  |  |  | |  | |  |
|  | VaD | 3 | 148 | -0.45 | -1.34 | 0.59 | 0.623 | | 35, 42, 97 | |  |
|  | $\tau_{Study}$= 0.81 [0.08, 2.35] | | | | | | | |  | |  |
| Other Measures of Verbal Learning | |  |  |  |  |  |  | |  | |  |
|  | VaD^S^ | 6 | 465 | -0.48 | -1.00 | 0.06 | 1.48 | | 2, 22, 42, 62, 93, 97 | |  |
|  | $\tau_{Study}$= 0.39 [0.02, 1.14]  $\tau_{Study/ES}$= 0.27 [0.01, 0.76] | | | | | | | |  | |  |
| Other Measures of Episodic Memory: Prose | |  |  |  |  |  |  | |  | |  |
| 1. Immediate Recall |  |  |  |  |  |  |  | |  | |  |
|  | sVaD | 2 | 476 | -0.50 | -1.00 | 0.02 | 1.69 | | 42, 63, 93, 110 | |  |
|  | VaD | 2 | 75 | -0.33 | -0.96 | 0.26 | 0.461 | |  |  |  |
| 1. Delayed Recall |  |  |  |  |  |  |  | |  | |  |
|  | sVaD | 4 | 670 | -0.70 | -1.12 | -0.27 | **13.11** | | 19, 42, 63, 93, 95, 110, 112, 115 | |  |
|  | VaD | 5 | 965 | -0.42 | -0.87 | -0.09 | 2.47 | |  |  |  |
|  | $\tau_{Study}$= 0.30 [0.03, 0.63]  $\tau_{Study/ES}$= 0.17 [0.01, 0.48] | | | | | | | |  | |  |
| Other Measures of Episodic Memory: Word Lists | | |  |  |  |  |  | |  | |  |
| 1. Immediate Recall |  |  |  |  |  |  |  | |  | |  |
|  | sVaD^S,E^ | 5 | 698 | -0.38 | -0.62 | -0.10 | 2.79 | | 2, 41, 42, 47, 62, 63, 80, 97, 110, 118 | |  |
|  | VaD^S,E^ | 5 | 410 | -0.35 | -0.64 | -0.03 | 1.45 | |  |  |  |
| 1. Delayed Recall |  |  |  |  |  |  |  | |  | |  |
|  | sVaD^S,E^ | 7 | 805 | -0.64 | -0.88 | -0.36 | **72.97** | | 22, 42, 63, 67, 72, 80, 95, 97, 106, 110, 118 | |  |
|  | VaD^S,E^ | 4 | 235 | -0.61 | -0.97 | -0.26 | **22.71** | |  |  |  |
| 1. Severity |  |  |  | 0.36 | 0.17 | 0.56 | **11.48** | |  | |  |
|  | $\tau_{Study}$= 0.18 [0.01, 0.43]  $\tau_{Study/ES}$= 0.09 [0.00, 0.25] | | | | | | | |  | |  |
| Other Measures of Cued Recall of Word Lists | | |  |  |  |  |  | |  | |  |
| 1. Immediate Recall | VaD^S^ | 2 | 82 | -0.61 | -1.45 | 0.18 | 1.32 | | 2, 83 | |  |
| 1. Delayed Recall | VaD^S^ | 5 | 246 | -0.41 | -0.87 | 0.11 | 0.840 | | 41, 72, 80, 83, 97 | |  |
|  | $\tau_{Study}$= 0.27 [0.01, 0.86]  $\tau_{Study/ES}$= 0.27 [0.01, 0.85] | | | | | | | |  | |  |
| Global Measures of Associative Memory | | |  |  |  |  |  | |  | |  |
|  | VaD | 2 | 43 | -0.95 | -1.83 | 0.11 | 2.95 | | 18, 81 | |  |
|  | $\tau_{Study}$= 0.47 [0.01, 1.93] | | | | | | | |  | |  |
| Discriminability – *d’* |  |  |  |  |  |  |  | |  | |  |
| 1. Verbal |  |  |  |  |  |  |  | |  | |  |
|  | sVaD | 4 | 489 | -0.76 | -1.26 | -0.26 | **11.50** | | 2, 26, 46, 72, 82, 110 | |  |
|  | VaD | 2 | 168 | 0.27 | -0.27 | 0.84 | 0.414 | |  |  |  |
| - 1. sVaD – VaD |  |  |  | -1.08 | -1.82 | -0.32 |  | |  | |  |
| 1. Visual |  |  |  |  |  |  |  | |  | |  |
|  | VaD | 3 | 230 | 0.24 | -0.29 | 0.73 | **0.298** | | 37, 42, 82 | |  |
|  | $\tau_{Study}$= 0.28 [0.02, 0.74]  $\tau_{Study/ES}$= 0.21 [0.01, 0.60] | | | | | | | |  | |  |
| Wechsler Memory Scale Recognition | |  |  |  |  |  |  | |  | |  |
| 1. Verbal | VaD | 2 | 85 | -1.12 | -1.75 | 0.04 | **4.72** | | 30, 48 | |  |
|  | $\tau_{Study}$= 0.42 [0.01, 1.84] | | | | | | | |  | |  |
| Rey’s Auditory Verbal Learning Test: Recognition | | |  |  |  |  |  | |  | |  |
|  | VaD | 2 | 174 | 0.17 | -0.43 | 0.78 | **0.286** | | 87, 89 | |  |
|  | $\tau_{Study}$= 0.33 [0.01, 1.37] | | | | | | | |  | |  |
| California Verbal Learning Test: Recognition | | |  |  |  |  |  | |  | |  |
|  | VAD | 2 | 96 | -0.03 | -0.70 | 0.58 | **0.212** | | 83, 84 | |  |
|  | $\tau_{Study}$= 0.36 [0.01, 1.40] | | | | | | | |  | |  |
| CERAD: Recognition |  |  |  |  |  |  |  | |  | |  |
|  | VaD | 4 | 228 | -0.92 | -1.38 | -0.43 | **19.21** | | 57, 113, 120 | |  |
|  | $\tau_{Study}$= 0.27 [0.01, 0.87] | | | | | | | |  | |  |
| Rey Osterrieth Complex Figure Test | |  |  |  |  |  |  | |  | |  |
| 1. Immediate Recall |  |  |  |  |  |  |  | |  | |  |
|  | VaD^S^ | 2 | 238 | -0.02 | -0.81 | 0.74 | **0.289** | | 28, 118 | |  |
| 1. Delayed Recall |  |  |  |  |  |  |  | |  | |  |
|  | VaD^S^ | 5 | 732 | -0.26 | -0.84 | 0.31 | 0.369 | | 28, 88, 90, 110, 118 | |  |
|  | $\tau_{Study}$= 0.47 [0.03, 1.23]  $\tau_{Study/ES}$= 0.34 [0.02, 0.98] | | | | | | | |  | |  |
| Recognition Hits and False Alarms | |  |  |  |  |  |  | |  | |  |
| 1. Hits |  |  |  |  |  |  |  | |  | |  |
|  | VaD | 3 | 158 | 0.08 | -0.55 | 0.69 | **0.209** | | 2, 37, 57 | |  |
| 1. False Alarms |  |  |  |  |  |  |  | |  | |  |
|  | VaD | 4 | 262 | -0.37 | -0.87 | 0.24 | 0.589 | | 2, 25, 37, 57 | |  |
|  | $\tau_{Study}$= 0.44 [0.03, 1.12]  $\tau_{Study/ES}$= 0.29 [0.03, 0.66] | | | | | | | |  | |  |
| Other Measures of Visual Recognition Memory | | |  |  |  |  |  | |  | |  |
|  | VaD | 4 | 321 | -0.19 | -0.66 | 0.29 | **0.280** | | 48, 89, 98, 118 | |  |
|  | $\tau_{Study}$= 0.25 [0.01, 0.85]  $\tau_{Study/ES}$= 0.27 [0.01, 0.81] | | | | | | | |  | |  |
| Other Measures of Verbal Recognition Memory | | |  |  |  |  |  | |  | |  |
|  | VaD | 4 | 226 | -0.43 | -0.82 | 0..04 | 1.43 | | 22, 42, 80, 118 | |  |
|  | $\tau_{Study}$= 0.21 [0.01, 0.78]  $\tau_{Study/ES}$= 0.18 [0.01, 0.59] | | | | | | | |  | |  |
| Memory Intrusions |  |  |  |  |  |  |  | |  | |  |
| 1. Verbal |  |  |  |  |  |  |  | |  | |  |
|  | sVaD | 5 | 597 | -0.35 | -0.77 | 0.02 | 0.965 | | 6, 25, 57, 72, 93, 113, 120 | |  |
|  | VaD | 4 | 215 | -0.43 | -0.90 | 0.05 | 1.16 | |  |  |  |
|  | $\tau_{Study}$= 0.27 [0.02, 0.62]  $\tau_{Study/ES}$= 0.23 [0.02, 0.56] | | | | | | | |  | |  |
| Semantic Memory |  |  |  |  |  |  |  |  | |  | |
| 1. WAIS |  |  |  |  |  |  |  | |  | |  |
| - 1. Information | VaD^S,E^ | 3 | 357 | -0.27 | -0.97 | 0.45 | 0.358 | | 84, 93, 116 | |  |
| - 1. Vocabulary | VaD^S,E^ | 5 | 457 | 0.13 | -0.48 | 0.77 | **0.272** | | 48, 56, 93, 116, 122 | |  |
| 1. Semantic Fluency Errors | VaD^S,E^ | 2 | 124 | -0.36 | -1.37 | 0.51 | 0.534 | | 9, 25 | |  |
| 1. ABCD | VaD^S,E^ | 2 | 252 | 0.12 | -1.22 | 1.41 | 0.482 | | 42, 104 | |  |
| 1. Lexical Decision Task | VaD^S,E^ | 2 | 67 | -0.08 | -1.11 | 0.84 | 0.380 | | 18, 57 | |  |
| 1. Other Measures | VaD^S,E^ | 4 | 179 | -0.79 | -1.47 | -0.09 | **3.85** | | 9, 18, 56, 98 | |  |
|  | $\tau_{Study}$= 0.95 [0.35, 1.70]  $\tau_{Study/ES}$= 0.17 [0.01, 0.55] | | | | | | | |  | |  |
| Global Measures of Memory |  |  |  |  |  |  |  | |  | |  |
|  | VaD | 2 | 95 | -0.62 | -1.19 | 0.22 | 1.08 | | 42, 48 | |  |
|  | $\tau_{Study}$= 0.38 [0.01, 1.52] | | | | | | | |  | |  |
| Visual Associative Memory |  |  |  |  |  |  |  | |  | |  |
|  | VaD | 4 | 354 | -1.01 | -1.49 | -0.43 | **20.02** | | 22, 26, 81, 104 | |  |
|  | $\tau_{Study}$= 0.25 [0.01, 0.85]  $\tau_{Study/ES}$= 0.22 [0.01, 0.72] | | | | | | | |  | |  |
| Other Measures of Visual Memory | |  |  |  |  |  |  | |  | |  |
| 1. Forgetting | VaD^S^ | 2 | 77 | -0.53 | -1.12 | 0.01 | 1.92 | | 16, 57 | |  |
| 1. Delayed Recall | VaD^S^ | 5 | 177 | -0.85 | -1.29 | -0.32 | **13.67** | | 14, 57, 79, 80, 93 | |  |
| 1. Cued Recall | VaD^S^ | 2 | 259 | -0.63 | -1.22 | 0.10 | 1.55 | | 18, 85 | |  |
|  | $\tau_{Study}$= 0.18 [0.01, 0.60]  $\tau_{Study/ES}$= 0.20 [0.01, 0.60] | | | | | | | |  | |  |
| **Notes:**  ETI stands for 95% equal-tailed credibility interval.  For τ parameters 95% confidence intervals are reported.  ^S,E^ – denote considered moderators   1. S = difference in dementia severity between AD and VaD; 2. E = difference in years of education between AD and VaD.   CERAD: CERAD: Consortium to Establish a Registry for Alzheimer's Disease cognitive test battery | | | | | | | | | | |  |

### Memory –Verbal Delayed Recall: Quality Sensitivity Analysis

The final sample contained two studies [72, 106] and 123 subjects. Measures used were the Memory Assessment Scales – Delayed Recall and the Free and Cued Selective Reminding Test. The analysis showed an overall mean effect of $\beta_{g}$ = -0.01 (95% ETI [-0.66, 0.67], BF = 0.421) and the heterogeneity parameter $\tau_{Study}$= 0.39 [0.01, 1.48]. For τ parameters 95% confidence intervals are reported.

# Table of included Studies

Table 22. Table of included Studies and their Nottingham-Ottawa Scale Ratings. S: Selectivity, C: Comparability, E: Exposure

| **Study Number** | **Study** | **S** | **C** | **E** |
| --- | --- | --- | --- | --- |
| 1 | Alexopoulos, P., Greim, B., Nadler, K., Martens, U., Krecklow, B., Domes, G., Herpertz, S., & Kurz, A. (2006). Validation of the Addenbrookes Cognitive Examination for Detecting Early Alzheimers Disease and Mild Vascular Dementia in a German Population. *Dement Geriatr Cogn Disord*, *22*(5–6), 385–391. https://doi.org/10.1159/000095642 | 3 | 0 | 1 |
| 2 | Almkvist, O., Fratiglioni, L., Agüero-Torres, H., Viitanen, M., & Bäckman, L. (1999). Cognitive support at episodic encoding and retrieval: Similar patterns of utilization in community-based samples of Alzheimer’s disease and vascular dementia patients. *Journal of Clinical and Experimental Neuropsychology*, *21*(6), 816–830. https://doi.org/10.1076/jcen.21.6.816.862 | 4 | 1 | 1 |
| 3 | Almkvist, O., Wahlund, L. O., Andersson-Lundman, G., Basun, H., & Bäckman, L. (1992). White-matter hyperintensity and neuropsychological functions in dementia and healthy aging. *Archives of Neurology*, *49*(6), 626–632. https://doi.org/10.1001/archneur.1992.00530300062011 | 4 | 0 | 1 |
| 4 | Anor, C. J., O’Connor, S., Saund, A., Tang-Wai, D. F., Keren, R., & Tartaglia, M. C. (2017). Neuropsychiatric Symptoms in Alzheimer Disease, Vascular Dementia, and Mixed Dementia. *Neuro-Degenerative Diseases*, *17*(4–5), 127–134. https://doi.org/10.1159/000455127 | 4 | 0 | 1 |
| 5 | Barber, R., Scheltens, P., Gholkar, A., Ballard, C., McKeith, I., Ince, P., Perry, R., & O’Brien, J. (1999). White matter lesions on magnetic resonance imaging in dementia with Lewy bodies, Alzheimer’s disease, vascular dementia, and normal aging. *Journal of Neurology, Neurosurgery, and Psychiatry*, *67*(1), 66–72. https://doi.org/10.1136/jnnp.67.1.66 | 3 | 1 | 1 |
| 6 | Barr, A., Benedict, R., Tune, L., & Brandt, J. (1992). Neuropsychological differentiation of Alzheimer’s disease from vascular dementia. *International Journal of Geriatric Psychiatry*, *7*(9), 621–627. https://doi.org/10.1002/gps.930070903 | 4 | 0 | 3 |
| 7 | Batchelder, W. H., Chosak-Reiter, J., Shankle, W. R., & Dick, M. B. (1997). A multinomial modeling analysis of memory deficits in Alzheimer’s disease and vascular dementia. *The Journals of Gerontology. Series B, Psychological Sciences and Social Sciences*, *52*(5), P206-215. https://doi.org/10.1093/geronb/52b.5.p206 | 3 | 1 | 1 |
| 8 | Beber, B. C., Kochhann, R., Matias, B., & Chaves, M. L. F. (2016). The Clock Drawing Test: Performance differences between the free-drawn and incomplete-copy versions in patients with MCI and dementia. *Dementia & Neuropsychologia*, *10*(3), 227–231. https://doi.org/10.1590/S1980-5764-2016DN1003009 | 2 | 0 | 2 |
| 9 | Bentham, P. W., Jones, S., & Hodges, J. R. (1997). A COMPARISON OF SEMANTIC MEMORY IN VASCULAR DEMENTIA AND DEMENTIA OF ALZHEIMER’S TYPE. *International Journal of Geriatric Psychiatry*, 6. | 3 | 1 | 1 |
| 10 | Binetti, G., Bianchetti, A., Padovani, A., Lenzi, G., De Leo, D., & Trabucchi, M. (1993). Delusions in Alzheimer’s disease and multi-infarct dementia. *Acta Neurologica Scandinavica*, *88*(1), 5–9. https://doi.org/10.1111/j.1600-0404.1993.tb04177.x | 4 | 0 | 1 |
| 11 | Binetti, G., Padovani, A., Magni, E., Bianchetti, A., Scuratti, A., Lenzi, G. L., & Trabucchi, M. (1995). Delusions and dementia: Clinical and CT correlates. *Acta Neurologica Scandinavica*, *91*(4), 271–275. https://doi.org/10.1111/j.1600-0404.1995.tb07003.x | 4 | 0 | 2 |
| 12 | Boban, M., Malojčić, B., Mimica, N., Vuković, S., & Zrilić, I. (2012). The Frontal Assessment Battery in the Differential Diagnosis of Dementia. *Journal of Geriatric Psychiatry and Neurology*, *25*(4), 201–207. https://doi.org/10.1177/0891988712464821 | 4 | 0 | 1 |
| 13 | Buckwalter, J. G., Rizzo, A. A., McCleary, R., Shankle, R., Dick, M., & Henderson, V. W. (1996). Gender comparisons of cognitive performances among vascular dementia, Alzheimer disease, and older adults without dementia. *Archives of Neurology*, *53*(5), 436–439. https://doi.org/10.1001/archneur.1996.00550050066025 | 3 | 1 | 1 |
| 14 | Cannatà, A. P., Alberoni, M., Franceschi, M., & Mariani, C. (2002). Frontal impairment in subcortical ischemic vascular dementia in comparison to Alzheimer’s disease. *Dementia and Geriatric Cognitive Disorders*, *13*(2), 101–111. https://doi.org/10.1159/000048641 | 4 | 1 | 1 |
| 15 | Caputo, M., Monastero, R., Mariani, E., Santucci, A., Mangialasche, F., Camarda, R., Senin, U., & Mecocci, P. (2008). Neuropsychiatric symptoms in 921 elderly subjects with dementia: A comparison between vascular and neurodegenerative types. *Acta Psychiatrica Scandinavica*, *117*(6), 455–464. https://doi.org/10.1111/j.1600-0447.2008.01175.x | 4 | 2 | 2 |
| 16 | Carlesimo, G. A., Fadda, L., Bonci, A., & Caltagirone, C. (1993). Differential rates of forgetting from long-term memory in Alzheimer’s and multi-infarct dementia. *The International Journal of Neuroscience*, *73*(1–2), 1–11. https://doi.org/10.3109/00207459308987206 | 2 | 0 | 1 |
| 17 | Carlesimo, G. A., Fadda, L., Lorusso, S., & Caltagirone, C. (1994). Verbal and spatial memory spans in Alzheimer’s and multi-infarct dementia. *Acta Neurologica Scandinavica*, *89*(2), 132–138. https://doi.org/10.1111/j.1600-0404.1994.tb01648.x | 3 | 0 | 1 |
| 18 | Carlesimo, G. A., Fadda, L., Marfia, G. A., & Caltagirone, C. (1995). Explicit memory and repetition priming in dementia: Evidence for a common basic mechanism underlying conscious and unconscious retrieval deficits. *Journal of Clinical and Experimental Neuropsychology*, *17*(1), 44–57. https://doi.org/10.1080/13803399508406580 | 3 | 0 | 1 |
| 19 | Carolis, A. D., Cipollini, V., Donato, N., Sepe-Monti, M., Orzi, F., & Giubilei, F. (2017). Cognitive proﬁles in degenerative dementia without evidence of small vessel pathology and small vessel vascular dementia. *Neurol Sci*, 8. | 3 | 0 | 2 |
| 20 | Chin, J., & Suh, M. K. (2005). The Closing-in Phenomenon in Alzheimer’s Disease and Vascular Dementia. *Journal of Clinical Neurology*, *1*(1), 8. | 3 | 0 | 2 |
| 21 | Chosak Reiter, J. (2000). Measuring cognitive processes underlying picture naming in Alzheimer’s and cerebrovascular dementia: A general processing tree approach. *Journal of Clinical and Experimental Neuropsychology*, *22*(3), 351–369. https://doi.org/10.1076/1380-3395(200006)22:3;1-V;FT351 | 3 | 1 | 1 |
| 22 | Chuang, W.-L., Wang, H.-M., Hsieh, Y.-C., Chang, T.-F., Kuo, H.-C., & Huang, C.-C. (2006). Visual association memory test in differentiating early stage of Alzheimer’s disease from vascular dementia. *Acta Neurologica Taiwanica*, *15*(2), 98–104. | 4 | 1 | 1 |
| 23 | Chui, H. C., Zarow, C., Mack, W. J., Ellis, W. G., Zheng, L., Jagust, W. J., Mungas, D., Reed, B. R., Kramer, J. H., Decarli, C. C., Weiner, M. W., & Vinters, H. V. (2006). Cognitive impact of subcortical vascular and Alzheimer’s disease pathology. *Annals of Neurology*, *60*(6), 677–687. https://doi.org/10.1002/ana.21009 | 4 | 2 | 2 |
| 24 | Claus, J. J., Staekenborg, S. S., Roorda, J. J., Stevens, M., Herderschee, D., van Maarschalkerweerd, W., Schuurmans, L., Tielkes, C. E. M., Koster, P., Bavinck, C., & Scheltens, P. (2016). Low Prevalence of Mixed Dementia in a Cohort of 2,000 Elderly Patients in a Memory Clinic Setting. *Journal of Alzheimer’s Disease*, *50*(3), 10. https://doi.org/10.3233/JAD-150796 | 4 | 2 | 2 |
| 25 | Davis, K. L., Price, C. C., Kaplan, E., & Libon, D. J. (2002). Error analysis of the nine-word California Verbal Learning Test (CVLT-9) among older adults with and without dementia. *The Clinical Neuropsychologist*, *16*(1), 81–89. https://doi.org/10.1076/clin.16.1.81.8330 | 3 | 0 | 1 |
| 26 | De Jager, C. A., Hogervorst, E., Combrinck, M., & Budge, M. M. (2003). Sensitivity and specificity of neuropsychological tests for mild cognitive impairment, vascular cognitive impairment and Alzheimer’s disease. *Psychological Medicine*, *33*(6), 1039–1050. https://doi.org/10.1017/s0033291703008031 | 3 | 0 | 0 |
| 27 | De Lucia, N., Grossi, D., Milan, G., & Trojano, L. (2020). The closing-in phenomenon in constructional tasks in dementia and mild cognitive impairment. *Neuropsychology*, *34*(2), 168–175. https://doi.org/10.1037/neu0000596 | 4 | 0 | 1 |
| 28 | De Lucia, N., Grossi, D., & Trojano, L. (2014). The genesis of closing-in in Alzheimer disease and vascular dementia: A comparative clinical and experimental study. *Neuropsychology*, *28*(2), 312–318. https://doi.org/10.1037/neu0000036 | 3 | 1 | 1 |
| 29 | DeFigueiredo, R. J. P., Shankle, W. R., Maccato, A., Dick, M. B., Mundkur, P., Mena, I., & Cotman, C. W. (1995). Neural-network-based classification of cognitively normal, demented, Alzheimer disease and vascular dementia from single photon emission with computed tomography image data from brain. *Proc. Natl. Acad. Sci. USA*, 5. | 2 | 0 | 2 |
| 30 | Doddy, R. S., Massman, P. J., Mawad, M., & Nance, M. (1998). Cognitive consequences of subcortical magnetic resonance imaging changes in Alzheimer’s disease: Comparison to small vessel ischemic vascular dementia. *Neuropsychiatry, Neuropsychology, and Behavioral Neurology*, *11*(4), 191–199. | 3 | 1 | 1 |
| 31 | D’Onofrio, G., Panza, F., Sancarlo, D., Addante, F., Solfrizzi, V., Cantarini, C., Mangiacotti, A., Lauriola, M., Cascavilla, L., Paris, F., Lozupone, M., Daniele, A., Greco, A., & Seripa, D. (2018). Executive Dysfunction Detected with the Frontal Assessment Battery in Alzheimer’s Disease Versus Vascular Dementia. *Journal of Alzheimer’s Disease*, *62*(2), 699–711. https://doi.org/10.3233/JAD-170365 | 4 | 1 | 1 |
| 32 | D’Onofrio, G., Sancarlo, D., Addante, F., Ciccone, F., Cascavilla, L., Paris, F., Picoco, M., Nuzzaci, C., Elia, A. C., Greco, A., Chiarini, R., Panza, F., & Pilotto, A. (2014). Caregiver burden characterization in patients with Alzheimer’s disease or vascular dementia. *Int J Geriatr Psychiatry*, 9. | 4 | 0 | 1 |
| 33 | D’Onofrio, G., Sancarlo, D., Panza, F., Copetti, M., Cascavilla, L., Paris, F., Seripa, D., Matera, M. G., Solfrizzi, V., Pellegrini, F., & Pilotto, A. (2012). Neuropsychiatric symptoms and functional status in Alzheimer’s disease and vascular dementia patients. *Current Alzheimer Research*, *9*(6), 759–771. https://doi.org/10.2174/156720512801322582 | 4 | 1 | 1 |
| 34 | Duro, D., Tábuas-Pereira, M., Freitas, S., Santiago, B., Botelho, M. A., & Santana, I. (2018). Validity and Clinical Utility of Different Clock Drawing Test Scoring Systems in Multiple Forms of Dementia. *Journal of Geriatric Psychiatry and Neurology*, *31*(3), 114–122. https://doi.org/10.1177/0891988718774432 | 3 | 0 | 2 |
| 35 | Engel, P., Cummings, J. L., Villanueva-Meyer, J., & Mena, I. (1993). Single photon emission computed tomography in dementia: Relationship of perfusion to cognitive deficits. *Journal of Geriatric Psychiatry and Neurology*, *6*(3), 144–151. https://doi.org/10.1177/089198879300600303 | 3 | 0 | 1 |
| 36 | Erker, G. J., Searight, H. R., & Peterson, P. (1995). Patterns of neuropsychological functioning among patients with multi-infarct and Alzheimer’s dementia: A comparative analysis. *International Psychogeriatrics*, *7*(3), 393–406. https://doi.org/10.1017/s1041610295002146 | 3 | 0 | 2 |
| 37 | Fahlander, K., Wahlin, A., Almkvist, O., & Bäckman, L. (2002). Cognitive functioning in Alzheimer’s disease and vascular dementia: Further evidence for similar patterns of deficits. *Journal of Clinical and Experimental Neuropsychology*, *24*(6), 734–744. https://doi.org/10.1076/jcen.24.6.734.8404 | 1 | 0 | 0 |
| 38 | Fernández-Martínez, M., Castro, J., Molano, A., Zarranz, J. J., Rodrigo, R. M., & Ortega, R. (2008). Prevalence of neuropsychiatric symptoms in Alzheimer’s disease and vascular dementia. *Current Alzheimer Research*, *5*(1), 61–69. https://doi.org/10.2174/156720508783884585 | 4 | 1 | 1 |
| 39 | Fischer, P., Gatterer, G., Marterer, A., & Danielczyk, W. (1988). Nonspecificity of semantic impairment in dementia of Alzheimer’s type. *Archives of Neurology*, *45*(12), 1341–1343. https://doi.org/10.1001/archneur.1988.00520360059012 | 3 | 2 | 1 |
| 40 | Fitten, L. J., Perryman, K. M., Wilkinson, C. J., Little, R. J., Burns, M. M., Pachana, N., Mervis, J. R., Malmgren, R., Siembieda, D. W., & Ganzell, S. (1995). Alzheimer and vascular dementias and driving. A prospective road and laboratory study. *JAMA*, *273*(17), 1360–1365. | 3 | 1 | 2 |
| 41 | Flynn, F. G., Cummings, J. L., & Gornbein, J. (1991). Delusions in dementia syndromes: Investigation of behavioral and neuropsychological correlates. *The Journal of Neuropsychiatry and Clinical Neurosciences*, *3*(4), 364–370. https://doi.org/10.1176/jnp.3.4.364 | 4 | 0 | 1 |
| 42 | Freitas, M. I. D., Porto, C. S., Oliveira, M. O., Brucki, S. M. D., Mansur, L. L., Nitrini, R., & Radanovic, M. (2018). Linguistic abilities in major vascular cognitive impairment: A comparative study with Alzheimer’s disease. *Acta Neurologica Belgica*, *118*(3), 465–473. https://doi.org/10.1007/s13760-018-0977-x | 3 | 1 | 1 |
| 43 | Freitas, S., Prieto, G., Simões, M. R., & Santana, I. (2014). Psychometric properties of the Montreal Cognitive Assessment (MoCA): An analysis using the Rasch model. *The Clinical Neuropsychologist*, *28*(1), 65–83. https://doi.org/10.1080/13854046.2013.870231 | 3 | 1 | 1 |
| 44 | Fukui, T., Lee, E., Kitamura, M., Hosoda, H., Bokui, C., Ikusu, K., & Okita, K. (2009). Visuospatial dysfunction may be a key in the differentiation between Alzheimer’s disease and subcortical cognitive impairment in moderate to severe stages. *Dementia and Geriatric Cognitive Disorders*, *28*(4), 288–294. https://doi.org/10.1159/000245157 | 4 | 1 | 2 |
| 45 | Gill, D. P., Hubbard, R. A., Koepsell, T. D., Borrie, M. J., Petrella, R. J., Knopman, D. S., & Kukull, W. A. (2013). Differences in rate of functional decline across three dementia types. *Alzheimer’s & Dementia: The Journal of the Alzheimer’s Association*, *9*(5 Suppl), S63-71. https://doi.org/10.1016/j.jalz.2012.10.010 | 3 | 2 | 1 |
| 46 | Giovannetti, T., Schmidt, K. S., Gallo, J. L., Sestito, N., & Libon, D. J. (2006). Everyday action in dementia: Evidence for differential deficits in Alzheimer’s disease versus subcortical vascular dementia. *Journal of the International Neuropsychological Society: JINS*, *12*(1), 45–53. https://doi.org/10.1017/S1355617706060012 | 3 | 0 | 1 |
| 47 | Godinho, C., Gorczevski, I., Heisler, A., Cerveira, M. O., & Chaves, M. L. (2010). Clinical and demographic characteristics of elderly patients with dementia assisted at an outpatient clinic in Southern Brazil. *Dementia & Neuropsychologia*, *4*(1), 42–46. https://doi.org/10.1590/S1980-57642010DN40100007 | 3 | 0 | 1 |
| 48 | Gonçalves, C., Pinho, M. S., Cruz, V., Gens, H., Oliveira, F., Pais, J., Rente, J., Santana, I., & Santos, J. M. (2017). Portuguese version of Wechsler Memory Scale-3rd edition’s utility with demented elderly adults. *Applied Neuropsychology. Adult*, *24*(3), 212–225. https://doi.org/10.1080/23279095.2015.1135440 | 3 | 1 | 1 |
| 49 | Gonçalves, C., Pinho, M. S., Cruz, V., Pais, J., Gens, H., Oliveira, F., Santana, I., Rente, J., & Santos, J. M. (2015). The Portuguese version of Addenbrooke’s Cognitive Examination-Revised (ACE-R) in the diagnosis of subcortical vascular dementia and Alzheimer’s disease. *Neuropsychology, Development, and Cognition. Section B, Aging, Neuropsychology and Cognition*, *22*(4), 473–485. https://doi.org/10.1080/13825585.2014.984652 | 3 | 0 | 1 |
| 50 | Hall, J. R., Harvey, M., Vo, H. T., & O’Bryant, S. E. (2011). Performance on a measure of category fluency in cognitively impaired elderly. *Neuropsychology, Development, and Cognition. Section B, Aging, Neuropsychology and Cognition*, *18*(3), 353–361. https://doi.org/10.1080/13825585.2011.557495 | 3 | 0 | 1 |
| 51 | Hargrave, Rita, Stoeklin, Maria, Haan, Mary, & Reed, Bruce. (2000). Psychopathology and functional impairment in Alzheimer’s disease and related dementias. *Journal of Mental Health and Aging*, *6*(3), 201–2011. | 3 | 2 | 1 |
| 52 | Hassing, L., & Bäckman, L. (1997). Episodic memory functioning in population-based samples of very old adults with Alzheimer’s disease and vascular dementia. *Dementia and Geriatric Cognitive Disorders*, *8*(6), 376–383. https://doi.org/10.1159/000106658 | 1 | 0 | 1 |
| 53 | Heinik, J., Solomesh, I., & Berkman, P. (2004). Correlation between the CAMCOG, the MMSE, and three clock drawing tests in a specialized outpatient psychogeriatric service. *Arch. Gerontol. Geriatr.*, 8. | 3 | 0 | 2 |
| 54 | Heinik, J., Solomesh, I., Raikher, B., & Lin, R. (2002). Can clock drawing test help to differentiate between dementia of the Alzheimer’s type and vascular dementia? A preliminary study. *Int J Geriatr Psychiatry*, 5. | 3 | 1 | 2 |
| 55 | Heyanka, D. J., Mackelprang, J. L., Golden, C. J., & Marke, C. D. (2010). Distinguishing Alzheimer’s Disease From Vascular Dementia: An Exploration of Five Cognitive Domains. *International Journal of Neuroscience*, 7. | 2 | 1 | 1 |
| 56 | Hier, D. B., Warach, J. D., Gorelick, P. B., & Thomas, J. (1989). Predictors of survival in clinically diagnosed Alzheimer’s disease and multi-infarct dementia. *Archives of Neurology*, *46*(11), 1213–1216. https://doi.org/10.1001/archneur.1989.00520470073030 | 4 | 0 | 1 |
| 57 | Hildebrandt, H., Haldenwanger, A., & Eling, P. (2009). False recognition helps to distinguish patients with Alzheimer’s disease and amnestic MCI from patients with other kinds of dementia. *Dementia and Geriatric Cognitive Disorders*, *28*(2), 159–167. https://doi.org/10.1159/000235643 | 3 | 1 | 1 |
| 58 | Isik, A. T., Cankurtaran, M., Bozoglu, E., Comert, B., Doruk, H., & Mas, M. R. (2007). Is there any relation between insulin resistance and cognitive function in the elderly? *International Psychogeriatrics*, *19*(4), 745–756. https://doi.org/10.1017/S1041610207005406 | 3 | 0 | 2 |
| 59 | Jagust, W. J., Zheng, L., Harvey, D. J., Mack, W. J., Vinters, H. V., Weiner, M. W., Ellis, W. G., Zarow, C., Mungas, D., Reed, B. R., Kramer, J. H., Schuff, N., DeCarli, C., & Chui, H. C. (2008). Neuropathological basis of magnetic resonance images in aging and dementia. *Annals of Neurology*, *63*(1), 72–80. https://doi.org/10.1002/ana.21296 | 4 | 0 | 1 |
| 60 | Jairani, P. S., Aswathy, P. M., Gopala, S., Verghese, J., & Mathuranath, P. S. (2016). Interaction with the MAPT H1H1 Genotype Increases Dementia Risk in APOE ε4 Carriers in a Population of Southern India. *Dement Geriatr Cogn Disord*, 10. | 4 | 0 | 2 |
| 61 | Jiang, S. (2017). Mismatch negativity as a potential neurobiological marker of early-stage Alzheimer disease and vascular dementia. *Neuroscience Letters*, 6. | 3 | 0 | 1 |
| 62 | Jones, S., Laukka, E. J., Small, B. J., Fratiglioni, L., & Bäckman, L. (2004). A preclinical phase in vascular dementia: Cognitive impairment three years before diagnosis. *Dementia and Geriatric Cognitive Disorders*, *18*(3–4), 233–239. https://doi.org/10.1159/000080021 | 2 | 1 | 2 |
| 63 | Kandiah, N., Narasimhalu, K., Lee, J., & Chen, C. L. P. H. (2009). Differences exist in the cognitive profile of mild Alzheimer’s disease and subcortical ischemic vascular dementia. *Dementia and Geriatric Cognitive Disorders*, *27*(5), 399–403. https://doi.org/10.1159/000210387 | 4 | 1 | 1 |
| 64 | Kazui, H., Yoshiyama, K., Kanemoto, H., Suzuki, Y., Sato, S., Hashimoto, M., Ikeda, M., Tanaka, H., Hatada, Y., Matsushita, M., Nishio, Y., Mori, E., Tanimukai, S., Komori, K., Yoshida, T., Shimizu, H., Matsumoto, T., Mori, T., Kashibayashi, T., … Tanaka, T. (2016). Differences of Behavioral and Psychological Symptoms of Dementia in Disease Severity in Four Major Dementias. *PLOS ONE*, 16. | 4 | 1 | 1 |
| 65 | Kertesz, A., & Clydesdale, S. (1994). Neuropsychological deficits in vascular dementia vs Alzheimer’s disease. Frontal lobe deficits prominent in vascular dementia. *Archives of Neurology*, *51*(12), 1226–1231. https://doi.org/10.1001/archneur.1994.00540240070018 | 4 | 0 | 1 |
| 66 | Kim, H.-J., Moon, W.-J., & Han, S.-H. (2013). Differential cholinergic pathway involvement in Alzheimer’s disease and subcortical ischemic vascular dementia. *Journal of Alzheimer’s Disease: JAD*, *35*(1), 129–136. https://doi.org/10.3233/JAD-122320 | 4 | 0 | 2 |
| 67 | Kugo, A., Terada, S., Ata, T., Ido, Y., Kado, Y., Ishihara, T., Hikiji, M., Fujisawa, Y., Sasaki, K., & Kuroda, S. (2007). Japanese version of the Frontal Assessment Battery for dementia. *Psychiatry Research*, 7. | 4 | 0 | 2 |
| 68 | Kuslansky, G., Katz, M., Verghese, J., Hall, C. B., Lapuerta, P., LaRuffa, G., & Lipton, R. B. (2004). Detecting dementia with the Hopkins Verbal Learning Test and the Mini-Mental State Examination. *Archives of Clinical Neuropsychology*, 16. | 3 | 1 | 1 |
| 69 | Kwak, Y. T. (2004). “Closing-in” phenomenon in Alzheimer’s disease and subcortical vascular dementia. *BMC Neurology*, 7. | 3 | 0 | 1 |
| 70 | Kwak, Y. T., Yang, Y., & Kim, G. W. (2010). Korean Addenbrooke’s Cognitive Examination Revised (K-ACER) for differential diagnosis of Alzheimer’s disease and subcortical ischemic vascular dementia. *Geriatrics & Gerontology International*, *10*(4), 295–301. https://doi.org/10.1111/j.1447-0594.2010.00624.x | 3 | 0 | 1 |
| 71 | Kweon, O. J., Youn, Y. C., Lim, Y. K., Lee, M.-K., & Kim, H. R. (2019). Clinical utility of serum hepcidin and iron profile measurements in Alzheimer’s disease. *Journal of the Neurological Sciences*, *403*, 85–91. https://doi.org/10.1016/j.jns.2019.06.008 | 4 | 0 | 2 |
| 72 | Lafosse, J. M., Reed, B. R., Mungas, D., Sterling, S. B., Wahbeh, H., & Jagust, W. J. (1997). Fluency and memory differences between ischemic vascular dementia and Alzheimer’s disease. *Neuropsychology*, *11*(4), 514–522. https://doi.org/10.1037//0894-4105.11.4.514 | 3 | 2 | 2 |
| 73 | Lamar, M., Price, C. C., Davis, K. L., Kaplan, E., & Libon, D. J. (2002). Capacity to maintain mental set in dementia. *Neuropsychologia*, *40*(4), 435–445. https://doi.org/10.1016/s0028-3932(01)00125-7 | 3 | 0 | 1 |
| 74 | Lee, D. R., McKeith, I., Mosimann, U., Ghosh-Nodyal, A., & Thomas, A. J. (2012). Examining carer stress in dementia: The role of subtype diagnosis and neuropsychiatric symptoms. *Int J Geriatr Psychiatry*, 7. | 3 | 0 | 2 |
| 75 | Libon, D. J., Bogdanoff, B., Cloud, B. S., Skalina, S., Giovannetti, T., Gitlin, H. L., & Bonavita, J. (1998). Declarative and procedural learning, quantitative measures of the hippocampus, and subcortical white alterations in Alzheimer’s disease and ischaemic vascular dementia. *Journal of Clinical and Experimental Neuropsychology*, *20*(1), 30–41. https://doi.org/10.1076/jcen.20.1.30.1490 | 3 | 0 | 1 |
| 76 | Lim, K.-B., Kim, J., Lee, H.-J., Yoo, J., Kim, H. S., Kim, C., & Lee, H. (2019). COWAT Performance of Persons with Alzheimer Dementia, Vascular Dementia, and Parkinson Disease Dementia According to Stage of Cognitive Impairment. *PM R*, 8. | 3 | 0 | 1 |
| 77 | Loewenstein, D. A., Acevedo, A., Agron, J., Issacson, R., Strauman, S., Crocco, E., Barker, W. W., & Duara, R. (2006). Cognitive profiles in Alzheimer’s disease and in mild cognitive impairment of different etiologies. *Dementia and Geriatric Cognitive Disorders*, *21*(5–6), 309–315. https://doi.org/10.1159/000091522 | 3 | 1 | 2 |
| 78 | Lukatela, K., Malloy, P., Jenkins, M., & Cohen, R. (1998). The naming deficit in early Alzheimer’s and vascular dementia. *Neuropsychology*, *12*(4), 565–572. https://doi.org/10.1037//0894-4105.12.4.565 | 3 | 0 | 1 |
| 79 | Matioli, M. N. P. S., & Caramelli, P. (2010). Limitations in differentiating vascular dementia from Alzheimer’s disease with brief cognitive tests. *Arquivos De Neuro-Psiquiatria*, *68*(2), 185–188. https://doi.org/10.1590/s0004-282x2010000200006 | 3 | 2 | 1 |
| 80 | Matioli, M. N. P. S., & Caramelli, P. (2012). NEUROPSI battery subtest profile in subcortical vascular dementia and Alzheimer’s disease. *Dementia & Neuropsychologia*, *6*(3), 170–174. https://doi.org/10.1590/S1980-57642012DN06030010 | 3 | 2 | 1 |
| 81 | Matsuda, O., Saito, M., & Sugishita, M. (1998). Cognitive deficits of mild dementia: A comparison between dementia of the Alzheimer’s type and vascular dementia. *Psychiatry and Clinical Neurosciences*, *52*(1), 87–91. https://doi.org/10.1111/j.1440-1819.1998.tb00978.x | 3 | 1 | 1 |
| 82 | McGuinness, B., Barrett, S. L., Craig, D., Lawson, J., & Passmore, A. P. (2010). Executive functioning in Alzheimer’s disease and vascular dementia. *Int J Geriatr Psychiatry*, 7. | 3 | 0 | 1 |
| 83 | Mendez, M. F., & Ashla-Mendez, M. (1991). Differences between multi-infarct dementia and Alzheimer’s disease on unstructured neuropsychological tasks. *Journal of Clinical and Experimental Neuropsychology*, *13*(6), 923–932. https://doi.org/10.1080/01688639108405108 | 3 | 0 | 2 |
| 84 | Mendez, M. F., Cherrier, M. M., & Perryman, K. M. (1997). Differences between Alzheimer’s disease and vascular dementia on information processing measures. *Brain and Cognition*, *34*(2), 301–310. https://doi.org/10.1006/brcg.1997.0923 | 3 | 0 | 1 |
| 85 | Meulen, E. F. J., Schmand, B., van Campen, J. P., de Koning, S. J., Ponds, R. W., Scheltens, P., & Verhey, F. R. (2004). The seven minute screen: A neurocognitive screening test highly sensitive to various types of dementia. *Journal of Neurology, Neurosurgery, and Psychiatry*, *75*(5), 700–705. https://doi.org/10.1136/jnnp.2003.021055 | 3 | 0 | 1 |
| 86 | Milan, G., Lamenza, F., Iavarone, A., Galeone, F., Lorè, E., de Falco, C., Sorrentino, P., & Postiglione, A. (2008). Frontal Behavioural Inventory in the differential diagnosis of dementia. *Acta Neurologica Scandinavica*, *117*(4), 260–265. https://doi.org/10.1111/j.1600-0404.2007.00934.x | 3 | 2 | 1 |
| 87 | Misciagna, S., Masullo, C., Giordano, A., & Silveri, M. C. (2005). Vascular dementia and Alzheimer’s disease: The unsolved problem of clinical and neuropsychological differential diagnosis. *The International Journal of Neuroscience*, *115*(12), 1657–1667. https://doi.org/10.1080/00207450590958501 | 3 | 1 | 1 |
| 88 | Muscoso, E. G., Costanzo, E., Daniele, O., Maugeri, D., Natale, E., & Caravaglios, G. (2006). Auditory event-related potentials in subcortical vascular cognitive impairment and in Alzheimer’s disease. *Journal of Neural Transmission (Vienna, Austria: 1996)*, *113*(11), 1779–1786. https://doi.org/10.1007/s00702-006-0574-7 | 3 | 1 | 2 |
| 89 | Nordlund, A., Rolstad, S., Klang, O., Lind, K., Hansen, S., & Wallin, A. (2007). Cognitive profiles of mild cognitive impairment with and without vascular disease. *Neuropsychology*, *21*(6), 706–712. https://doi.org/10.1037/0894-4105.21.6.706 | 4 | 0 | 1 |
| 90 | Nyenhuis, D. L., Gorelick, P. B., Freels, S., & Garron, D. C. (2002). Cognitive and functional decline in African Americans with VaD, AD, and stroke without dementia. *Neurology*, *58*(1), 56–61. https://doi.org/10.1212/wnl.58.1.56 | 3 | 2 | 1 |
| 91 | Okazaki, M., Kasai, M., Meguro, K., Yamaguchi, S., & Ishii, H. (2009). Disturbances in everyday life activities and sequence disabilities in tool use for Alzheimer disease and vascular dementia. *Cognitive and Behavioral Neurology: Official Journal of the Society for Behavioral and Cognitive Neurology*, *22*(4), 215–221. https://doi.org/10.1097/WNN.0b013e3181b278d4 | 3 | 0 | 1 |
| 92 | Osawa, A., Maeshima, S., Shimamoto, Y., Maeshima, E., Sekiguchi, E., Kakishita, K., Ozaki, F., & Moriwaki, H. (2004). Relationship between cognitive function and regional cerebral blood flow in different types of dementia. *Disability and Rehabilitation*, *26*(12), 739–745. https://doi.org/10.1080/09638280410001704331 | 1 | 0 | 1 |
| 93 | Padovani, A., Di Piero, V., Bragoni, M., Iacoboni, M., Gualdi, G. F., & Lenzi, G. L. (1995). Patterns of neuropsychological impairment in mild dementia: A comparison between Alzheimer’s disease and multi-infarct dementia. *Acta Neurologica Scandinavica*, *92*(6), 433–442. https://doi.org/10.1111/j.1600-0404.1995.tb00477.x | 3 | 0 | 1 |
| 94 | Park, J. H., Lee, S. B., Lee, T. J., Lee, D. Y., Jhoo, J. H., Youn, J. C., Choo, I. H., Choi, E. A., Jeong, J. W., Choe, J. Y., Woo, J. I., & Kim, K. W. (2007). Depression in vascular dementia is quantitatively and qualitatively different from depression in Alzheimer’s disease. *Dementia and Geriatric Cognitive Disorders*, *23*(2), 67–73. https://doi.org/10.1159/000097039 | 3 | 1 | 1 |
| 95 | Perri, R., Monaco, M., Fadda, L., Caltagirone, C., & Carlesimo, G. A. (2014). Neuropsychological correlates of behavioral symptoms in Alzheimer’s disease, frontal variant of frontotemporal, subcortical vascular, and lewy body dementias: A comparative study. *Journal of Alzheimer’s Disease: JAD*, *39*(3), 669–677. https://doi.org/10.3233/JAD-131337 | 4 | 1 | 1 |
| 96 | Porter, V. R., Buxton, W. G., Fairbanks, L. A., Strickland, T., O’Connor, S. M., & Rosenberg-Thompson, S. (2003). Frequency and Characteristics of Anxiety Among Patients With Alzheimer’s Disease and Related Dementias. *J Neuropsychiatry Clin Neurosci*, 7. | 3 | 1 | 1 |
| 97 | Ramirez-Gomez, L., Zheng, L., Reed, B., Kramer, J., Mungas, D., Zarow, C., Vinters, H., Ringman, J. M., & Chui, H. (2017). Neuropsychological Profiles Differentiate Alzheimer Disease from Subcortical Ischemic Vascular Dementia in an Autopsy-Defined Cohort. *Dement Geriatr Cogn Disord*, 11. | 3 | 0 | 1 |
| 98 | Ricker, J. H., Keenan, P. A., & Jacobson, M. W. (1994). Visuoperceptual-spatial ability and visual memory in vascular dementia and dementia of the Alzheimer type. *Neuropsychologia*, *32*(10), 1287–1296. https://doi.org/10.1016/0028-3932(94)90110-4 | 3 | 0 | 1 |
| 99 | Rojas, G., Bartoloni, L., Dillon, C., Serrano, C. M., Iturry, M., & Allegri, R. F. (2011). Clinical and economic characteristics associated with direct costs of Alzheimer’s, frontotemporal and vascular dementia in Argentina. *International Psychogeriatrics*, *23*(4), 554–561. https://doi.org/10.1017/S1041610210002012 | 4 | 0 | 1 |
| 100 | Schmidt, K. S., Gallo, J. L., Ferri, C., Giovannetti, T., Sestito, N., Libon, D. J., & Schmidt, P. S. (2005). The neuropsychological profile of alcohol-related dementia suggests cortical and subcortical pathology. *Dementia and Geriatric Cognitive Disorders*, *20*(5), 286–291. https://doi.org/10.1159/000088306 | 3 | 0 | 1 |
| 101 | Senanarong, V., Cummings, J. L., Fairbanks, L., Mega, M., Masterman, D. M., O’Connor, S. M., & Strickland, T. L. (2004). Agitation in Alzheimer’s disease is a manifestation of frontal lobe dysfunction. *Dementia and Geriatric Cognitive Disorders*, *17*(1–2), 14–20. https://doi.org/10.1159/000074080 | 3 | 1 | 1 |
| 102 | Shany-Ur, T., Poorzand, P., Grossman, S. N., Growdon, M. E., Jang, J. Y., Ketelle, R. S., Miller, B. L., & Rankin, K. P. (2012). Comprehension of insincere communication in neurodegenerative disease: Lies, sarcasm, and theory of mind. *Cortex; a Journal Devoted to the Study of the Nervous System and Behavior*, *48*(10), 1329–1341. https://doi.org/10.1016/j.cortex.2011.08.003 | 3 | 2 | 1 |
| 103 | Small, B. J., Viitanen, M., Winblad, B., & Bäckman, L. (1997). Cognitive changes in very old persons with dementia: The influence of demographic, psychometric, and biological variables. *Journal of Clinical and Experimental Neuropsychology*, *19*(2), 245–260. https://doi.org/10.1080/01688639708403855 | 3 | 1 | 1 |
| 104 | Smits, L. L., van Harten, A. C., Pijnenburg, Y. a. L., Koedam, E. L. G. E., Bouwman, F. H., Sistermans, N., Reuling, I. E. W., Prins, N. D., Lemstra, A. W., Scheltens, P., & van der Flier, W. M. (2015). Trajectories of cognitive decline in different types of dementia. *Psychological Medicine*, *45*(5), 1051–1059. https://doi.org/10.1017/S0033291714002153 | 3 | 1 | 1 |
| 105 | Stanzani Maserati, M., Matacena, C., Sambati, L., Oppi, F., Poda, R., De Matteis, M., & Gallassi, R. (2015). The Tree-Drawing Test (Koch’s Baum Test): A Useful Aid to Diagnose Cognitive Impairment. *Behavioural Neurology*, *2015*, 534681. https://doi.org/10.1155/2015/534681 | 4 | 1 | 2 |
| 106 | Starkstein, S. E., Sabe, L., Vazquez, S., Teson, A., Petracca, G., Chemerinski, E., Di Lorenzo, G., & Leiguarda, R. (1996). Neuropsychological, psychiatric, and cerebral blood flow findings in vascular dementia and Alzheimer’s disease. *Stroke*, *27*(3), 408–414. https://doi.org/10.1161/01.str.27.3.408 | 4 | 1 | 2 |
| 107 | Swartz, R. H., & Black, S. E. (2006). Anterior-medial thalamic lesions in dementia: Frequent, and volume dependently associated with sudden cognitive decline. *Journal of Neurology, Neurosurgery, and Psychiatry*, *77*(12), 1307–1312. https://doi.org/10.1136/jnnp.2006.091561 | 4 | 0 | 2 |
| 108 | Tanaka, N., Nakatsuka, M., Ishii, H., Nakayama, R., Hosaka, R., & Meguro, K. (2013). Clinical utility of the functional independence measure for assessment of patients with Alzheimer’s disease and vascular dementia. *Psychogeriatrics: The Official Journal of the Japanese Psychogeriatric Society*, *13*(4), 199–205. https://doi.org/10.1111/psyg.12012 | 2 | 0 | 2 |
| 109 | Tisato, V., Rimondi, E., Brombo, G., Volpato, S., Zurlo, A., Zauli, G., Secchiero, P., & Zuliani, G. (2016). Serum Soluble Tumor Necrosis Factor-Related Apoptosis-Inducing Ligand Levels in Older Subjects with Dementia and Mild Cognitive Impairment. *Dement Geriatr Cogn Disord*, 8. | 4 | 1 | 2 |
| 110 | Tosi, G., Borsani, C., Castiglioni, S., Daini, R., Franceschi, M., & Romano, D. (2020). Complexity in neuropsychological assessments of cognitive impairment: A network analysis approach. *Cortex; a Journal Devoted to the Study of the Nervous System and Behavior*, *124*, 85–96. https://doi.org/10.1016/j.cortex.2019.11.004 | 3 | 2 | 1 |
| 111 | Tu, M.-C., Huang, W.-H., Hsu, Y.-H., Lo, C.-P., Deng, J. F., & Huang, C.-F. (2017). Comparison of neuropsychiatric symptoms and diffusion tensor imaging correlates among patients with subcortical ischemic vascular disease and Alzheimer’s disease. *BMC Neurology*, *17*(1), 144. https://doi.org/10.1186/s12883-017-0911-5 | 4 | 1 | 1 |
| 112 | Vallotti, B., Mossello, E., Cantini, C., Moretti, G., Fumagalli, S., Caleri, V., Ungar, A., Bruscoli, M., Tilli, S., & Masotti, G. (2001). Determinants of functional status in Alzheimer’s disease and vascular dementia. *Archives of Gerontology and Geriatrics. Supplement*, *7*, 419–428. https://doi.org/10.1016/s0167-4943(01)00169-8 | 4 | 1 | 1 |
| 113 | Vanderploeg, R. D., Yuspeh, R. L., & Schinka, J. A. (2001). Differential episodic and semantic memory performance in Alzheimer’s disease and vascular dementias. *Journal of the International Neuropsychological Society: JINS*, *7*(5), 563–573. https://doi.org/10.1017/s135561770175504x | 3 | 2 | 1 |
| 114 | Villardita, C. (1993). Alzheimer’s disease compared with cerebrovascular dementia. Neuropsychological similarities and differences. *Acta Neurologica Scandinavica*, *87*(4), 299–308. https://doi.org/10.1111/j.1600-0404.1993.tb05512.x | 4 | 1 | 1 |
| 115 | Wei, M., Shi, J., Li, T., Ni, J., Zhang, X., Li, Y., Kang, S., Ma, F., Xie, H., Qin, B., Fan, D., Zhang, L., Wang, Y., & Tian, J. (2018). Diagnostic Accuracy of the Chinese Version of the Trail-Making Test for Screening Cognitive Impairment. *Journal of the American Geriatrics Society*, *66*(1), 92–99. https://doi.org/10.1111/jgs.15135 | 3 | 1 | 1 |
| 116 | Wiechmann, A., Hall, J. R., & O’Bryant, S. E. (2011). The utility of the spatial span in a clinical geriatric population. *Neuropsychology, Development, and Cognition. Section B, Aging, Neuropsychology and Cognition*, *18*(1), 56–63. https://doi.org/10.1080/13825585.2010.510556 | 3 | 1 | 1 |
| 117 | Yamashita, H., Hirono, N., Ikeda, M., Ikejiri, Y., Imamura, T., Shimomura, T., & Mori, E. (1997). Examining the diagnostic utility of the Fuld cholinergic deficit profile on the Japanese WAIS-R. *Journal of Clinical and Experimental Neuropsychology*, *19*(2), 300–304. https://doi.org/10.1080/01688639708403859 | 4 | 0 | 1 |
| 118 | Yoon, C. W., Shin, J. S., Kim, H. J., Cho, H., Noh, Y., Kim, G. H., Chin, J. H., Oh, S. J., Kim, J. S., Choe, Y. S., Lee, K.-H., Lee, J.-H., Seo, S. W., & Na, D. L. (2013). Cognitive deficits of pure subcortical vascular dementia vs. Alzheimer disease: PiB-PET-based study. *Neurology*, *80*(6), 569–573. https://doi.org/10.1212/WNL.0b013e3182815485 | 3 | 1 | 1 |
| 119 | Yoshida, H. (2011). Validation of Addenbrooke’s cognitive examination for detecting early dementia in a Japanese population. *Psychiatry Research*, 4. | 4 | 0 | 1 |
| 120 | Yuspeh, R. L., Vanderploeg, R. D., Crowell, T. A., & Mullan, M. (2002). Differences in executive functioning between Alzheimer’s disease and subcortical ischemic vascular dementia. *Journal of Clinical and Experimental Neuropsychology*, *24*(6), 745–754. https://doi.org/10.1076/jcen.24.6.745.8399 | 3 | 1 | 1 |
| 121 | Zanetti, O., Vallotti, B., Frisoni, G. B., Geroldi, C., Bianchetti, A., Pasqualetti, P., & Trabucchi, M. (1999). Insight in dementia: When does it occur? Evidence for a nonlinear relationship between insight and cognitive status. *The Journals of Gerontology. Series B, Psychological Sciences and Social Sciences*, *54*(2), P100-106. https://doi.org/10.1093/geronb/54b.2.p100 | 4 | 1 | 1 |
| 122 | Zekry, D., Duyckaerts, C., Belmin, J., Geoffre, C., Herrmann, F., Moulias, R., & Hauw, J.-J. (2003). The vascular lesions in vascular and mixed dementia: The weight of functional neuroanatomy. *Neurobiology of Aging*, 7. | 3 | 0 | 2 |
