# Supplementary material for: Neuropsychological differential diagnosis of Alzheimer’s disease and vascular dementia: a systematic review with meta-regressions
Source: Front Aging Neurosci. 2023 Nov 6;15:1267434. doi: 10.3389/fnagi.2023.1267434 (PMC10657839; doi:10.3389/fnagi.2023.1267434)
Supplement: Supplementary file 2 [file Table_2.DOCX]

**Supplementary Materials 2: Forest Plots**

**Contents**

Global Functioning 5

Clinical Dementia Rating Scale 5

Global Deterioration Scale 6

Dementia Rating Scale 6

Orientation to Time and Space 7

Activities of Daily Living 8

Affective Symptoms 10

Affective Symptoms: Quality Sensitivity Analysis 11

Disease Awareness 13

Neuropsychiatric Symptoms 13

Global Scores 13

Global Scores: Quality Sensitivity Analysis 14

Domains 15

Domains: Quality Sensitivity Analysis 17

Apraxia 19

Apraxia 19

Facial Apraxia 19

Ideomotor Apraxia 20

Motor Functioning 21

Visuo-Spatial Processing 22

Wechsler Adult Intelligence Scale 22

Clock Drawing Test 23

Rey-Osterrieth Complex Figure Test 24

Visual Object and Space Perception Battery 25

CERAD: Constructional Praxis 25

Addenbrooke’s Cognitive Examination - R 26

Judgment of Line Orientation 26

Hooper’s Test 27

Line Bisection 27

Other Copy Measures 28

Other Construction Measures 29

Other Measures of Visuo-Spatial Processing 30

Visuo-Spatial Processing: Quality Sensitivity Analysis 31

Intelligence Measures 32

Attention 33

Trail Making Test – A 33

Digit Span Forward 34

Digit Symbol Substitution Test 35

Symbol Digit Modalities Test 35

Choice Reaction Time 36

Other Measures of Selective Attention 36

Continuous Performance Tests 37

Other Measures of Sustained Attention 37

Other Measures of Visual Attention 38

Other Measures of Attention 38

Attention: Quality Sensitivity Analysis 39

Processing Speed 39

Stroop Task Word Reading and Colour Naming 39

Simple Reaction Time 40

Other Measures of Processing Speed 40

Language Production 41

Fluency: Multi-infarct Dementia 41

Fluency: Vascular Dementia 42

Fluency: Subcortical Vascular Dementia 43

Fluency: Vascular Cognitive Impairment 44

Boston Naming Test 45

Other Naming Measures 47

Addenbrooke’s Cognitive Examination 48

Writing 48

Other Measures of Language Production 49

Boston Naming Test: Quality Sensitivity Analysis 49

Phonemic Fluency: Quality Sensitivity Analysis 50

Language Comprehension 50

Token Test 50

Aphasia Inventories 51

Other Measures of Language Comprehension 51

Reading 52

Reasoning 53

Wechsler Adult Intelligence Scale 53

Wisconsin Card Sorting Test 54

Raven’s Progressive Matrices 54

Raven’s Coloured Progressive Matrices 55

Attentional Matrices 55

Frontal Assessment Battery: Abstraction 56

Other Abstraction Measures 56

Other Reasoning Measures 57

Executive Functioning 57

Wechsler Adult Intelligence Scale 57

Trail Making Test 58

Frontal Assessment Battery 59

Stroop – Interference Condition 59

Wechsler Memory Scale 60

Digit Span Backwards 61

Visual Span 62

Maze Tasks 62

Graphical Sequence Test 63

Repetition of Words and Sentences 63

Arithmetic 64

Cognitive Control of Memory 64

Cognitive Flexibility 65

Cognitive Estimation 65

Global Measures of Executive Functioning 66

Set Maintenance 66

Other Measures of Verbal Working Memory 67

Other Measures of Visual Working Memory 67

Sequencing 67

Benton Visual Retention Test 68

Quality Sensitivity Analyses 68

Digit Span Backwards 68

Visual Working Memory 69

Cognitive Flexibility 69

Memory 70

Wechsler Memory Scale: Verbal 70

Rey’s Auditory Verbal Learning Test 71

California Verbal Learning Test 72

CERAD Word List 73

Hopkin’s Verbal Learning Test 74

Addenbrooke’s Cognitive Examination 74

Fuld Object Memory Examination 75

General Verbal Memory Measures 75

Other Measures of Verbal Learning 76

Other Measures of Episodic Memory: Prose 77

Other Measures of Episodic Memory: Word Lists 78

Other Measures of Cued Recall of Word Lists 79

Memory Intrusions 80

Semantic Memory 81

Global Measures of Memory 82

Quality Sensitivity Analysis: Verbal Delayed Recall 82

Visual Memory 83

Wechsler Memory Scale 83

Rey-Osterrieth Complex Figure Test 84

Visual Associative Memory 84

Other Measures of Visual Memory 85

Recognition Memory 86

Wechsler Memory Scale 86

Rey’s Auditory Verbal Learning Test 86

California Verbal Learning Test 87

CERAD: Word List Recognition 87

Discriminability (*d’*) 88

Recognition Hits and False Alarms 89

Other Measures of Visual Recognition Memory 89

Other Measures of Verbal Recognition Memory 90

# Global Functioning

## Clinical Dementia Rating Scale


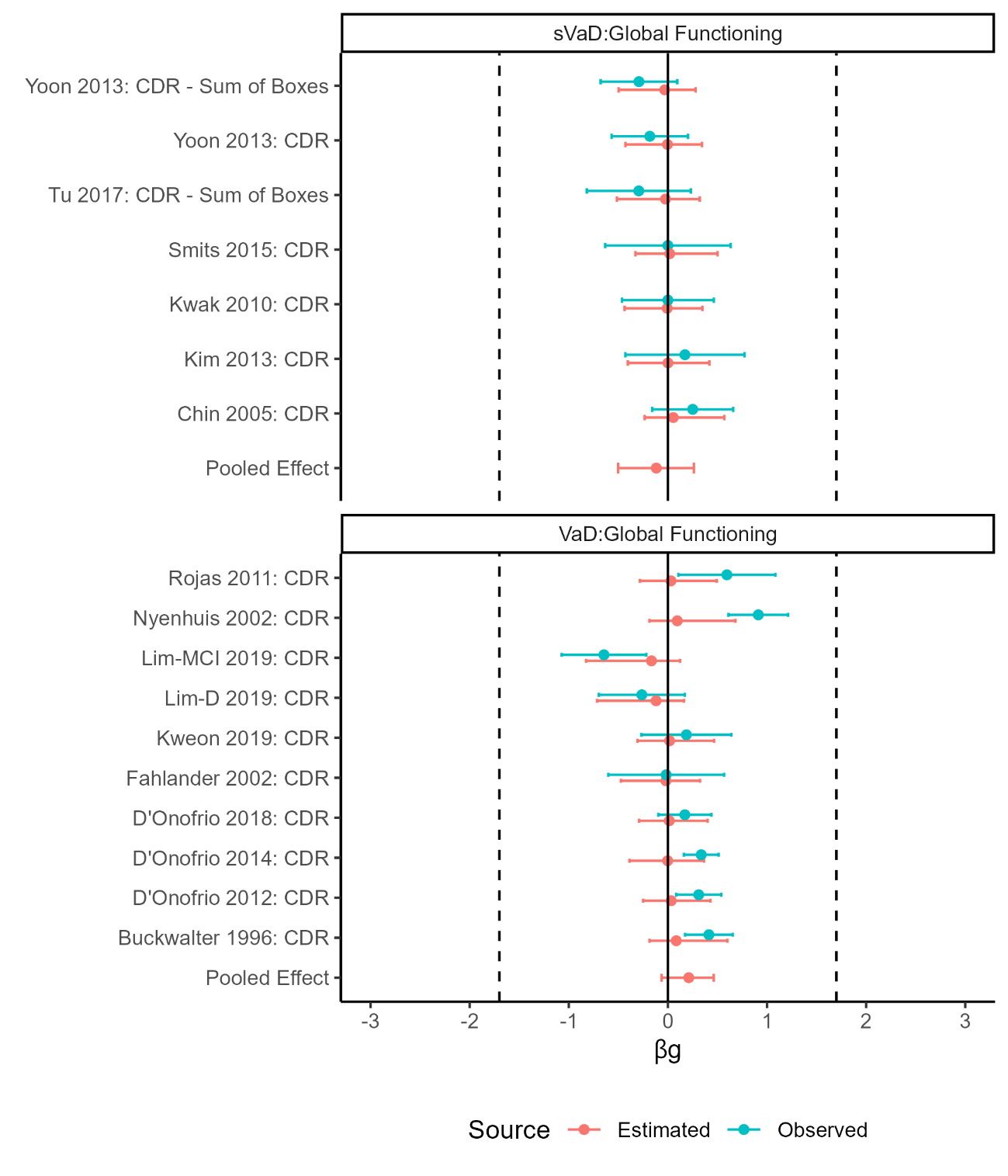


Figure 1. Forest plot for the Clinical Dementia Rating Scale. Regression coefficients and 95% confidence intervals are displayed. Dashed vertical lines show the lower and upper bound of the Region of Practical Equivalence set at ±1.7 *g*. Estimated effect sizes are regression coefficient estimates and Observed effects are the effect sizes and confidence intervals from the included studies.

## Global Deterioration Scale


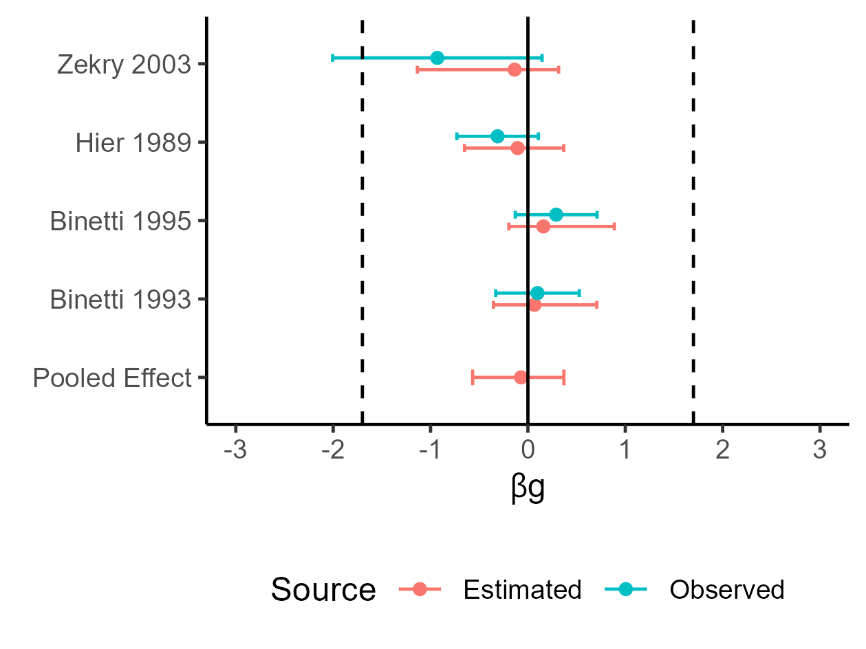


Figure 1. Forest plot for the Clinical Deterioration Scale. Regression coefficients and 95% confidence intervals are displayed. Dashed vertical lines show the lower and upper bound of the Region of Practical Equivalence set at ±1.7 *g*. Estimated effect sizes are regression coefficient estimates and Observed effects are the effect sizes and confidence intervals from the included studies.

## Dementia Rating Scale


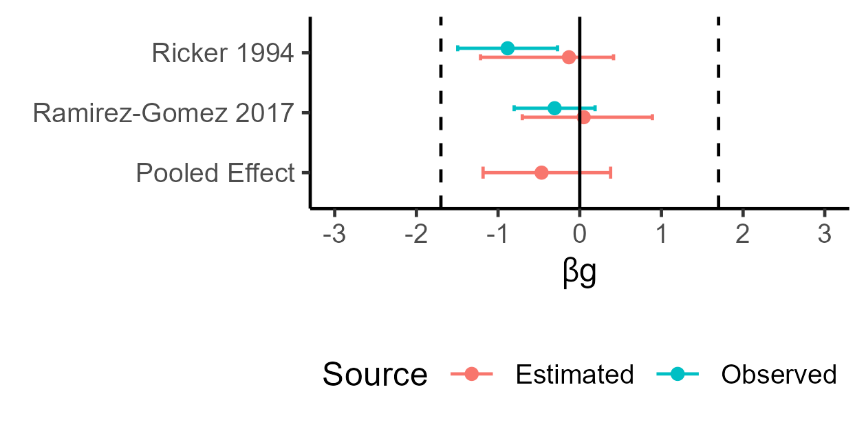


Figure 1. Forest plot for the Dementia Rating Scale. Regression coefficients and 95% confidence intervals are displayed. Dashed vertical lines show the lower and upper bound of the Region of Practical Equivalence set at ±1.7 *g*. Estimated effect sizes are regression coefficient estimates and Observed effects are the effect sizes and confidence intervals from the included studies.

# Orientation to Time and Space


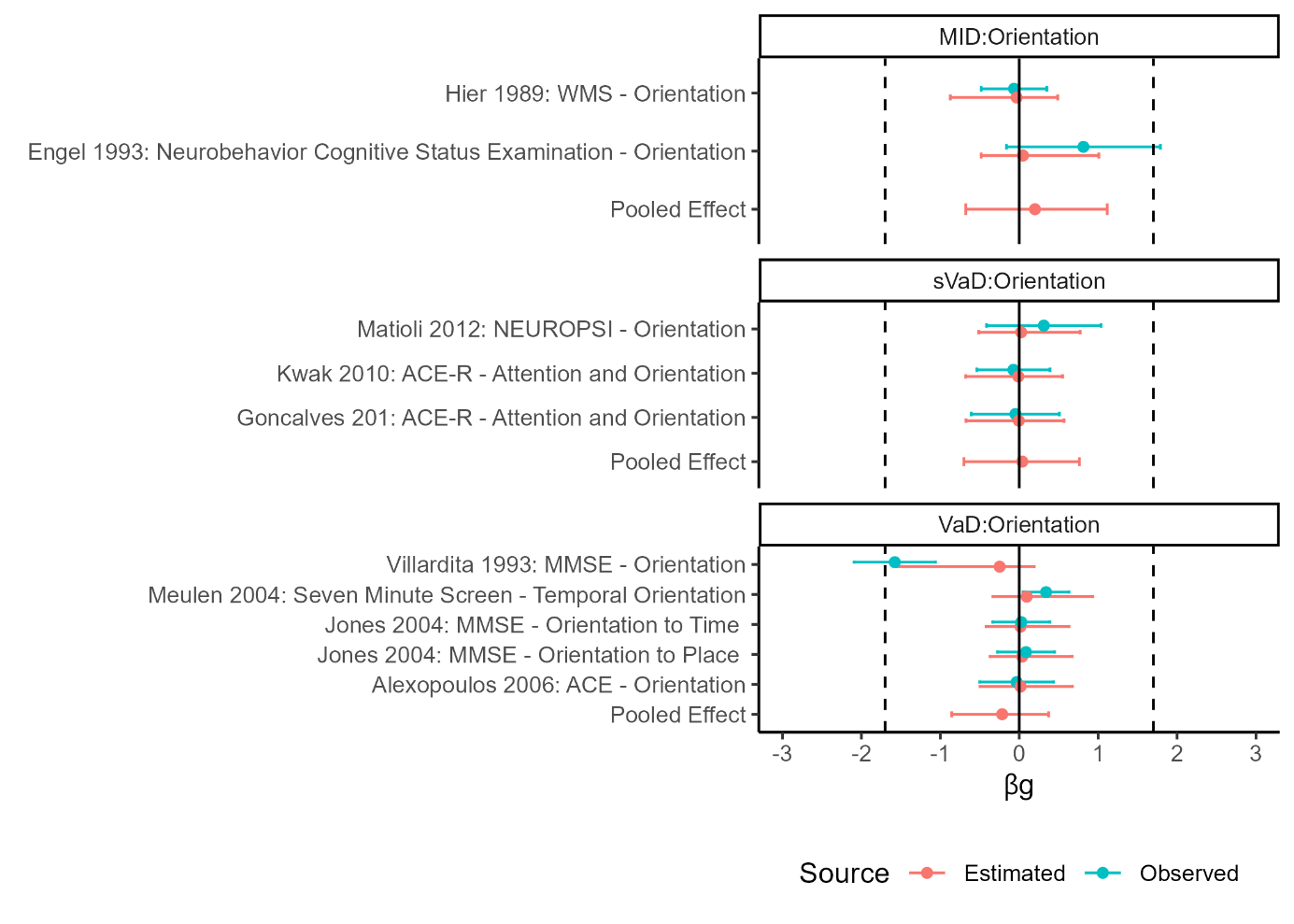


Figure 1. Forest plot for the Orientation to Time and Space measures. Regression coefficients and 95% confidence intervals are displayed. Dashed vertical lines show the lower and upper bound of the Region of Practical Equivalence set at ±1.7 *g*. Estimated effect sizes are regression coefficient estimates and Observed effects are the effect sizes and confidence intervals from the included studies. WMS: Wechsler Memory Scale, MMSE: Mini-Mental-State-Examination, ACE: Addenbrooke’s Cognitive Examination.

# Activities of Daily Living


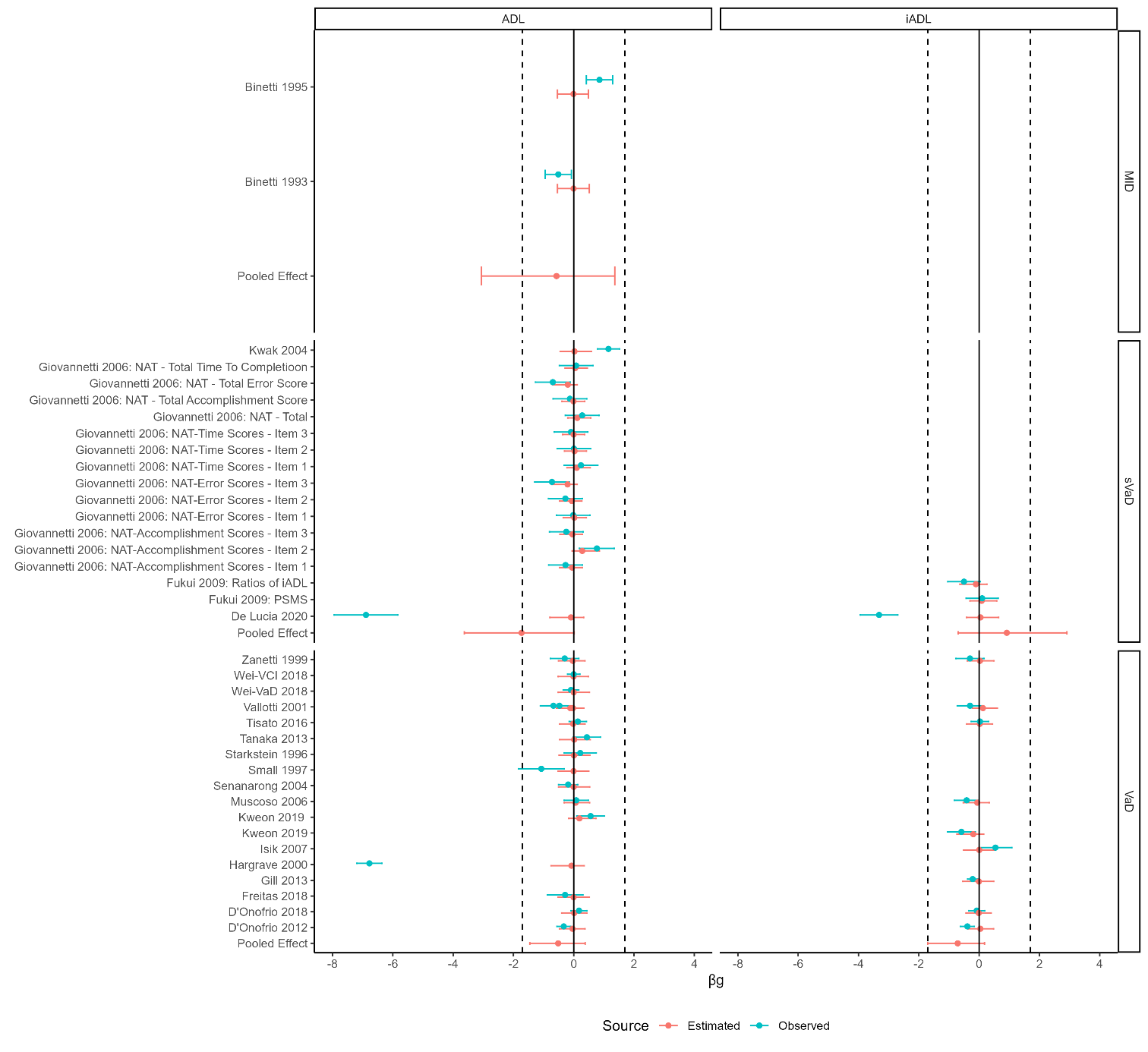


Figure 1. Forest plot for the basic (ADL) and instrumental Activities of Daily Living (iADL). Regression coefficients and 95% confidence intervals are displayed. Dashed vertical lines show the lower and upper bound of the Region of Practical Equivalence set at ±1.7 *g*. Estimated effect sizes are regression coefficient estimates and Observed effects are the effect sizes and confidence intervals from the included studies. NAT: Naturalistic Action Test, PSMS: Physical Self-Maintenance Scale

# Affective Symptoms


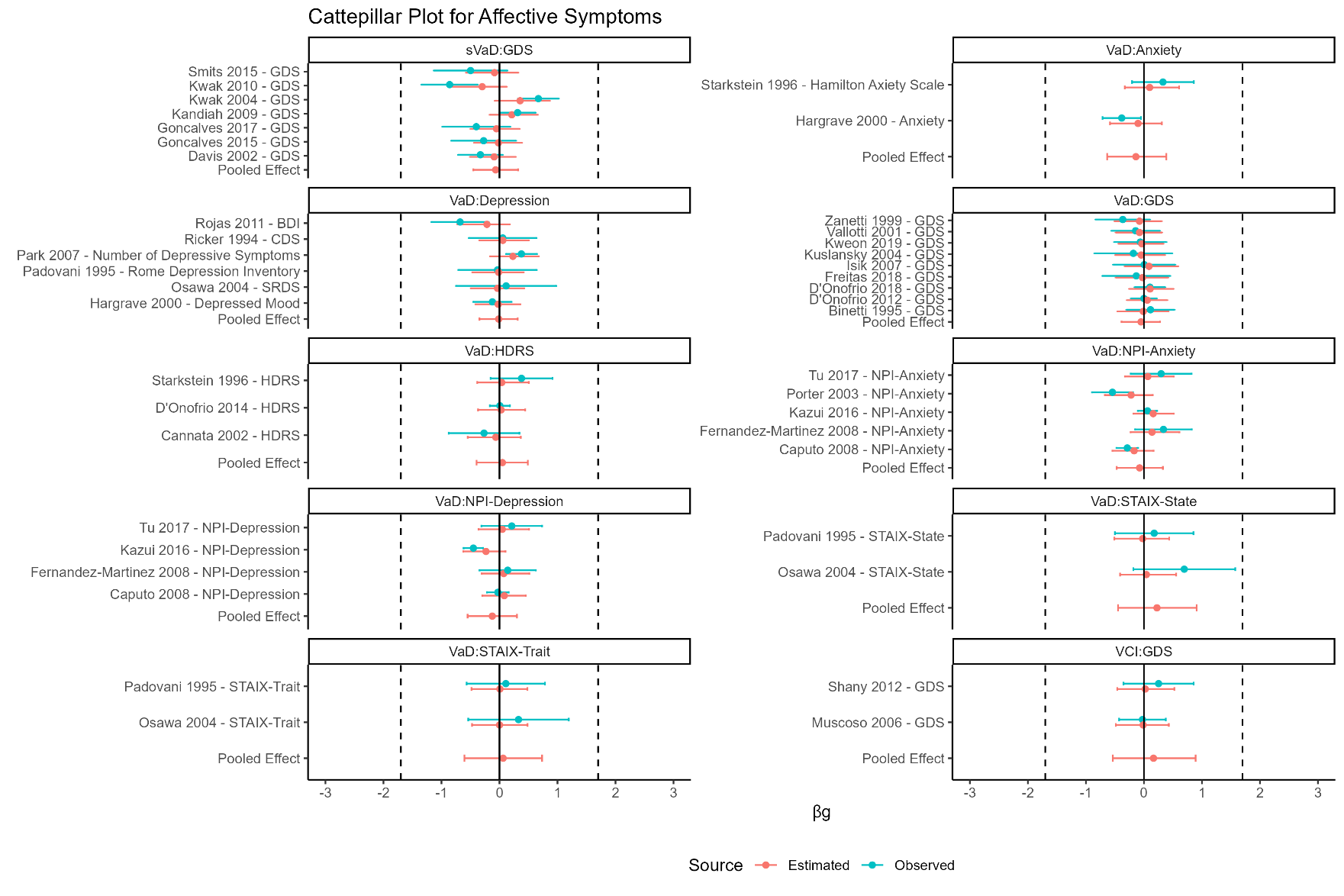


Figure 1. Forest plot for measures of Affective Symptoms. Regression coefficients and 95% confidence intervals are displayed. Dashed vertical lines show the lower and upper bound of the Region of Practical Equivalence set at ±1.7 *g*. Estimated effect sizes are regression coefficient estimates and Observed effects are the effect sizes and confidence intervals from the included studies. BDI: Beck’s Depression Inventory, CDS: Cornell Scale for Depression in Dementia, GDS: Geriatric Depression Scale, HDRS: Hamilton Depression Rating Scale, NPI: Neuropsychiatric Inventory, SRDS: Self-Rating Depression Scale, STAIX: State-Trait Anxiety Inventory.

## Affective Symptoms: Quality Sensitivity Analysis


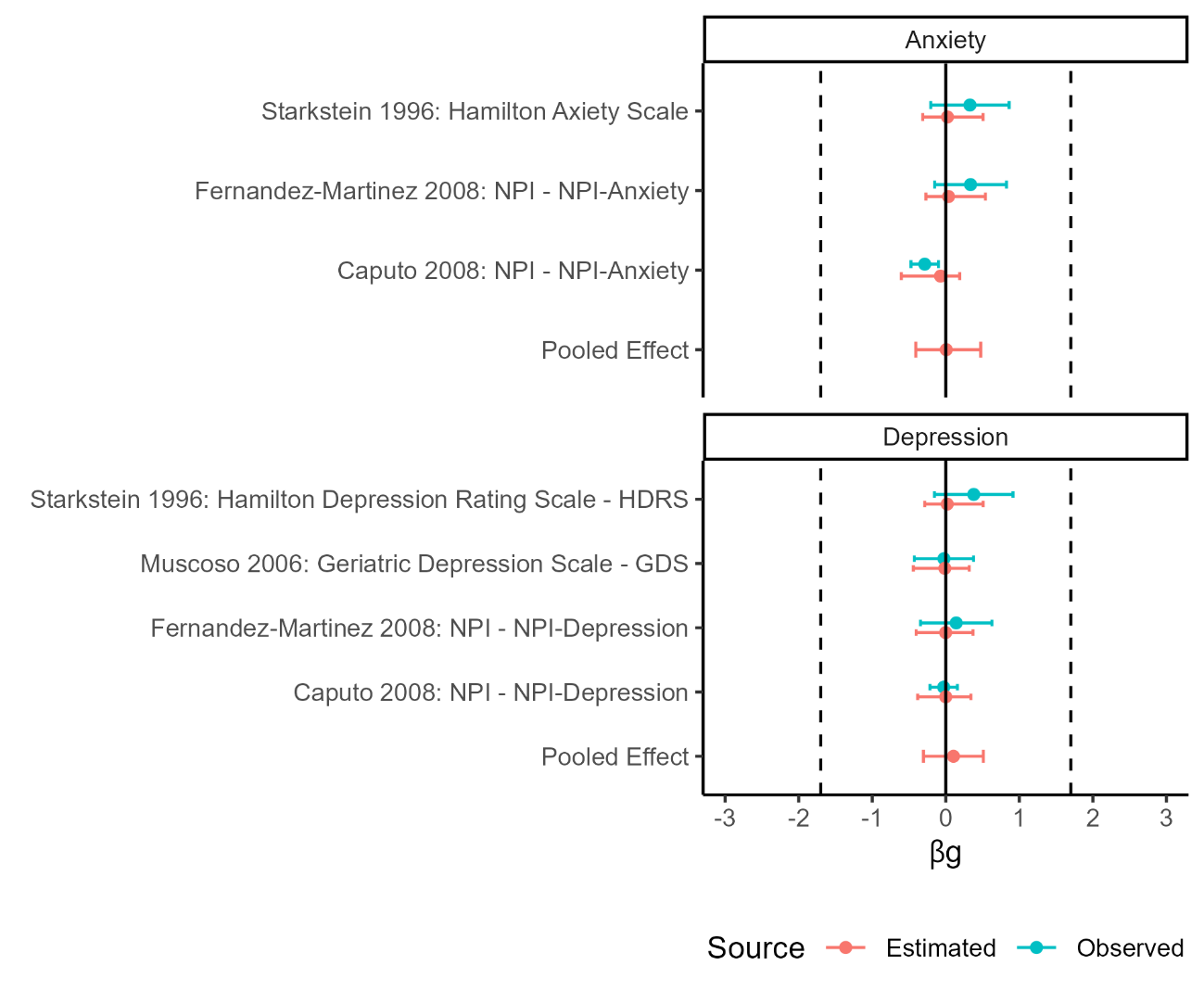


**Figure 1.** Forest plot for study quality sensitivity analysis for measures of Affective Symptoms. Effect sizes and 95% confidence intervals are displayed. Dashed vertical lines show the lower and upper bound of the Region of Practical Equivalence set at ±1.7 *g*. Estimated effect sizes are regression coefficient estimates and Observed effects are the effect sizes and confidence intervals from the included studies. HDRS: Hamilton Depression Rating Scale, NPI: Neuropsychiatric Inventory.

# Disease Awareness


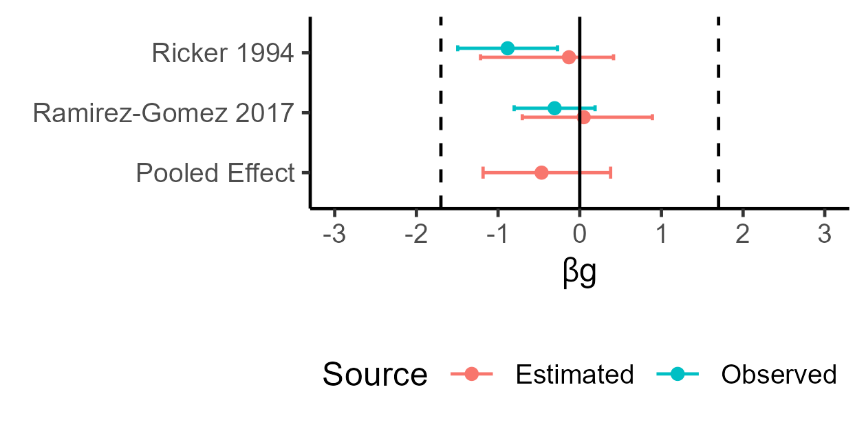


Figure 1. Forest plot for Disease Awareness. Regression coefficients and 95% confidence intervals are displayed. Dashed vertical lines show the lower and upper bound of the Region of Practical Equivalence set at ±1.7 *g*. Estimated effect sizes are regression coefficient estimates and Observed effects are the effect sizes and confidence intervals from the included studies.

# Neuropsychiatric Symptoms

## Global Scores


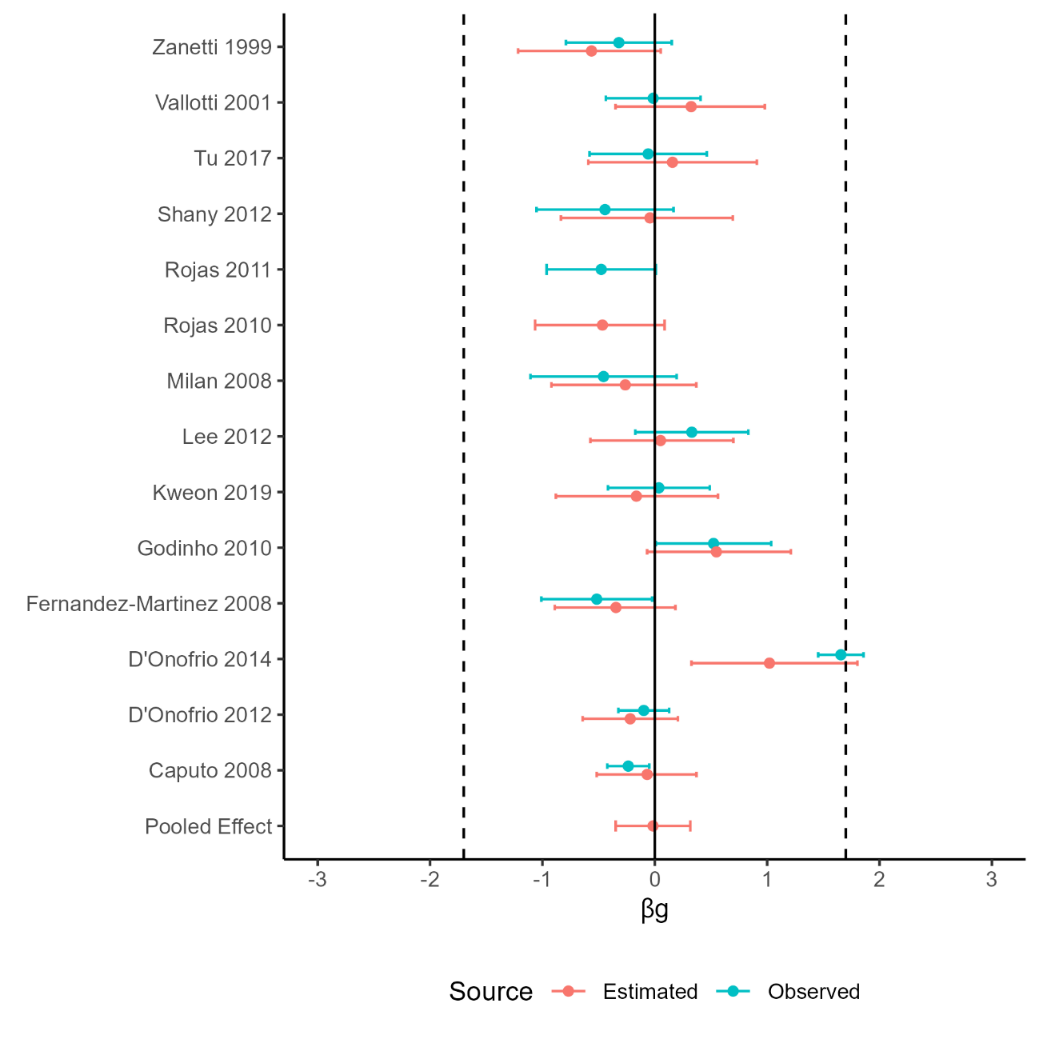


Figure 1. Forest plot for Neuropsychiatric Symptoms Total Scores. Regression coefficients and 95% confidence intervals are displayed. Dashed vertical lines show the lower and upper bound of the Region of Practical Equivalence set at ±1.7 *g*. Estimated effect sizes are regression coefficient estimates and Observed effects are the effect sizes and confidence intervals from the included studies.

### Global Scores: Quality Sensitivity Analysis


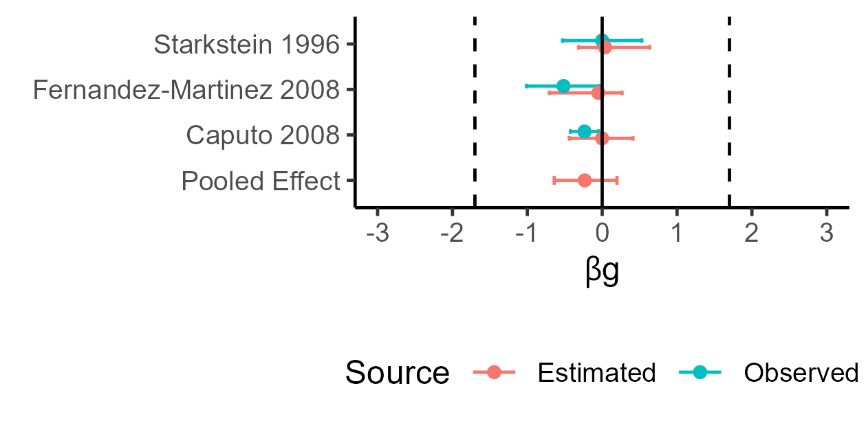


Figure 1. Forest plot for the study quality sensitivity analysis for Neuropsychiatric Symptoms Total Scores. Regression coefficients and 95% confidence intervals are displayed. Dashed vertical lines show the lower and upper bound of the Region of Practical Equivalence set at ±1.7 *g*. Estimated effect sizes are regression coefficient estimates and Observed effects are the effect sizes and confidence intervals from the included studies.

## Domains


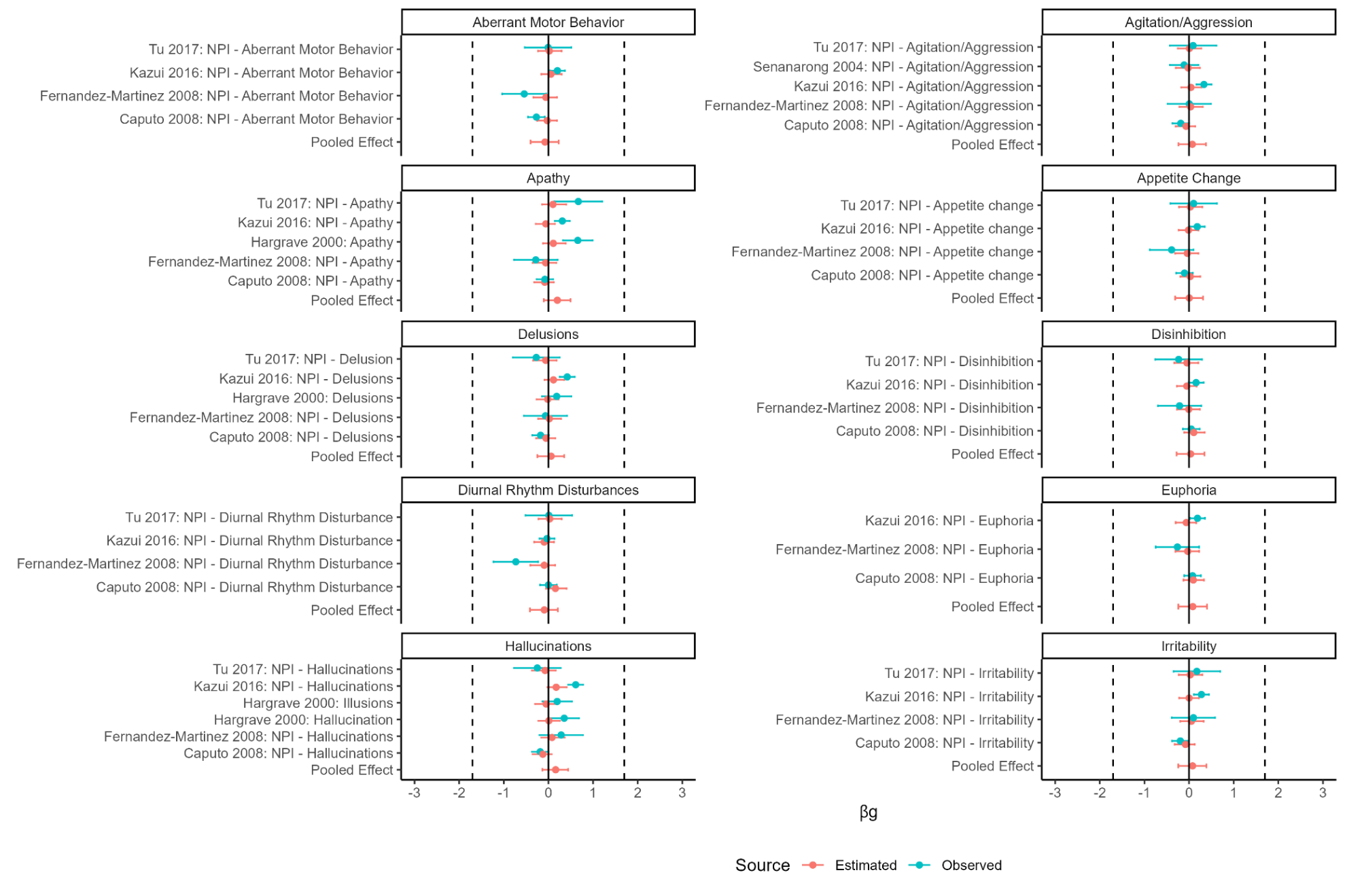


Figure 1. Forest plot for Neuropsychiatric Symptoms: Domain Scores. Regression coefficients and 95% confidence intervals are displayed. Dashed vertical lines show the lower and upper bound of the Region of Practical Equivalence set at ±1.7 *g*. Estimated effect sizes are regression coefficient estimates and Observed effects are the effect sizes and confidence intervals from the included studies. NPI: Neuropsychiatric Inventory.

### Domains: Quality Sensitivity Analysis


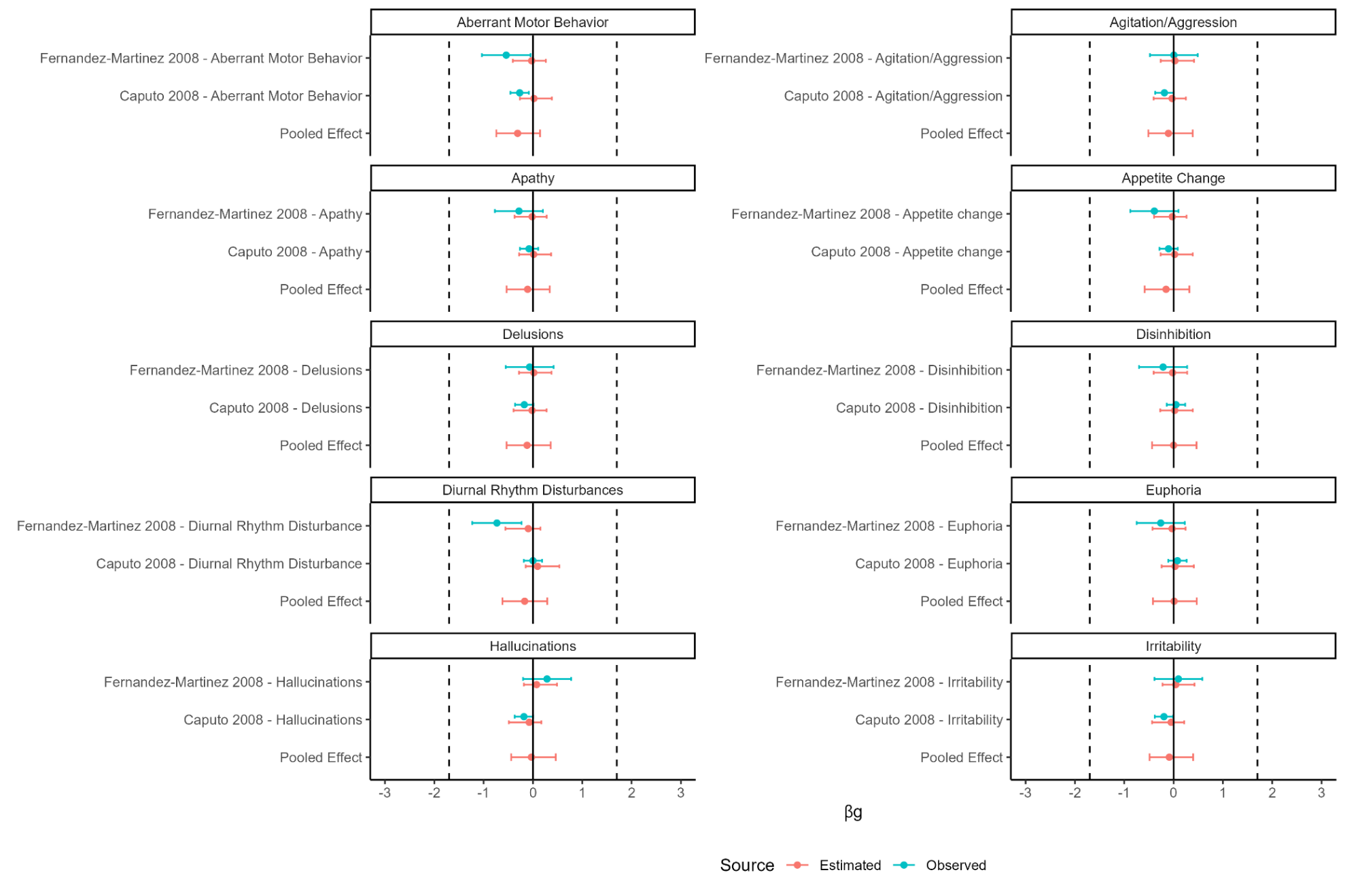


Figure 1. Forest plot of the study quality sensitivity analysis for Neuropsychiatric Symptoms: Domain Scores. Regression coefficients and 95% confidence intervals are displayed. Dashed vertical lines show the lower and upper bound of the Region of Practical Equivalence set at ±1.7 *g*. Estimated effect sizes are regression coefficient estimates and Observed effects are the effect sizes and confidence intervals from the included studies.

# Apraxia

## Apraxia


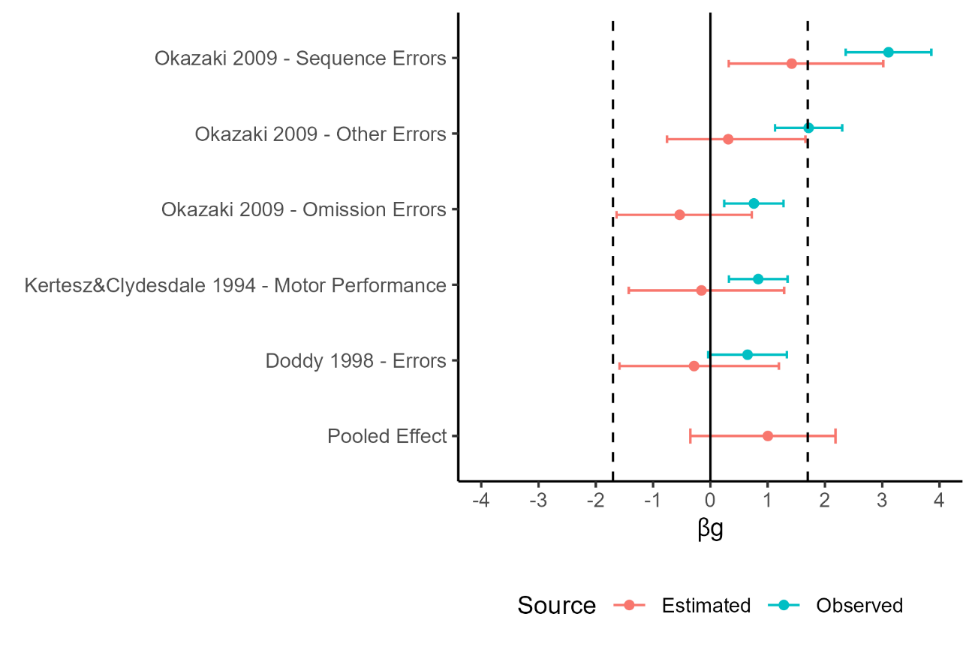


Figure 1. Forest plot for Apraxia measures. Regression coefficients and 95% confidence intervals are displayed. Dashed vertical lines show the lower and upper bound of the Region of Practical Equivalence set at ±1.7 *g*. Estimated effect sizes are regression coefficient estimates and Observed effects are the effect sizes and confidence intervals from the included studies.

## Facial Apraxia


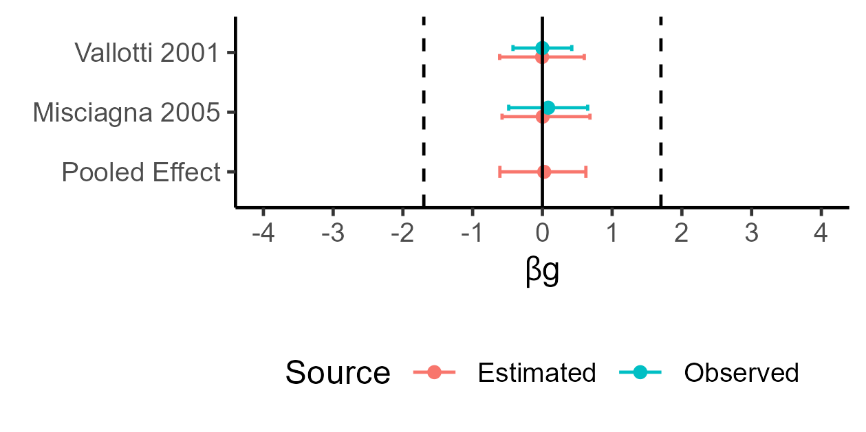


Figure 1. Forest plot for Facial Apraxia measures. Regression coefficients and 95% confidence intervals are displayed. Dashed vertical lines show the lower and upper bound of the Region of Practical Equivalence set at ±1.7 *g*. Estimated effect sizes are regression coefficient estimates and Observed effects are the effect sizes and confidence intervals from the included studies.

## Ideomotor Apraxia


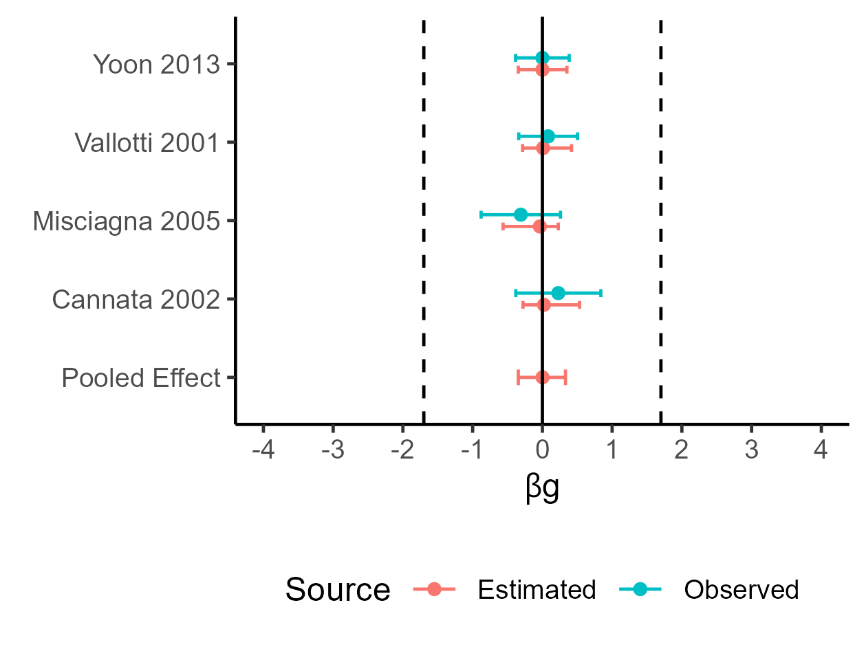


Figure 1. Forest plot for Ideomotor Apraxia measures. Regression coefficients and 95% confidence intervals are displayed. Dashed vertical lines show the lower and upper bound of the Region of Practical Equivalence set at ±1.7 *g*. Estimated effect sizes are regression coefficient estimates and Observed effects are the effect sizes and confidence intervals from the included studies.

# Motor Functioning


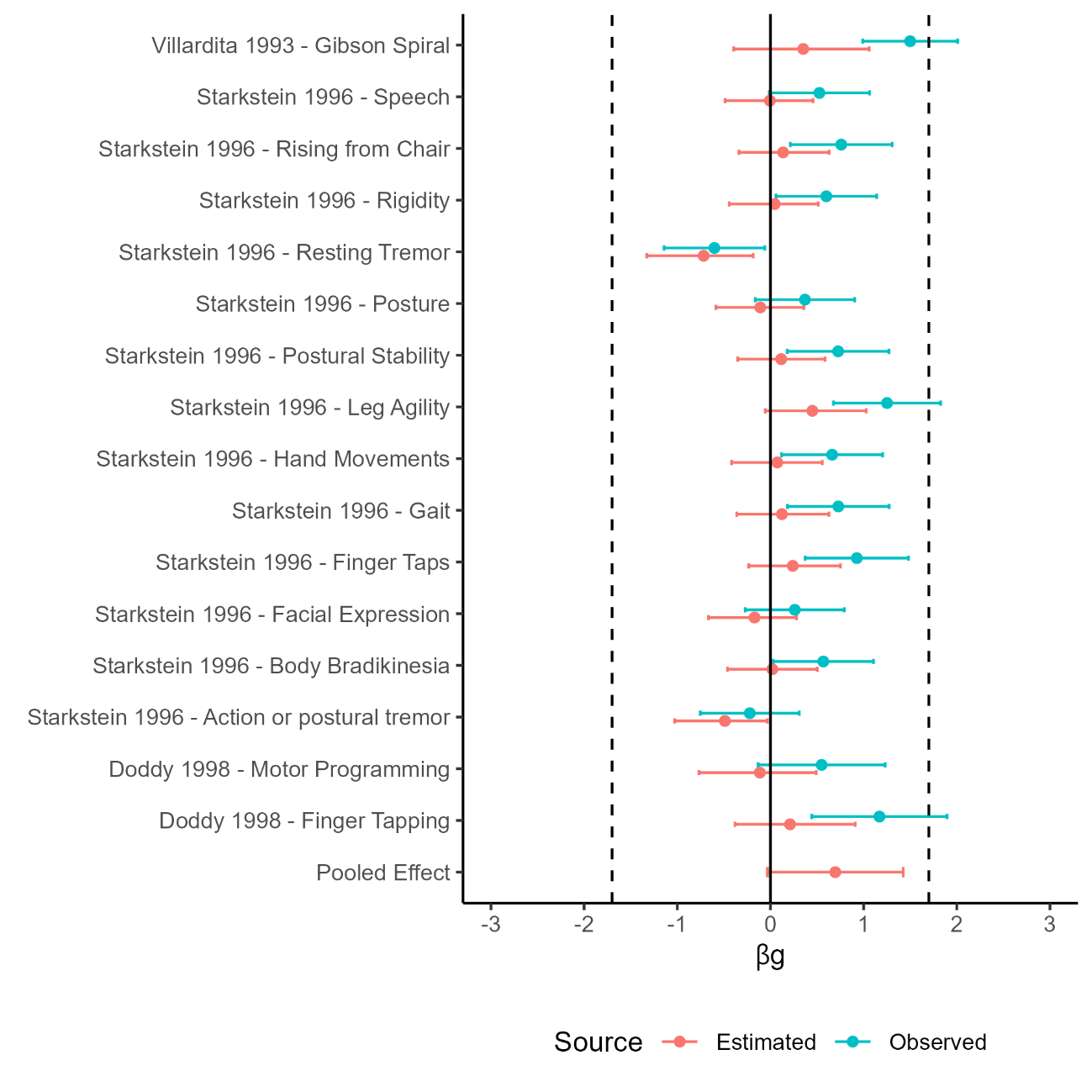


Figure 1. Forest plot for measures of Motor Functioning. Regression coefficients and 95% confidence intervals are displayed. Dashed vertical lines show the lower and upper bound of the Region of Practical Equivalence set at ±1.7 *g*. Estimated effect sizes are regression coefficient estimates and Observed effects are the effect sizes and confidence intervals from the included studies. Starkstein et al. 1996 reported the Unified Parkinson’s Disease Rating Scale subscales.

# Visuo-Spatial Processing

## Wechsler Adult Intelligence Scale


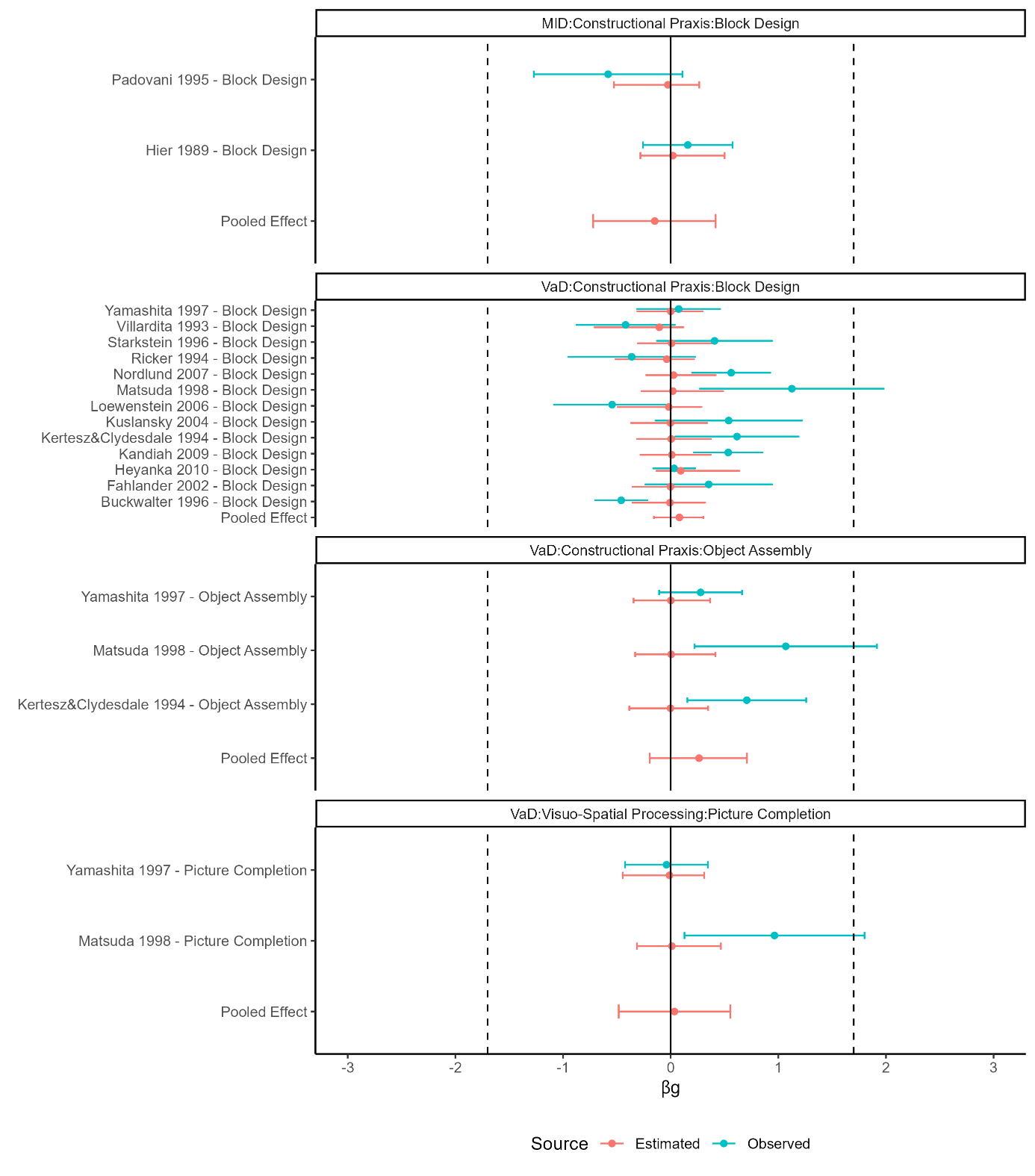


Figure 1. Forest plot for Wechsler Adult Intelligence Scale visuo-spatial processing subtests. Regression coefficients and 95% confidence intervals are displayed. Dashed vertical lines show the lower and upper bound of the Region of Practical Equivalence set at ±1.7 *g*. Estimated effect sizes are regression coefficient estimates and Observed effects are the effect sizes and confidence intervals from the included studies. MID: multi-infarct dementia, VaD: vascular dementia.

## Clock Drawing Test


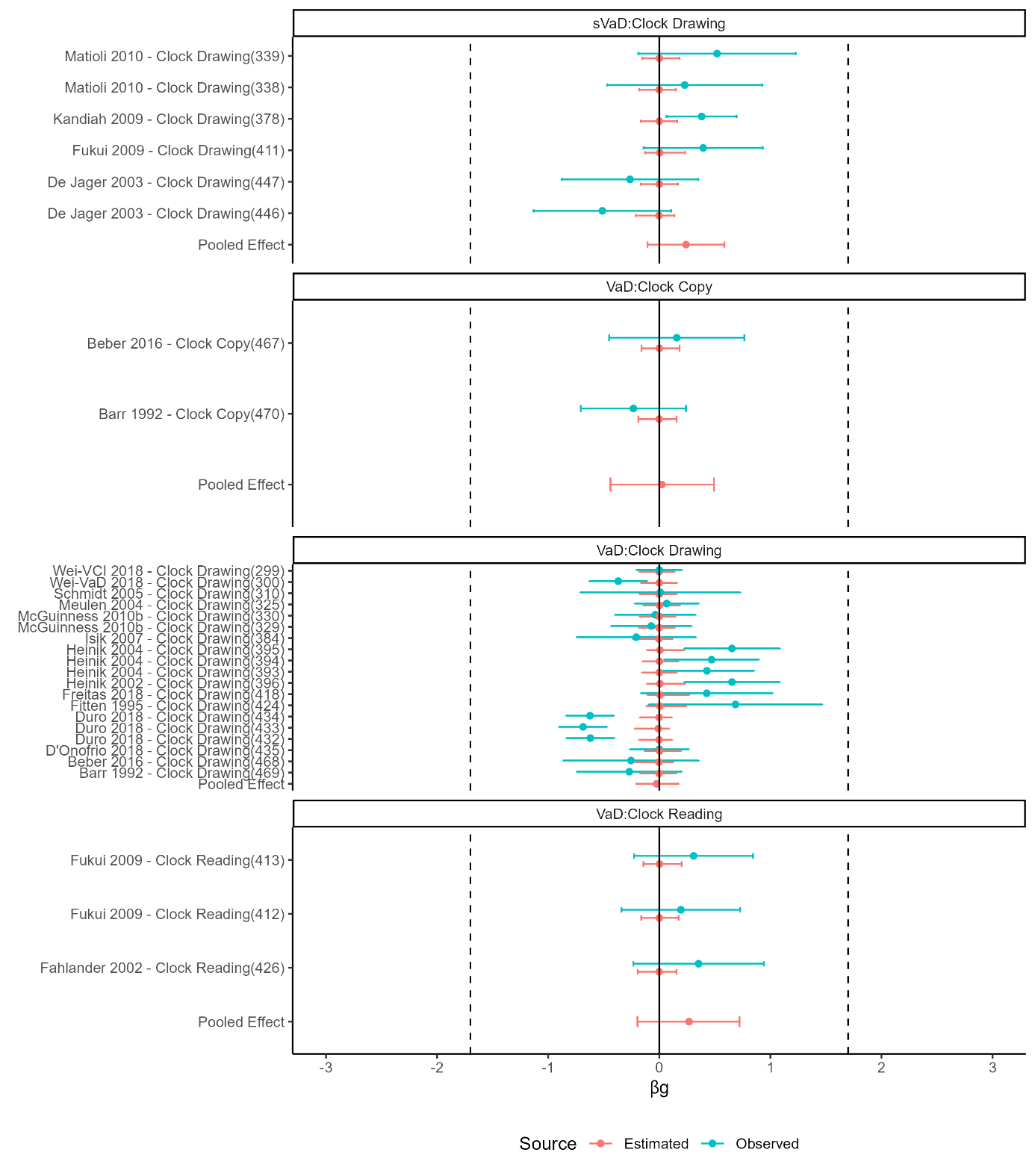


Figure 1. Forest plot for the Clock Drawing Test measures. Regression coefficients and 95% confidence intervals are displayed. Dashed vertical lines show the lower and upper bound of the Region of Practical Equivalence set at ±1.7 *g*. Estimated effect sizes are regression coefficient estimates and Observed effects are the effect sizes and confidence intervals from the included studies. Numbers in parentheses denote different effect sizes reported in the studies. VaD: vascular dementia, sVaD: subcortical vascular dementia.

## Rey-Osterrieth Complex Figure Test


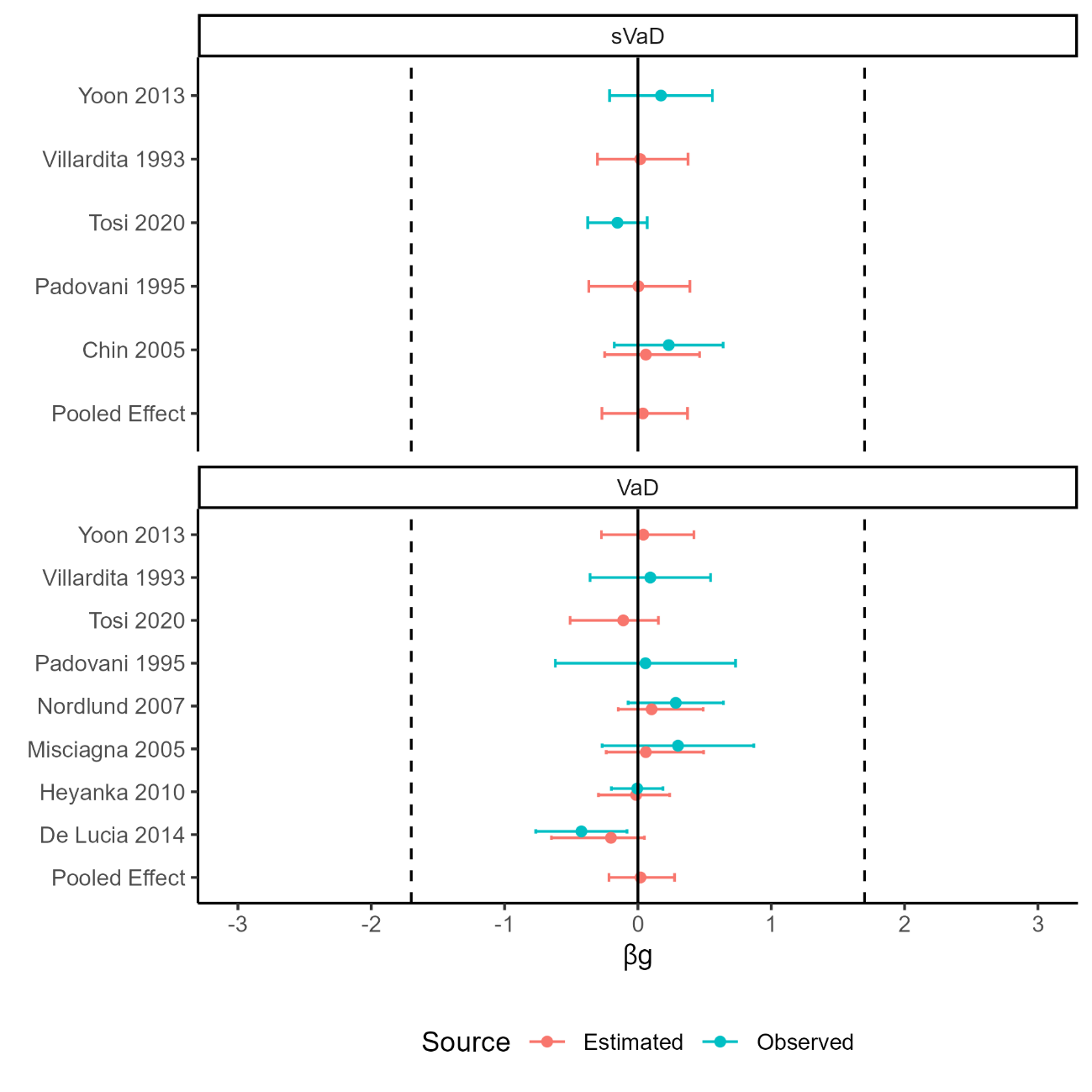


Figure 1. Forest plot for the Rey-Osterrieth Complex Figure Test: Copy. Regression coefficients and 95% confidence intervals are displayed. Dashed vertical lines show the lower and upper bound of the Region of Practical Equivalence set at ±1.7 *g*. Estimated effect sizes are regression coefficient estimates and Observed effects are the effect sizes and confidence intervals from the included studies. VaD: vascular dementia, sVaD: subcortical vascular dementia.

## Visual Object and Space Perception Battery


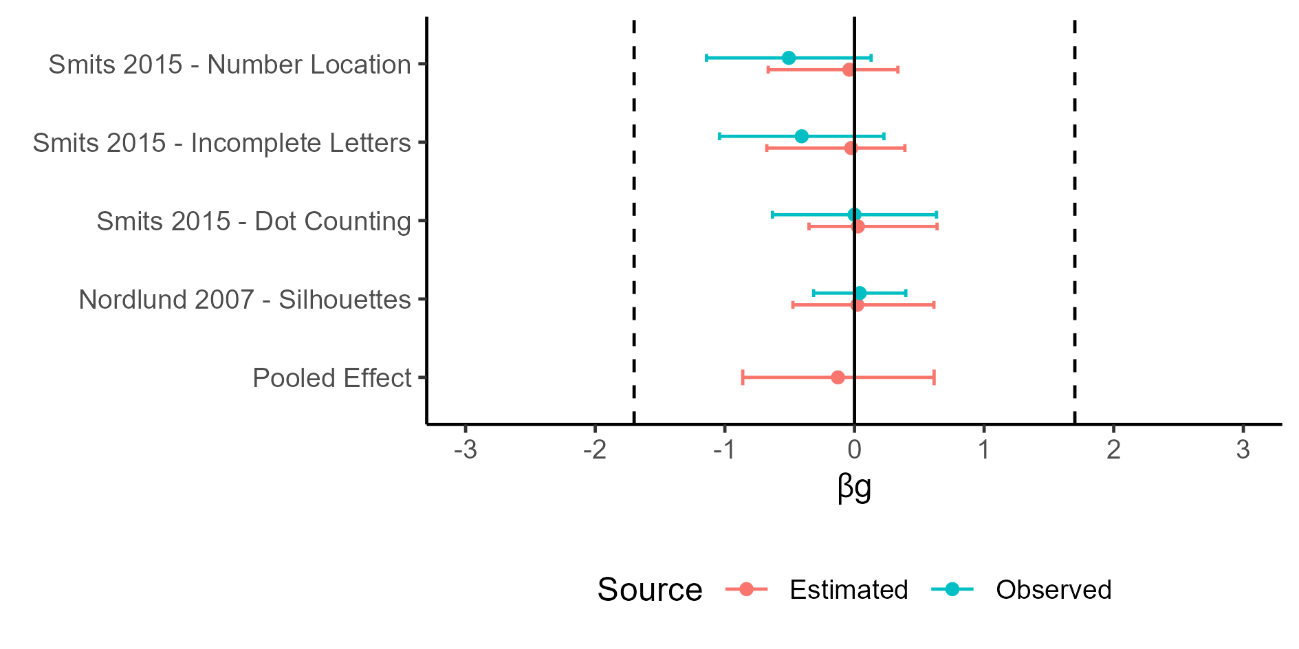


Figure 1. Forest plot for the Visual Object and Space Perception Battery subtests. Regression coefficients and 95% confidence intervals are displayed. Dashed vertical lines show the lower and upper bound of the Region of Practical Equivalence set at ±1.7 *g*. Estimated effect sizes are regression coefficient estimates and Observed effects are the effect sizes and confidence intervals from the included studies.

## CERAD: Constructional Praxis


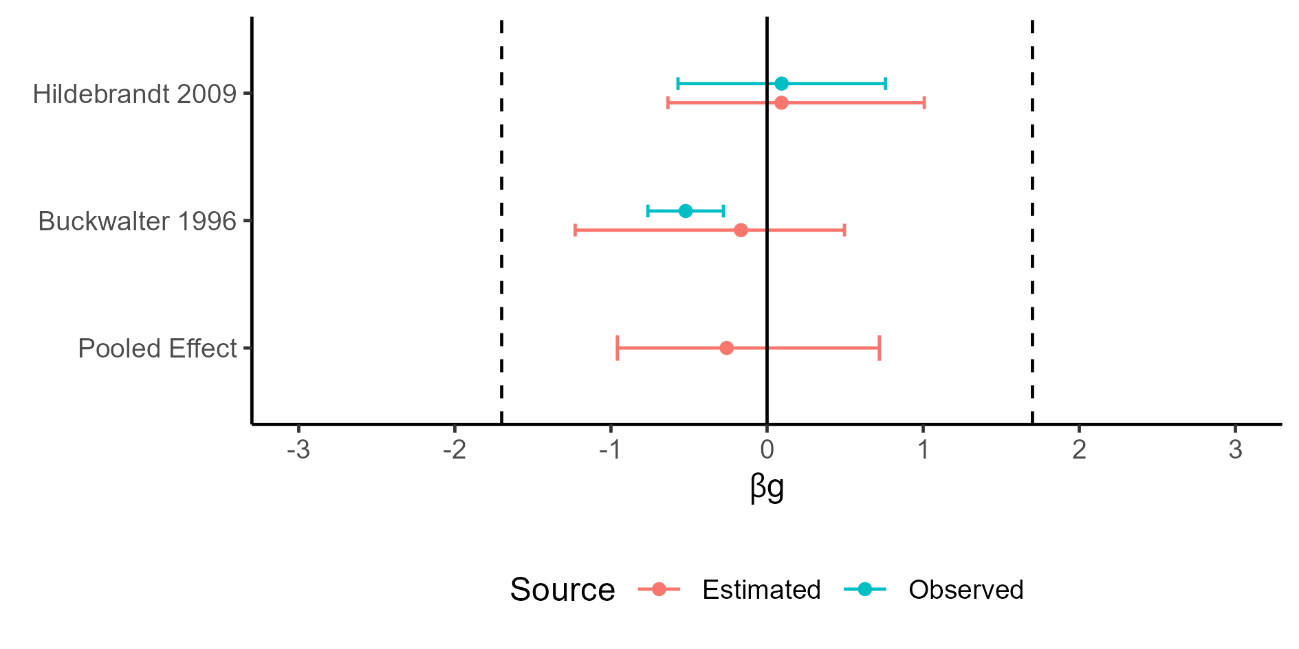


Figure 1. Forest plot for the Consortium to Establish a Registry for Alzheimer’s Disease (CERAD): Constructional Praxis subtest. Regression coefficients and 95% confidence intervals are displayed. Dashed vertical lines show the lower and upper bound of the Region of Practical Equivalence set at ±1.7 *g*. Estimated effect sizes are regression coefficient estimates and Observed effects are the effect sizes and confidence intervals from the included studies.

## Addenbrooke’s Cognitive Examination - R


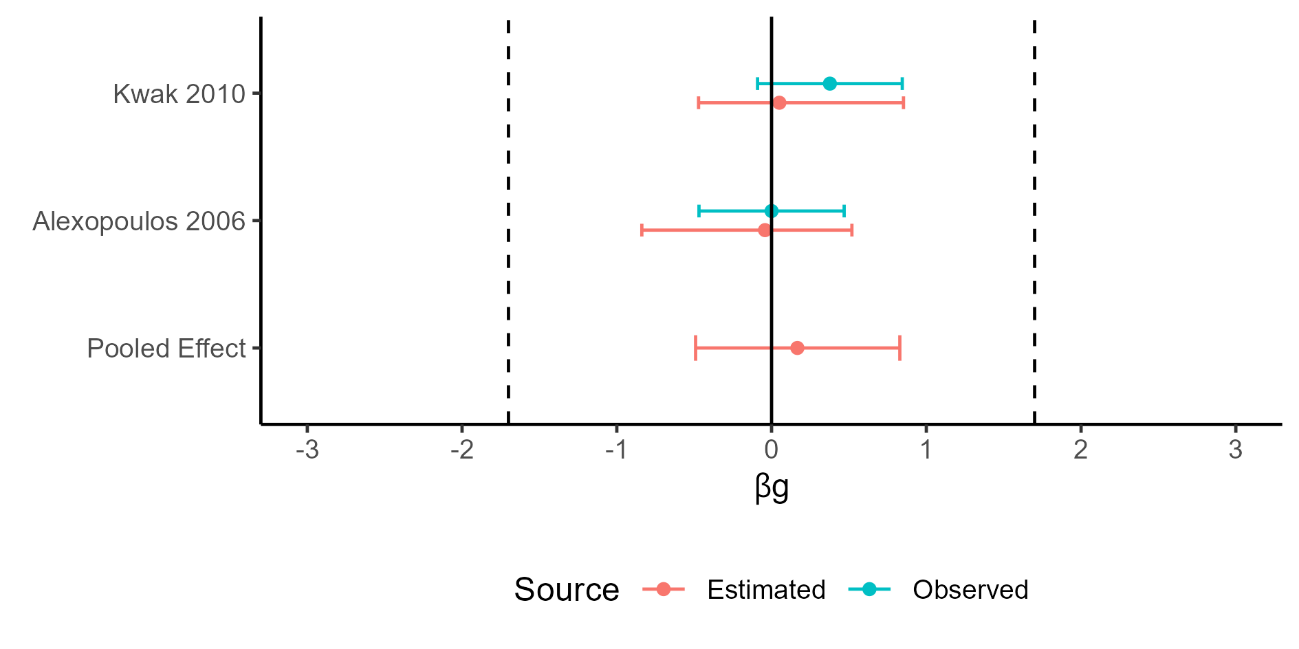


Figure 1. Forest plot for the Addenbrooke’s Cognitive Examination: Visuospatial subscale. Regression coefficients and 95% confidence intervals are displayed. Dashed vertical lines show the lower and upper bound of the Region of Practical Equivalence set at ±1.7 *g*. Estimated effect sizes are regression coefficient estimates and Observed effects are the effect sizes and confidence intervals from the included studies.

## Judgment of Line Orientation


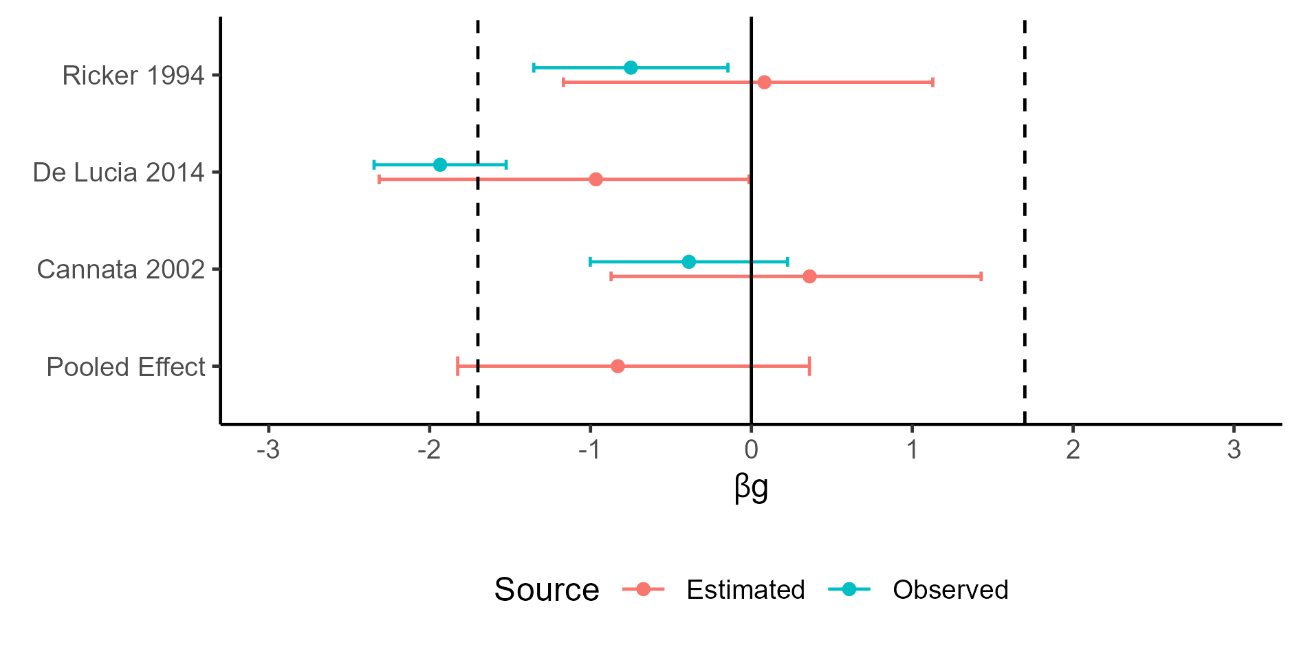


Figure 1. Forest plot for the Judgment of Line Orientation test. Regression coefficients and 95% confidence intervals are displayed. Dashed vertical lines show the lower and upper bound of the Region of Practical Equivalence set at ±1.7 *g*. Estimated effect sizes are regression coefficient estimates and Observed effects are the effect sizes and confidence intervals from the included studies.

## Hooper’s Test


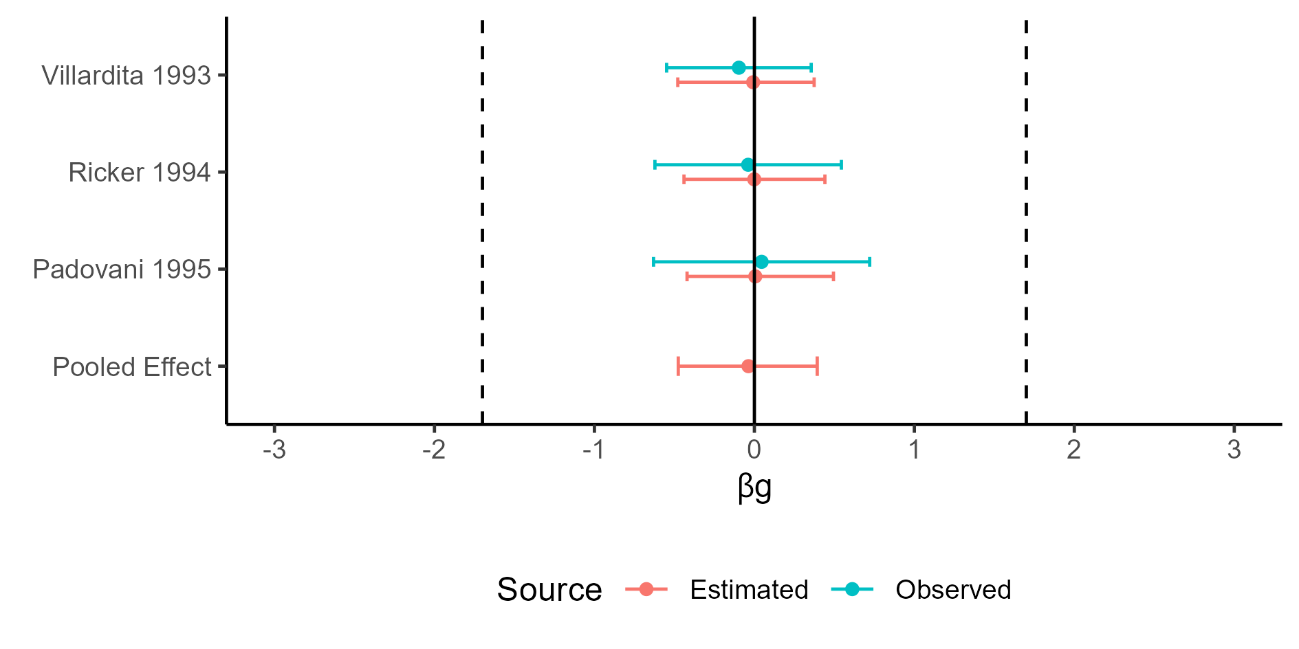


Figure 1. Forest plot for the Hooper’s Visual Organisation Test. Regression coefficients and 95% confidence intervals are displayed. Dashed vertical lines show the lower and upper bound of the Region of Practical Equivalence set at ±1.7 *g*. Estimated effect sizes are regression coefficient estimates and Observed effects are the effect sizes and confidence intervals from the included studies.

## Line Bisection


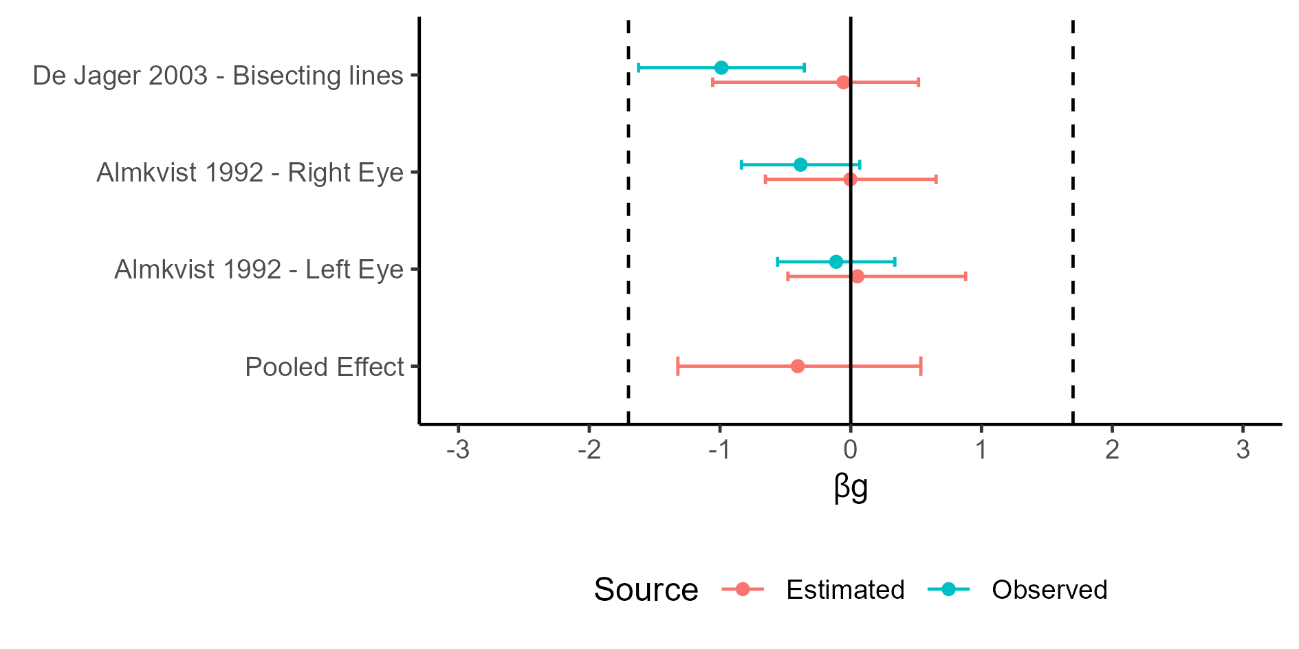


Figure 1. Forest plot for the Line Bisection measures. Regression coefficients and 95% confidence intervals are displayed. Dashed vertical lines show the lower and upper bound of the Region of Practical Equivalence set at ±1.7 *g*. Estimated effect sizes are regression coefficient estimates and Observed effects are the effect sizes and confidence intervals from the included studies.

## Other Copy Measures


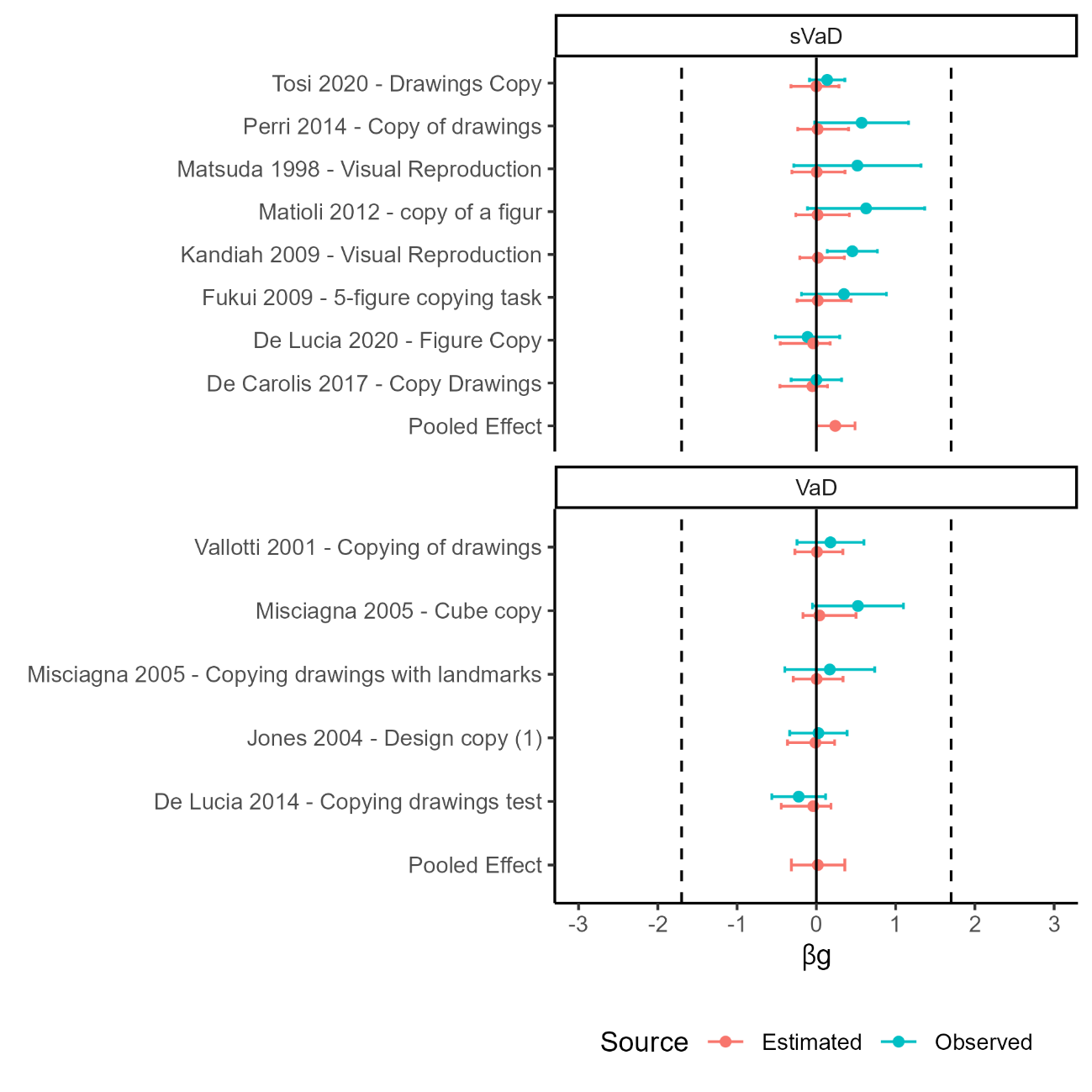


Figure 1. Forest plot for Other Measures of Figure Copy. Regression coefficients and 95% confidence intervals are displayed. Dashed vertical lines show the lower and upper bound of the Region of Practical Equivalence set at ±1.7 *g*. Estimated effect sizes are regression coefficient estimates and Observed effects are the effect sizes and confidence intervals from the included studies. VaD: vascular dementia, sVaD: subcortical vascular dementia.

## Other Construction Measures


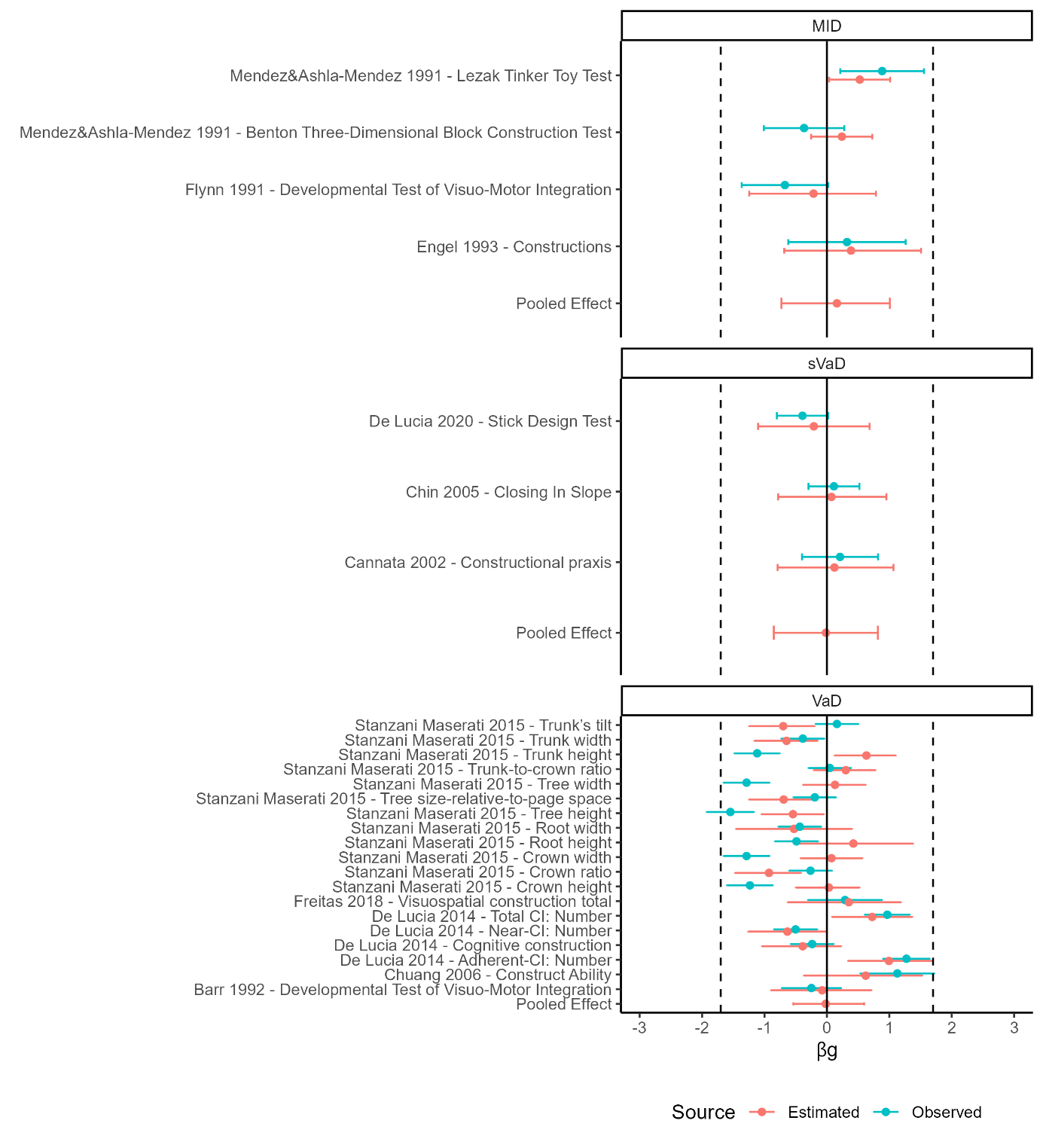


Figure 1. Forest plot for Other Measures of Constructional Praxis. Regression coefficients and 95% confidence intervals are displayed. Dashed vertical lines show the lower and upper bound of the Region of Practical Equivalence set at ±1.7 *g*. Estimated effect sizes are regression coefficient estimates and Observed effects are the effect sizes and confidence intervals from the included studies. MID: multi-infarct dementia, VaD: vascular dementia, sVaD: subcortical vascular dementia.

## Other Measures of Visuo-Spatial Processing


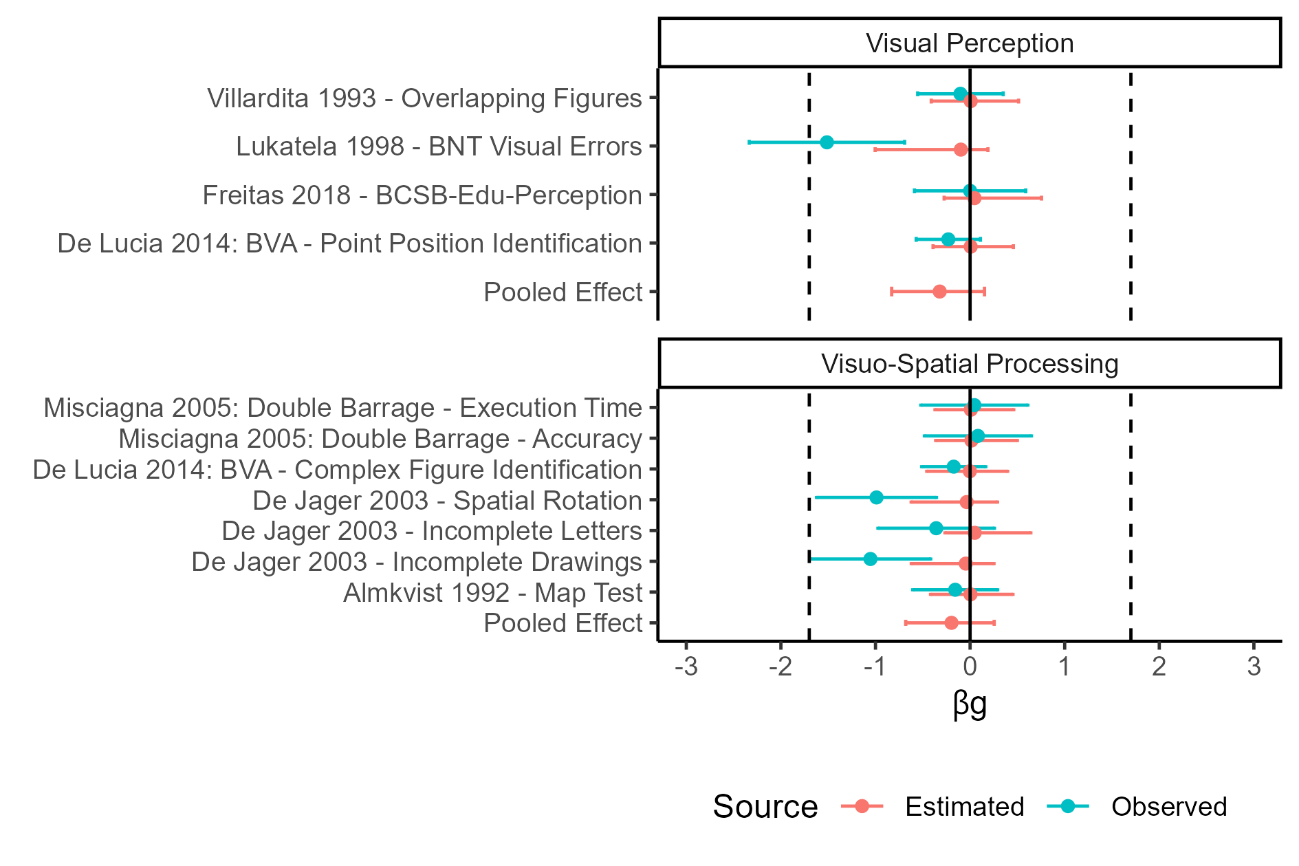


Figure 1. Forest plot for Other Measures of Visuo-Spatial Processing. Regression coefficients and 95% confidence intervals are displayed. Dashed vertical lines show the lower and upper bound of the Region of Practical Equivalence set at ±1.7 *g*. Estimated effect sizes are regression coefficient estimates and Observed effects are the effect sizes and confidence intervals from the included studies. BCSB-Edu: Brief Cognitive Screening Battery – Edu, BVA: Battery for Visuospatial Abilities, BNT: Boston Naming Test.

## Visuo-Spatial Processing: Quality Sensitivity Analysis


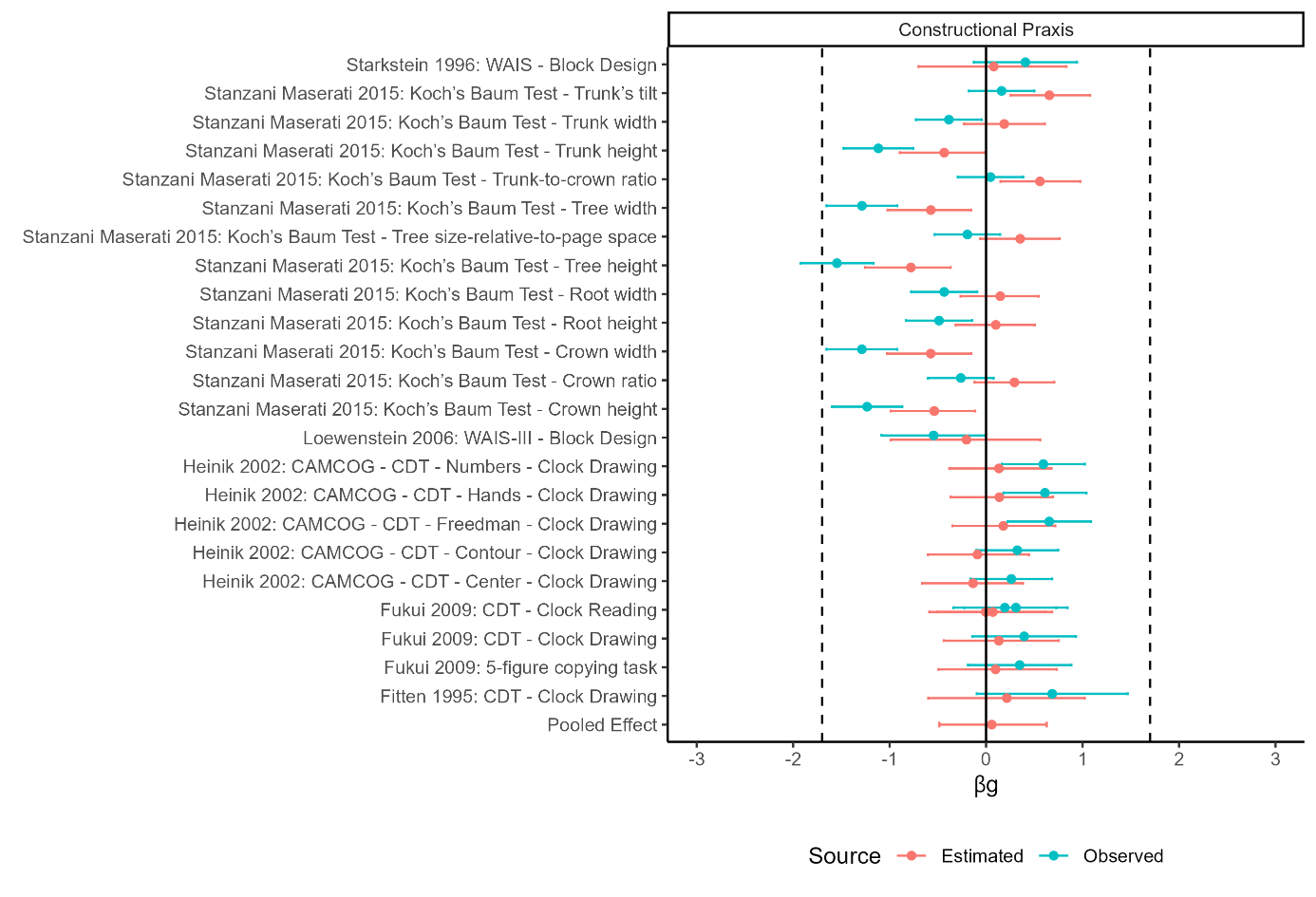


Figure 1. Forest plot of the study quality sensitivity analyses for Measures of Visuo-Spatial Processing. Regression coefficients and 95% confidence intervals are displayed. Dashed vertical lines show the lower and upper bound of the Region of Practical Equivalence set at ±1.7 *g*. Estimated effect sizes are regression coefficient estimates and Observed effects are the effect sizes and confidence intervals from the included studies. WAIS: Wechsler Adult Intelligence Scale, CDT: Clock Drawing Test.

# Intelligence Measures


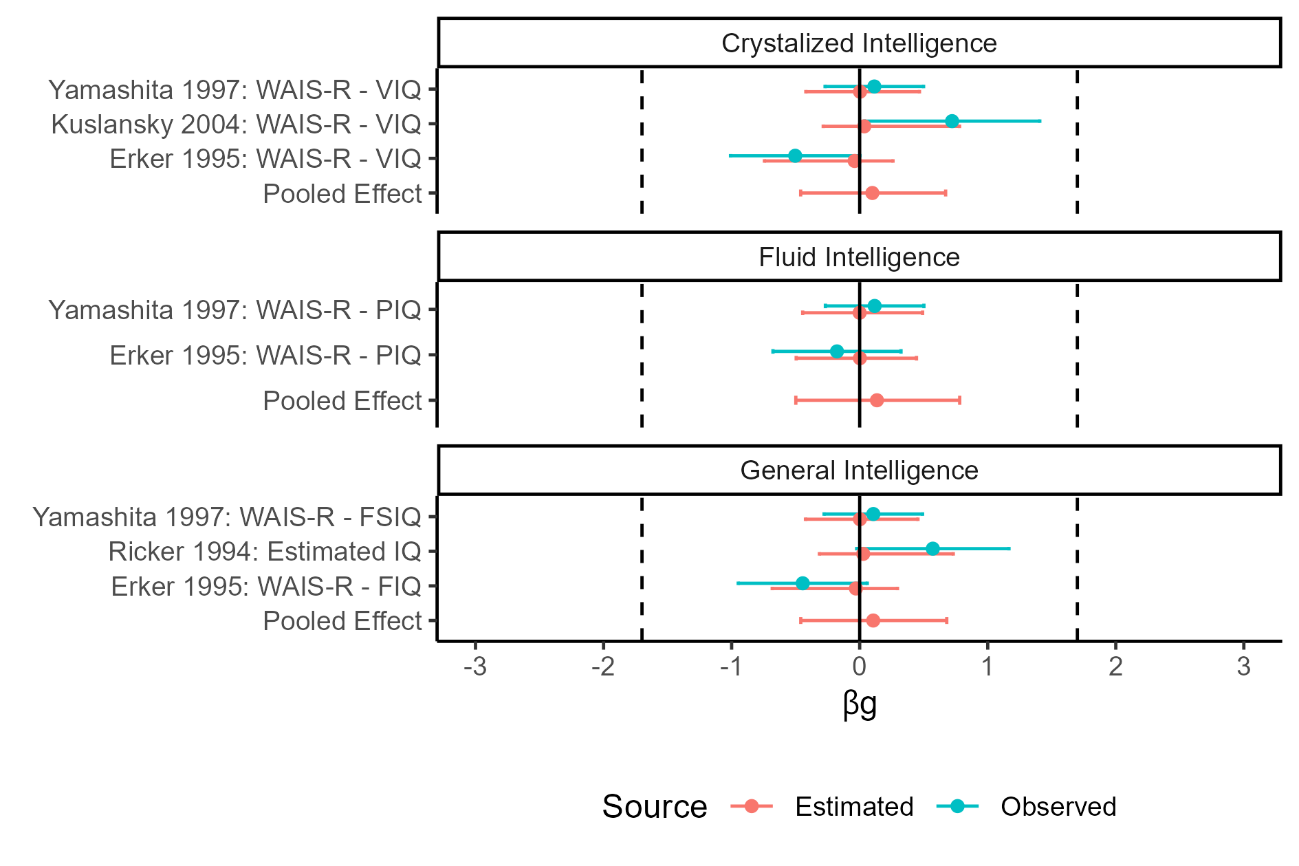


Figure 1. Forest plot for Intelligence Measures. Regression coefficients and 95% confidence intervals are displayed. Dashed vertical lines show the lower and upper bound of the Region of Practical Equivalence set at ±1.7 *g*. Estimated effect sizes are regression coefficient estimates and Observed effects are the effect sizes and confidence intervals from the included studies. WAIS-R: Wechsler Adult Intelligence Scale revised.

# Attention

## Trail Making Test – A


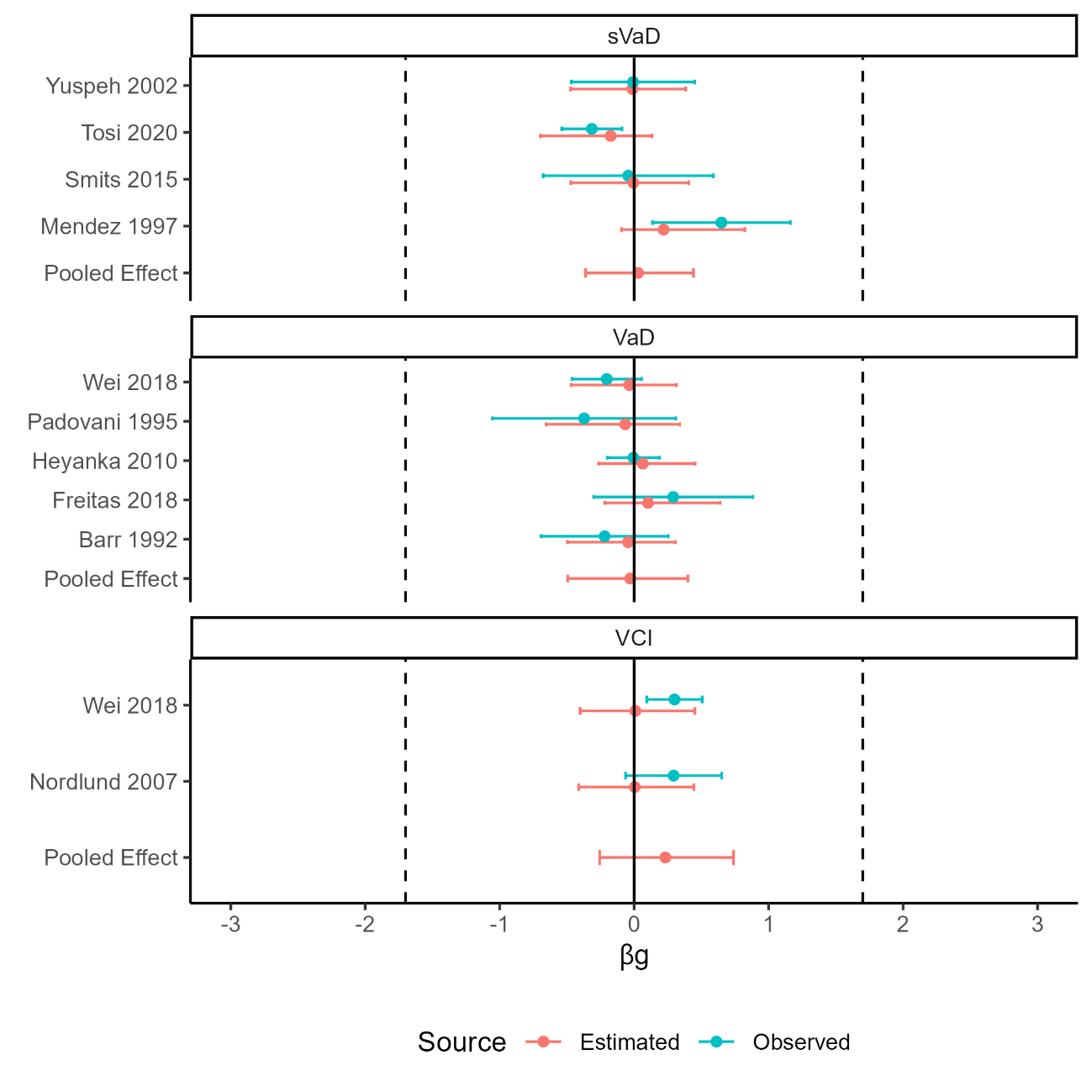


Figure 1. Forest plot for the Trail Making Test: A. Regression coefficients and 95% confidence intervals are displayed. Dashed vertical lines show the lower and upper bound of the Region of Practical Equivalence set at ±1.7 *g*. Estimated effect sizes are regression coefficient estimates and Observed effects are the effect sizes and confidence intervals from the included studies.

## Digit Span Forward


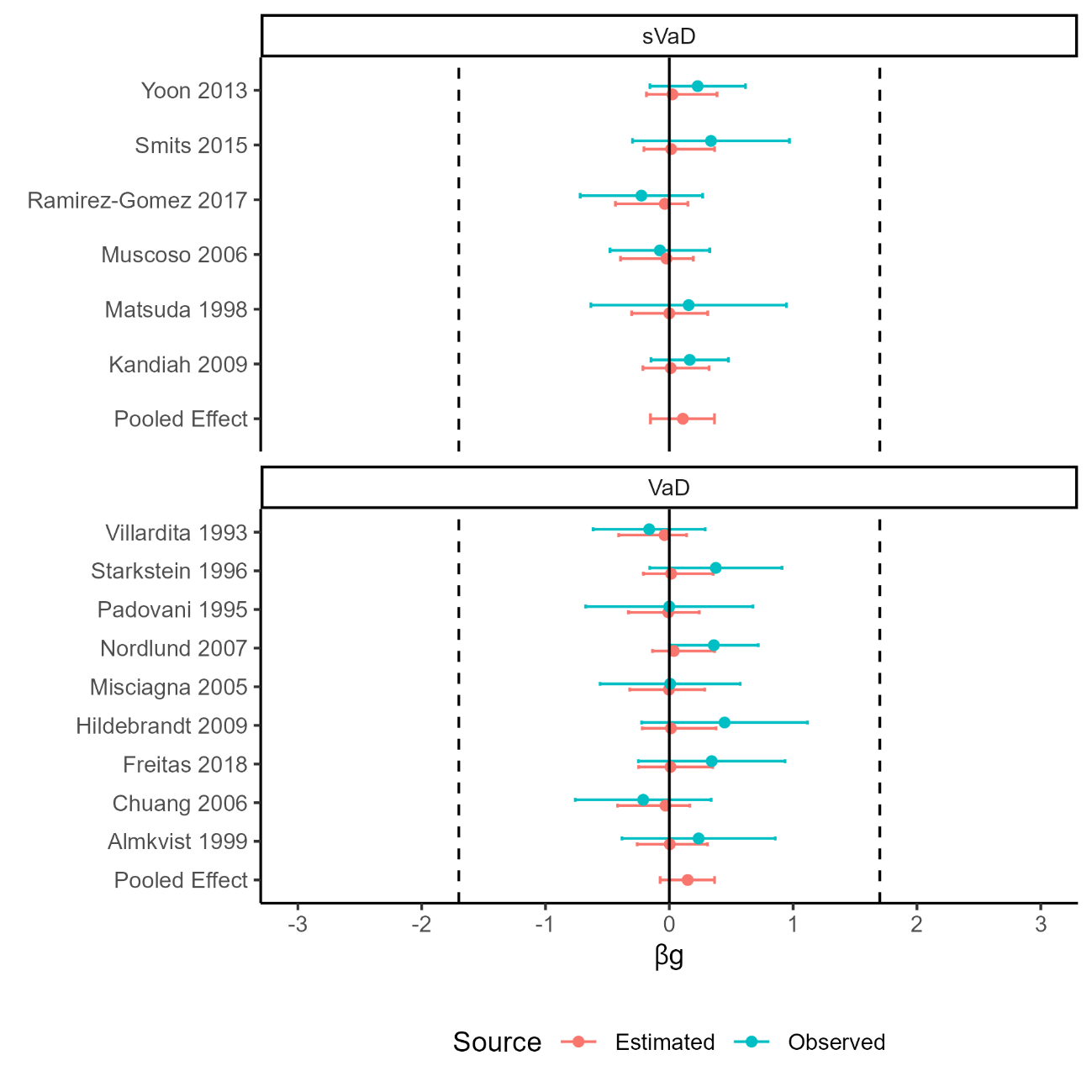


Figure 1. Forest plot for the Digit Span Forward. Regression coefficients and 95% confidence intervals are displayed. Dashed vertical lines show the lower and upper bound of the Region of Practical Equivalence set at ±1.7 *g*. Estimated effect sizes are regression coefficient estimates and Observed effects are the effect sizes and confidence intervals from the included studies.

## Digit Symbol Substitution Test


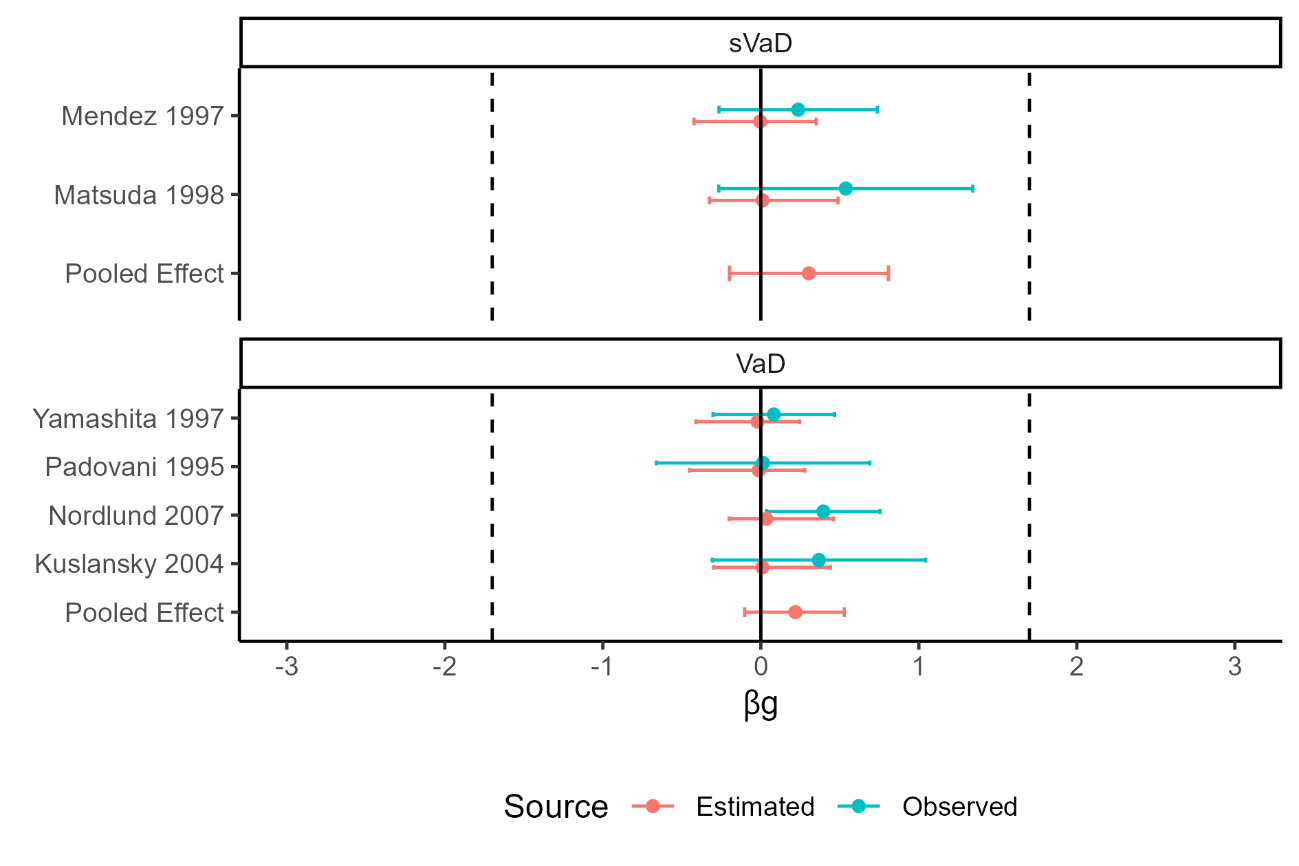


Figure 1. Forest plot for the Digit Symbol Substitution Test. Regression coefficients and 95% confidence intervals are displayed. Dashed vertical lines show the lower and upper bound of the Region of Practical Equivalence set at ±1.7 *g*. Estimated effect sizes are regression coefficient estimates and Observed effects are the effect sizes and confidence intervals from the included studies.

## Symbol Digit Modalities Test


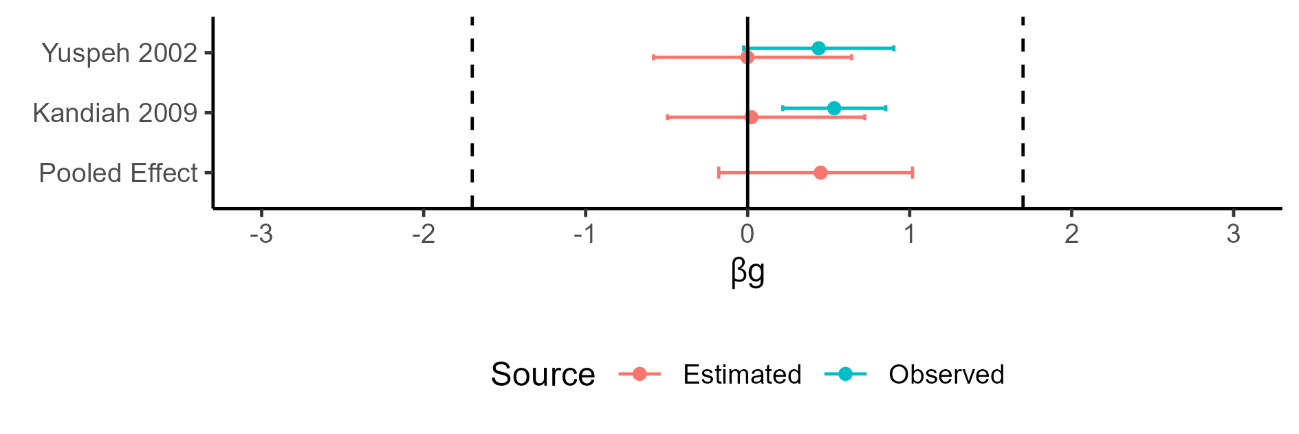


Figure 1. Forest plot for the Symbol Digit Modalities Test. Regression coefficients and 95% confidence intervals are displayed. Dashed vertical lines show the lower and upper bound of the Region of Practical Equivalence set at ±1.7 *g*. Estimated effect sizes are regression coefficient estimates and Observed effects are the effect sizes and confidence intervals from the included studies.

## Choice Reaction Time


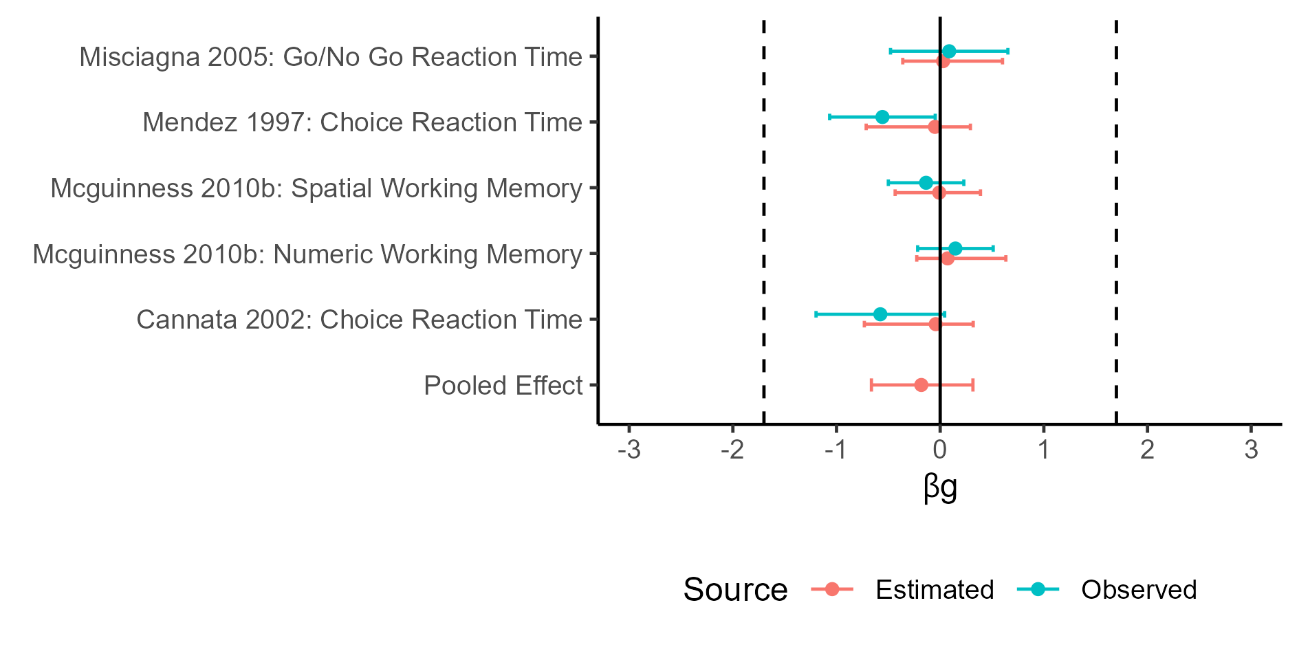


Figure 1. Forest plot for Choice Reaction Time measures. Regression coefficients and 95% confidence intervals are displayed. Dashed vertical lines show the lower and upper bound of the Region of Practical Equivalence set at ±1.7 *g*. Estimated effect sizes are regression coefficient estimates and Observed effects are the effect sizes and confidence intervals from the included studies.

## Other Measures of Selective Attention


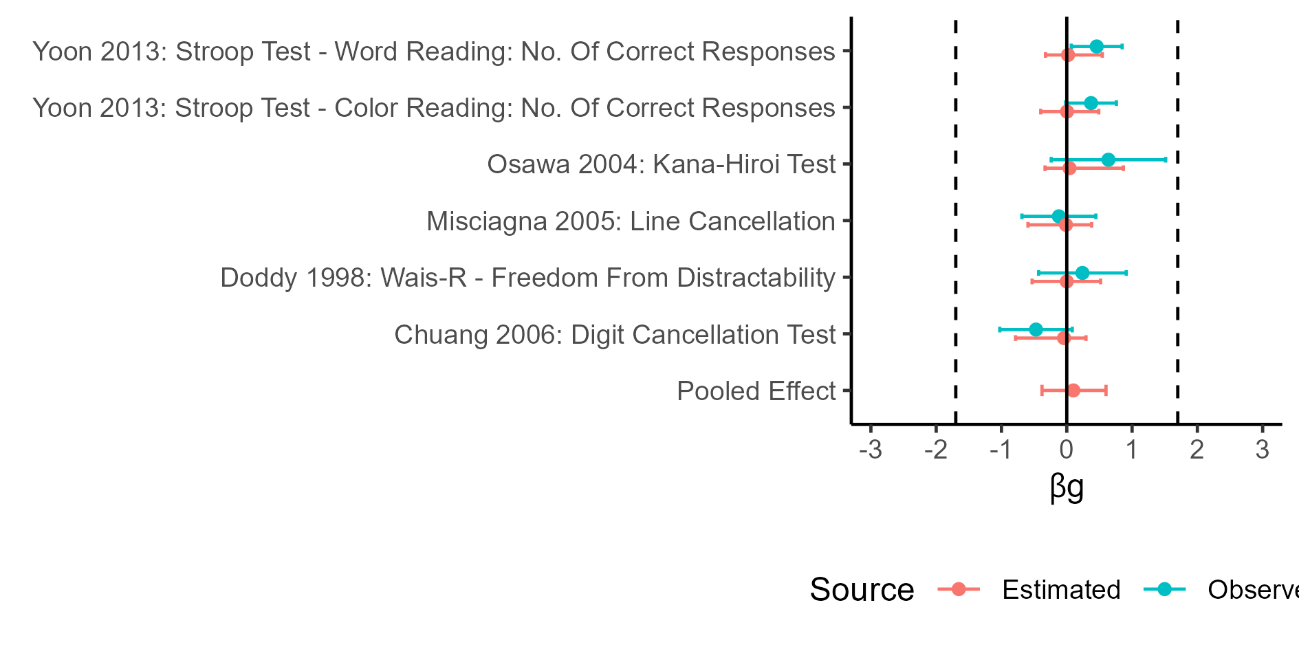


Figure 1. Forest plot for Other Measures of Selective Attention. Regression coefficients and 95% confidence intervals are displayed. Dashed vertical lines show the lower and upper bound of the Region of Practical Equivalence set at ±1.7 *g*. Estimated effect sizes are regression coefficient estimates and Observed effects are the effect sizes and confidence intervals from the included studies.

## Continuous Performance Tests


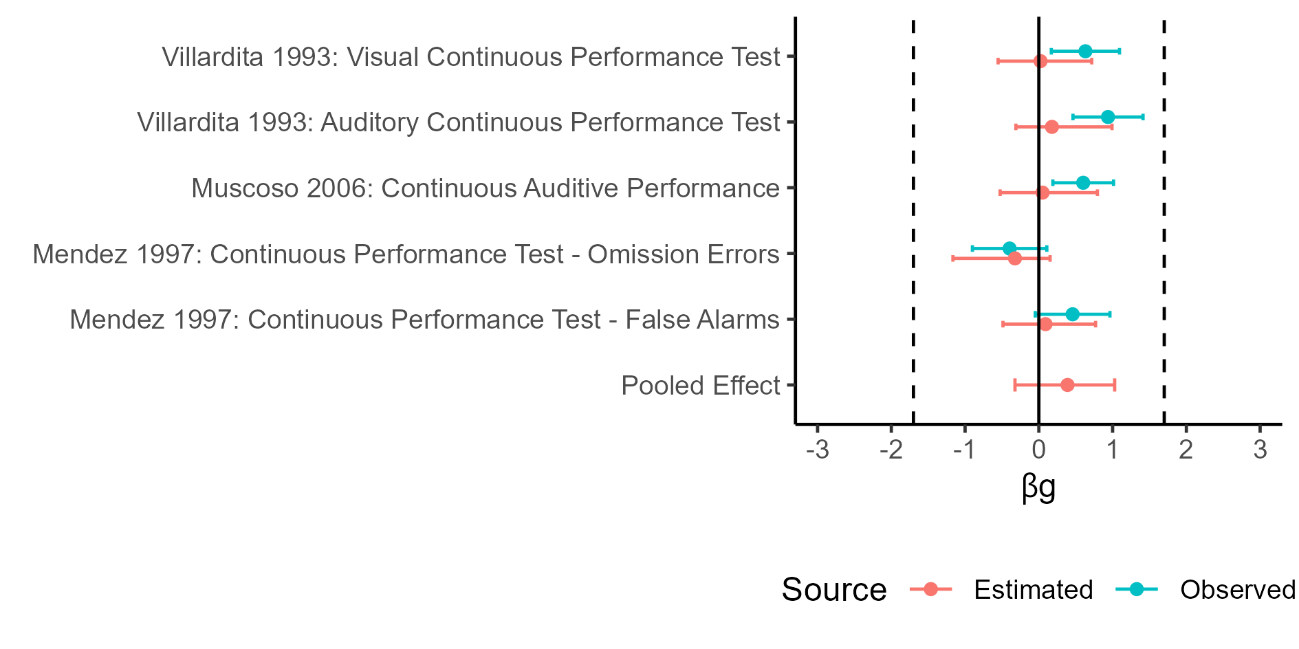


Figure 1. Forest plot for Continuous Performance Tests. Regression coefficients and 95% confidence intervals are displayed. Dashed vertical lines show the lower and upper bound of the Region of Practical Equivalence set at ±1.7 *g*. Estimated effect sizes are regression coefficient estimates and Observed effects are the effect sizes and confidence intervals from the included studies.

## Other Measures of Sustained Attention


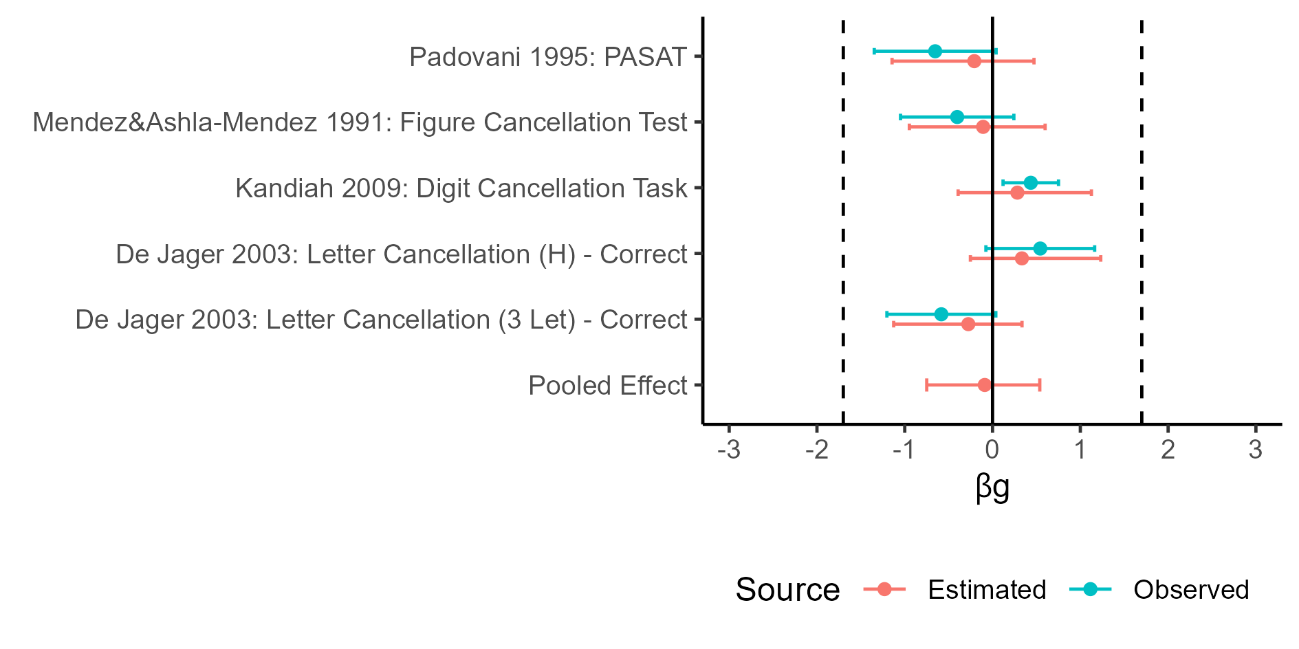


Figure 1. Forest plot for Other Measures of Sustained Attention. Regression coefficients and 95% confidence intervals are displayed. Dashed vertical lines show the lower and upper bound of the Region of Practical Equivalence set at ±1.7 *g*. Estimated effect sizes are regression coefficient estimates and Observed effects are the effect sizes and confidence intervals from the included studies. PASAT: Paced Auditory Serial Addition Test.

## Other Measures of Visual Attention


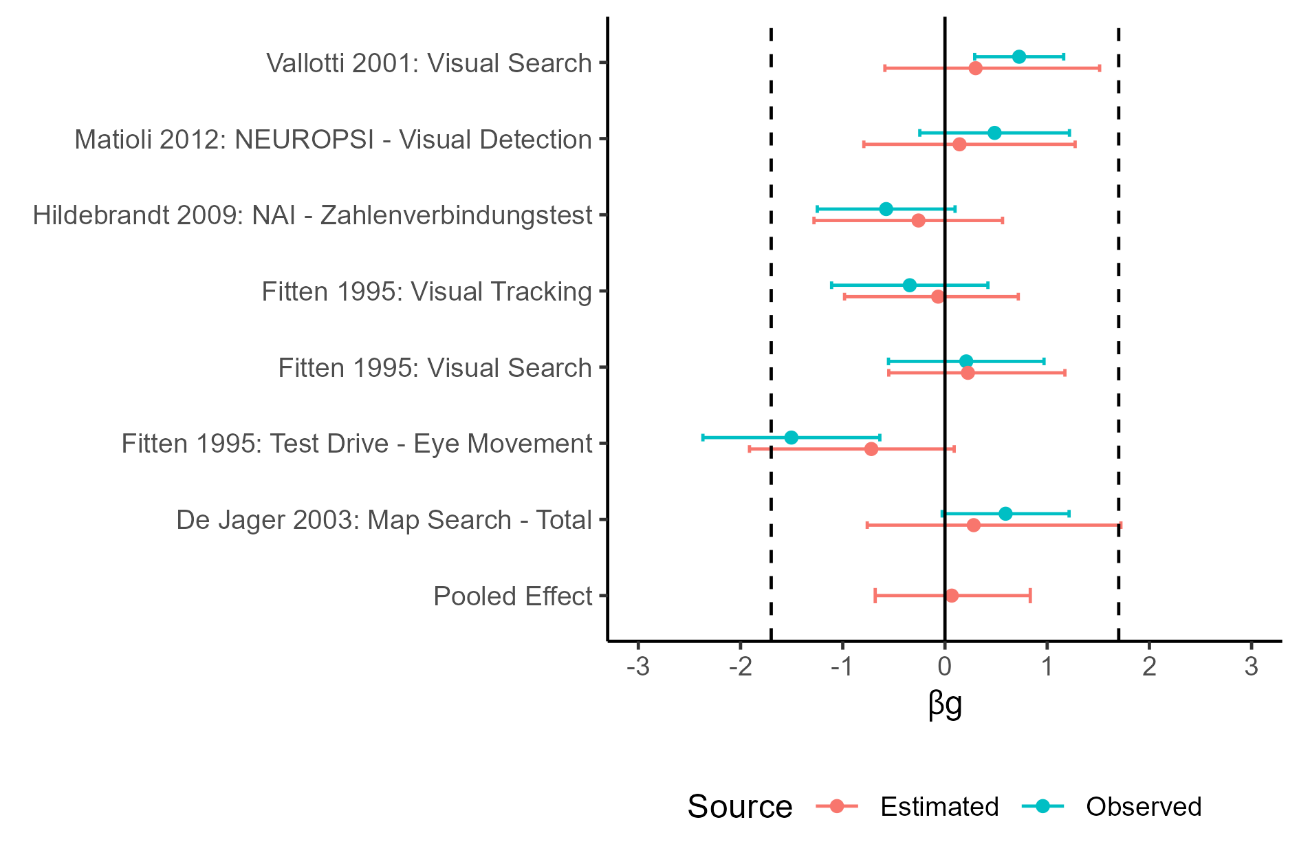


Figure 1. Forest plot for Other Measures of Visual Attention. Regression coefficients and 95% confidence intervals are displayed. Dashed vertical lines show the lower and upper bound of the Region of Practical Equivalence set at ±1.7 *g*. Estimated effect sizes are regression coefficient estimates and Observed effects are the effect sizes and confidence intervals from the included studies. NAI: Nürnberger Alersinventar.

## Other Measures of Attention


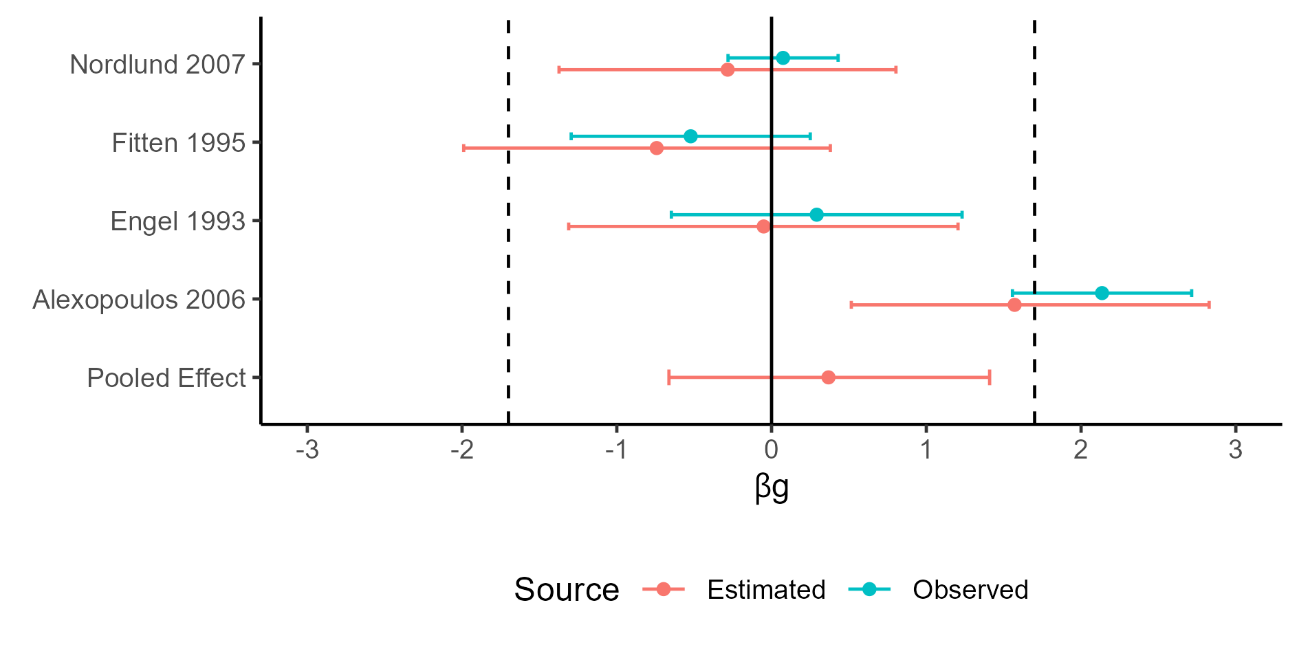


Figure 1. Forest plot for Other Measures of Attention. Regression coefficients and 95% confidence intervals are displayed. Dashed vertical lines show the lower and upper bound of the Region of Practical Equivalence set at ±1.7 *g*. Estimated effect sizes are regression coefficient estimates and Observed effects are the effect sizes and confidence intervals from the included studies. Tasks reported are: Alexopoulos et al. (2006): Addenbrooke’s Cognitive Examination – Attention scores; Engel et al. (1993): Neurobehavior Cognitive Status Examination - Attention scores; Fitten et al. (1995): a vigilance task; Nordlund et al. (2007): a dual task assessing divided attention.

## Attention: Quality Sensitivity Analysis


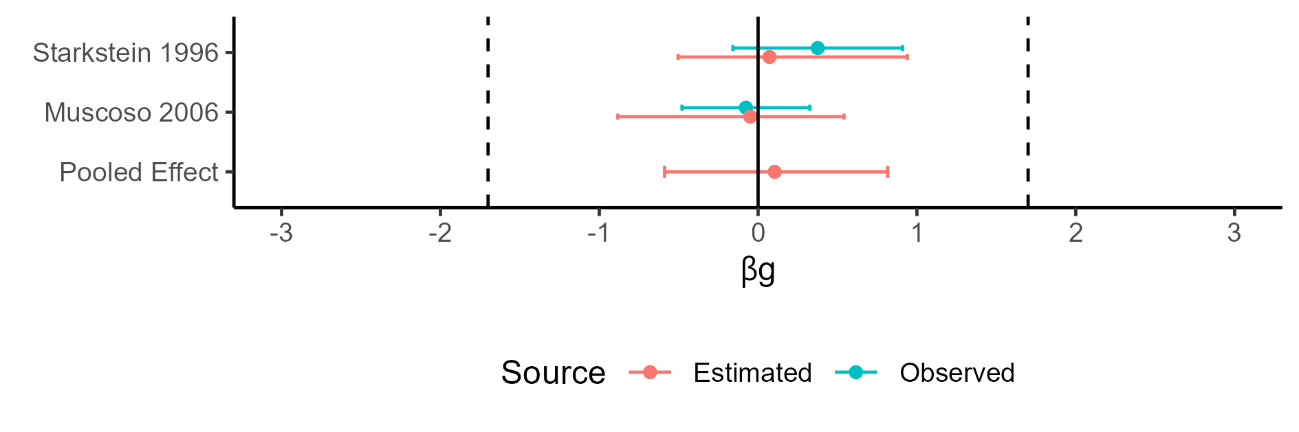


Figure 1. Forest plot for the Digit Span Forward study quality sensitivity analysis. Regression coefficients and 95% confidence intervals are displayed. Dashed vertical lines show the lower and upper bound of the Region of Practical Equivalence set at ±1.7 *g*. Estimated effect sizes are regression coefficient estimates and Observed effects are the effect sizes and confidence intervals from the included studies.

# Processing Speed

## Stroop Task Word Reading and Colour Naming


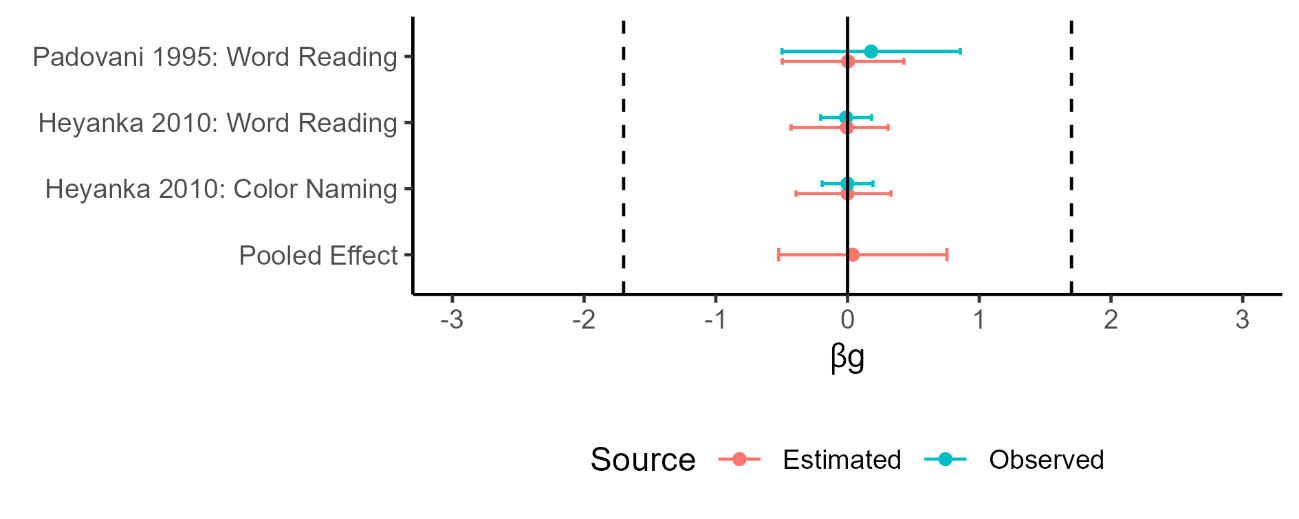


Figure 1. Forest plot for the Stroop Task: Word Reading and Colour Naming scores. Regression coefficients and 95% confidence intervals are displayed. Dashed vertical lines show the lower and upper bound of the Region of Practical Equivalence set at ±1.7 *g*. Estimated effect sizes are regression coefficient estimates and Observed effects are the effect sizes and confidence intervals from the included studies.

## Simple Reaction Time


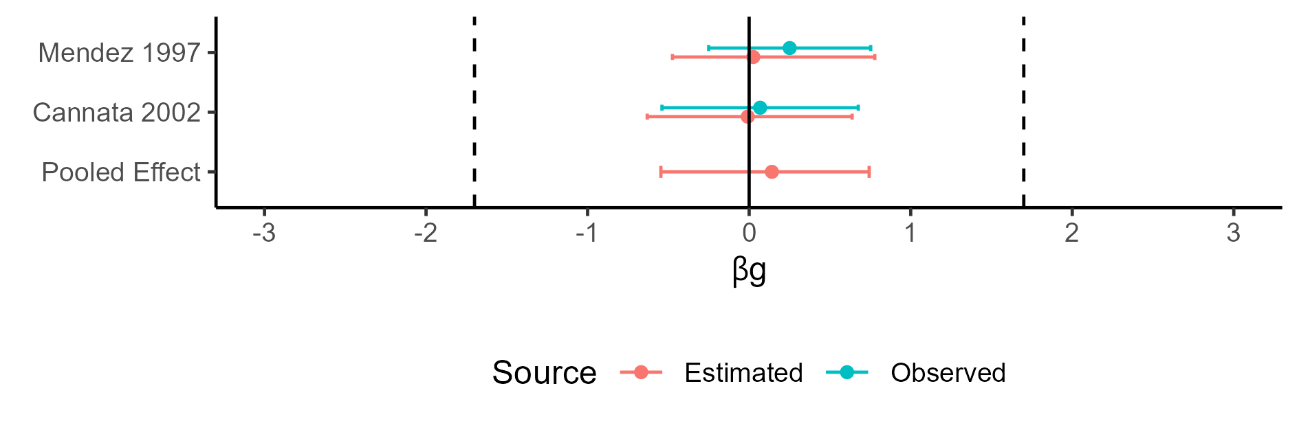


Figure 1. Forest plot for Simple Reaction Time tasks. Regression coefficients and 95% confidence intervals are displayed. Dashed vertical lines show the lower and upper bound of the Region of Practical Equivalence set at ±1.7 *g*. Estimated effect sizes are regression coefficient estimates and Observed effects are the effect sizes and confidence intervals from the included studies.

## Other Measures of Processing Speed


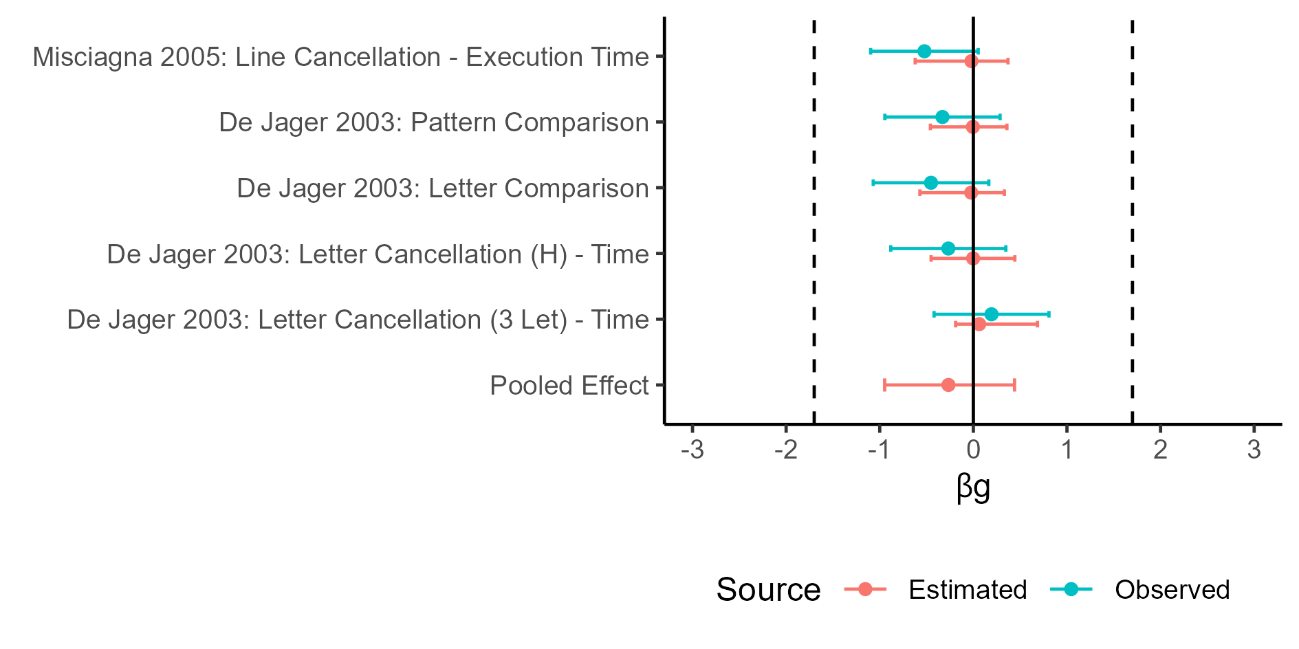


Figure 1. Forest plot for Other Measures of Processing Speed scores. Regression coefficients and 95% confidence intervals are displayed. Dashed vertical lines show the lower and upper bound of the Region of Practical Equivalence set at ±1.7 *g*. Estimated effect sizes are regression coefficient estimates and Observed effects are the effect sizes and confidence intervals from the included studies.

# Language Production

## Fluency: Multi-infarct Dementia


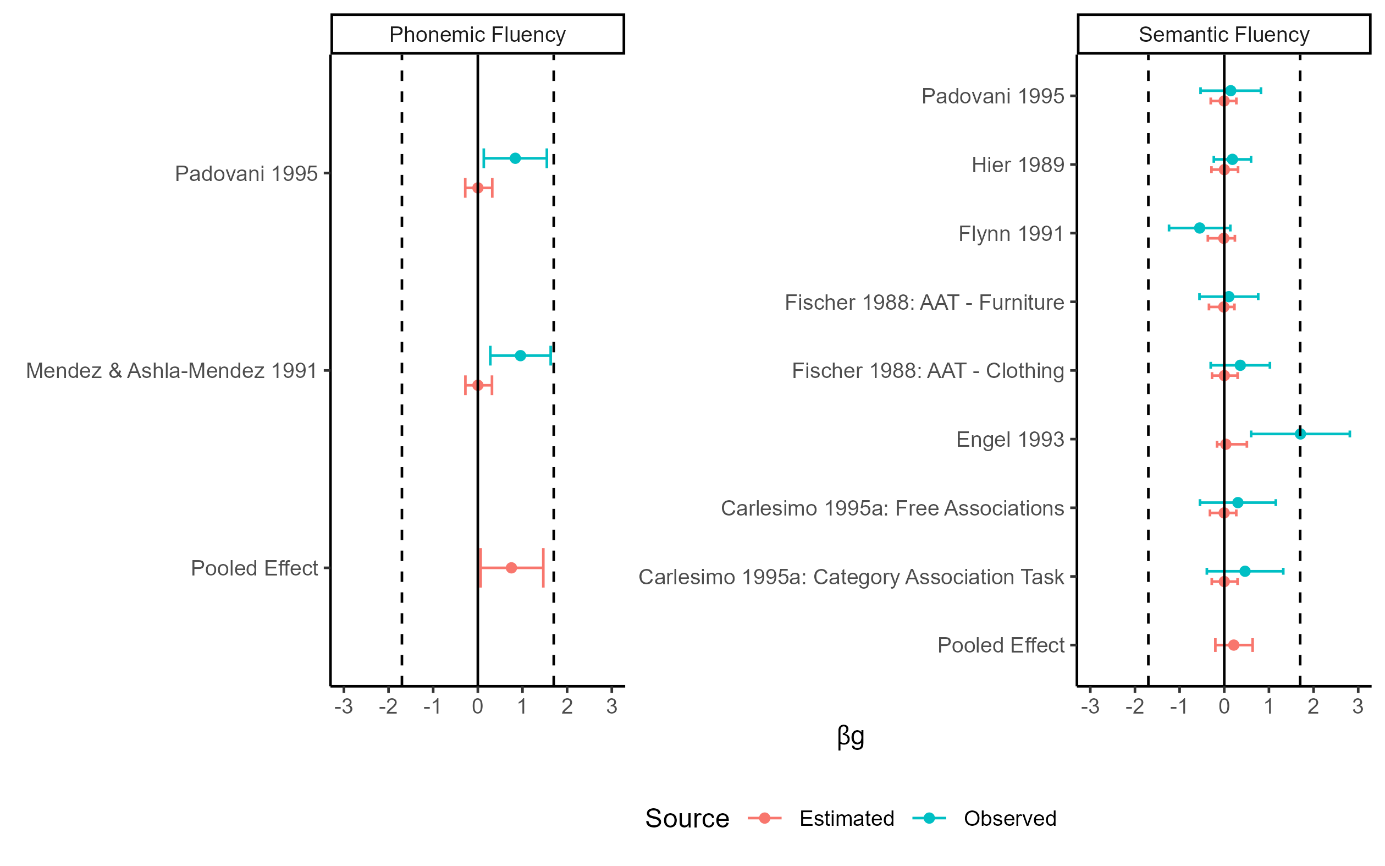


Figure 1. Forest plot for Verbal Fluency measures in multi-infarct dementia. Regression coefficients and 95% confidence intervals are displayed. Dashed vertical lines show the lower and upper bound of the Region of Practical Equivalence set at ±1.7 *g*. Estimated effect sizes are regression coefficient estimates and Observed effects are the effect sizes and confidence intervals from the included studies. AAT: Aachener Aphasie Test.

## Fluency: Vascular Dementia


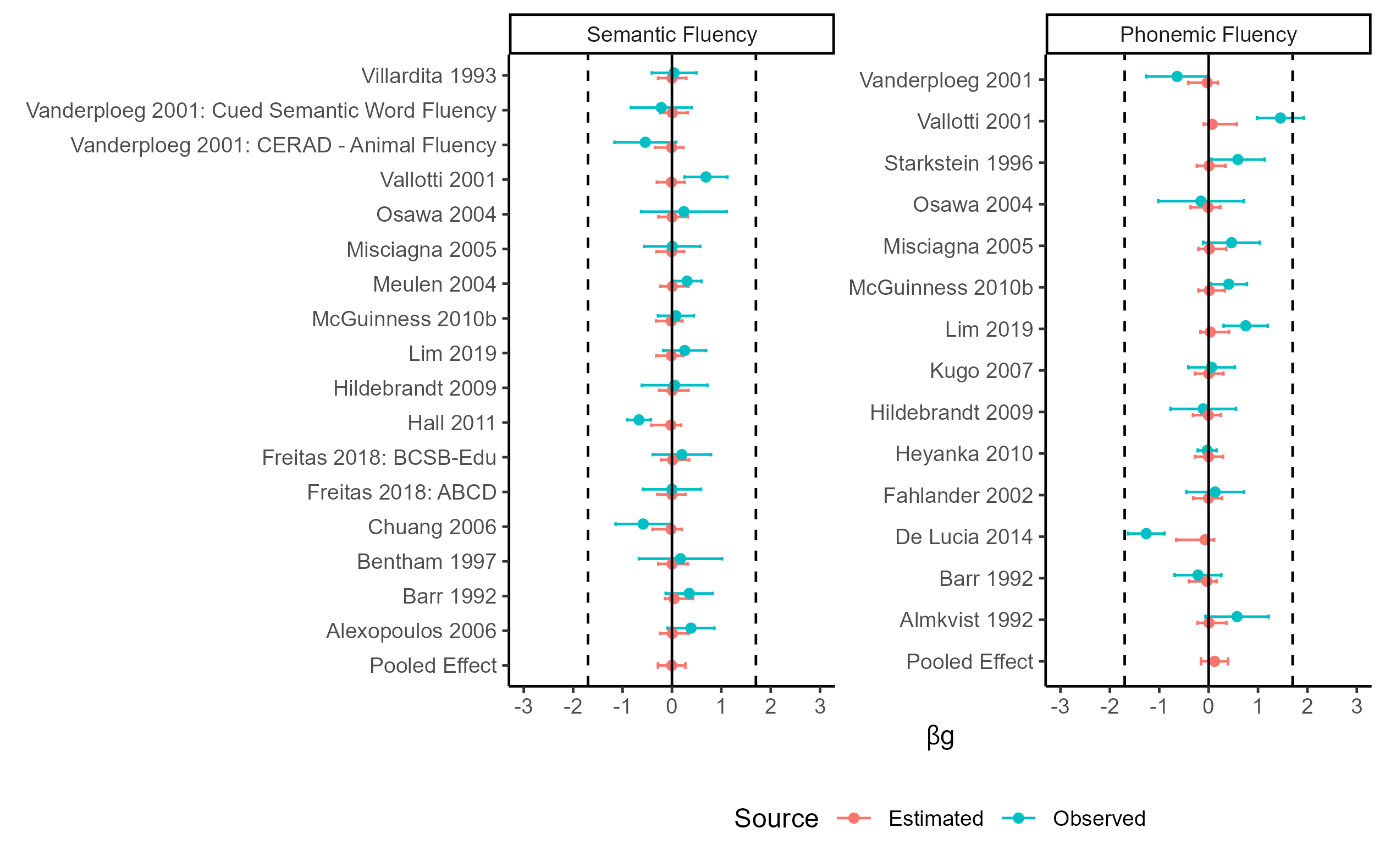


**Figure 1.** Forest plot for Verbal Fluency measures in vascular dementia. Regression coefficients and 95% confidence intervals are displayed. Dashed vertical lines show the lower and upper bound of the Region of Practical Equivalence set at ±1.7 *g*. Estimated effect sizes are regression coefficient estimates and Observed effects are the effect sizes and confidence intervals from the included studies. ABCD: Arizona Battery for Communication Disorders of Dementia, BCSB-Edu: Brief Cognitive Screening Battery-Edu, CERAD: Consortium to Establish a Registry for Alzheimer’s Disease.

## Fluency: Subcortical Vascular Dementia


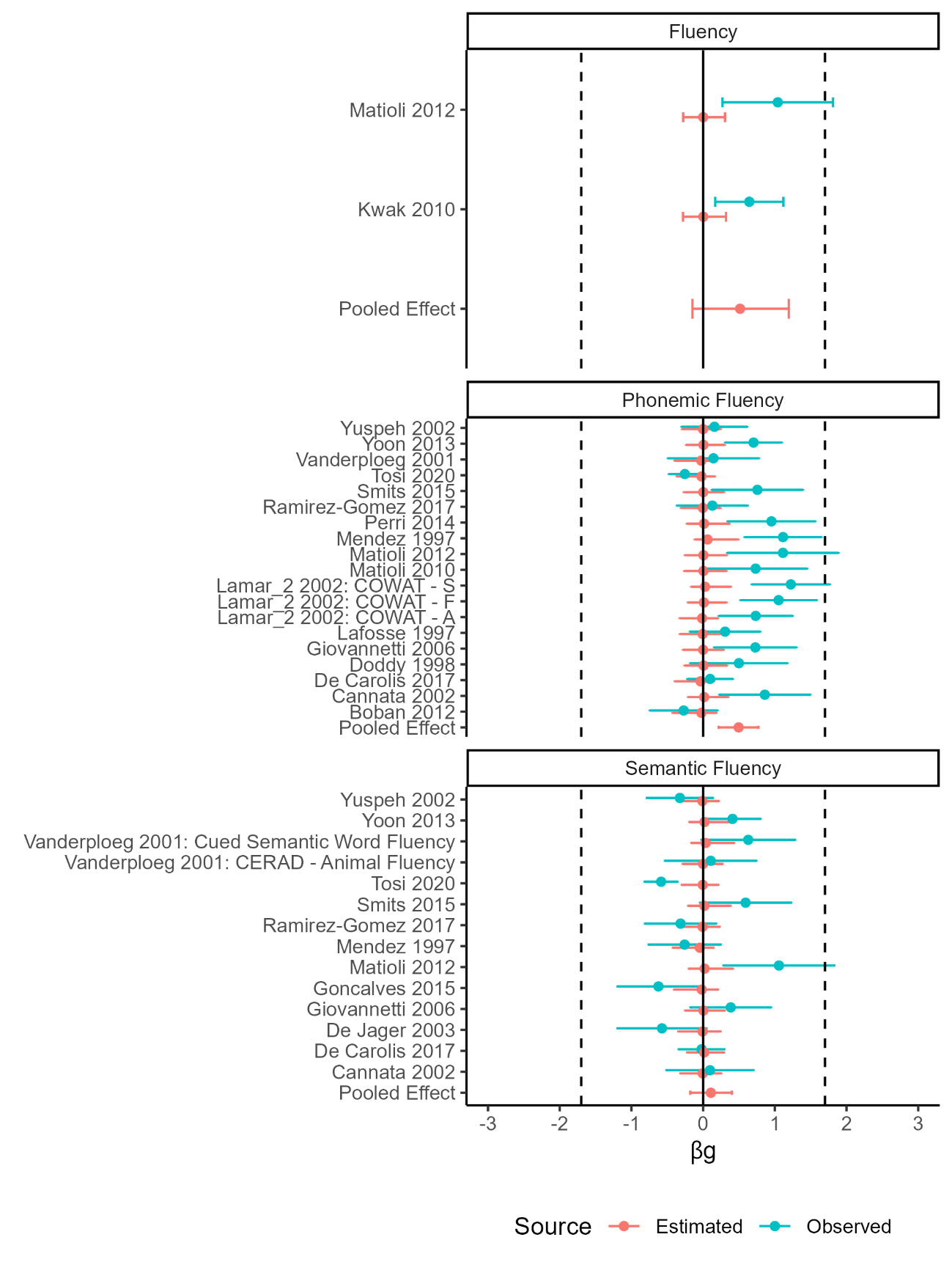


**Figure 1.** Forest plot for Verbal Fluency measures in subcortical vascular dementia. Regression coefficients and 95% confidence intervals are displayed. Dashed vertical lines show the lower and upper bound of the Region of Practical Equivalence set at ±1.7 *g*. Estimated effect sizes are regression coefficient estimates and Observed effects are the effect sizes and confidence intervals from the included studies. CERAD: Consortium to Establish a Registry for Alzheimer’s Disease, COWAT: Controlled Oral Word Association Test.

## Fluency: Vascular Cognitive Impairment


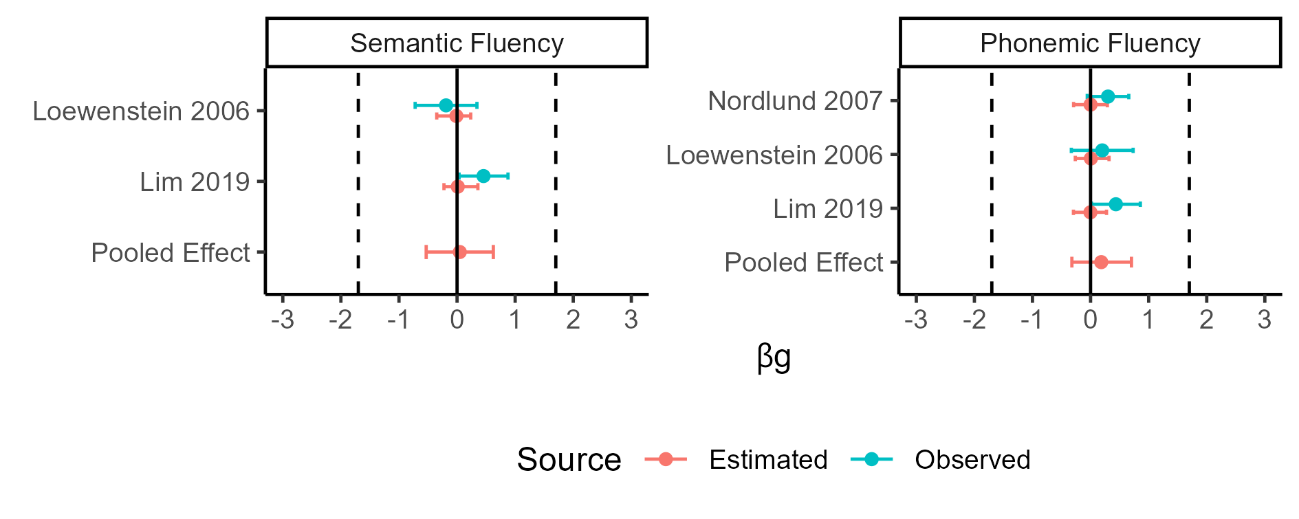


**Figure 1.** Forest plot for Verbal Fluency measures in vascular cognitive impairment. Regression coefficients and 95% confidence intervals are displayed. Dashed vertical lines show the lower and upper bound of the Region of Practical Equivalence set at ±1.7 *g*. Estimated effect sizes are regression coefficient estimates and Observed effects are the effect sizes and confidence intervals from the included studies.

## Boston Naming Test


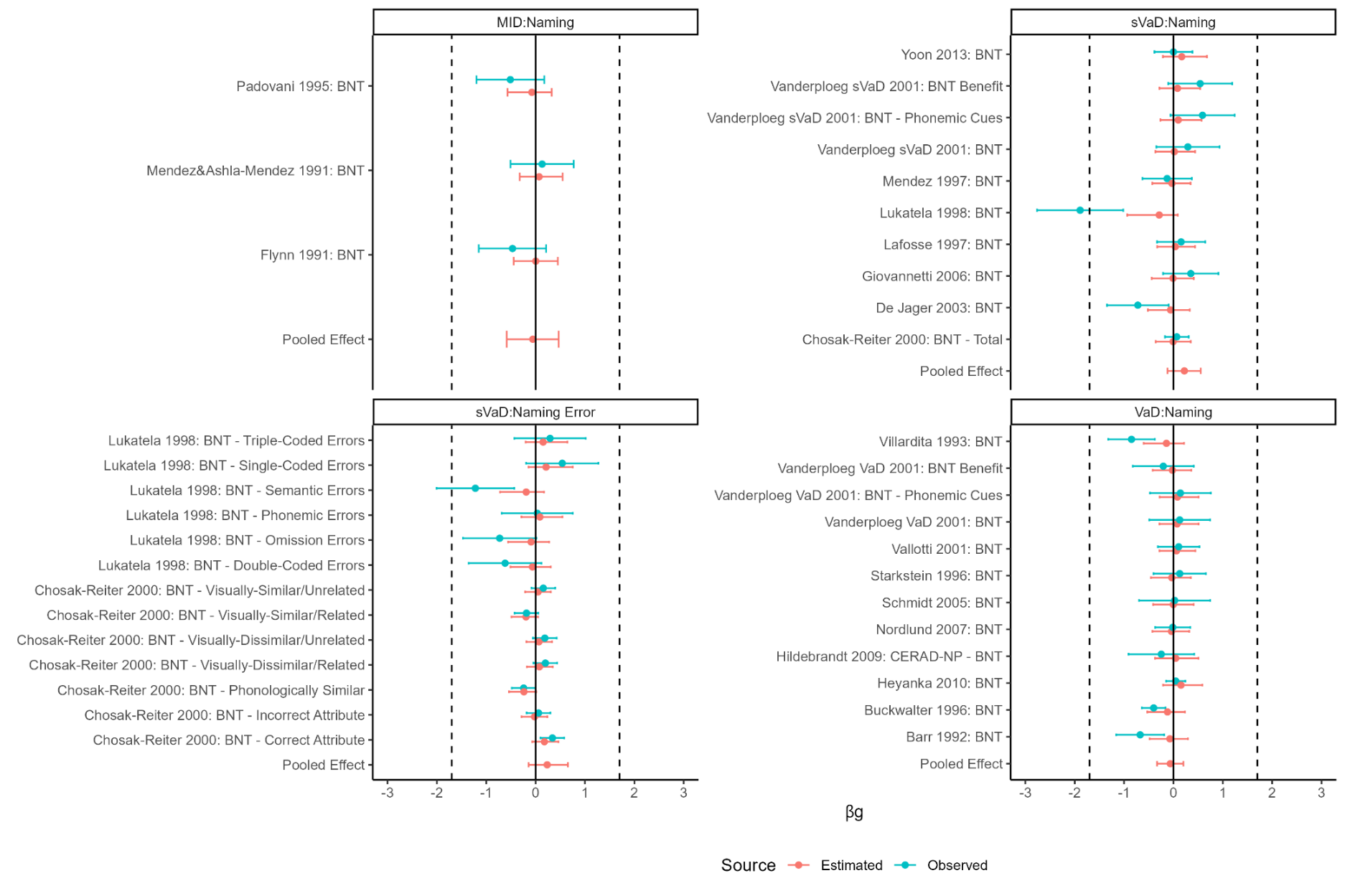


Figure 1. Forest plot for the Boston Naming Test (BNT). Regression coefficients and 95% confidence intervals are displayed. Dashed vertical lines show the lower and upper bound of the Region of Practical Equivalence set at ±1.7 *g*. Estimated effect sizes are regression coefficient estimates and Observed effects are the effect sizes and confidence intervals from the included studies.

## Other Naming Measures


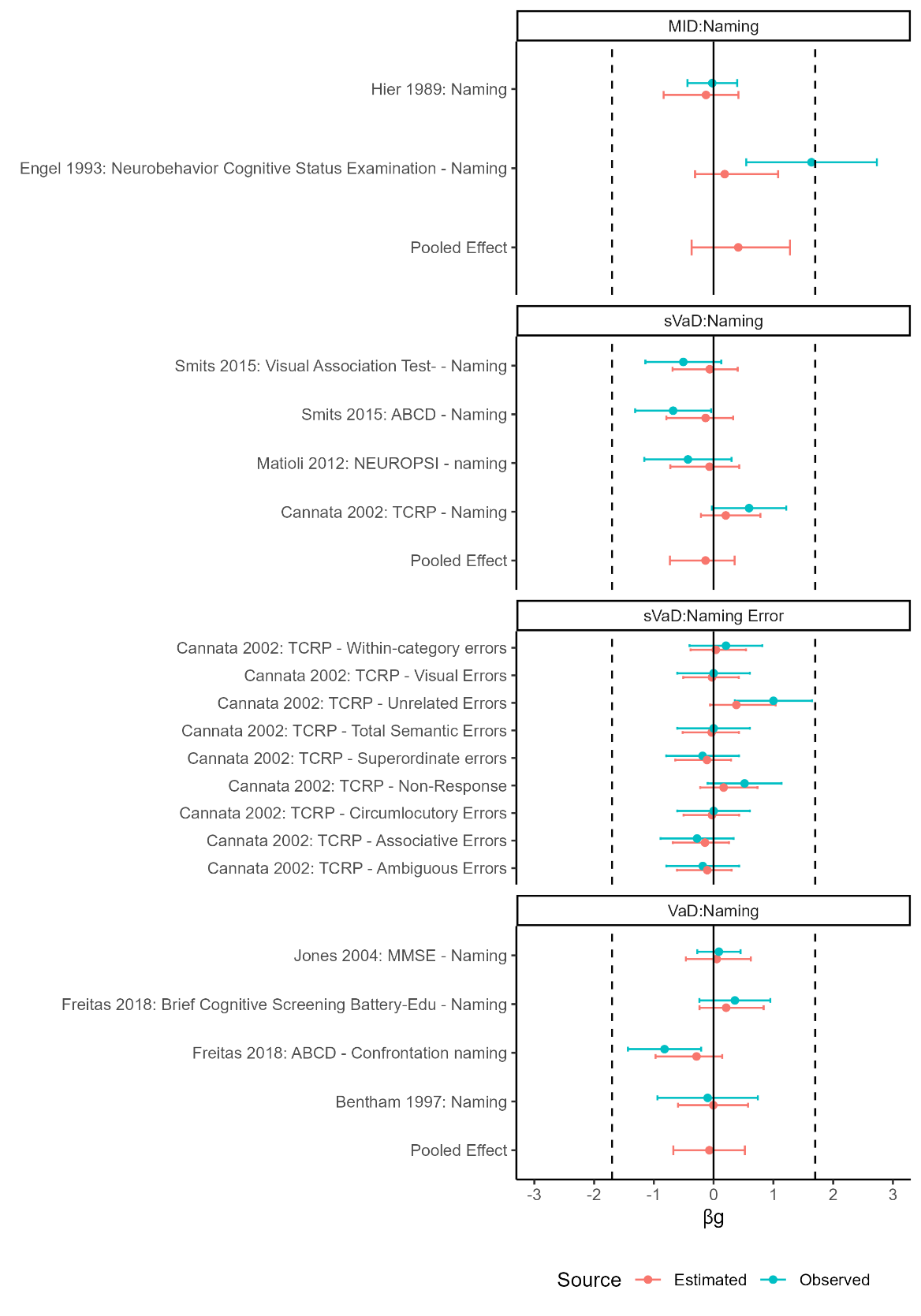


Figure 1. Forest plot for Other Naming Measures. Regression coefficients and 95% confidence intervals are displayed. Dashed vertical lines show the lower and upper bound of the Region of Practical Equivalence set at ±1.7 *g*. Estimated effect sizes are regression coefficient estimates and Observed effects are the effect sizes and confidence intervals from the included studies. ABCD: Arizona Battery for Communication Disorders of Dementia; MMSE: Mini Mental State Examination; TCRP: Test of Classification and Recall of Pictures.

## Addenbrooke’s Cognitive Examination


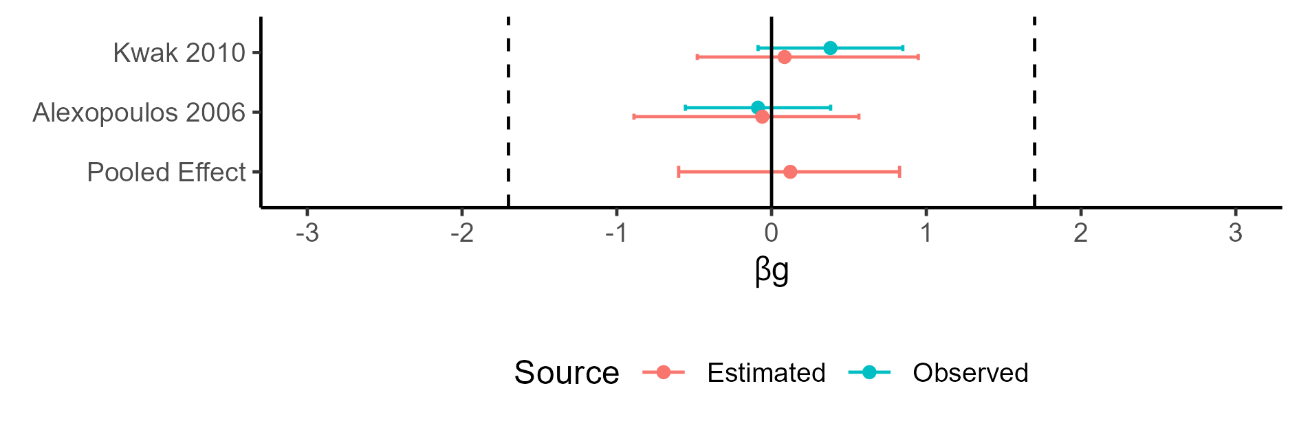


Figure 1. Forest plot for the Addenbrooke’s Cognitive Examination: Naming subscale. Regression coefficients and 95% confidence intervals are displayed. Dashed vertical lines show the lower and upper bound of the Region of Practical Equivalence set at ±1.7 *g*. Estimated effect sizes are regression coefficient estimates and Observed effects are the effect sizes and confidence intervals from the included studies.

## Writing


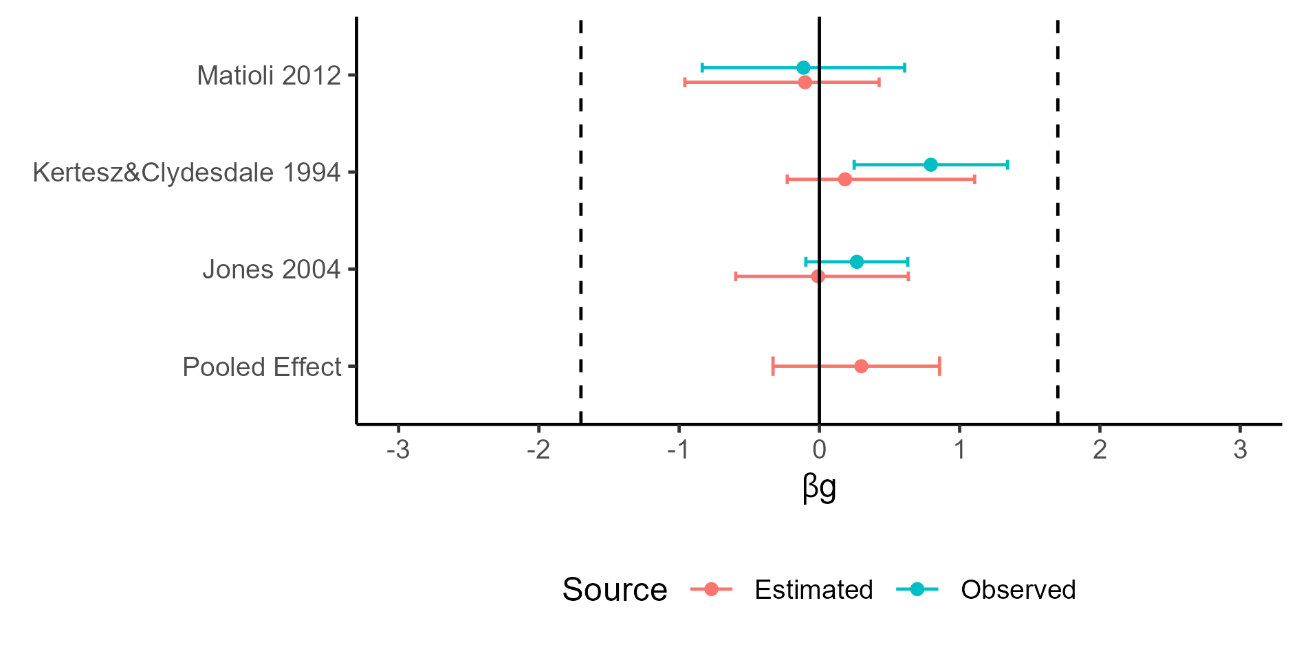


Figure 1. Forest plot for measures of Writing: Naming subscale. Regression coefficients and 95% confidence intervals are displayed. Dashed vertical lines show the lower and upper bound of the Region of Practical Equivalence set at ±1.7 *g*. Estimated effect sizes are regression coefficient estimates and Observed effects are the effect sizes and confidence intervals from the included studies.

## Other Measures of Language Production


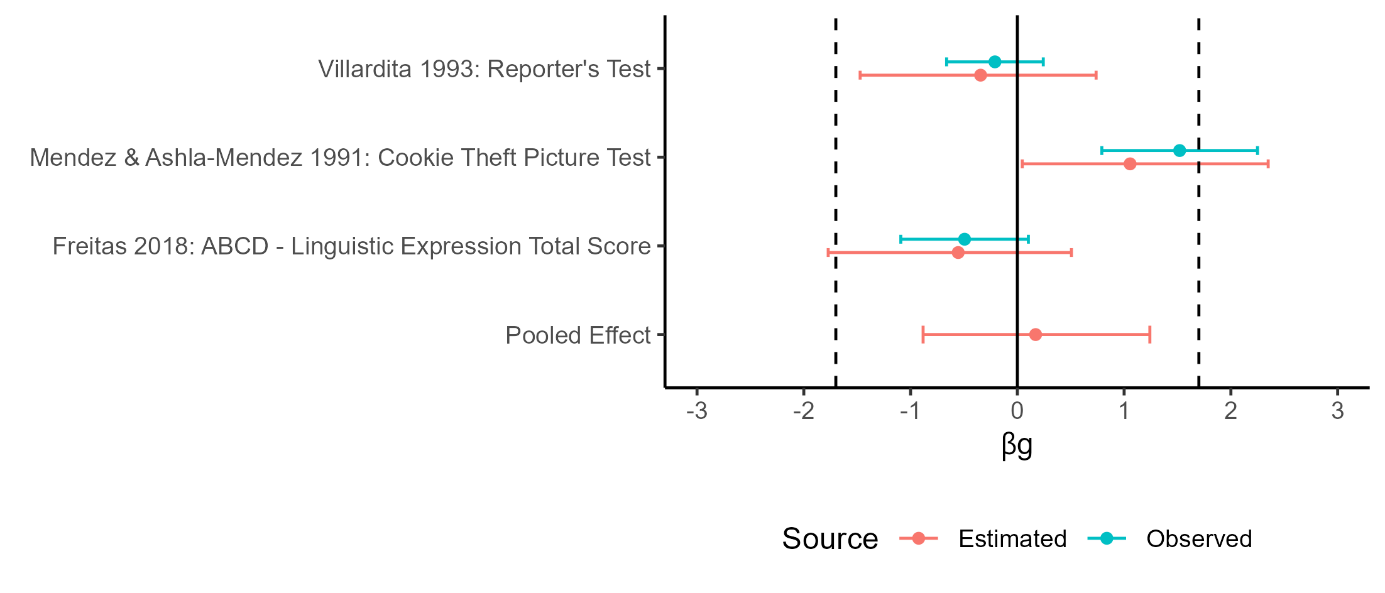


Figure 1. Forest plot for Other Measures of Language Production. Regression coefficients and 95% confidence intervals are displayed. Dashed vertical lines show the lower and upper bound of the Region of Practical Equivalence set at ±1.7 *g*. Estimated effect sizes are regression coefficient estimates and Observed effects are the effect sizes and confidence intervals from the included studies. ABCD: Arizona Battery for Communication Disorders of Dementia

## Boston Naming Test: Quality Sensitivity Analysis


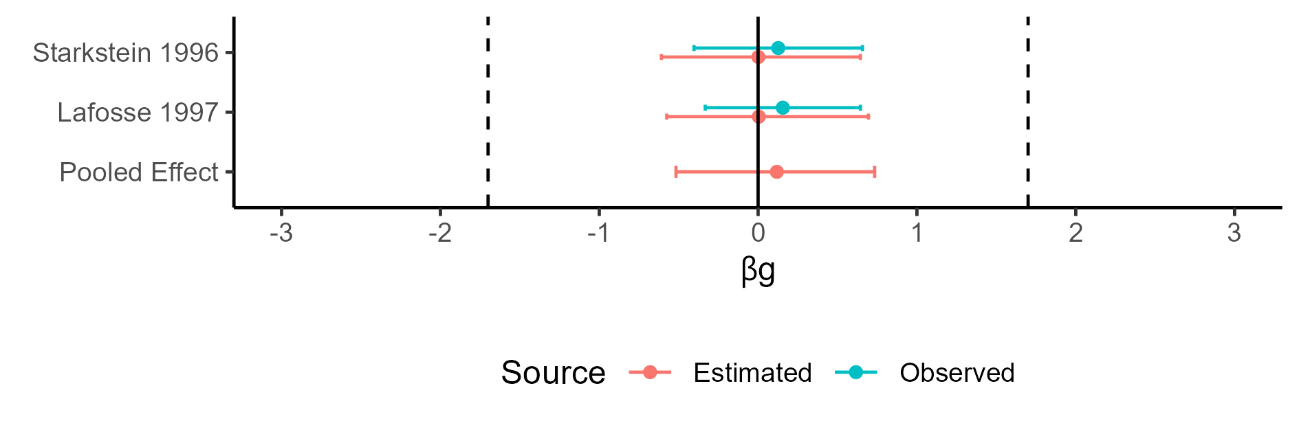


Figure 1. Forest plot of the study quality sensitivity analysis for the Boston Naming Test. Regression coefficients and 95% confidence intervals are displayed. Dashed vertical lines show the lower and upper bound of the Region of Practical Equivalence set at ±1.7 *g*. Estimated effect sizes are regression coefficient estimates and Observed effects are the effect sizes and confidence intervals from the included studies.

## Phonemic Fluency: Quality Sensitivity Analysis


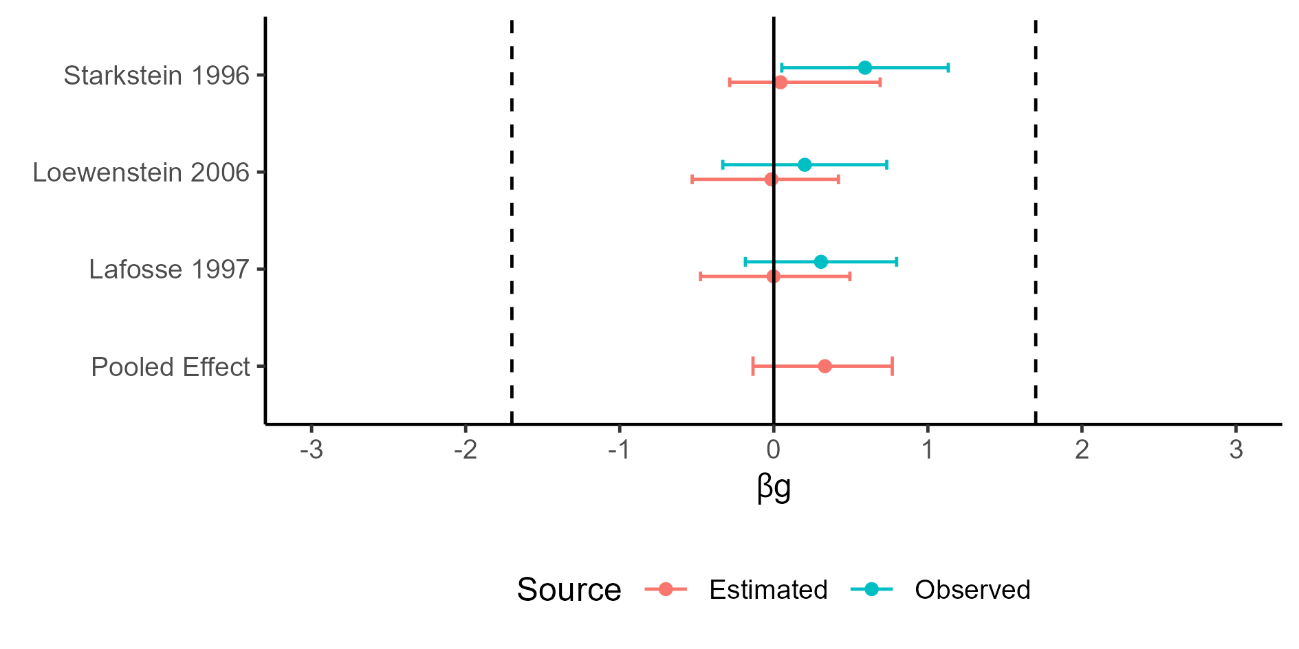


Figure 1. Forest plot of the study quality sensitivity analysis for Phonemic Fluency. Regression coefficients and 95% confidence intervals are displayed. Dashed vertical lines show the lower and upper bound of the Region of Practical Equivalence set at ±1.7 *g*. Estimated effect sizes are regression coefficient estimates and Observed effects are the effect sizes and confidence intervals from the included studies.

# Language Comprehension

## Token Test


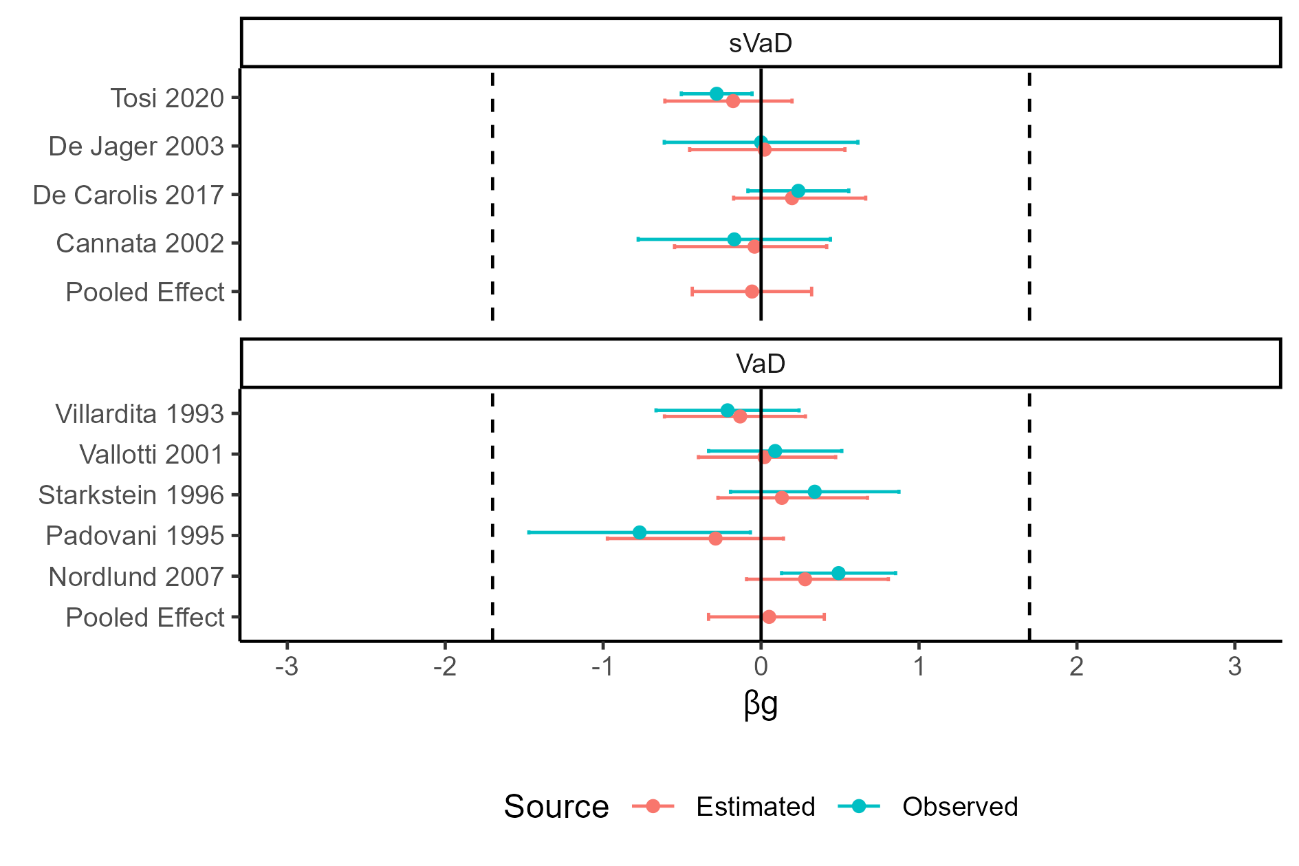


Figure 1. Forest plot for the Token Test. Regression coefficients and 95% confidence intervals are displayed. Dashed vertical lines show the lower and upper bound of the Region of Practical Equivalence set at ±1.7 *g*. Estimated effect sizes are regression coefficient estimates and Observed effects are the effect sizes and confidence intervals from the included studies.

## Aphasia Inventories


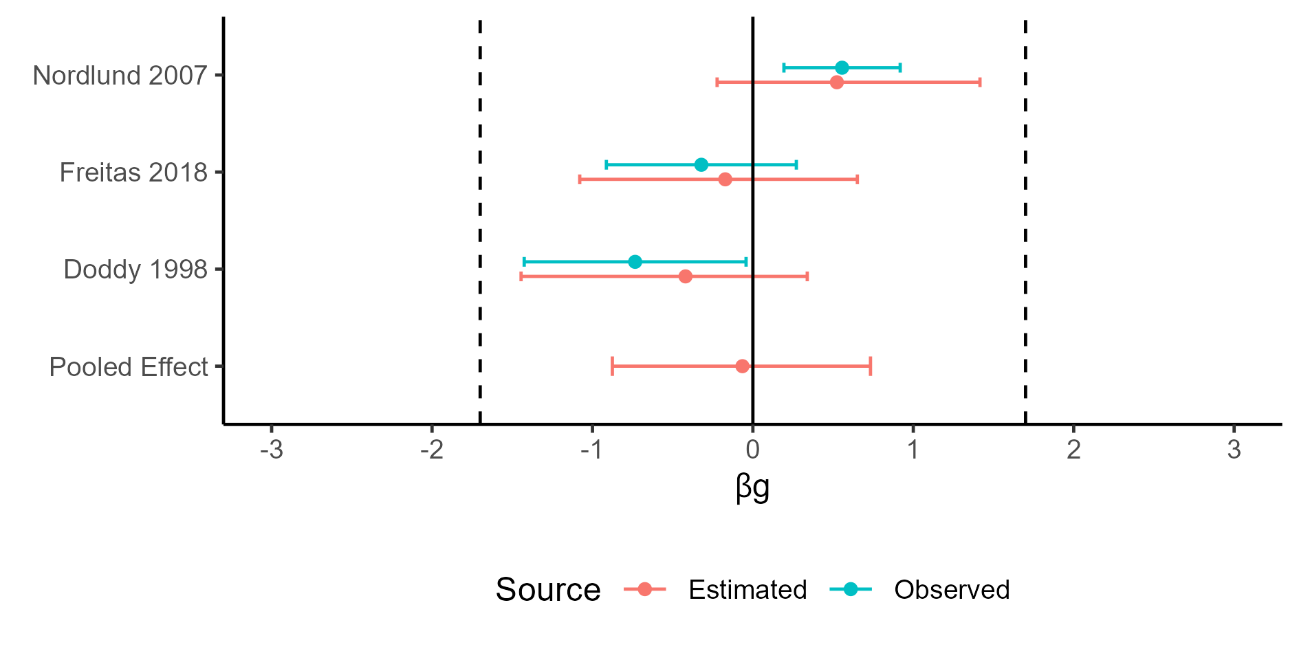


Figure 1. Forest plot for the Aphasia Inventories. Regression coefficients and 95% confidence intervals are displayed. Dashed vertical lines show the lower and upper bound of the Region of Practical Equivalence set at ±1.7 *g*. Estimated effect sizes are regression coefficient estimates and Observed effects are the effect sizes and confidence intervals from the included studies. The reported aphasia inventories are: Assessment of Subtle Language Deficits Logical Grammar subtest (Nordlund et al. 2007), and the Arizona Battery for Communication Disorders of Dementia: Linguistic Comprehension (Freitas et al. 2018); Western Aphasia Battery: Segmental Commands (Doddy et al. 1998).

## Other Measures of Language Comprehension


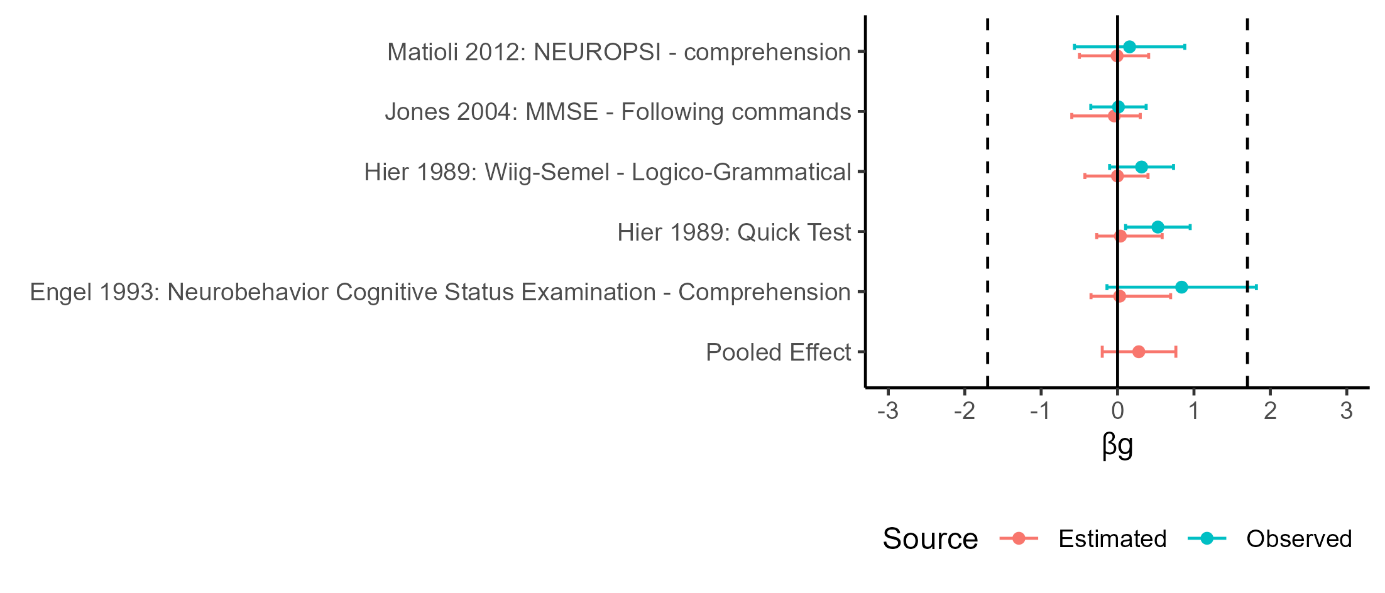


Figure 1. Forest plot for Other Measures of Language Comprehension. Regression coefficients and 95% confidence intervals are displayed. Dashed vertical lines show the lower and upper bound of the Region of Practical Equivalence set at ±1.7 *g*. Estimated effect sizes are regression coefficient estimates and Observed effects are the effect sizes and confidence intervals from the included studies. MMSE: Mini-Mental-State-Examination.

# Reading


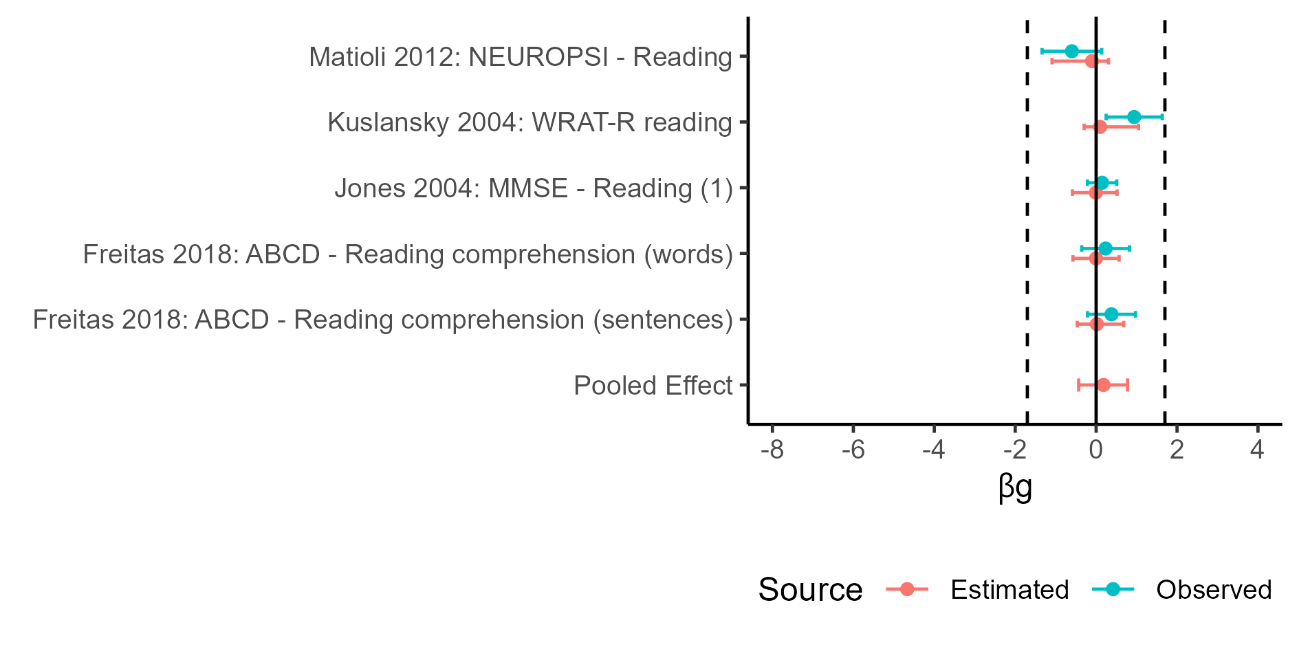


Figure 1. Forest plot for tests of Reading ability. Regression coefficients and 95% confidence intervals are displayed. Dashed vertical lines show the lower and upper bound of the Region of Practical Equivalence set at ±1.7 *g*. Estimated effect sizes are regression coefficient estimates and Observed effects are the effect sizes and confidence intervals from the included studies. ABCD: Arizona Battery for Communication Disorders of Dementia; MMSE: Mini-Mental-State-Examination; WRAT-R: Wide Range Achievement Test.

# Reasoning

## Wechsler Adult Intelligence Scale


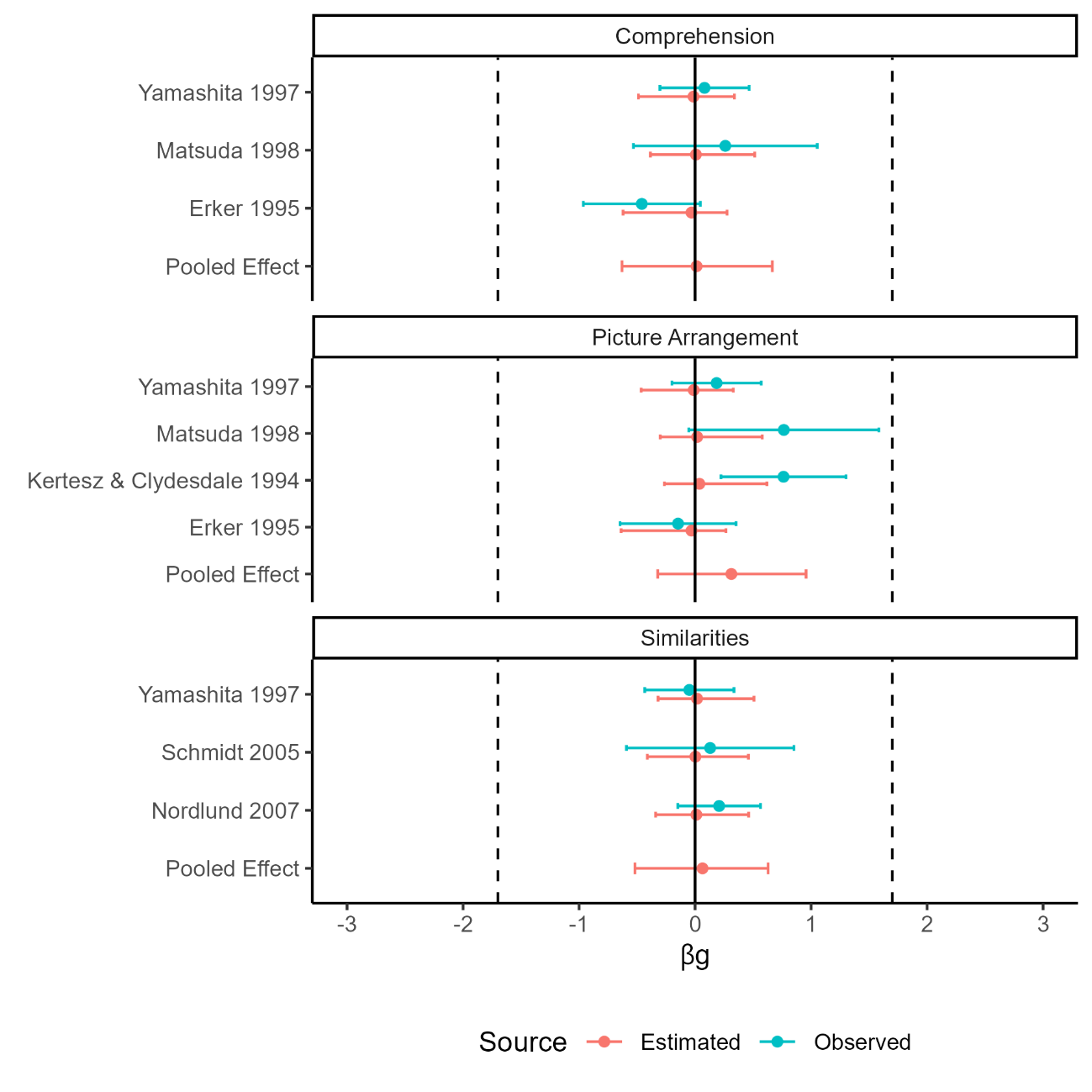


Figure 1. Forest plot for the Wechsler Adult Intelligence Scale reasoning subtests. Regression coefficients and 95% confidence intervals are displayed. Dashed vertical lines show the lower and upper bound of the Region of Practical Equivalence set at ±1.7 *g*. Estimated effect sizes are regression coefficient estimates and Observed effects are the effect sizes and confidence intervals from the included studies.

## Wisconsin Card Sorting Test


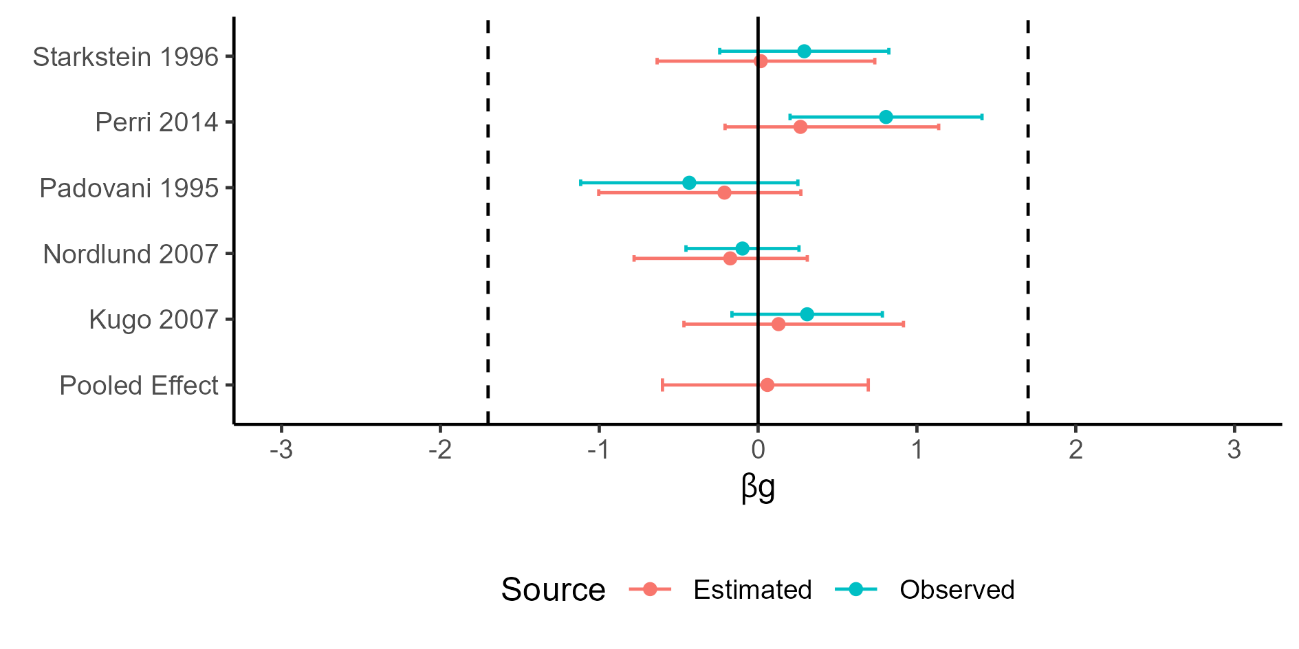


Figure 1. Forest plot for the Wisconsin Card Sorting Test: number of correct categories. Regression coefficients and 95% confidence intervals are displayed. Dashed vertical lines show the lower and upper bound of the Region of Practical Equivalence set at ±1.7 *g*. Estimated effect sizes are regression coefficient estimates and Observed effects are the effect sizes and confidence intervals from the included studies.

## Raven’s Progressive Matrices


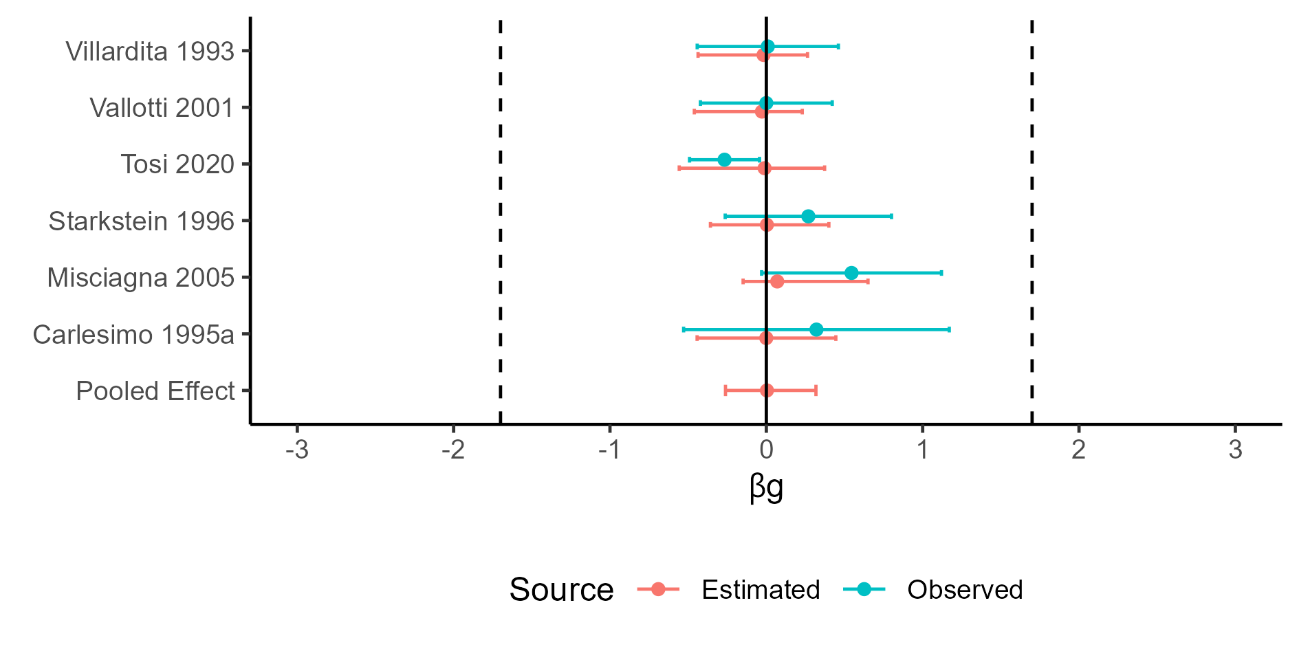


Figure 1. Forest plot for the Raven’s Progressive Matrices test. Regression coefficients and 95% confidence intervals are displayed. Dashed vertical lines show the lower and upper bound of the Region of Practical Equivalence set at ±1.7 *g*. Estimated effect sizes are regression coefficient estimates and Observed effects are the effect sizes and confidence intervals from the included studies.

## Raven’s Coloured Progressive Matrices


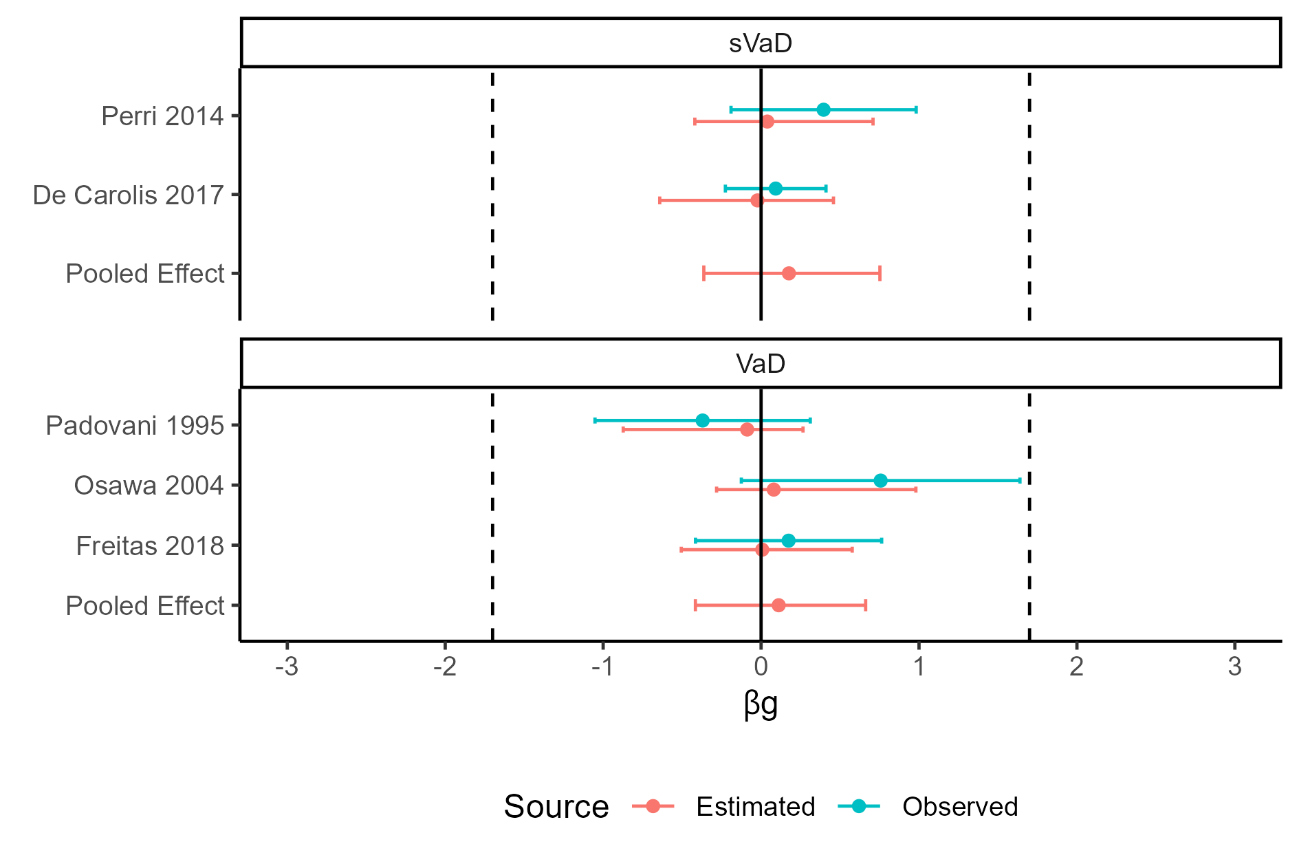


Figure 1. Forest plot for the Raven’s Coloured Progressive Matrices test. Regression coefficients and 95% confidence intervals are displayed. Dashed vertical lines show the lower and upper bound of the Region of Practical Equivalence set at ±1.7 *g*. Estimated effect sizes are regression coefficient estimates and Observed effects are the effect sizes and confidence intervals from the included studies.

## Attentional Matrices


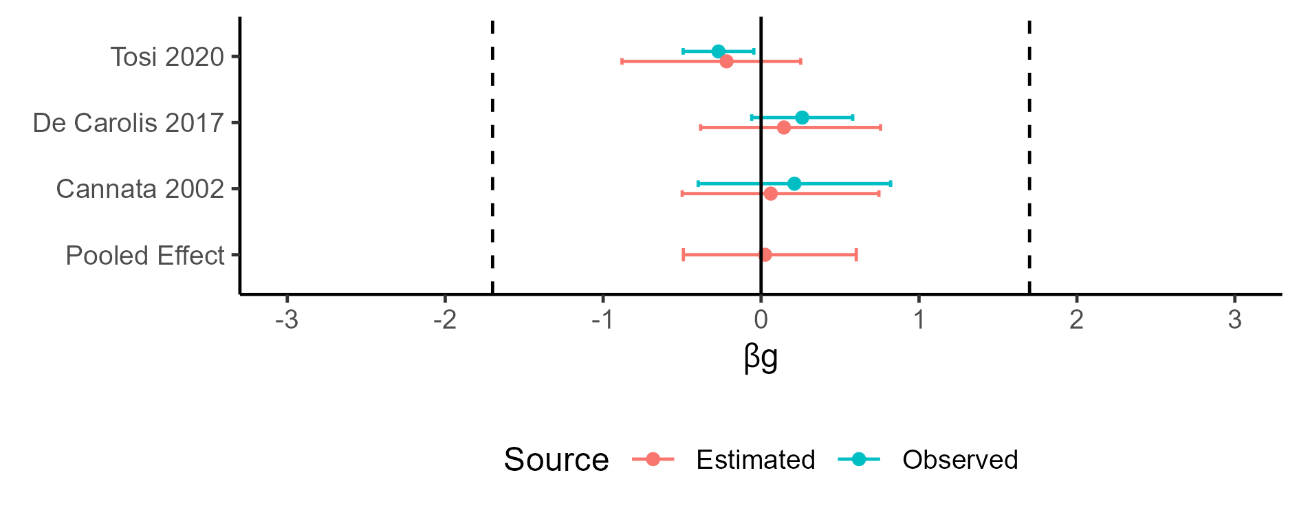


Figure 1. Forest plot for the Attentional Matrices test. Regression coefficients and 95% confidence intervals are displayed. Dashed vertical lines show the lower and upper bound of the Region of Practical Equivalence set at ±1.7 *g*. Estimated effect sizes are regression coefficient estimates and Observed effects are the effect sizes and confidence intervals from the included studies.

## Frontal Assessment Battery: Abstraction


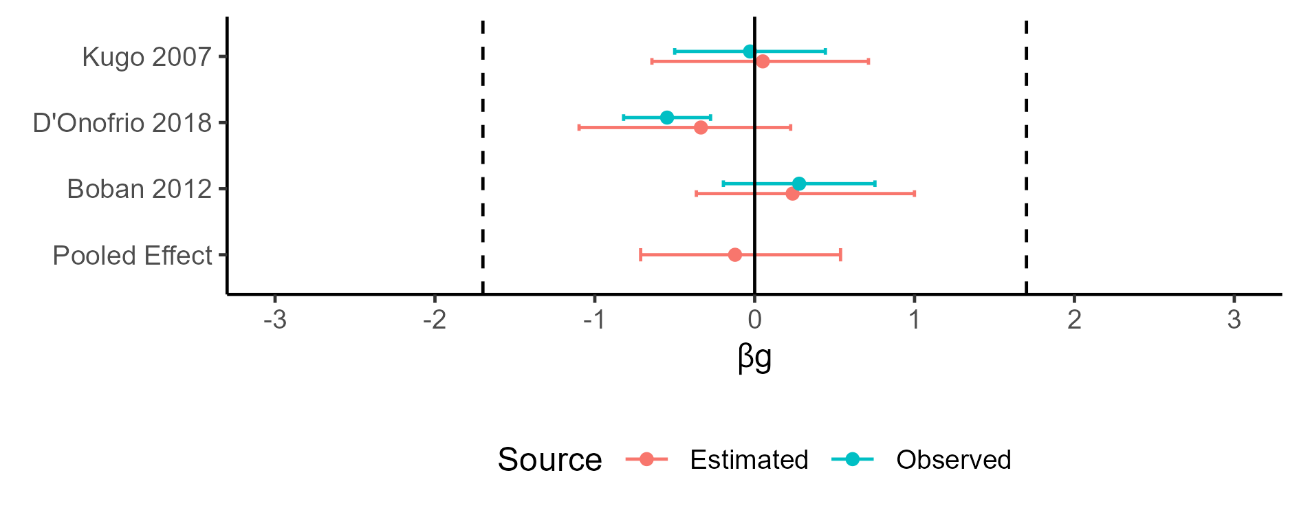


Figure 1. Forest plot for the Frontal Assessment Battery: Abstraction task. Regression coefficients and 95% confidence intervals are displayed. Dashed vertical lines show the lower and upper bound of the Region of Practical Equivalence set at ±1.7 *g*. Estimated effect sizes are regression coefficient estimates and Observed effects are the effect sizes and confidence intervals from the included studies.

## Other Abstraction Measures


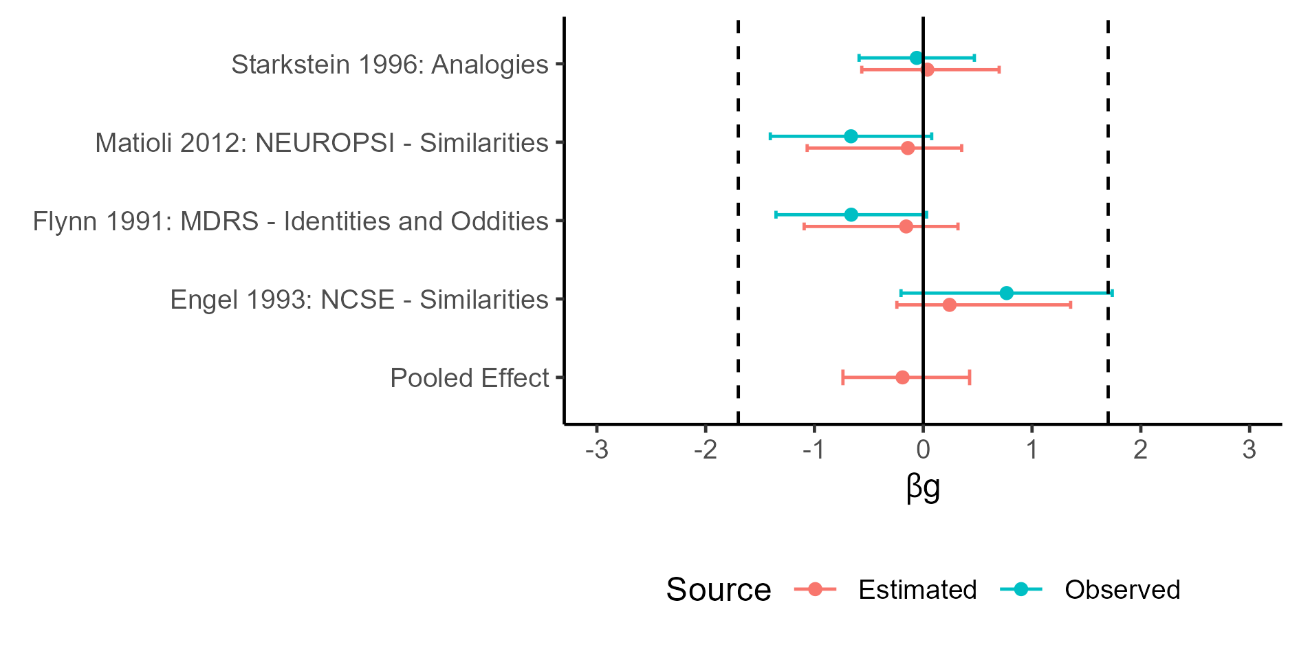


Figure 1. Forest plot for Other Abstraction Measures. Regression coefficients and 95% confidence intervals are displayed. Dashed vertical lines show the lower and upper bound of the Region of Practical Equivalence set at ±1.7 *g*. Estimated effect sizes are regression coefficient estimates and Observed effects are the effect sizes and confidence intervals from the included studies. MDRS: Mattis Dementia Rating Scale; NCSE: Neurobehavior Cognitive Status Examination.

## Other Reasoning Measures


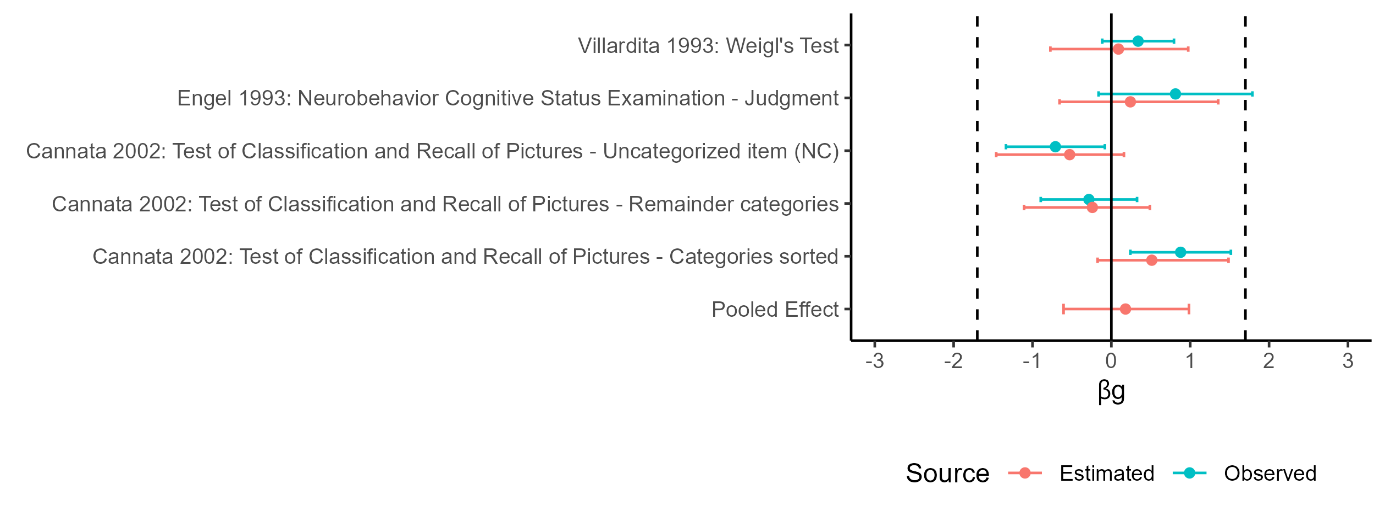


Figure 1. Forest plot for Other Reasoning Measures. Regression coefficients and 95% confidence intervals are displayed. Dashed vertical lines show the lower and upper bound of the Region of Practical Equivalence set at ±1.7 *g*. Estimated effect sizes are regression coefficient estimates and Observed effects are the effect sizes and confidence intervals from the included studies.

# Executive Functioning

## Wechsler Adult Intelligence Scale


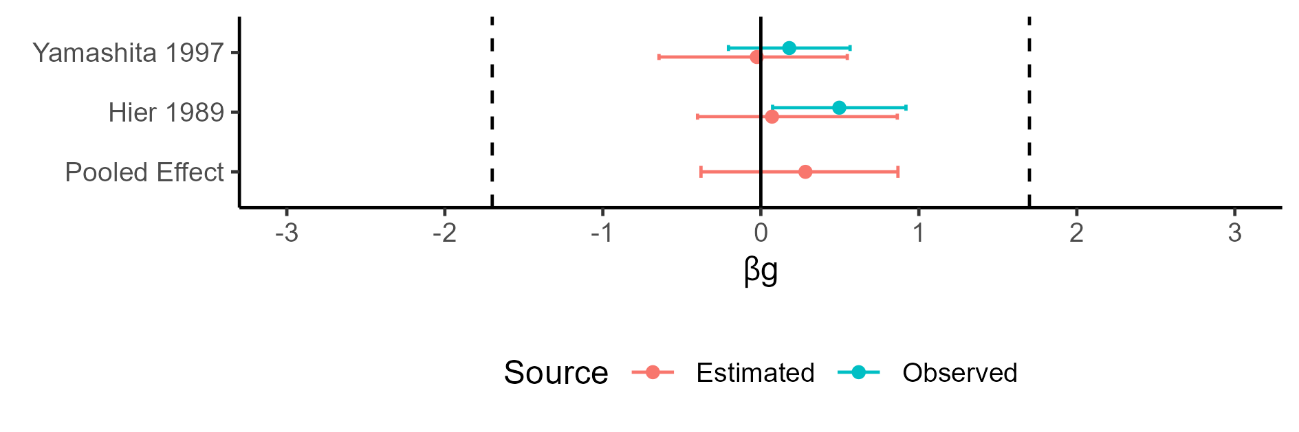


Figure 1. Forest plot for the Wechsler Adult Intelligence Scale: Arithmetic and Total Digits scores. Regression coefficients and 95% confidence intervals are displayed. Dashed vertical lines show the lower and upper bound of the Region of Practical Equivalence set at ±1.7 *g*. Estimated effect sizes are regression coefficient estimates and Observed effects are the effect sizes and confidence intervals from the included studies.

## Trail Making Test


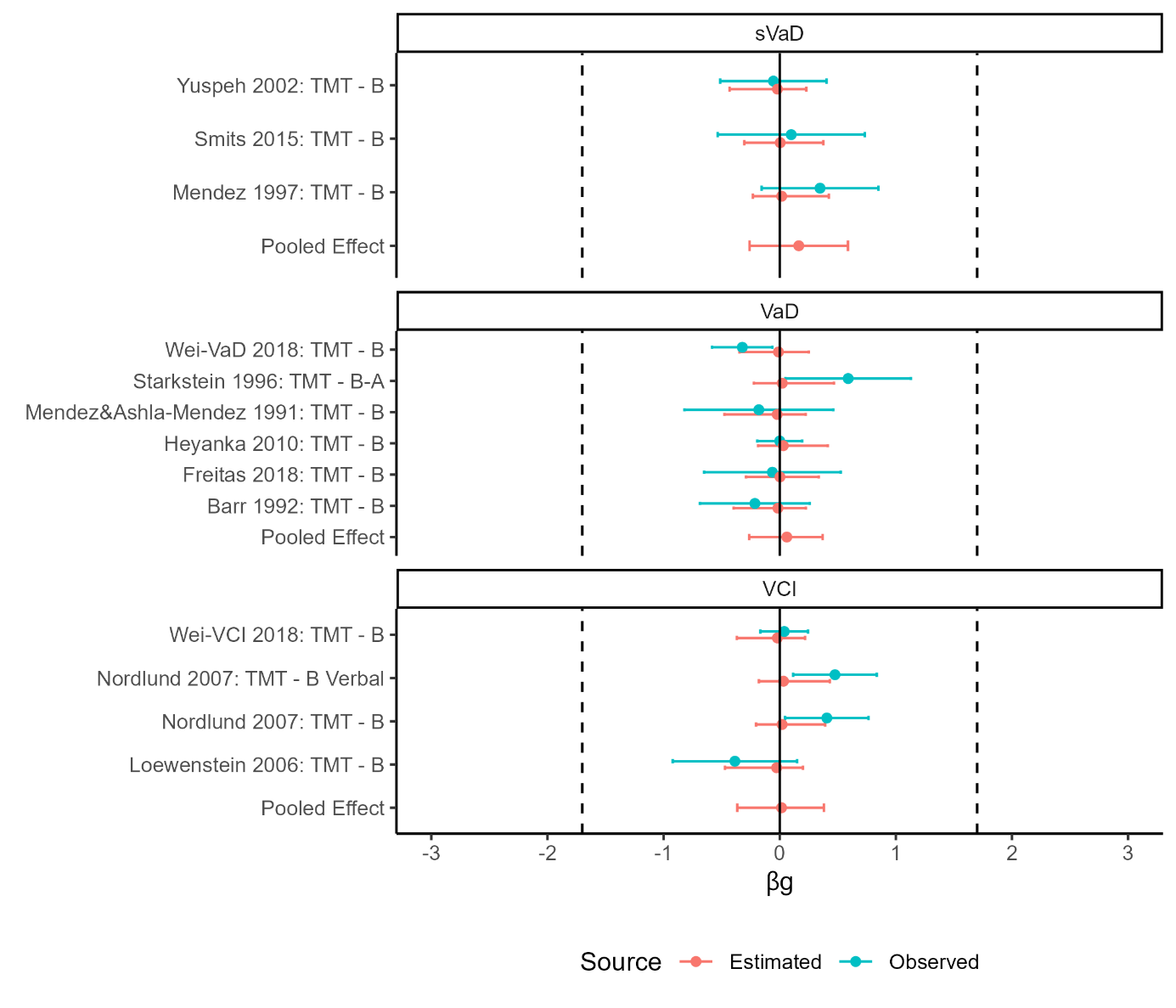


Figure 1. Forest plot for the Trail Making Test: B measures. Regression coefficients and 95% confidence intervals are displayed. Dashed vertical lines show the lower and upper bound of the Region of Practical Equivalence set at ±1.7 *g*. Estimated effect sizes are regression coefficient estimates and Observed effects are the effect sizes and confidence intervals from the included studies.

## Frontal Assessment Battery


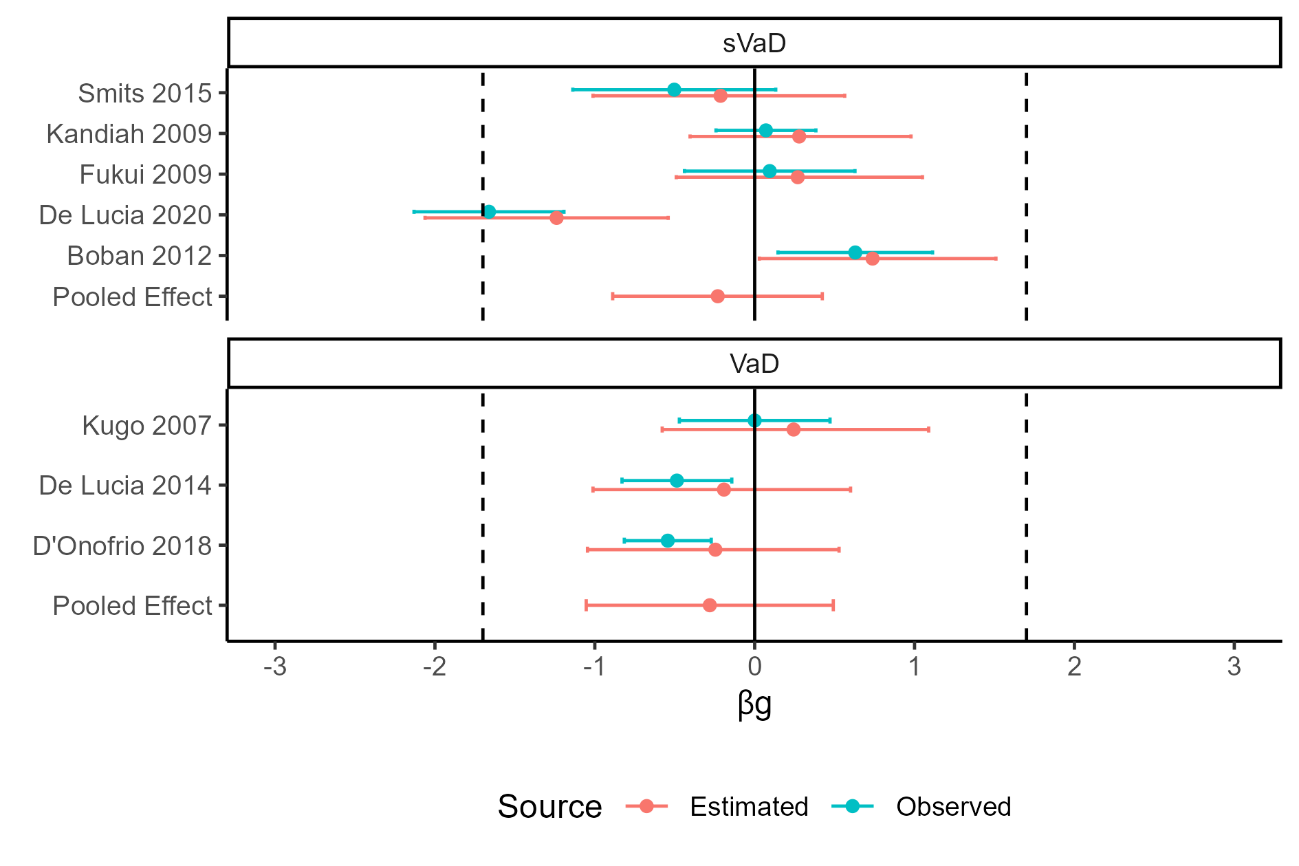


Figure 1. Forest plot for the Frontal Assessment Battery: Total Score. Regression coefficients and 95% confidence intervals are displayed. Dashed vertical lines show the lower and upper bound of the Region of Practical Equivalence set at ±1.7 *g*. Estimated effect sizes are regression coefficient estimates and Observed effects are the effect sizes and confidence intervals from the included studies.

## Stroop – Interference Condition


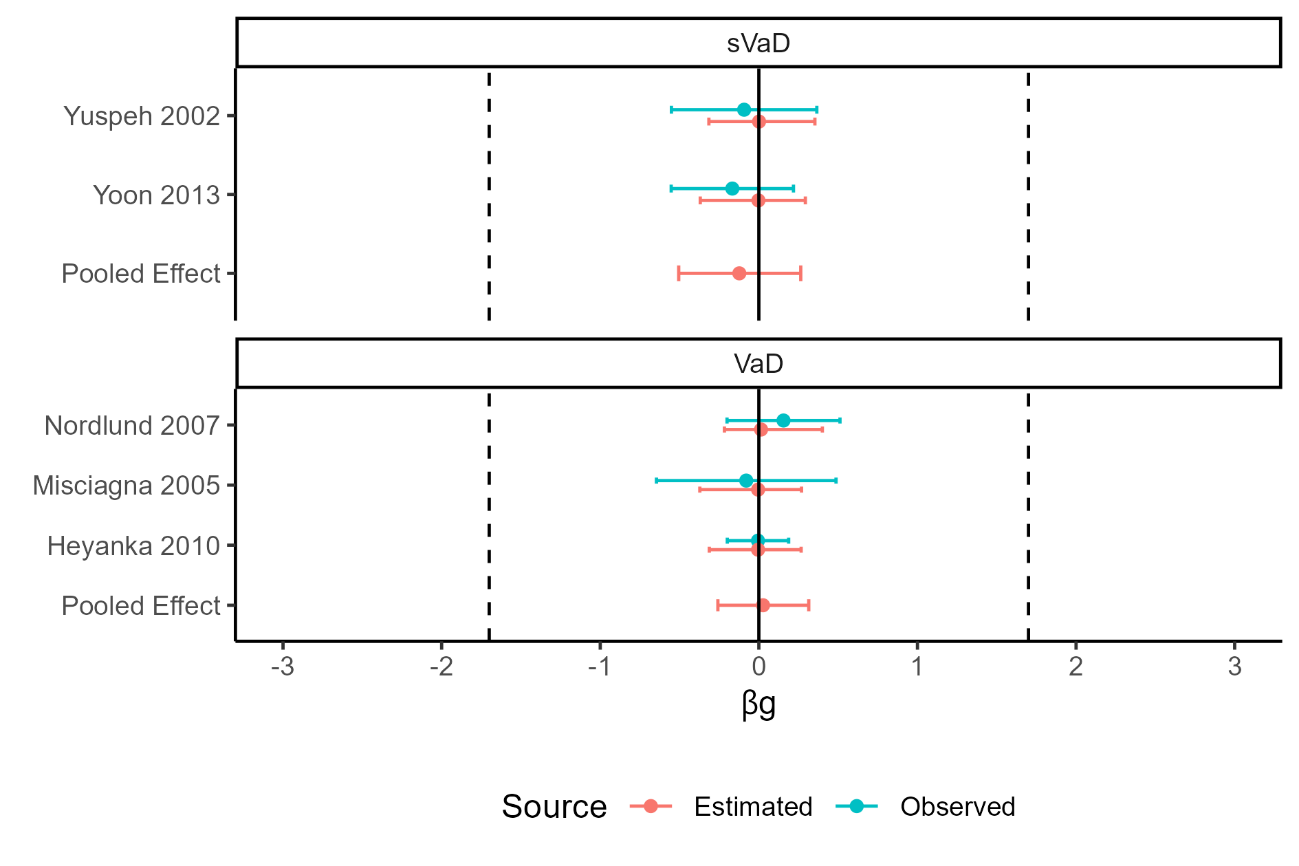


Figure 1. Forest plot for the Stroop Test: Interference Condition. Regression coefficients and 95% confidence intervals are displayed. Dashed vertical lines show the lower and upper bound of the Region of Practical Equivalence set at ±1.7 *g*. Estimated effect sizes are regression coefficient estimates and Observed effects are the effect sizes and confidence intervals from the included studies.

## Wechsler Memory Scale


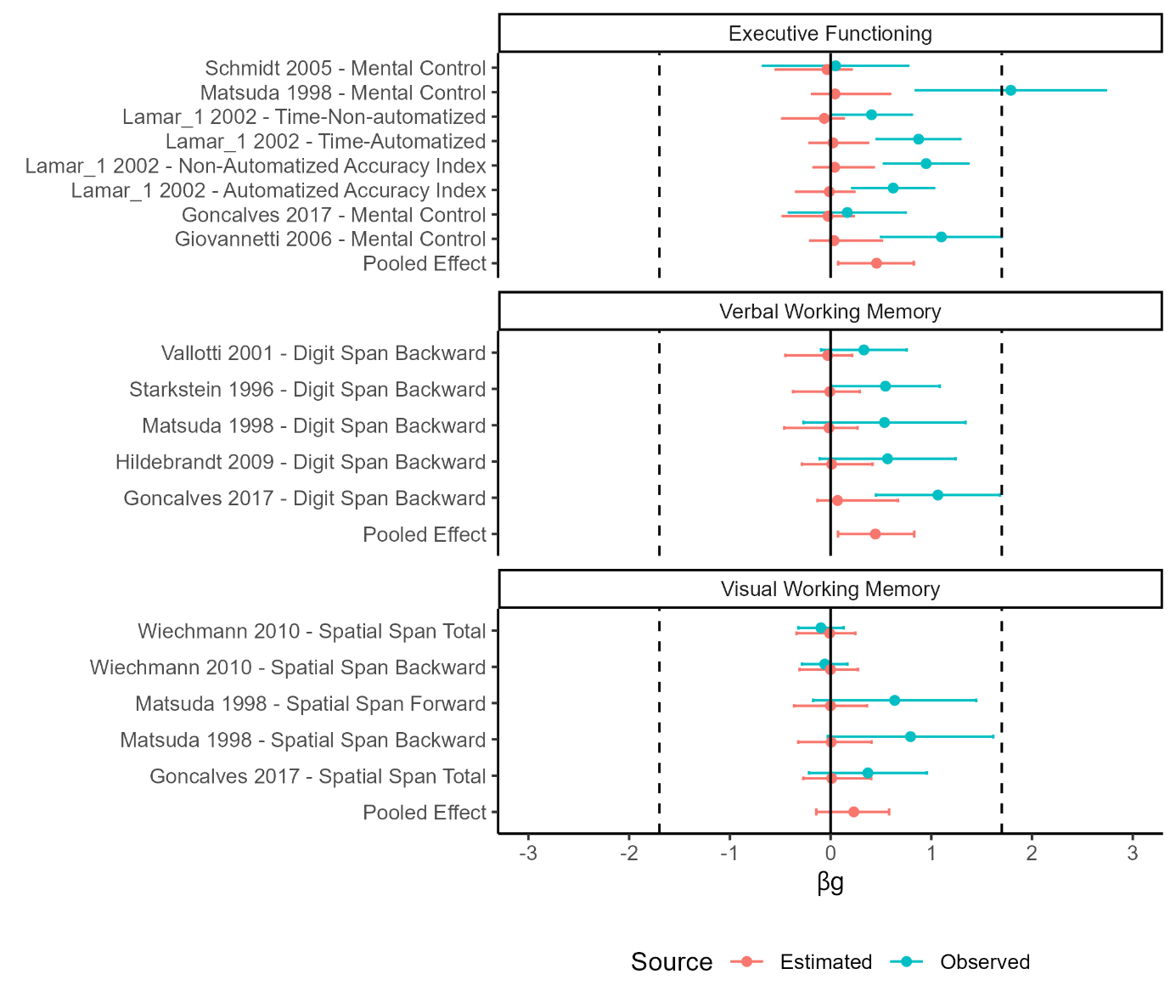


Figure 1. Forest plot for the Wechsler Memory Scale: executive function measures. Regression coefficients and 95% confidence intervals are displayed. Dashed vertical lines show the lower and upper bound of the Region of Practical Equivalence set at ±1.7 *g*. Estimated effect sizes are regression coefficient estimates and Observed effects are the effect sizes and confidence intervals from the included studies.

## Digit Span Backwards


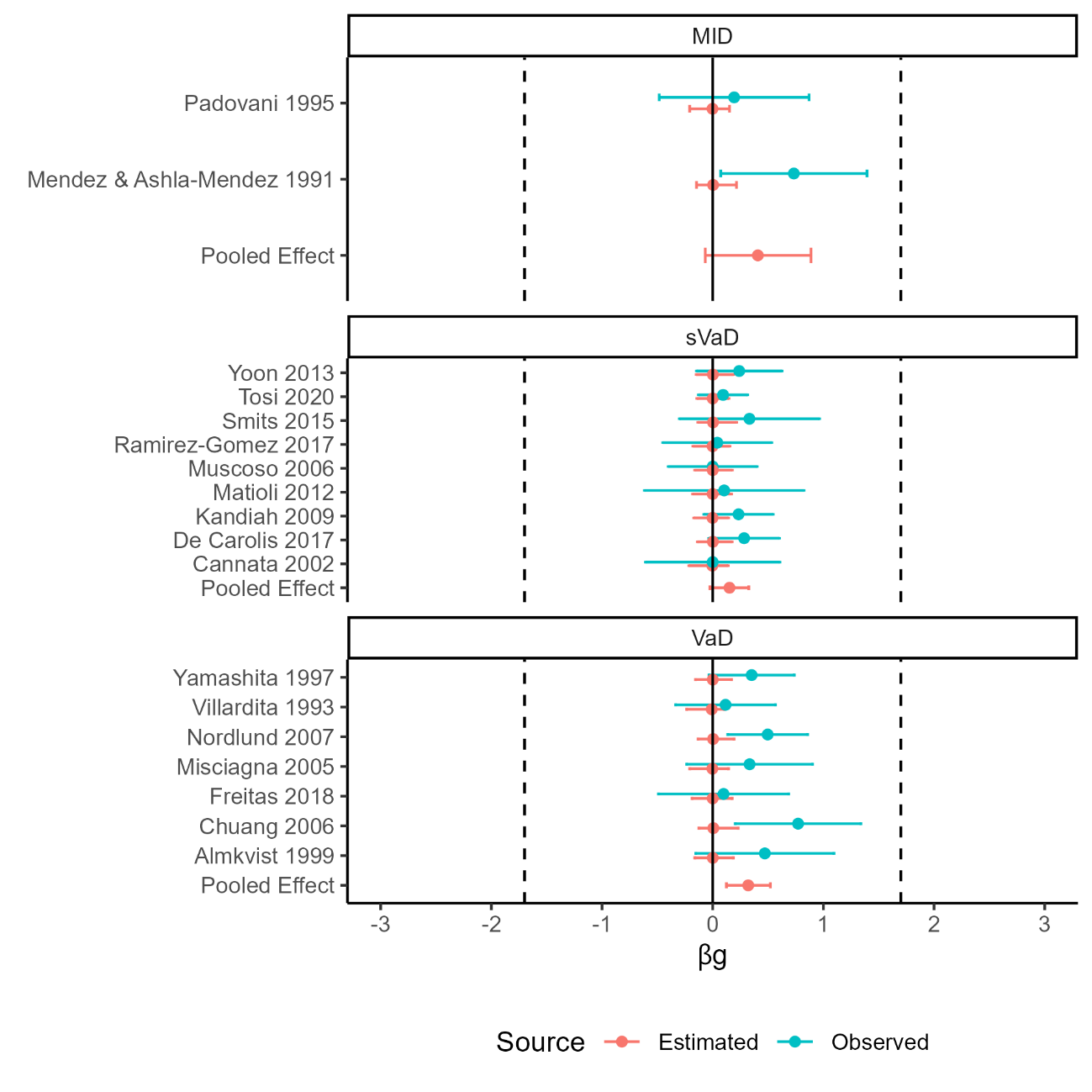


Figure 1. Forest plot for the Digit Span Backwards. Regression coefficients and 95% confidence intervals are displayed. Dashed vertical lines show the lower and upper bound of the Region of Practical Equivalence set at ±1.7 *g*. Estimated effect sizes are regression coefficient estimates and Observed effects are the effect sizes and confidence intervals from the included studies.

## Visual Span


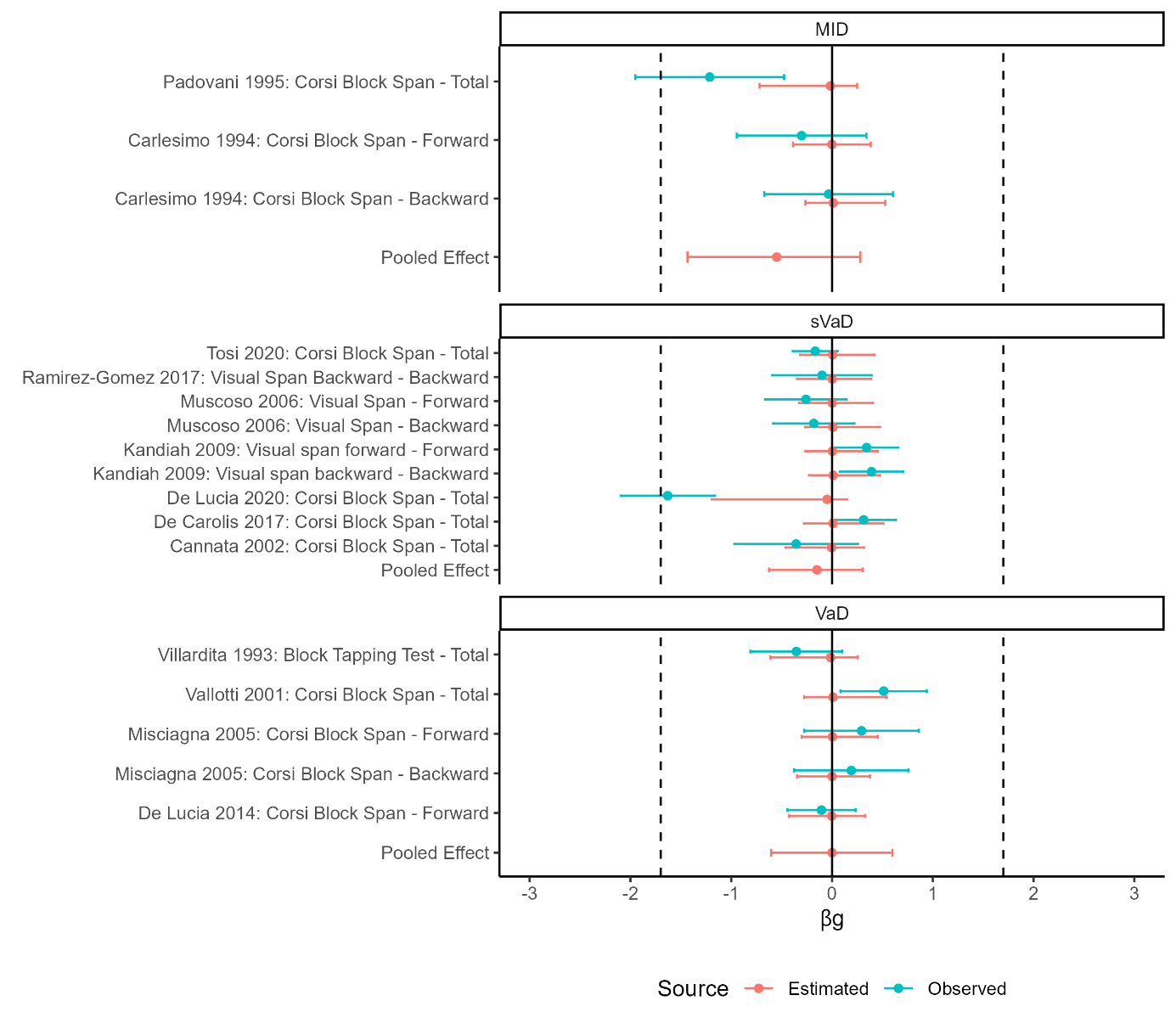


Figure 1. Forest plot for the measures of Visual Span. Regression coefficients and 95% confidence intervals are displayed. Dashed vertical lines show the lower and upper bound of the Region of Practical Equivalence set at ±1.7 *g*. Estimated effect sizes are regression coefficient estimates and Observed effects are the effect sizes and confidence intervals from the included studies.

## Maze Tasks


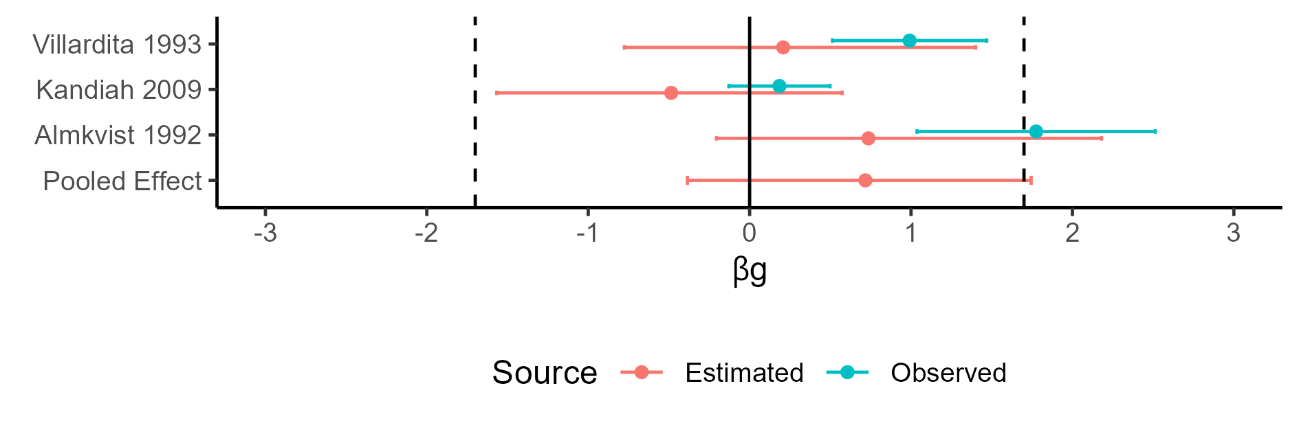


Figure 1. Forest plot for the Maze Tasks. Regression coefficients and 95% confidence intervals are displayed. Dashed vertical lines show the lower and upper bound of the Region of Practical Equivalence set at ±1.7 *g*. Estimated effect sizes are regression coefficient estimates and Observed effects are the effect sizes and confidence intervals from the included studies.

## Graphical Sequence Test


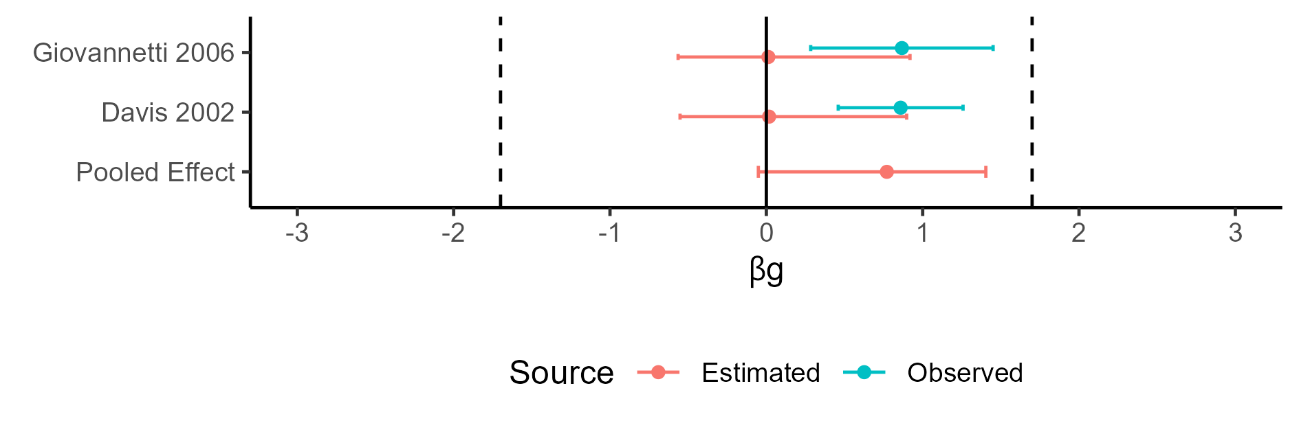


Figure 1. Forest plot for the Graphical Sequence Test. Regression coefficients and 95% confidence intervals are displayed. Dashed vertical lines show the lower and upper bound of the Region of Practical Equivalence set at ±1.7 *g*. Estimated effect sizes are regression coefficient estimates and Observed effects are the effect sizes and confidence intervals from the included studies.

## Repetition of Words and Sentences


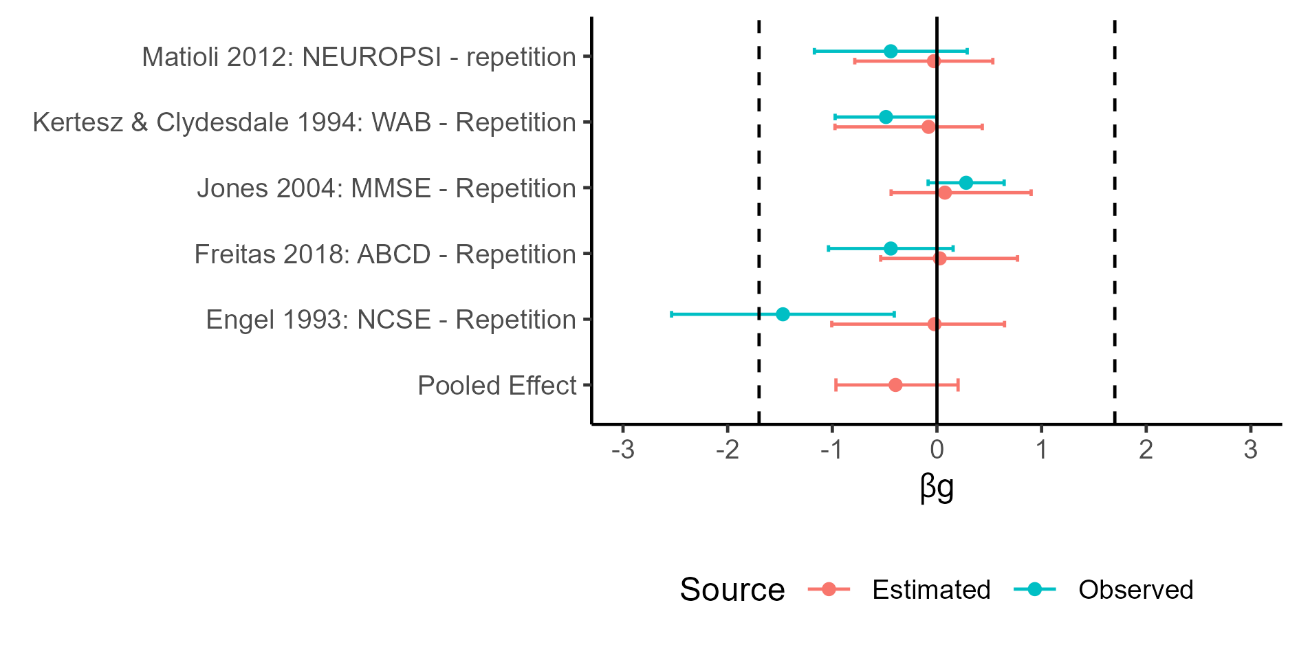


Figure 1. Forest plot for the tests of Repetition of Words and Sentences. Regression coefficients and 95% confidence intervals are displayed. Dashed vertical lines show the lower and upper bound of the Region of Practical Equivalence set at ±1.7 *g*. Estimated effect sizes are regression coefficient estimates and Observed effects are the effect sizes and confidence intervals from the included studies. ABCD: Arizona Battery for Communication Disorders of Dementia; MMSE: Mini-Mental-State-Examination; NCSE: Neurobehavior Cognitive Status Examination; WAB: Western Aphasia Battery.

## Arithmetic


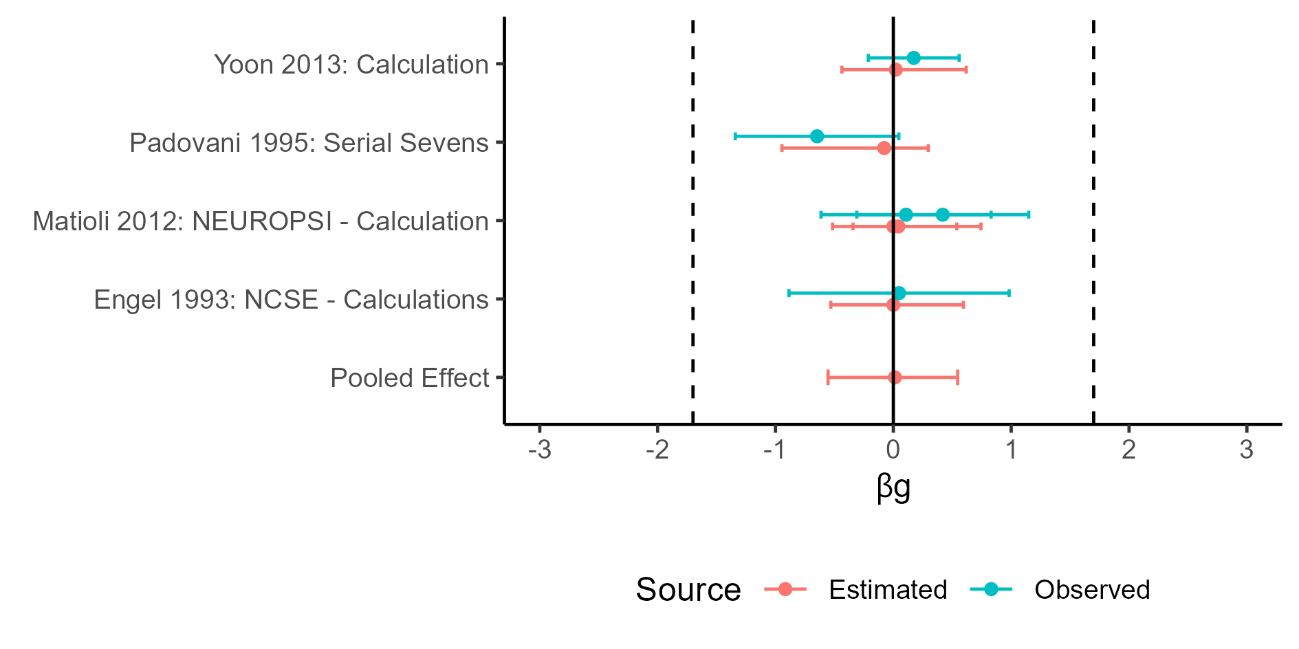


Figure 1. Forest plot for Arithmetic tests. Regression coefficients and 95% confidence intervals are displayed. Dashed vertical lines show the lower and upper bound of the Region of Practical Equivalence set at ±1.7 *g*. Estimated effect sizes are regression coefficient estimates and Observed effects are the effect sizes and confidence intervals from the included studies. NCSE: Neurobehavior Cognitive Status Examination.

## Cognitive Control of Memory


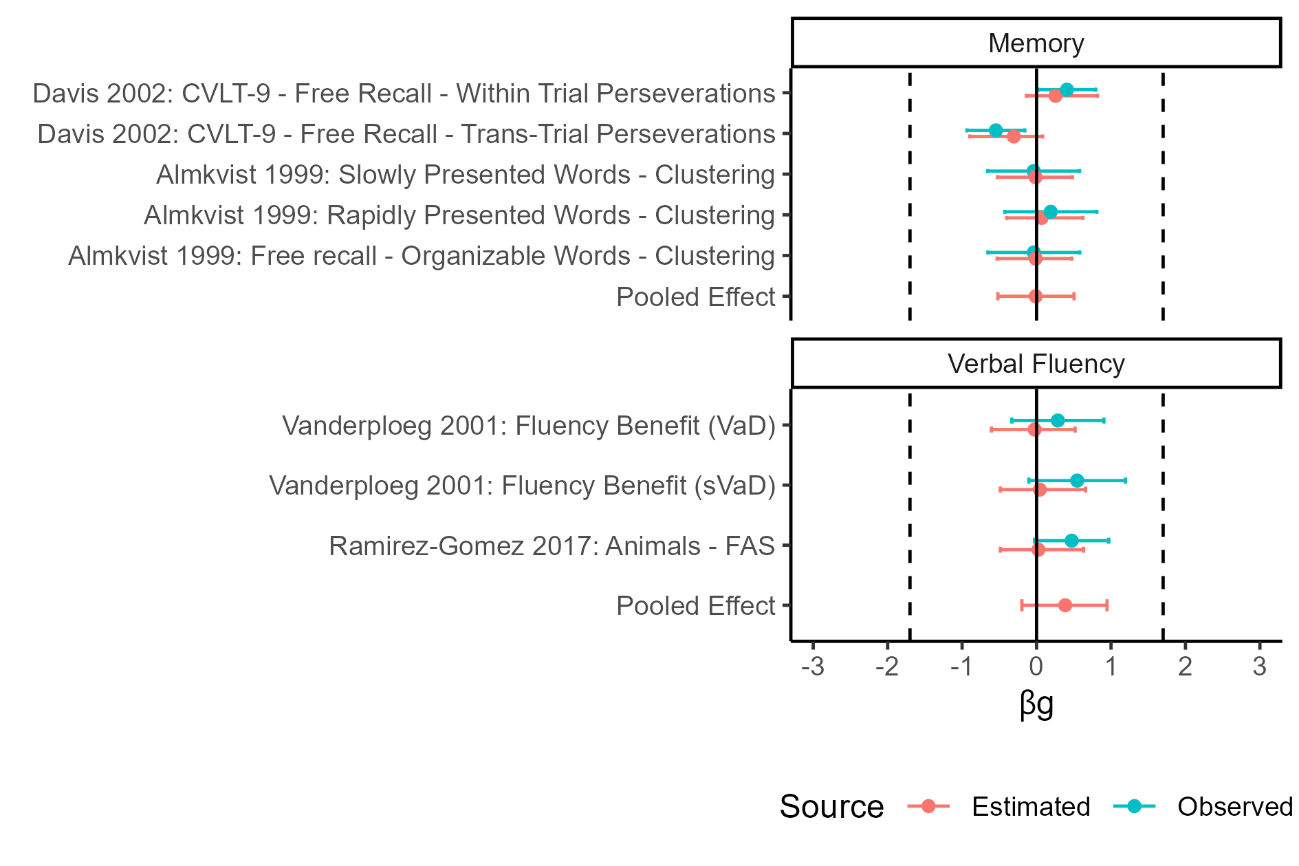


Figure 1. Forest plot for the measures of Cognitive Control of Memory. Regression coefficients and 95% confidence intervals are displayed. Dashed vertical lines show the lower and upper bound of the Region of Practical Equivalence set at ±1.7 *g*. Estimated effect sizes are regression coefficient estimates and Observed effects are the effect sizes and confidence intervals from the included studies. CVLT-9: California Verbal Learning Test short version; FAS: Controlled Oral Word Association Test phonemic fluency total score; sVaD: subcortical vascular dementia; VaD: vascular dementia.

## Cognitive Flexibility


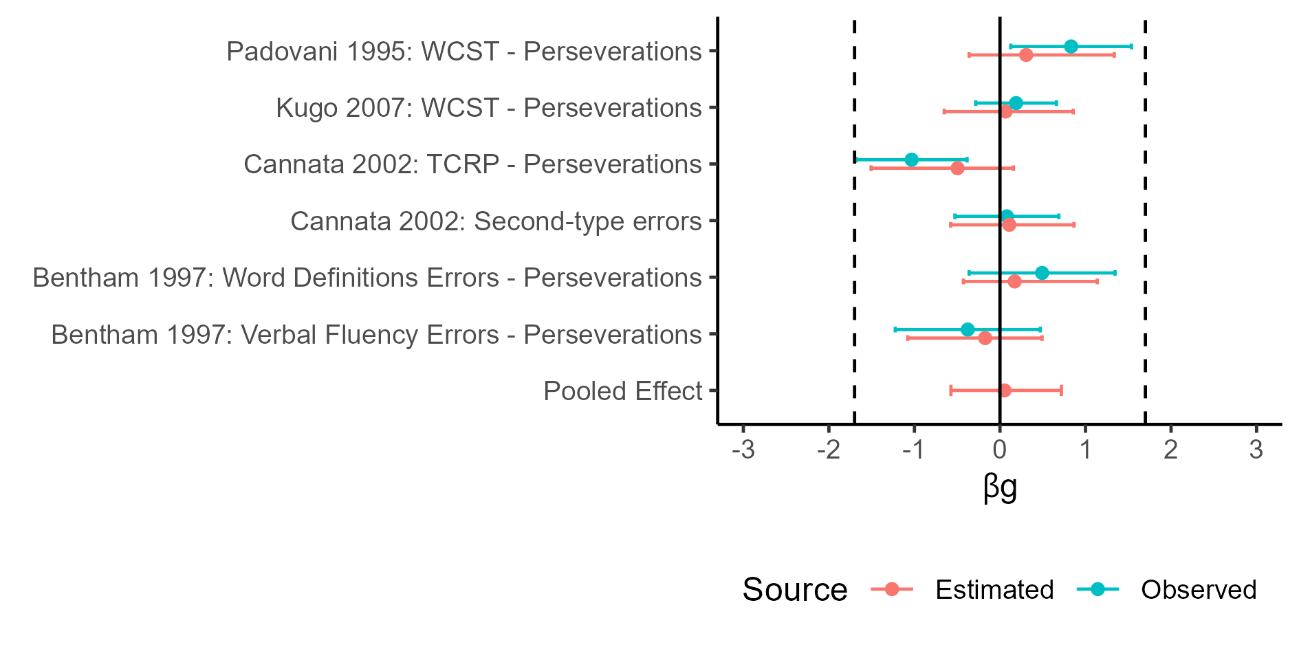


Figure 1. Forest plot for the measures of Cognitive Flexibility. Regression coefficients and 95% confidence intervals are displayed. Dashed vertical lines show the lower and upper bound of the Region of Practical Equivalence set at ±1.7 *g*. Estimated effect sizes are regression coefficient estimates and Observed effects are the effect sizes and confidence intervals from the included studies. TCRP: Test of Classification and Recall of Pictures; WCST: Wisconsin Card Sorting Test.

## Cognitive Estimation


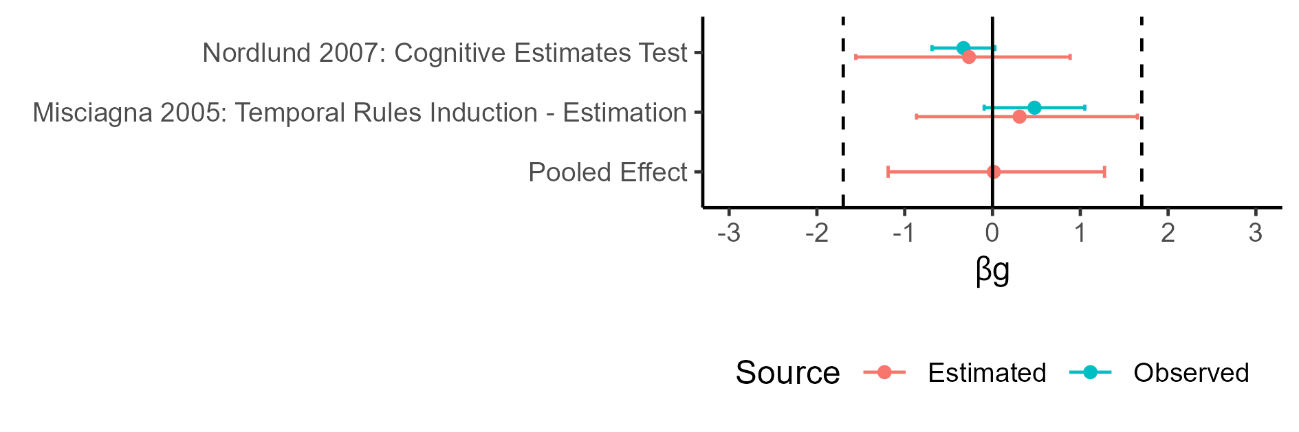


Figure 1. Forest plot for the measures of Cognitive Estimation. Regression coefficients and 95% confidence intervals are displayed. Dashed vertical lines show the lower and upper bound of the Region of Practical Equivalence set at ±1.7 *g*. Estimated effect sizes are regression coefficient estimates and Observed effects are the effect sizes and confidence intervals from the included studies.

## Global Measures of Executive Functioning


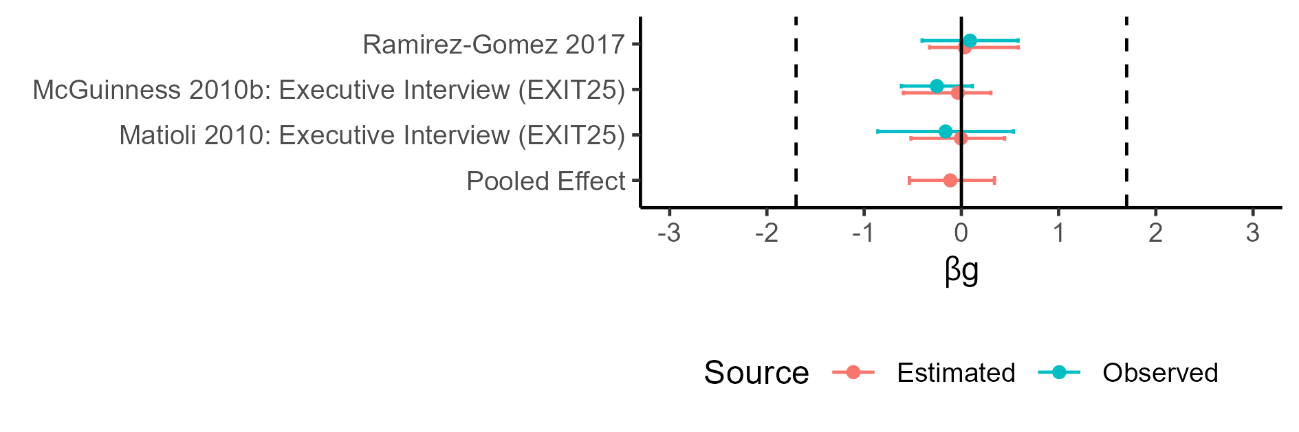


Figure 1. Forest plot for Global Measures of Executive Functioning. Regression coefficients and 95% confidence intervals are displayed. Dashed vertical lines show the lower and upper bound of the Region of Practical Equivalence set at ±1.7 *g*. Estimated effect sizes are regression coefficient estimates and Observed effects are the effect sizes and confidence intervals from the included studies.

## Set Maintenance


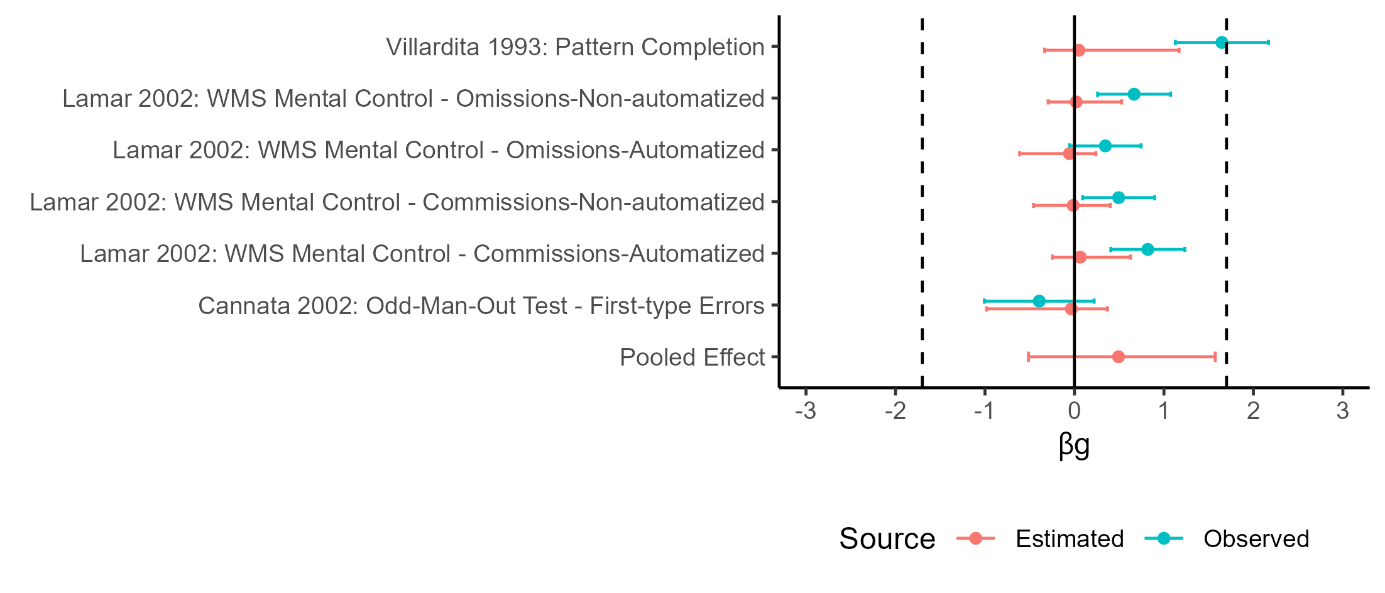


Figure 1. Forest plot for measures of Set Maintenance. Regression coefficients and 95% confidence intervals are displayed. Dashed vertical lines show the lower and upper bound of the Region of Practical Equivalence set at ±1.7 *g*. Estimated effect sizes are regression coefficient estimates and Observed effects are the effect sizes and confidence intervals from the included studies. WMS: Wechsler Memory Scale.

## Other Measures of Verbal Working Memory


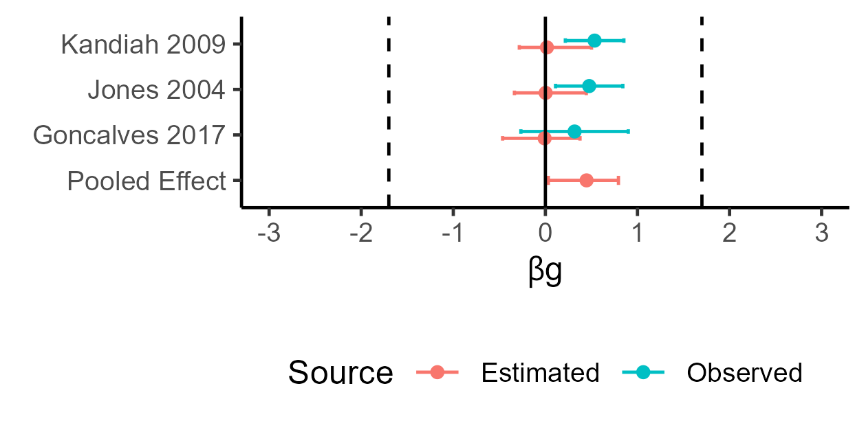


Figure 1. Forest plot for Other Measures of Verbal Working Memory. Regression coefficients and 95% confidence intervals are displayed. Dashed vertical lines show the lower and upper bound of the Region of Practical Equivalence set at ±1.7 *g*. Estimated effect sizes are regression coefficient estimates and Observed effects are the effect sizes and confidence intervals from the included studies.

## Other Measures of Visual Working Memory


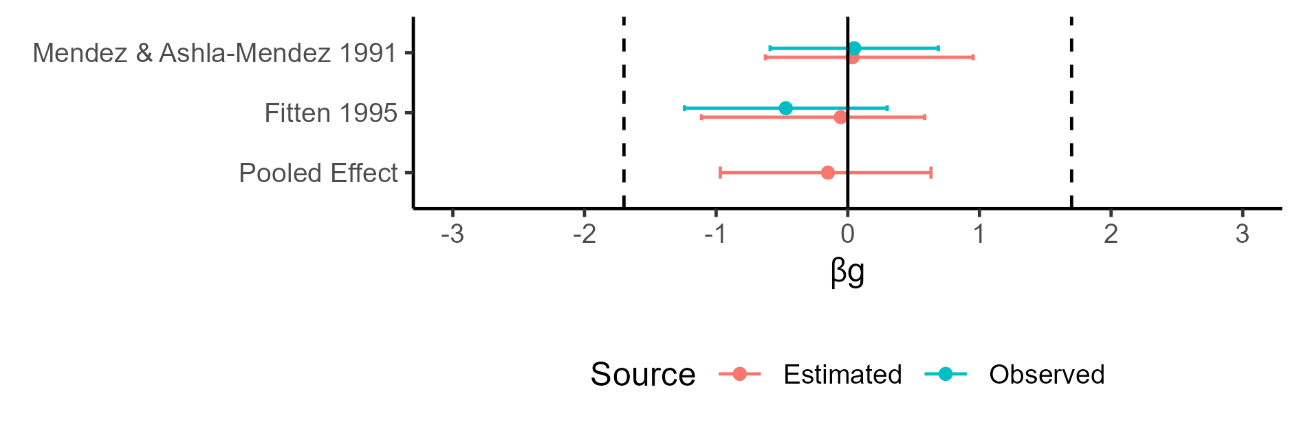


Figure 1. Forest plot for Other Measures of Visual Working Memory. Regression coefficients and 95% confidence intervals are displayed. Dashed vertical lines show the lower and upper bound of the Region of Practical Equivalence set at ±1.7 *g*. Estimated effect sizes are regression coefficient estimates and Observed effects are the effect sizes and confidence intervals from the included studies.

## Sequencing


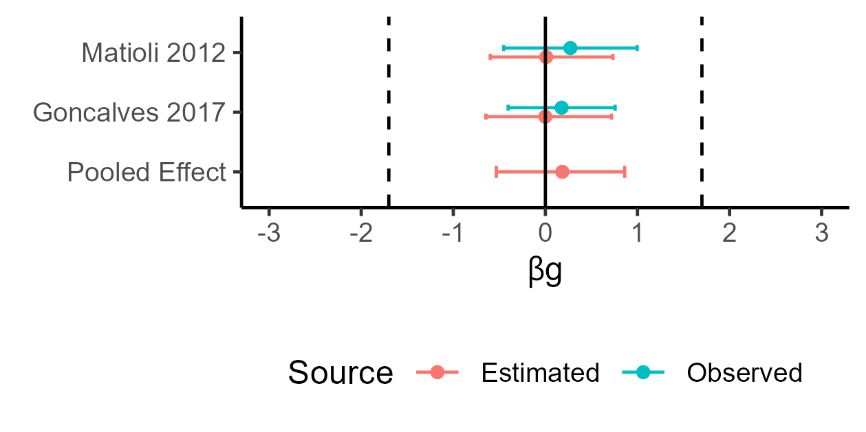


Figure 1. Forest plot for Sequencing measures. Regression coefficients and 95% confidence intervals are displayed. Dashed vertical lines show the lower and upper bound of the Region of Practical Equivalence set at ±1.7 *g*. Estimated effect sizes are regression coefficient estimates and Observed effects are the effect sizes and confidence intervals from the included studies.

## Benton Visual Retention Test


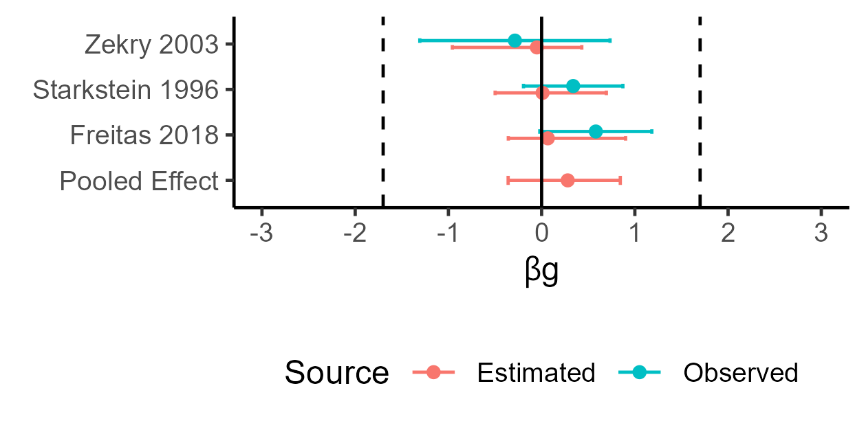


Figure 1. Forest plot for the Benton Visual Retention Test. Regression coefficients and 95% confidence intervals are displayed. Dashed vertical lines show the lower and upper bound of the Region of Practical Equivalence set at ±1.7 *g*. Estimated effect sizes are regression coefficient estimates and Observed effects are the effect sizes and confidence intervals from the included studies.

## Quality Sensitivity Analyses

### Digit Span Backwards


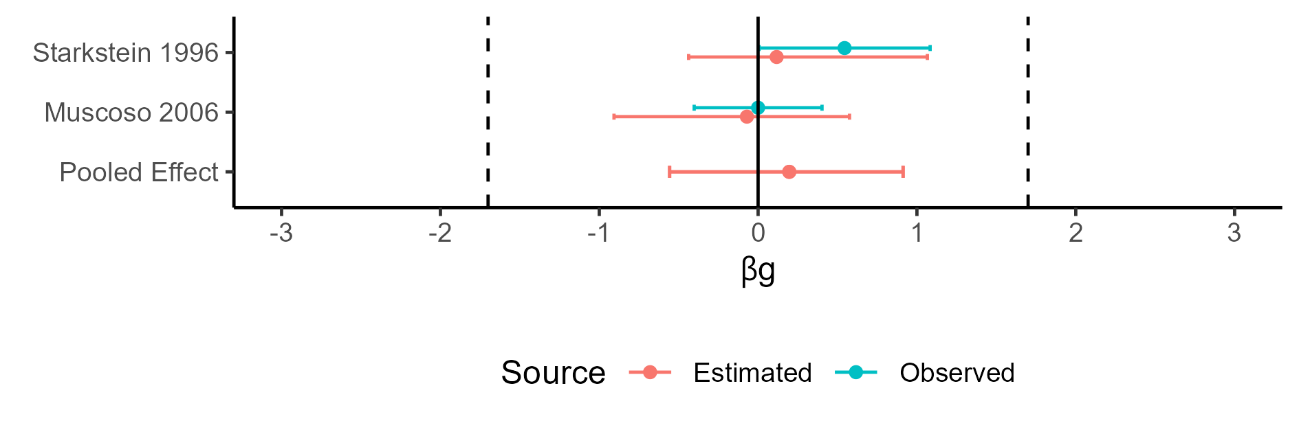


Figure 1. Forest plot of the study quality sensitivity analysis for the Digit Span Backwards. Regression coefficients and 95% confidence intervals are displayed. Dashed vertical lines show the lower and upper bound of the Region of Practical Equivalence set at ±1.7 *g*. Estimated effect sizes are regression coefficient estimates and Observed effects are the effect sizes and confidence intervals from the included studies.

### Visual Working Memory


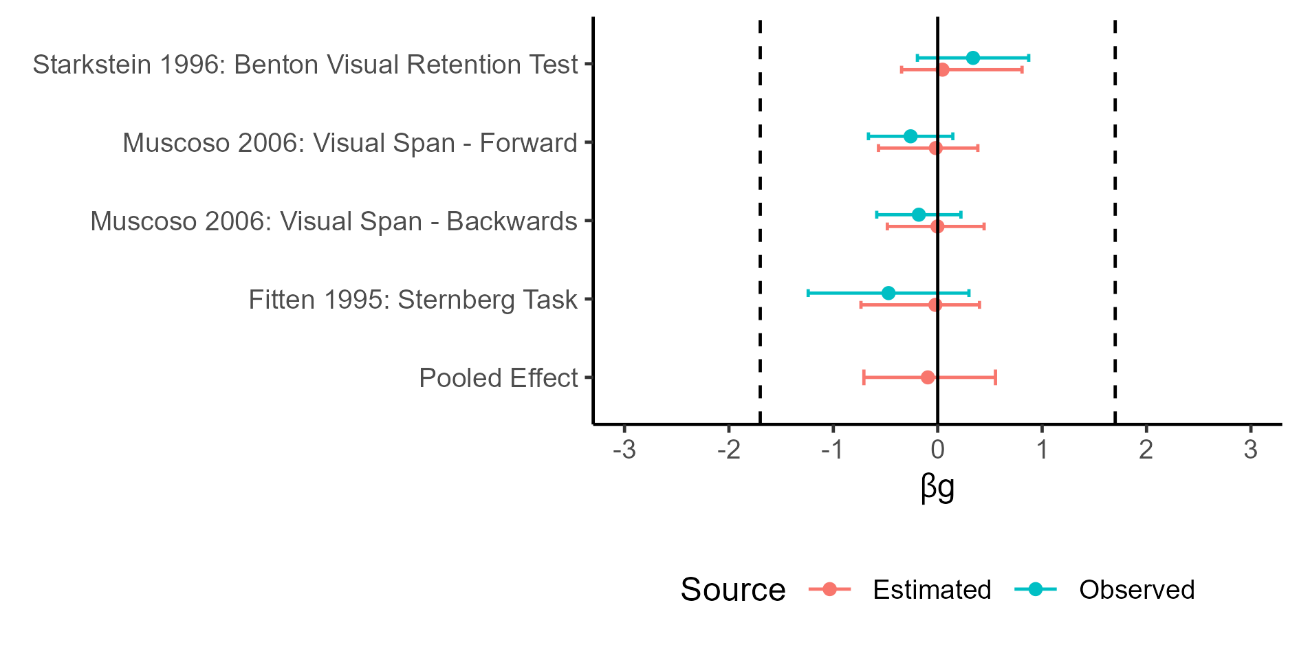


Figure 1. Forest plot of the study quality sensitivity analysis for the Measures of Visual Working Memory. Regression coefficients and 95% confidence intervals are displayed. Dashed vertical lines show the lower and upper bound of the Region of Practical Equivalence set at ±1.7 *g*. Estimated effect sizes are regression coefficient estimates and Observed effects are the effect sizes and confidence intervals from the included studies.

### Cognitive Flexibility


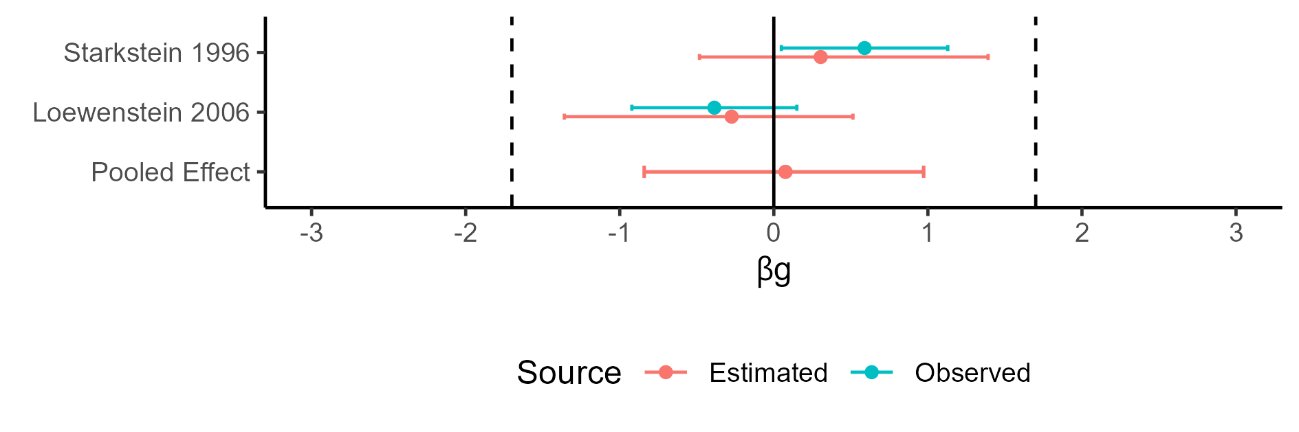


Figure 1. Forest plot of the study quality sensitivity analysis for measures of Cognitive Flexibility. Regression coefficients and 95% confidence intervals are displayed. Dashed vertical lines show the lower and upper bound of the Region of Practical Equivalence set at ±1.7 *g*. Estimated effect sizes are regression coefficient estimates and Observed effects are the effect sizes and confidence intervals from the included studies.

# Memory

## Wechsler Memory Scale: Verbal


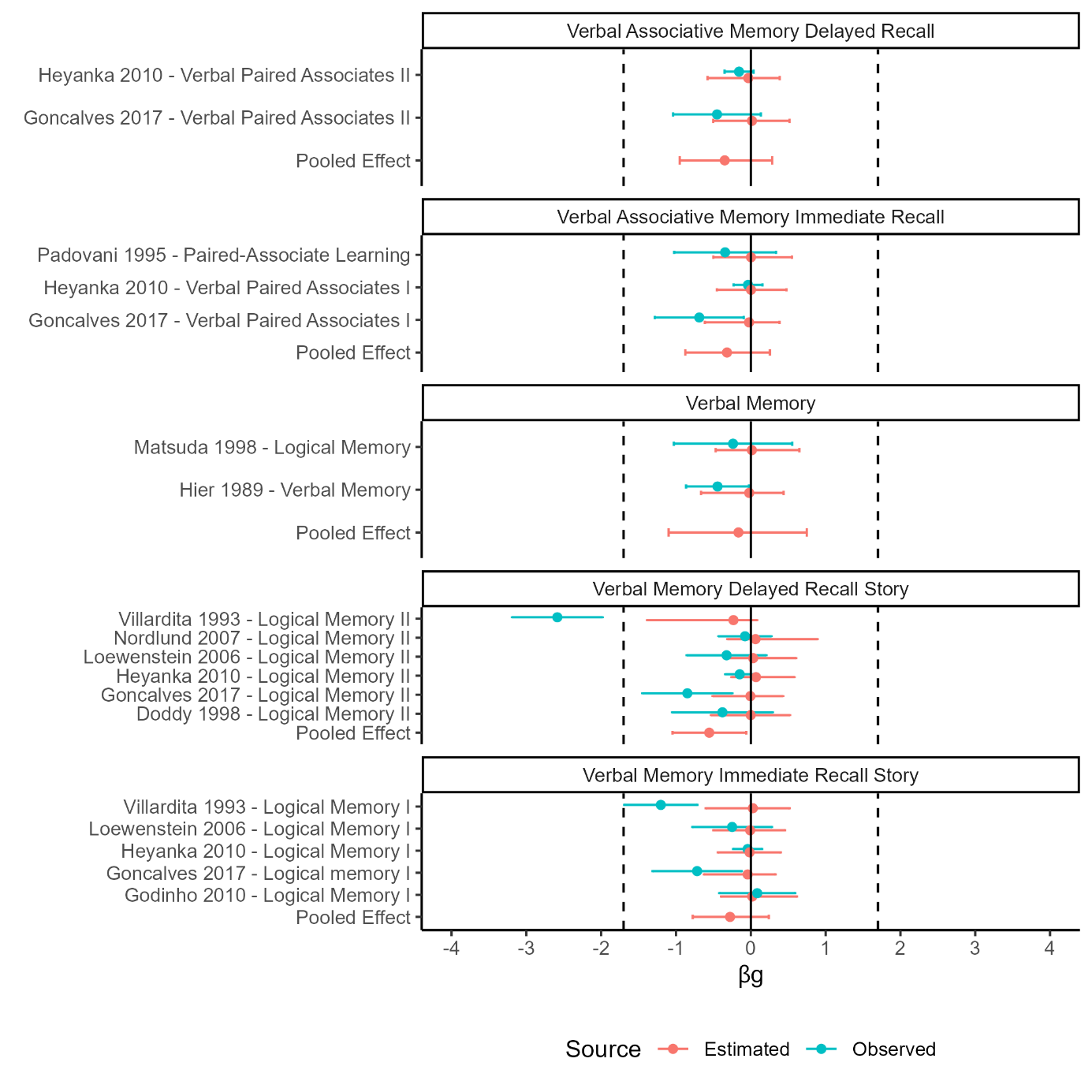


Figure 1. Forest plot for the Weschler Memory Scale: Memory Subtests. Regression coefficients and 95% confidence intervals are displayed. Dashed vertical lines show the lower and upper bound of the Region of Practical Equivalence set at ±1.7 *g*. Estimated effect sizes are regression coefficient estimates and Observed effects are the effect sizes and confidence intervals from the included studies.

## Rey’s Auditory Verbal Learning Test


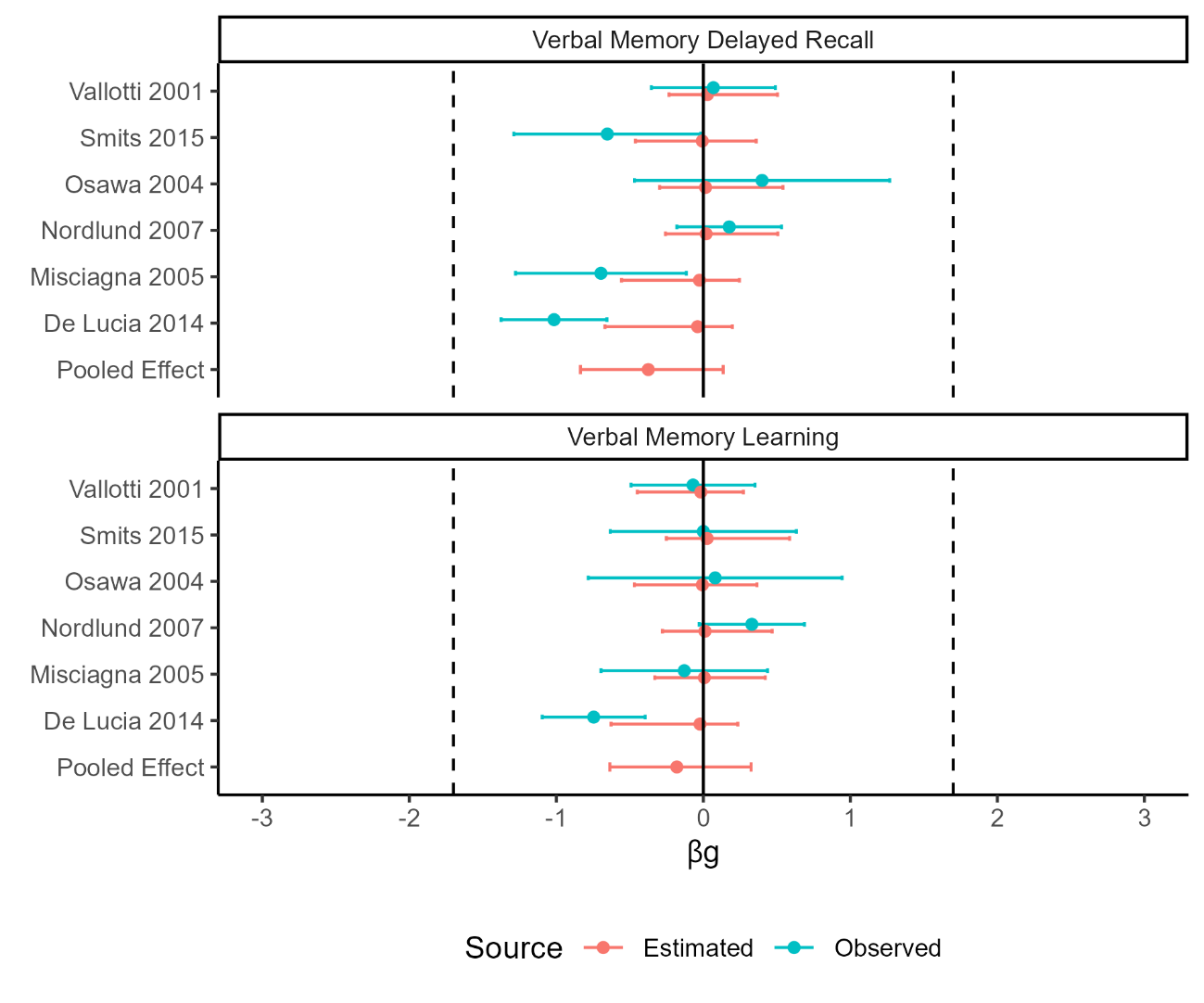


Figure 1. Forest plot for the Rey’ Auditory Verbal Learning Test. Regression coefficients and 95% confidence intervals are displayed. Dashed vertical lines show the lower and upper bound of the Region of Practical Equivalence set at ±1.7 *g*. Estimated effect sizes are regression coefficient estimates and Observed effects are the effect sizes and confidence intervals from the included studies.

## California Verbal Learning Test


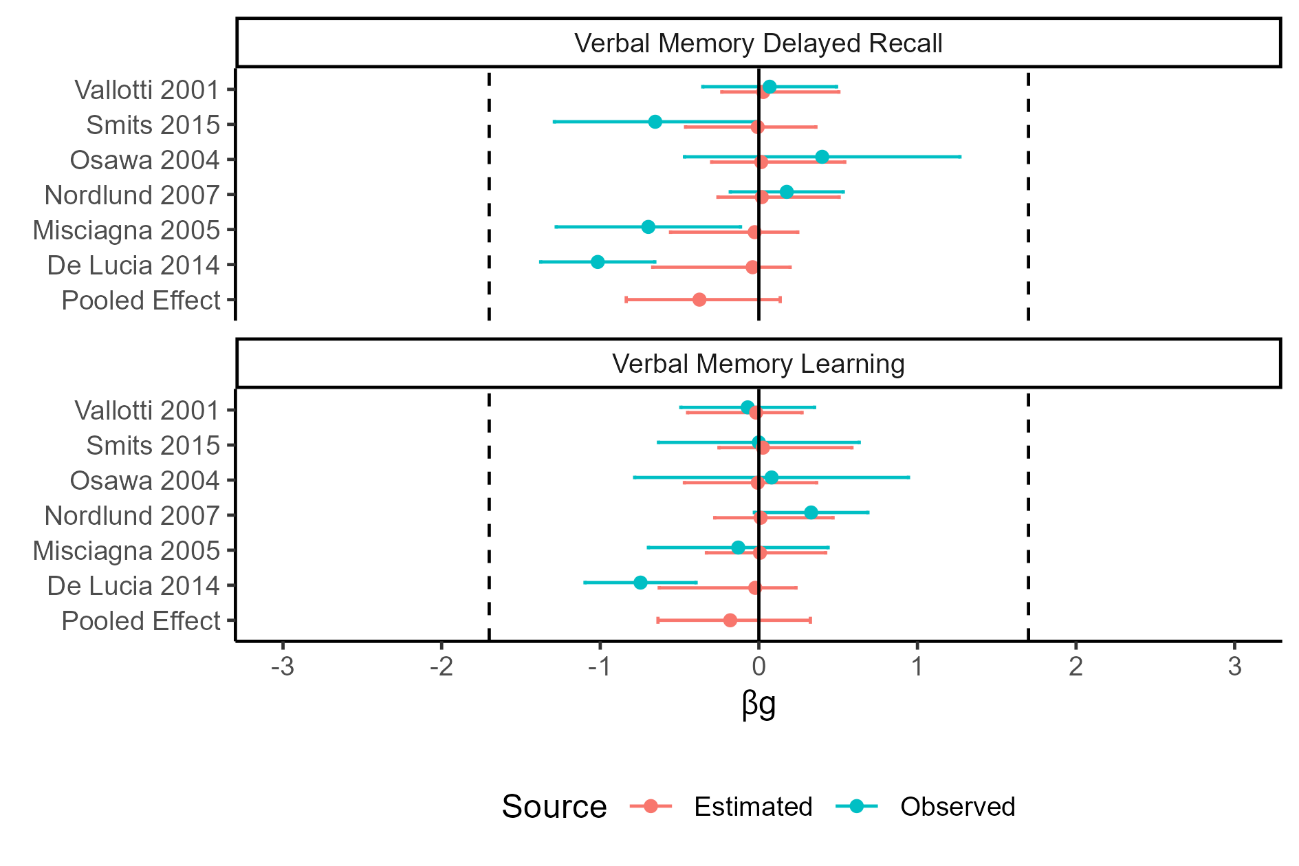


Figure 1. Forest plot for the California Verbal Learning Test. Regression coefficients and 95% confidence intervals are displayed. Dashed vertical lines show the lower and upper bound of the Region of Practical Equivalence set at ±1.7 *g*. Estimated effect sizes are regression coefficient estimates and Observed effects are the effect sizes and confidence intervals from the included studies.

## CERAD Word List


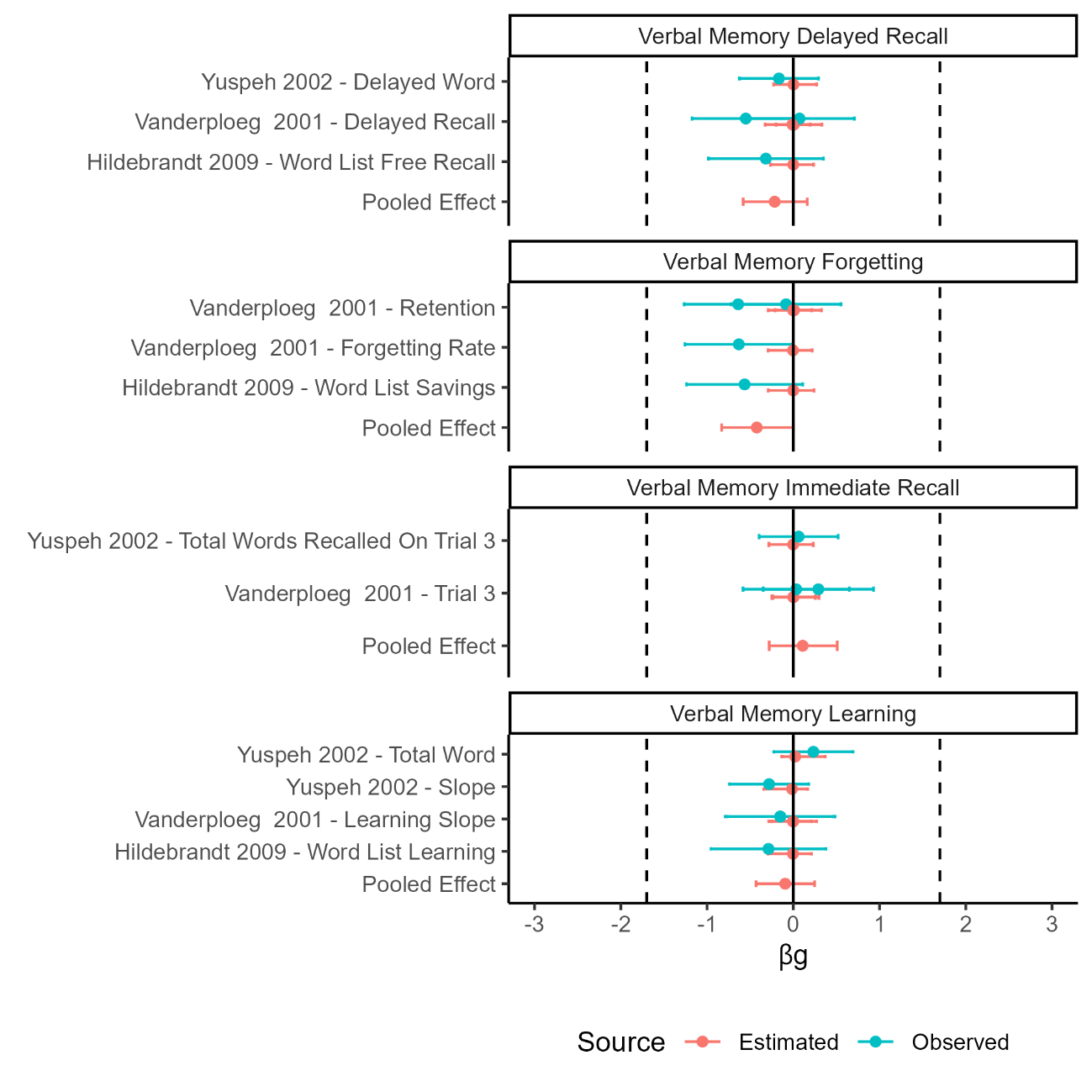


Figure 1. Forest plot for the Consortium to Establish a Registry for Alzheimer’s Disease (CERAD): Word List. Regression coefficients and 95% confidence intervals are displayed. Dashed vertical lines show the lower and upper bound of the Region of Practical Equivalence set at ±1.7 *g*. Estimated effect sizes are regression coefficient estimates and Observed effects are the effect sizes and confidence intervals from the included studies.

## Hopkin’s Verbal Learning Test


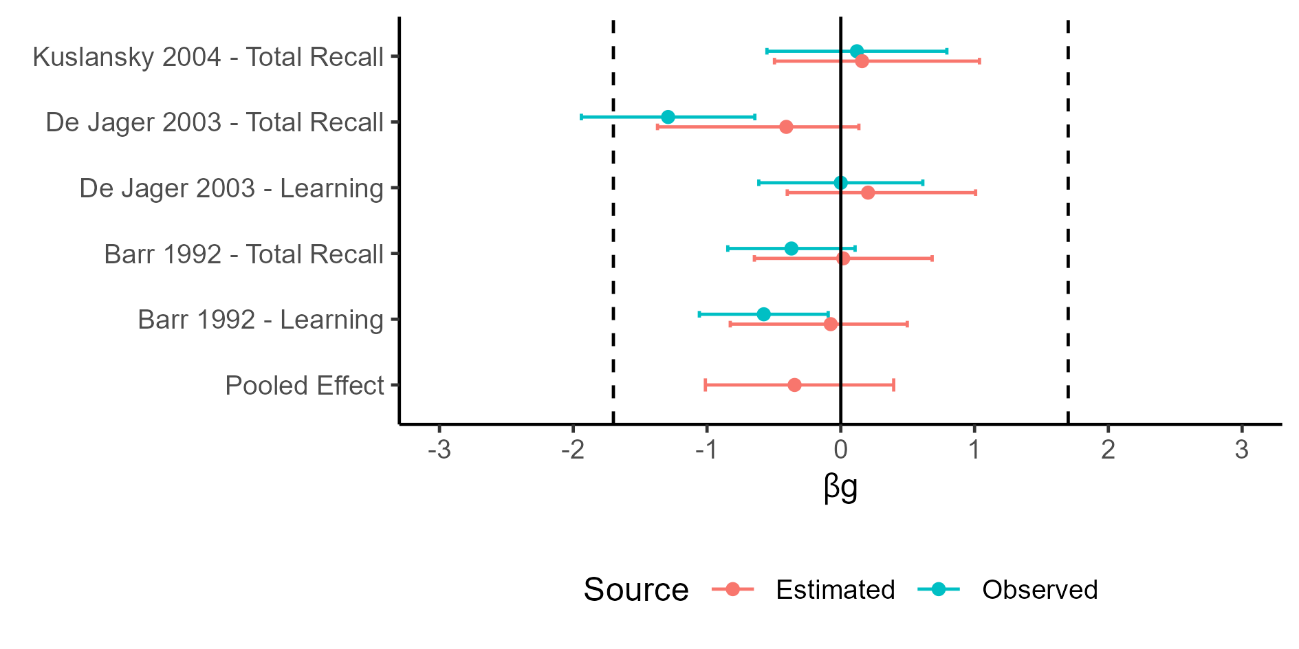


Figure 1. Forest plot for the Hopkin’s Verbal Learning Test. Regression coefficients and 95% confidence intervals are displayed. Dashed vertical lines show the lower and upper bound of the Region of Practical Equivalence set at ±1.7 *g*. Estimated effect sizes are regression coefficient estimates and Observed effects are the effect sizes and confidence intervals from the included studies.

## Addenbrooke’s Cognitive Examination


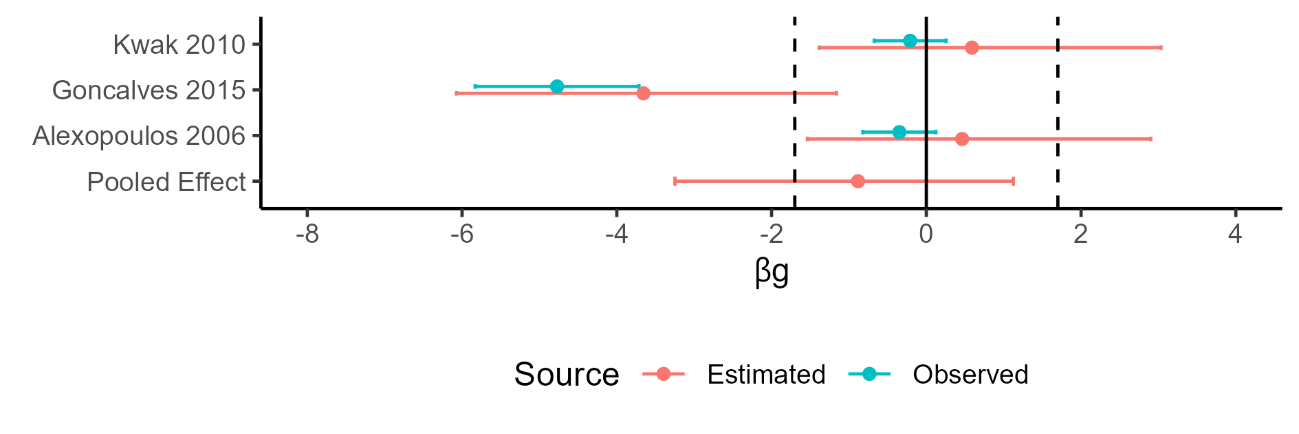


Figure 1. Forest plot for the Addenbrooke’s Cognitive Examination: Memory subscale. Regression coefficients and 95% confidence intervals are displayed. Dashed vertical lines show the lower and upper bound of the Region of Practical Equivalence set at ±1.7 *g*. Estimated effect sizes are regression coefficient estimates and Observed effects are the effect sizes and confidence intervals from the included studies.

## Fuld Object Memory Examination


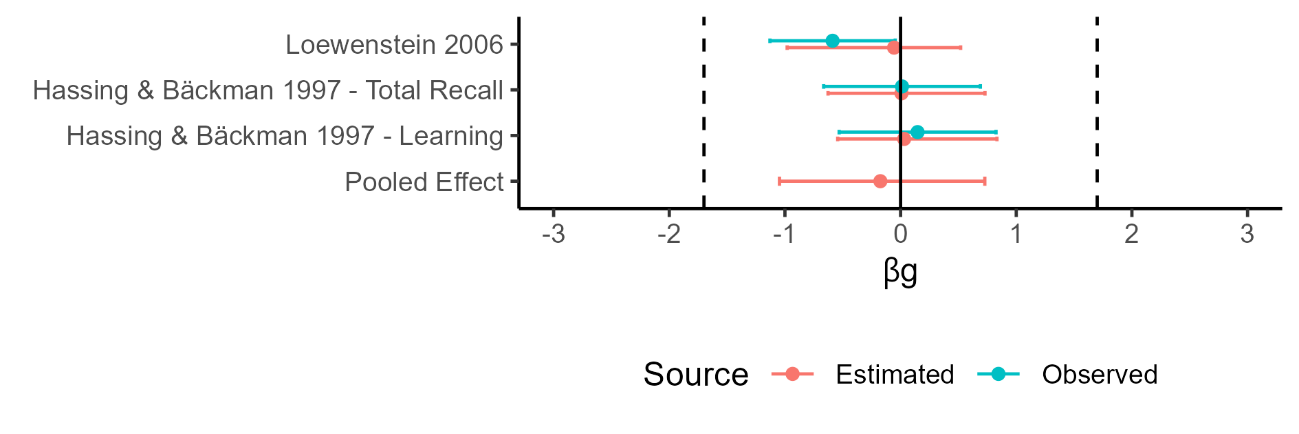


Figure 1. Forest plot for the Fuld Object Memory Examination. Regression coefficients and 95% confidence intervals are displayed. Dashed vertical lines show the lower and upper bound of the Region of Practical Equivalence set at ±1.7 *g*. Estimated effect sizes are regression coefficient estimates and Observed effects are the effect sizes and confidence intervals from the included studies.

## General Verbal Memory Measures


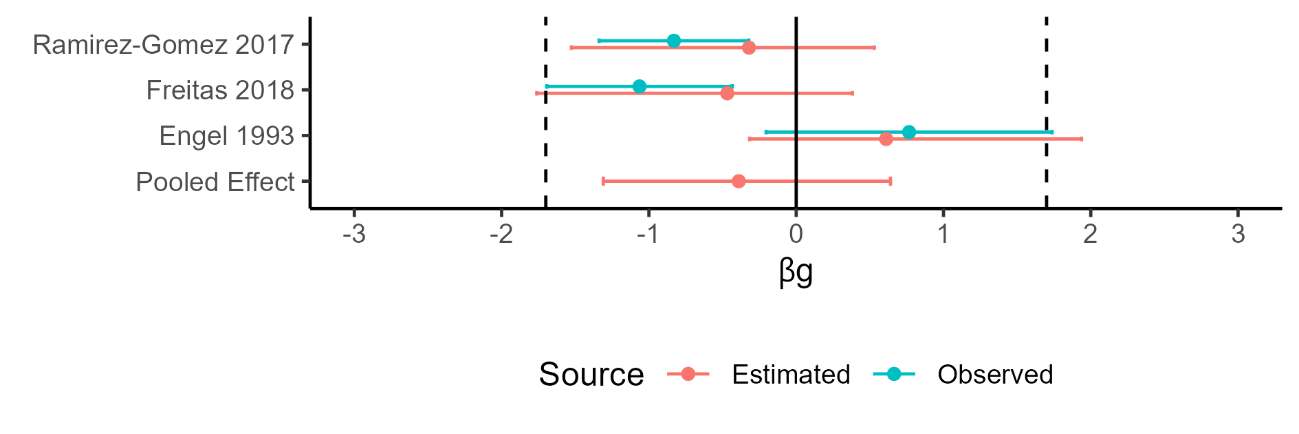


Figure 1. Forest plot for the General Verbal Memory Measures. Regression coefficients and 95% confidence intervals are displayed. Dashed vertical lines show the lower and upper bound of the Region of Practical Equivalence set at ±1.7 *g*. Estimated effect sizes are regression coefficient estimates and Observed effects are the effect sizes and confidence intervals from the included studies.

Other Measures of Verbal Associative Memory


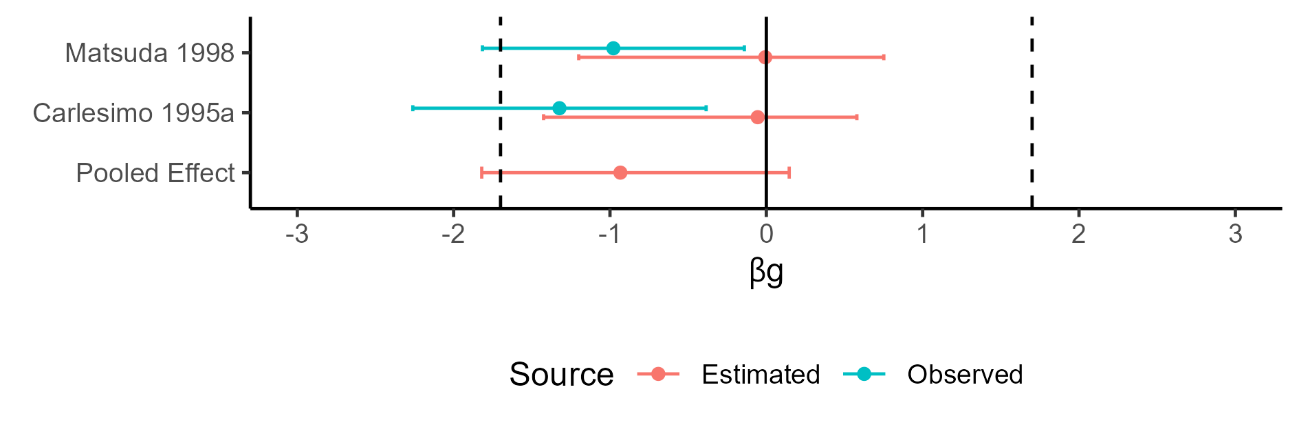


Figure 1. Forest plot for Other Measures of Verbal Associative Memory. Regression coefficients and 95% confidence intervals are displayed. Dashed vertical lines show the lower and upper bound of the Region of Practical Equivalence set at ±1.7 *g*. Estimated effect sizes are regression coefficient estimates and Observed effects are the effect sizes and confidence intervals from the included studies.

## Other Measures of Verbal Learning


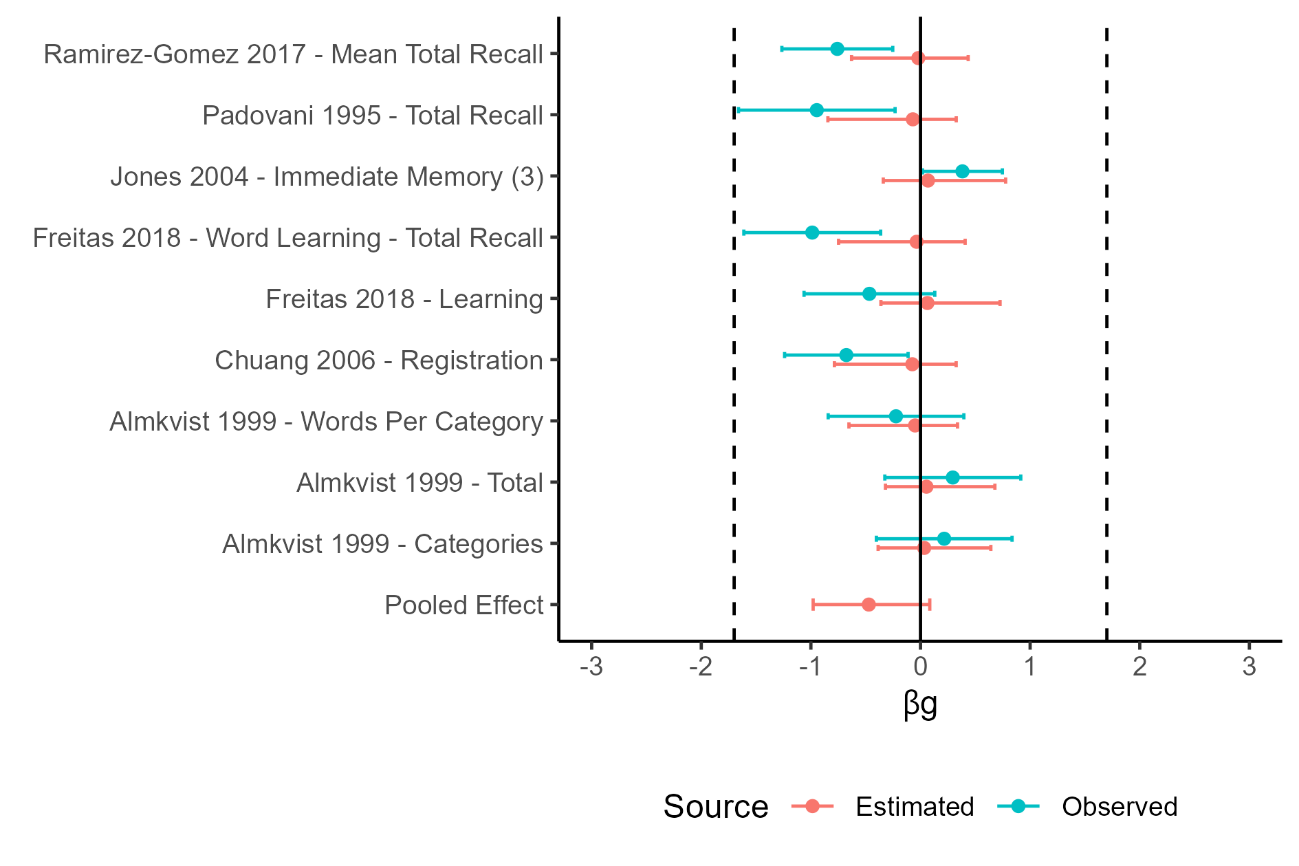


Figure 1. Forest plot for Other Measures of Verbal Learning. Regression coefficients and 95% confidence intervals are displayed. Dashed vertical lines show the lower and upper bound of the Region of Practical Equivalence set at ±1.7 *g*. Estimated effect sizes are regression coefficient estimates and Observed effects are the effect sizes and confidence intervals from the included studies.

## Other Measures of Episodic Memory: Prose

Figure 1. Forest plot for Other Measures of Episodic Memory: Prose. Regression coefficients and 95% confidence intervals are displayed. Dashed vertical lines show the lower and upper bound of the Region of Practical Equivalence set at ±1.7 *g*. Estimated effect sizes are regression coefficient estimates and Observed effects are the effect sizes and confidence intervals from the included studies. ABCD: Arizona Battery for Communication Disorders of Dementia.

## Other Measures of Episodic Memory: Word Lists

Figure 1. Forest plot for Other Measures of Episodic Memory: Word Lists. Regression coefficients and 95% confidence intervals are displayed. Dashed vertical lines show the lower and upper bound of the Region of Practical Equivalence set at ±1.7 *g*. Estimated effect sizes are regression coefficient estimates and Observed effects are the effect sizes and confidence intervals from the included studies. ABCD: Arizona Battery for Communication Disorders of Dementia; MAS: Memory Assessment Scales; MMSE: Mini-Mental-State-Examination.

## Other Measures of Cued Recall of Word Lists

Figure 1. Forest plot for Other Measures of Cued Recall of Word Lists. Regression coefficients and 95% confidence intervals are displayed. Dashed vertical lines show the lower and upper bound of the Region of Practical Equivalence set at ±1.7 *g*. Estimated effect sizes are regression coefficient estimates and Observed effects are the effect sizes and confidence intervals from the included studies. CVLT: California Verbal Learning Test; MAS: Memory Assessment Scales.

## Memory Intrusions

Figure 1. Forest plot for measures of Memory Intrusions. Regression coefficients and 95% confidence intervals are displayed. Dashed vertical lines show the lower and upper bound of the Region of Practical Equivalence set at ±1.7 *g*. Estimated effect sizes are regression coefficient estimates and Observed effects are the effect sizes and confidence intervals from the included studies. CERAD: Consortium to Establish a Registry for Alzheimer’s Disease; CVLT: California Verbal Learning Test; FR: Free Recall; HVLT: Hopkin’s Verbal Learning Test; MAS: Memory Assessment Scales.

## Semantic Memory

Figure 1. Forest plot for the measures of Semantic Memory. Regression coefficients and 95% confidence intervals are displayed. Dashed vertical lines show the lower and upper bound of the Region of Practical Equivalence set at ±1.7 *g*. Estimated effect sizes are regression coefficient estimates and Observed effects are the effect sizes and confidence intervals from the included studies. ABCD: Arizona Battery for Communication Disorders of Dementia; WAIS: Wechsler Adult Intelligence Scale.

## Global Measures of Memory

Figure 1. Forest plot for Global Measures of Memory. Regression coefficients and 95% confidence intervals are displayed. Dashed vertical lines show the lower and upper bound of the Region of Practical Equivalence set at ±1.7 *g*. Estimated effect sizes are regression coefficient estimates and Observed effects are the effect sizes and confidence intervals from the included studies.

## Quality Sensitivity Analysis: Verbal Delayed Recall

Figure 1. Forest plot of study quality sensitivity analysis for Verbal Delayed Recall of Word Lists. Regression coefficients and 95% confidence intervals are displayed. Dashed vertical lines show the lower and upper bound of the Region of Practical Equivalence set at ±1.7 *g*. Estimated effect sizes are regression coefficient estimates and Observed effects are the effect sizes and confidence intervals from the included studies.

# Visual Memory

## Wechsler Memory Scale

Figure 1. Forest plot for the Wechsler Memory Scale: Visual Memory Subtests. Regression coefficients and 95% confidence intervals are displayed. Dashed vertical lines show the lower and upper bound of the Region of Practical Equivalence set at ±1.7 *g*. Estimated effect sizes are regression coefficient estimates and Observed effects are the effect sizes and confidence intervals from the included studies.

## Rey-Osterrieth Complex Figure Test

Figure 1. Forest plot for the Rey-Osterrieth Complex Figure Test recall. Regression coefficients and 95% confidence intervals are displayed. Dashed vertical lines show the lower and upper bound of the Region of Practical Equivalence set at ±1.7 *g*. Estimated effect sizes are regression coefficient estimates and Observed effects are the effect sizes and confidence intervals from the included studies.

## Visual Associative Memory

Figure 1. Forest plot for the tests of Visual Associative Memory. Regression coefficients and 95% confidence intervals are displayed. Dashed vertical lines show the lower and upper bound of the Region of Practical Equivalence set at ±1.7 *g*. Estimated effect sizes are regression coefficient estimates and Observed effects are the effect sizes and confidence intervals from the included studies.

## Other Measures of Visual Memory

Figure 1. Forest plot for Other Measures of Visual Memory. Regression coefficients and 95% confidence intervals are displayed. Dashed vertical lines show the lower and upper bound of the Region of Practical Equivalence set at ±1.7 *g*. Estimated effect sizes are regression coefficient estimates and Observed effects are the effect sizes and confidence intervals from the included studies. CERAD-NP: Consortium to Establish a Registry for Alzheimer's Disease Neuropsychological Test Battery.

# Recognition Memory

## Wechsler Memory Scale

Figure 1. Forest plot for the Wechsler Memory Scale: Recognition Memory scores. Regression coefficients and 95% confidence intervals are displayed. Dashed vertical lines show the lower and upper bound of the Region of Practical Equivalence set at ±1.7 *g*. Estimated effect sizes are regression coefficient estimates and Observed effects are the effect sizes and confidence intervals from the included studies.

## Rey’s Auditory Verbal Learning Test

Figure 1. Forest plot for the Rey’s Auditory Verbal Learning Test: Recognition scores. Regression coefficients and 95% confidence intervals are displayed. Dashed vertical lines show the lower and upper bound of the Region of Practical Equivalence set at ±1.7 *g*. Estimated effect sizes are regression coefficient estimates and Observed effects are the effect sizes and confidence intervals from the included studies.

## California Verbal Learning Test

Figure 1. Forest plot for the California Verbal Learning Test: Recognition scores. Regression coefficients and 95% confidence intervals are displayed. Dashed vertical lines show the lower and upper bound of the Region of Practical Equivalence set at ±1.7 *g*. Estimated effect sizes are regression coefficient estimates and Observed effects are the effect sizes and confidence intervals from the included studies.

## CERAD: Word List Recognition

Figure 1. Forest plot for the Consortium to Establish a Registry for Alzheimer’s Disease (CERAD): Word List Recognition scores. Regression coefficients and 95% confidence intervals are displayed. Dashed vertical lines show the lower and upper bound of the Region of Practical Equivalence set at ±1.7 *g*. Estimated effect sizes are regression coefficient estimates and Observed effects are the effect sizes and confidence intervals from the included studies.

## Discriminability (*d’*)

Figure 1. Forest plot for the discriminability index (*d’*). Regression coefficients and 95% confidence intervals are displayed. Dashed vertical lines show the lower and upper bound of the Region of Practical Equivalence set at ±1.7 *g*. Estimated effect sizes are regression coefficient estimates and Observed effects are the effect sizes and confidence intervals from the included studies. MAS: Memory Assessment Scales.

## Recognition Hits and False Alarms

Figure 1. Forest plot for Recognition Hits and False Alarms. Regression coefficients and 95% confidence intervals are displayed. Dashed vertical lines show the lower and upper bound of the Region of Practical Equivalence set at ±1.7 *g*. Estimated effect sizes are regression coefficient estimates and Observed effects are the effect sizes and confidence intervals from the included studies. CVLT: California Verbal Learning Test; DRM Paradigm: Deese-Roediger-McDermott paradigm.

## Other Measures of Visual Recognition Memory

Figure 1. Forest plot for Other Measures of Visual Recognition Memory. Regression coefficients and 95% confidence intervals are displayed. Dashed vertical lines show the lower and upper bound of the Region of Practical Equivalence set at ±1.7 *g*. Estimated effect sizes are regression coefficient estimates and Observed effects are the effect sizes and confidence intervals from the included studies. RCFT: Rey-Osterrieth Complex Figure Test; WMS: Wechsler Memory Scale.

## Other Measures of Verbal Recognition Memory

Figure 119. Forest plot for Other Measures of Verbal Recognition Memory. Regression coefficients and 95% confidence intervals are displayed. Dashed vertical lines show the lower and upper bound of the Region of Practical Equivalence set at ±1.7 *g*. Estimated effect sizes are regression coefficient estimates and Observed effects are the effect sizes and confidence intervals from the included studies.
