# Supplementary material for: Neuropsychological differential diagnosis of Alzheimer’s disease and vascular dementia: a systematic review with meta-regressions
Source: Front Aging Neurosci. 2023 Nov 6;15:1267434. doi: 10.3389/fnagi.2023.1267434 (PMC10657839; doi:10.3389/fnagi.2023.1267434)
Supplement: Supplementary file 3 [file Data_Sheet_1.PDF]

Supplementary Materials 3: Prior Choice Sensitivity Analyses

Contents

General Information on Prior Choice Sensitivity Analyses 4
Global Functioning 5
  Clinical Dementia Rating Scale 5
  Global Deterioration Scale 8
  Dementia Rating Scale 9
Orientation to Time and Space 10
Activities of Daily Living 13
  Activities of Daily Living: Quality Sensitivity Analysis 16
Disease Awareness 19
Affective Symptoms 21
  Affective Symptoms: Quality Sensitivity Analysis 24
Neuropsychiatric Symptoms 26
  Total Scores 26
  Domain Scores 28
  Total and Domain Scores: Quality Sensitivity Analysis 31
Apraxia 34
  Apraxia 34
  Facial Apraxia 35
  Ideomotor Apraxia 36
Motor Functioning 37
Visuo-Spatial Processing 40
  WAIS: Block Design, Object Assembly, Picture Completion 40
  Clock Drawing Test: Clock Copying, Clock Drawing, Clock Reading 43
  Rey-Osterrieth Complex Figure Test 46
  Visual Object and Spatial Processing Test Battery (VOSP) 47
  CERAD: Constructional Praxis 48
  Addenbrooke's Cognitive Examination - R 49
  Judgment of Line Orientation 50
  Hooper's Test 51
  Line Bisection 52
  Other Figure Copy Measures 54
  Other Measures of Constructional Praxis 57
  Other Measures of Visuo-Spatial Processing 60
  Visuo-Spatial Processing: Quality Sensitivity Analysis 63
Intelligence Measures 66

Attention ... 69
Trail Making Test – A ... 69
Digit Span Forward ... 71
Digit Symbol Substitution Test ... 73
Symbol Digit Modalities Test ... 74
Choice Reaction Time ... 75
Other Measures of Selective Attention ... 76
Continuous Performance Tests ... 78
Other Measures of Sustained Attention ... 80
Other Measures of Visual Attention ... 81
Other Measures of Attention ... 83
Attention: Quality Sensitivity Analysis ... 84
Processing Speed ... 85
Stroop Test Word Reading and Colour Naming ... 85
Simple Reaction Time ... 87
Other Measures of Processing Speed ... 88
Language Production ... 90
Fluency ... 90
Boston Naming Test ... 94
Other Naming Measures ... 97
Addenbrooke's Cognitive Examination ... 100
Writing ... 101
Other Measures of Language Production ... 102
Boston Naming Test and Phonemic Fluency: Quality Sensitivity Analysis ... 103
Language Comprehension ... 104
Reading ... 106
Reasoning ... 108
Wechsler Adult Intelligence Scale ... 108
Wisconsin Card Sorting Test ... 111
Raven's Progressive Matrices ... 113
Raven's Coloured Progressive Matrices ... 115
Attentional Matrices ... 117
Frontal Assessment Battery: Abstraction ... 118
Other Measures of Reasoning and Abstraction ... 119
Executive Functioning ... 122
Wechsler Adult Intelligence Scale: Arithmetic & Total Digits ... 122
Trail Making Test ... 123
Frontal Assessment Battery Total Score ... 125

Stroop Interference Condition 126
Wechsler Memory Scale 127
Digit Span Backward 130
Visual Span 132
Maze Tasks 134
Graphical Sequence Test 135
Repetition of Words and Sentences 136
Arithmetic 138
Cognitive Control of Memory 140
Other Measures of Cognitive Flexibility 142
Cognitive Estimation 145
Global Measures of Executive Functioning 146
Set Maintenance 147
Other Measures of Verbal Working Memory 148
Other Measures of Visual Working Memory 149
Sequencing 150
Benton Visual Retention Test 151
Quality Sensitivity Analysis 152
Memory 154
Wechsler Memory Scale: Verbal Episodic Memory 154
Rey's Auditory Verbal Learning Test 157
California Verbal Learning Test 160
CERAD Word List 161
Hopkin's Verbal Learning Test: Learning 164
Addenbrooke's Cognitive Examination 165
General Measures of Verbal Memory 168
Other Measures of Verbal Learning 169
Global Measures of Associative Memory 172
Other Measures of Episodic Memory: Prose 173
Other Measures of Episodic Memory: Word Lists 176
Other Measures of Cued Recall of Word Lists 179
Memory Intrusions 182
Semantic Memory 185
Global Measures of Memory 188
Quality Sensitivity Analysis 189
Visual Episodic Memory 190
Wechsler Memory Scale 190
Rey-Osterrieth Complex Figure Test 191

Visual Associative Memory ... 194
Other Measures of Visual Memory ... 195
Recognition Memory ... 197
Wechsler Memory Scale ... 197
Rey's Auditory Verbal Learning Test ... 198
California Verbal Learning Test ... 199
CERAD Word List ... 200
Discriminability – *d'* ... 201
Recognition Hits and False Alarms ... 204
Other Measures of Visual Recognition Memory ... 207
Other Measures of Verbal Recognition Memory ... 208

General Information on Prior Choice Sensitivity Analyses

In this supplementary file we present plots showing the posterior means and 95% confidence intervals for regression coefficients and standard deviations of fixed and random effects. Models were fitted by combining each of the four coefficient priors with each of the three standard deviation priors (see Table 1). The same prior distribution settings were used for the study level standard deviations (τ_{Study}) and effect size level standard deviations ($\tau_{Study:Effect\ Size}$). This resulted in twelve different models fit for each test or group of tests. Not all models could be successfully fit. In plots where not all twelve prior combinations are displayed, the missing models did not converge.

Table 1. Table of prior distributions for regression coefficients and standard deviations of fixed and random effects used in the prior choice sensitivity analyses. Each coefficient prior was combined with every standard deviation prior, resulting in twelve models fit to each data set.

**Coefficient Priors**	**Prior Parameters**	**τ – Prior**	**τ – Prior Parameters**
Cauchy (0, 1)	location = 0, scale = 1	Cauchy (0, 0.5)	location = 0, scale = 0.5
Student-t (0, 1, *df* = 3)	location = 0, scale = 1, ν = 3	Exponential (1)	λ (rate) = 1
U (-10, 10)	lower bound = -10, upper bound = 10	Inverse Gamma (0.501, 0.501)	location = 0.501, scale = 0.501
N (0, 1)	location = 0, standard deviation = 1		

Global Functioning

Clinical Dementia Rating Scale

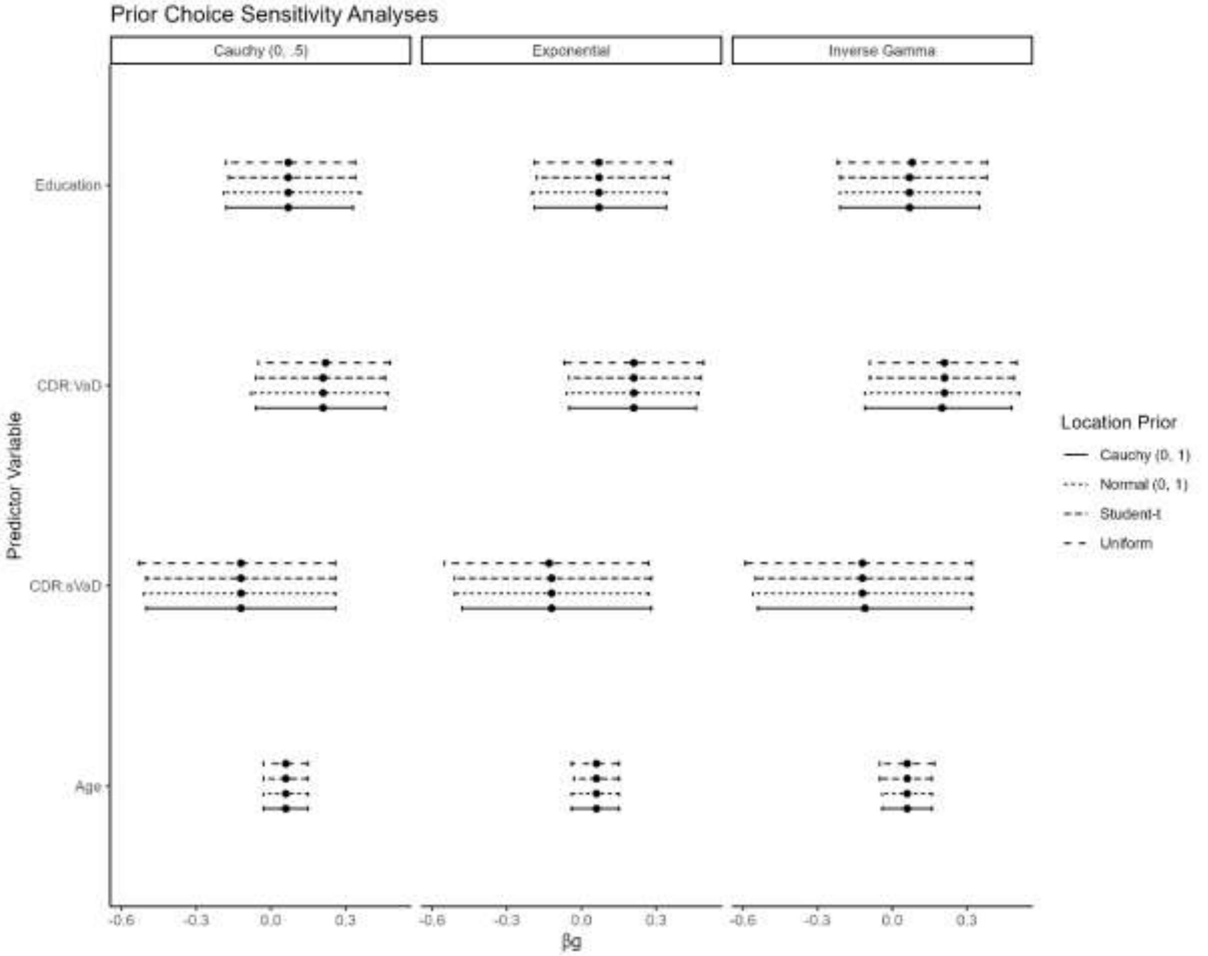

Figure 1. Regression coefficients with 95% confidence intervals for the Clinical Dementia Scale (CDR) model. Education: difference in years of education between the dementia groups.

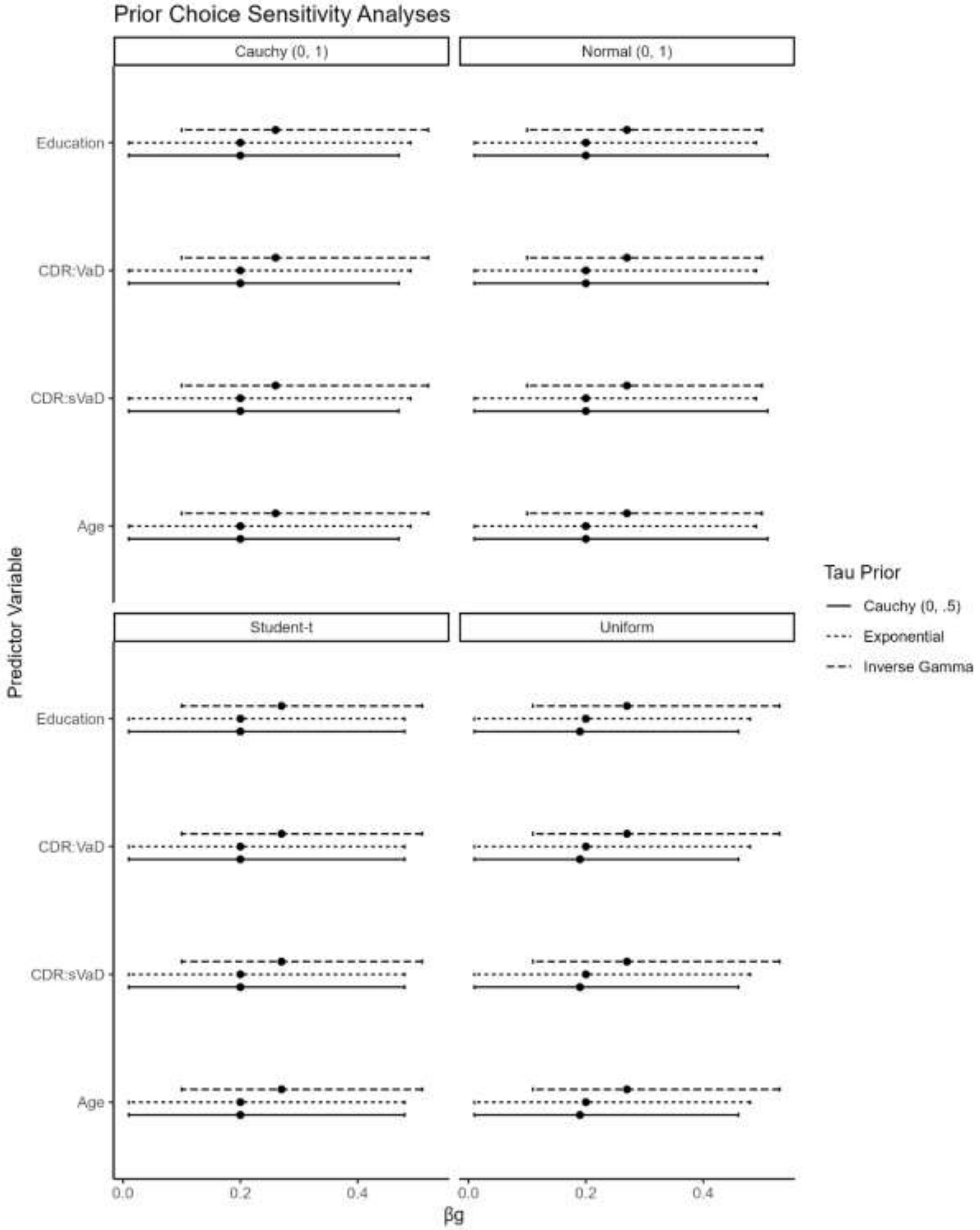

Figure 1. Study level standard deviation estimate with 95% confidence intervals for the Clinical Dementia Scale (CDR) model. Education: difference in years of education between the dementia groups.

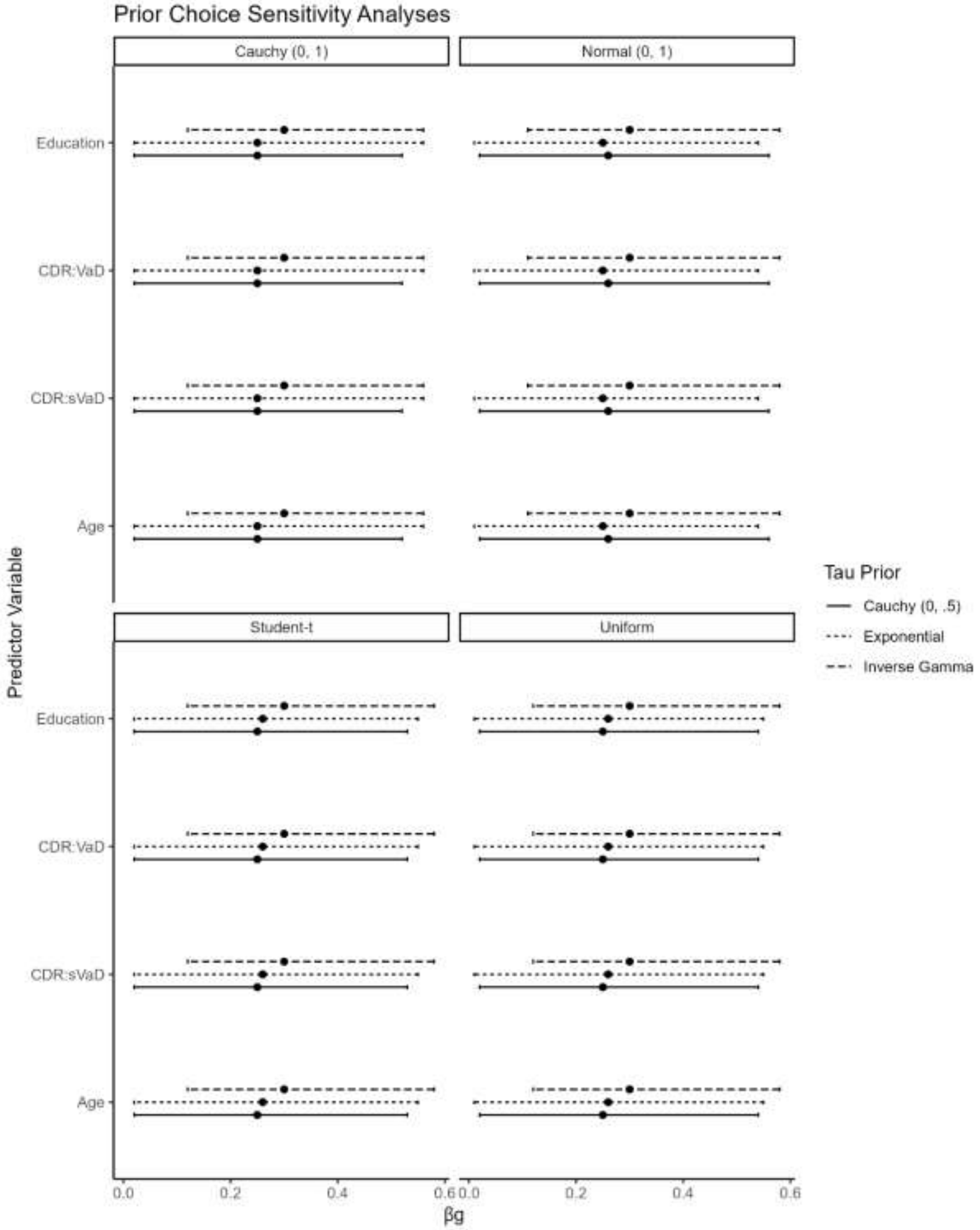

Figure 1. Effect size level standard deviation estimate with 95% confidence intervals for the Clinical Dementia Scale (CDR) model. Education: difference in years of education between the dementia groups.

Global Deterioration Scale

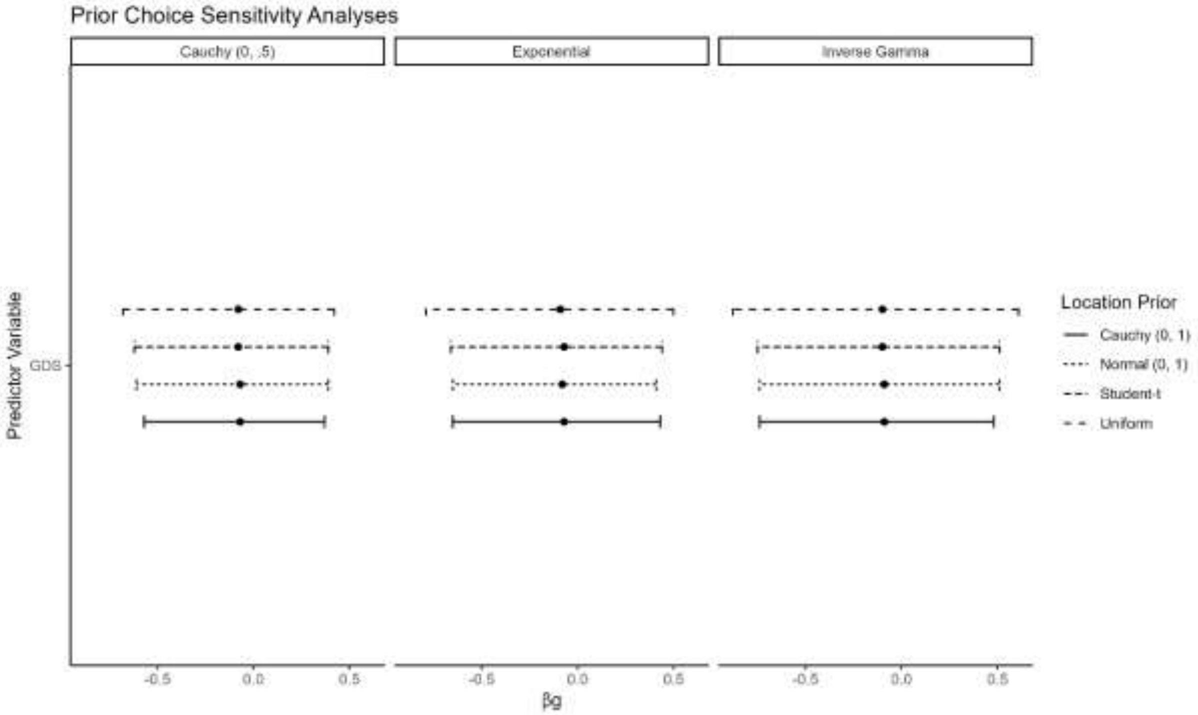

Figure 1. Regression coefficients with 95% confidence intervals for the Global Deterioration Scale (GDS) model.

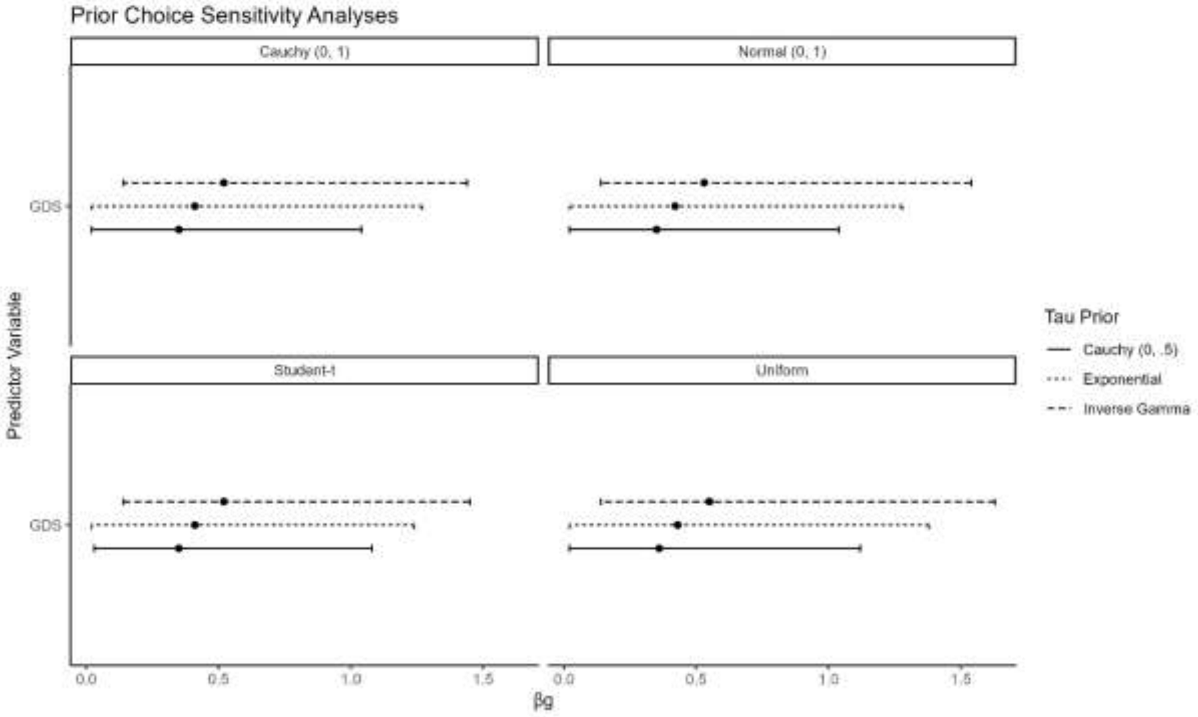

Figure 1. Study level standard deviation estimate with 95% confidence intervals for the Global Deterioration Scale (GDS) model.

Dementia Rating Scale

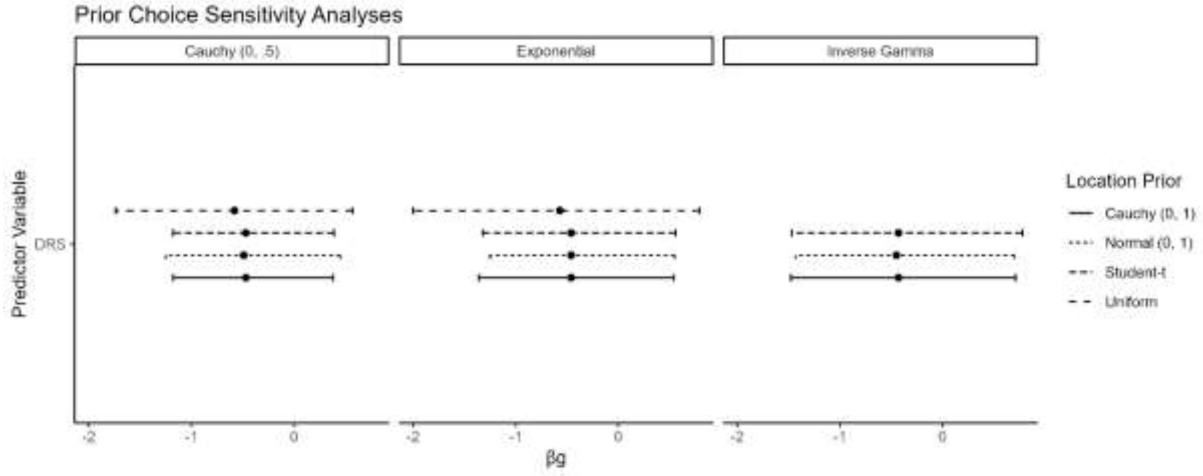

Figure 1. Regression coefficients with 95% confidence intervals for the Dementia Rating Scale (DRS) model.

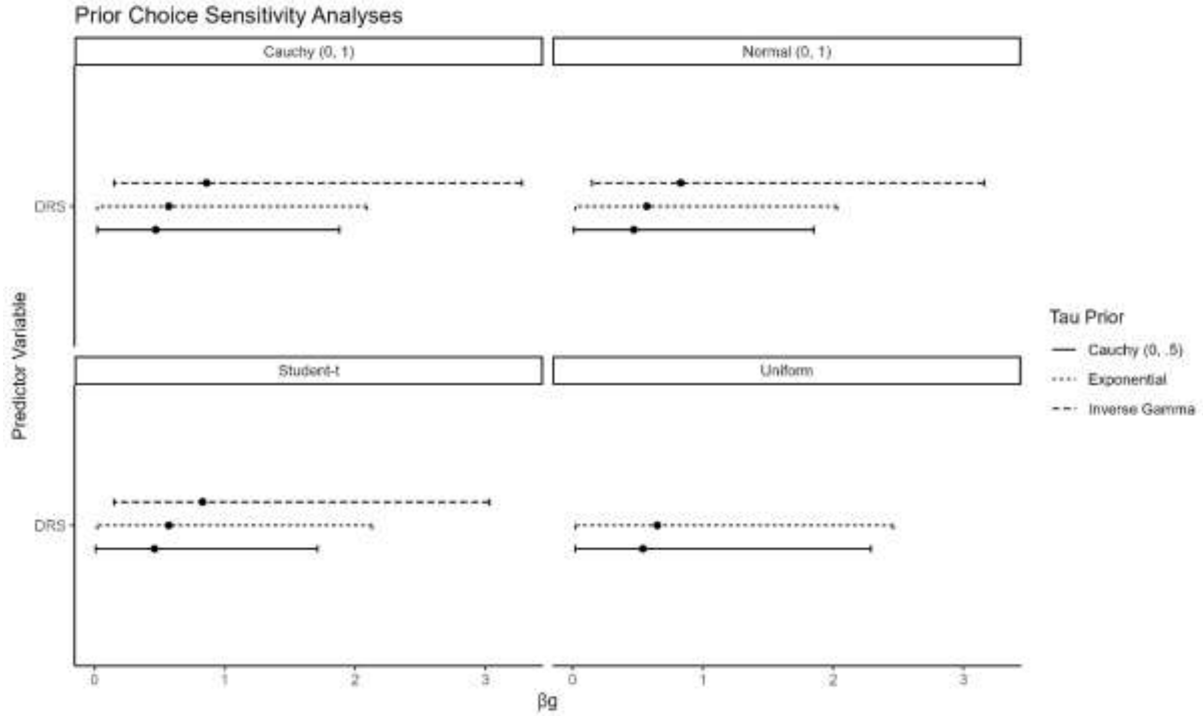

Figure 1. Study level standard deviation estimate with 95% confidence intervals for the Dementia Rating Scale (DRS) model.

Orientation to Time and Space

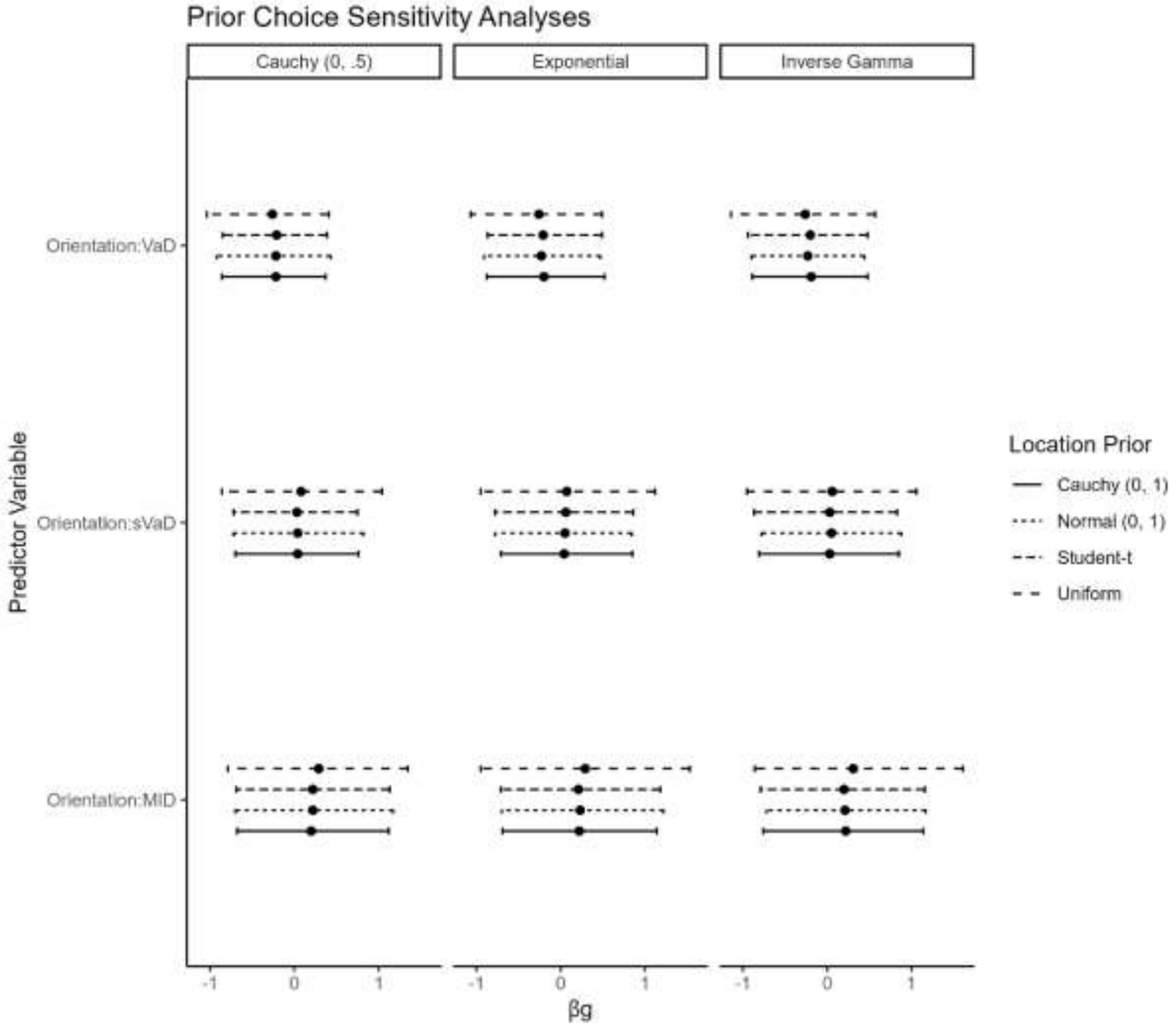

Figure 1. Regression coefficients with 95% confidence intervals for Orientation to Time and Space model. sVaD: subcortical vascular dementia, VaD: vascular dementia, MID: multi-infarct dementia.

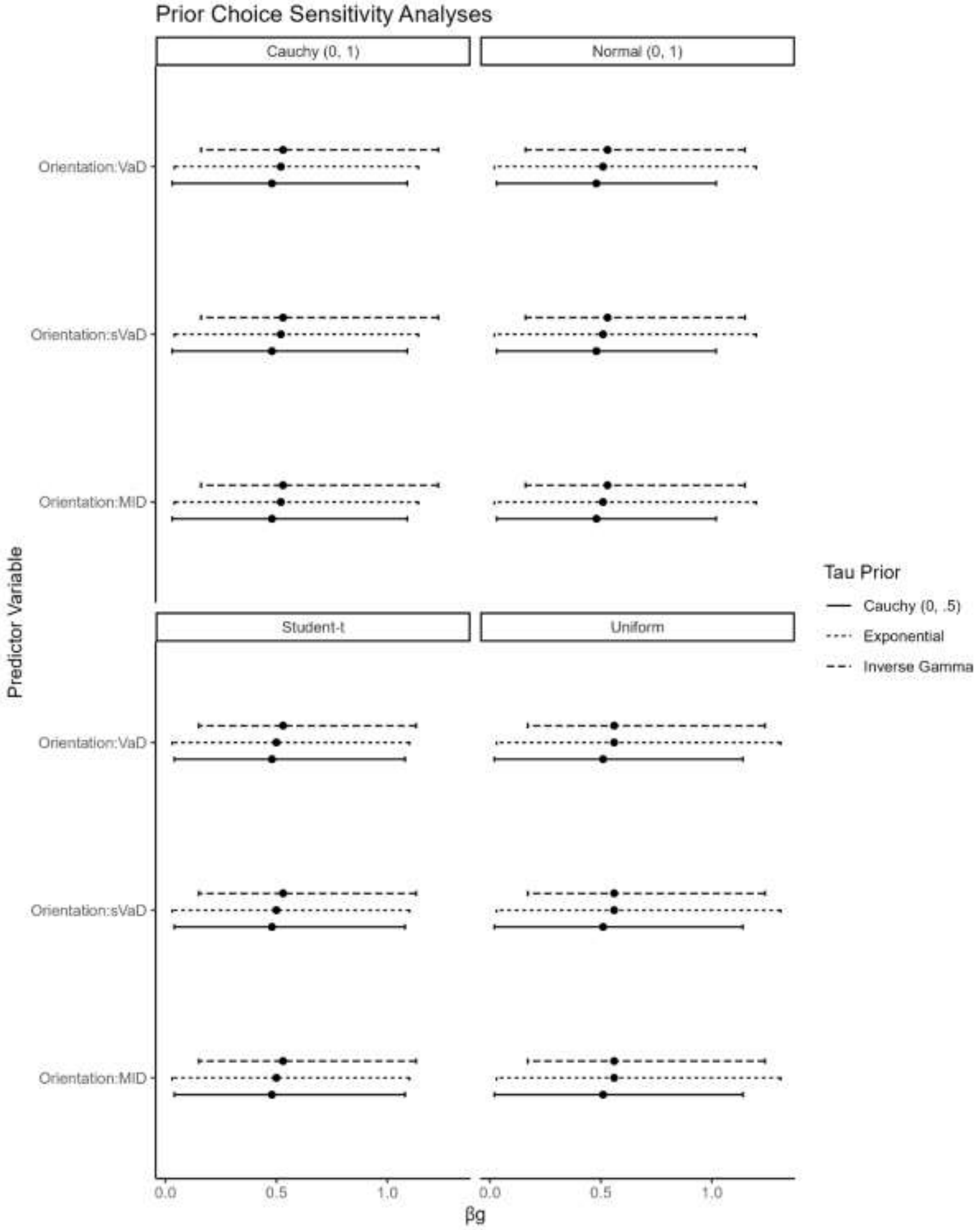

Figure 1. Study level standard deviation estimate with 95% confidence intervals for Orientation to Time and Space model. sVaD: subcortical vascular dementia, VaD: vascular dementia, MID: multi-infarct dementia.

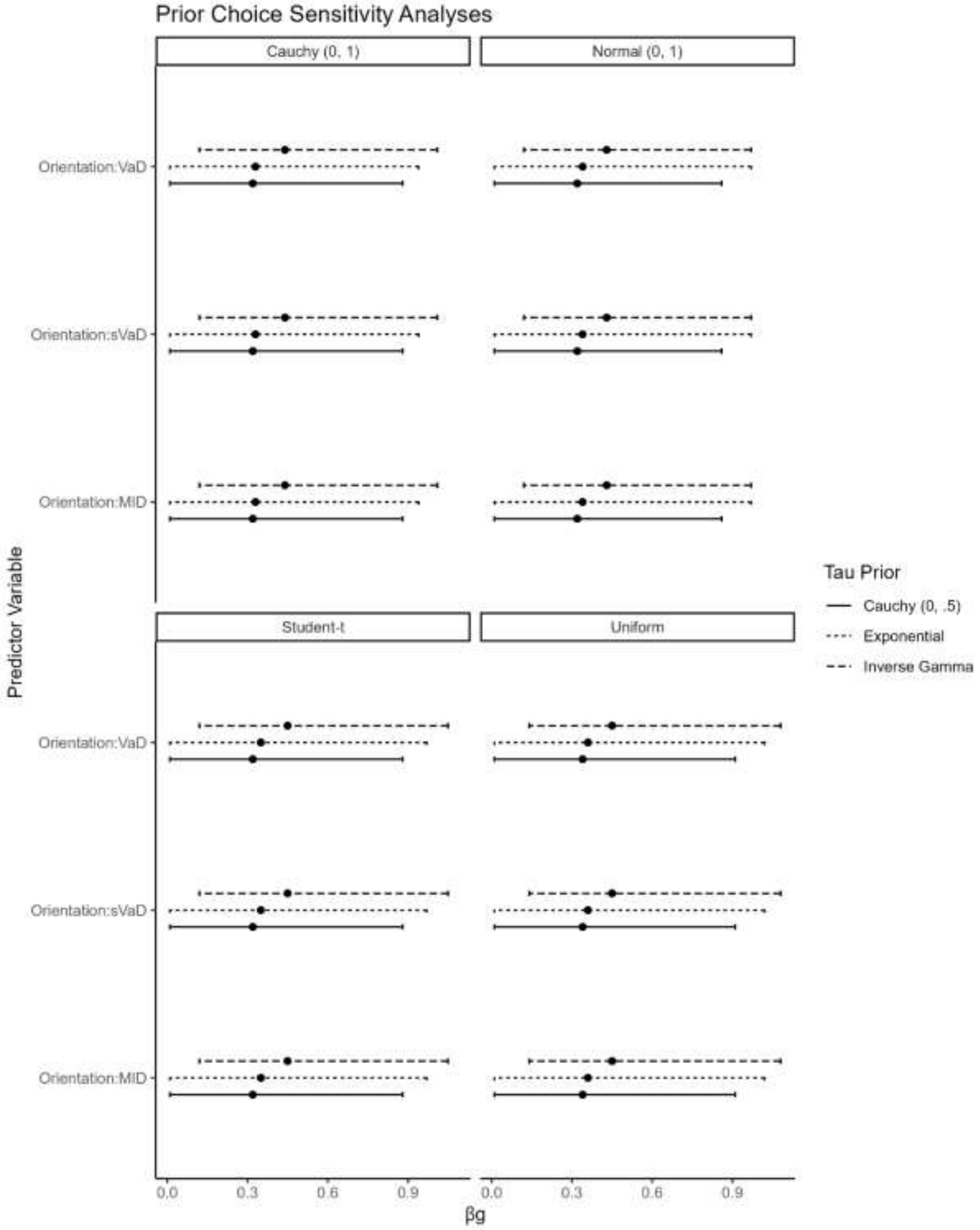

Figure 1. Effect size level standard deviation estimate with 95% confidence intervals for Orientation to Time and Space model. sVaD: subcortical vascular dementia, VaD: vascular dementia, MID: multi-infarct dementia.

Activities of Daily Living

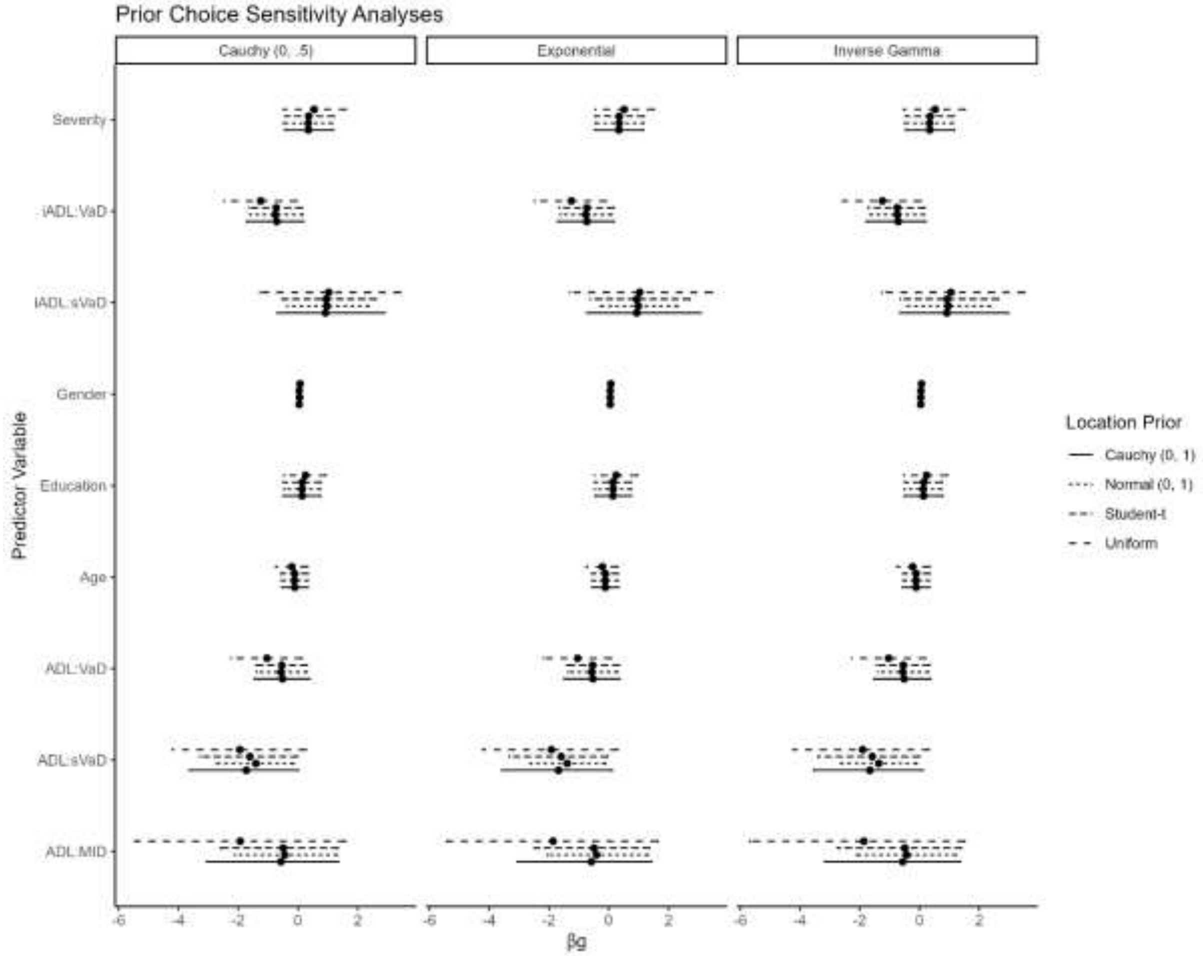

Figure 1. Regression coefficients with 95% confidence intervals for the basic (ADL) and instrumental Activities of Daily Living (iADL) model. sVaD: subcortical vascular dementia, VaD: vascular dementia, MID: multi-infarct dementia, Severity: difference in dementia severity between dementia groups, Education: difference in average years of education between dementia groups, Age: difference in average age between dementia groups, Gender: difference in proportion of women between dementia groups.

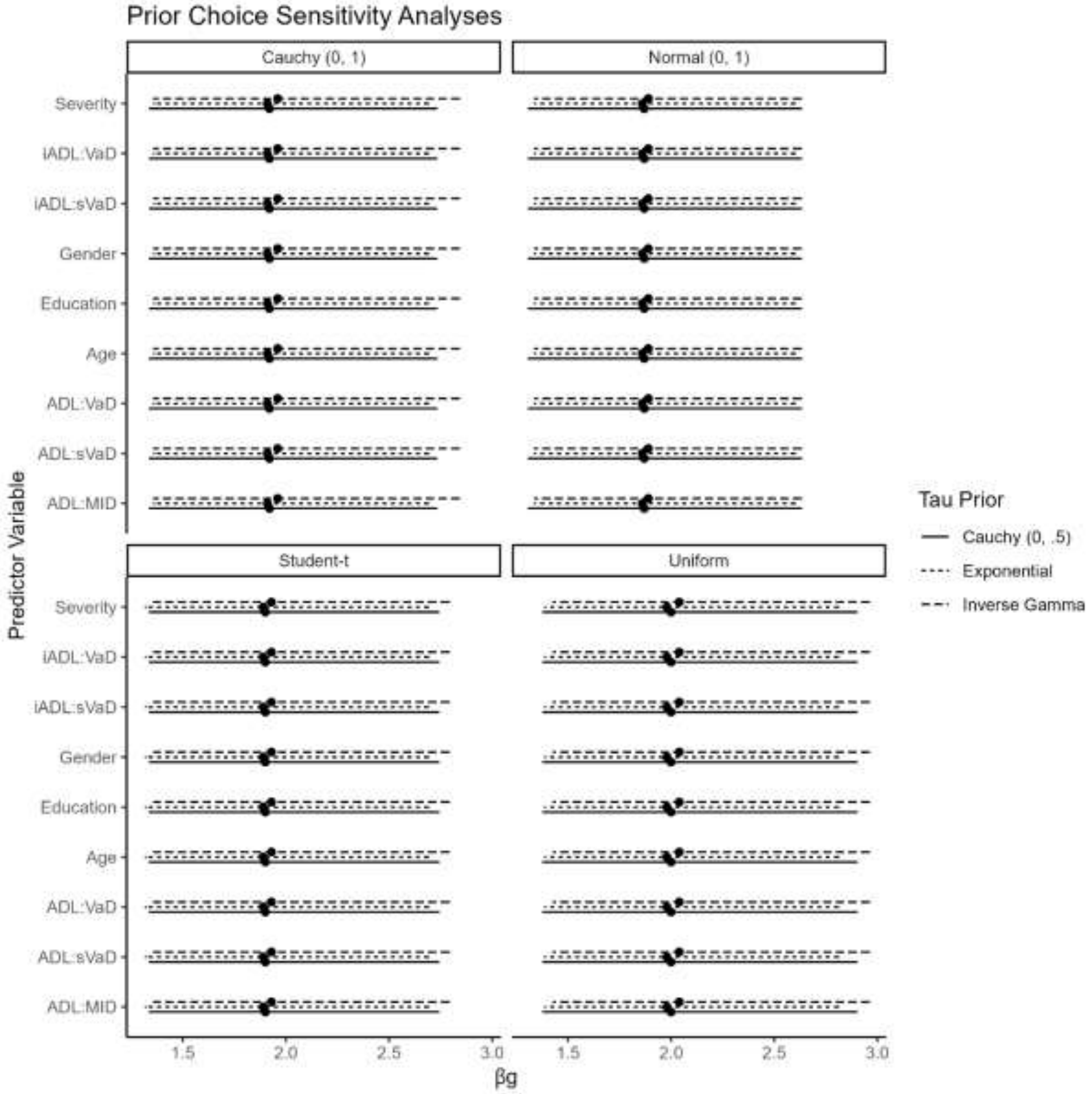

Figure 1. Study level standard deviation estimate with 95% confidence intervals for the basic (ADL) and instrumental Activities of Daily Living (iADL) model. sVaD: subcortical vascular dementia, VaD: vascular dementia, MID: multi-infarct dementia, Severity: difference in dementia severity between dementia groups, Education: difference in average years of education between dementia groups, Age: difference in average age between dementia groups, Gender: difference in proportion of women between dementia groups.

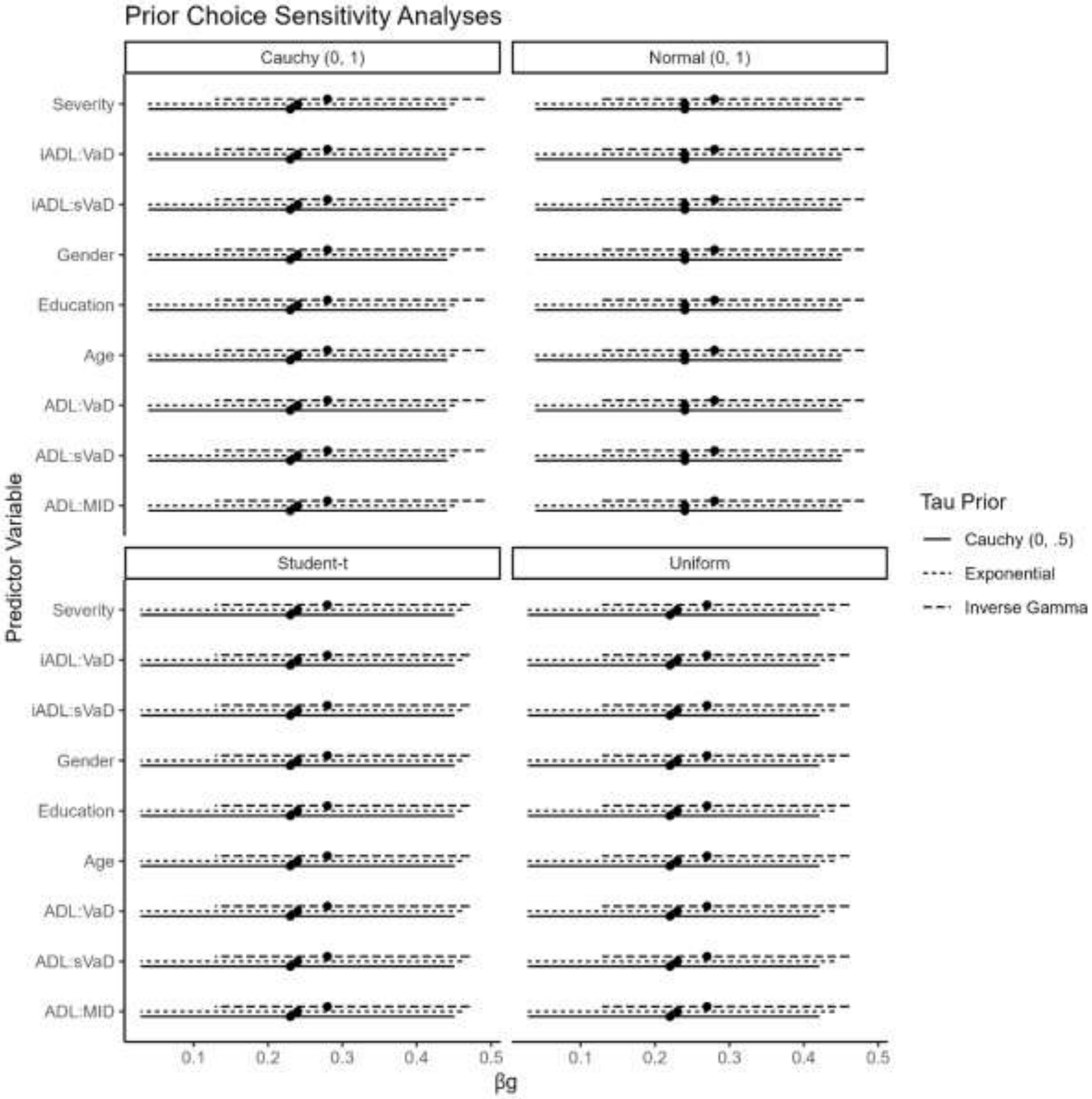

Figure 1. Effect size level standard deviation estimate with 95% confidence intervals for the basic (ADL) and instrumental Activities of Daily Living (iADL) model. sVaD: subcortical vascular dementia, VaD: vascular dementia, MID: multi-infarct dementia, Severity: difference in dementia severity between dementia groups, Education: difference in average years of education between dementia groups, Age: difference in average age between dementia groups, Gender: difference in proportion of women between dementia groups.

Activities of Daily Living: Quality Sensitivity Analysis

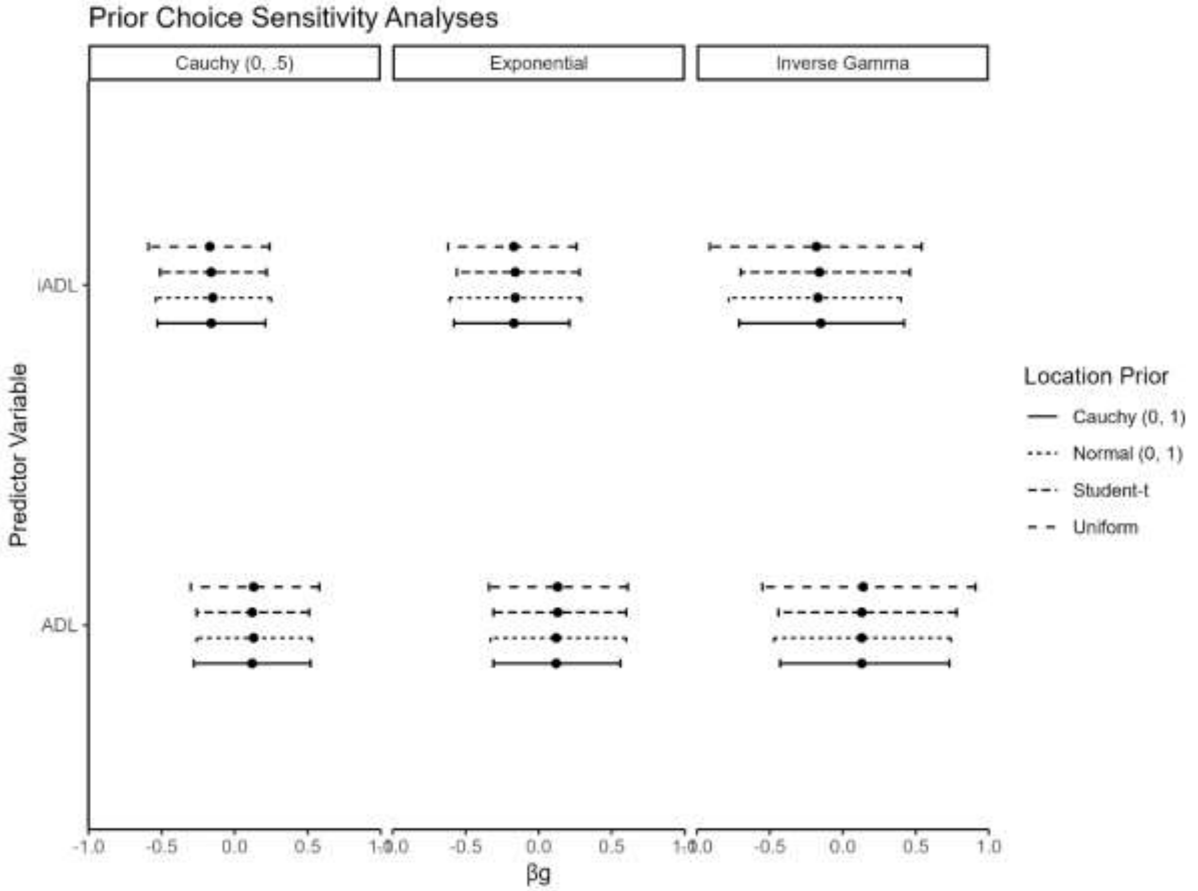

Figure 1. Regression coefficients with 95% confidence intervals for the basic (ADL) and instrumental Activities of Daily Living (iADL) study quality sensitivity analysis model.

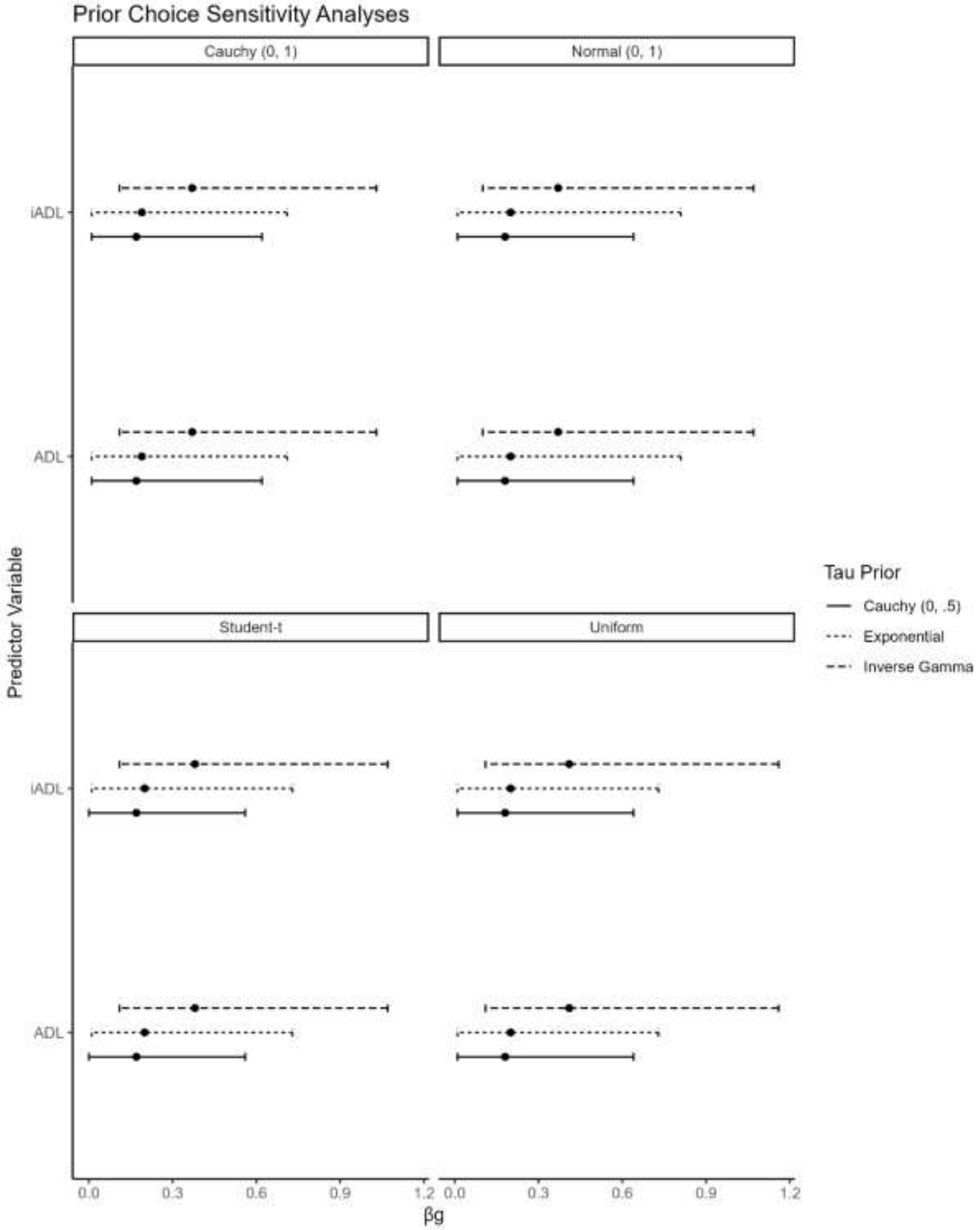

Figure 1. Study level standard deviation estimate with 95% confidence intervals for the basic (ADL) and instrumental Activities of Daily Living (iADL) study quality sensitivity analysis model.

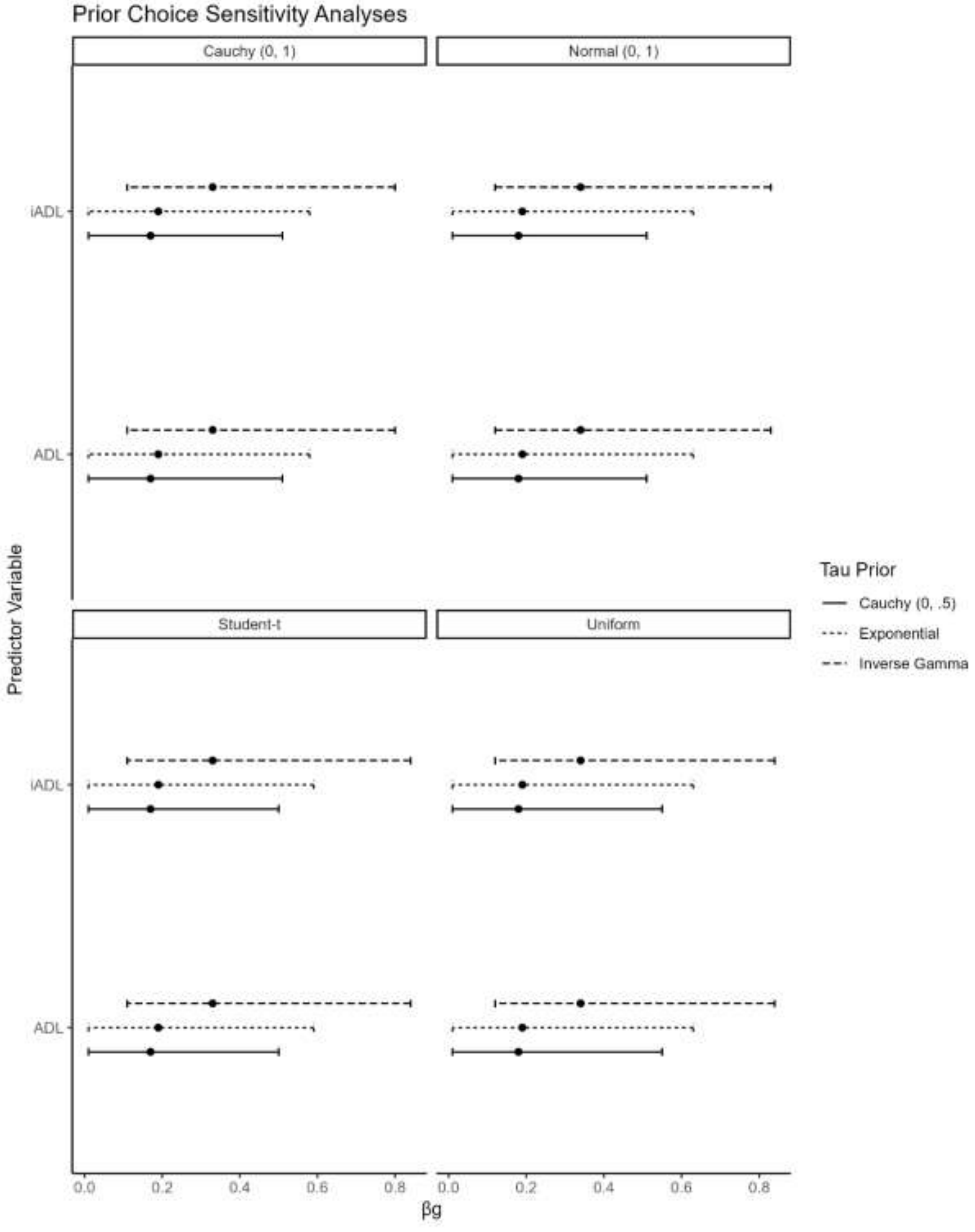

Figure 1. Effect size level standard deviation estimate with 95% confidence intervals for the basic (ADL) and instrumental Activities of Daily Living (iADL) study quality sensitivity analysis model.

Disease Awareness

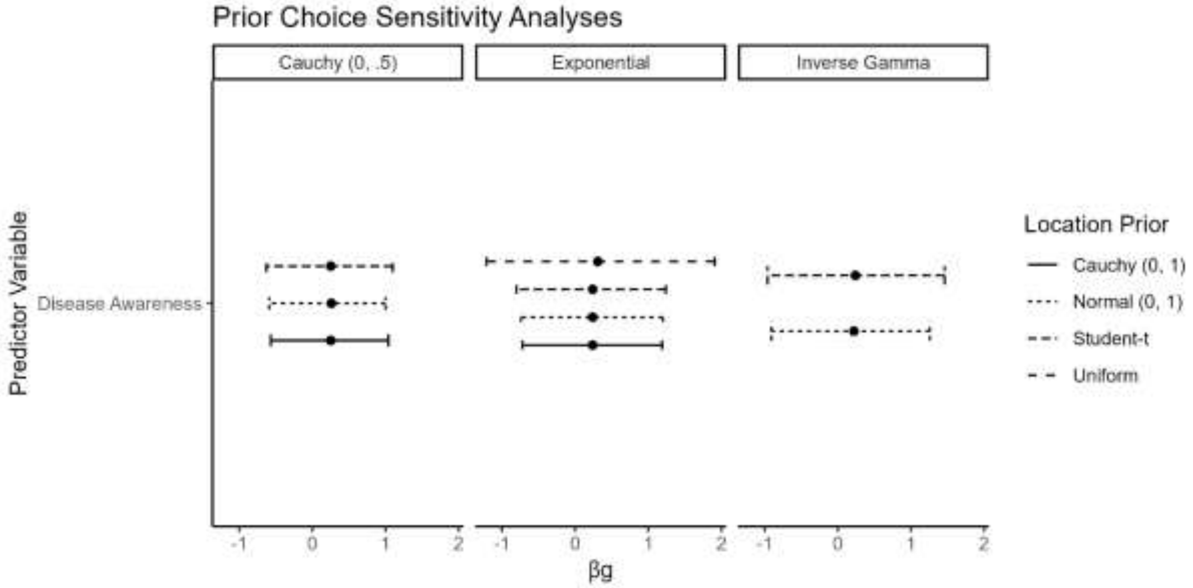

Figure 1. Regression coefficients with 95% confidence intervals for the Disease Awareness model.

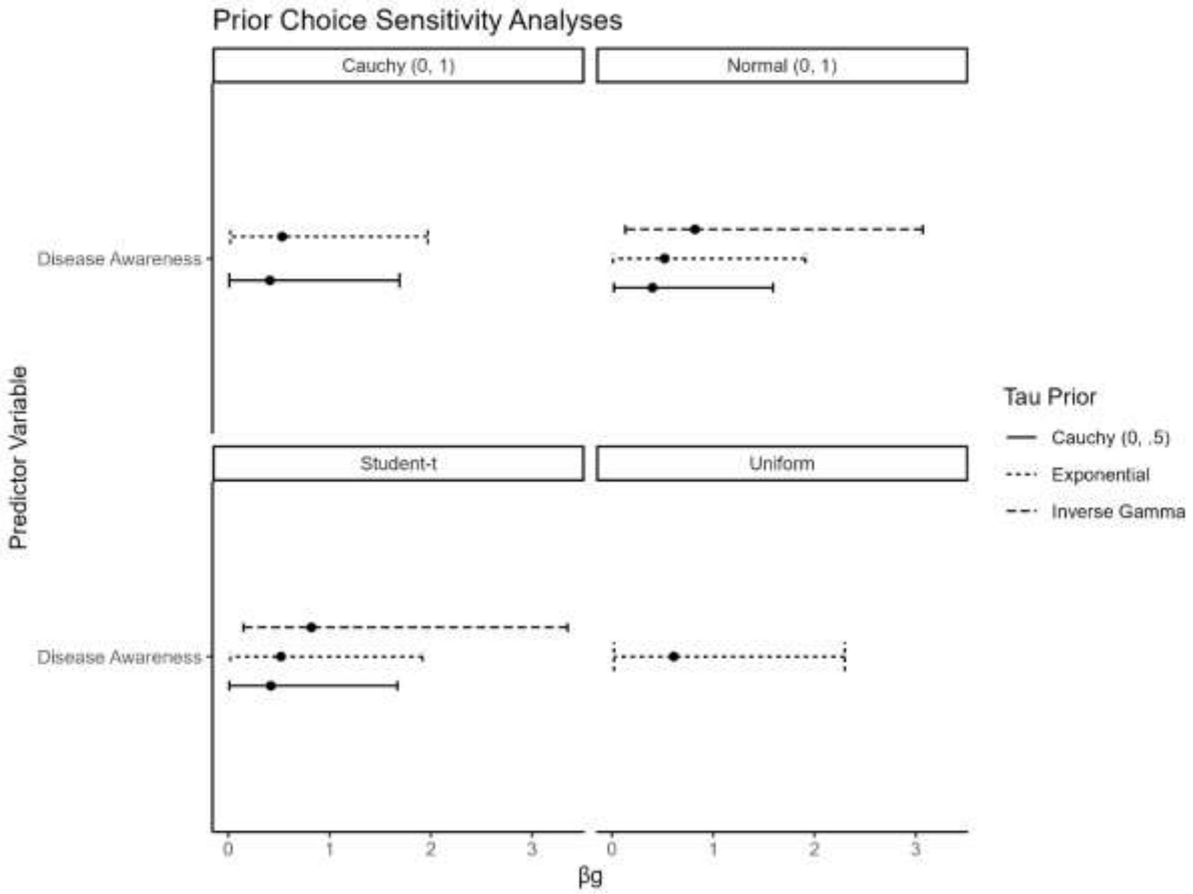

Figure 1. Study level standard deviation estimate with 95% confidence intervals for the Disease Awareness model.

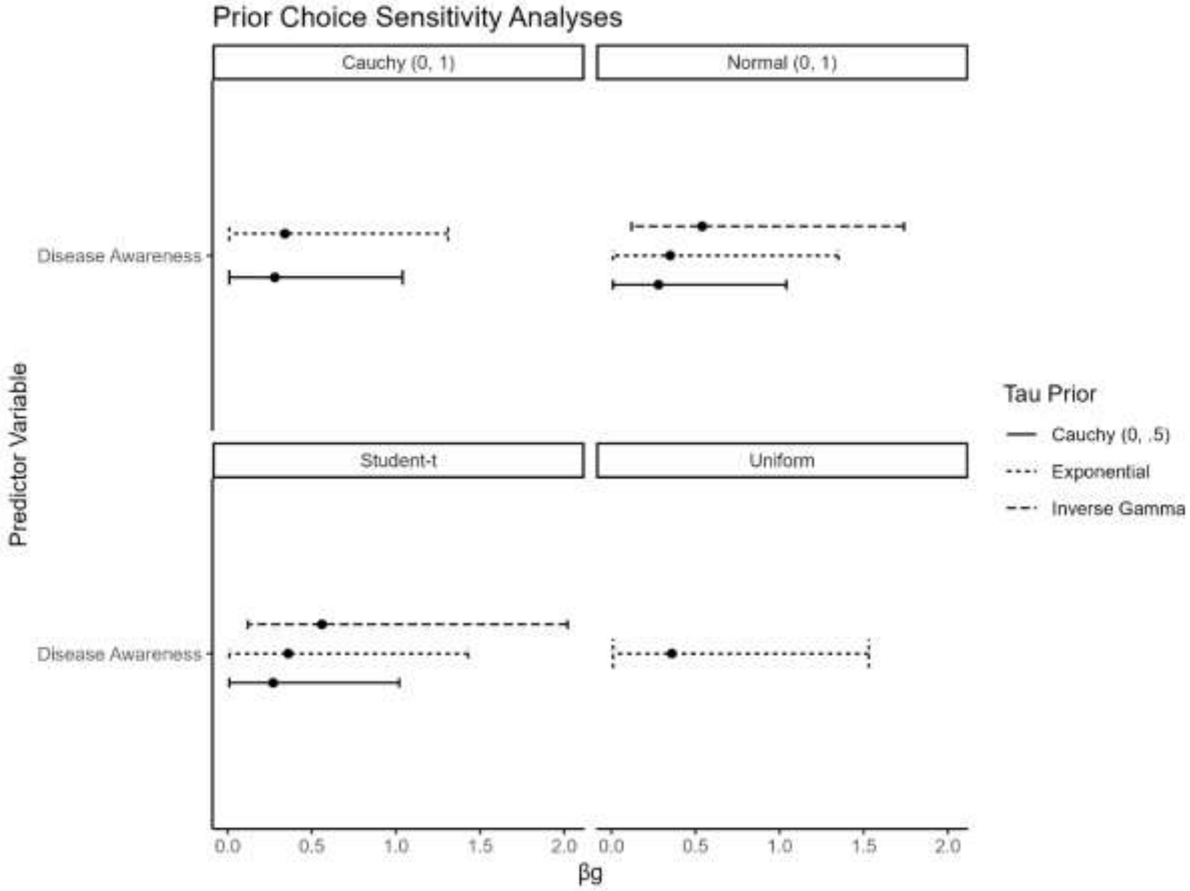

Figure 1. Effect size level standard deviation estimate with 95% confidence intervals for the Disease Awareness model.

Affective Symptoms

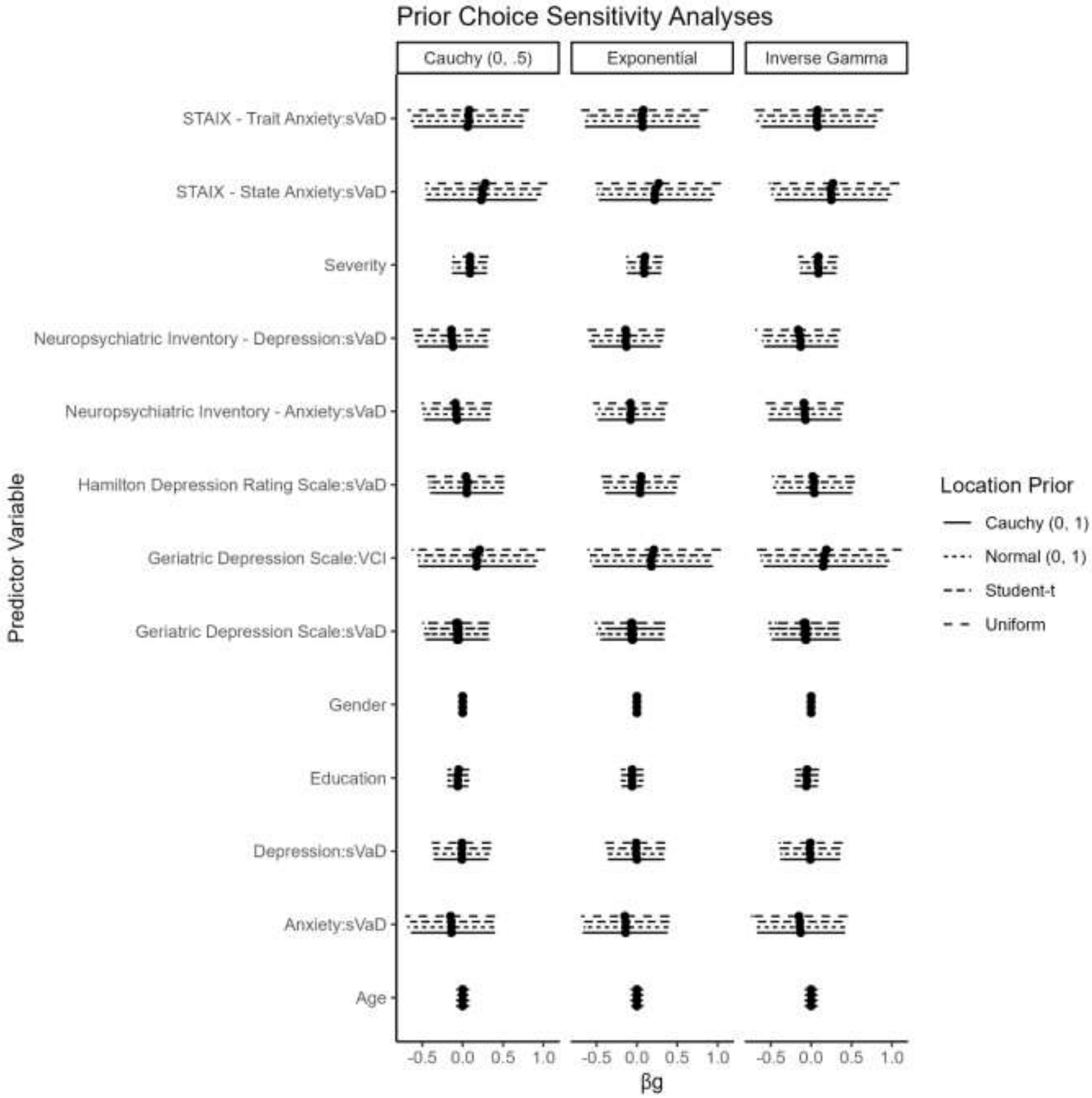

Figure 1. Regression coefficients with 95% confidence intervals for the Affective Symptoms model. STAIX: State-Trait Anxiety Inventory. sVaD: subcortical vascular dementia, VaD: vascular dementia, VCI: vascular cognitive impairment, Severity: difference in dementia severity between dementia groups, Education: difference in average years of education between dementia groups, Age: difference in average age between dementia groups, Gender: difference in proportion of women between dementia groups.

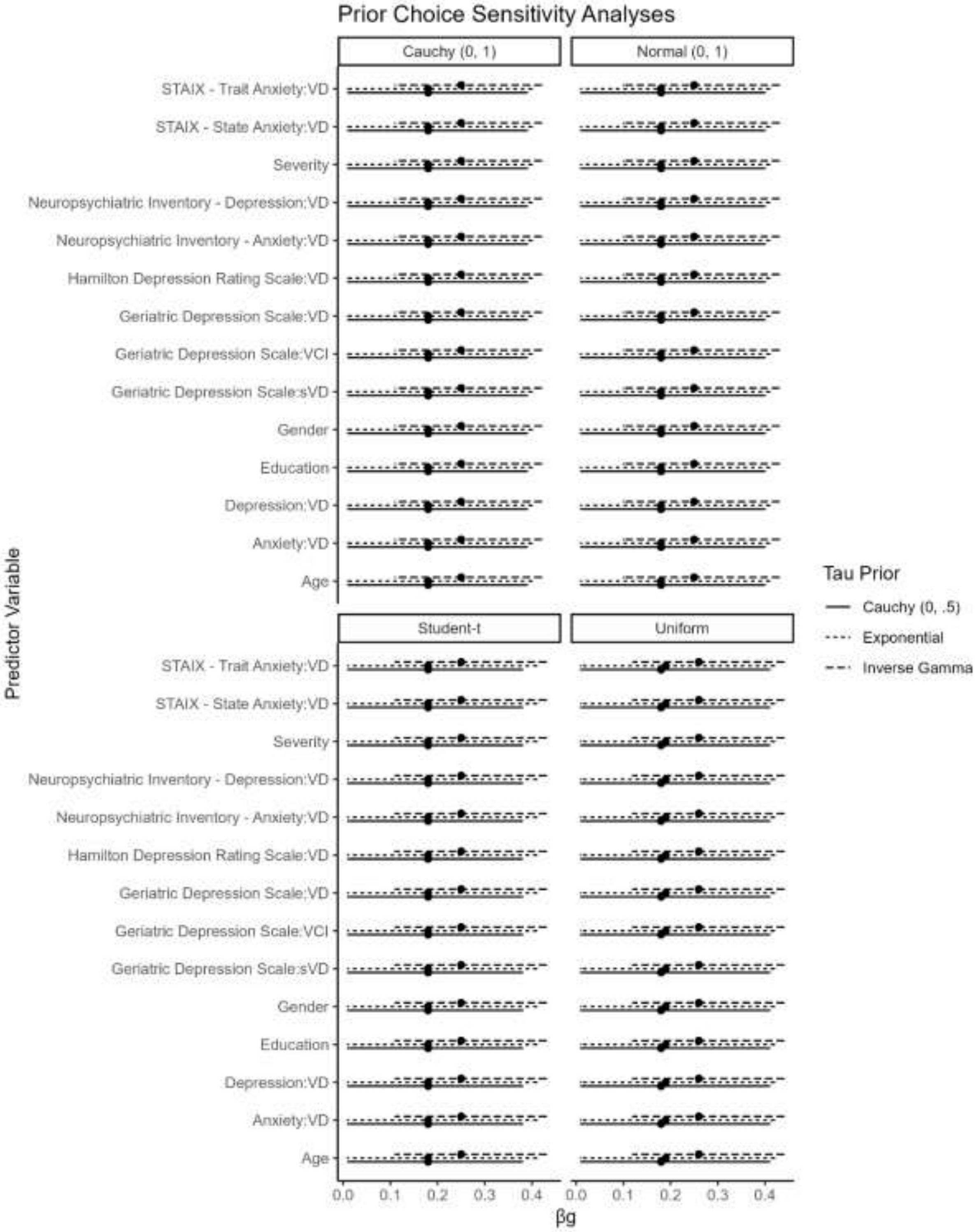

Figure 1. Study level standard deviation estimates with 95% confidence intervals for the Affective Symptoms model. STAIX: State-Trait Anxiety Inventory. sVaD: subcortical vascular dementia, VaD: vascular dementia, VCI: vascular cognitive impairment, Severity: difference in dementia severity between dementia groups, Education: difference in average years of education between dementia groups, Age: difference in average age between dementia groups, Gender: difference in proportion of women between dementia groups.

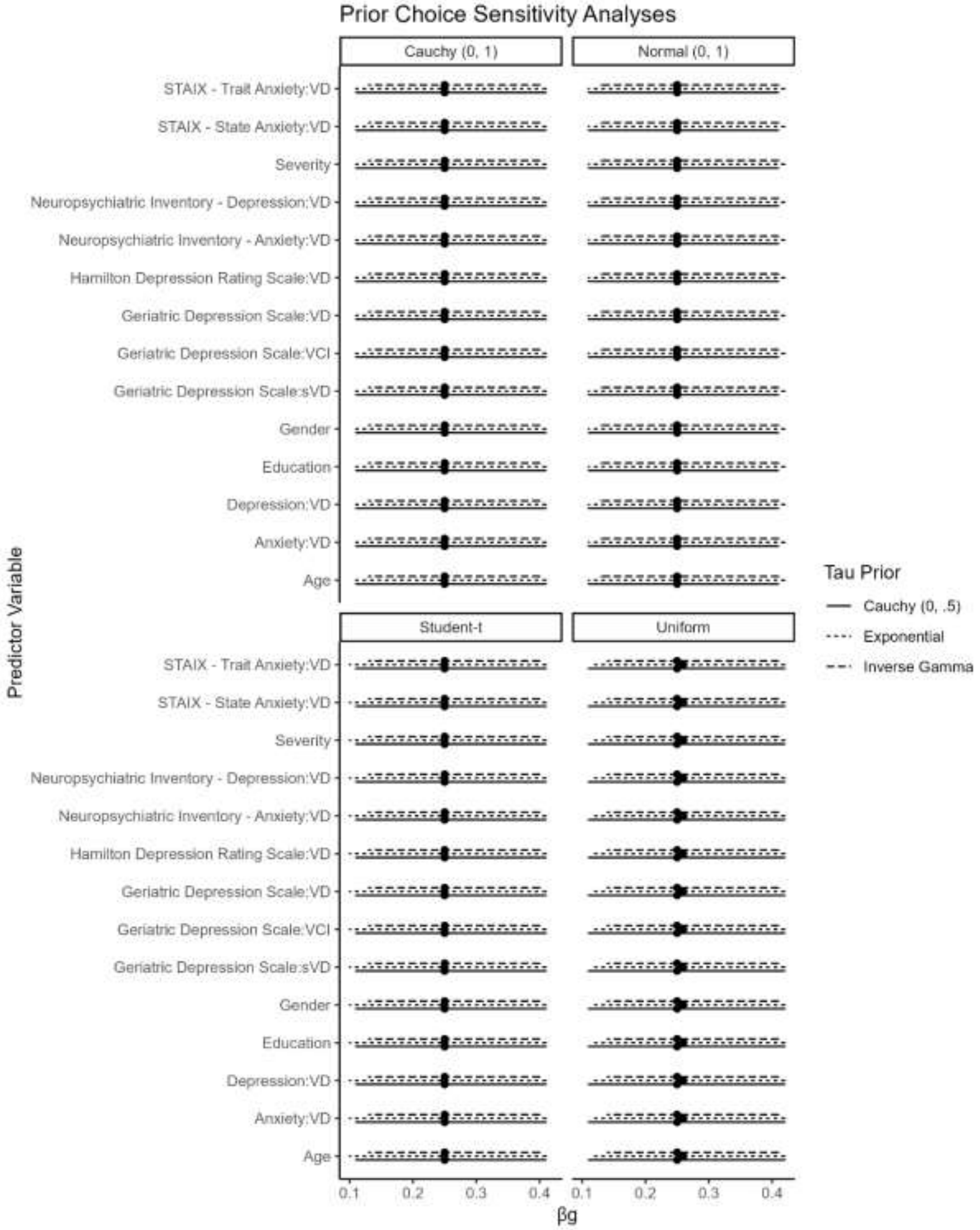

Figure 1. Effect size level standard deviation estimates with 95% confidence intervals for the Affective Symptoms model. STAIX: State-Trait Anxiety Inventory. sVaD: subcortical vascular dementia, VaD: vascular dementia, VCI: vascular cognitive impairment, Severity: difference in dementia severity between dementia groups, Education: difference in average years of education between dementia groups, Age: difference in average age between dementia groups, Gender: difference in proportion of women between dementia groups.

Affective Symptoms: Quality Sensitivity Analysis

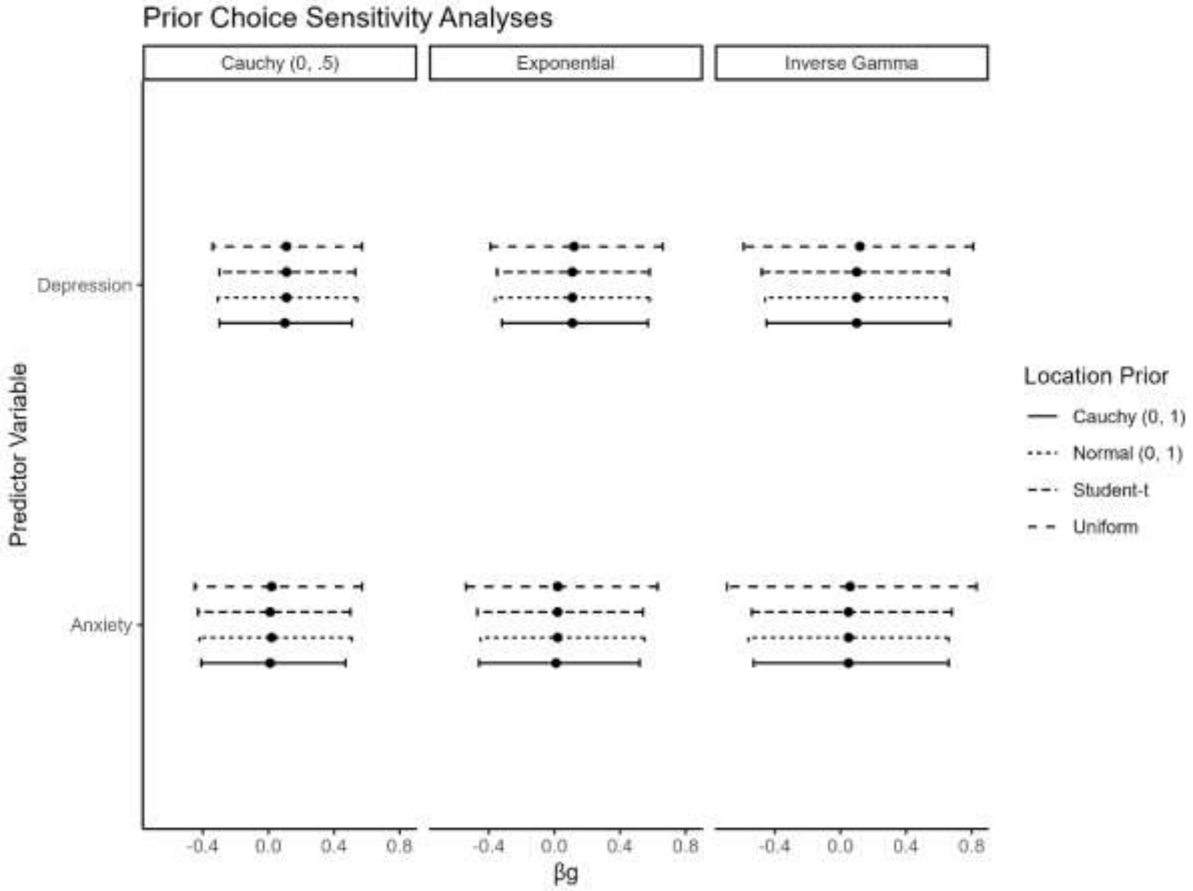

Figure 1. Regression coefficients with 95% confidence intervals for the study quality sensitivity analysis for Affective Symptoms model. For tests and studies included in the analysis see Supplementary Materials 1.

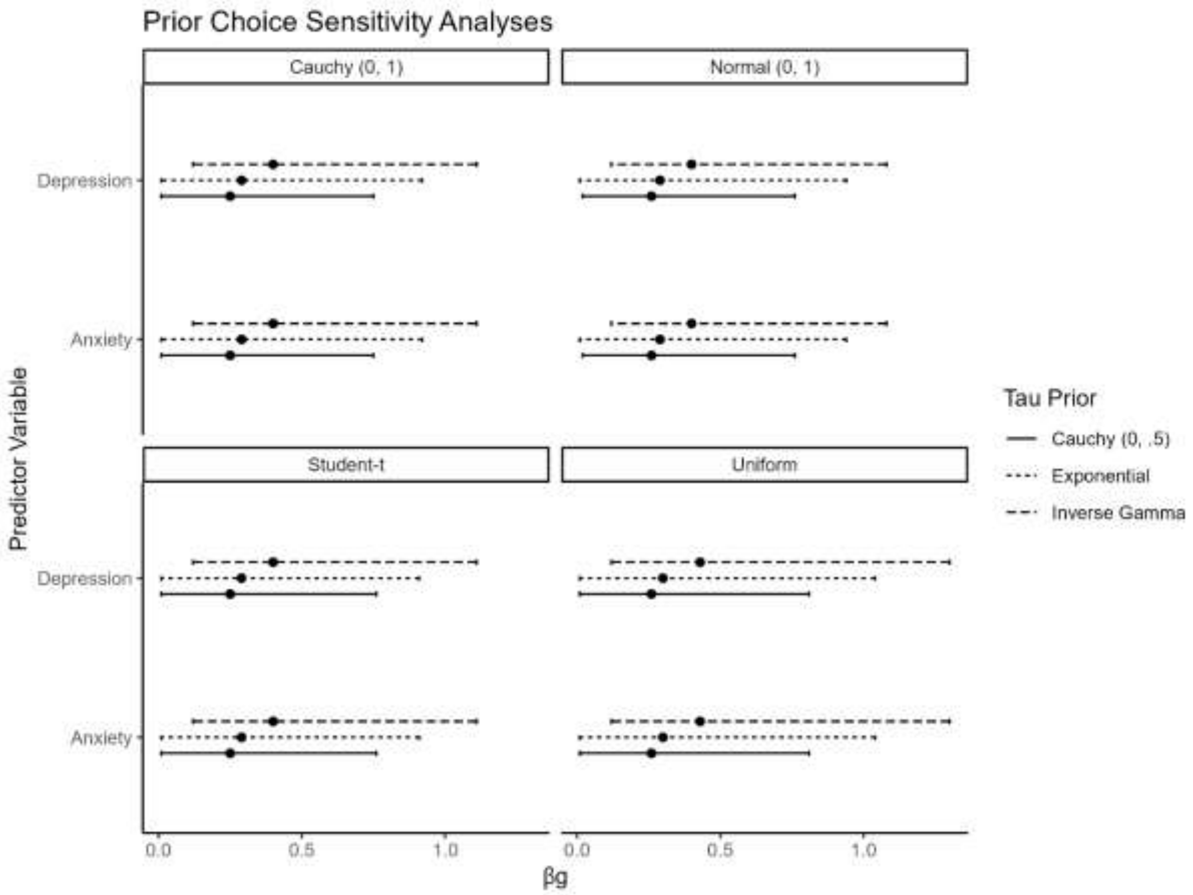

Figure 1. Study level standard deviation estimates with 95% confidence intervals for the study quality sensitivity analysis for Affective Symptoms model. For tests and studies included in the analysis see Supplementary Materials 1.

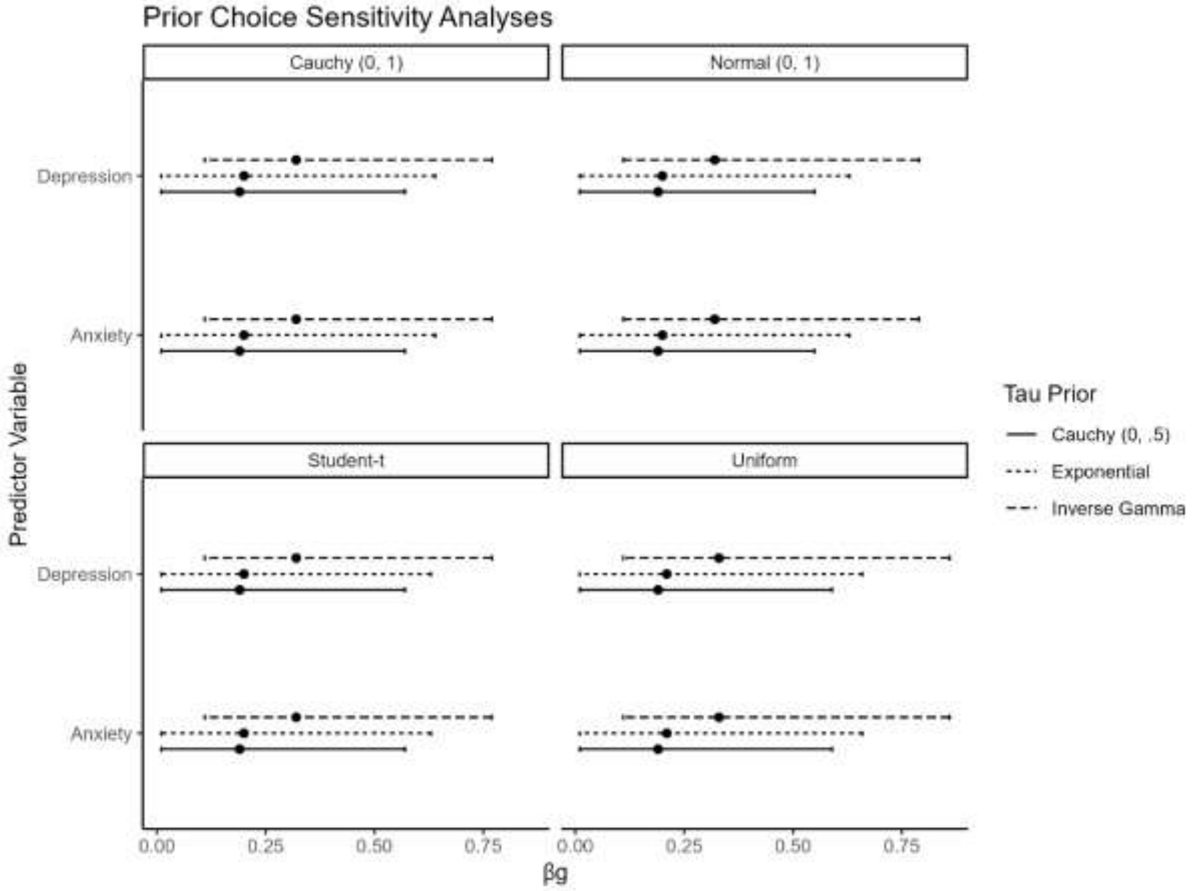

Figure 1. Effect size level standard deviation estimates with 95% confidence intervals for the study quality sensitivity analysis for Affective Symptoms model. For tests and studies included in the analysis see Supplementary Materials 1.

Neuropsychiatric Symptoms

Total Scores

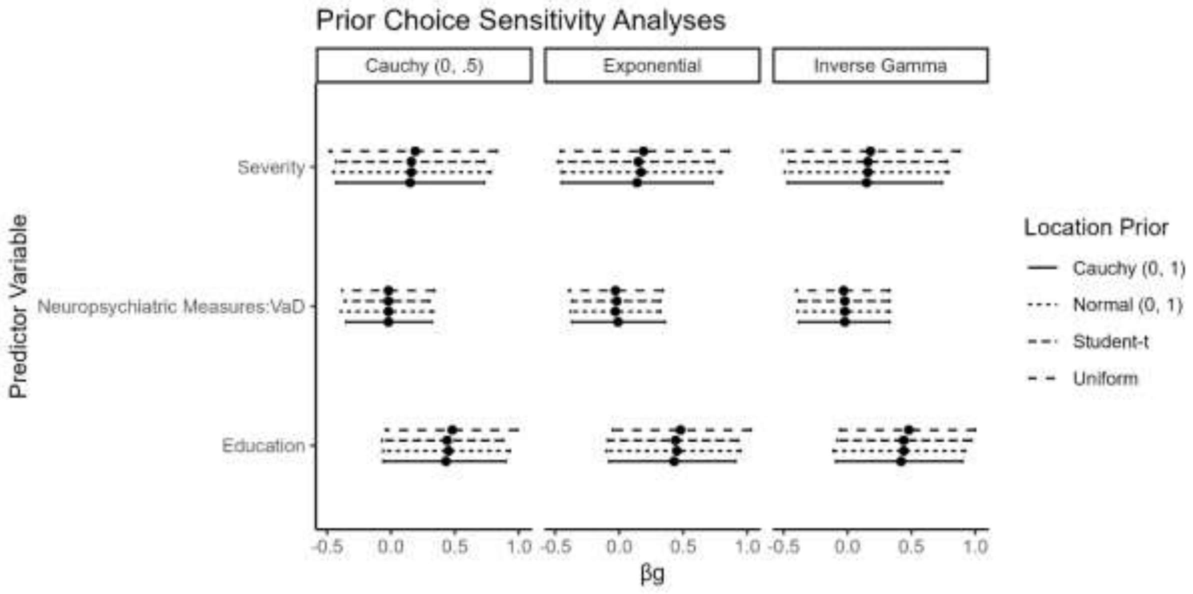

Figure 1. Regression coefficients with 95% confidence intervals for the Neuropsychiatric Symptoms Total Scores model. VaD: vascular dementia, Severity: difference in dementia

severity between dementia groups, Education: difference in average years of education between dementia groups.

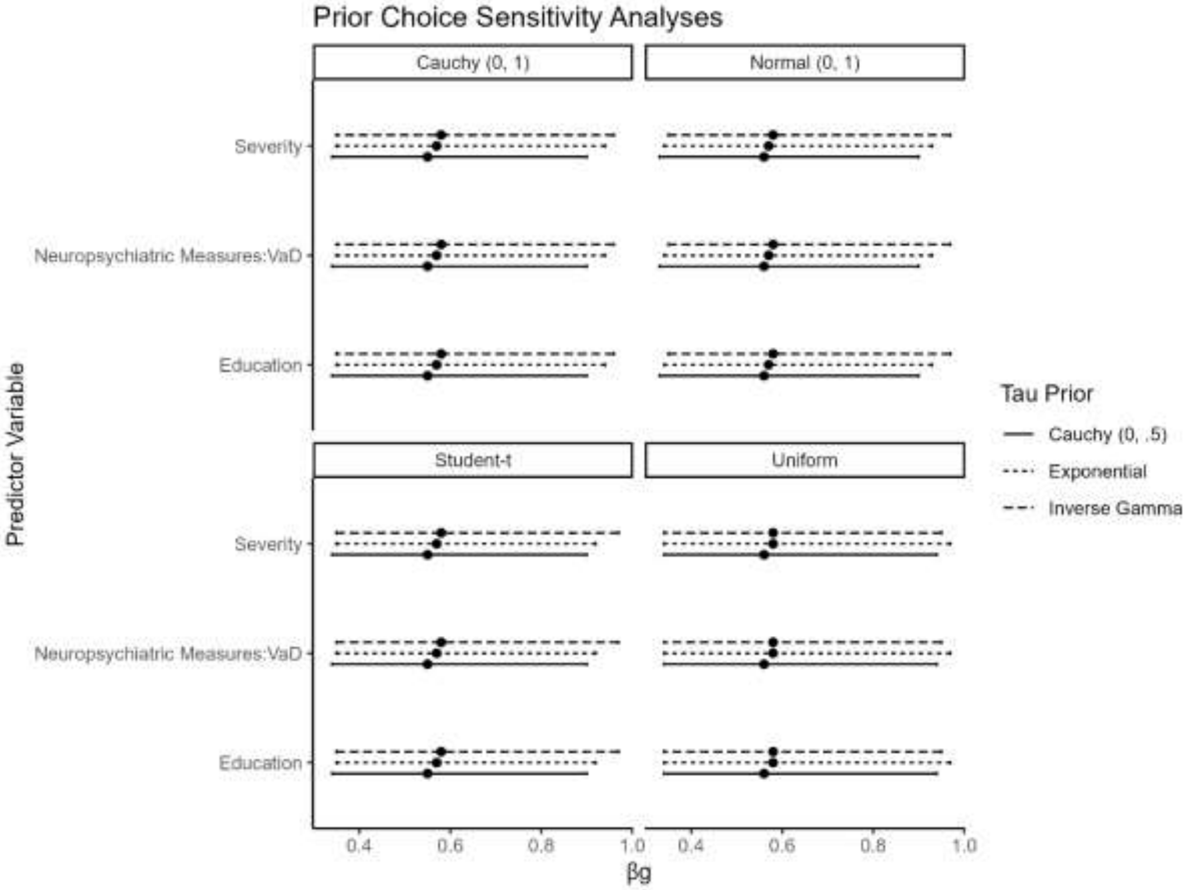

Figure 1. Study level standard deviation estimates with 95% confidence intervals for the Neuropsychiatric Symptoms Total Scores model. VaD: vascular dementia, Severity: difference in dementia severity between dementia groups, Education: difference in average years of education between dementia groups.

Domain Scores

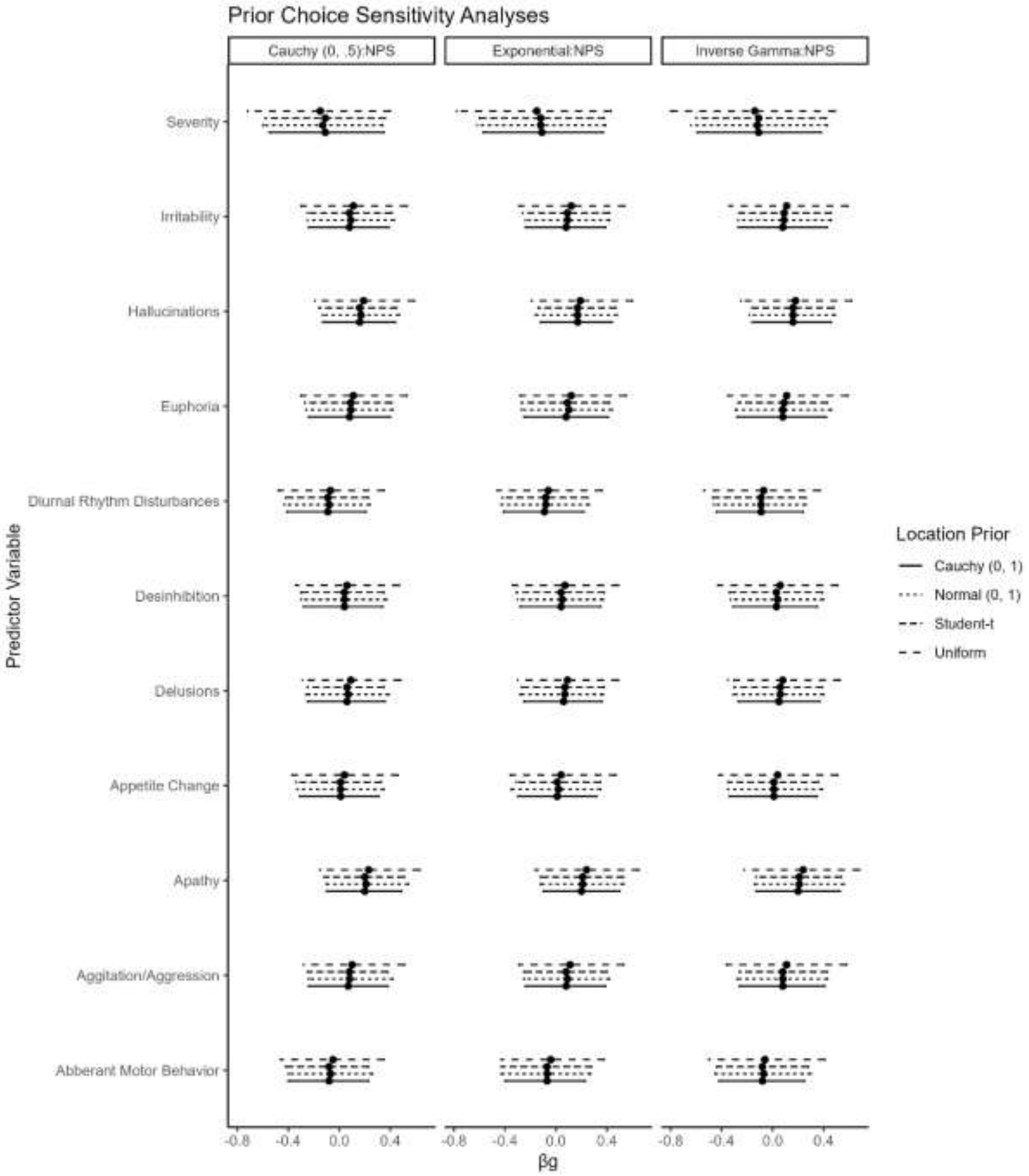

Figure 1. Regression coefficients with 95% confidence intervals for the Neuropsychiatric Symptoms Domains model. Severity: difference in dementia severity between dementia groups.

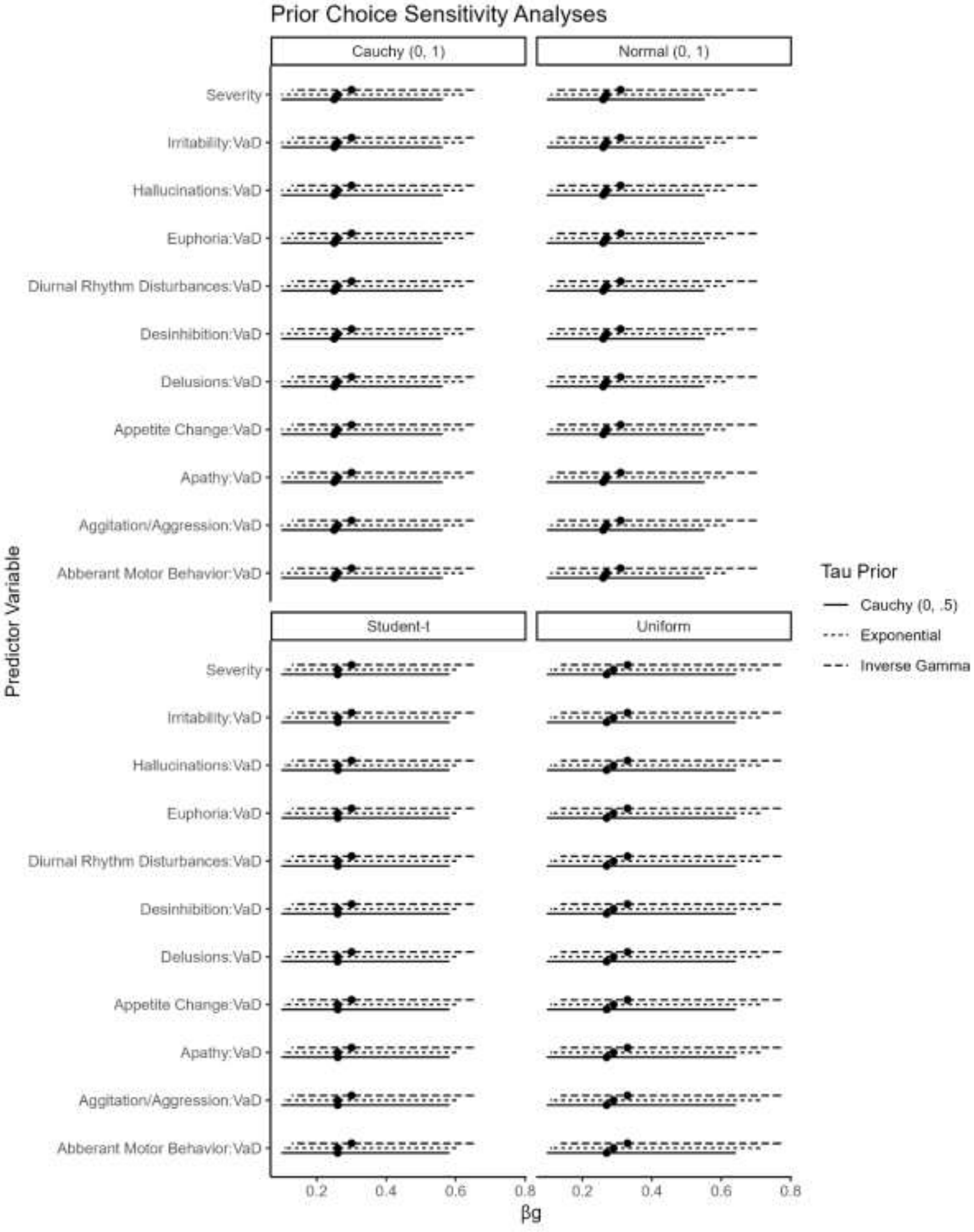

Figure 1. Study level standard deviation estimates with 95% confidence intervals for the Neuropsychiatric Symptoms Domains model. Severity: difference in dementia severity between dementia groups.

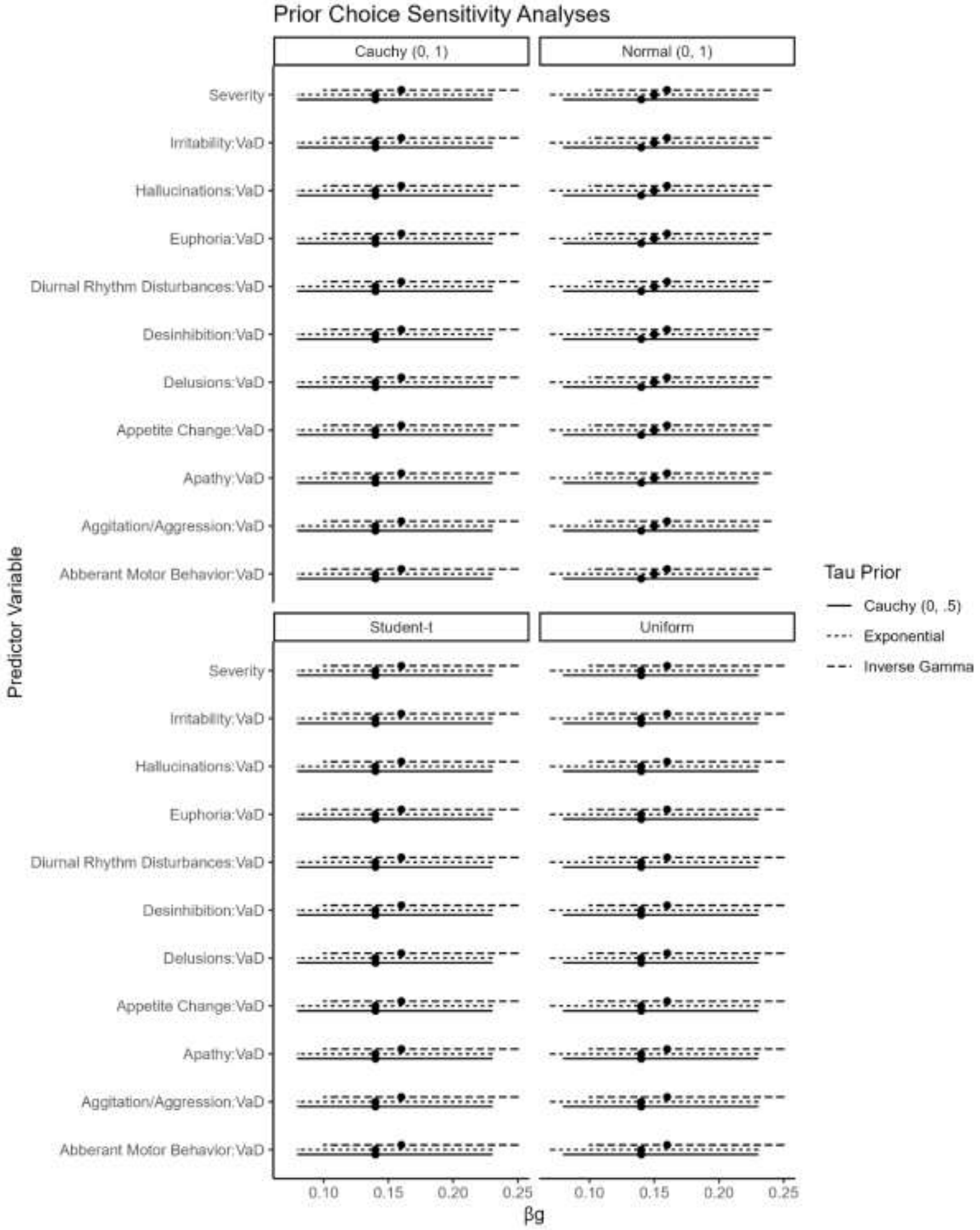

Figure 1. Effect size level standard deviation estimates with 95% confidence intervals for the Neuropsychiatric Symptoms Domains model. Severity: difference in dementia severity between dementia groups.

Total and Domain Scores: Quality Sensitivity Analysis

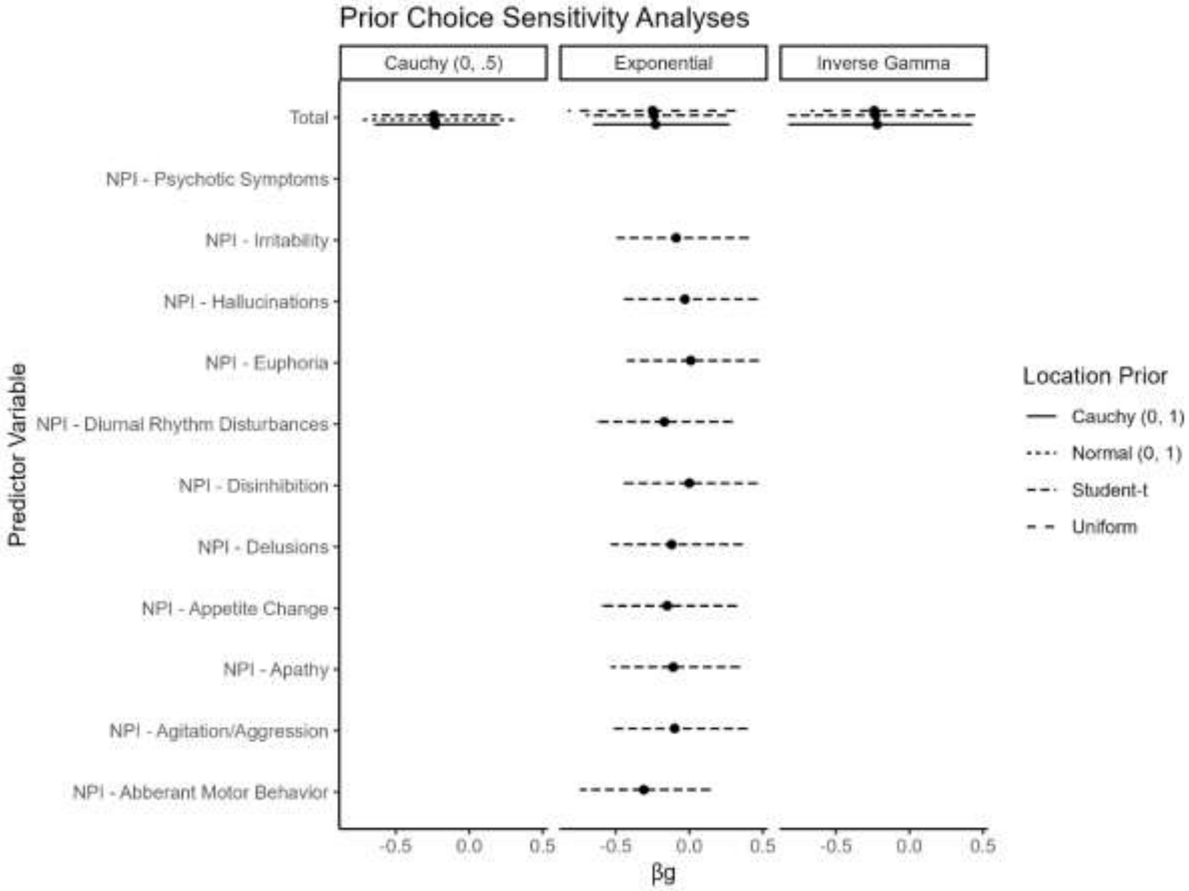

Figure 1. Regression coefficients with 95% confidence intervals for the Neuropsychiatric Symptoms study quality sensitivity analyses. Total: total scores on the Neuropsychiatric Inventory (NPI) and the Dementia Psychosis Scale.

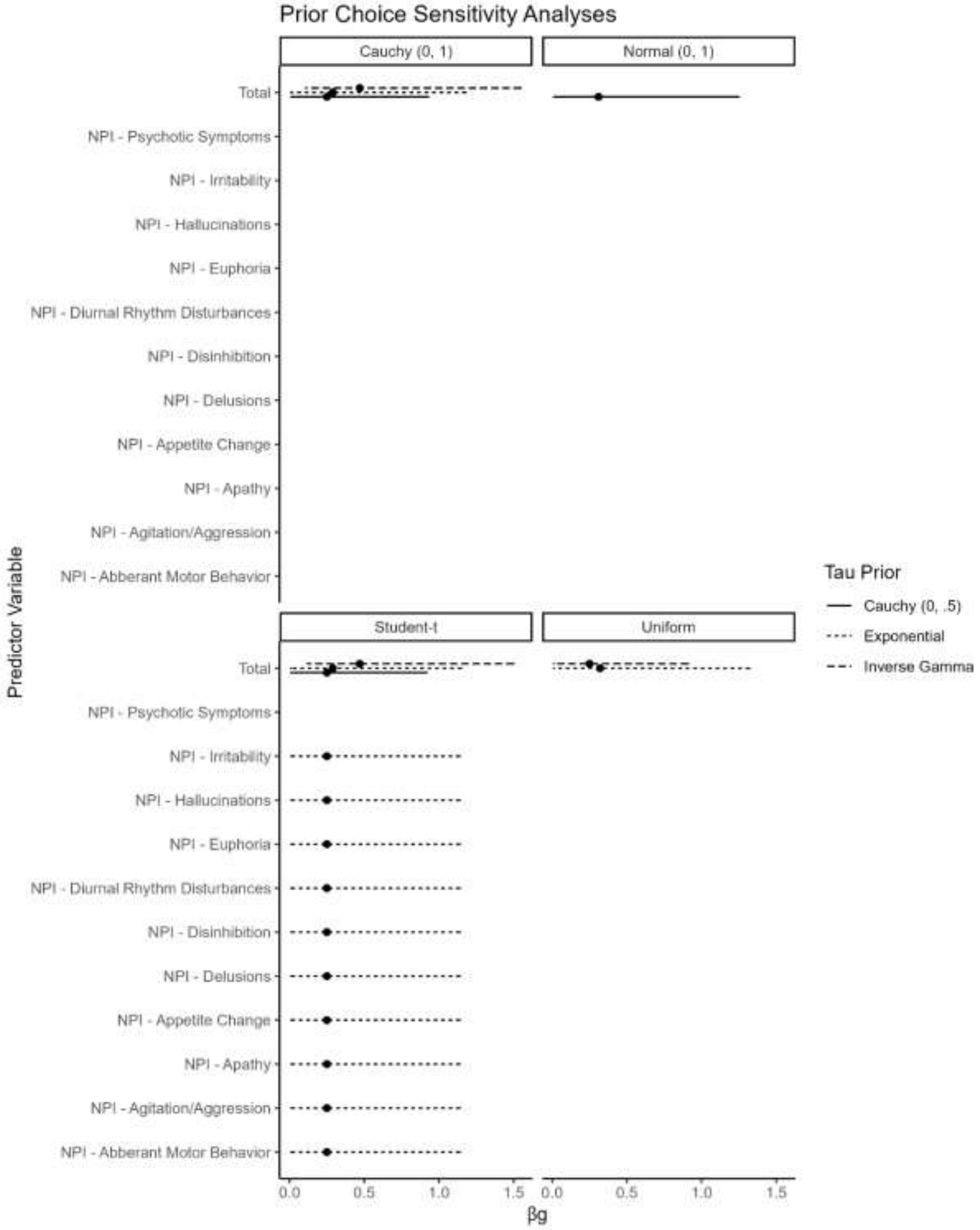

Figure 1. Study level standard deviation estimates with 95% confidence intervals for the Neuropsychiatric Symptoms study quality sensitivity analyses. Total: total scores on the Neuropsychiatric Inventory (NPI) and the Dementia Psychosis Scale.

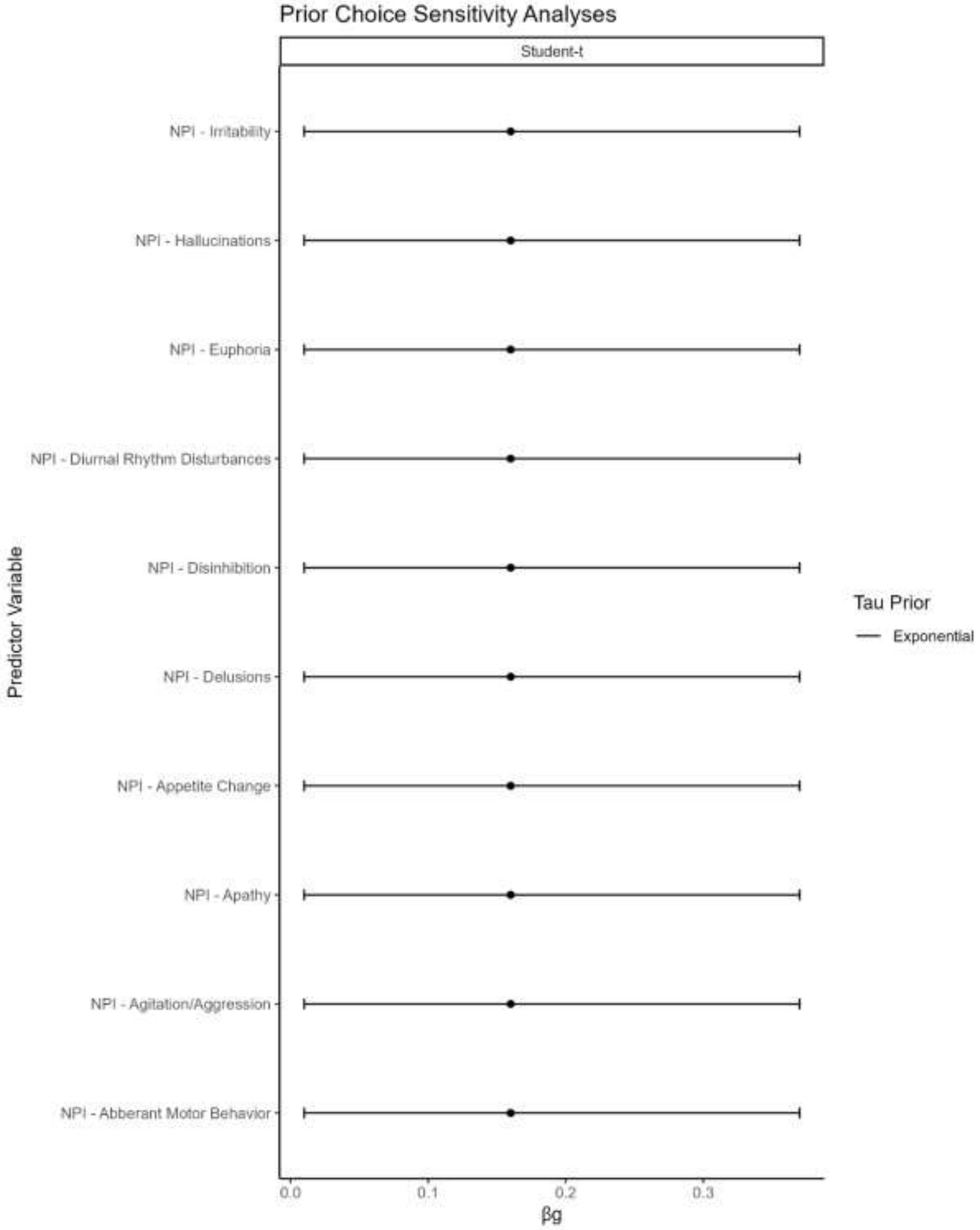

Figure 1. Effect size level standard deviation estimates with 95% confidence intervals for the Neuropsychiatric Symptoms study quality sensitivity analyses. Total: total scores on the Neuropsychiatric Inventory (NPI) and the Dementia Psychosis Scale.

Apraxia

Apraxia

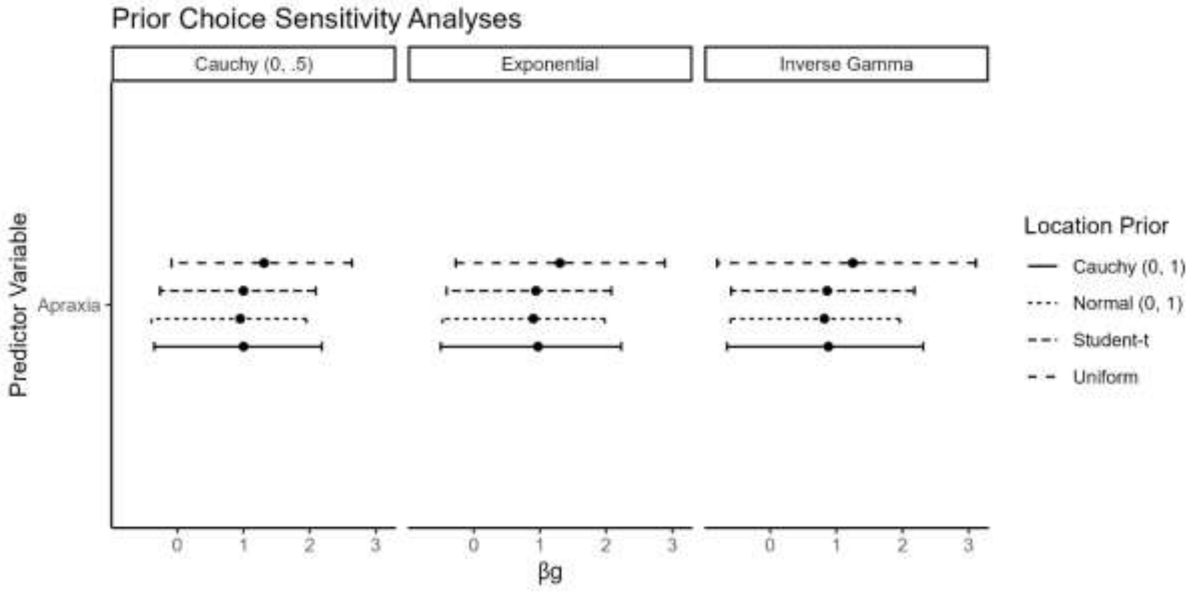

Figure 1. Regression coefficients with 95% confidence intervals for the Apraxia model.

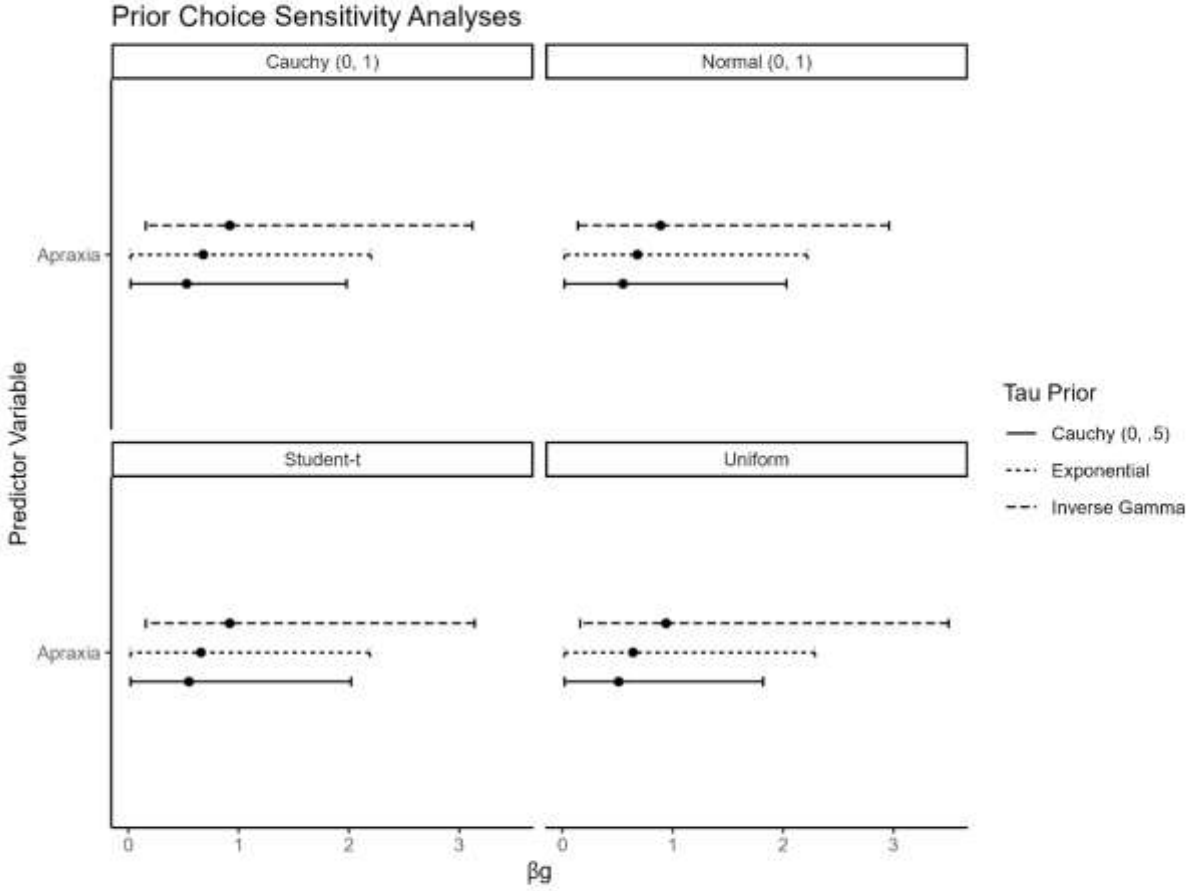

Figure 1. Study level standard deviation estimates with 95% confidence intervals for the Apraxia model.

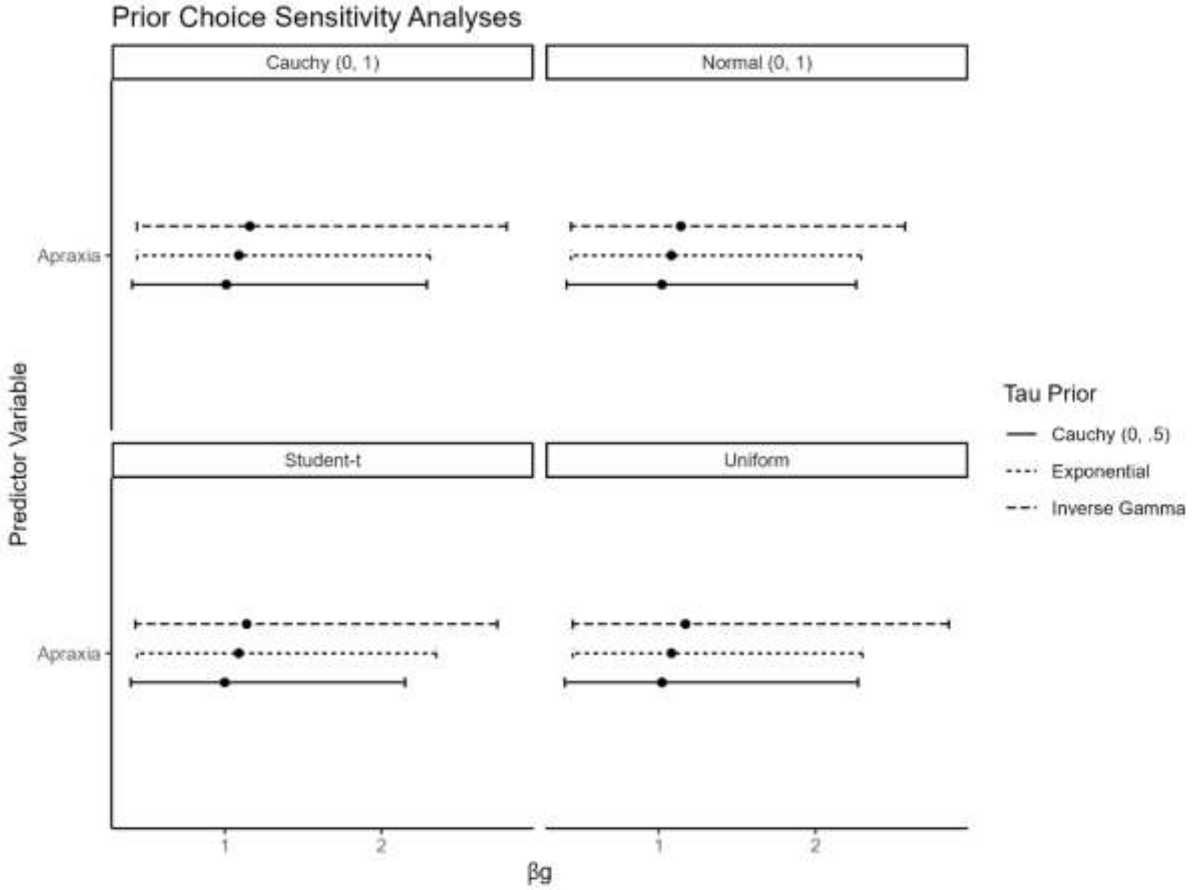

Figure 1. Effect size level standard deviation estimates with 95% confidence intervals for the Apraxia model.

Facial Apraxia

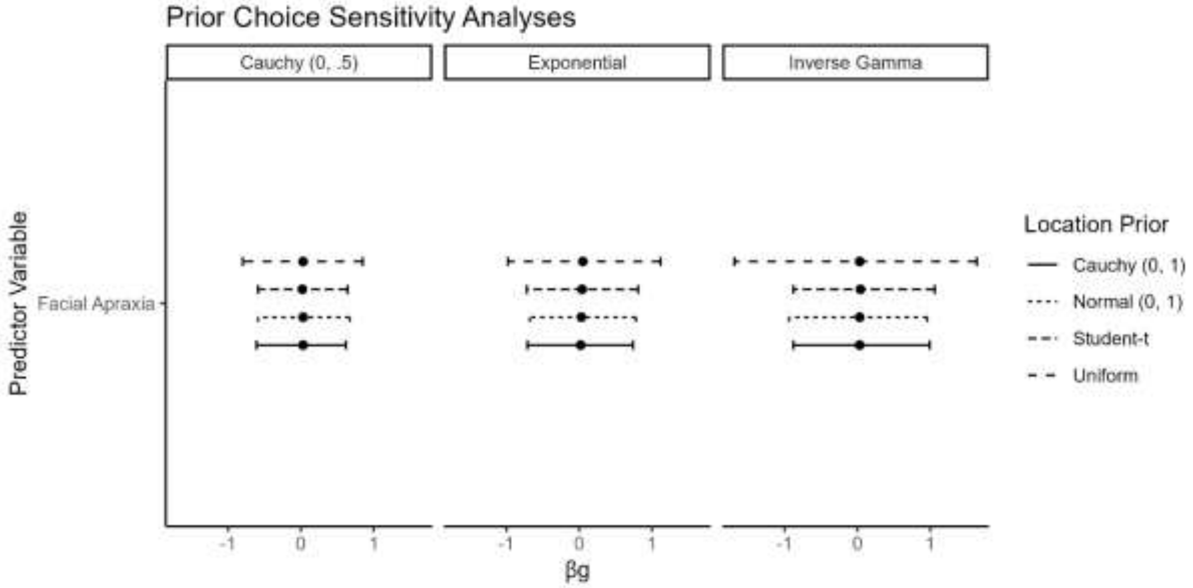

Figure 1. Regression coefficients with 95% confidence intervals for the Facial Apraxia model.

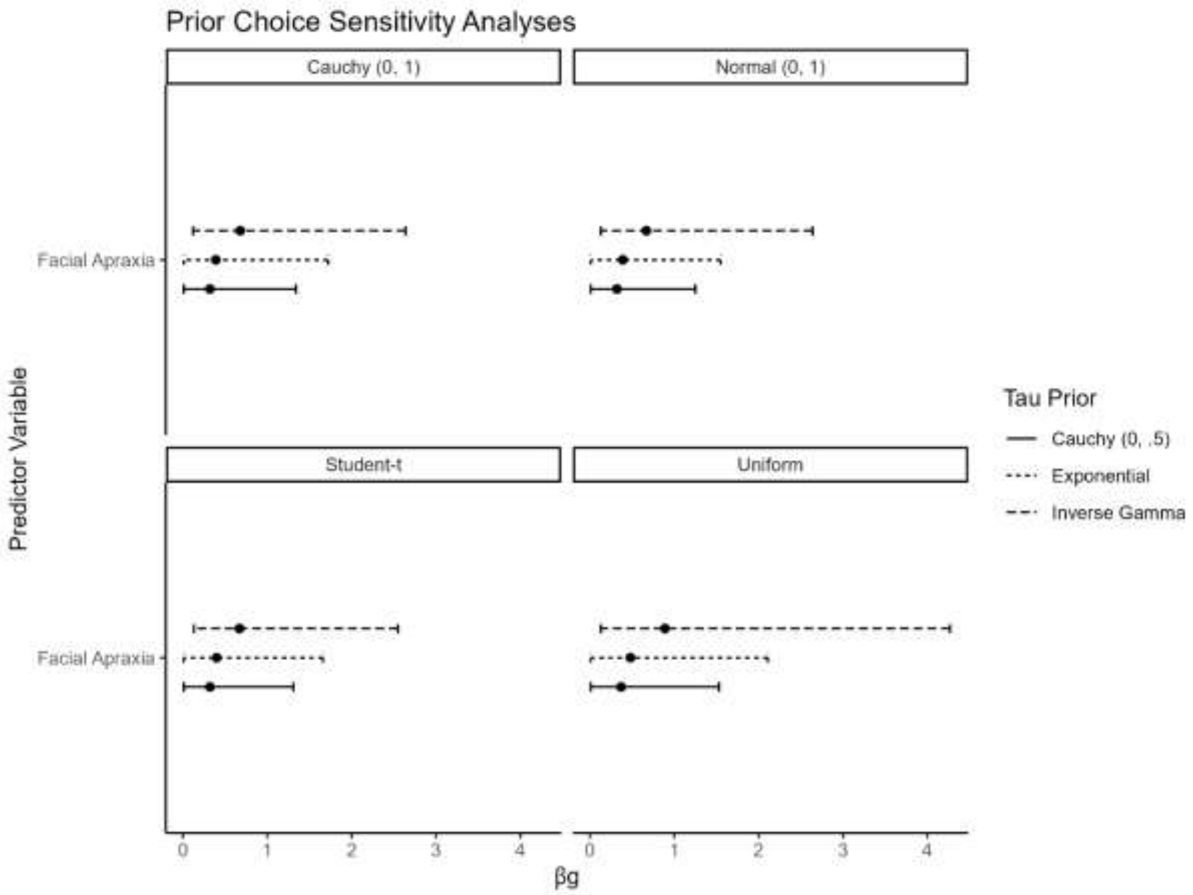

Figure 1. Study level standard deviation estimates with 95% confidence intervals for the Facial Apraxia model.

Ideomotor Apraxia

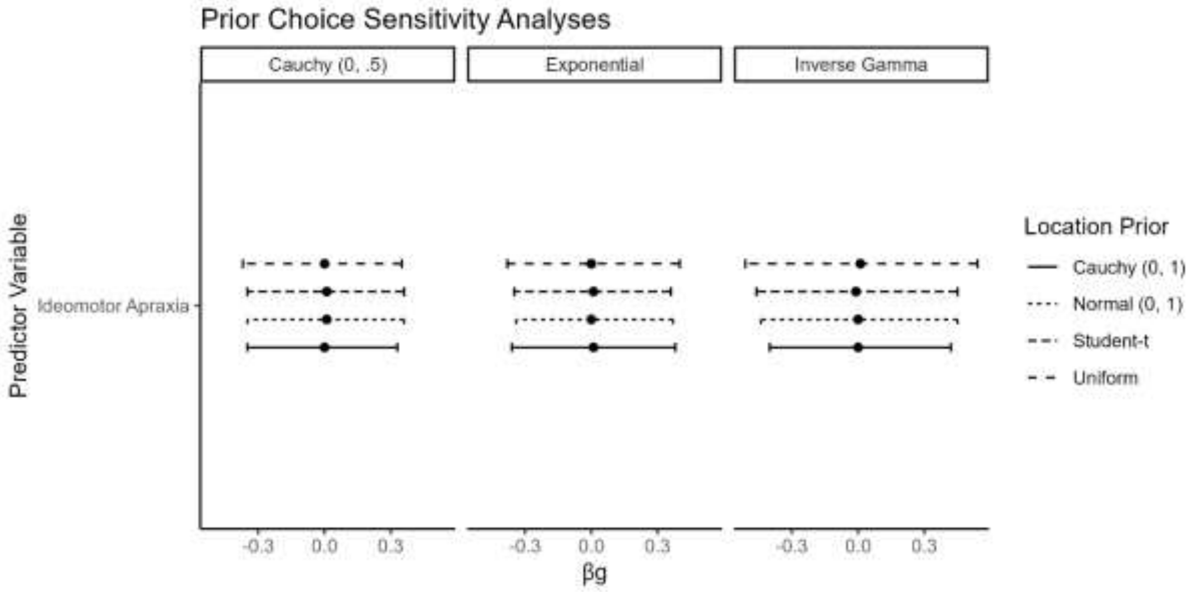

Figure 1. Regression coefficients with 95% confidence intervals for the Ideomotor Apraxia.

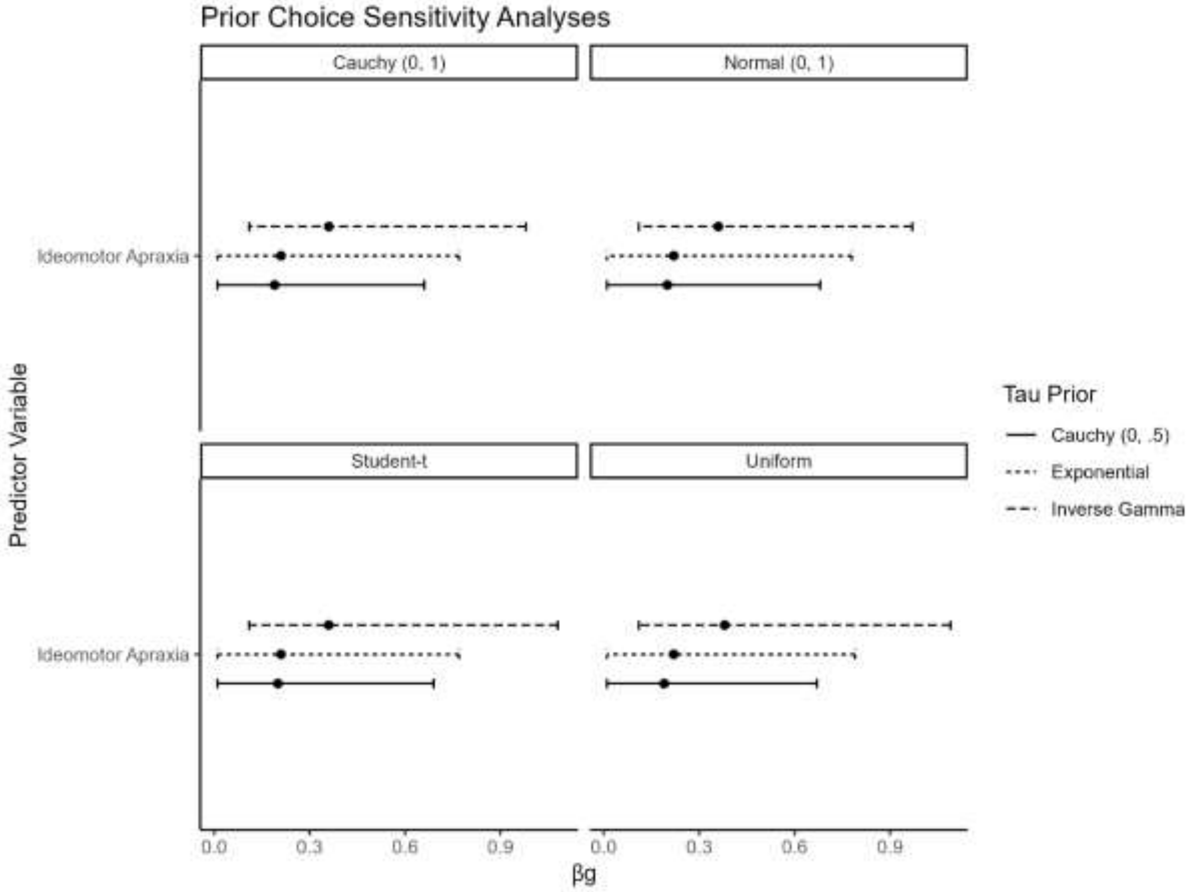

Figure 1. Study level standard deviation estimates with 95% confidence intervals for the Ideomotor Apraxia.

Motor Functioning

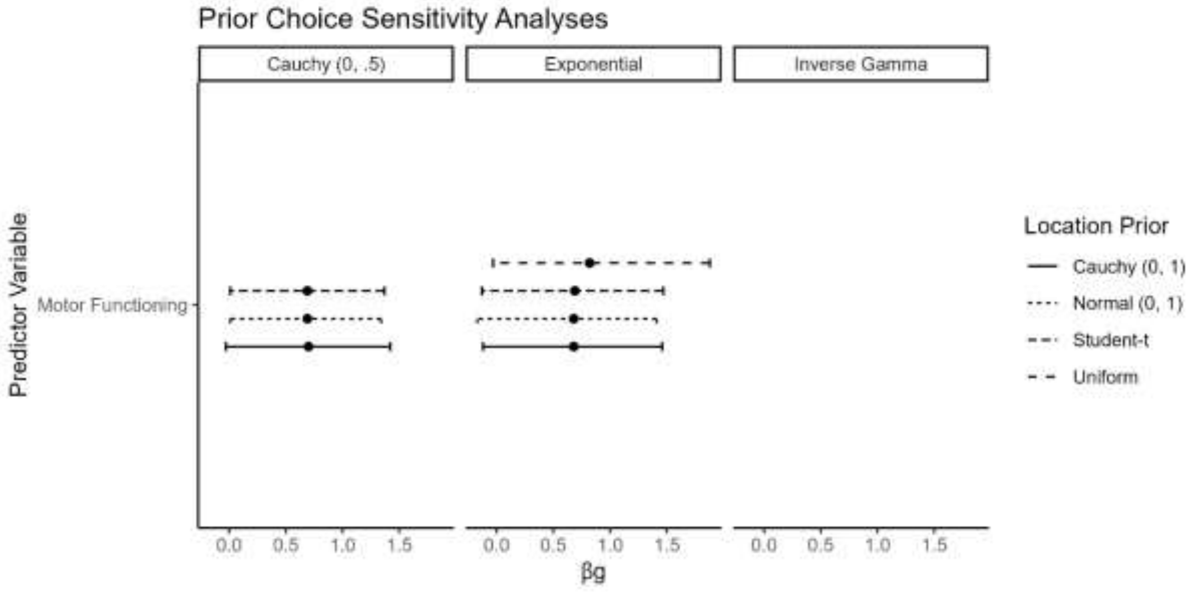

Figure 1. Regression coefficients with 95% confidence intervals for the impairments of Motor Functioning model.

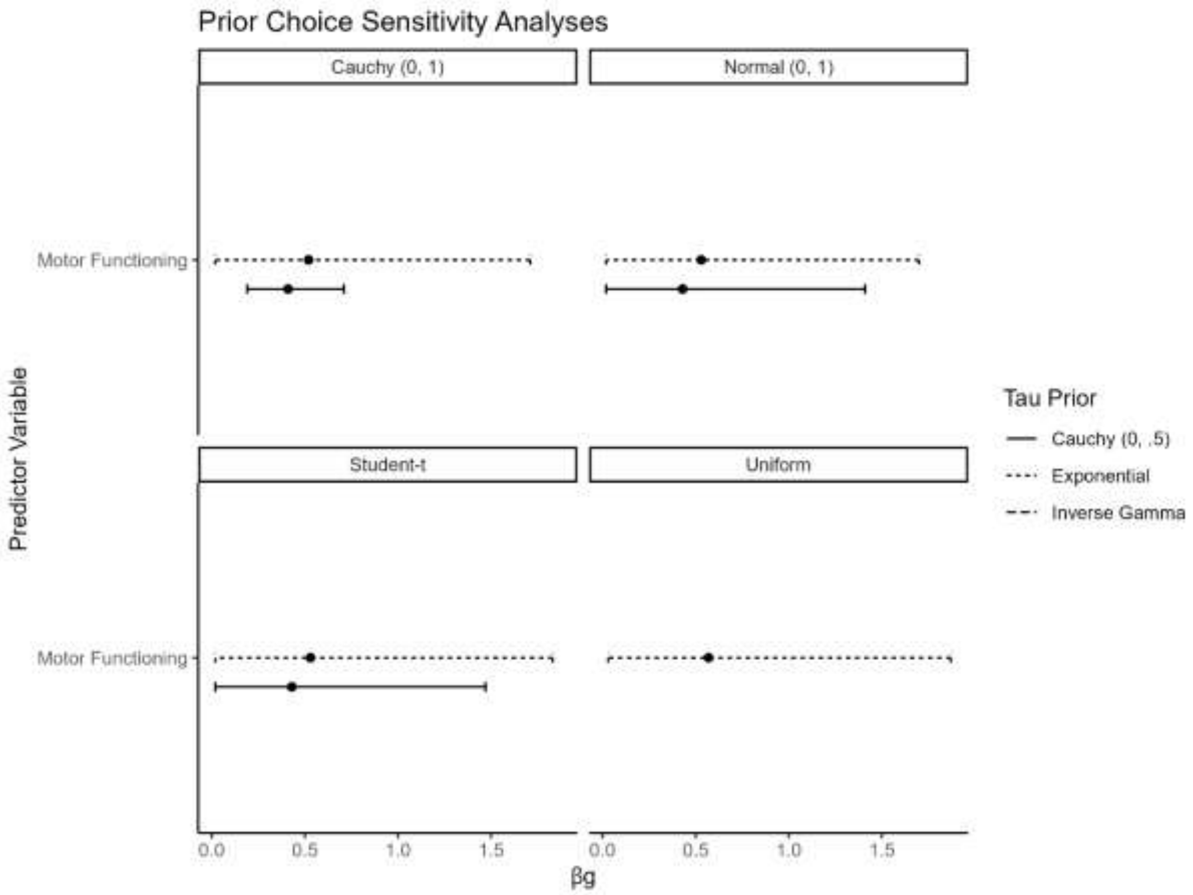

Figure 1. Study level standard deviation estimates with 95% confidence intervals for the impairments of Motor Functioning model.

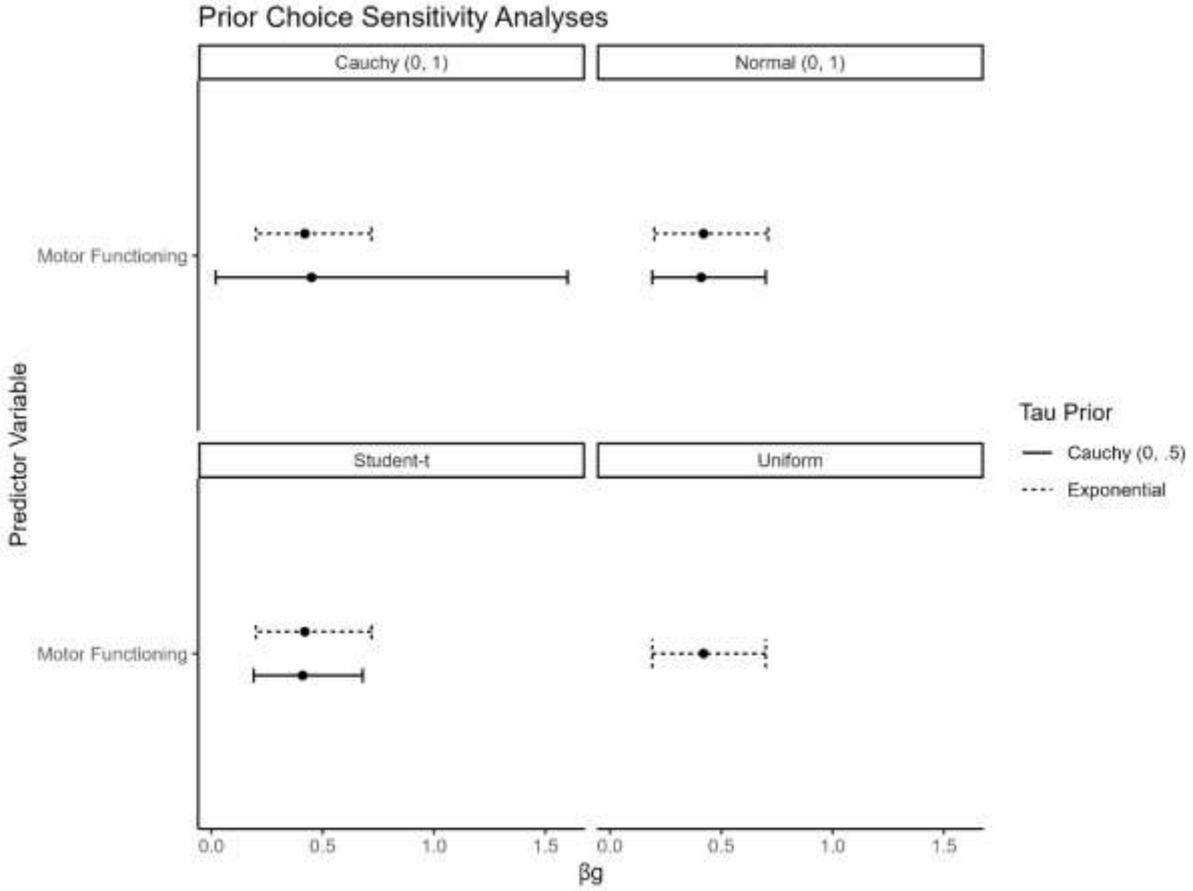

Figure 1. Effect size level standard deviation estimates with 95% confidence intervals for the impairments of Motor Functioning model.

Visuo-Spatial Processing

WAIS: Block Design, Object Assembly, Picture Completion

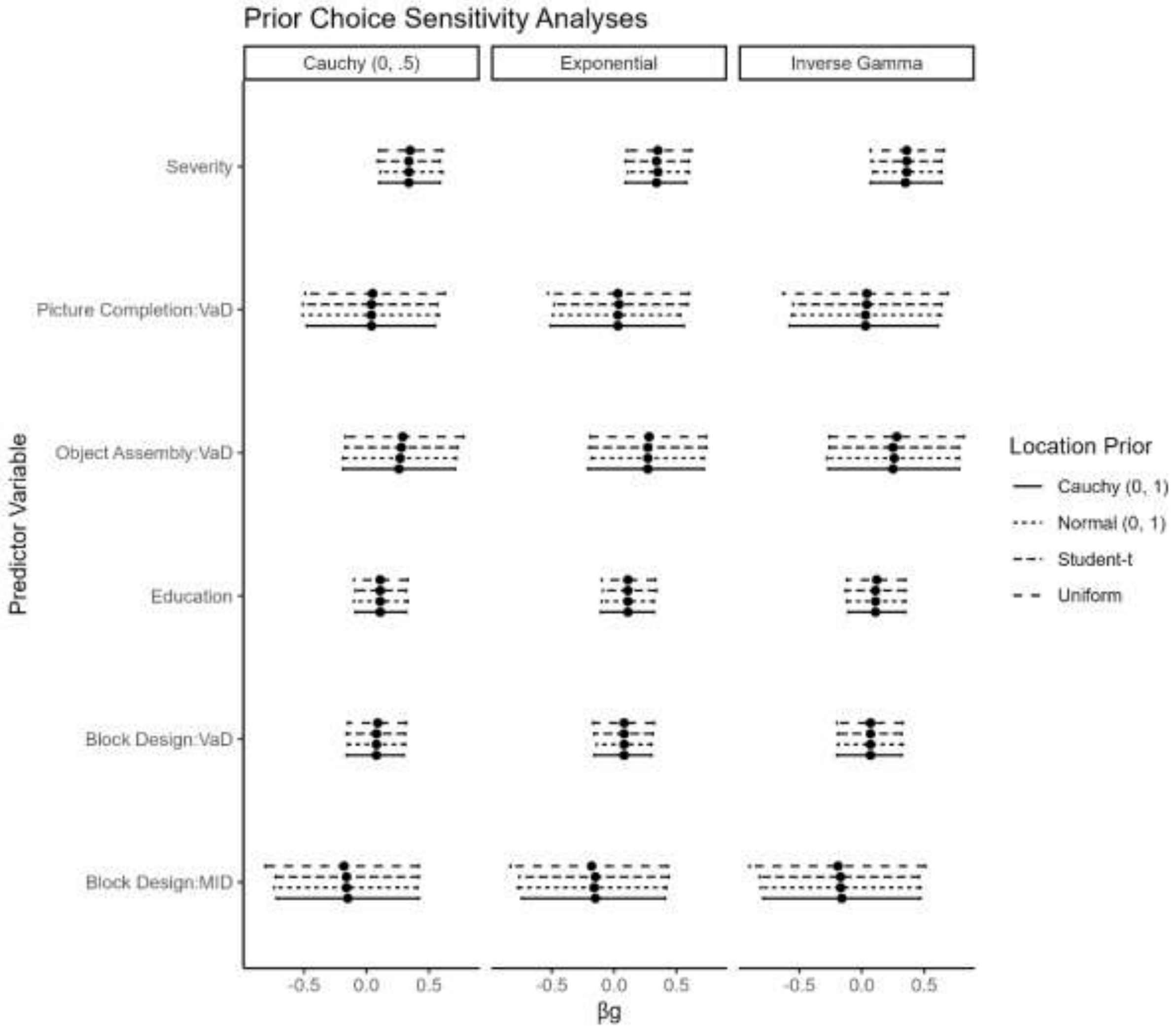

Figure 1. Regression coefficients with 95% confidence intervals for the Wechsler Adult Intelligence Scale (WAIS) visuo-spatial processing subtests model. VaD: vascular dementia, MID: multi-infarct dementia, Severity: difference in dementia severity between dementia groups, Education: difference in average years of education between dementia groups.

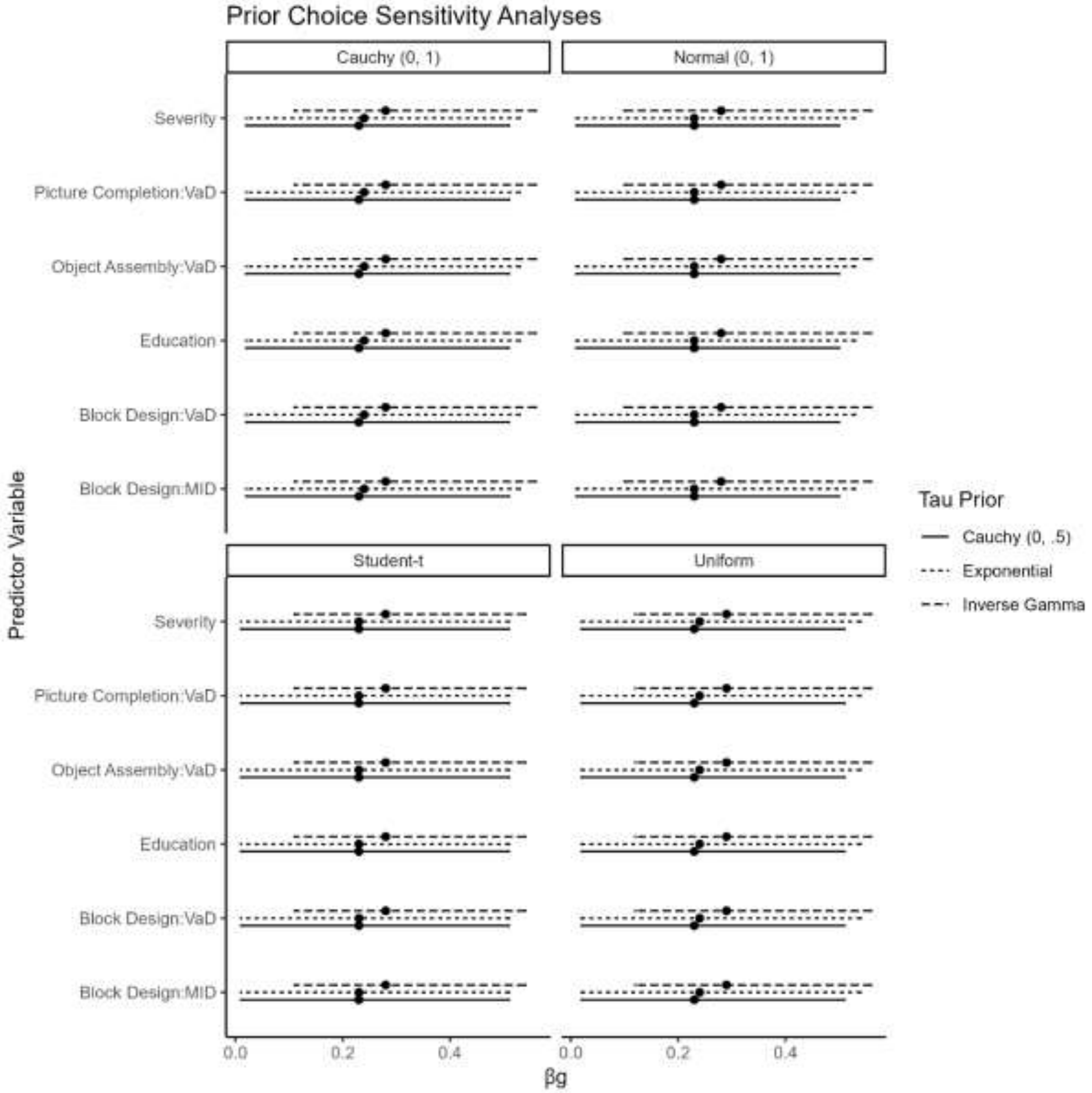

Figure 1. Study level standard deviation estimates with 95% confidence intervals for the Wechsler Adult Intelligence Scale (WAIS) visuo-spatial processing subtests model. VaD: vascular dementia, MID: multi-infarct dementia, Severity: difference in dementia severity between dementia groups, Education: difference in average years of education between dementia groups.

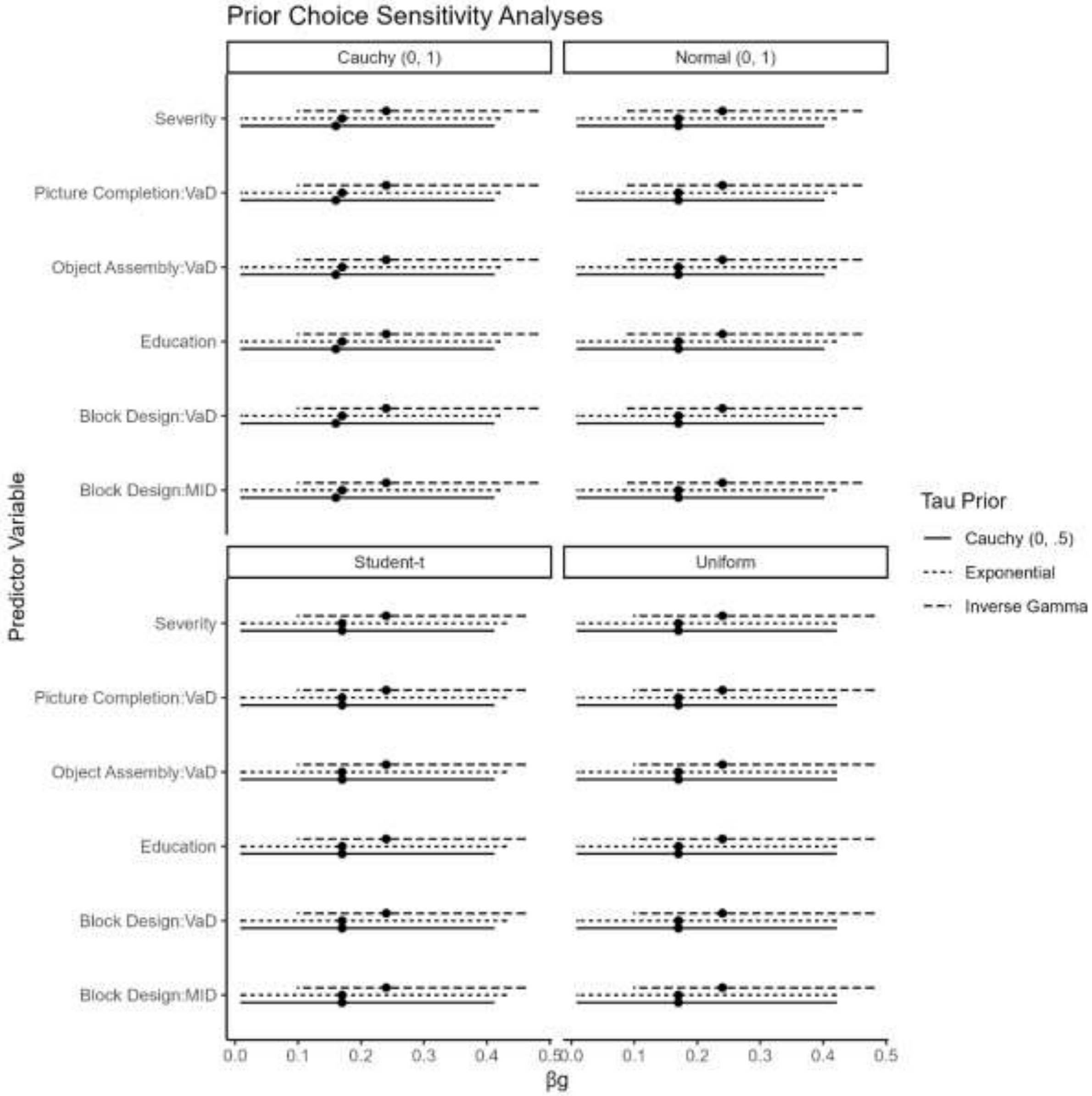

Figure 1. Effect size level standard deviation estimates with 95% confidence intervals for the Wechsler Adult Intelligence Scale (WAIS) visuo-spatial processing subtests model. VaD: vascular dementia, MID: multi-infarct dementia, Severity: difference in dementia severity between dementia groups, Education: difference in average years of education between dementia groups.

Clock Drawing Test: Clock Copying, Clock Drawing, Clock Reading

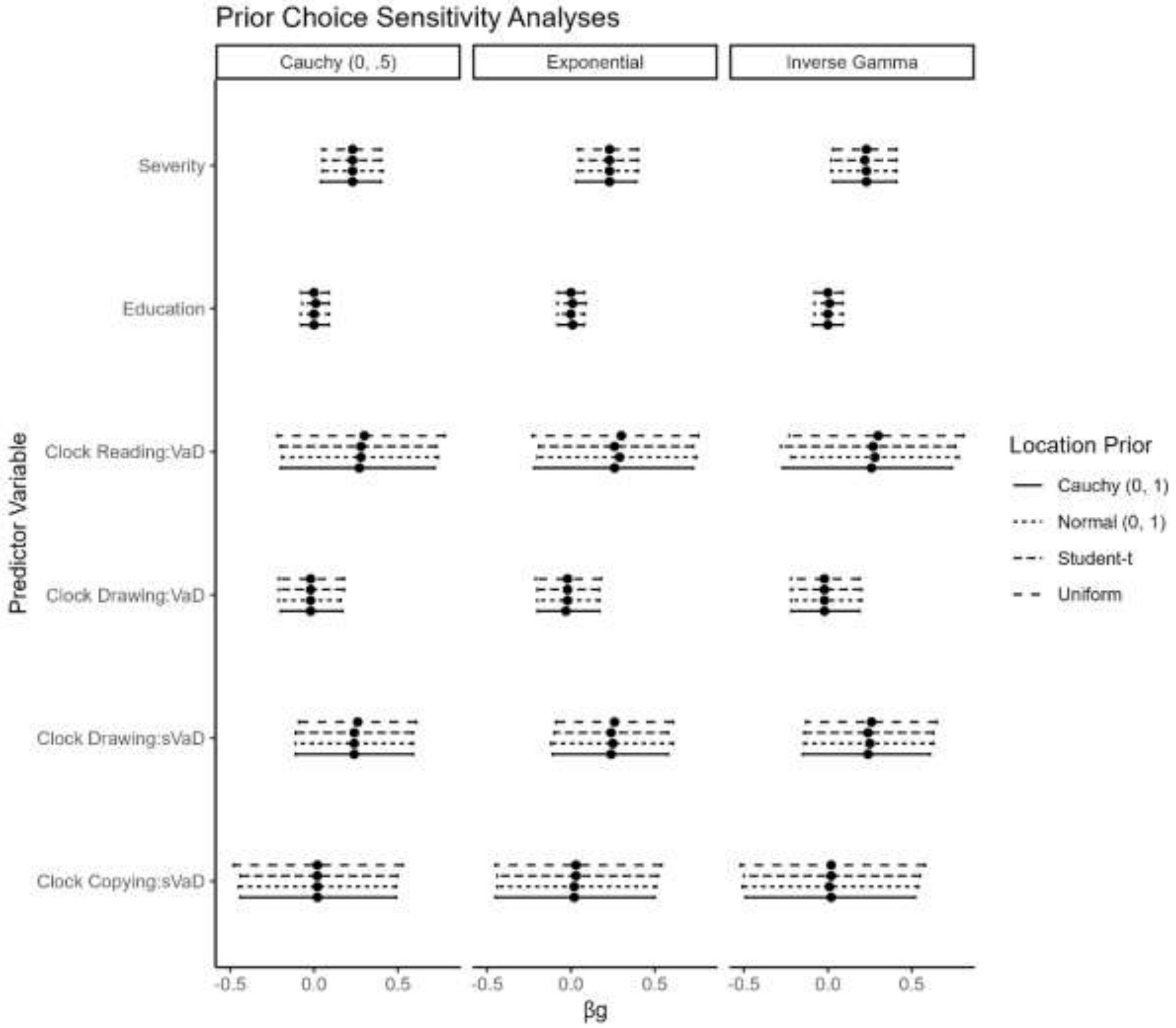

Figure 1. Regression coefficients with 95% confidence intervals for the Clock Drawing Test (CDT) model. sVaD: subcortical vascular dementia, VaD: vascular dementia, Severity: difference in dementia severity between dementia groups, Education: difference in average years of education between dementia groups.

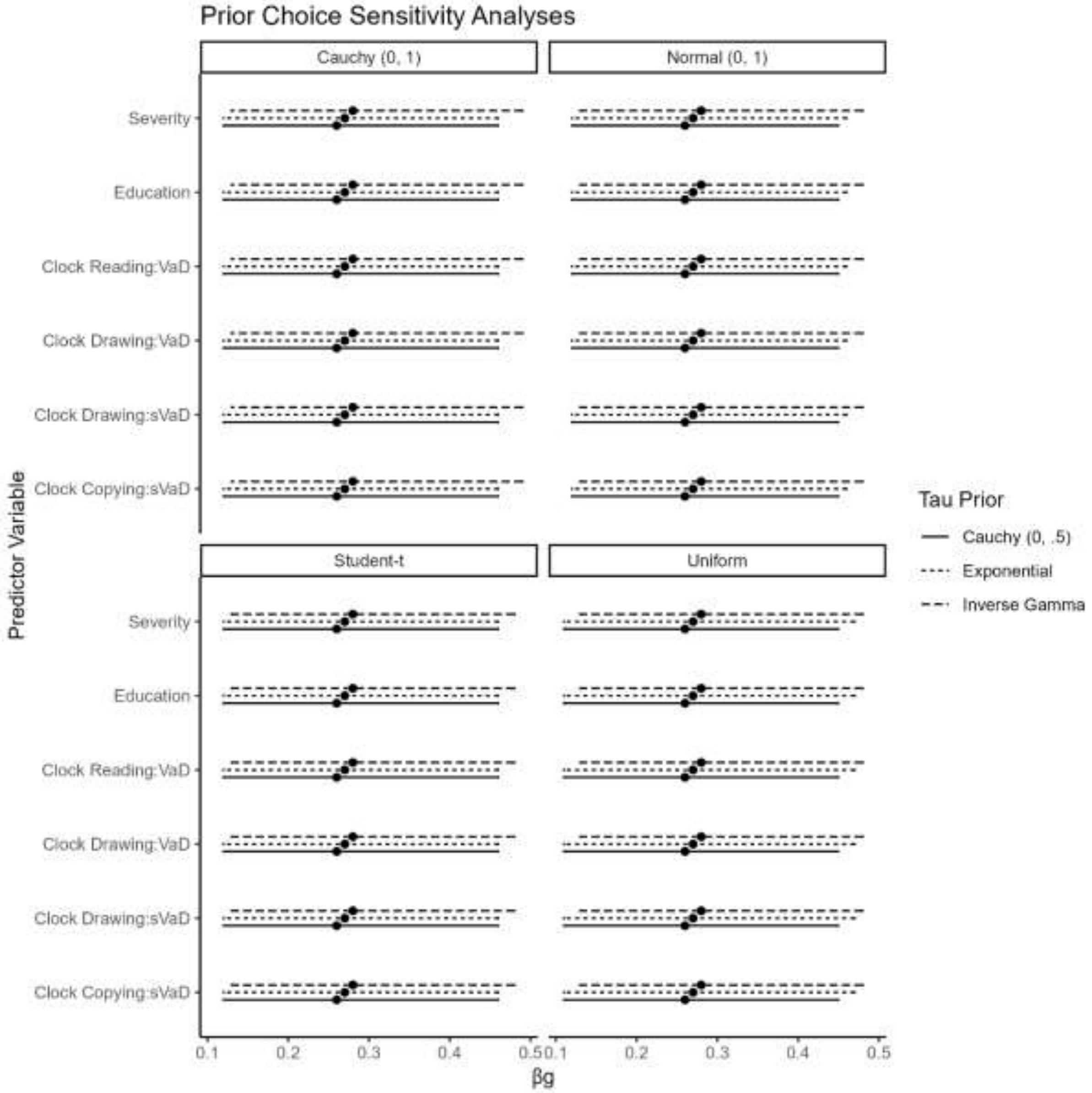

Figure 1. Study level standard deviation estimates with 95% confidence intervals for the Clock Drawing Test (CDT) model. sVaD: subcortical vascular dementia, VaD: vascular dementia, Severity: difference in dementia severity between dementia groups, Education: difference in average years of education between dementia groups.

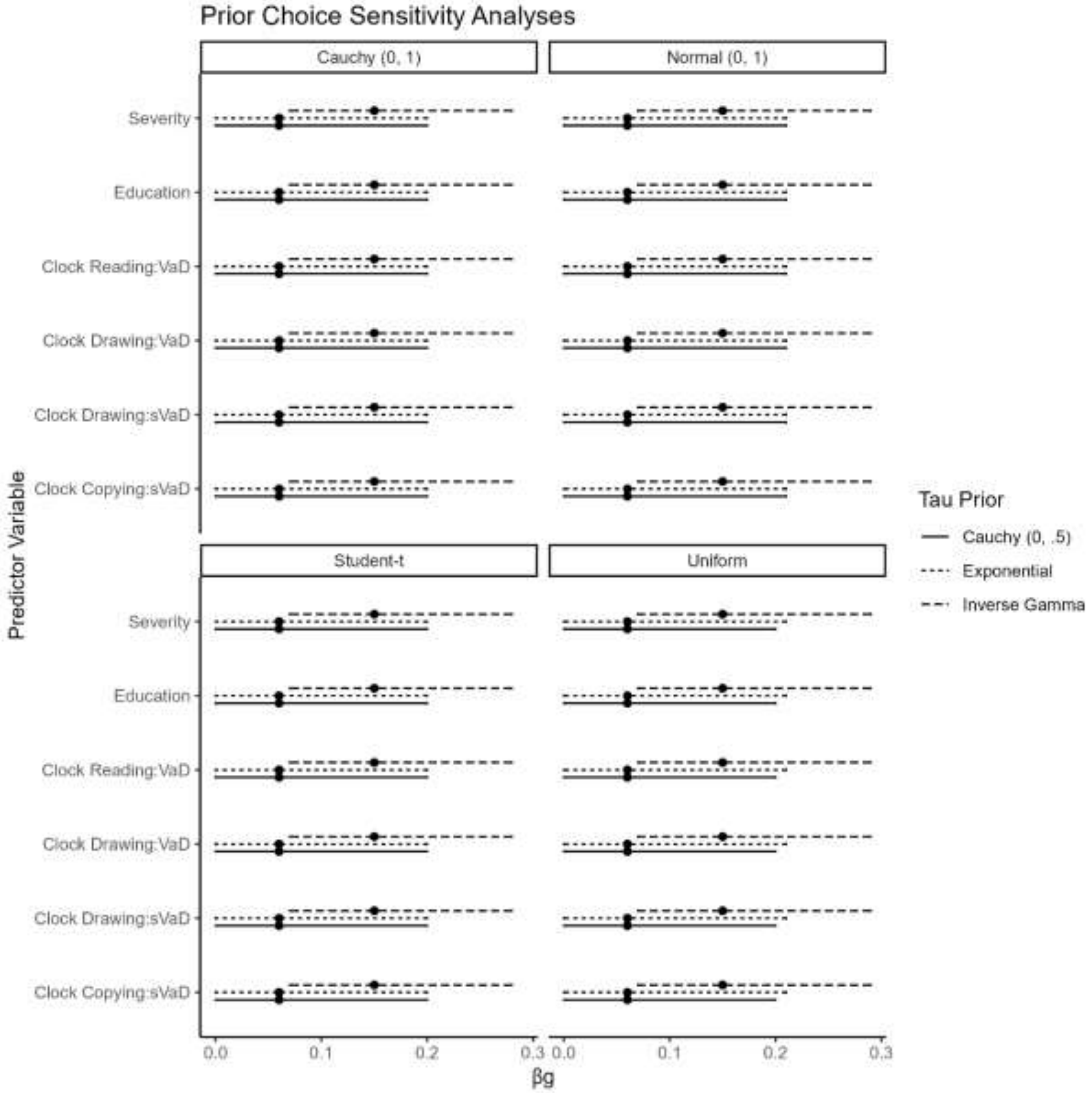

Figure 1. Effect size level standard deviation estimates with 95% confidence intervals for the Clock Drawing Test (CDT) model. sVaD: subcortical vascular dementia, VaD: vascular dementia, Severity: difference in dementia severity between dementia groups, Education: difference in average years of education between dementia groups.

Rey-Osterrieth Complex Figure Test

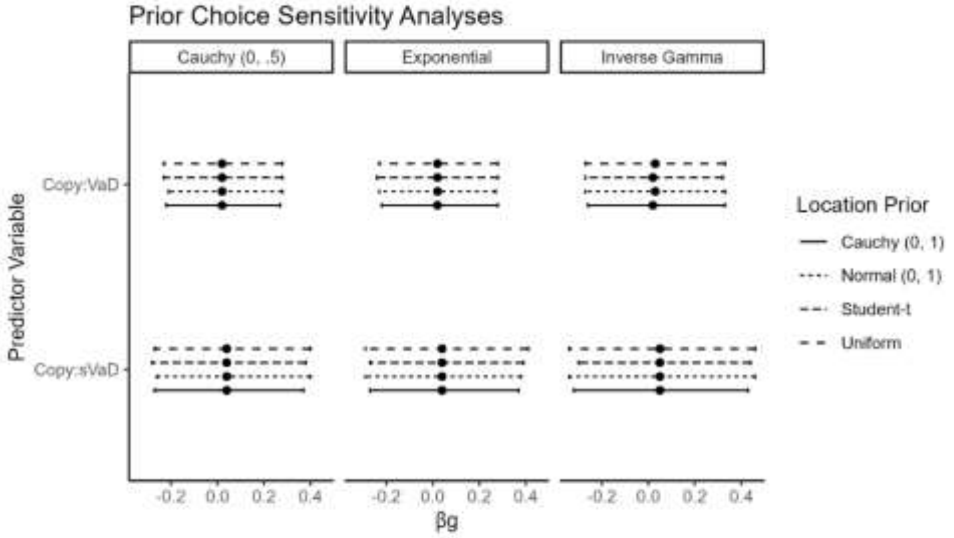

Figure 1. Regression coefficients with 95% confidence intervals for the Rey-Osterrieth Complex Figure Test: Figure Copy subtests model. sVaD: subcortical vascular dementia, VaD: vascular dementia.

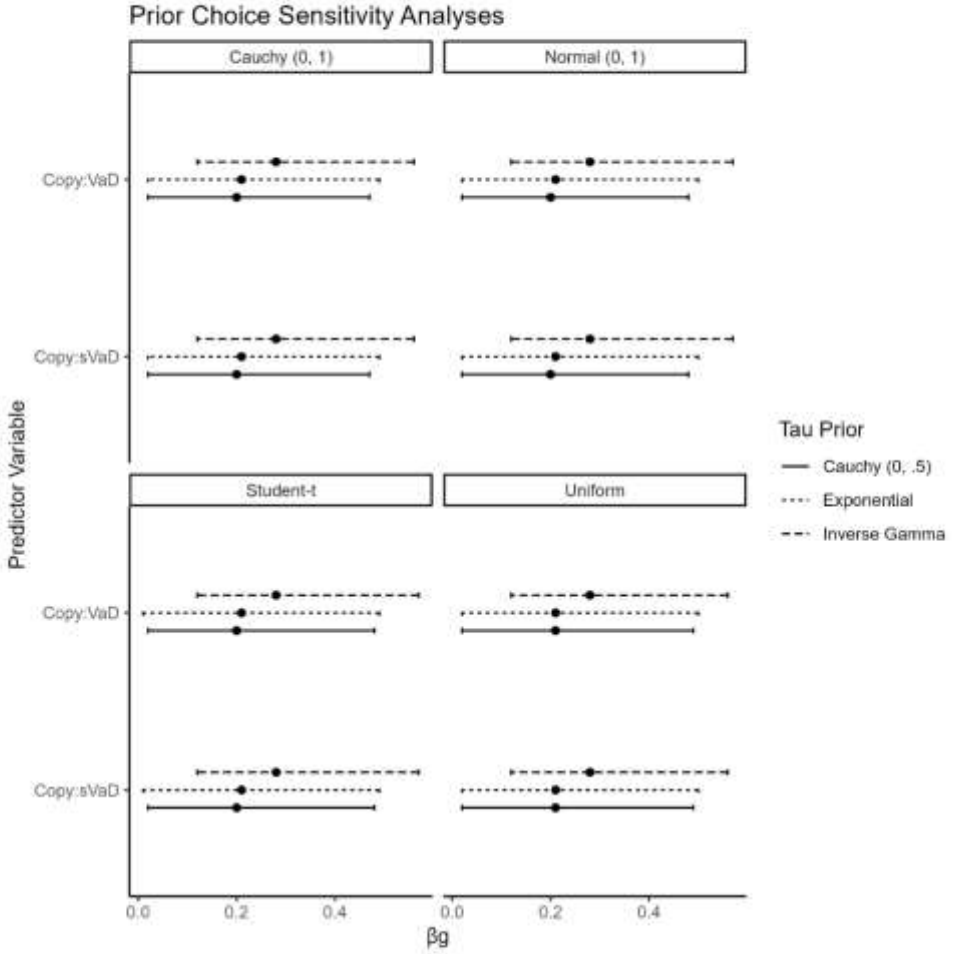

Figure 1. Study level standard deviation estimates with 95% confidence intervals for the Rey-Osterrieth Complex Figure Test: Figure Copy subtests model. sVaD: subcortical vascular dementia, VaD: vascular dementia.

Visual Object and Spatial Processing Test Battery (VOSP)

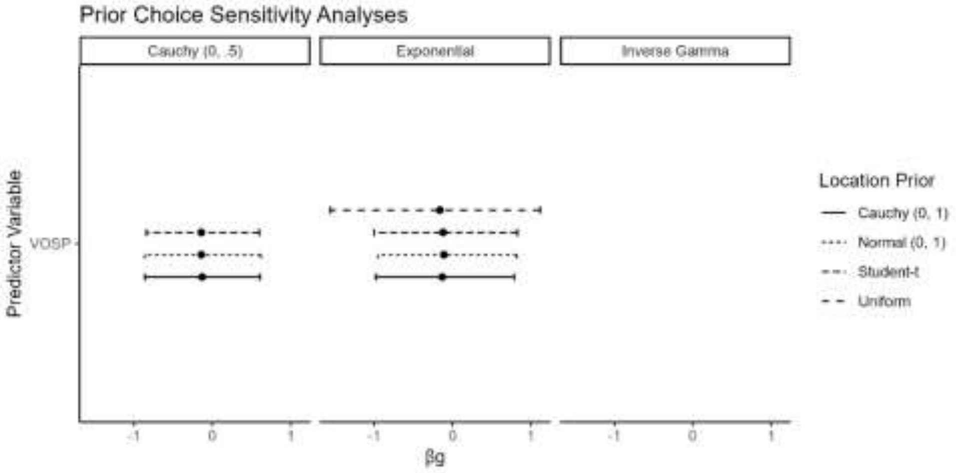

Figure 1. Regression coefficients with 95% confidence intervals for the Visual Object and Spatial Processing Test Battery (VOSP) model.

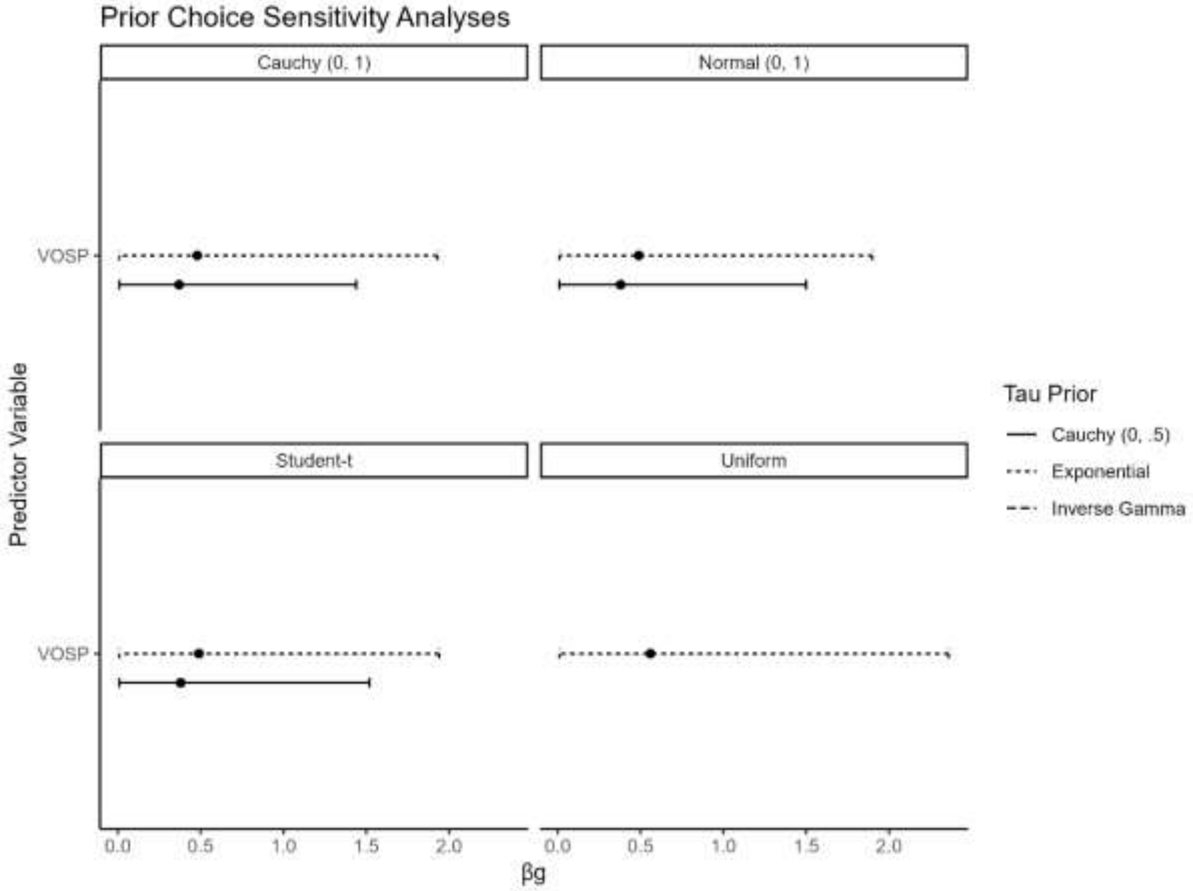

Figure 1. Study level standard deviation estimates with 95% confidence intervals for the Visual Object and Spatial Processing Test Battery (VOSP) model.

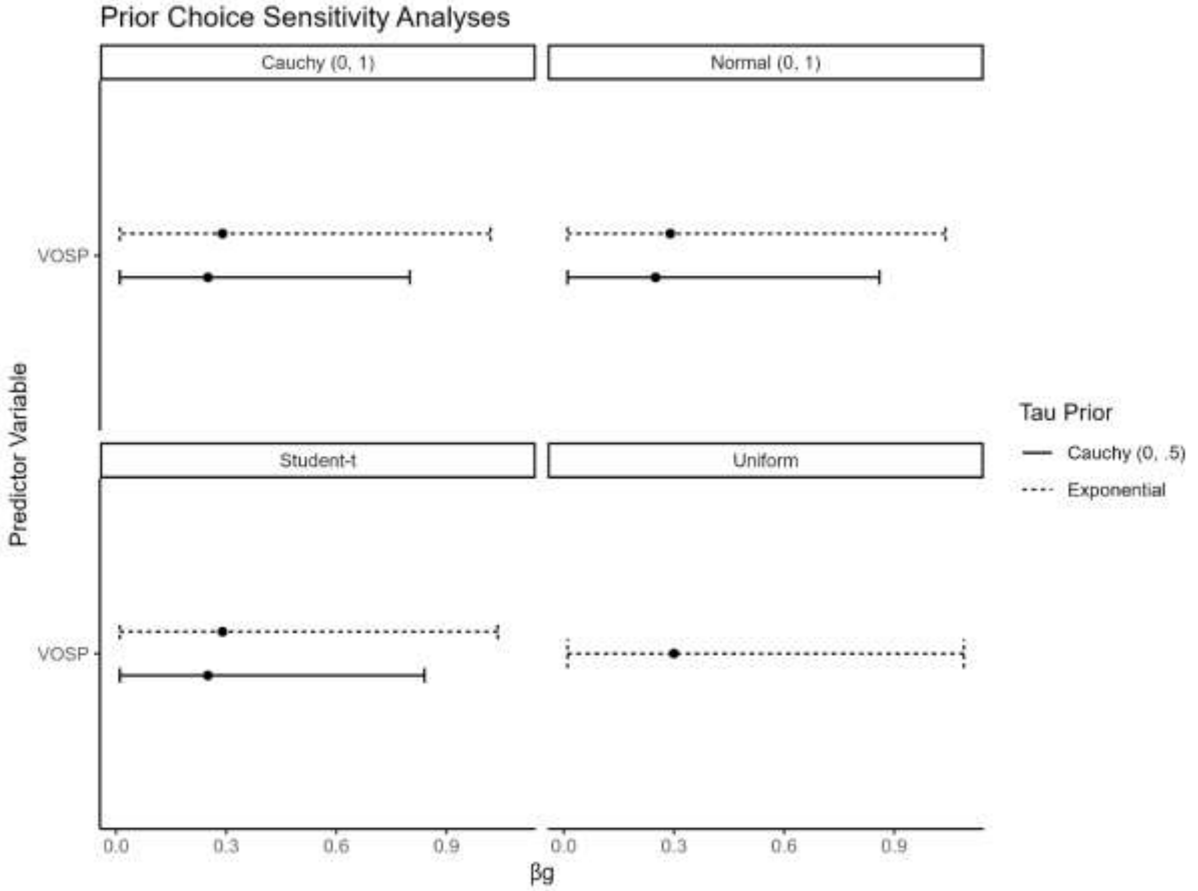

Figure 1. Effect size level standard deviation estimates with 95% confidence intervals for the Visual Object and Spatial Processing Test Battery (VOSP) model.

CERAD: Constructional Praxis

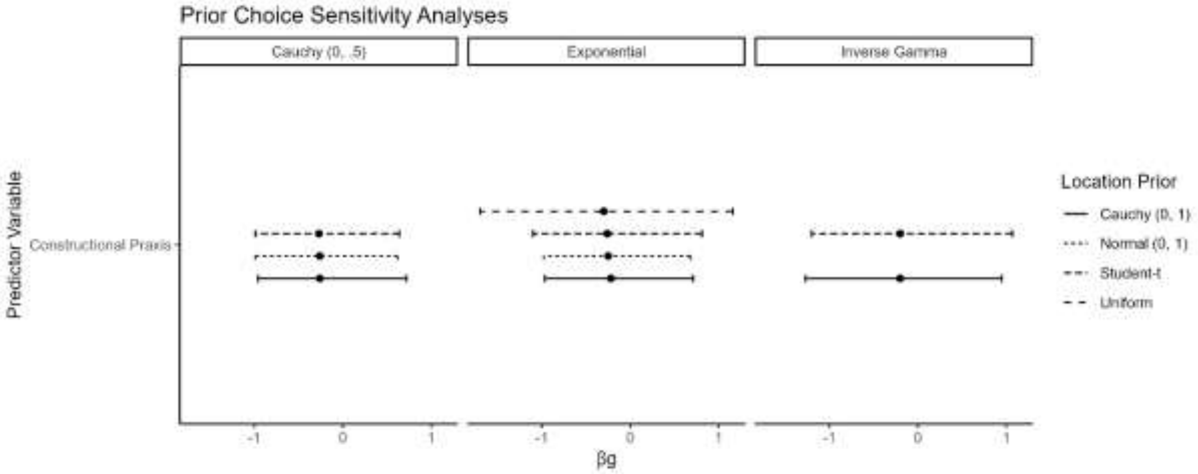

Figure 1. Regression coefficients with 95% confidence intervals for the Consortium to Establish a Registry for Alzheimer's Disease (CERAD) test battery Constructional Praxis subtest model.

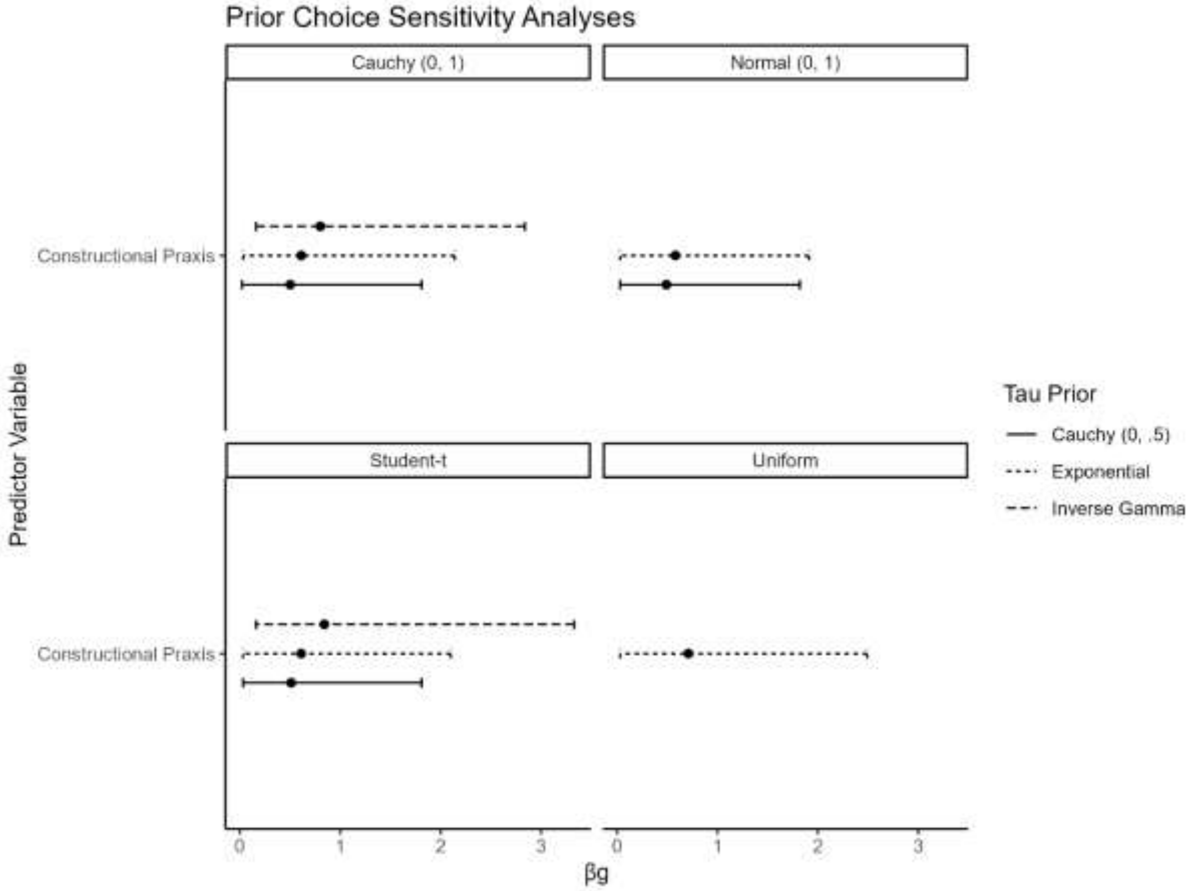

Figure 1. Study level standard deviation estimates with 95% confidence intervals for the Consortium to Establish a Registry for Alzheimer's Disease (CERAD) test battery Constructional Praxis subtest model.

Addenbrooke's Cognitive Examination - R

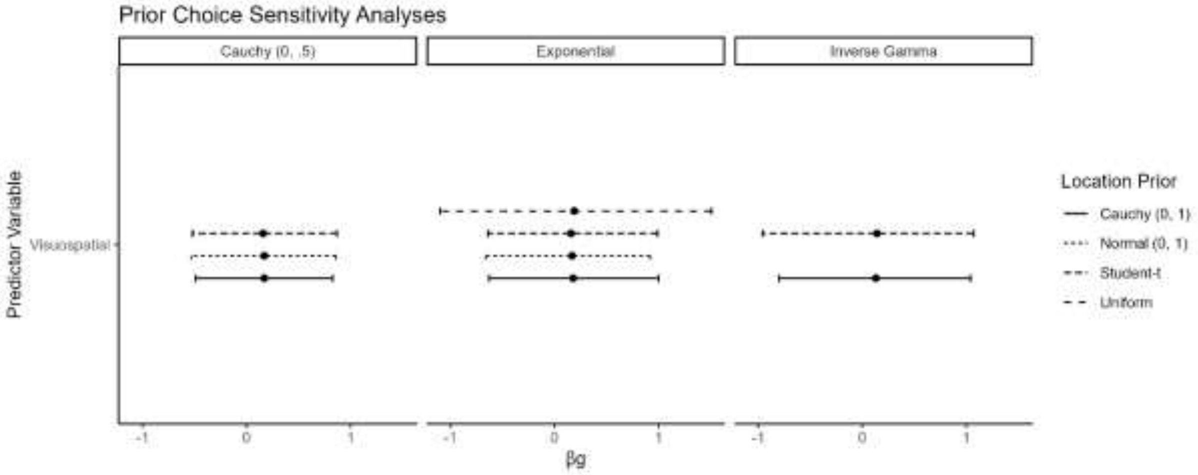

Figure 1. Regression coefficients with 95% confidence intervals for the Addenbrooke's Cognitive Examination: Visuospatial subtest model.

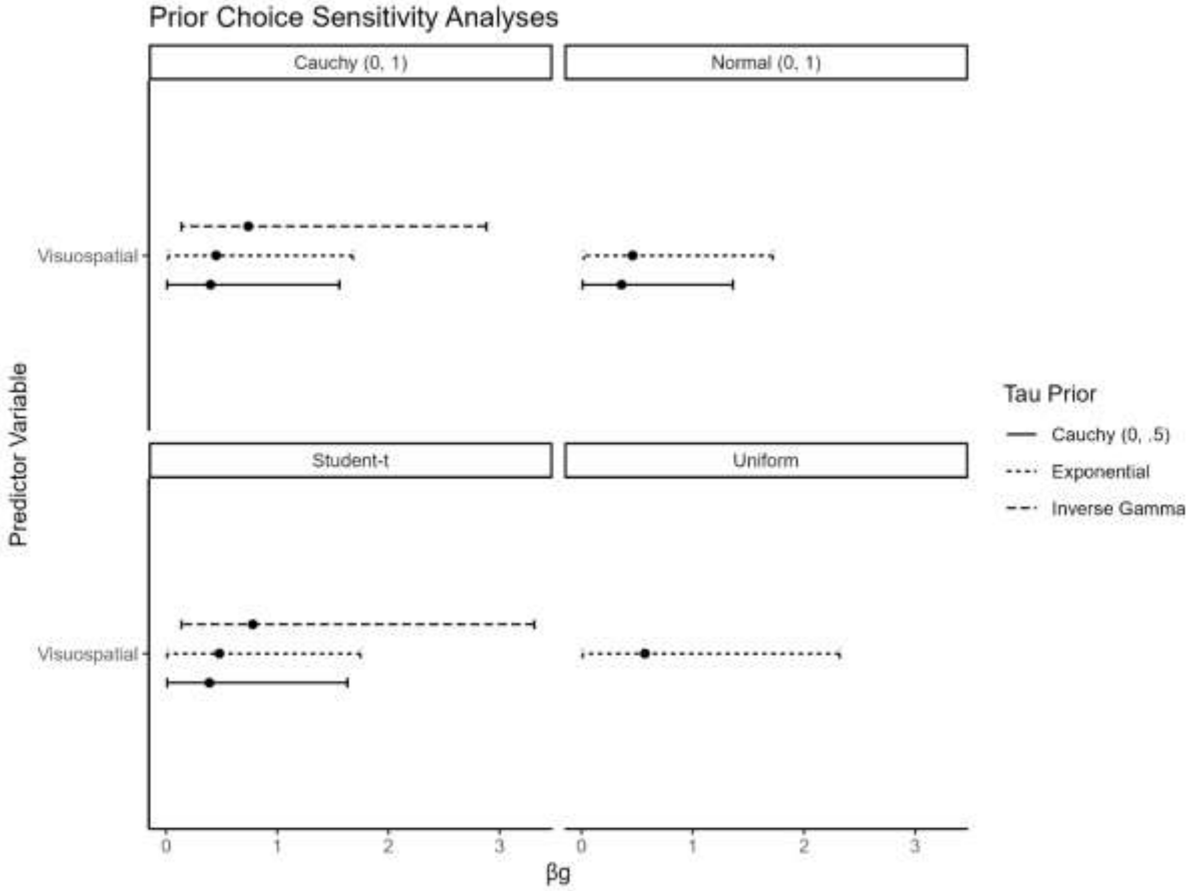

Figure 1. Study level standard deviation estimates with 95% confidence intervals for the Addenbrooke's Cognitive Examination: Visuospatial subtest model.

Judgment of Line Orientation

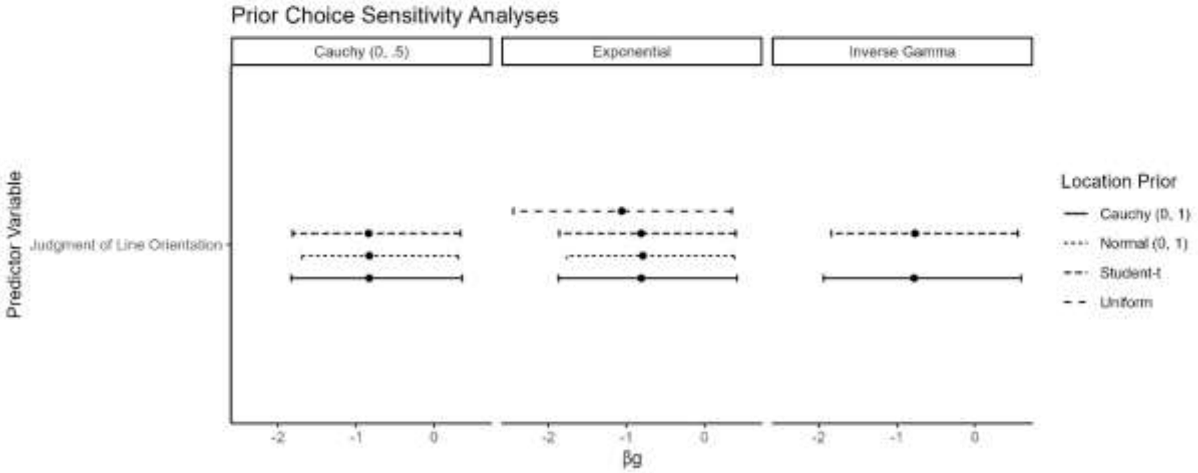

Figure 1. Regression coefficients with 95% confidence intervals for the Judgment of Line Orientation Test model.

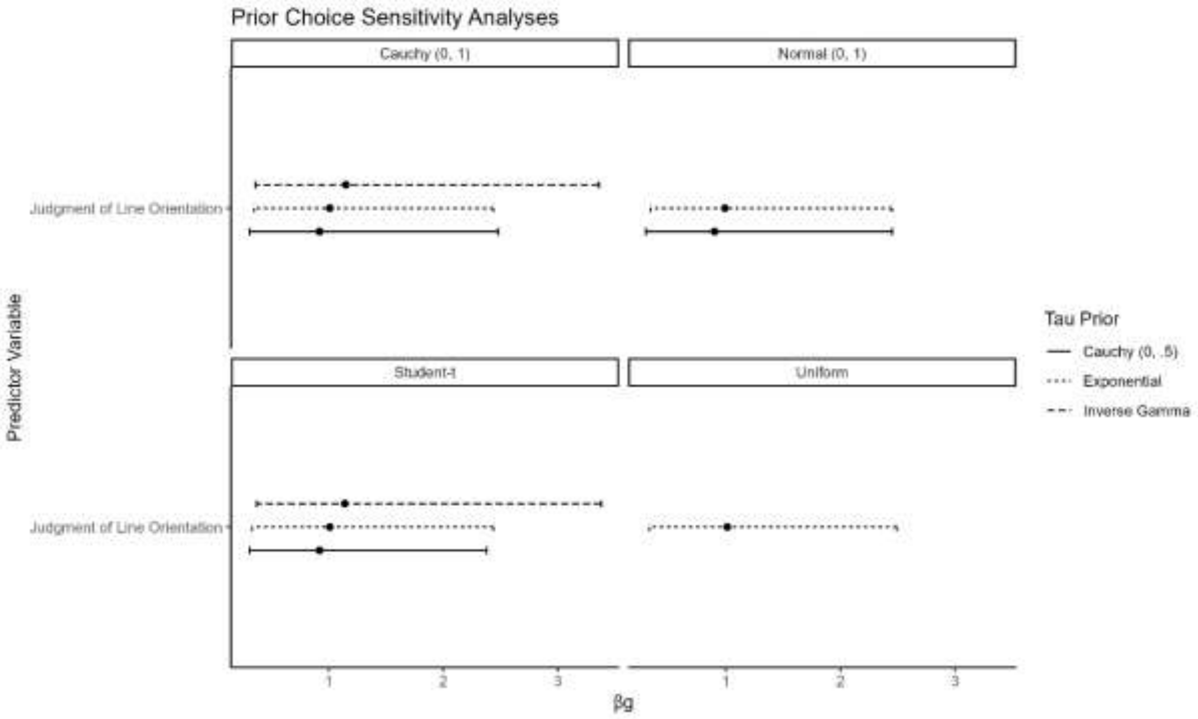

Figure 1. Study level standard deviation estimates with 95% confidence intervals for the Judgment of Line Orientation Test model.

Hooper's Test

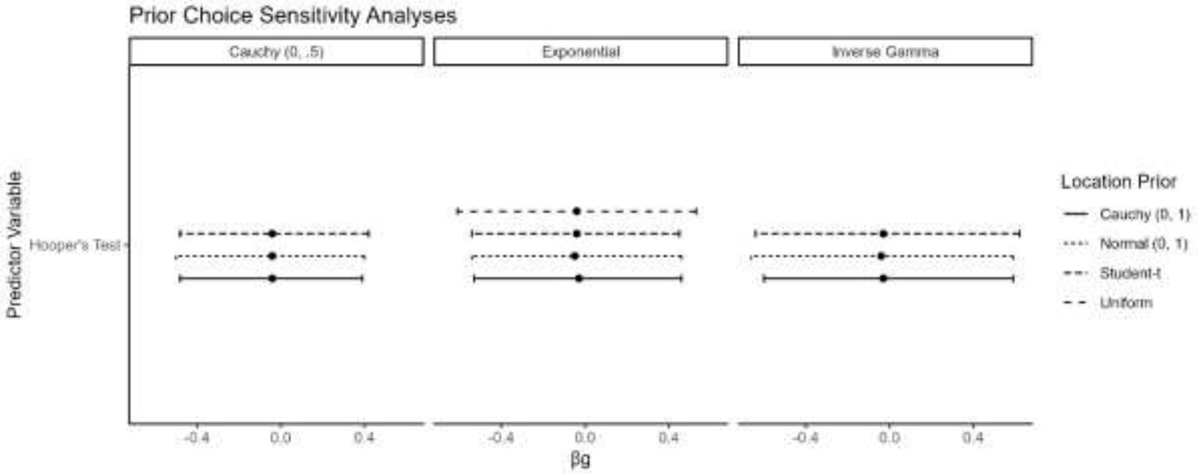

Figure 1. Regression coefficients with 95% confidence intervals for the Hooper's Visual Organisation Test model.

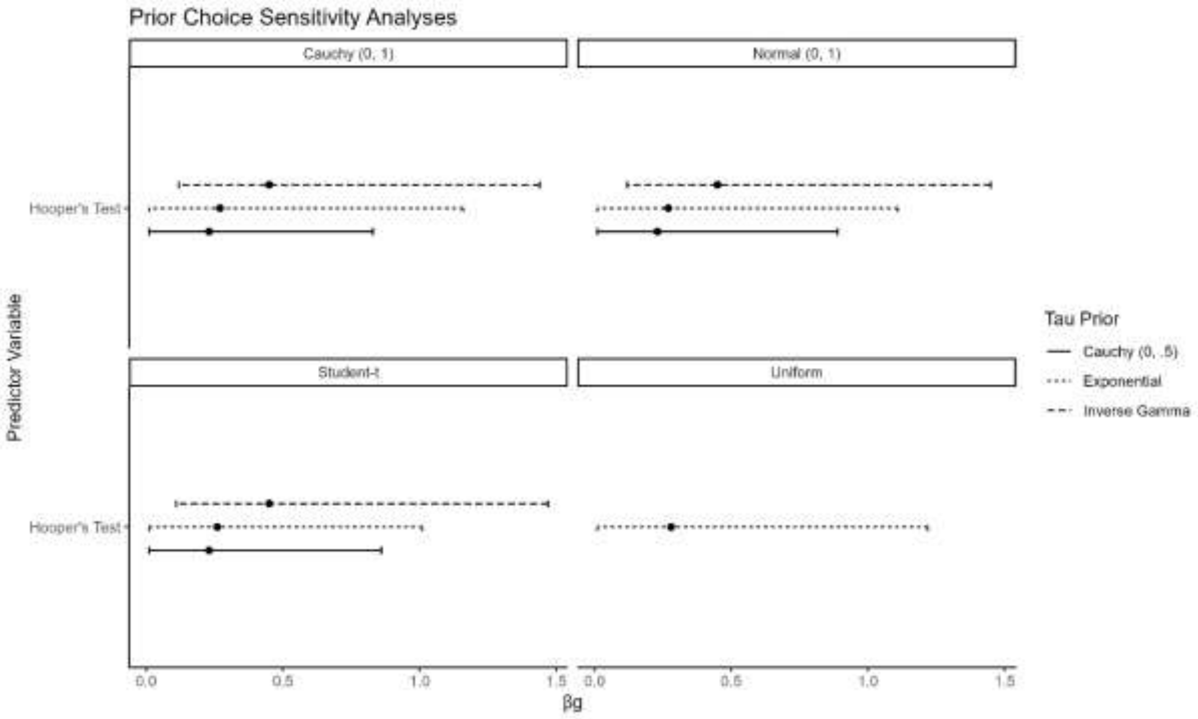

Figure 1. Study level standard deviation estimates with 95% confidence intervals for the Hooper's Visual Organisation Test model.

Line Bisection

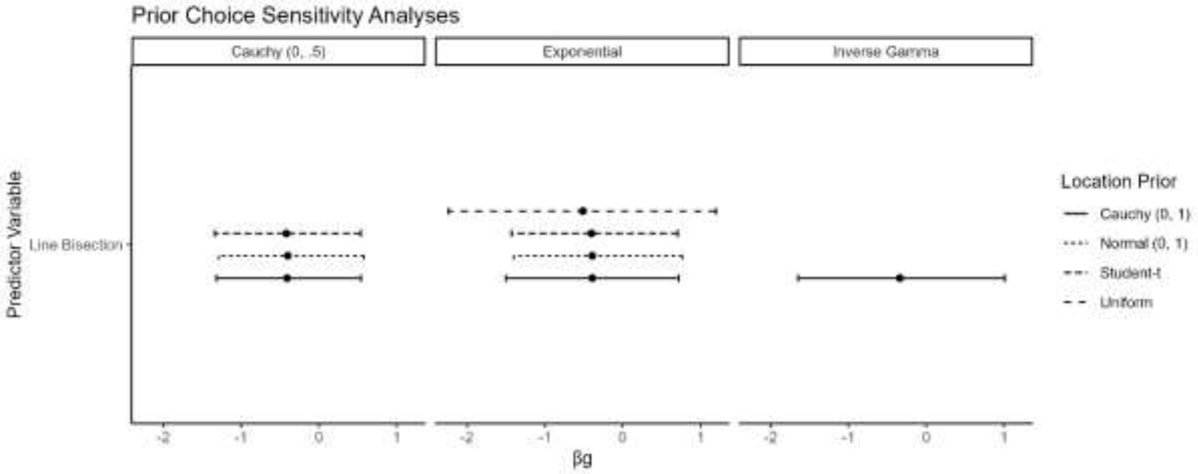

Figure 1. Regression coefficients with 95% confidence intervals for the Line Bisection model. For tests and studies included in the analysis see Supplementary Materials 1.

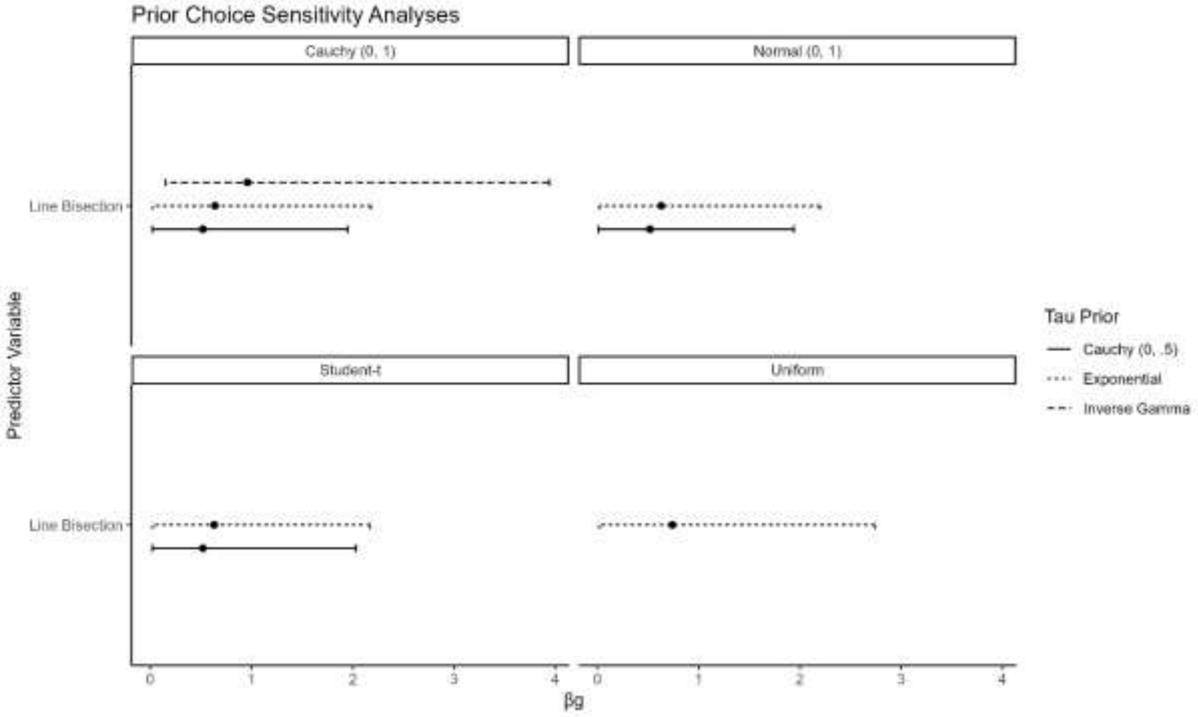

Figure 1. Study level standard deviation estimates with 95% confidence intervals for the Line Bisection model. For tests and studies included in the analysis see Supplementary Materials 1.

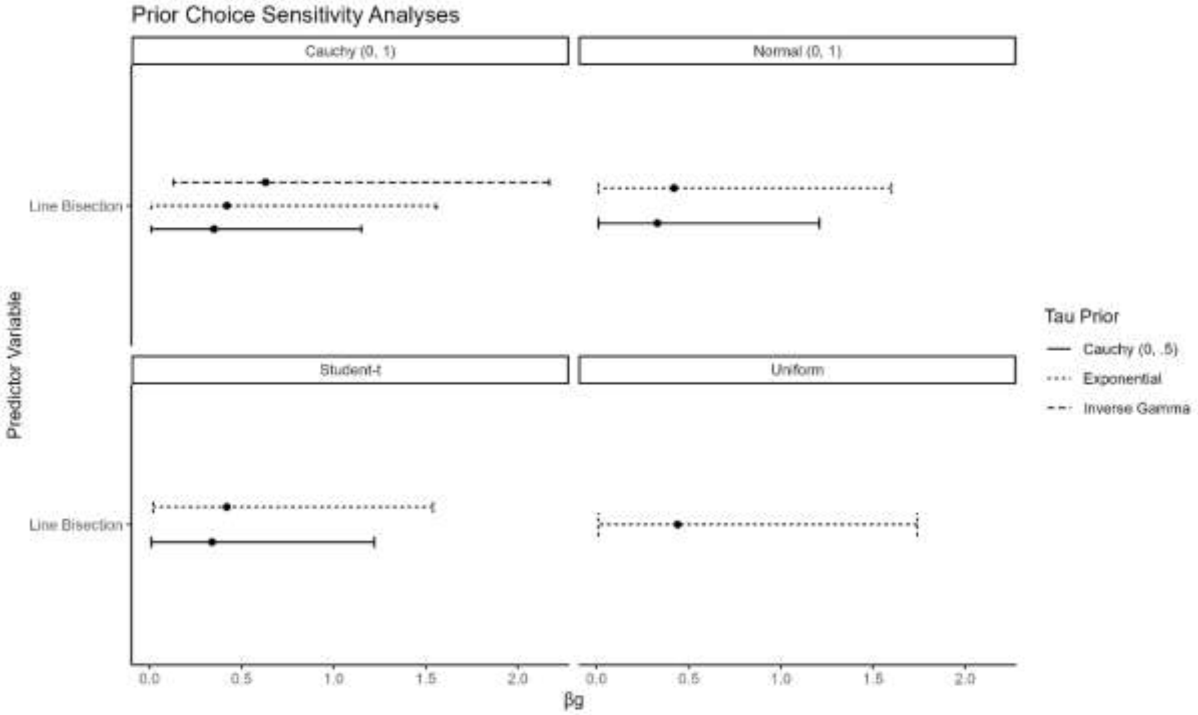

Figure 1. Effect size level standard deviation estimates with 95% confidence intervals for the Line Bisection model. For tests and studies included in the analysis see Supplementary Materials 1.

Other Figure Copy Measures

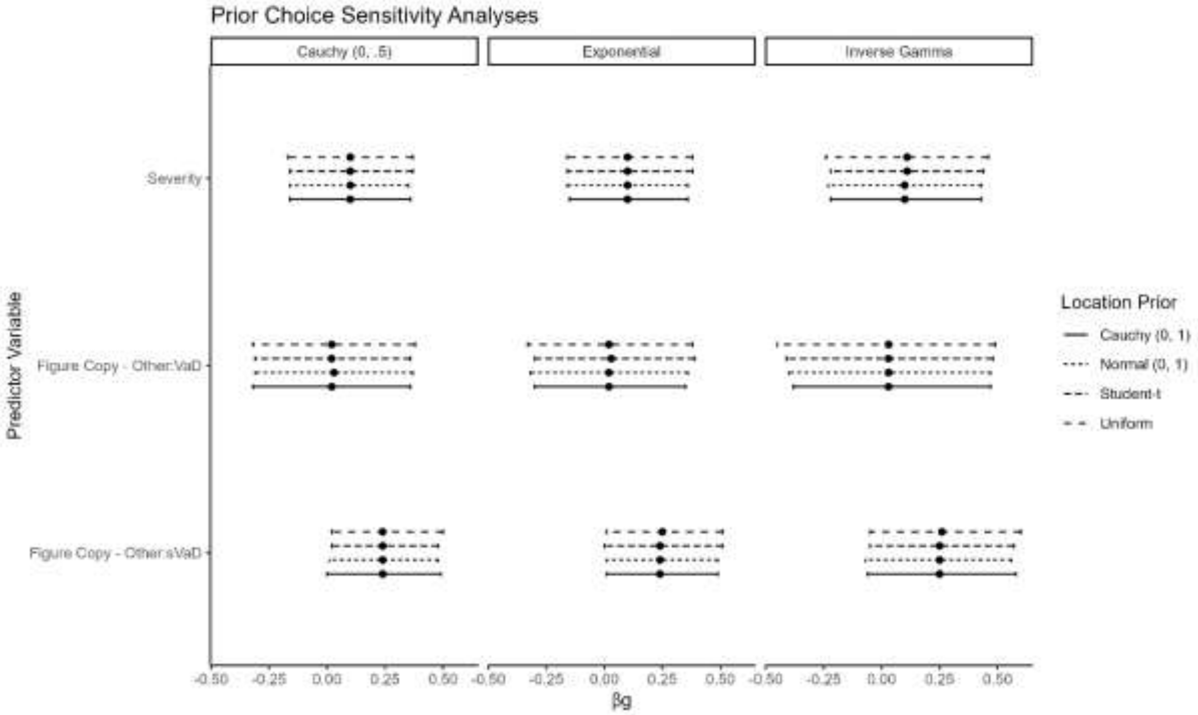

Figure 1. Regression coefficients with 95% confidence intervals for the Other Figure Copy Measures model. For tests included in the “Other Figure Copy Measures” see Supplementary Information 1.

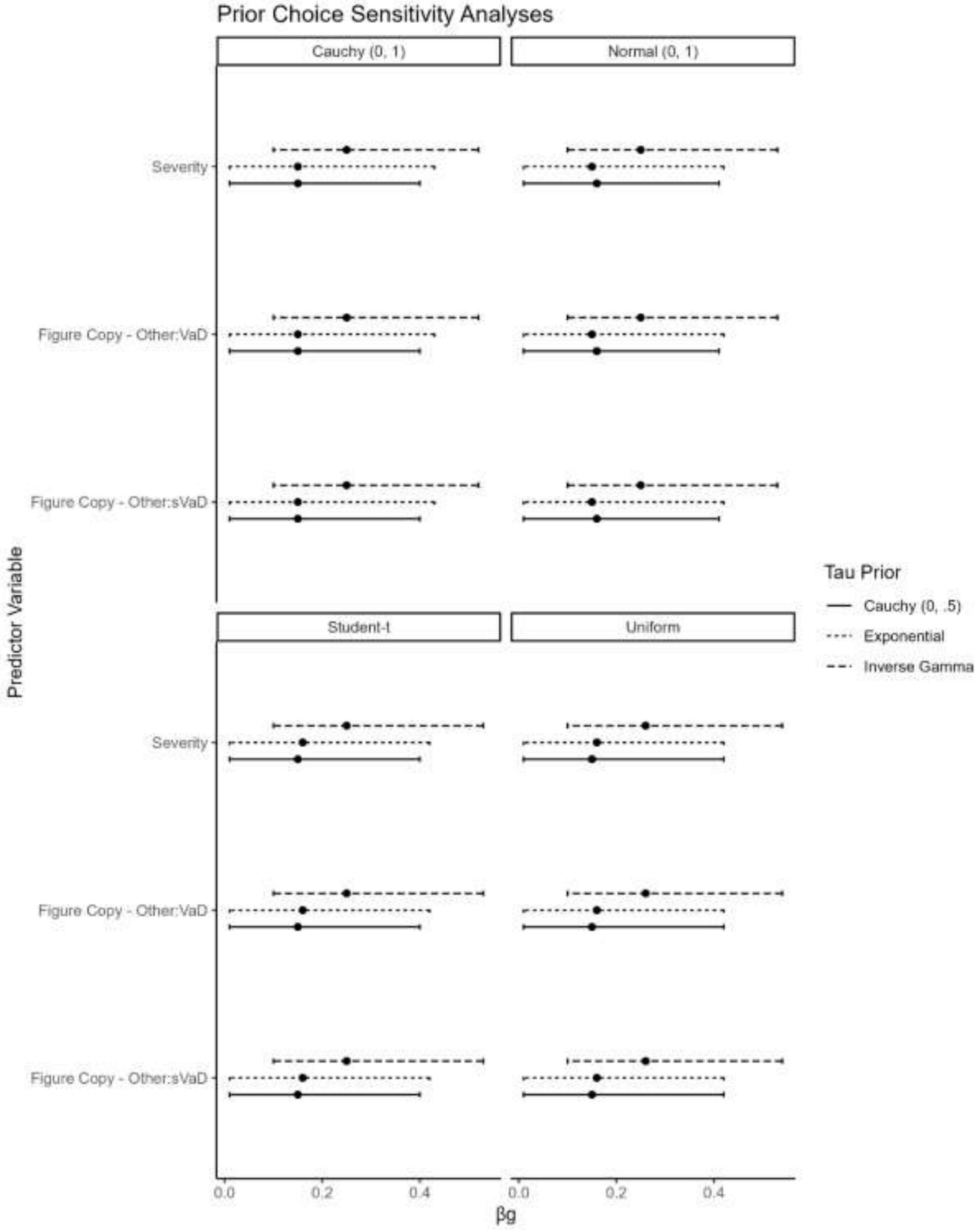

Figure 1. Study level standard deviation estimates with 95% confidence intervals for the Other Figure Copy Measures model. For tests included in the "Other Figure Copy Measures" see Supplementary Information 1.

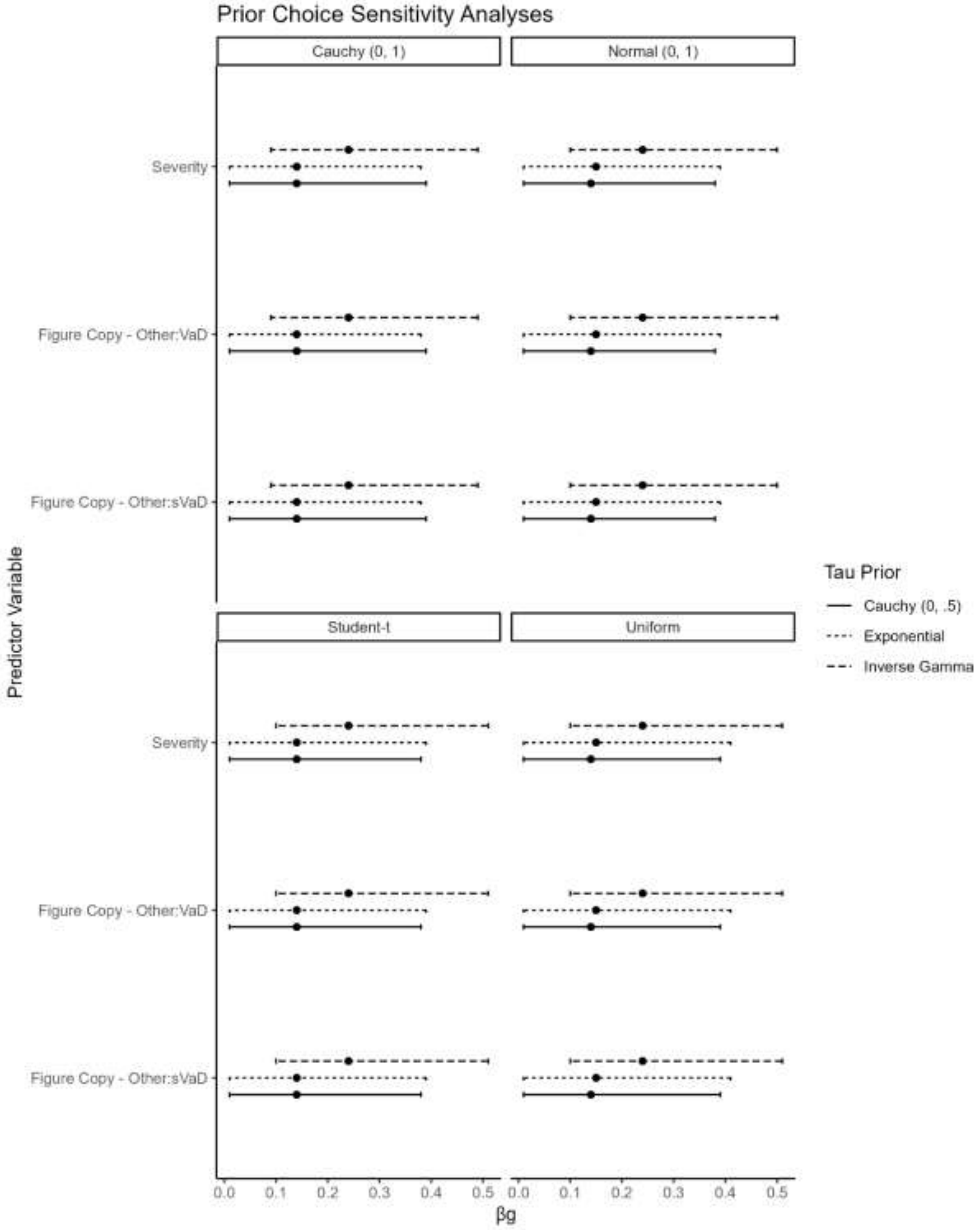

Figure 1. Effect size level standard deviation estimates with 95% confidence intervals for the Other Figure Copy Measures model. For tests included in the "Other Figure Copy Measures" see Supplementary Information 1.

Other Measures of Constructional Praxis

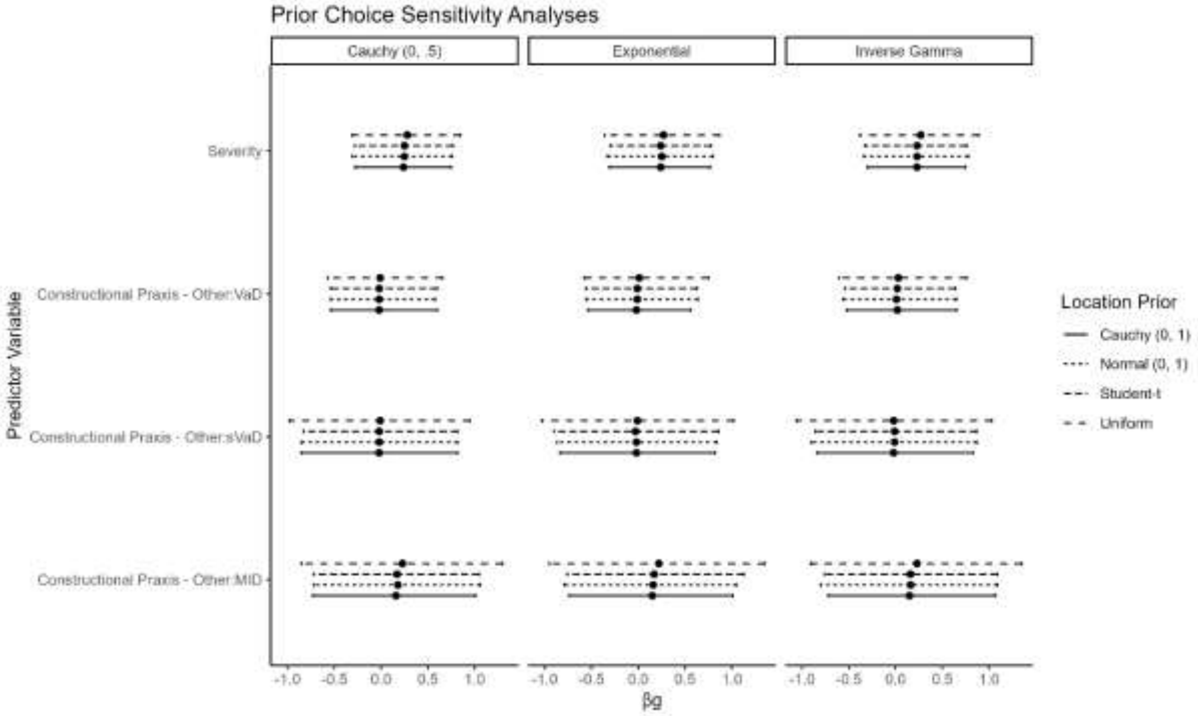

Figure 1. Regression coefficients with 95% confidence intervals for the Other Measures of Constructional Praxis model. For tests included in the "Other Measures of Constructional Praxis" see Supplementary Information 1. sVaD: subcortical vascular dementia, VaD: vascular dementia, MID: multi-infarct dementia, Severity: difference in dementia severity between dementia groups.

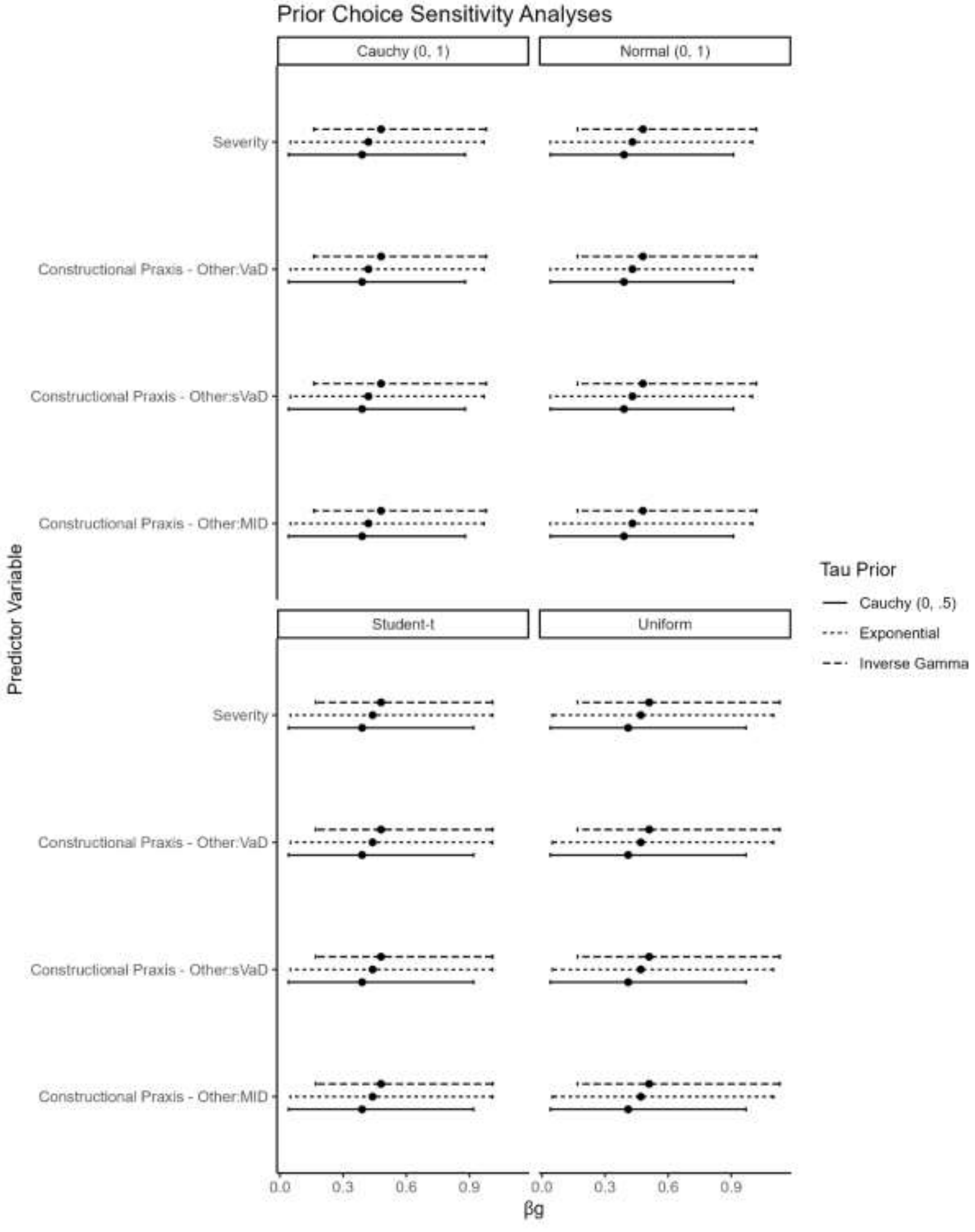

Figure 1. Study level standard deviation estimates with 95% confidence intervals for the Other Measures of Constructional Praxis model. For tests included in the "Other Measures of Constructional Praxis" see Supplementary Information 1. sVaD: subcortical vascular dementia, VaD: vascular dementia, MID: multi-infarct dementia, Severity: difference in dementia severity between dementia groups.

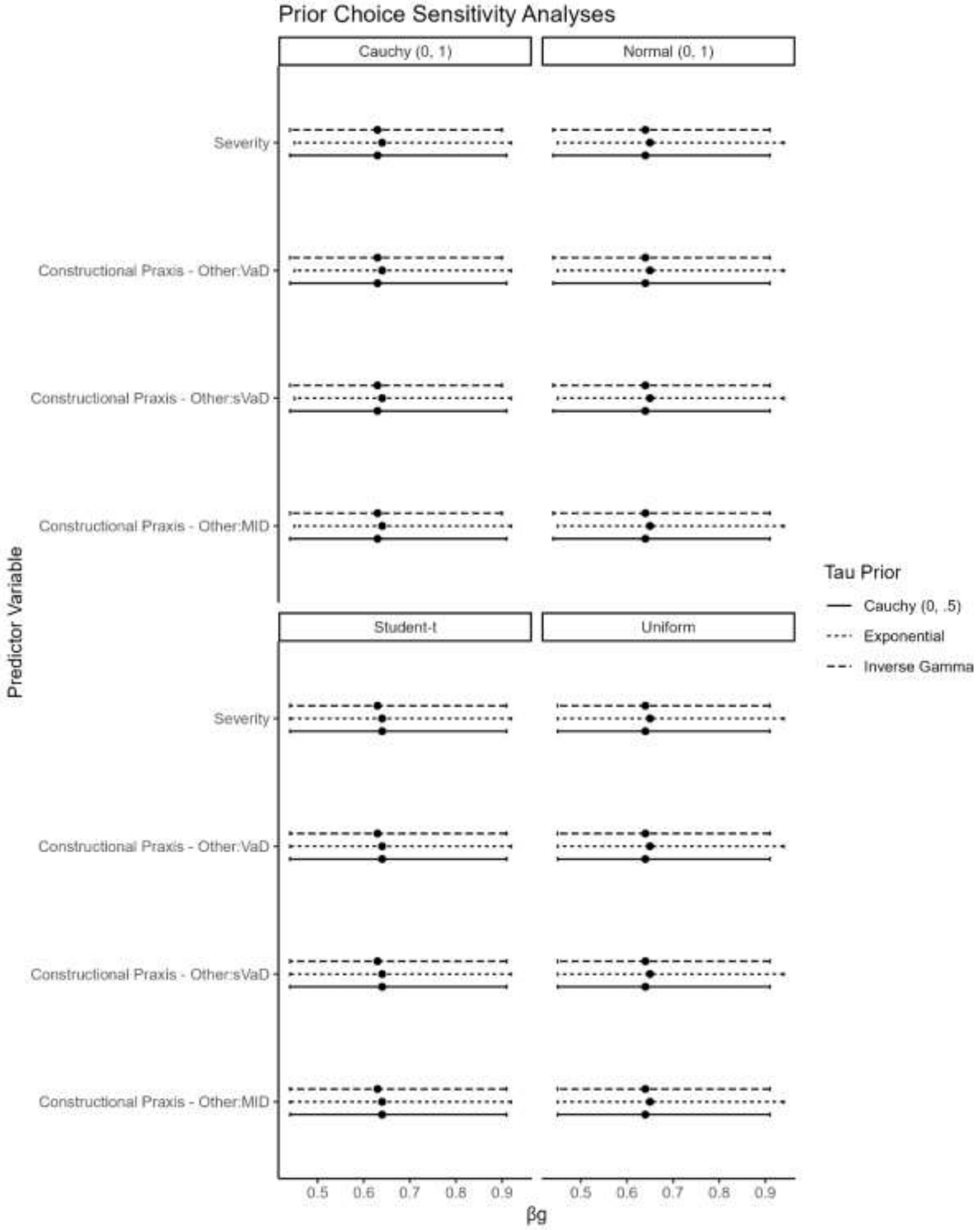

Figure 1. Effect size level standard deviation estimates with 95% confidence intervals for the Other Measures of Constructional Praxis model. For tests included in the "Other Measures of Constructional Praxis" see Supplementary Information 1. sVaD: subcortical vascular dementia, VaD: vascular dementia, MID: multi-infarct dementia, Severity: difference in dementia severity between dementia groups.

Other Measures of Visuo-Spatial Processing

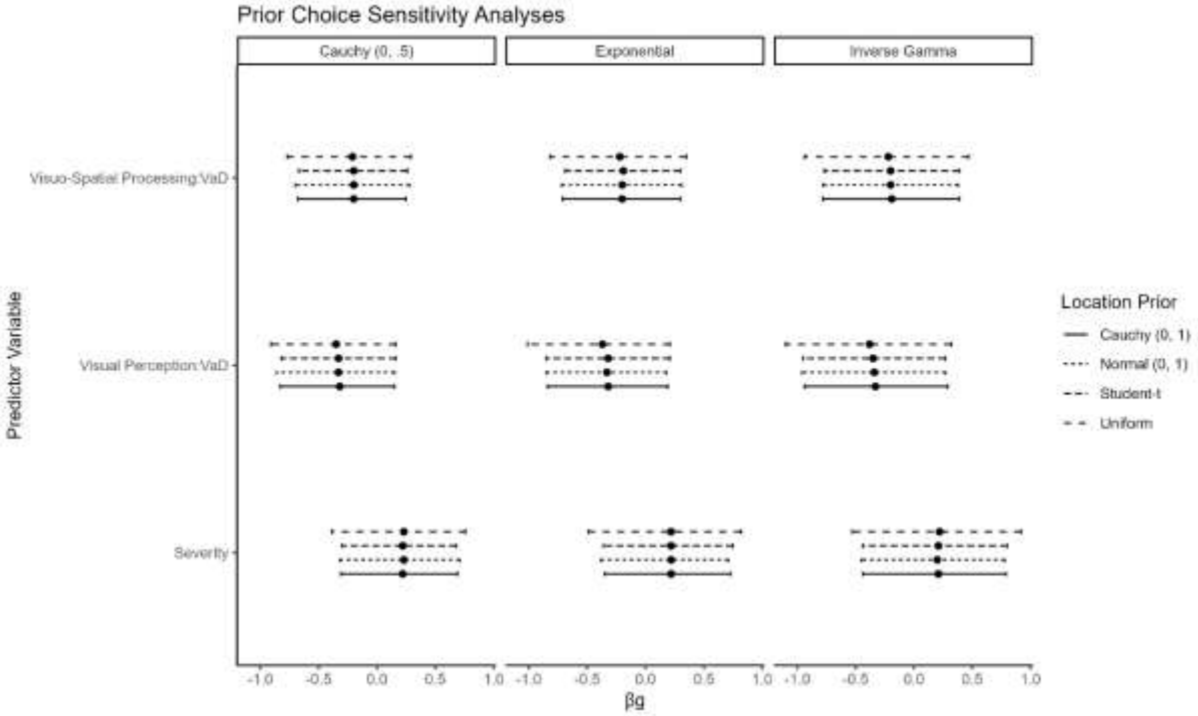

Figure 1. Regression coefficients with 95% confidence intervals for the Other Measures of Visuo-Spatial Processing model. For tests included in the “Other Measures of Visuo-Spatial Processing” see Supplementary Information 1. VaD: vascular dementia, Severity: difference in dementia severity between dementia groups.

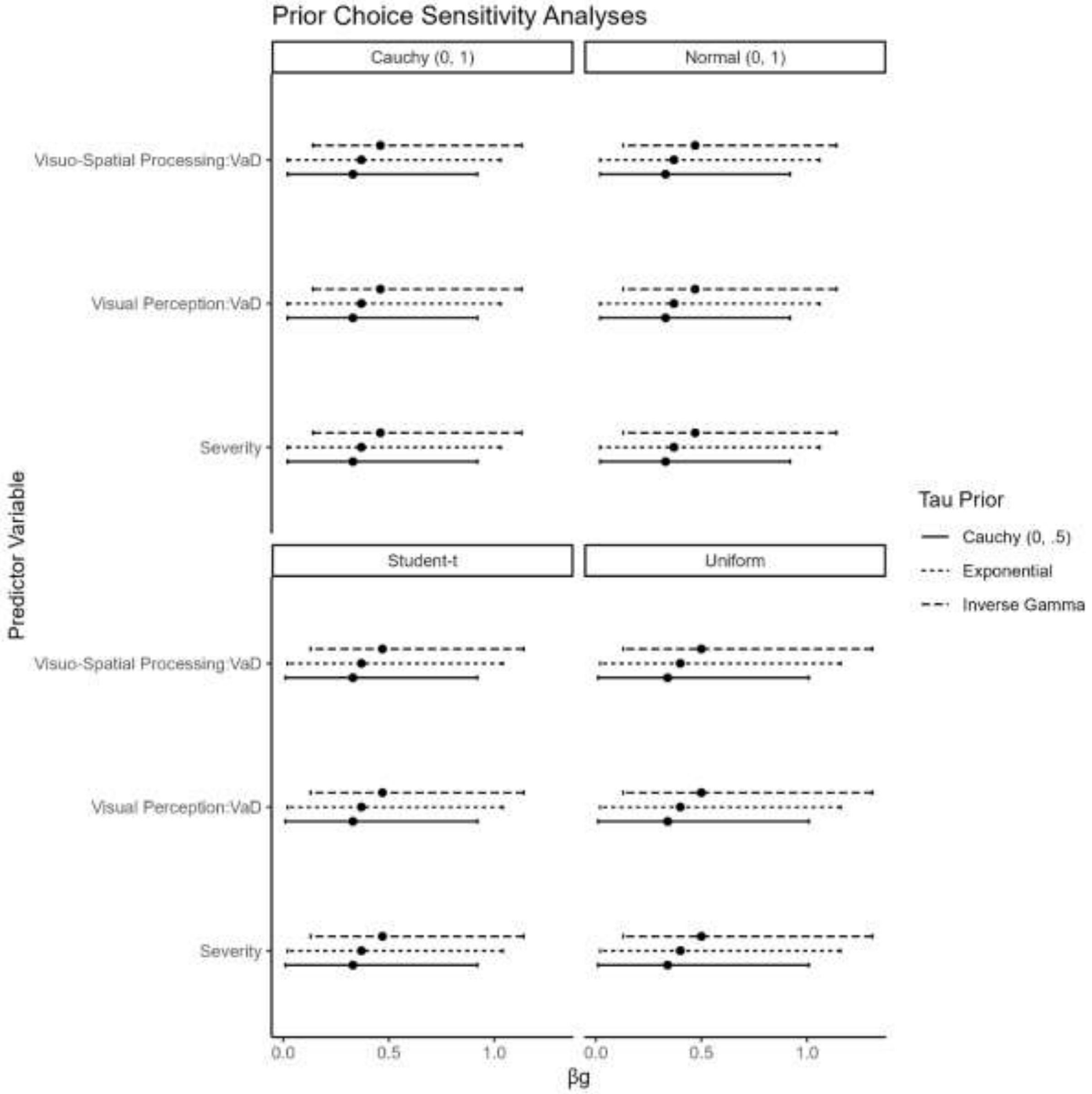

Figure 1. Study level standard deviation estimates with 95% confidence intervals for the Other Measures of Visuo-Spatial Processing model. For tests included in the "Other Measures of Visuo-Spatial Processing" see Supplementary Information 1. VaD: vascular dementia, Severity: difference in dementia severity between dementia groups.

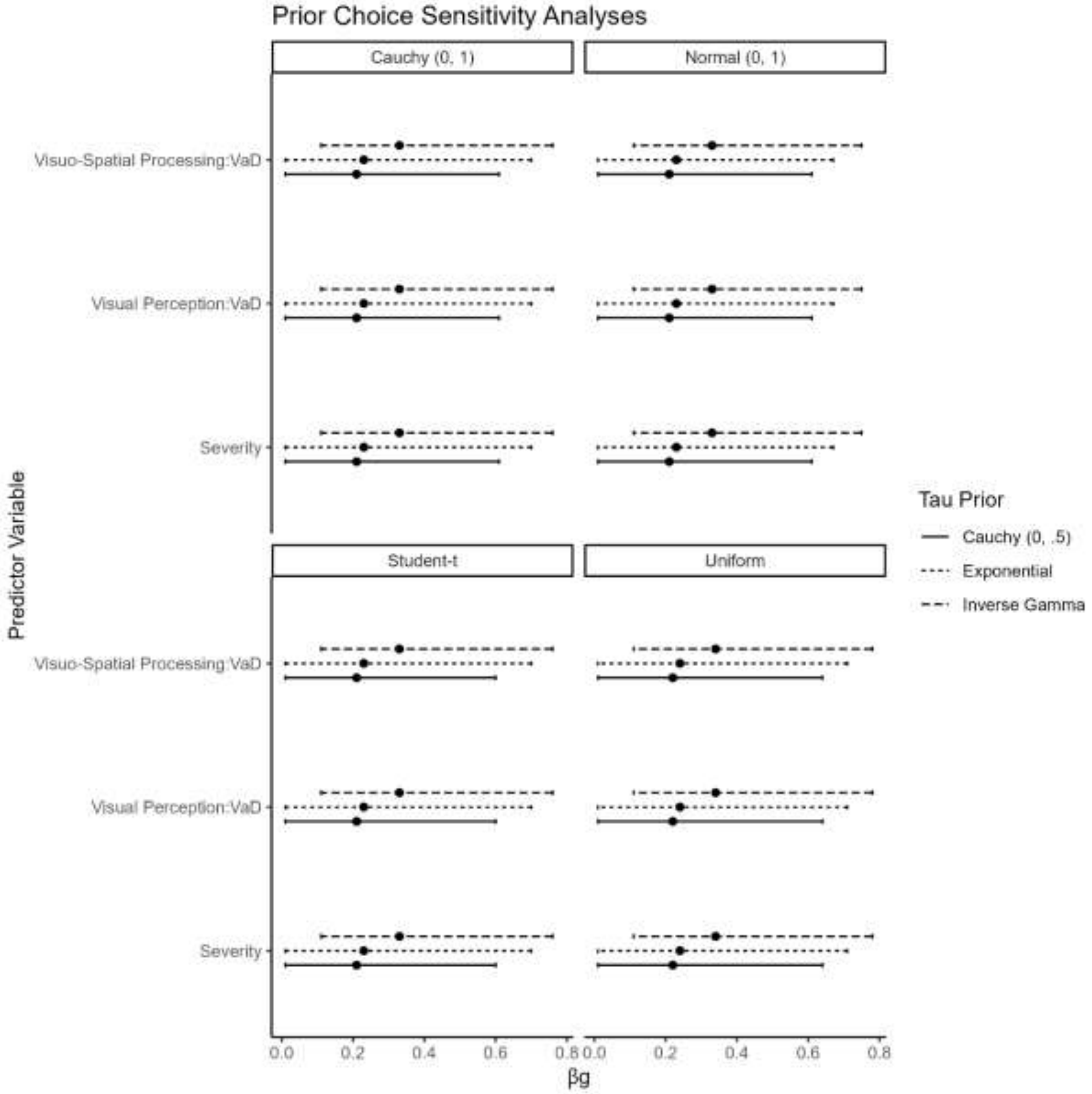

Figure 1. Effect size level standard deviation estimates with 95% confidence intervals for the Other Measures of Visuo-Spatial Processing model. For tests included in the "Other Measures of Visuo-Spatial Processing" see Supplementary Information 1. VaD: vascular dementia, Severity: difference in dementia severity between dementia groups.

Visuo-Spatial Processing: Quality Sensitivity Analysis

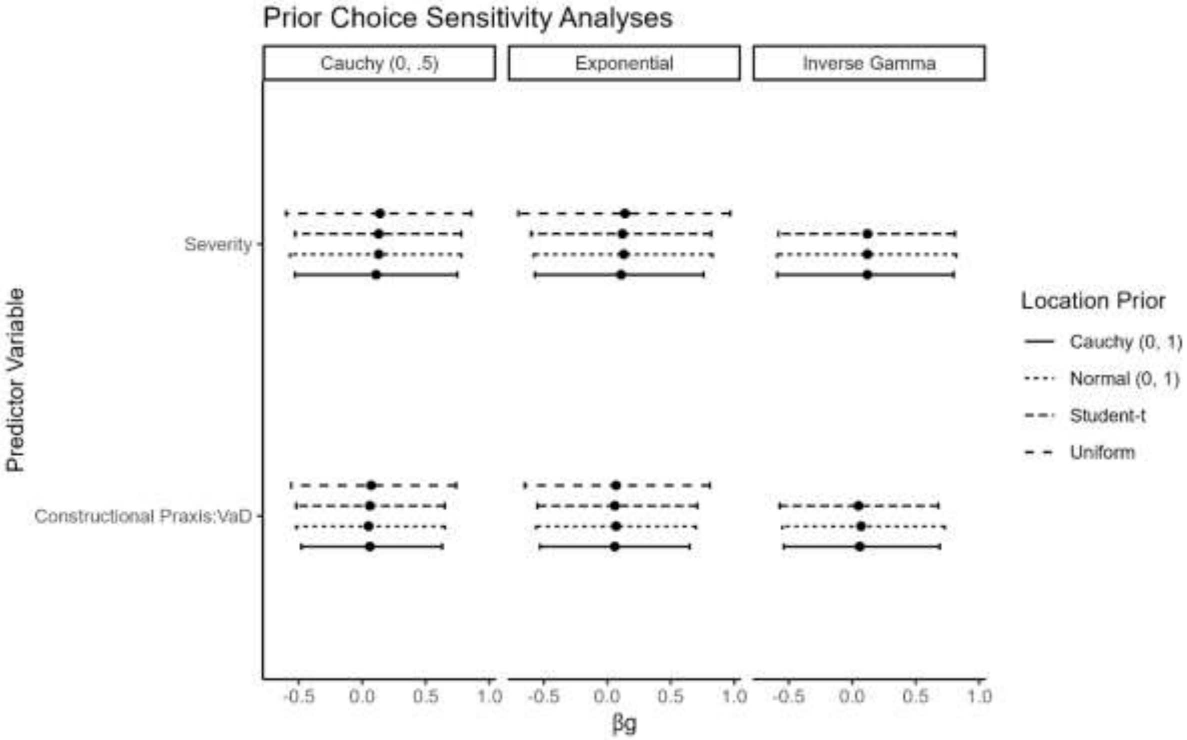

Figure 1. Regression coefficients with 95% confidence intervals for the study quality sensitivity analysis of Constructional Praxis. For tests included see Supplementary Information 1. VaD: vascular dementia, Severity: difference in dementia severity between dementia groups.

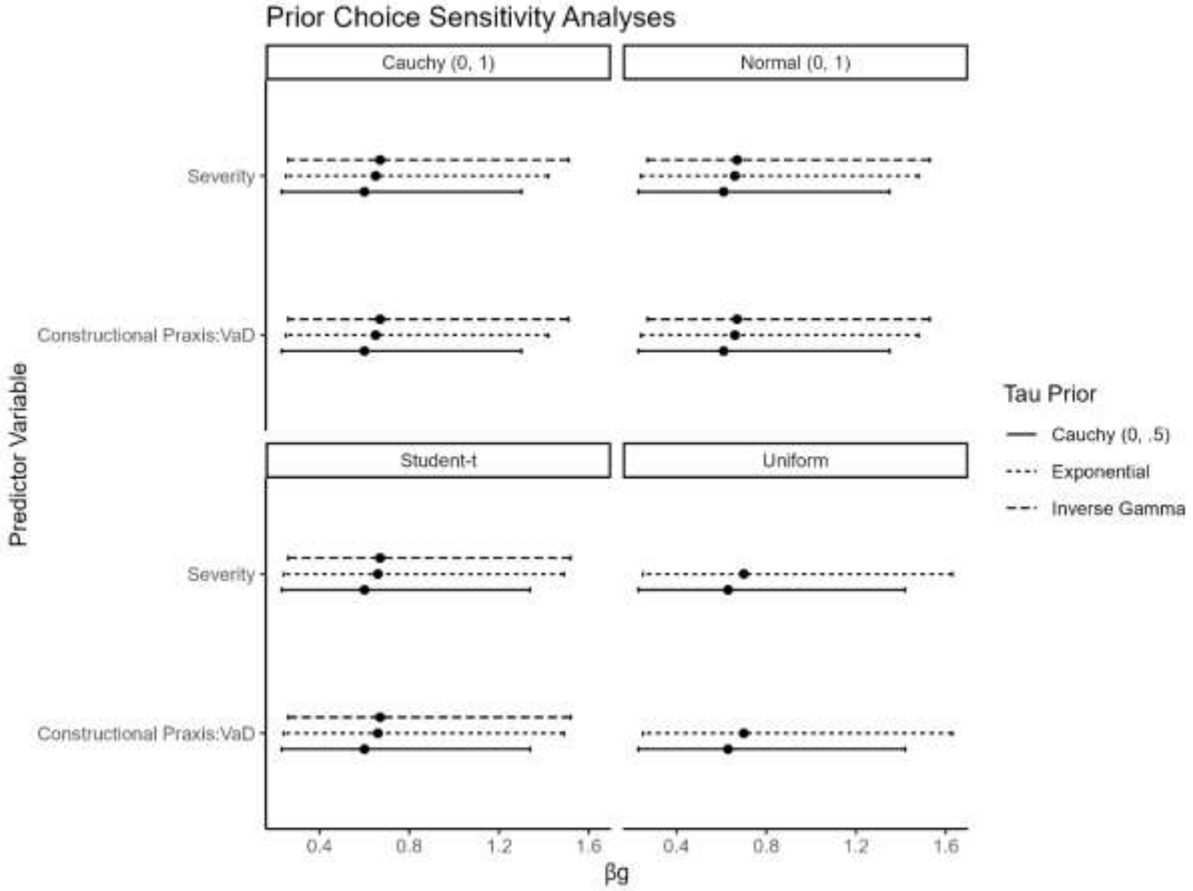

Figure 1. Study level standard deviation estimates with 95% confidence intervals for the study quality sensitivity analysis of Constructional Praxis. For tests included see Supplementary Information 1. VaD: vascular dementia, Severity: difference in dementia severity between dementia groups.

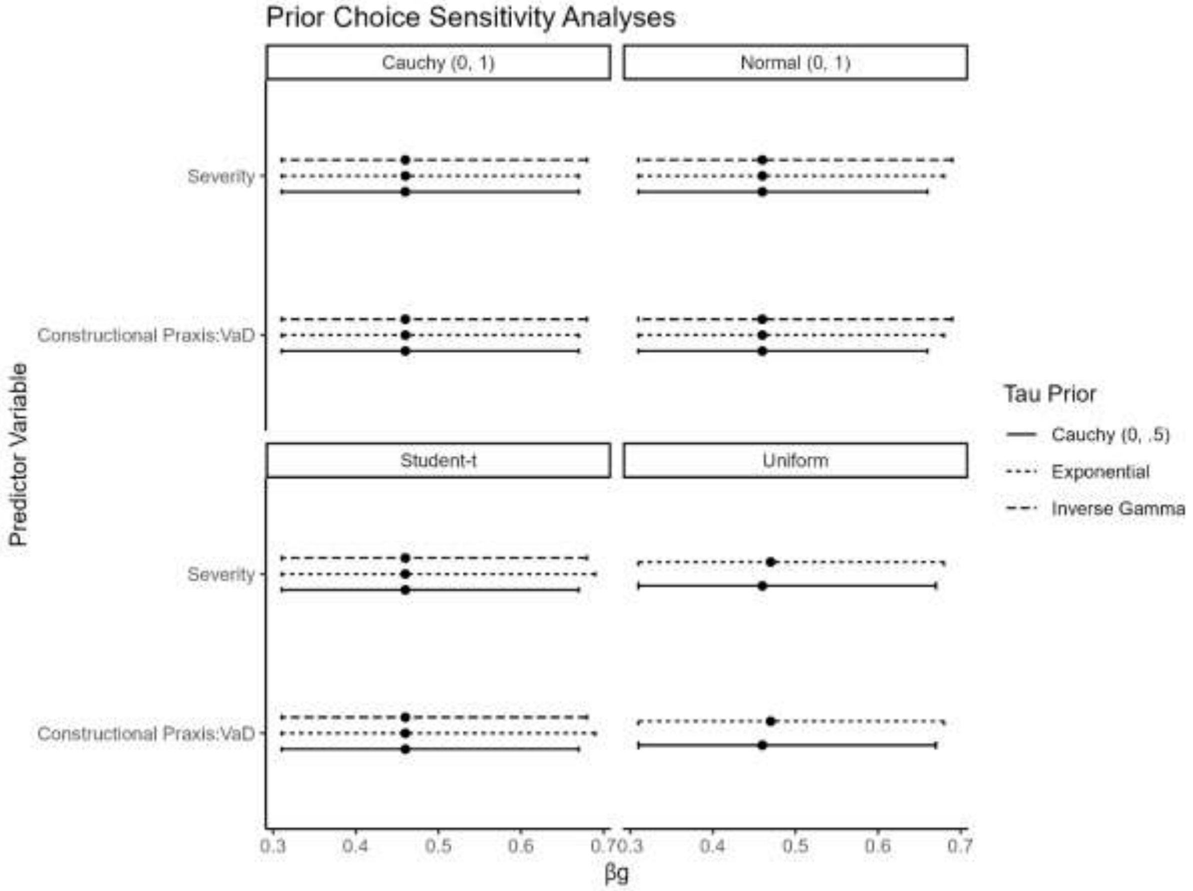

Figure 1. Effect size level standard deviation estimates with 95% confidence intervals for the study quality sensitivity analysis of Constructional Praxis. For tests included see Supplementary Information 1. VaD: vascular dementia, Severity: difference in dementia severity between dementia groups.

Intelligence Measures

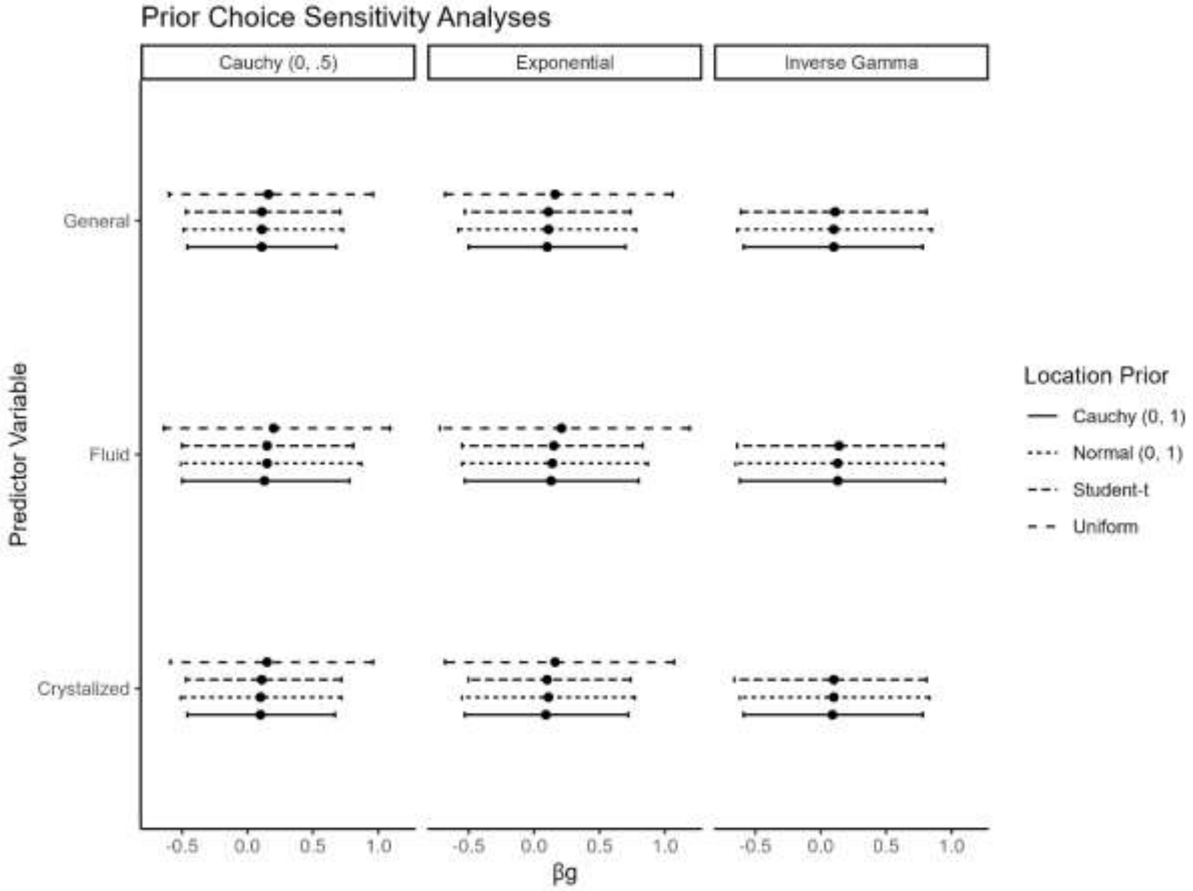

Figure 1. Regression coefficients with 95% confidence intervals for the Measures of general (General), fluid (Fluid) and crystalized (Crystalized) intelligence. For tests included in each domain, see Supplementary Information 1.

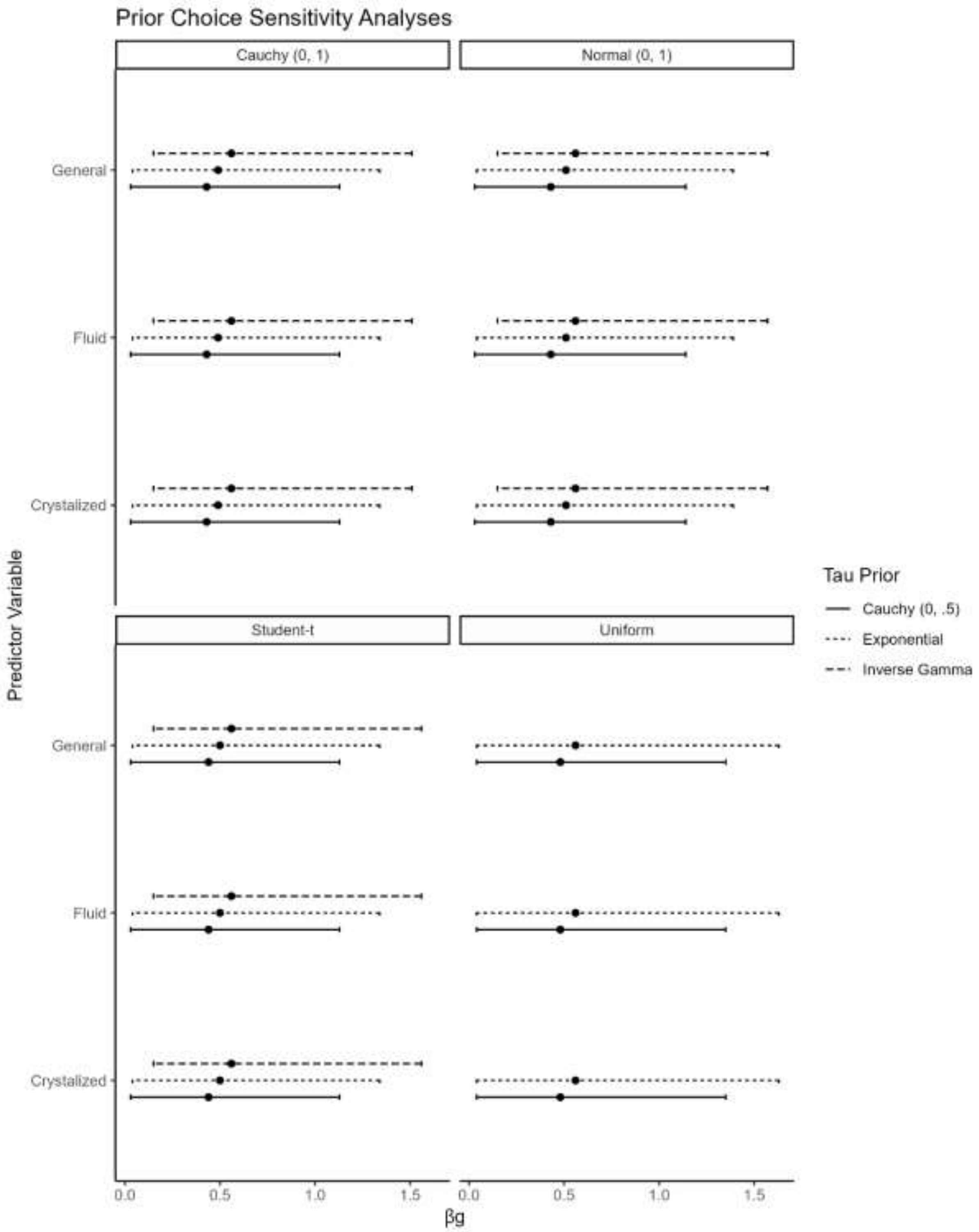

Figure 1. Study level standard deviation estimates with 95% confidence intervals for the Measures of general (General), fluid (Fluid) and crystalized (Crystalized) intelligence. For tests included in each domain, see Supplementary Information 1.

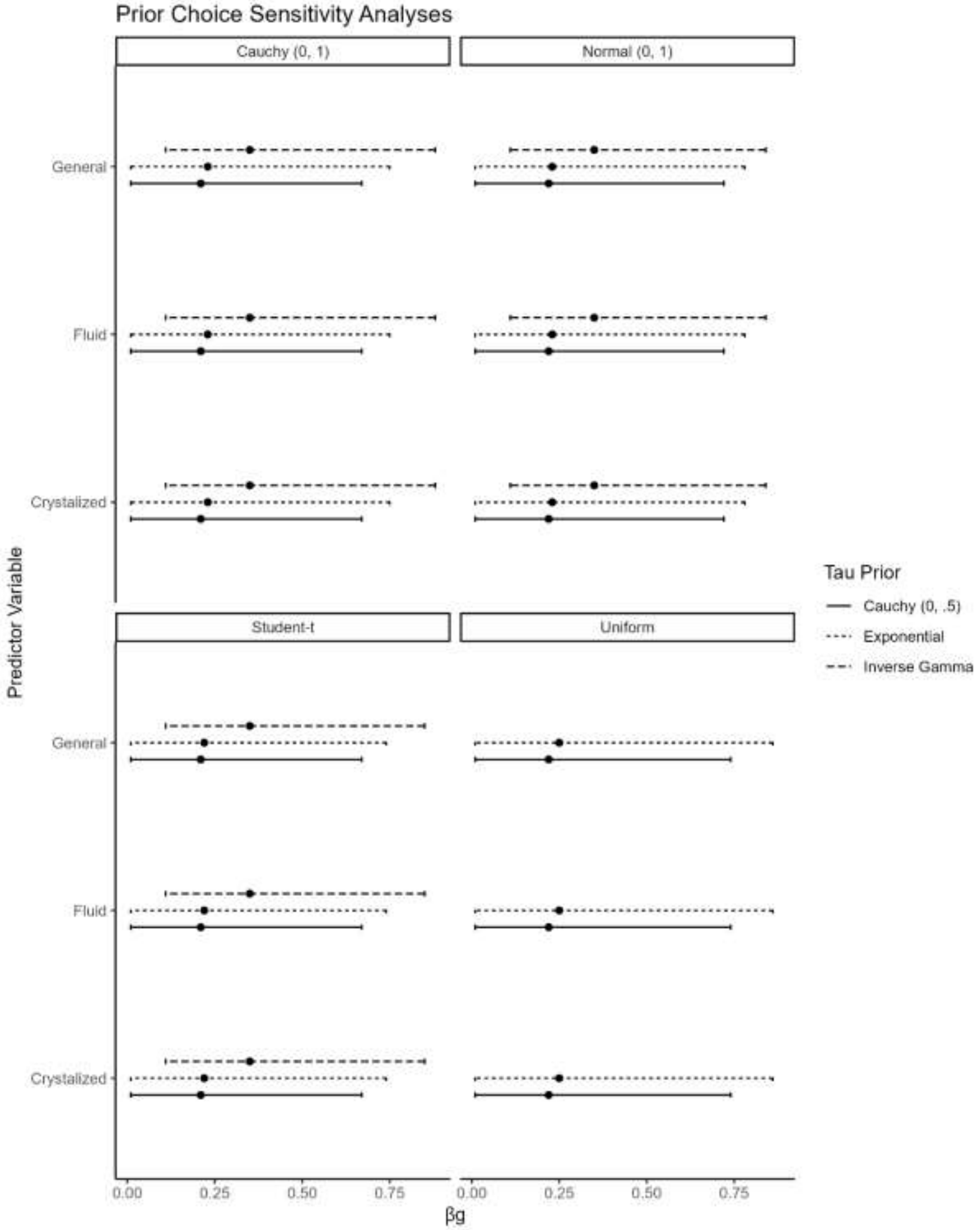

Figure 1. Effect size level standard deviation estimates with 95% confidence intervals for the Measures of general (General), fluid (Fluid) and crystalized (Crystalized) intelligence. For tests included in each domain, see Supplementary Information 1.

Attention

Trail Making Test – A

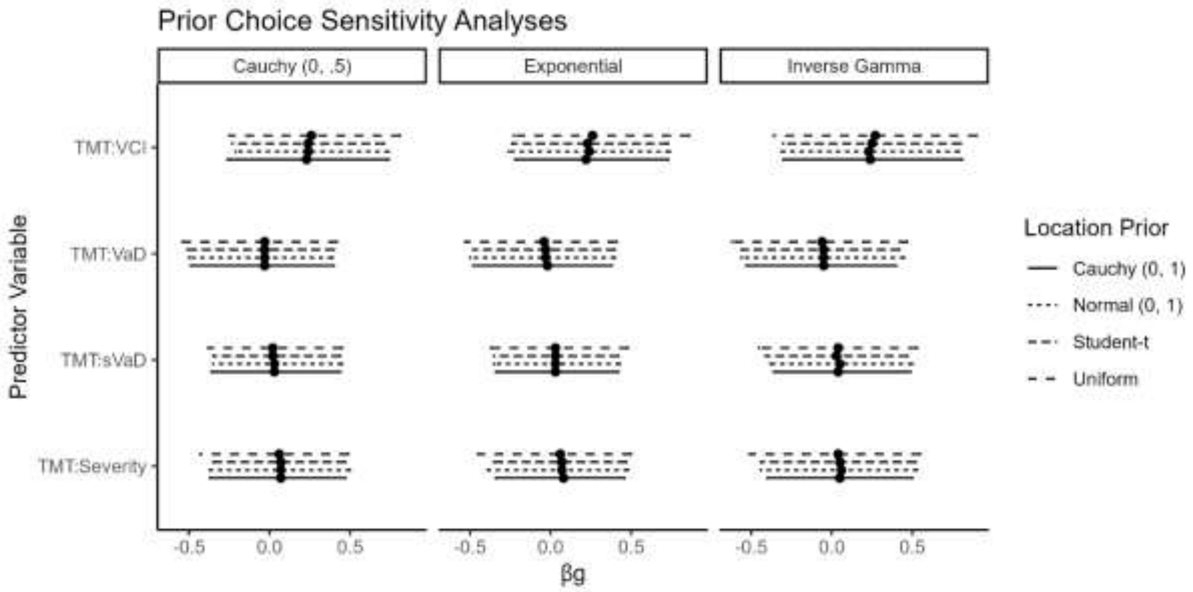

Figure 1. Regression coefficients with 95% confidence intervals for the Trail Making Test: A model. sVaD: subcortical vascular dementia, VaD: vascular dementia, VCI: vascular cognitive impairment, Severity: difference in dementia severity between dementia groups.

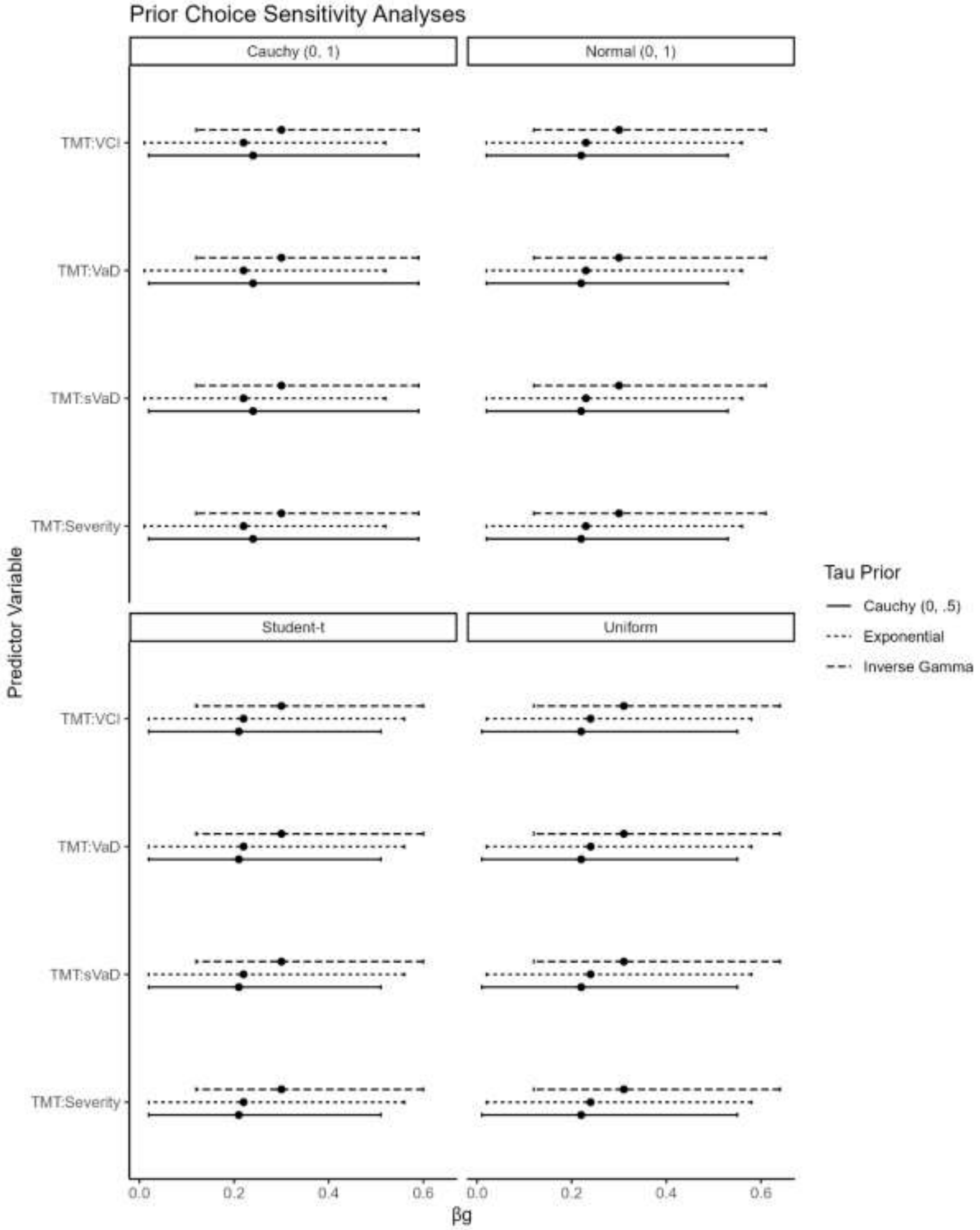

Figure 1. Study level standard deviation estimates with 95% confidence intervals for the Trail Making Test: A model. sVaD: subcortical vascular dementia, VaD: vascular dementia, VCI: vascular cognitive impairment, Severity: difference in dementia severity between dementia groups.

Digit Span Forward

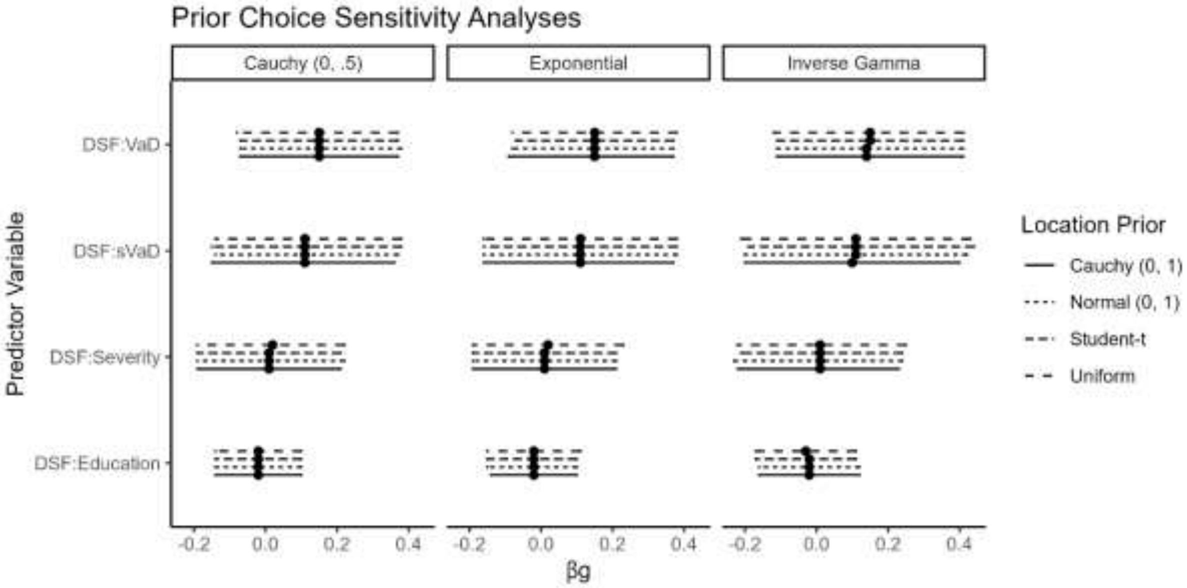

Figure 1. Regression coefficients with 95% confidence intervals for the Digit Span Forward model. For included tests see Supplementary Materials 1. sVaD: subcortical vascular dementia, VaD: vascular dementia, Severity: difference in dementia severity between dementia groups, Education: difference in average years of education between dementia groups.

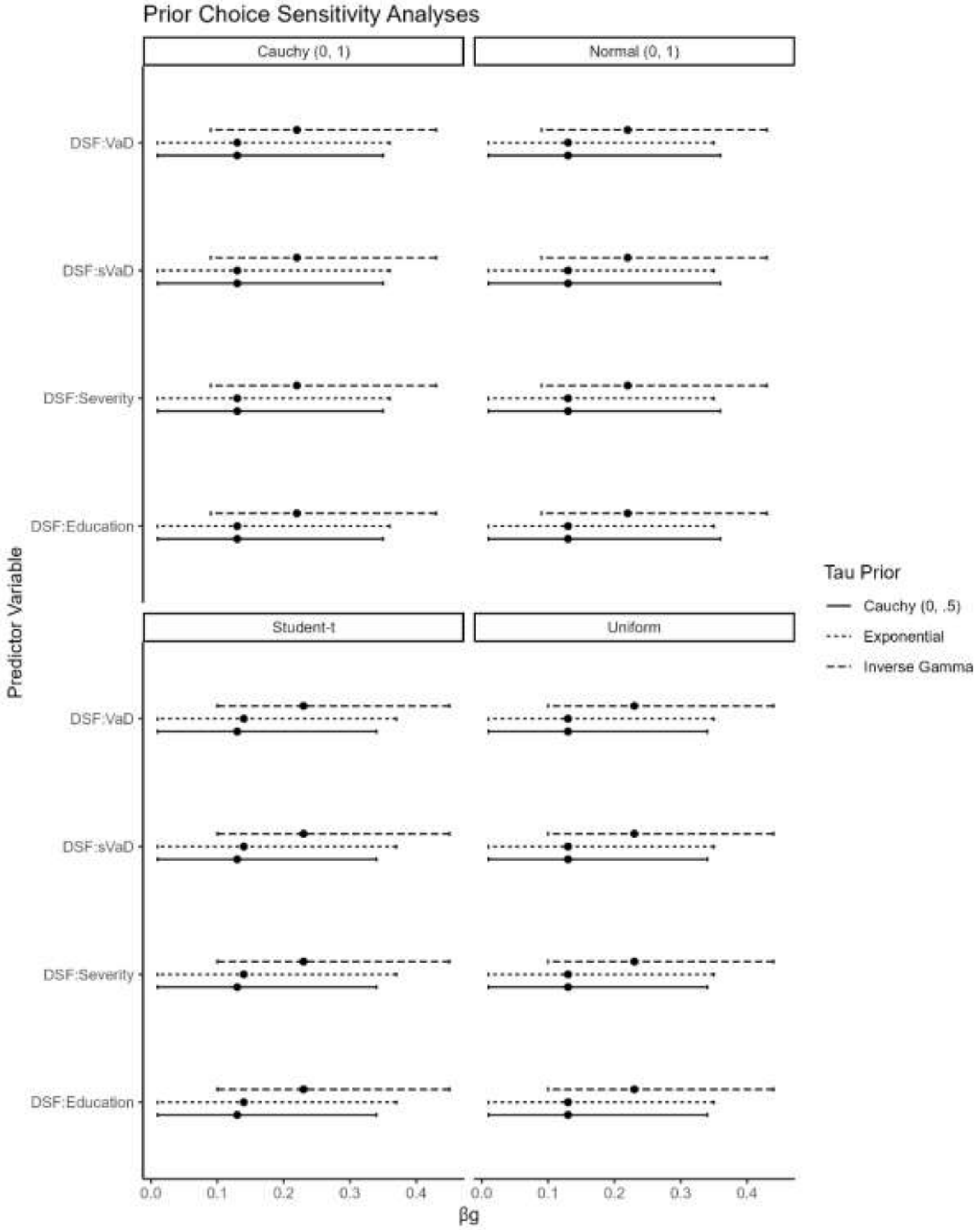

Figure 1. Study level standard deviation estimates with 95% confidence intervals for the Digit Span Forward model. For included tests see Supplementary Materials 1. sVaD: subcortical vascular dementia, VaD: vascular dementia, Severity: difference in dementia severity between dementia groups, Education: difference in average years of education between dementia groups.

Digit Symbol Substitution Test

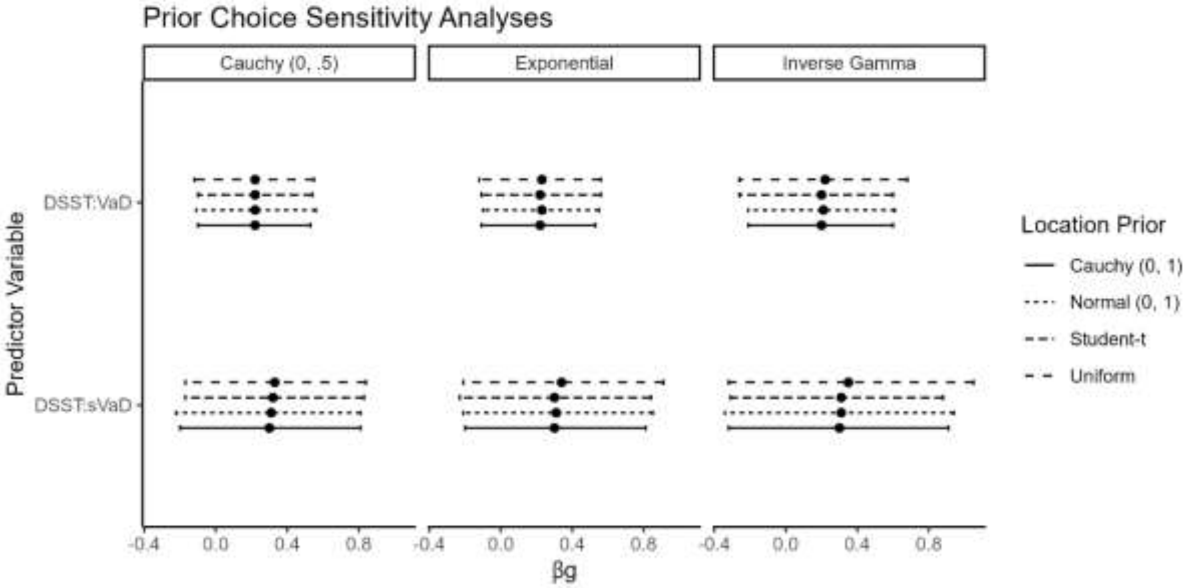

Figure 1. Regression coefficients with 95% confidence intervals for the Digit Symbol Substitutions Test model. sVaD: subcortical vascular dementia, VaD: vascular dementia.

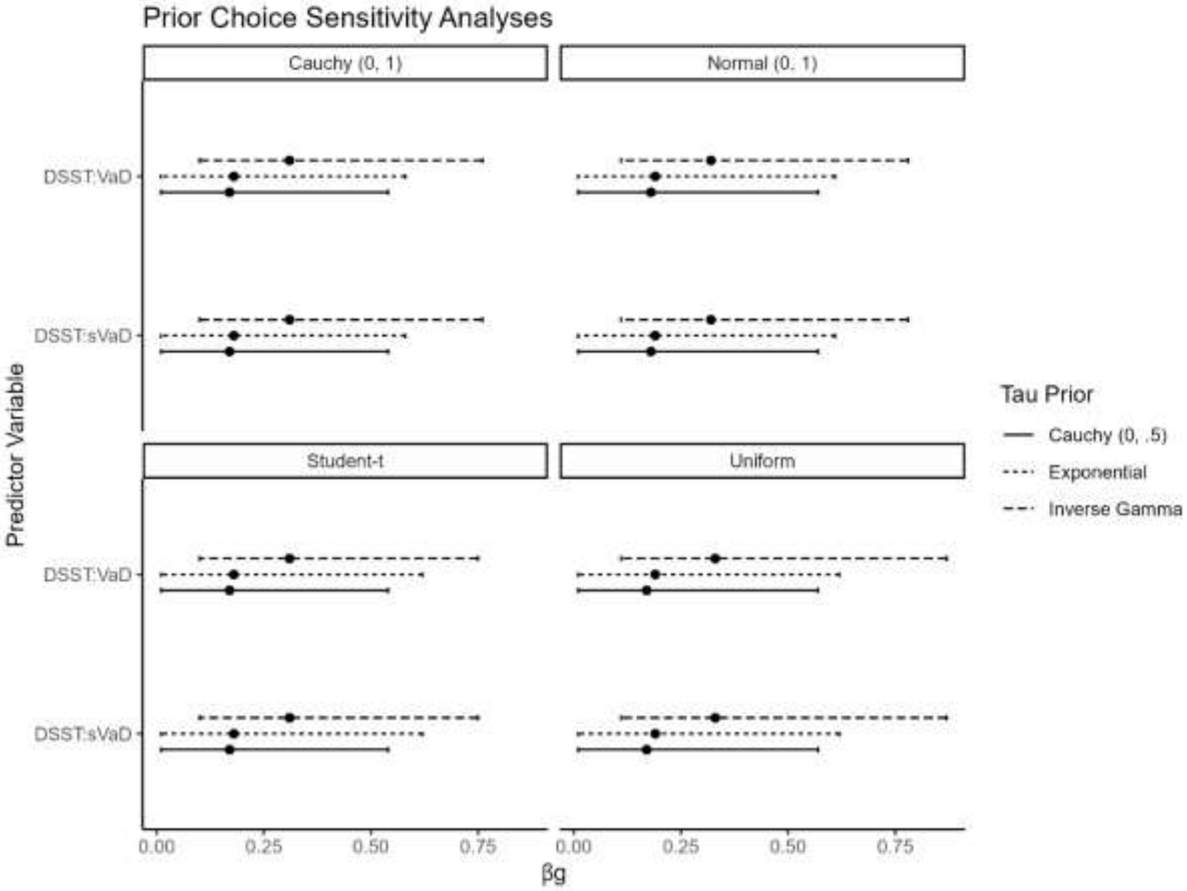

Figure 1. Study level standard deviation estimates with 95% confidence intervals for the Digit Symbol Substitutions Test model. sVaD: subcortical vascular dementia, VaD: vascular dementia.

Symbol Digit Modalities Test

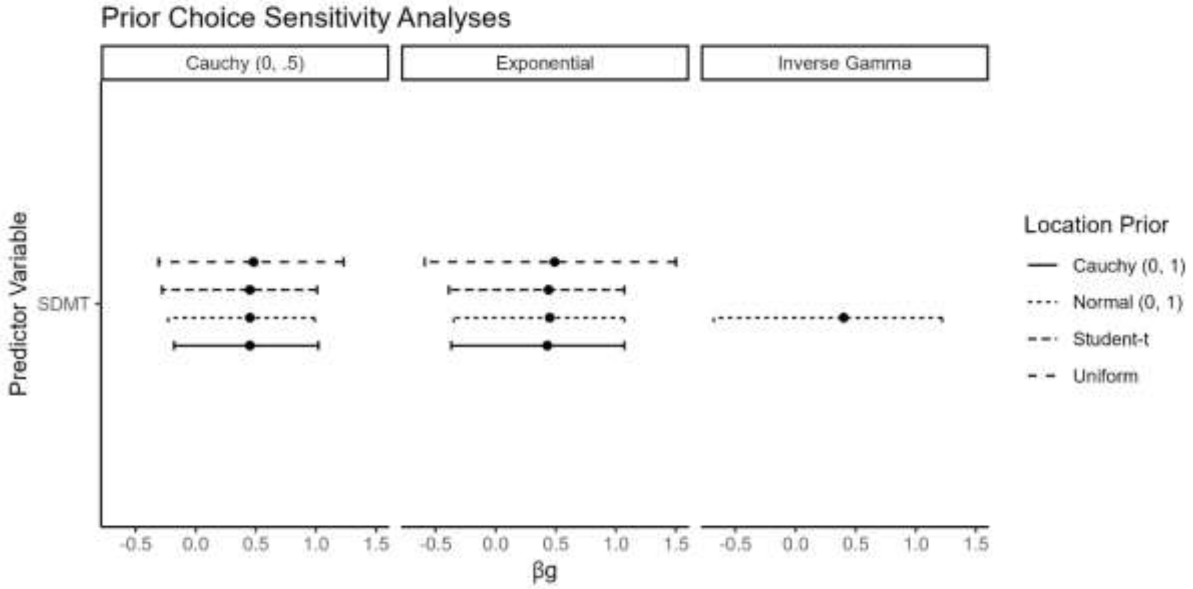

Figure 1. Regression coefficients with 95% confidence intervals for the Symbol Digit Modalities Test model.

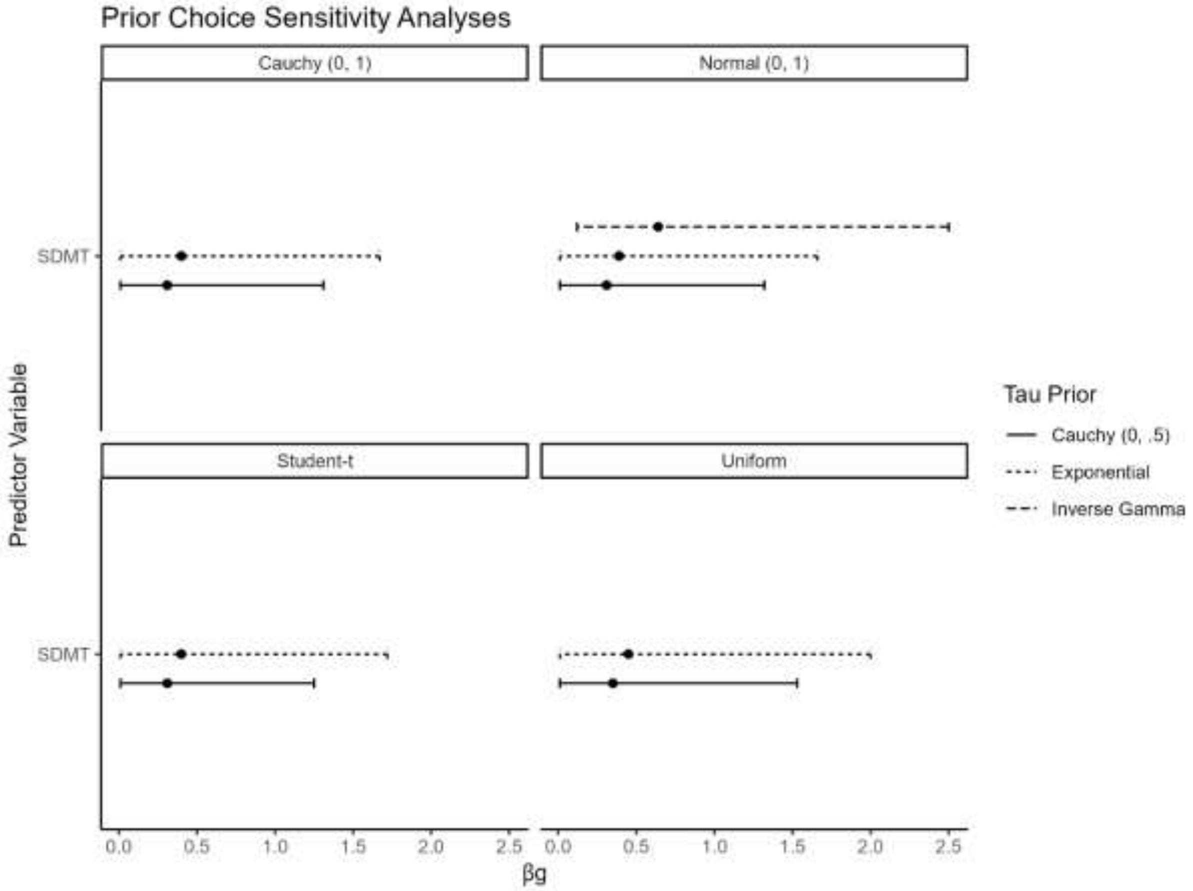

Figure 1. Study level standard deviation estimates with 95% confidence intervals for the Symbol Digit Modalities Test model.

Choice Reaction Time

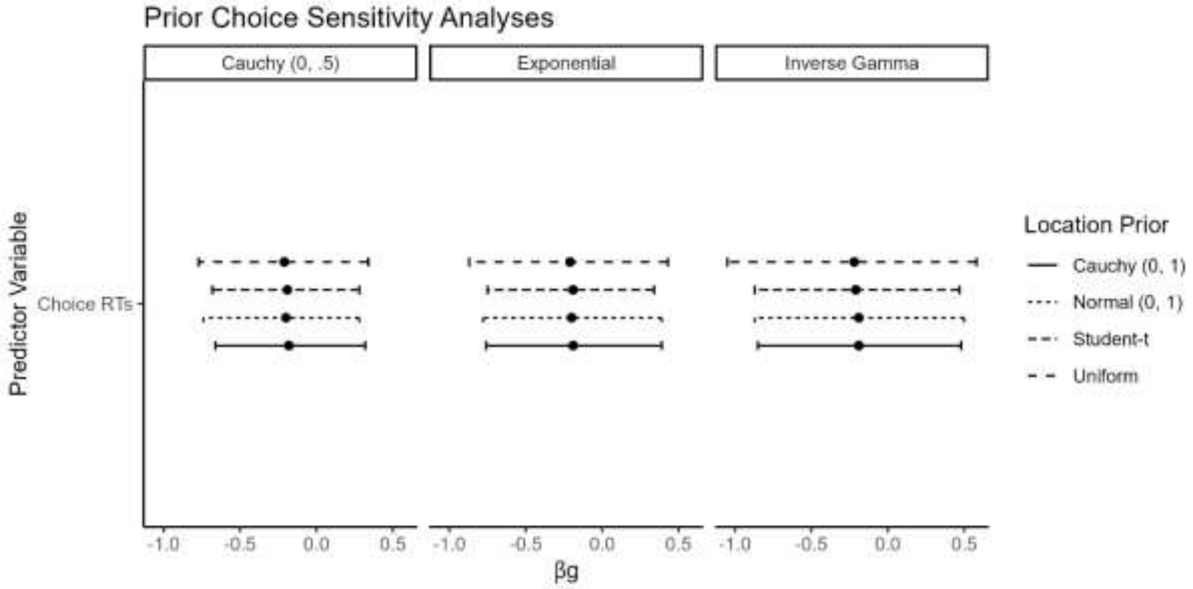

Figure 1. Regression coefficients with 95% confidence intervals for choice reaction times model. For included tests see Supplementary Materials 1.

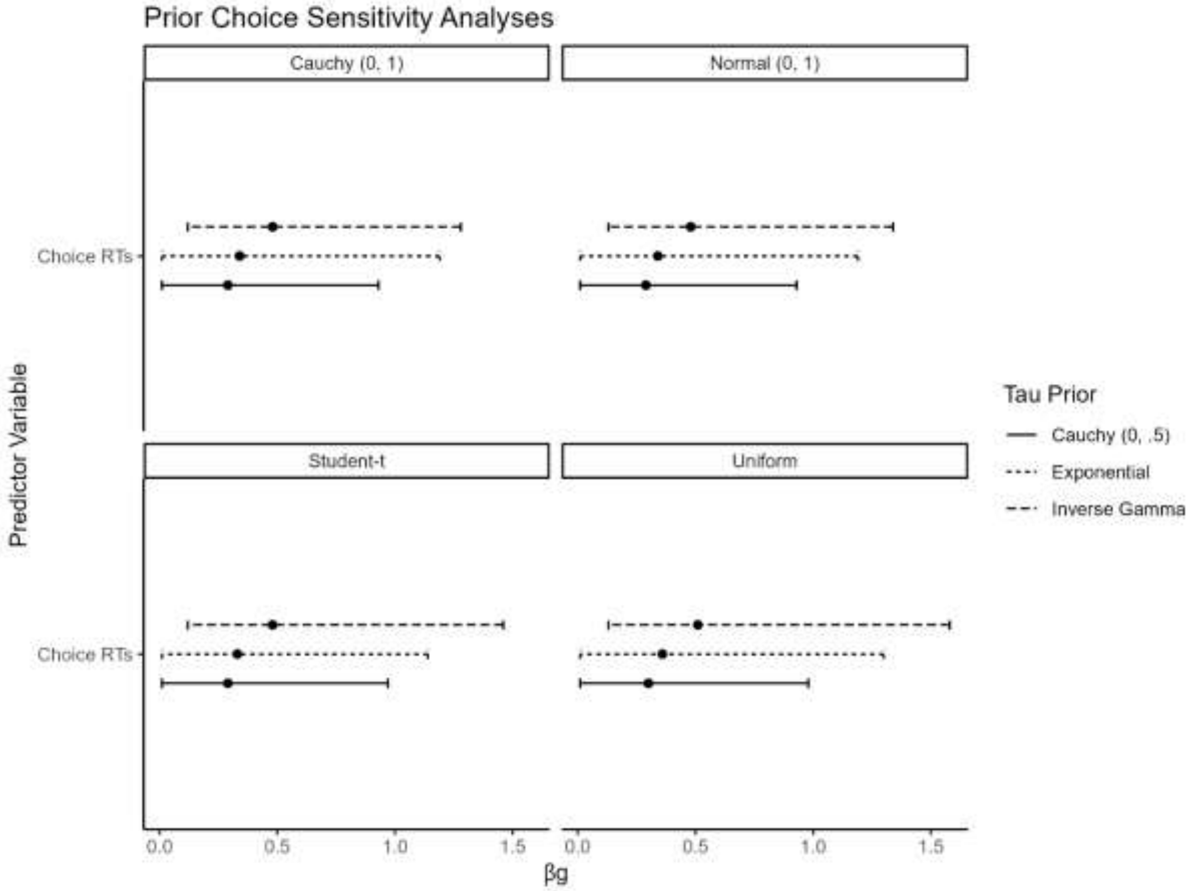

Figure 1. Study level standard deviation estimates with 95% confidence intervals for choice reaction times model. For included tests see Supplementary Materials 1.

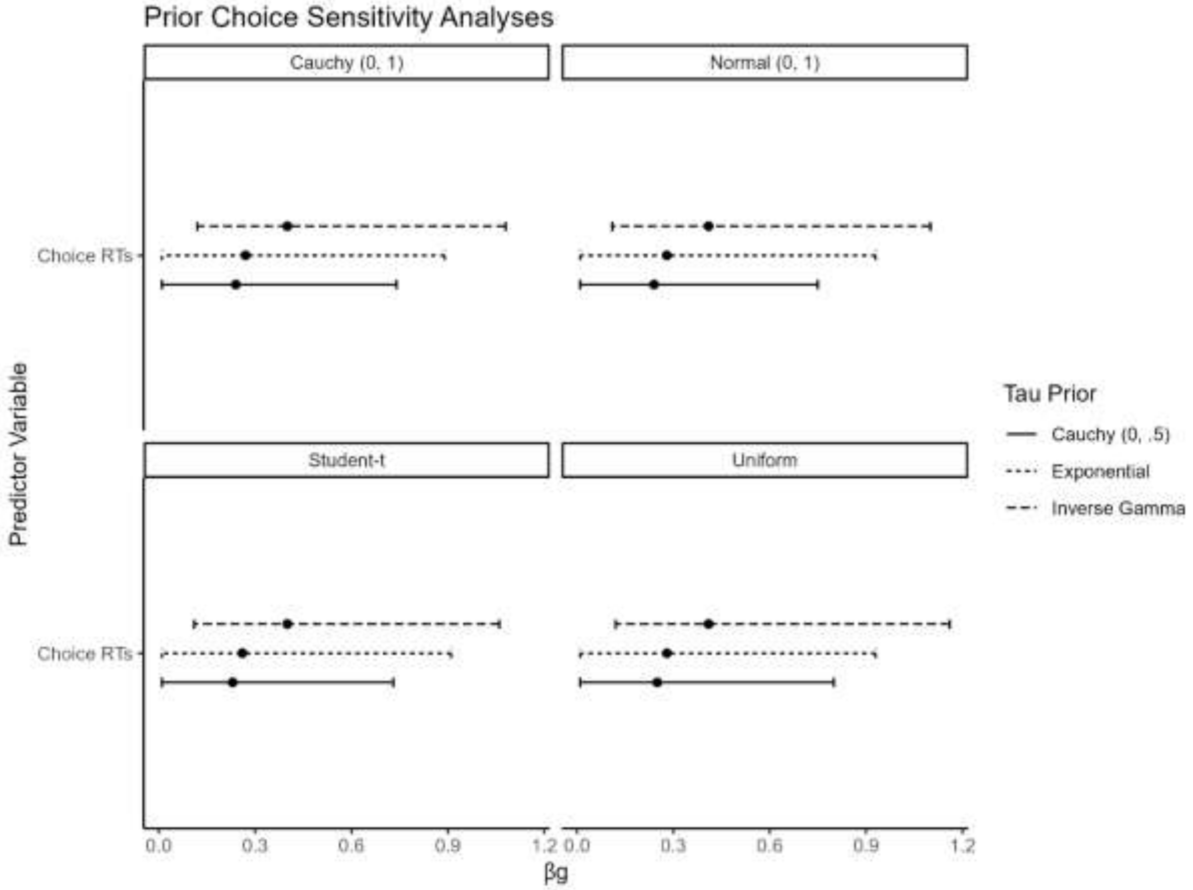

Figure 1. Effect size level standard deviation estimates with 95% confidence intervals for choice reaction times model. For included tests see Supplementary Materials 1.

Other Measures of Selective Attention

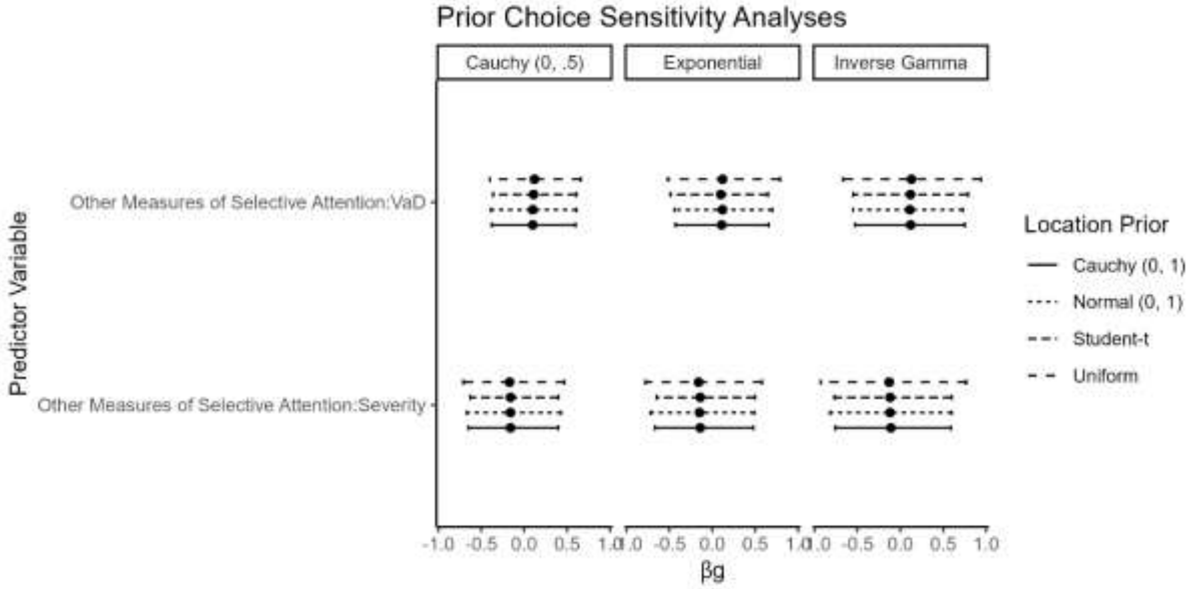

Figure 1. Regression coefficients with 95% confidence intervals for the Other Measures of Selective Attention model. For tests included in the analysis see Supplementary Materials 1. VaD: vascular dementia, Severity: difference in dementia severity between dementia groups.

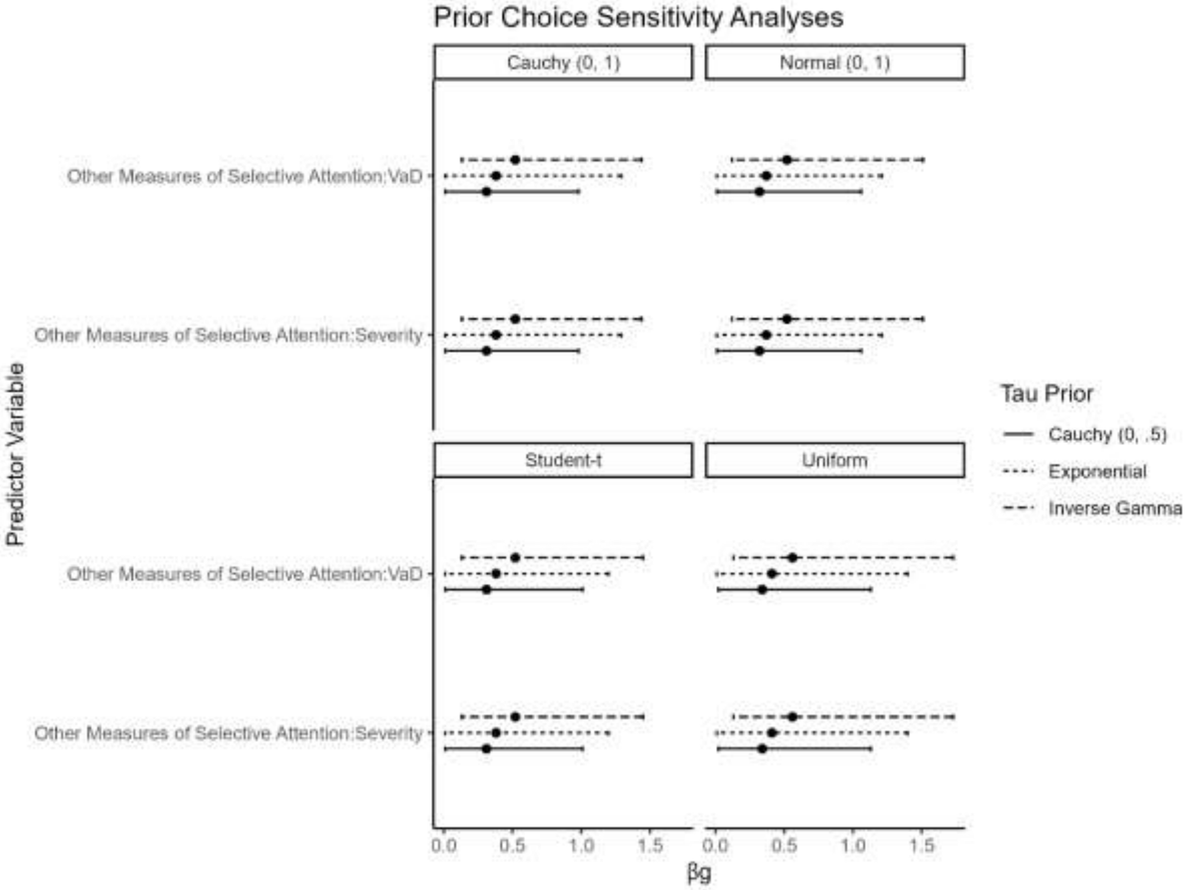

Figure 1. Study level standard deviation estimates with 95% confidence intervals for the Other Measures of Selective Attention model. For tests included in the analysis see Supplementary Materials 1. VaD: vascular dementia, Severity: difference in dementia severity between dementia groups.

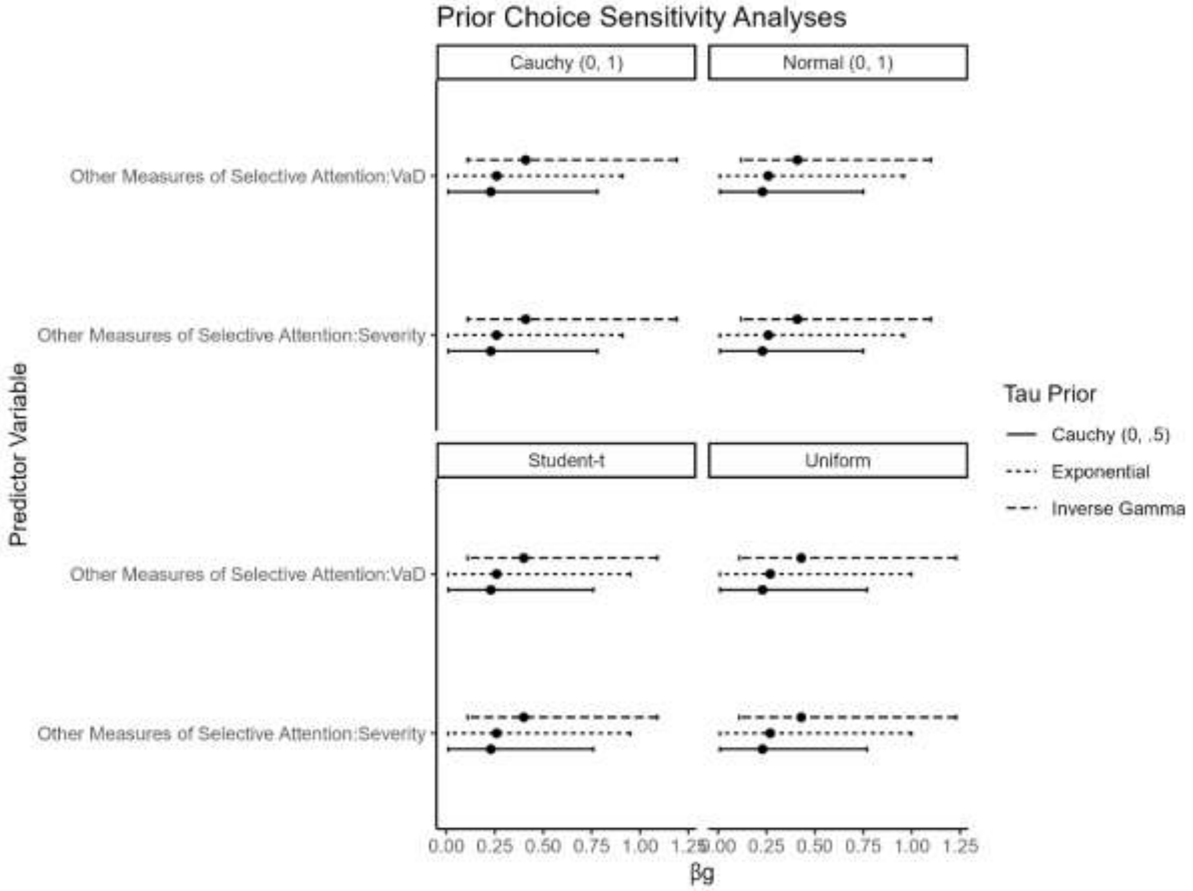

Figure 1. Effect size level standard deviation estimates with 95% confidence intervals for the Other Measures of Selective Attention model. For tests included in the analysis see Supplementary Materials 1. VaD: vascular dementia, Severity: difference in dementia severity between dementia groups.

Continuous Performance Tests

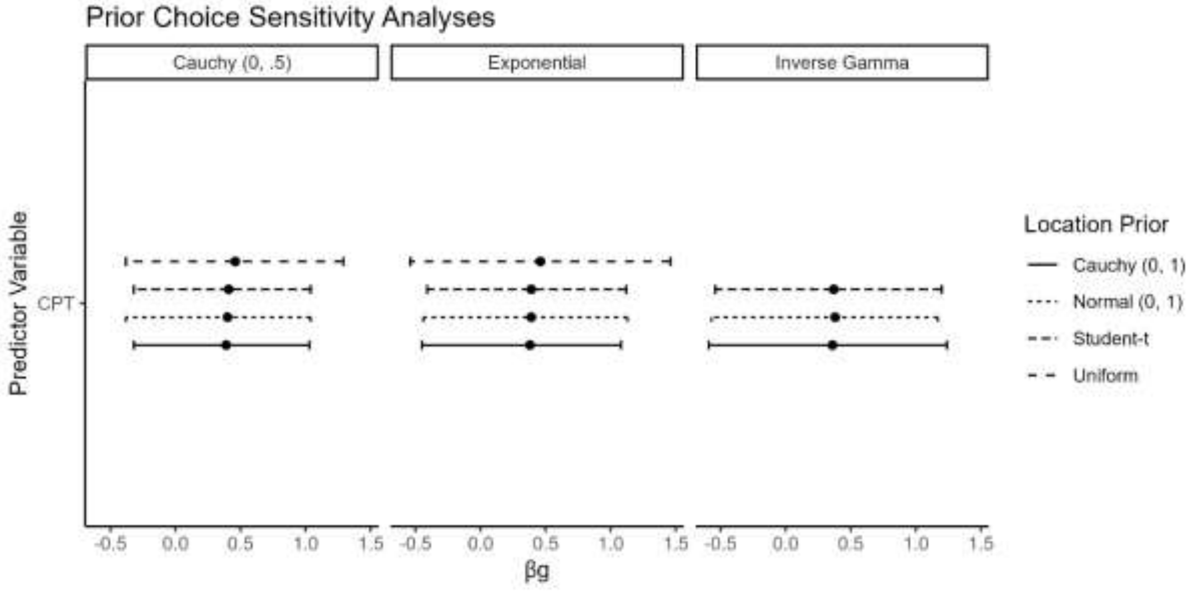

Figure 1. Regression coefficients with 95% confidence intervals for the Continuous Performance Tests model.

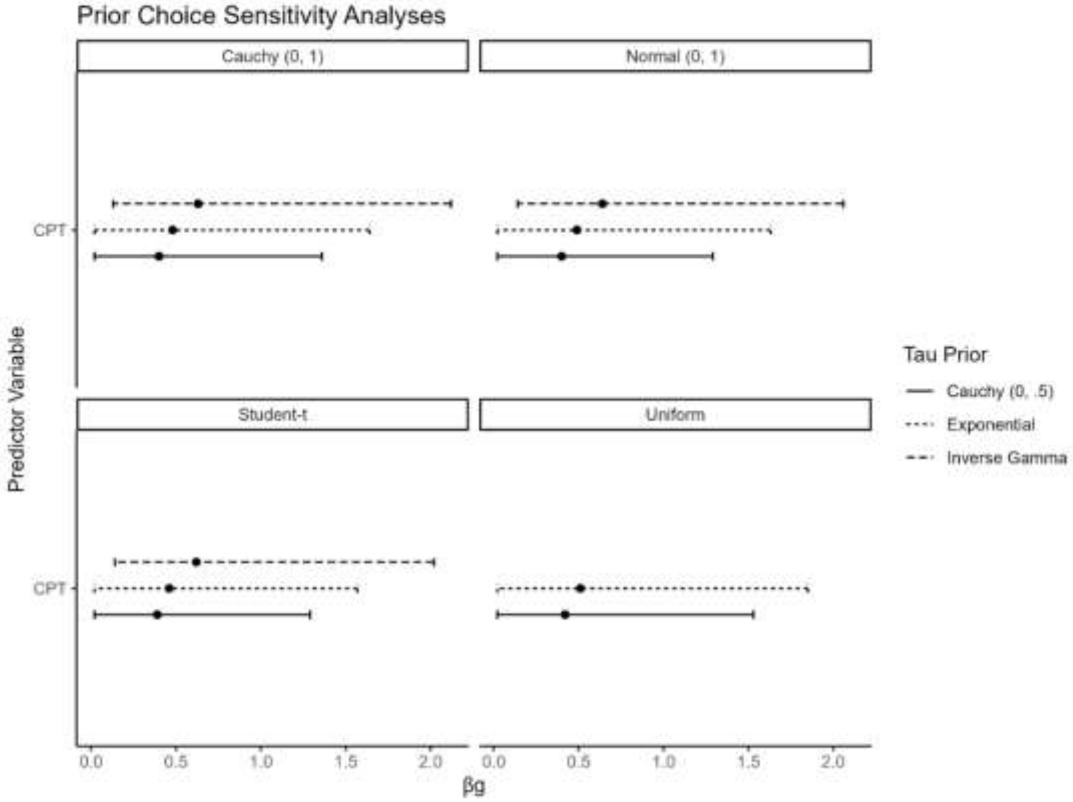

Figure 1. Study level standard deviation estimates with 95% confidence intervals for the Continuous Performance Tests model.

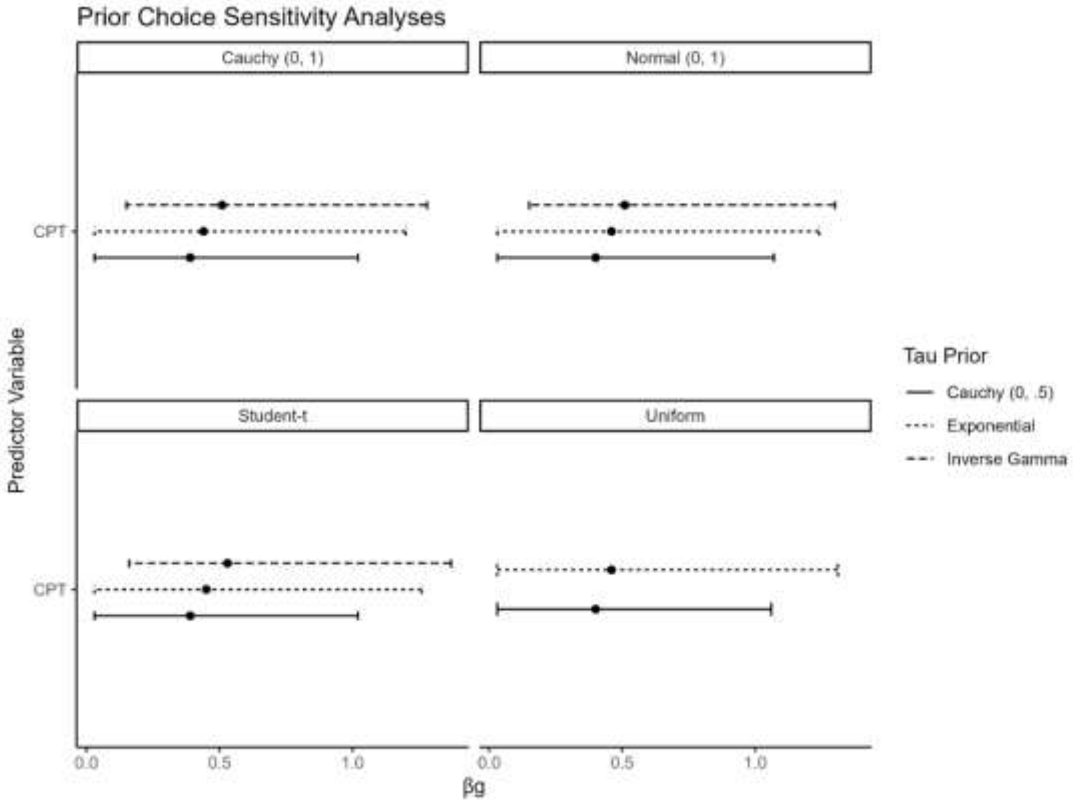

Figure 1. Effect size level standard deviation estimates with 95% confidence intervals for the Continuous Performance Tests model.

Other Measures of Sustained Attention

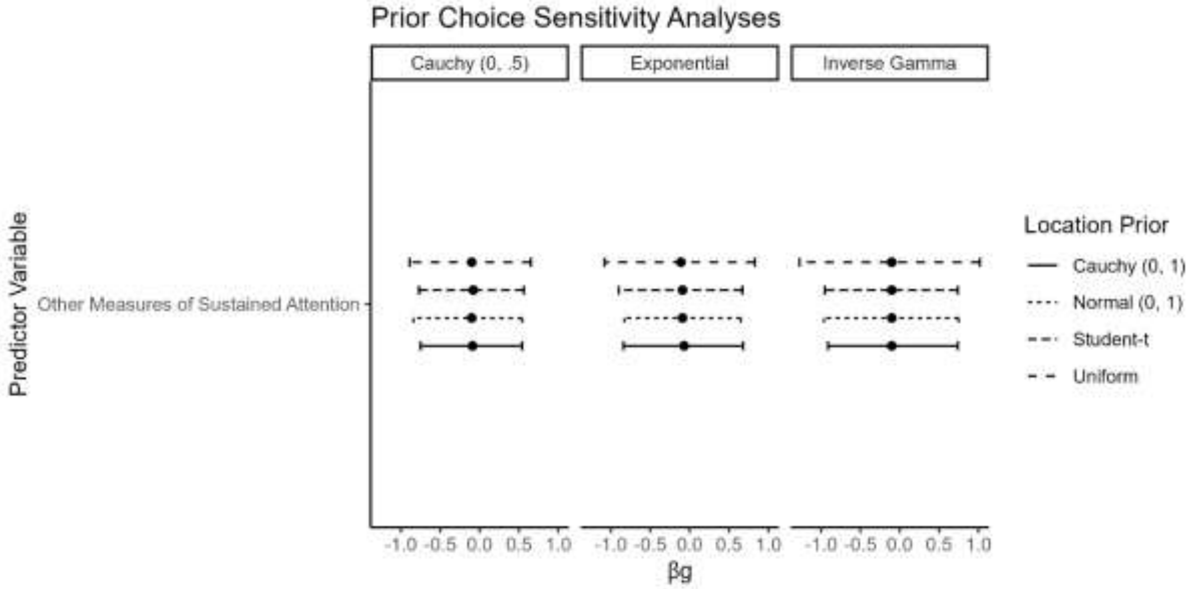

Figure 1. Regression coefficients with 95% confidence intervals for the Other Measures of Sustained Attention model. For tests included in the analysis see Supplementary Materials 1.

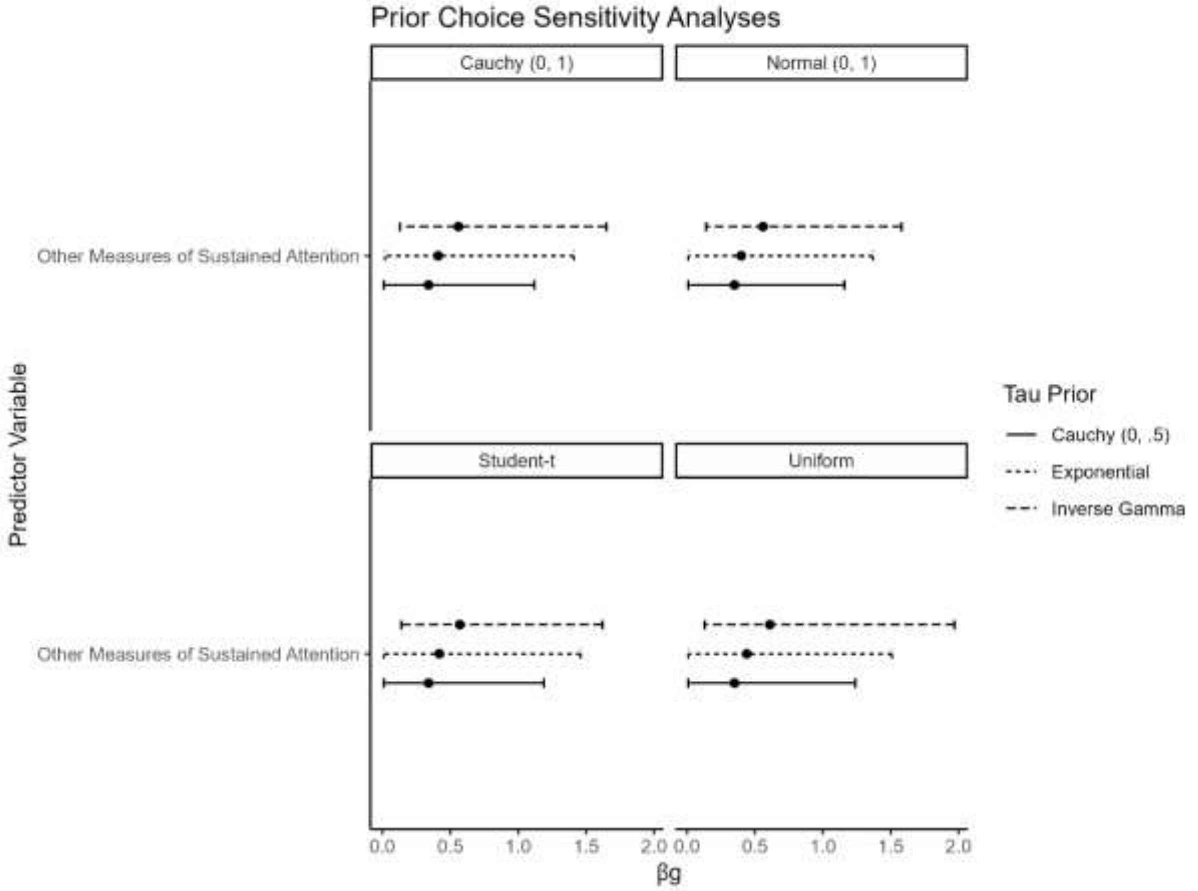

Figure 1. Study level standard deviation estimates with 95% confidence intervals for the Other Measures of Sustained Attention model. For tests included in the analysis see Supplementary Materials 1.

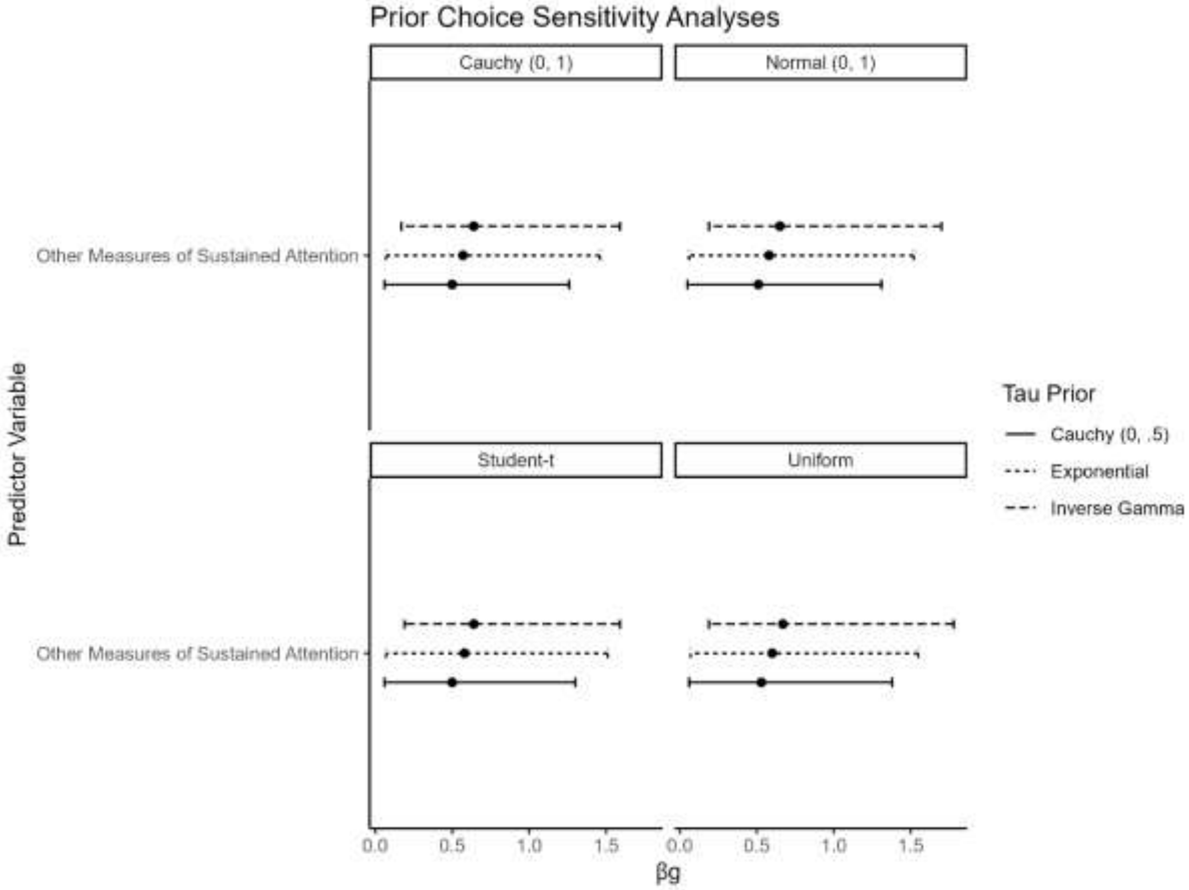

Figure 1. Effect size level standard deviation estimates with 95% confidence intervals for the Other Measures of Sustained Attention model. For tests included in the analysis see Supplementary Materials 1.

Other Measures of Visual Attention

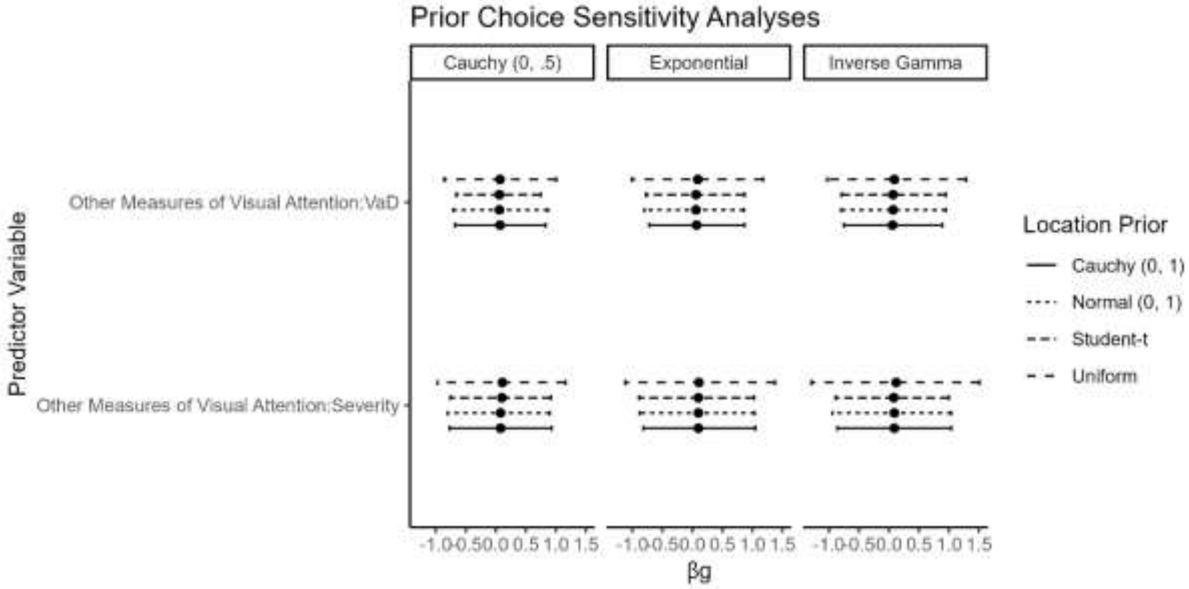

Figure 1. Regression coefficients with 95% confidence intervals for the Other Measures of Visual Attention model. For tests included in the analysis see Supplementary Materials 1. VaD: vascular dementia, Severity: difference in dementia severity between dementia groups.

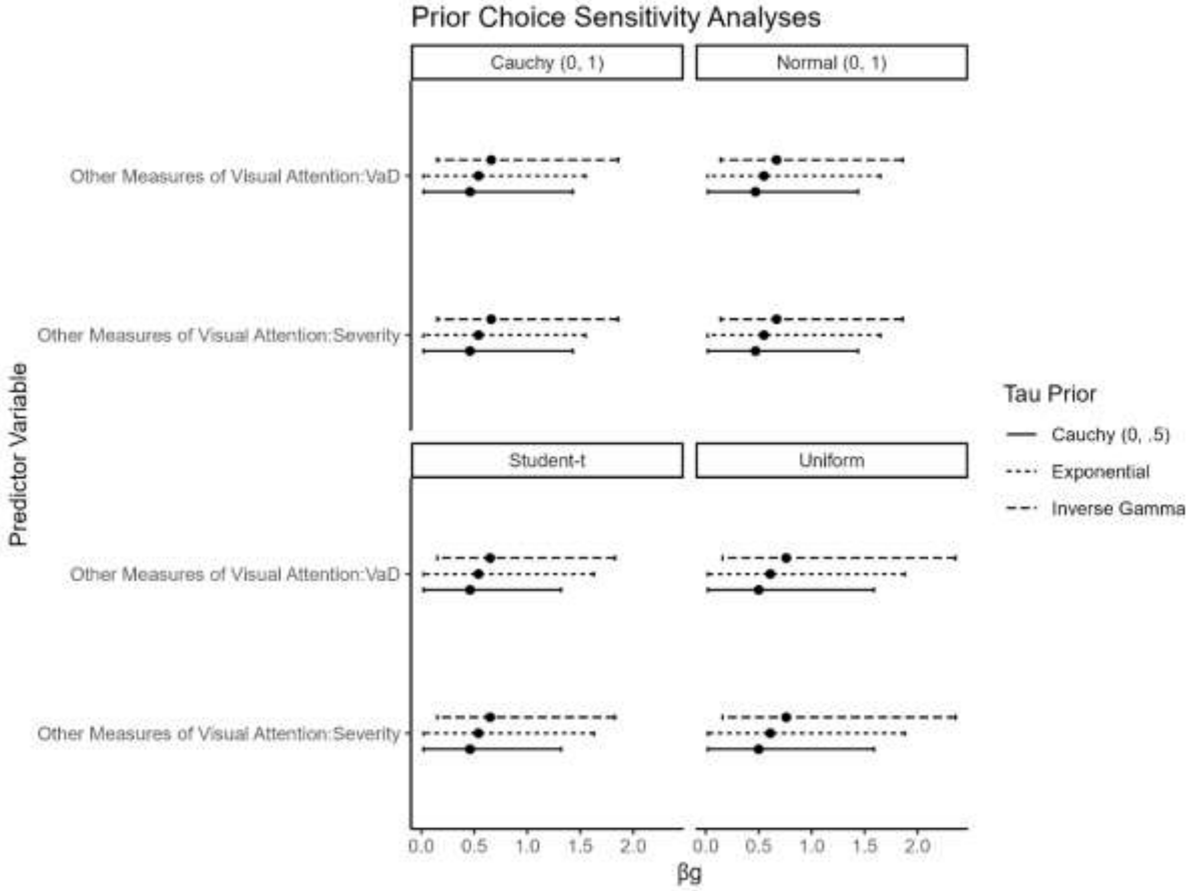

Figure 1. Study level standard deviation estimates with 95% confidence intervals for the Other Measures of Visual Attention model. For tests included in the analysis see Supplementary Materials 1. VaD: vascular dementia, Severity: difference in dementia severity between dementia groups.

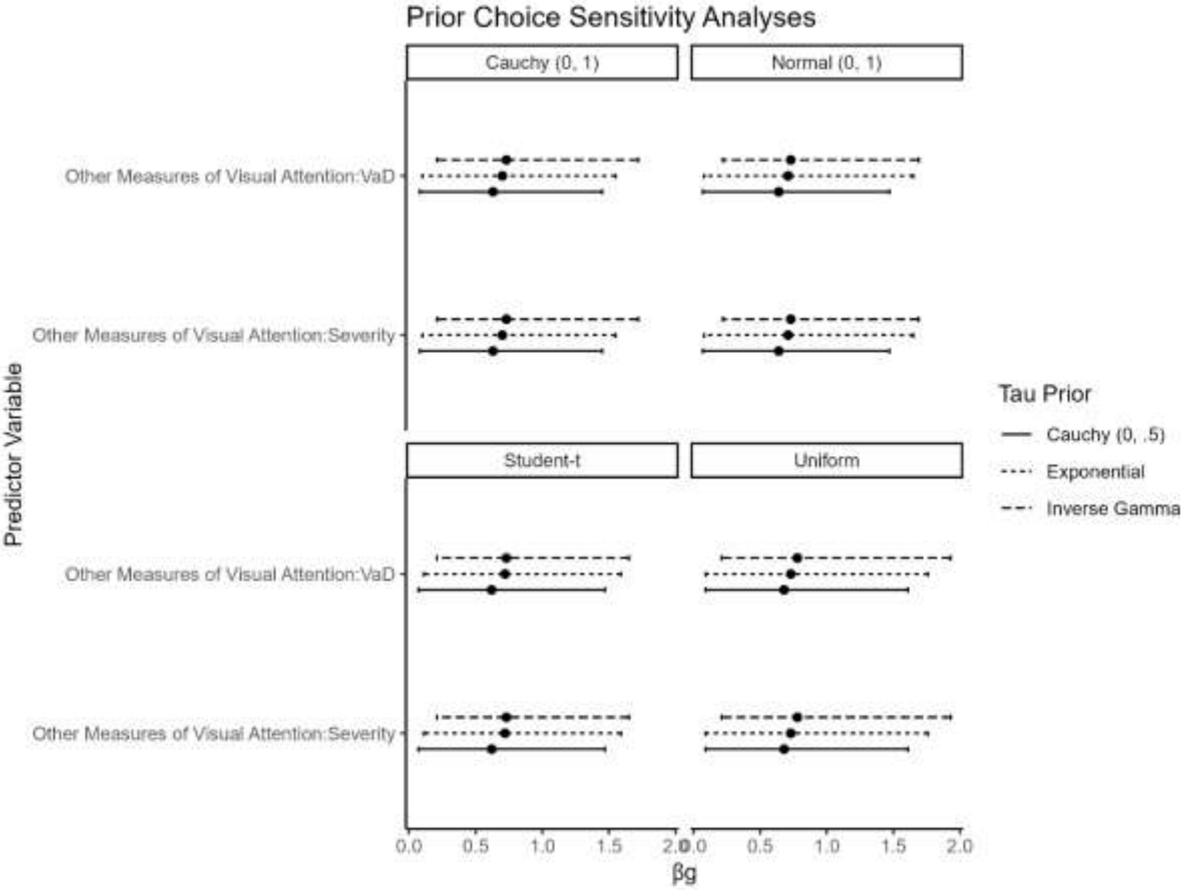

Figure 1. Effect size level standard deviation estimates with 95% confidence intervals for the Other Measures of Visual Attention model. For tests included in the analysis see Supplementary Materials 1. VaD: vascular dementia, Severity: difference in dementia severity between dementia groups.

Other Measures of Attention

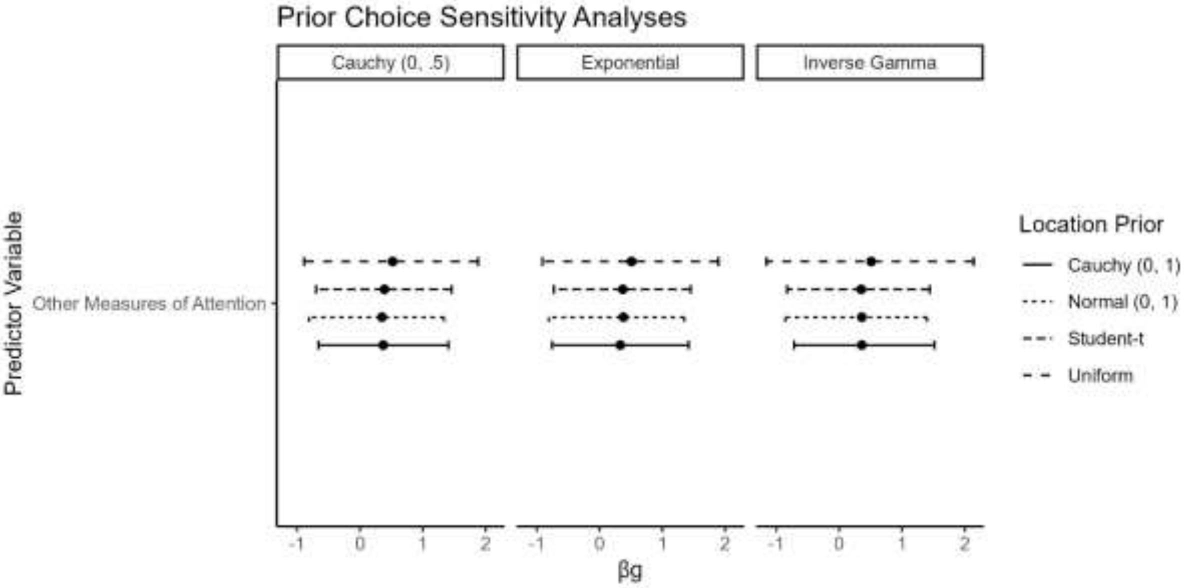

Figure 1. Regression coefficients with 95% confidence intervals for the Other Measures of Attention model. For tests included in the analysis see Supplementary Materials 1.

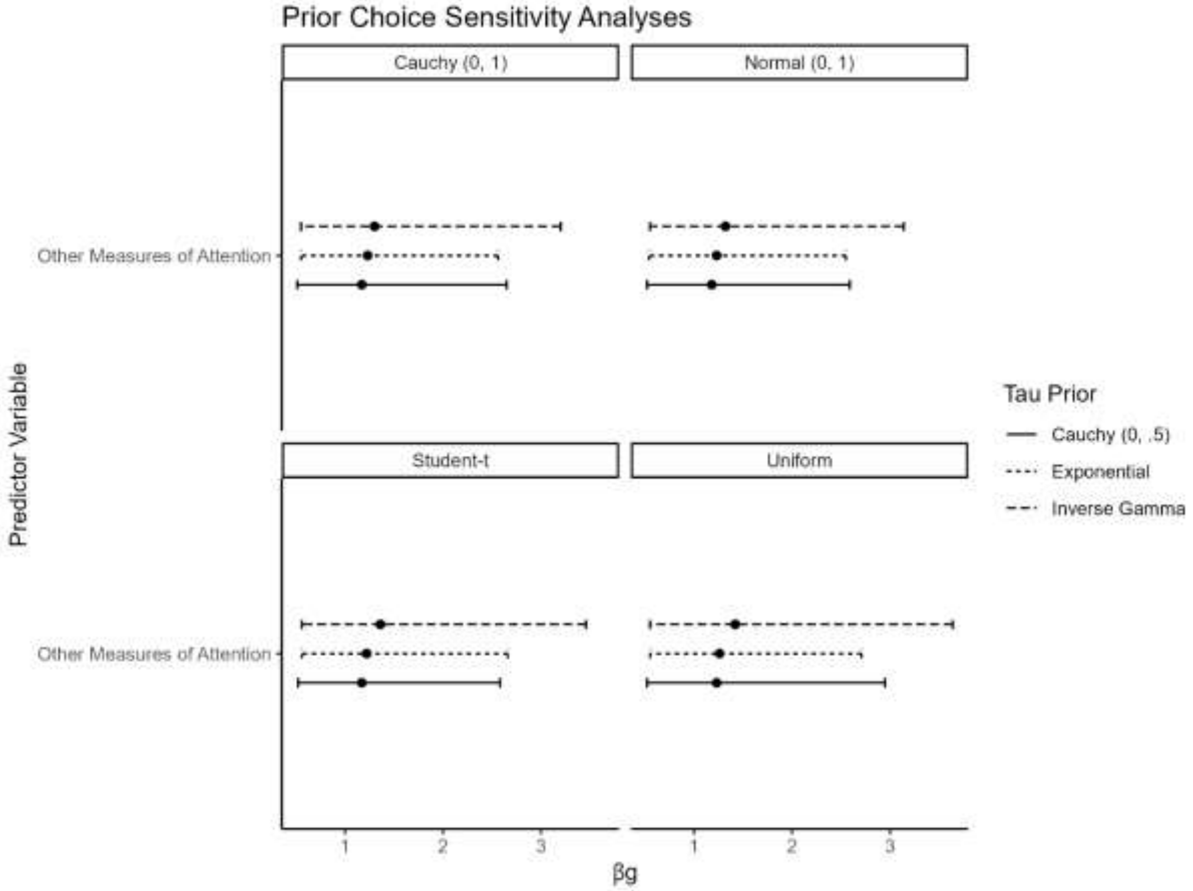

Figure 1. Study level standard deviation estimates with 95% confidence intervals for the Other Measures of Attention model. For tests included in the analysis see Supplementary Materials 1.

Attention: Quality Sensitivity Analysis

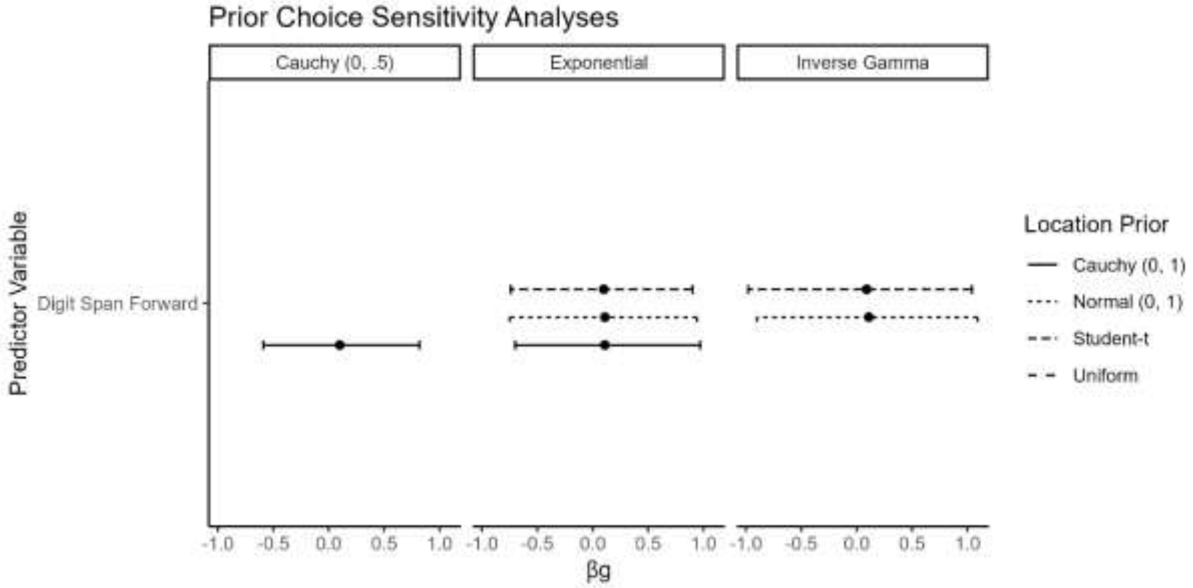

Figure 1. Regression coefficients with 95% confidence intervals for the study quality sensitivity analysis of Digit Span Forward measures model. For included tests see Supplementary Materials 1.

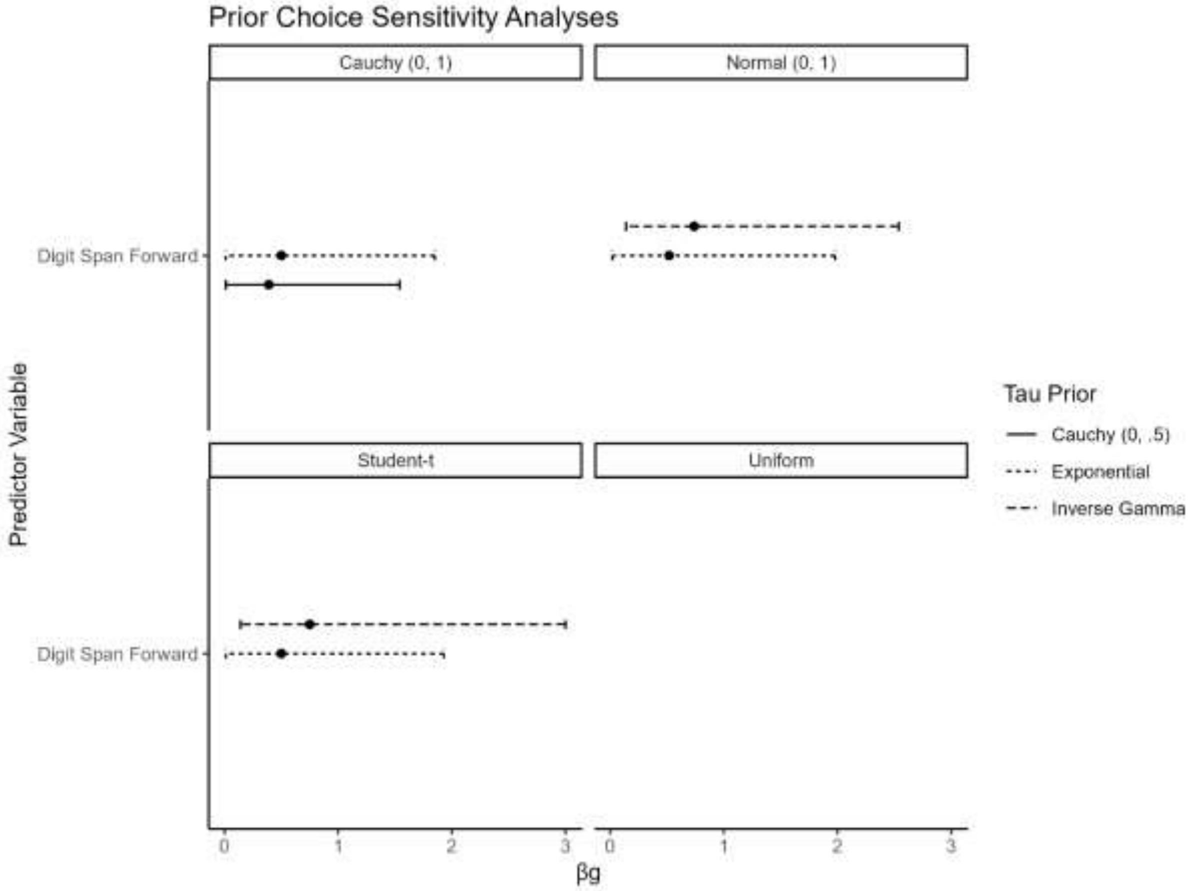

Figure 1. Study level standard deviation estimates with 95% confidence intervals for the study quality sensitivity analysis of Digit Span Forward measures model. For included tests see Supplementary Materials 1.

Processing Speed

Stroop Test Word Reading and Colour Naming

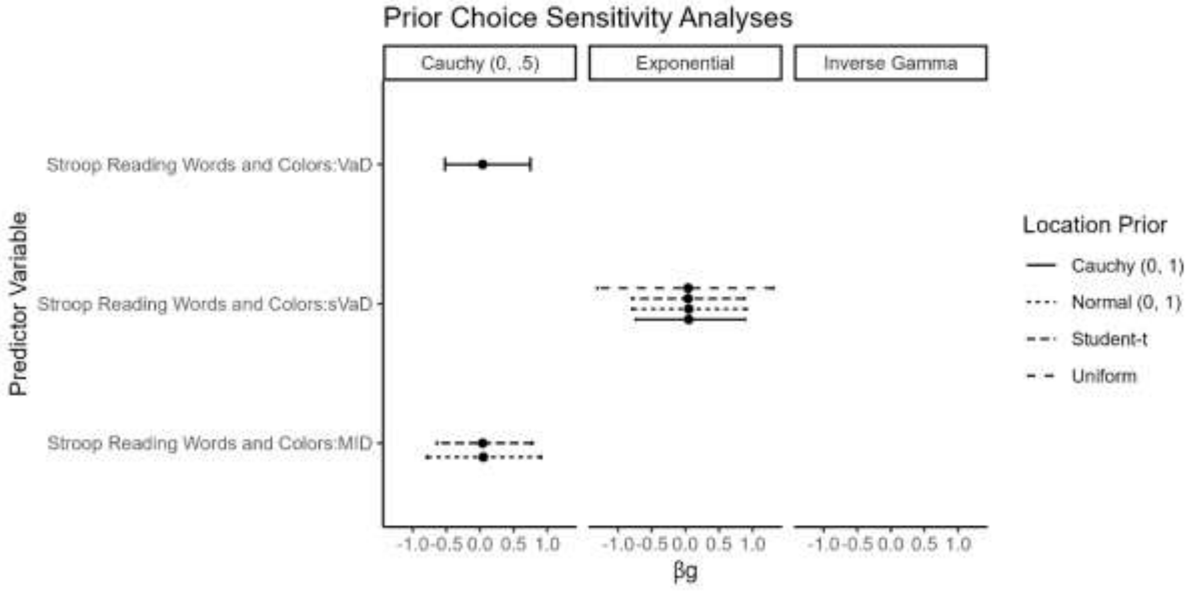

Figure 1. Regression coefficients with 95% confidence intervals for the Stroop Test Word reading and Colour Naming model. sVaD: subcortical vascular dementia, VaD: vascular dementia, MID: multi-infarct dementia.

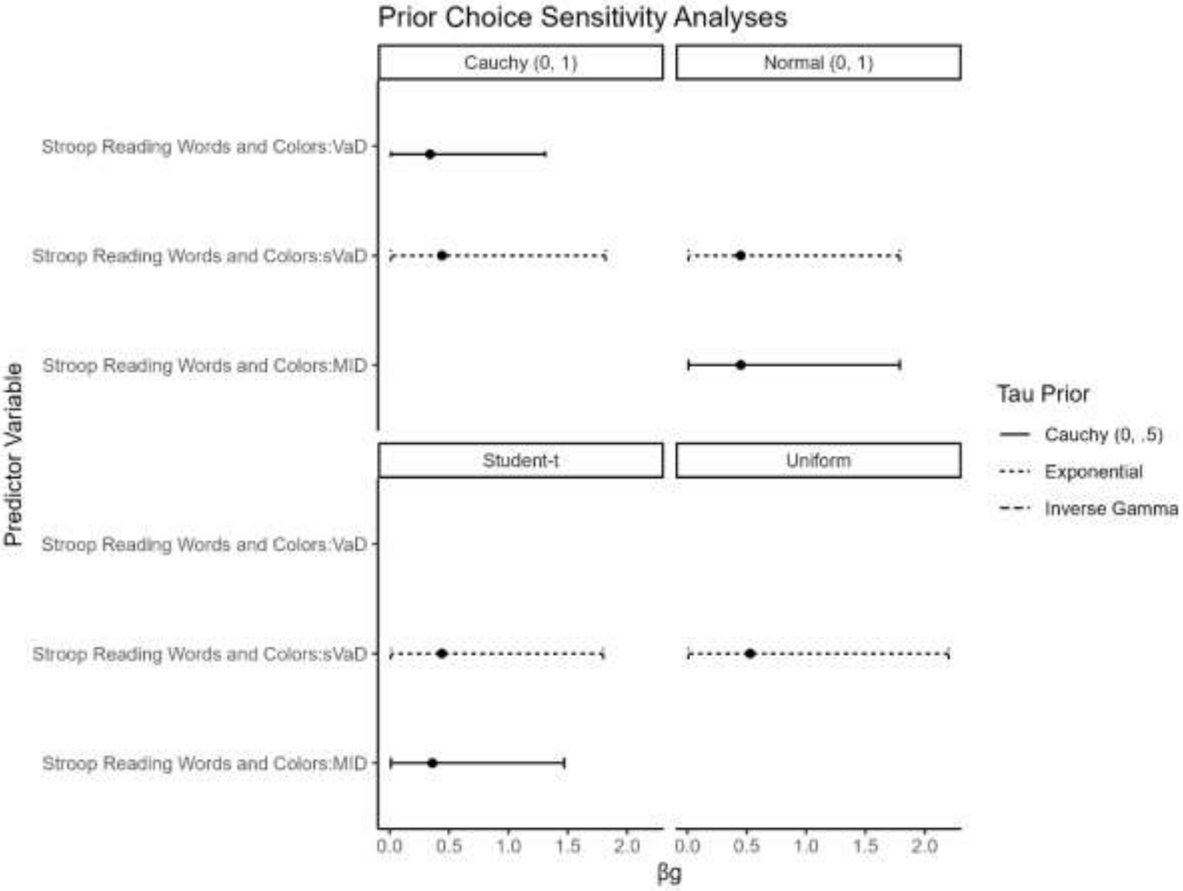

Figure 1. Study level standard deviation estimates with 95% confidence intervals for the Stroop Test Word reading and Colour Naming model. sVaD: subcortical vascular dementia, VaD: vascular dementia, MID: multi-infarct dementia.

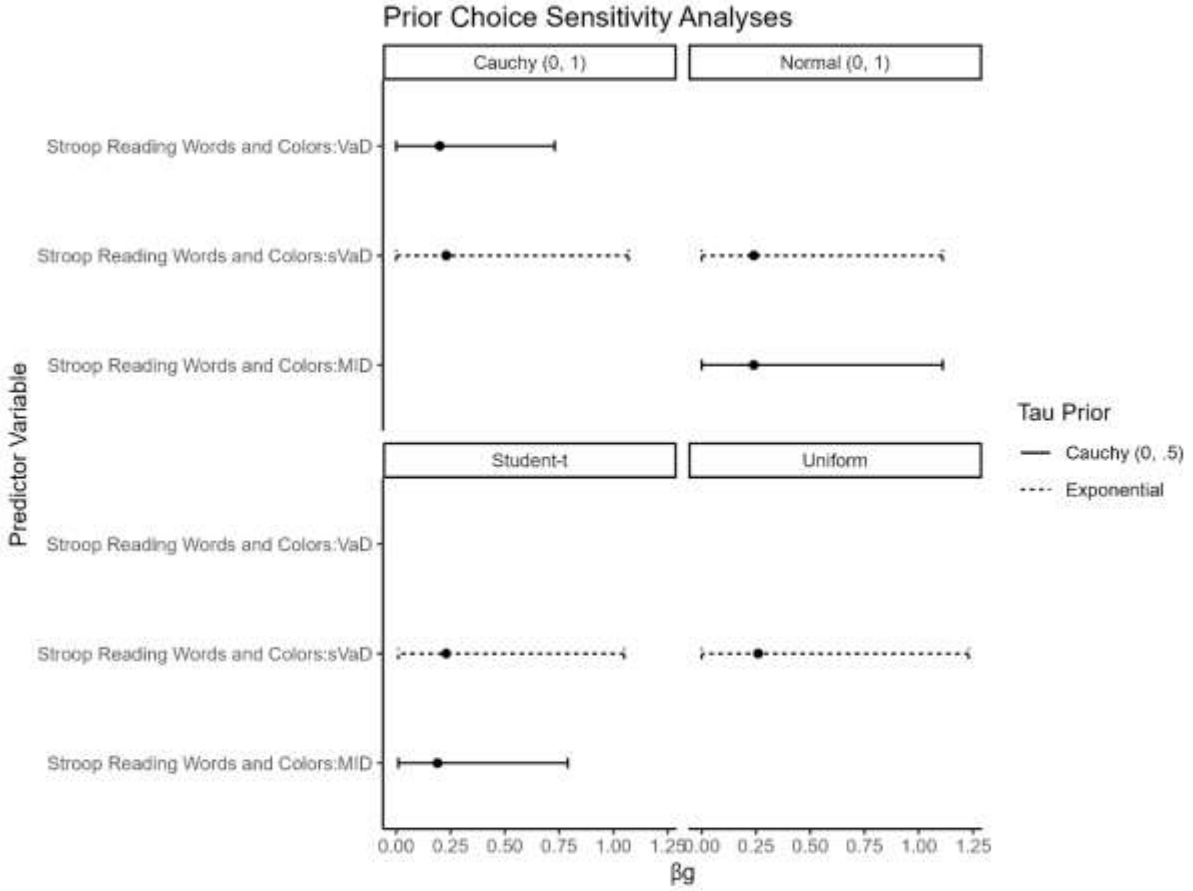

Figure 1. Effect size level standard deviation estimates with 95% confidence intervals for the Stroop Test Word reading and Colour Naming model. sVaD: subcortical vascular dementia, VaD: vascular dementia, MID: multi-infarct dementia.

Simple Reaction Time

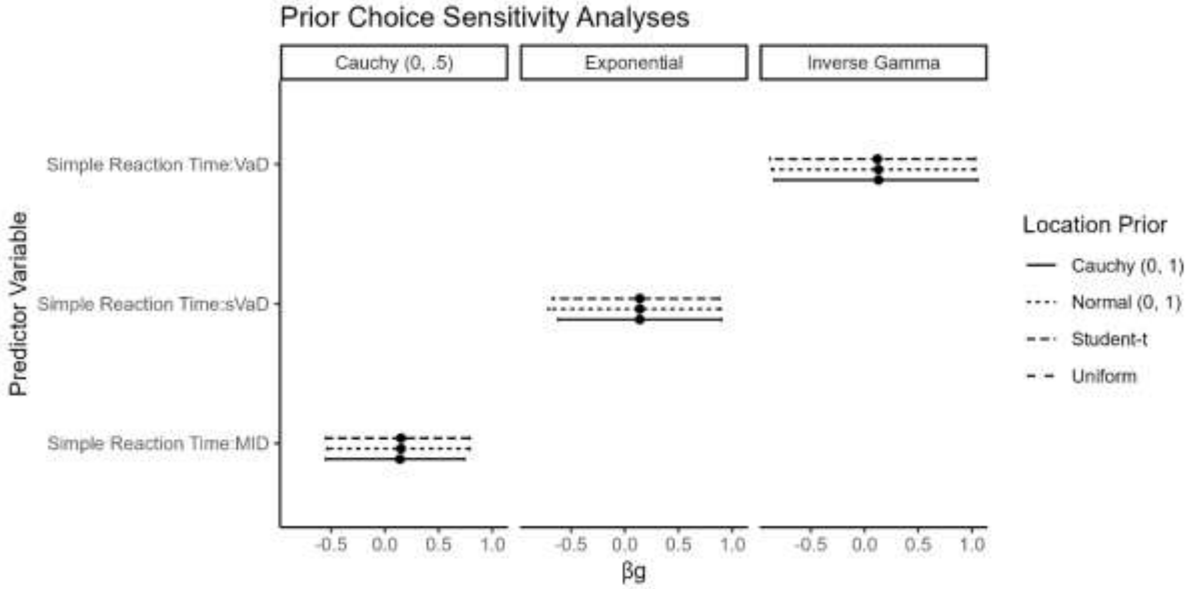

Figure 1. Regression coefficients with 95% confidence intervals for the Simple Reaction Time model. For test included see Supplementary Materials 1. sVaD: subcortical vascular dementia, VaD: vascular dementia, MID: multi-infarct dementia.

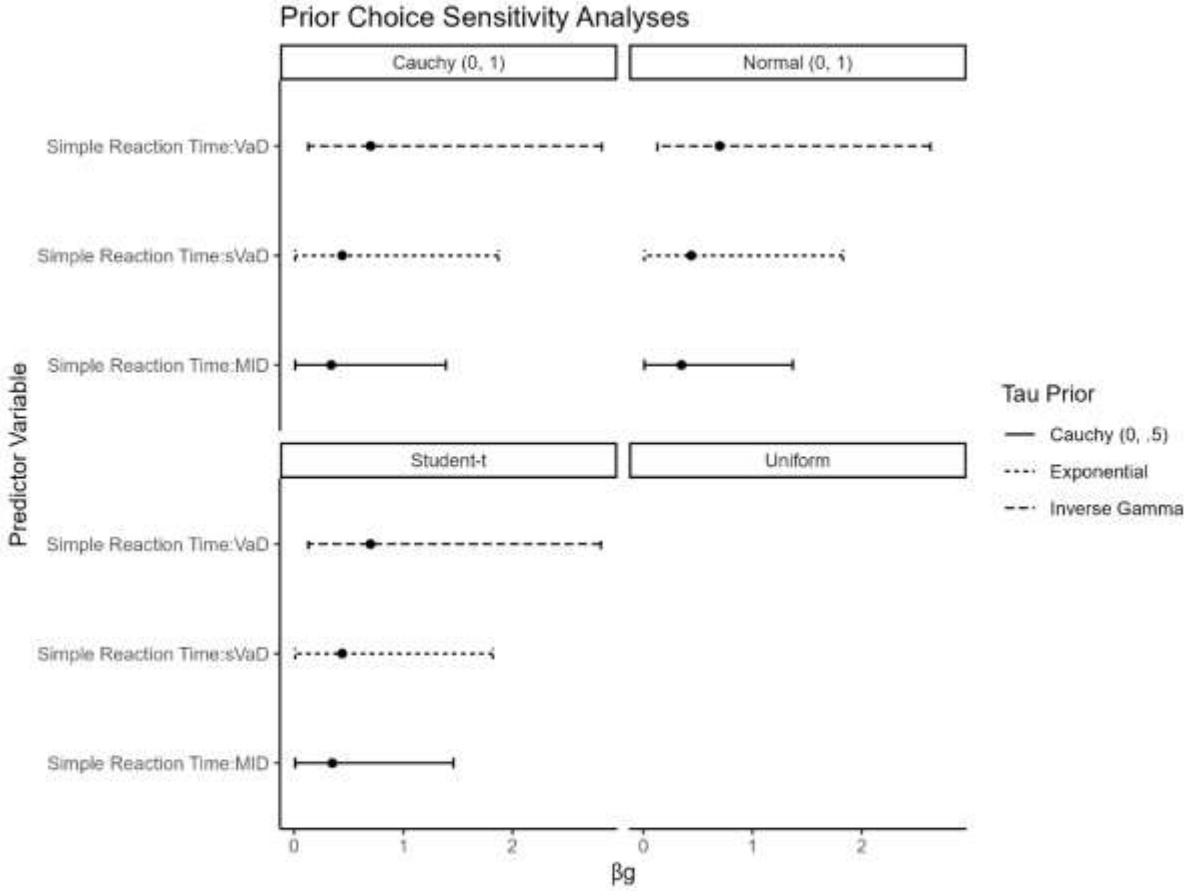

Figure 1. Study level standard deviation estimates with 95% confidence intervals for the Simple Reaction Time model. For test included see Supplementary Materials 1. sVaD: subcortical vascular dementia, VaD: vascular dementia, MID: multi-infarct dementia.

Other Measures of Processing Speed

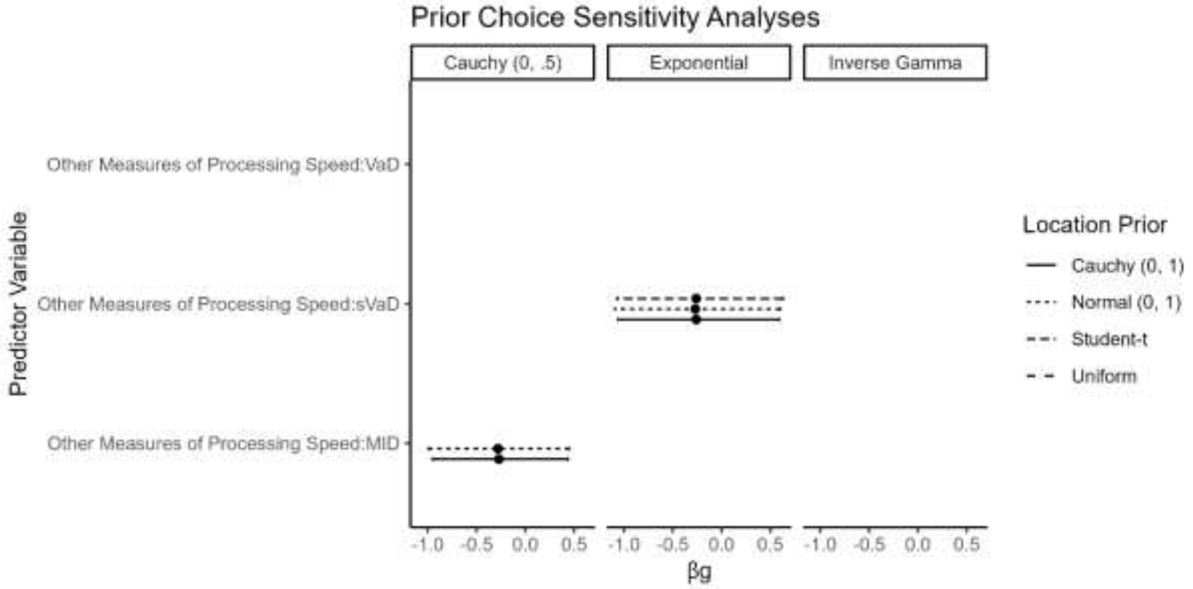

Figure 1. Regression coefficients with 95% confidence intervals for the Other Measures of Processing Speed model. For tests included in the analysis see Supplementary Materials 1. sVaD: subcortical vascular dementia, VaD: vascular dementia, MID: multi-infarct dementia.

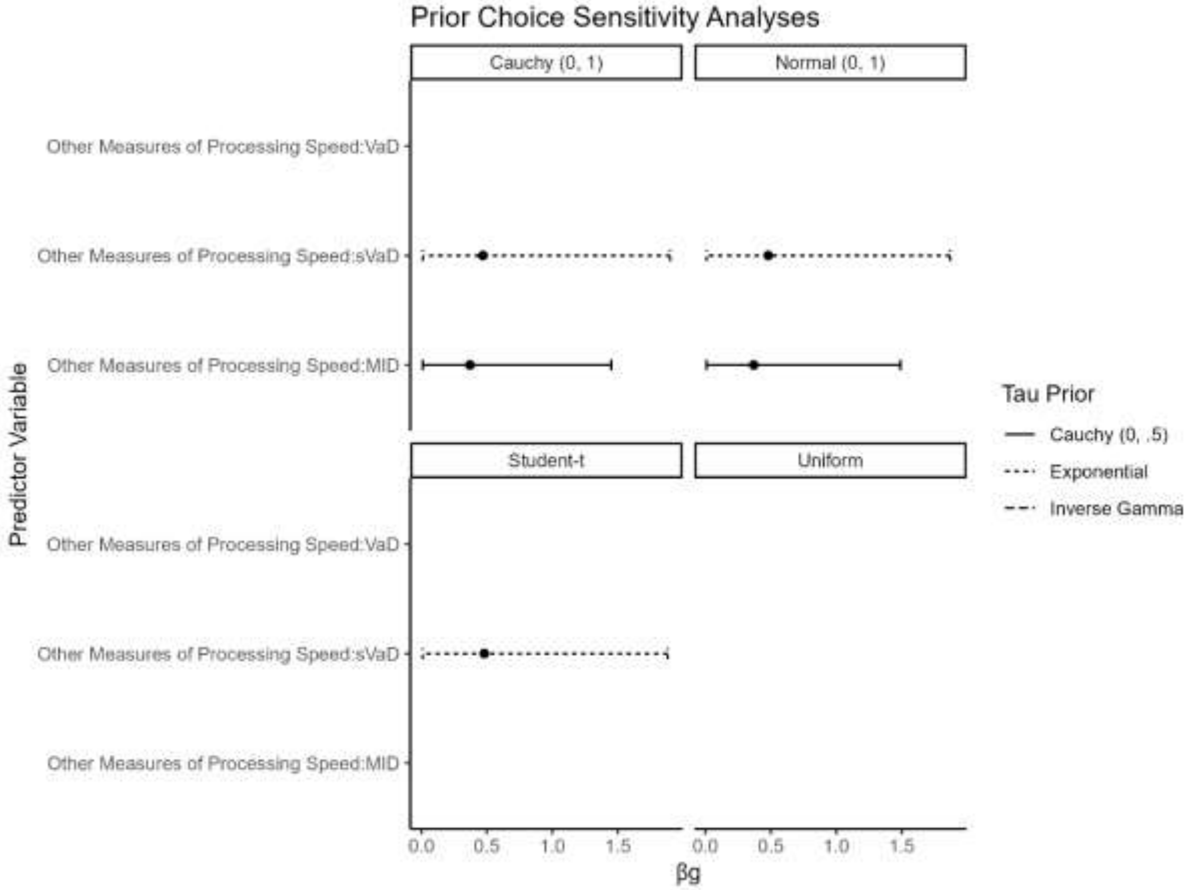

Figure 1. Study level standard deviation estimates with 95% confidence intervals for the Other Measures of Processing Speed model. For tests included in the analysis see Supplementary Materials 1. sVaD: subcortical vascular dementia, VaD: vascular dementia, MID: multi-infarct dementia.

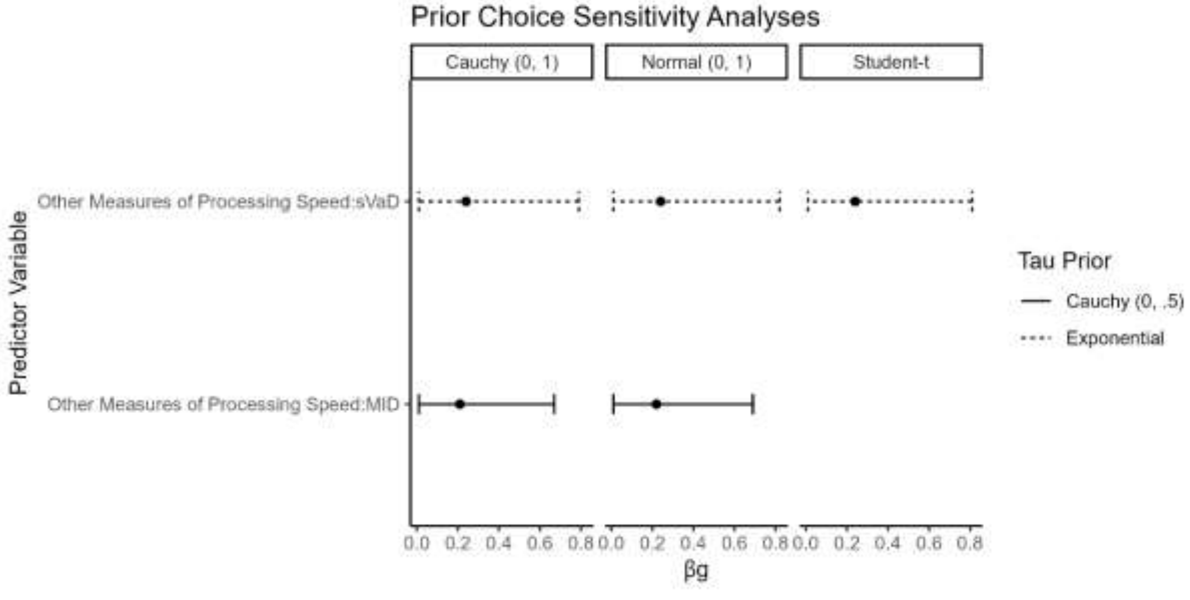

Figure 1. Effect size level standard deviation estimates with 95% confidence intervals for the Other Measures of Processing Speed model. For tests included in the analysis see Supplementary Materials 1. sVaD: subcortical vascular dementia, VaD: vascular dementia, MID: multi-infarct dementia.

Language Production

Fluency

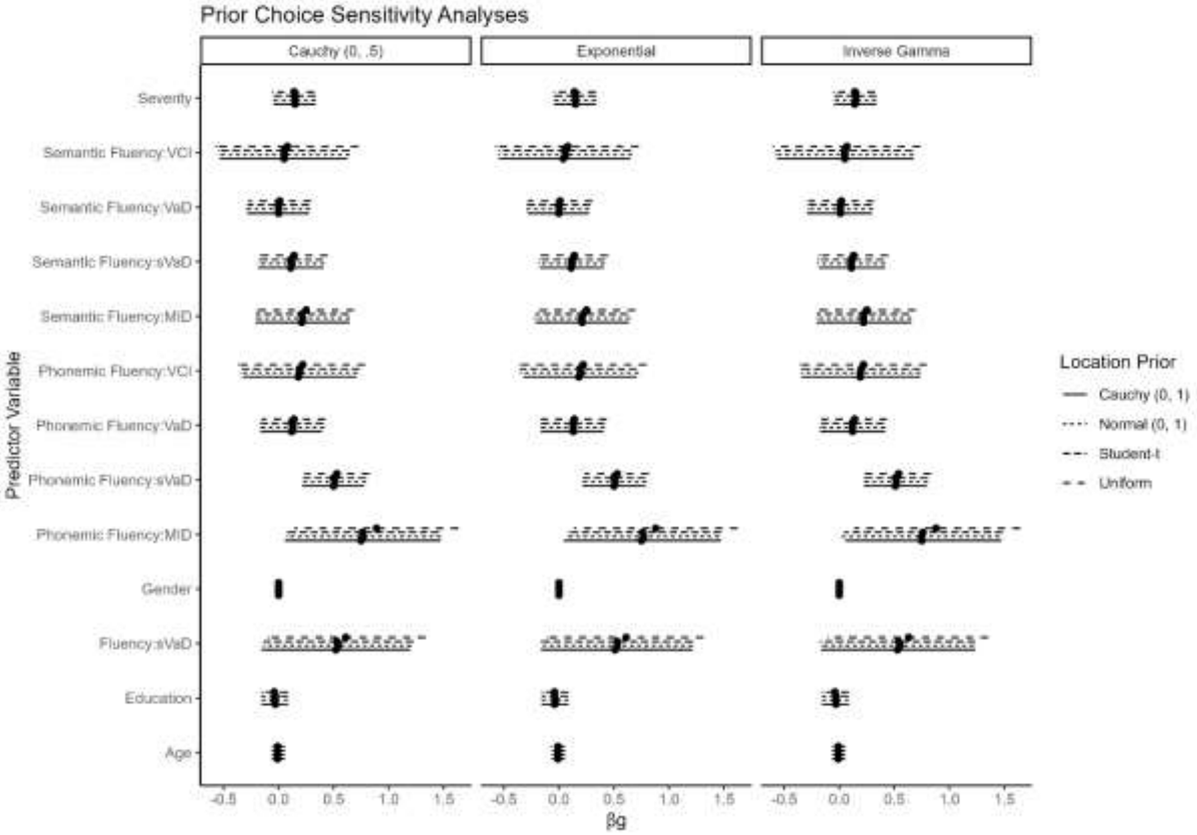

Figure 1. Regression coefficients with 95% confidence intervals for the Measures of Verbal Fluency model. sVaD: subcortical vascular dementia, VaD: vascular dementia, MID: multi-infarct dementia, Severity: difference in dementia severity between dementia groups, Education: difference in average years of education between dementia groups, Age: difference in average age between dementia groups, Gender: difference in proportion of women between dementia groups.

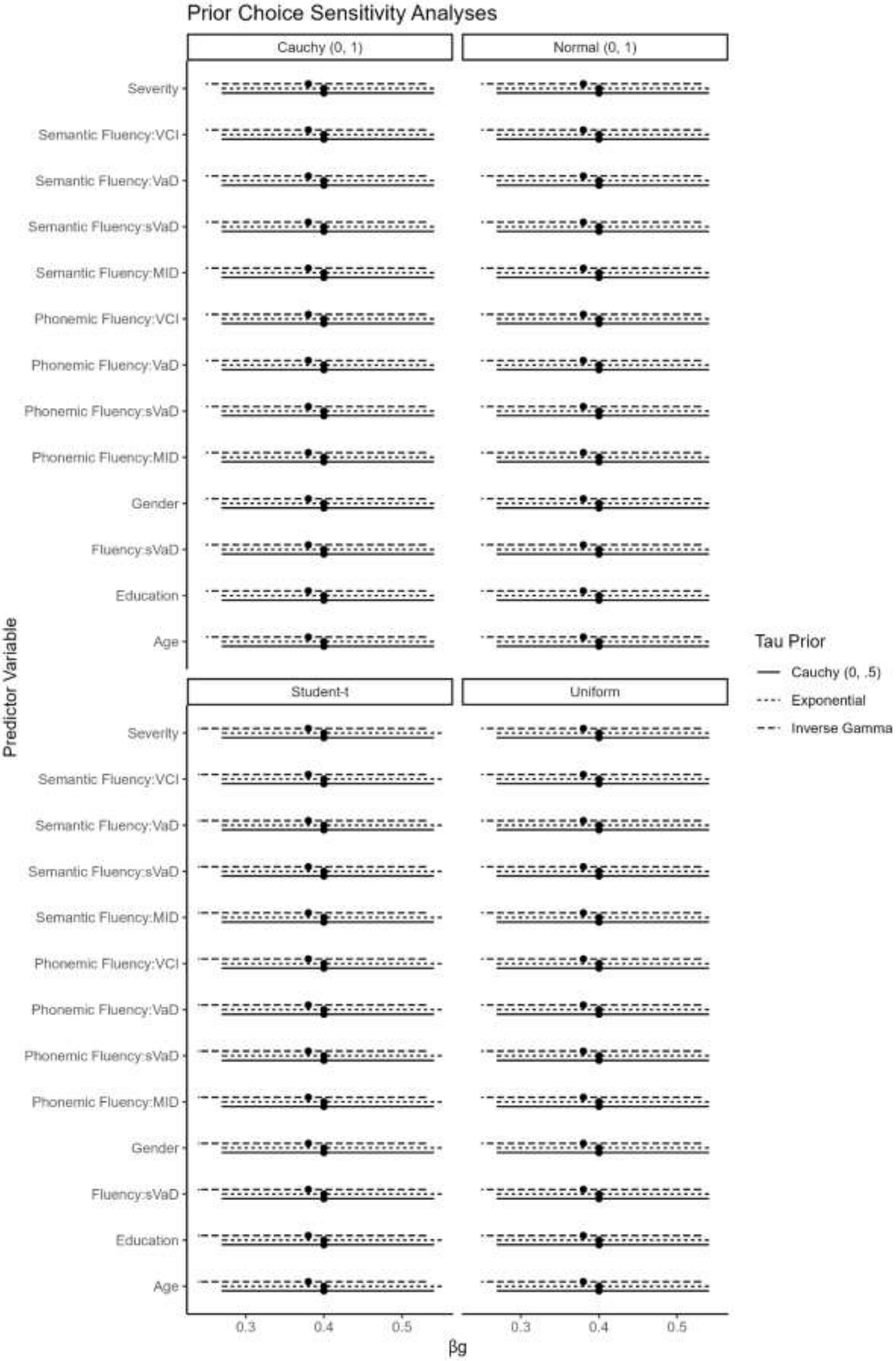

Figure 1. Study level standard deviation estimateswith 95% confidence intervals for the Measures of Verbal Fluency model. sVaD: subcortical vascular dementia, VaD: vascular

dementia, MID: multi-infarct dementia, Severity: difference in dementia severity between dementia groups, Education: difference in average years of education between dementia groups, Age: difference in average age between dementia groups, Gender: difference in proportion of women between dementia groups.

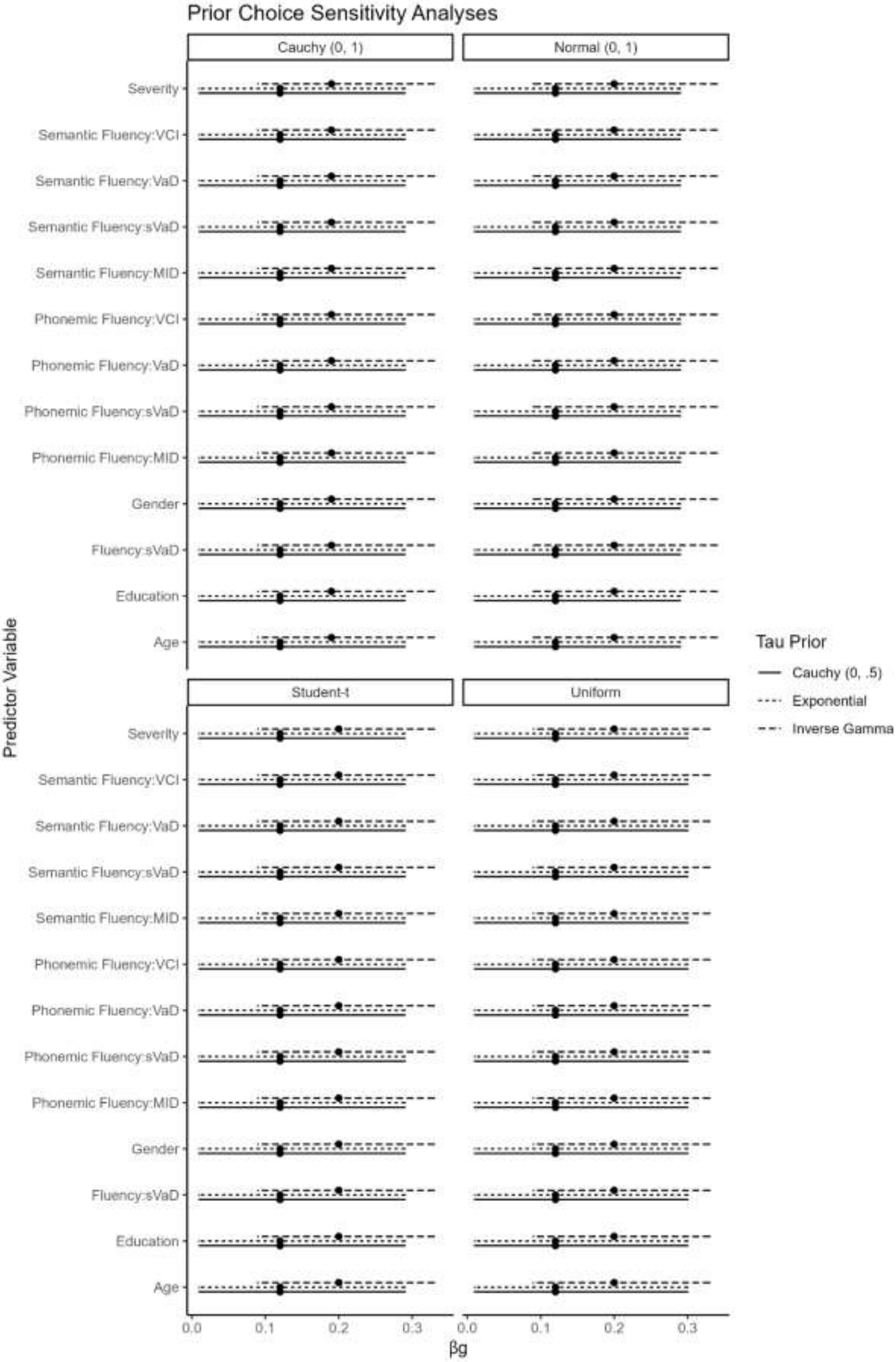

Figure 1. Effect size level standard deviation estimateswith 95% confidence intervals for the Measures of Verbal Fluency model. sVaD: subcortical vascular dementia, VaD: vascular

dementia, MID: multi-infarct dementia, Severity: difference in dementia severity between dementia groups, Education: difference in average years of education between dementia groups, Age: difference in average age between dementia groups, Gender: difference in proportion of women between dementia groups.

Boston Naming Test

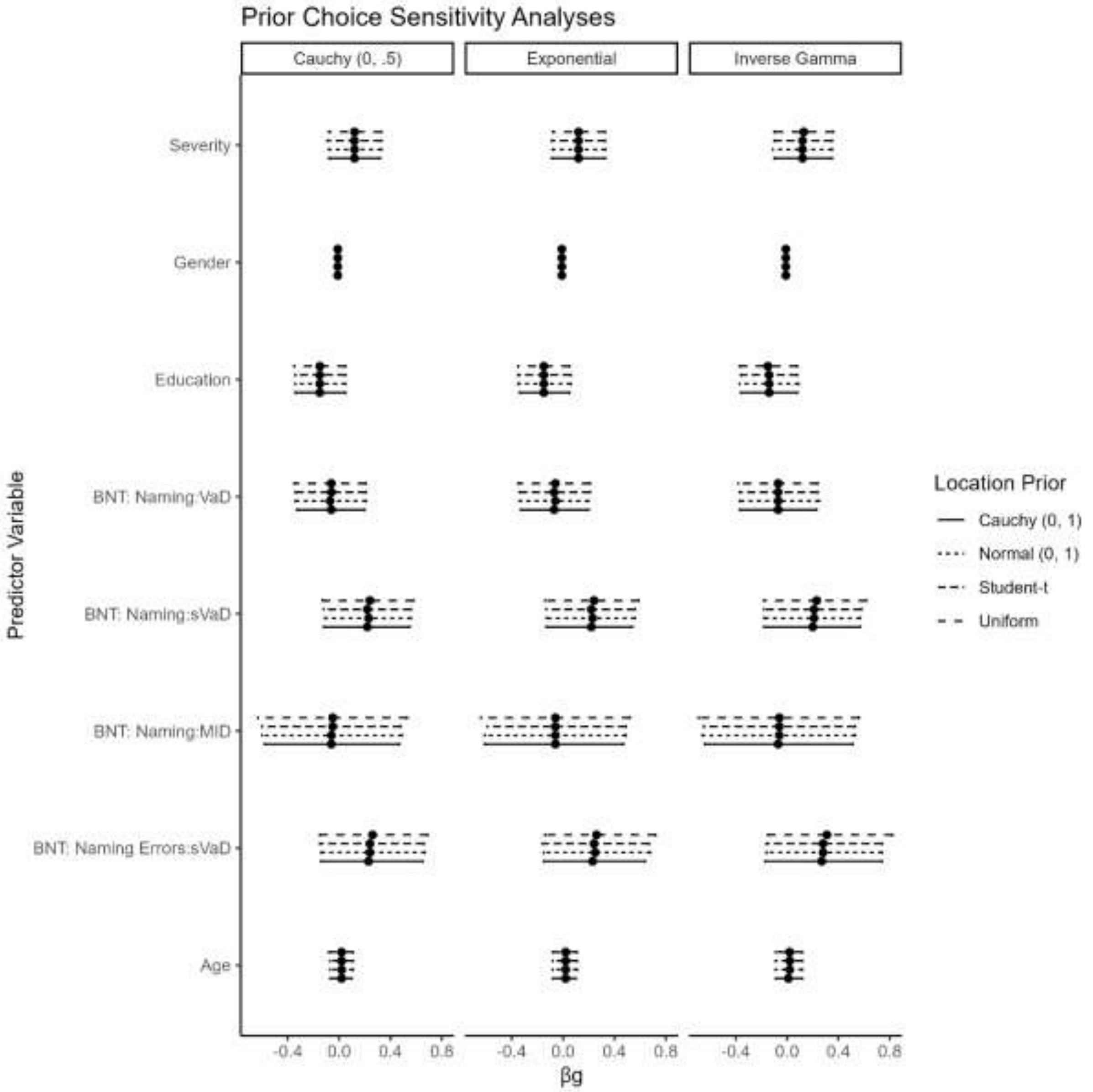

Figure 1. Regression coefficients with 95% confidence intervals for the Boston Naming Test (BNT) model. sVaD: subcortical vascular dementia, VaD: vascular dementia, MID: multi-infarct dementia, Severity: difference in dementia severity between dementia groups, Education: difference in average years of education between dementia groups, Age: difference in average age between dementia groups, Gender: difference in proportion of women between dementia groups.

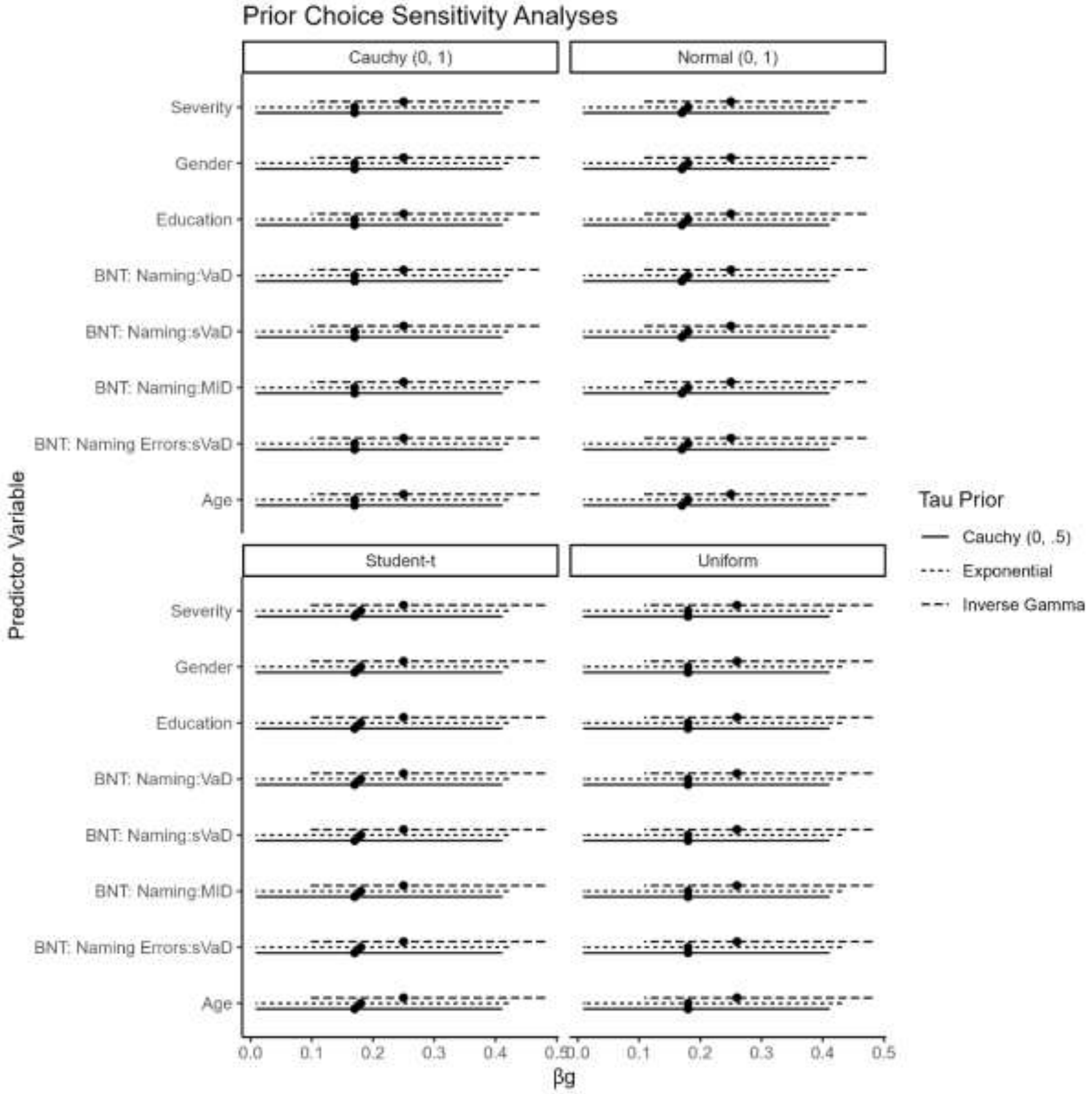

Figure 1. Study level standard deviation estimates with 95% confidence intervals for the Boston Naming Test (BNT) model. sVaD: subcortical vascular dementia, VaD: vascular dementia, MID: multi-infarct dementia, Severity: difference in dementia severity between dementia groups, Education: difference in average years of education between dementia groups, Age: difference in average age between dementia groups, Gender: difference in proportion of women between dementia groups.

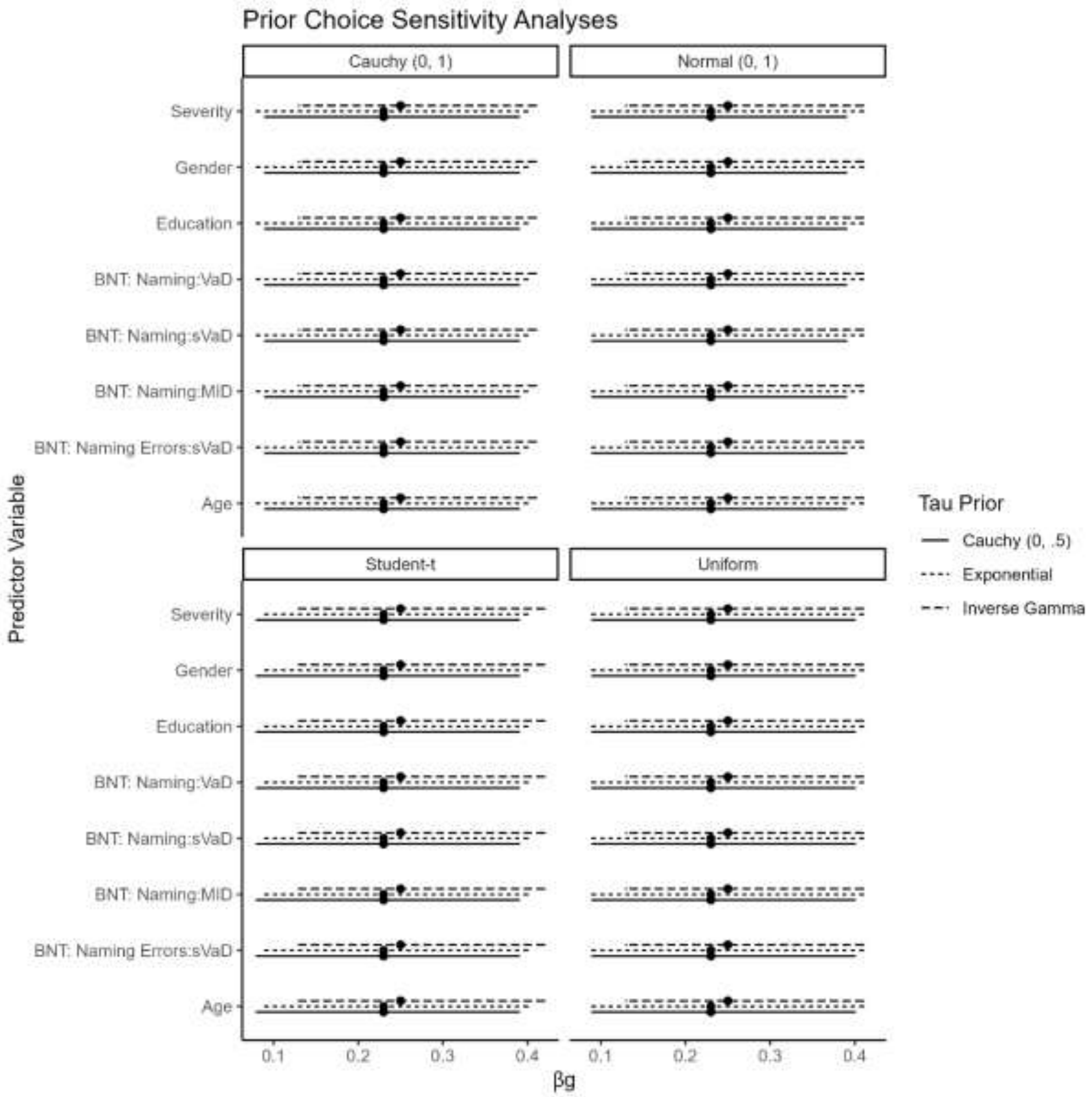

Figure 1. Effect size level standard deviation estimates with 95% confidence intervals for the Boston Naming Test (BNT) model. sVaD: subcortical vascular dementia, VaD: vascular dementia, MID: multi-infarct dementia, Severity: difference in dementia severity between dementia groups, Education: difference in average years of education between dementia groups, Age: difference in average age between dementia groups, Gender: difference in proportion of women between dementia groups.

Other Naming Measures

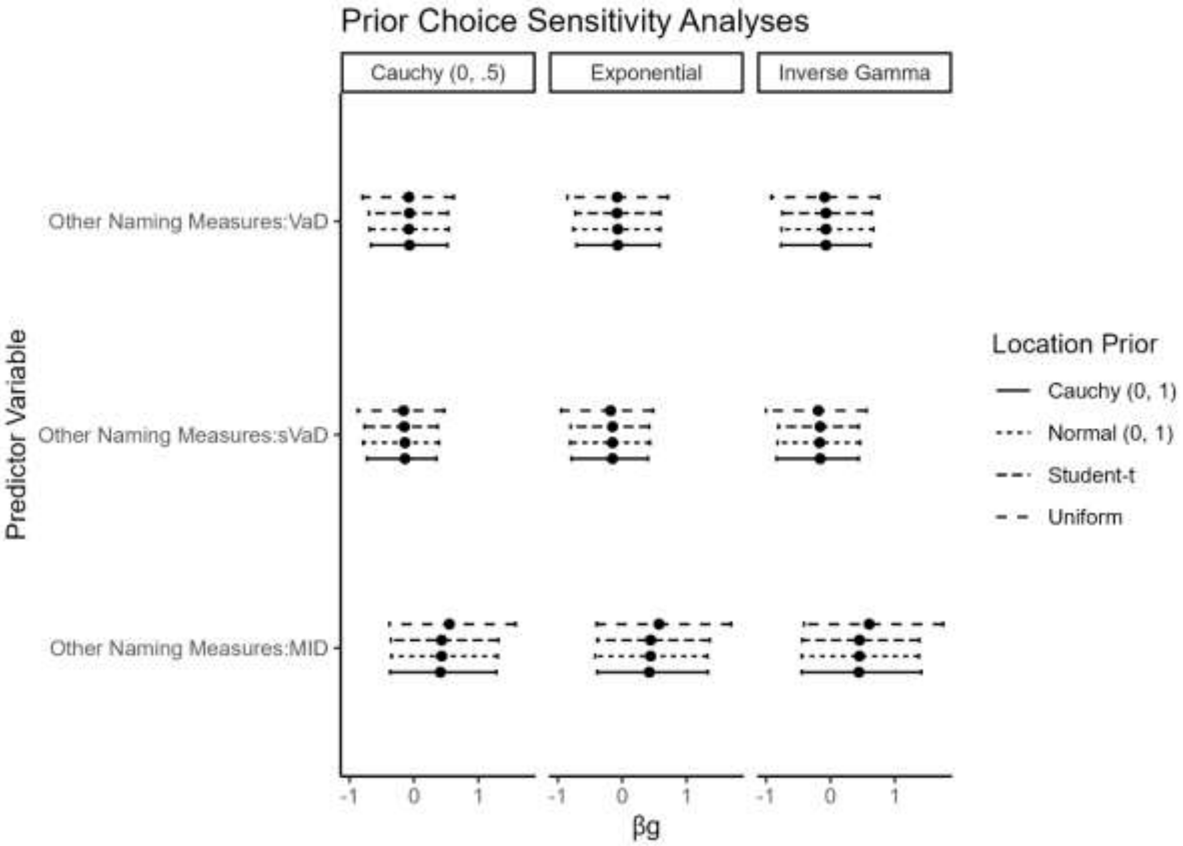

Figure 1. Regression coefficients with 95% confidence intervals for the Other Naming Measures model. For tests included in the analysis see Supplementary Materials 1. sVaD: subcortical vascular dementia, VaD: vascular dementia, MID: multi-infarct dementia.

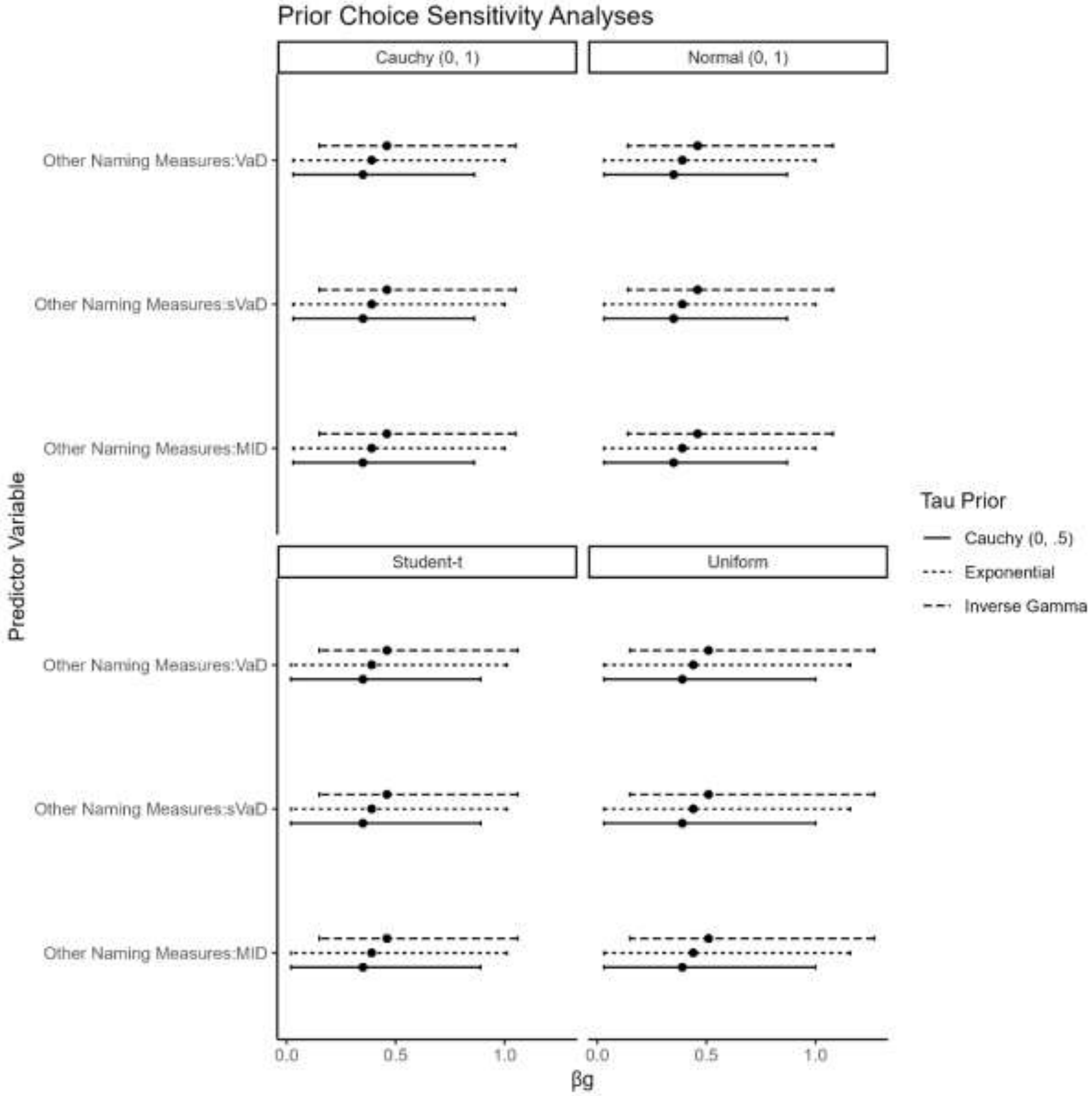

Figure 1. Study level standard deviation estimates with 95% confidence intervals for the Other Naming Measures model. For tests included in the analysis see Supplementary Materials 1. sVaD: subcortical vascular dementia, VaD: vascular dementia, MID: multi-infarct dementia.

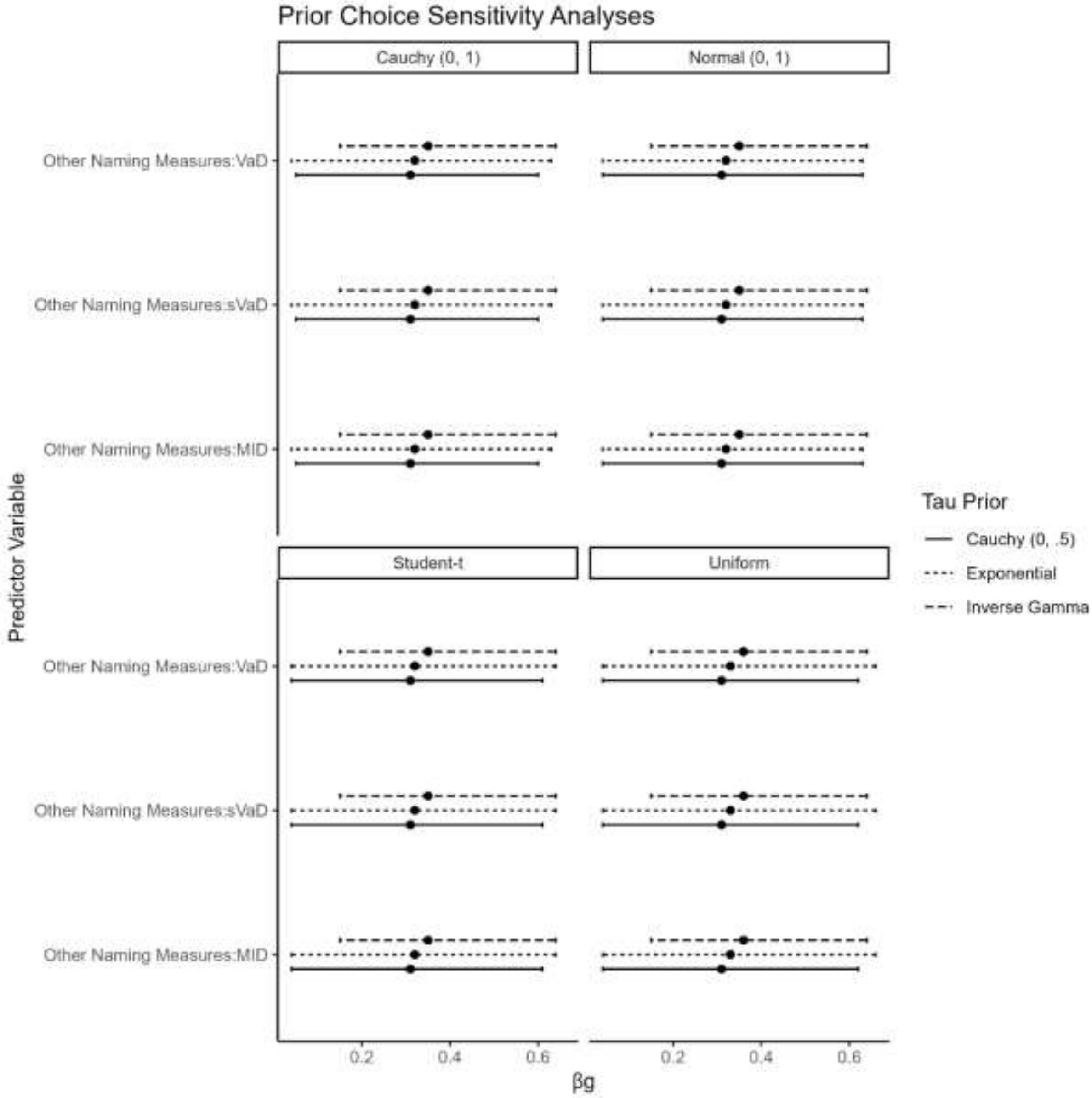

Figure 1. Effect size level standard deviation estimates with 95% confidence intervals for the Other Naming Measures model. For tests included in the analysis see Supplementary Materials 1. sVaD: subcortical vascular dementia, VaD: vascular dementia, MID: multi-infarct dementia.

Addenbrooke's Cognitive Examination

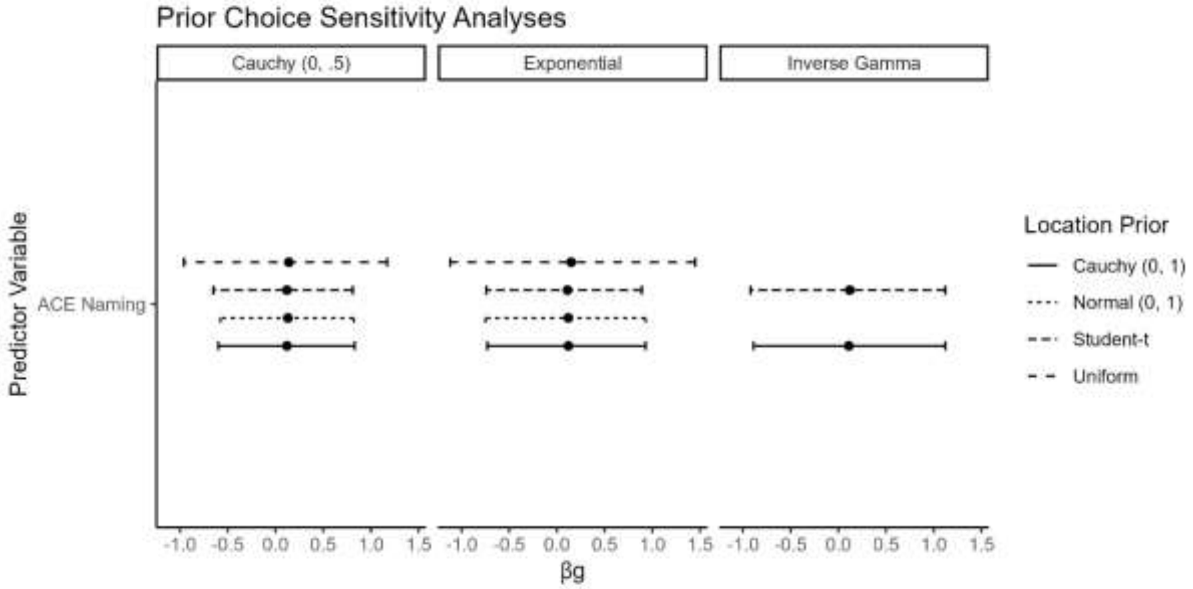

Figure 1. Regression coefficients with 95% confidence intervals for the Addenbrooke's Cognitive Examination: Naming model.

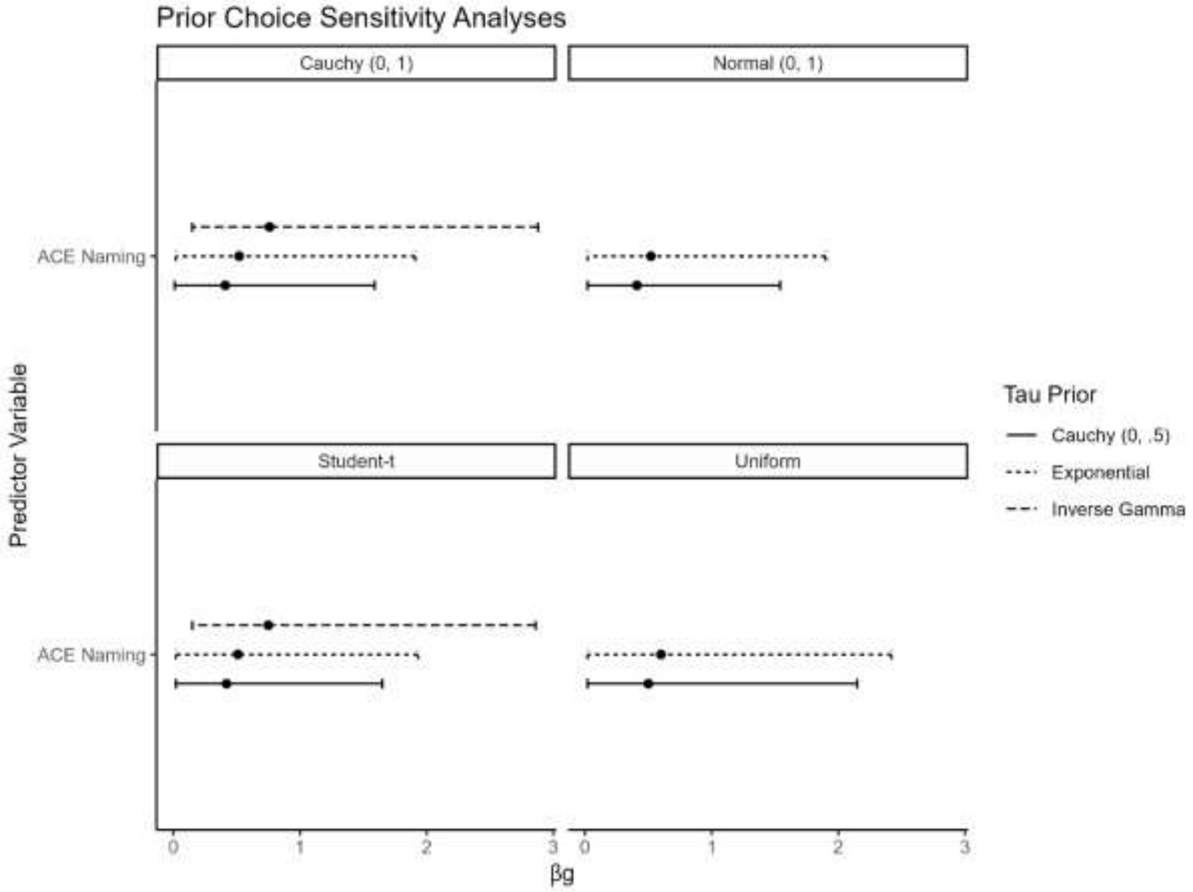

Figure 1. Study level standard deviation estimates with 95% confidence intervals for the Addenbrooke's Cognitive Examination: Naming model.

Writing

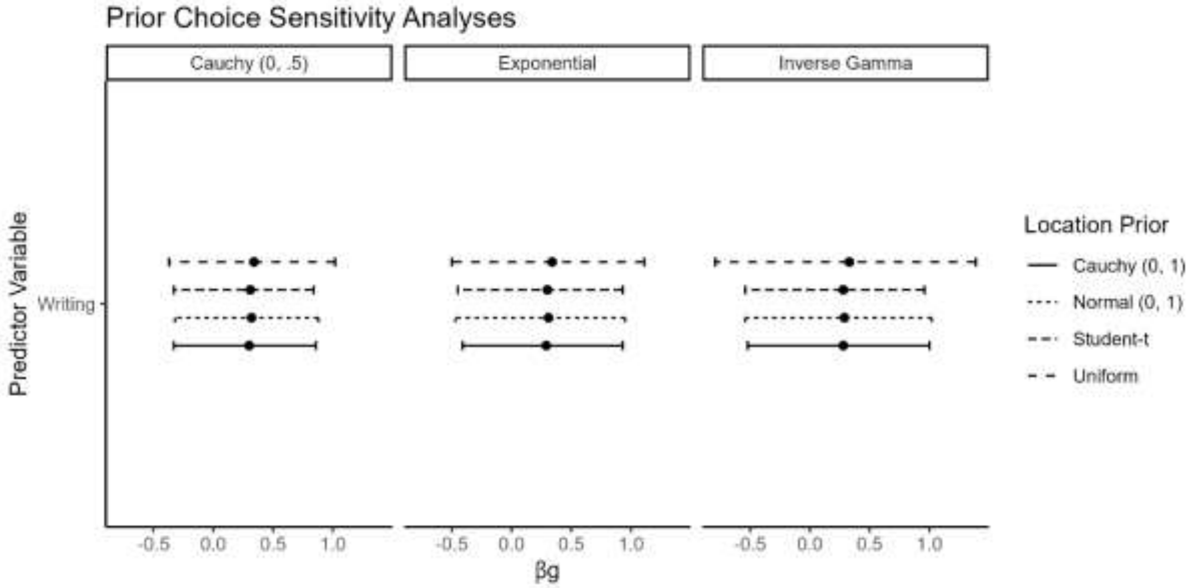

Figure 1. Regression coefficients with 95% confidence intervals for the measures of Writing model. For tests included in the analysis see Supplementary Materials 1.

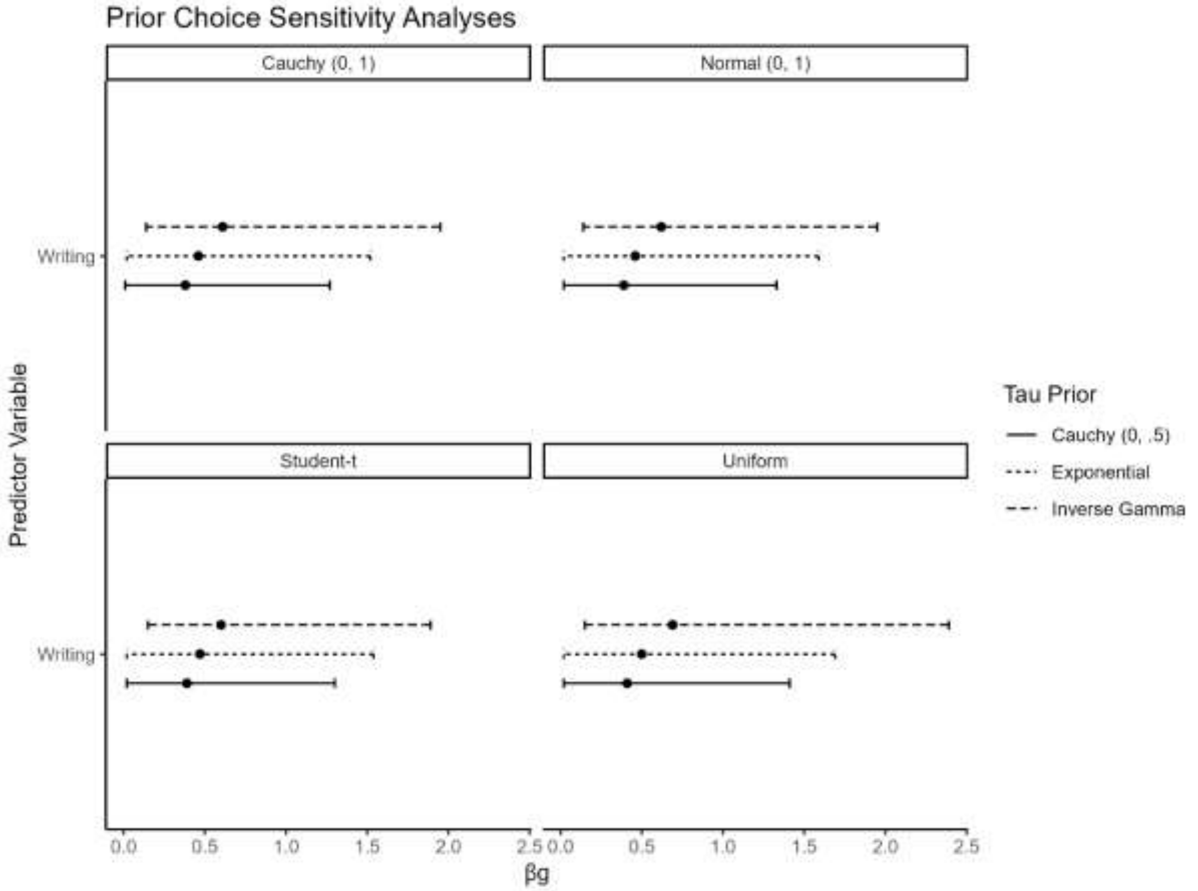

Figure 1. Study level standard deviation estimates with 95% confidence intervals for the measures of Writing model. For tests included in the analysis see Supplementary Materials 1.

Other Measures of Language Production

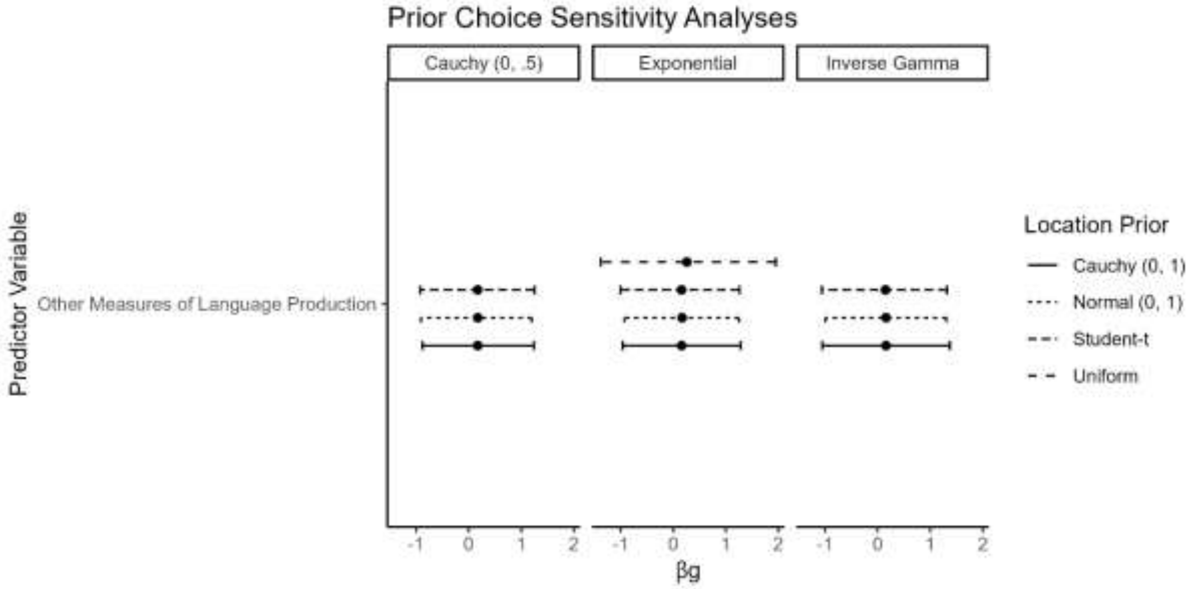

Figure 1. Regression coefficients with 95% confidence intervals for the Other Measures of Language Production model. For tests included see Supplementary Information 1.

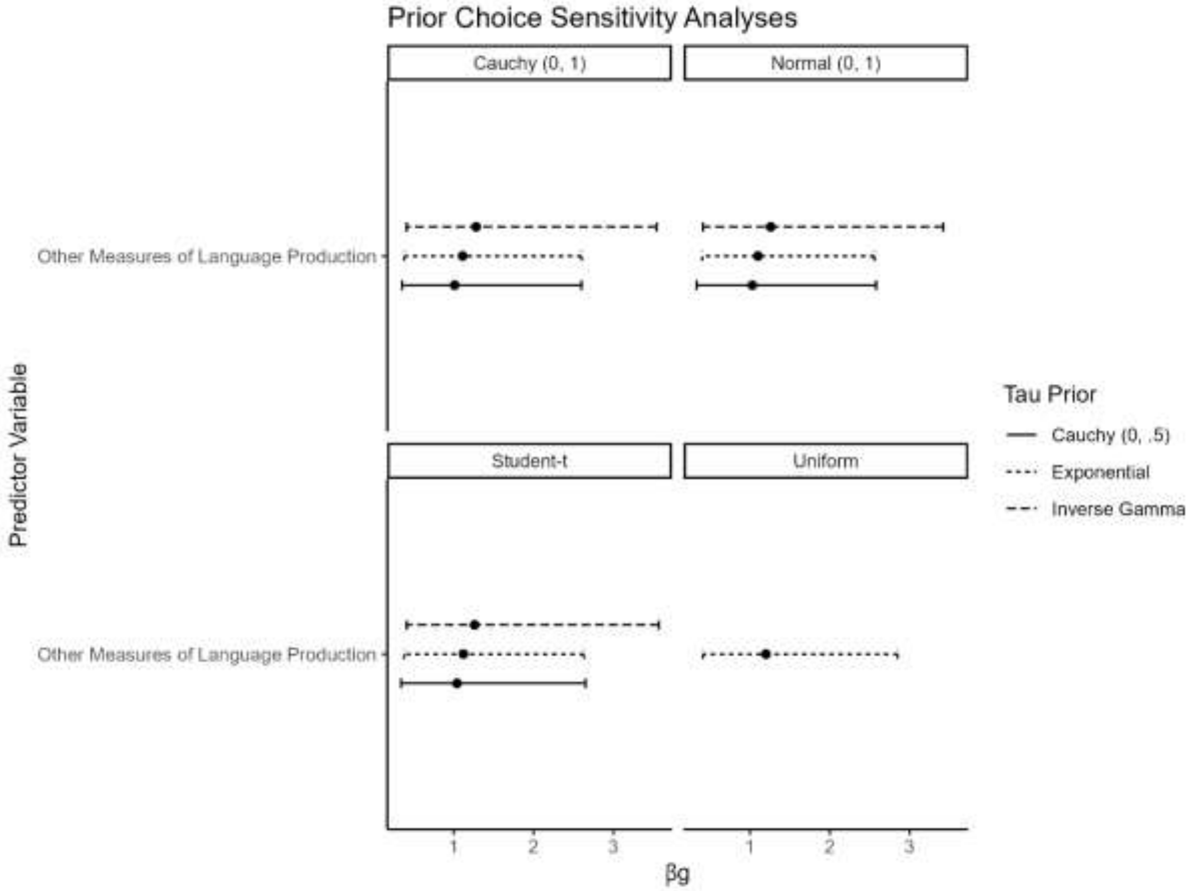

Figure 1. Study level standard deviation estimates with 95% confidence intervals for the Other Measures of Language Production model. For tests included see Supplementary Information 1.

Boston Naming Test and Phonemic Fluency: Quality Sensitivity Analysis

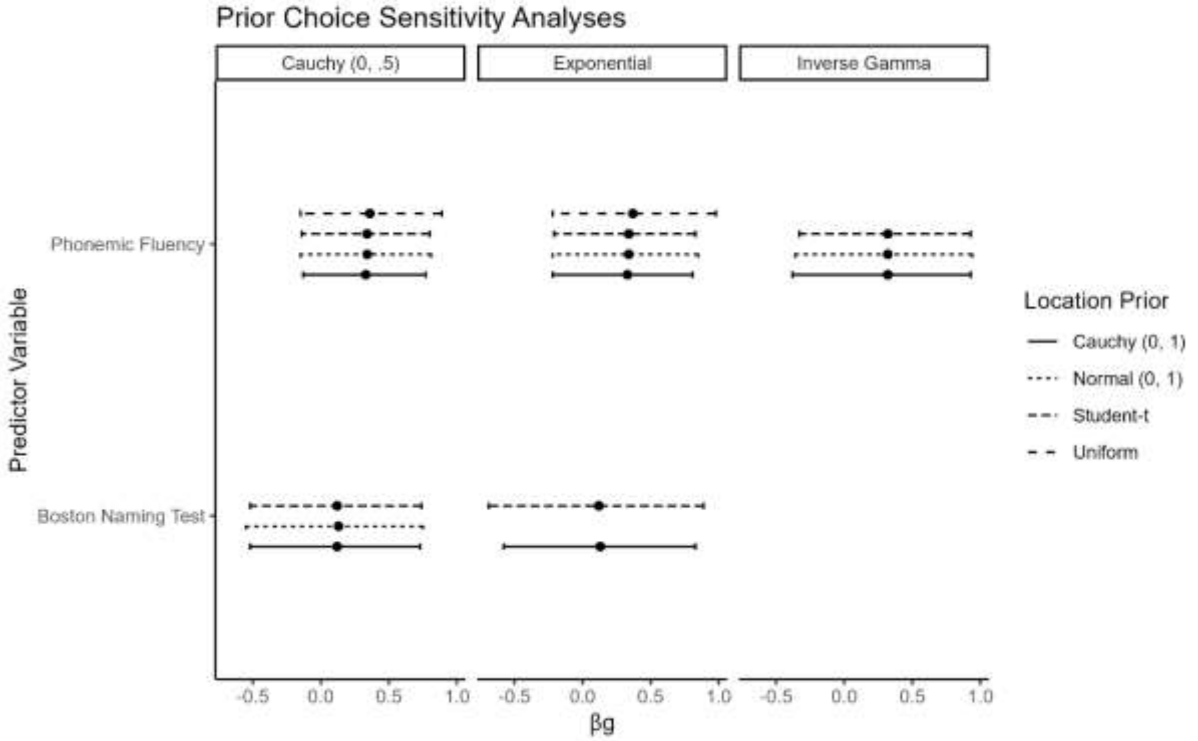

Figure 1. Regression coefficients with 95% confidence intervals for the study quality sensitivity analysis model of verbal Fluency and the Boston Naming Test. For included studies see Supplementary Materials 1.

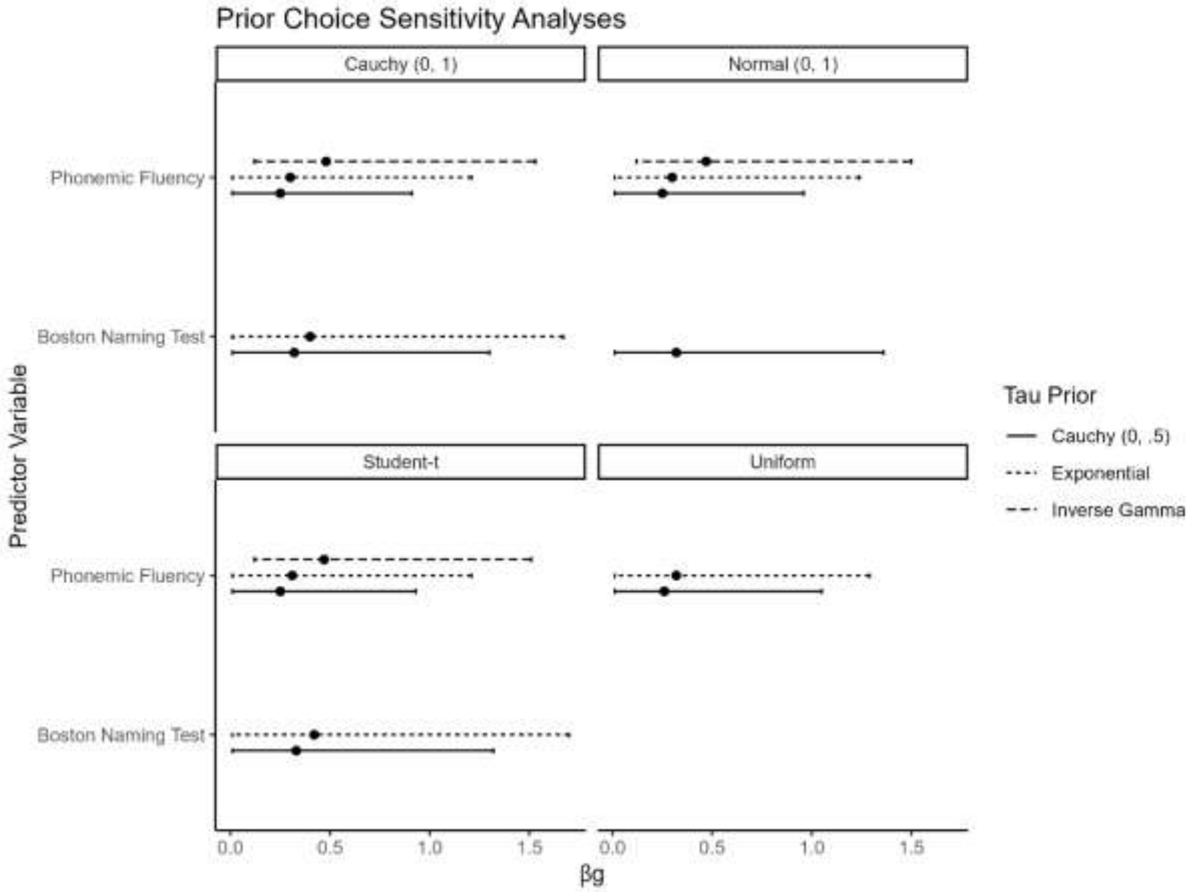

Figure 1. Study level standard deviation estimates with 95% confidence intervals for the study quality sensitivity analysis model of verbal Fluency and the Boston Naming Test. For included studies see Supplementary Materials 1.

Language Comprehension

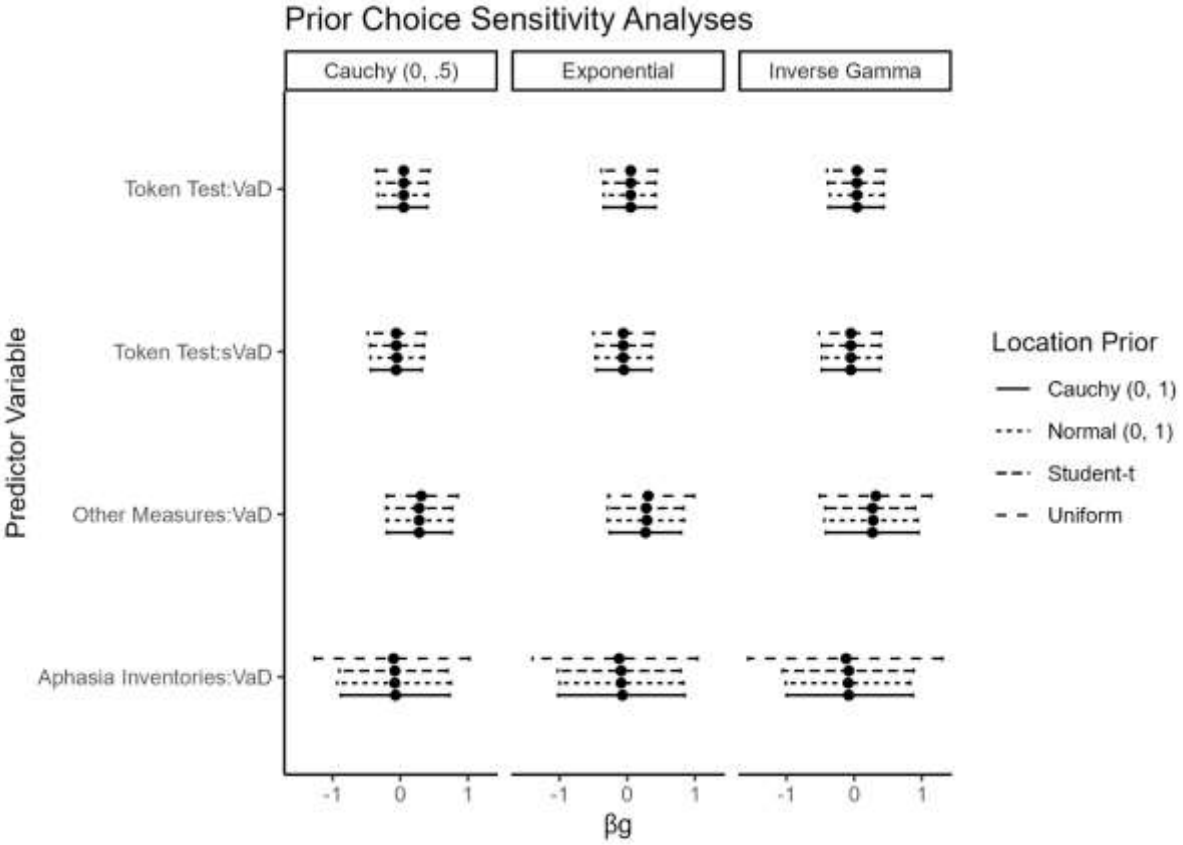

Figure 1. Regression coefficients with 95% confidence intervals for the models of language comprehension. For tests included in Other Measures of language comprehension and Aphasia Inventories see Supplementary Materials 1. sVaD: subcortical vascular dementia, VaD: vascular dementia.

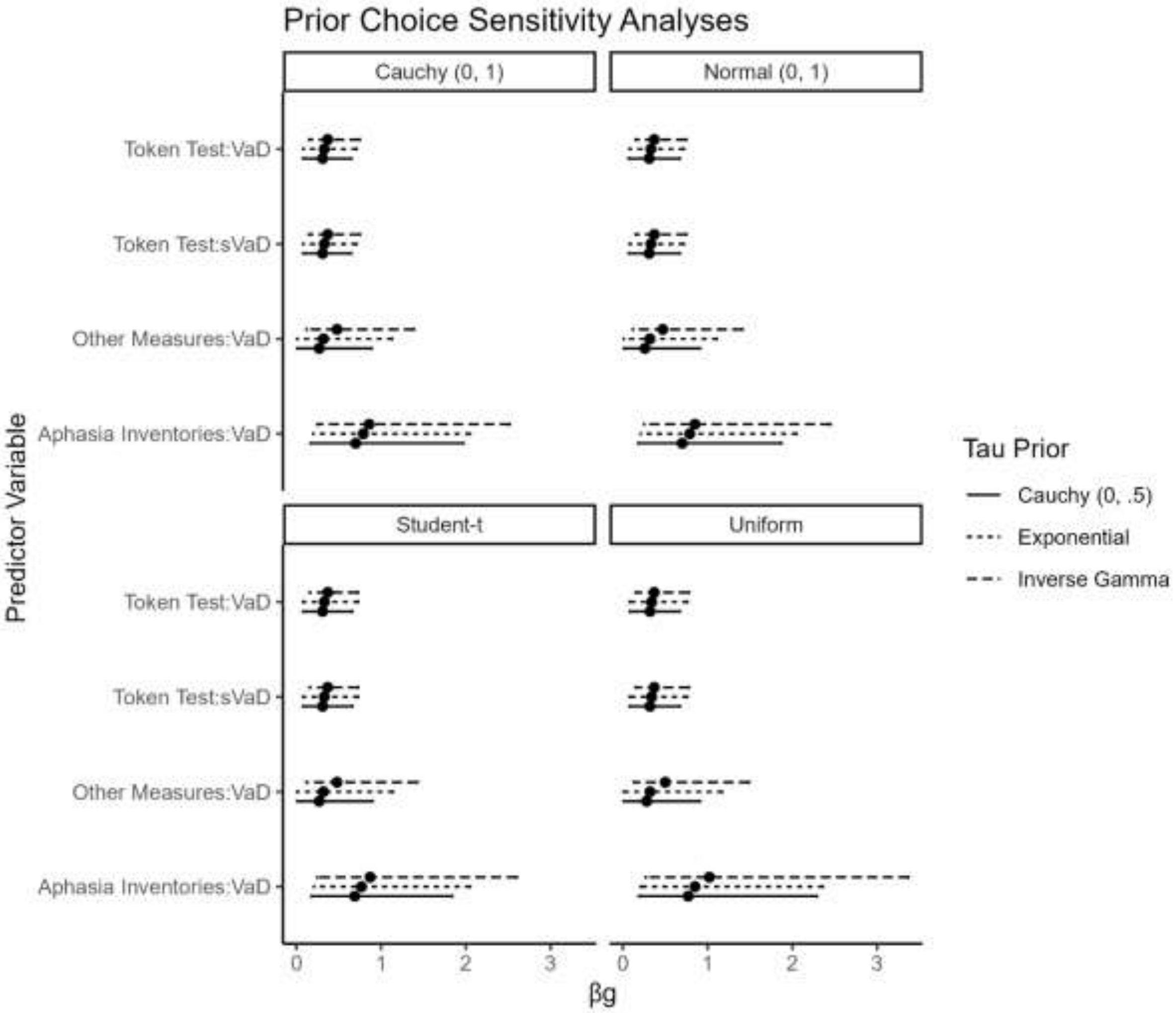

Figure 1. Study level standard deviation estimates with 95% confidence intervals for the models of language comprehension. For tests included in Other Measures of language comprehension and Aphasia Inventories see Supplementary Materials 1. sVaD: subcortical vascular dementia, VaD: vascular dementia.

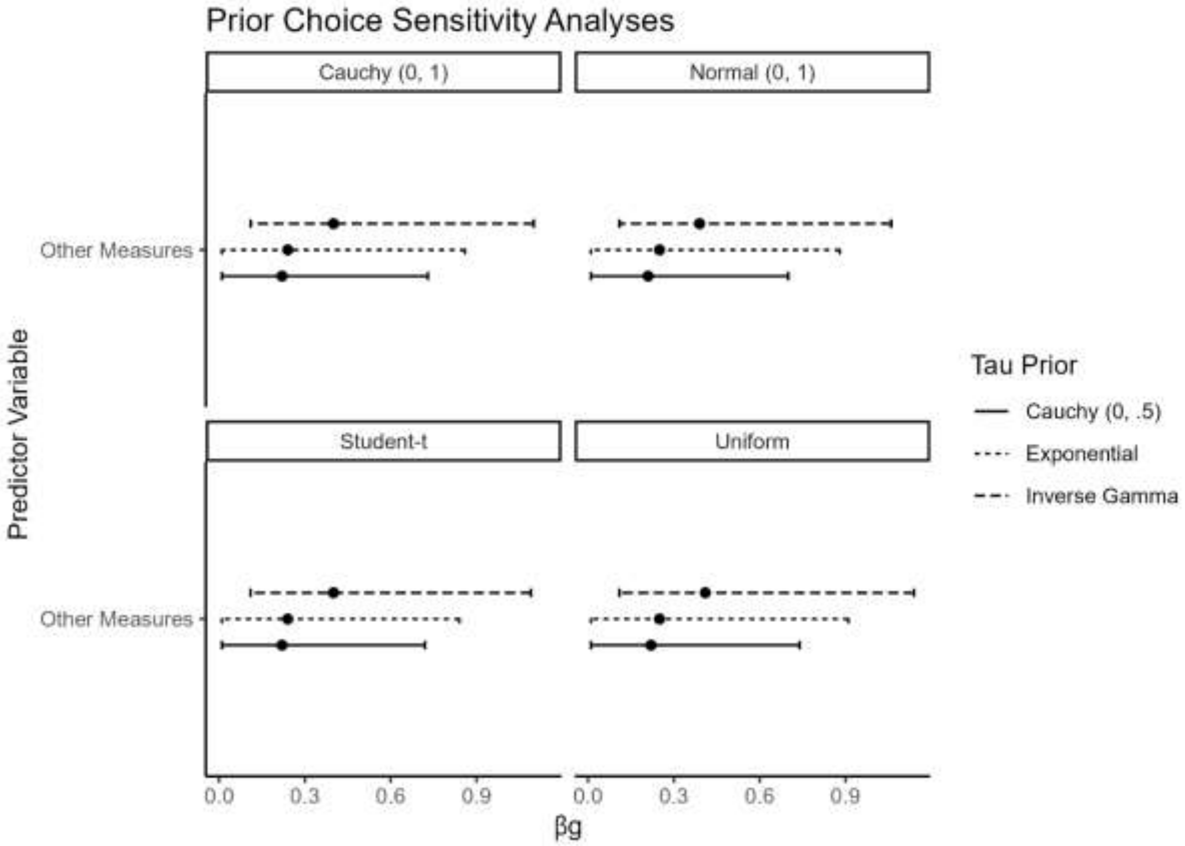

Figure 1. Effect size level standard deviation estimates with 95% confidence intervals for the models of language comprehension. For tests included in Other Measures of language comprehension and Aphasia Inventories see Supplementary Materials 1. sVaD: subcortical vascular dementia, VaD: vascular dementia.

Reading

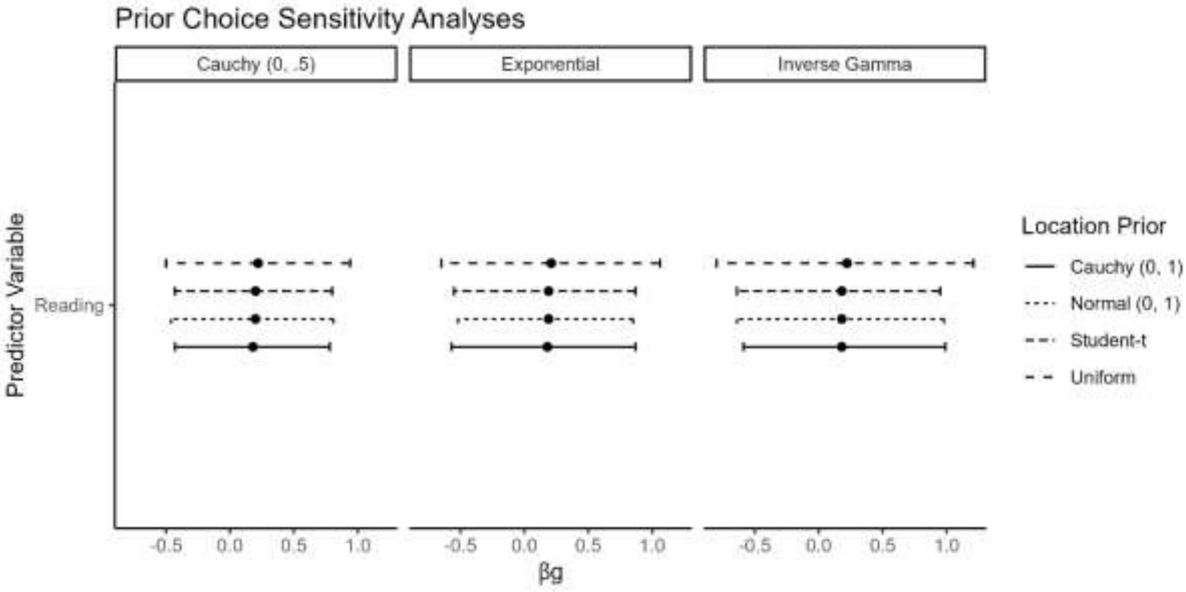

Figure 1. Regression coefficients with 95% confidence intervals for the measures of Reading model. For tests included see Supplementary Information 1.

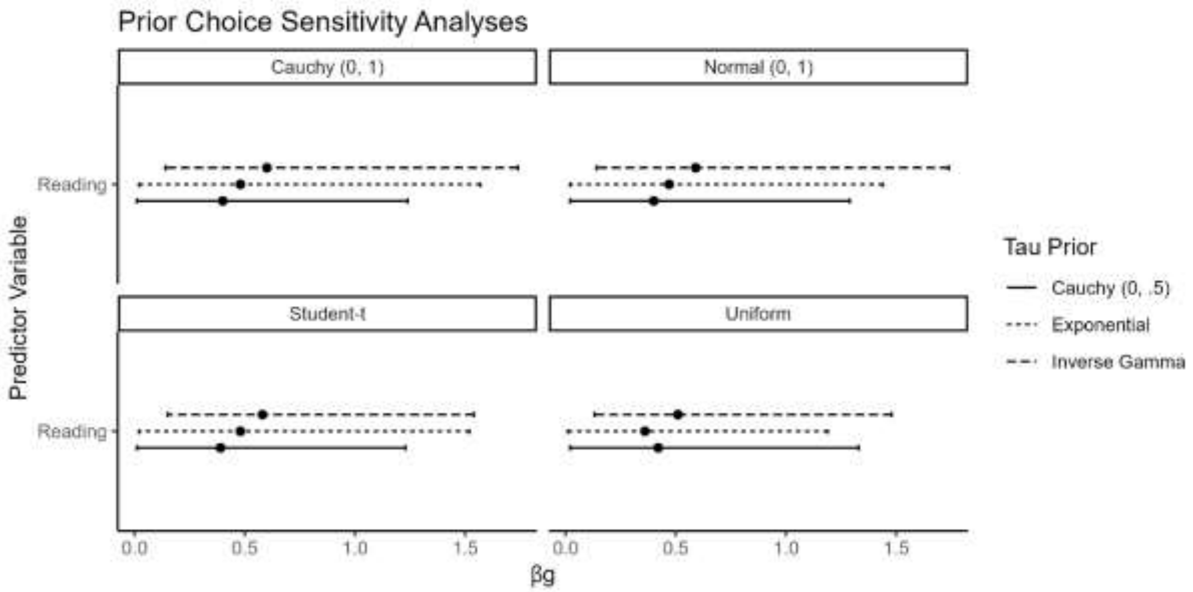

Figure 1. Study level standard deviation estimates with 95% confidence intervals for the measures of Reading model. For tests included see Supplementary Information 1.

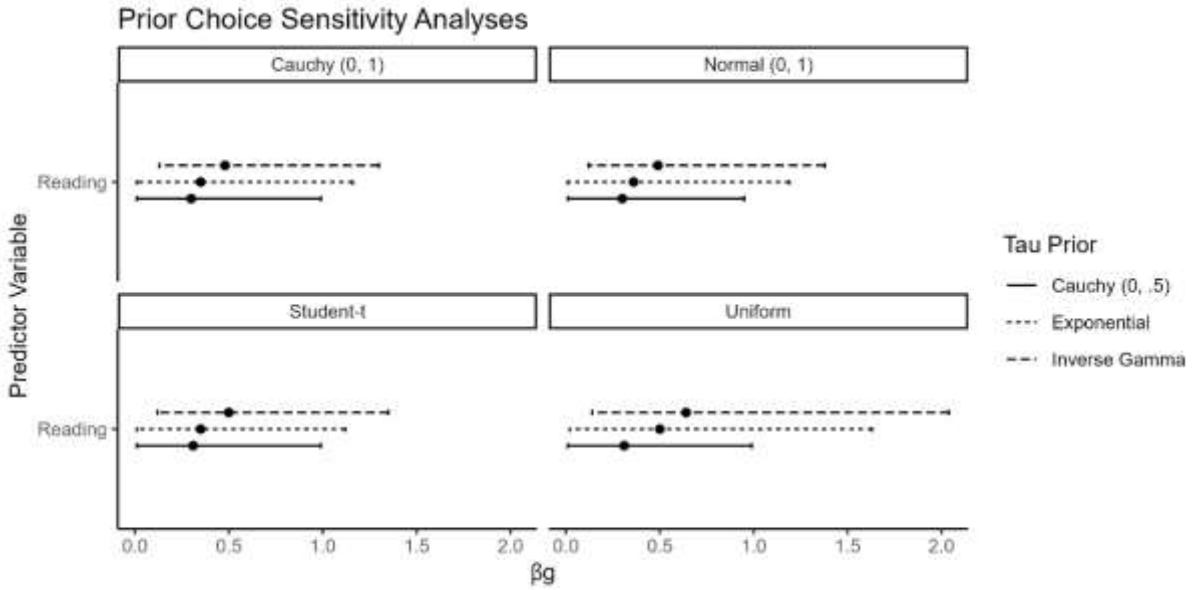

Figure 1. Effect size level standard deviation estimates with 95% confidence intervals for the measures of Reading model. For tests included see Supplementary Information 1.

Reasoning

Wechsler Adult Intelligence Scale

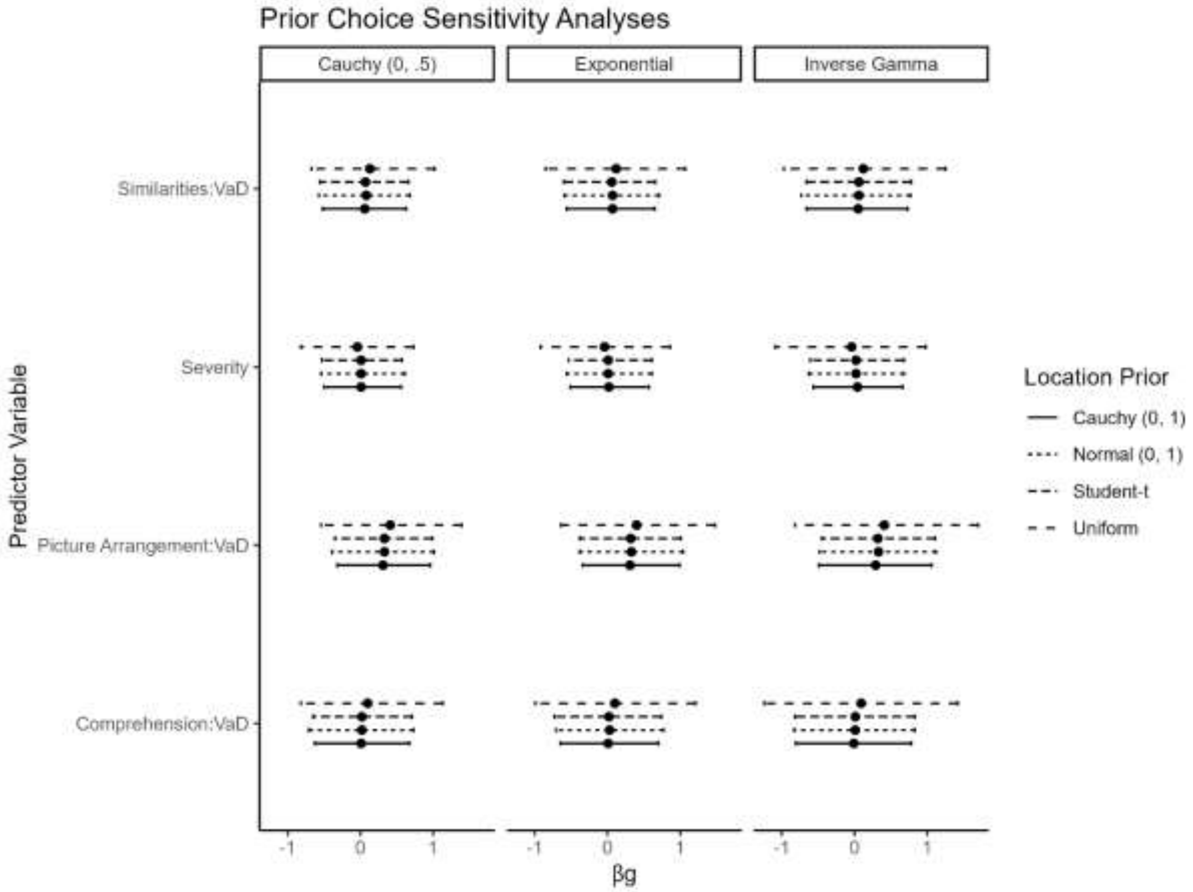

Figure 1. Regression coefficients with 95% confidence intervals for the Wechsler Adult Intelligence Scale (WAIS): Reasoning Subtests model. VaD: vascular dementia, Severity: difference in dementia severity between dementia groups.

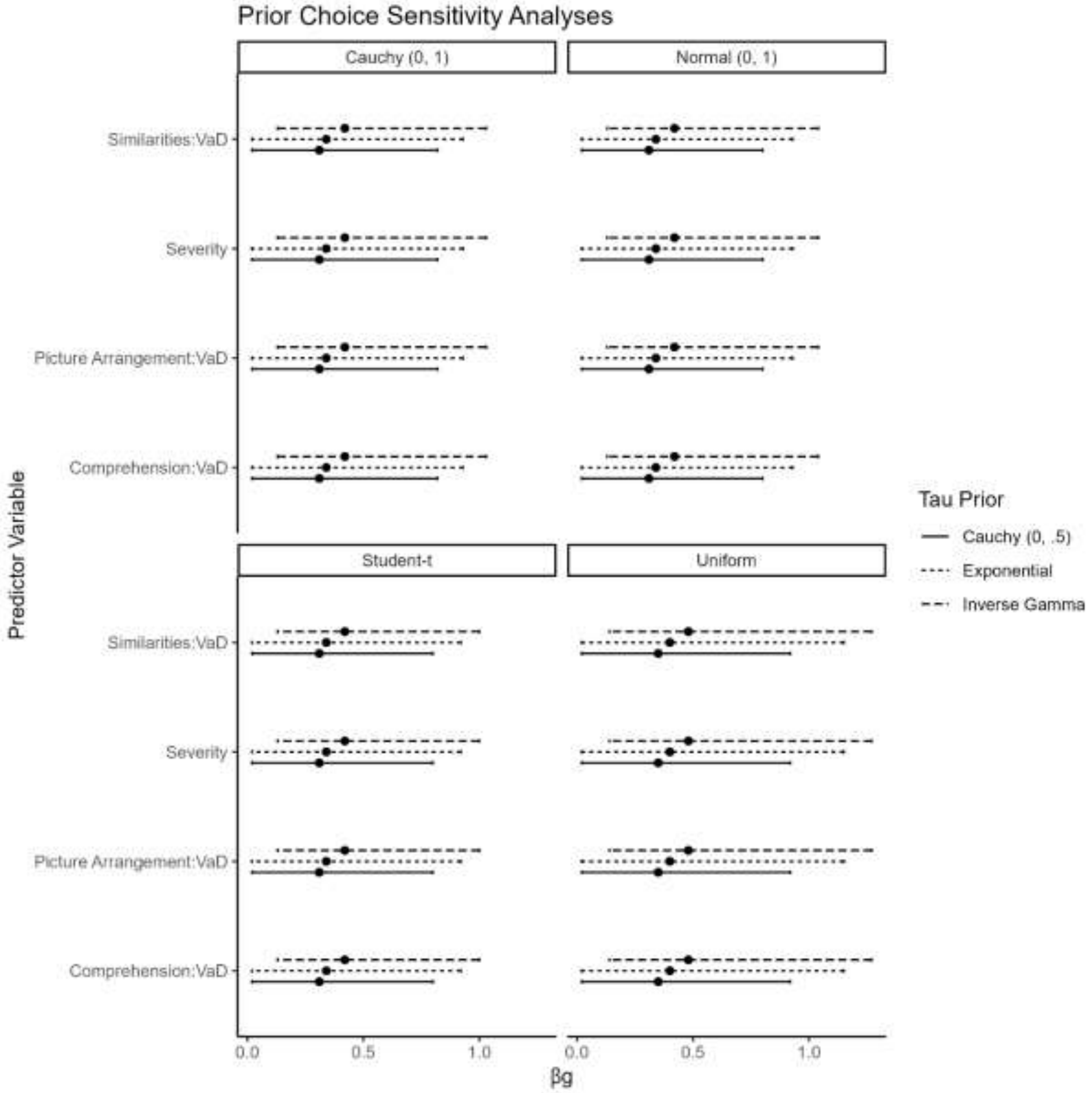

Figure 1. Study level standard deviation estimates with 95% confidence intervals for the Wechsler Adult Intelligence Scale (WAIS): Reasoning Subtests model. VaD: vascular dementia, Severity: difference in dementia severity between dementia groups.

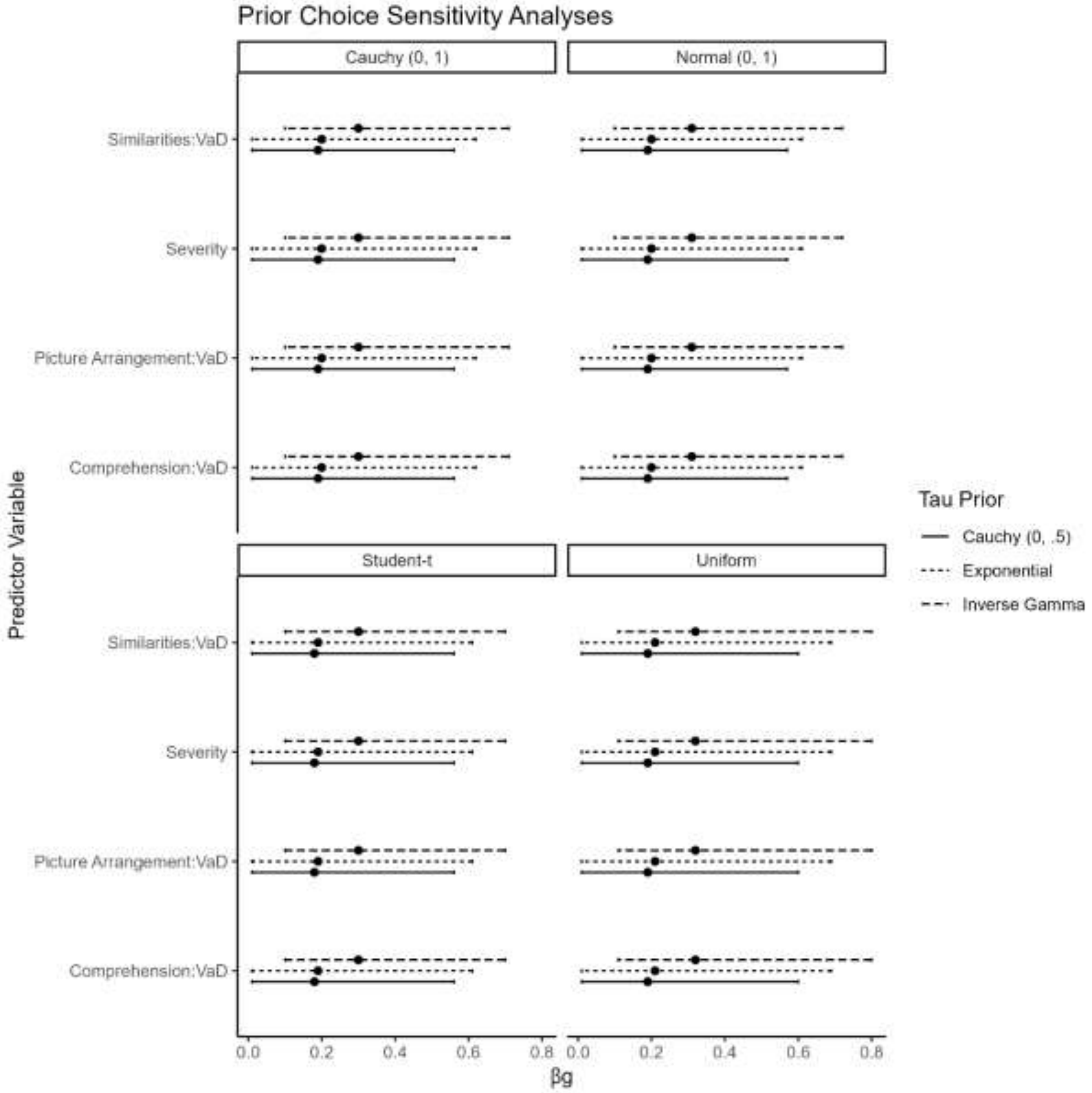

Figure 1. Effect size level standard deviation estimates with 95% confidence intervals for the Wechsler Adult Intelligence Scale (WAIS): Reasoning Subtests model. VaD: vascular dementia, Severity: difference in dementia severity between dementia groups.

Wisconsin Card Sorting Test

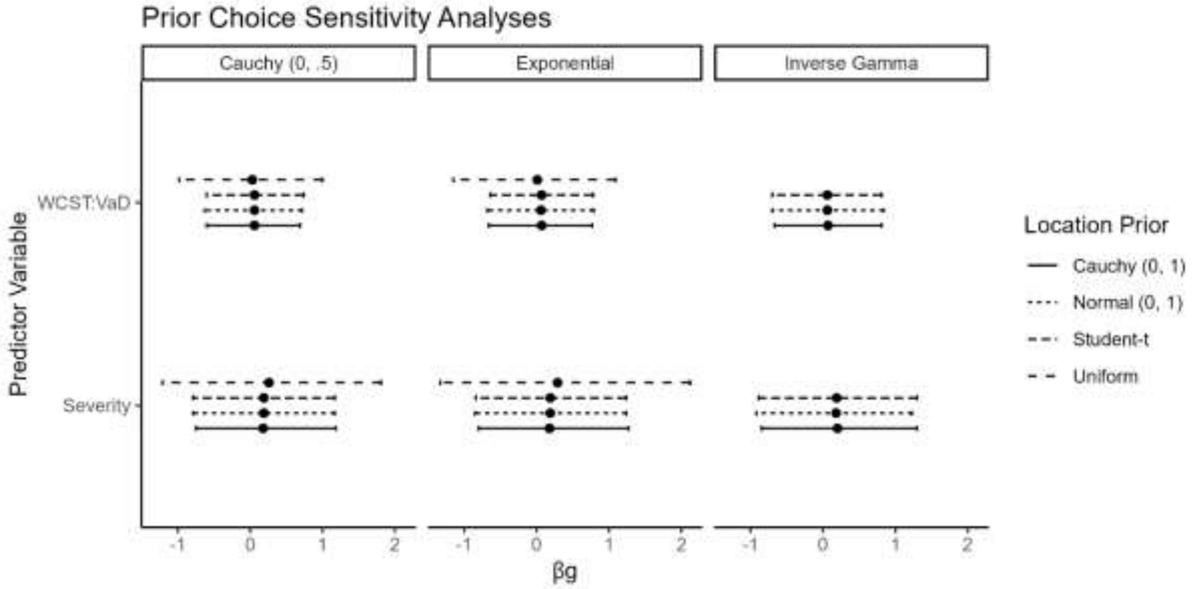

Figure 1. Regression coefficients with 95% confidence intervals for the Wisconsin Card Sorting Test model. VaD: vascular dementia, Severity: difference in dementia severity between dementia groups.

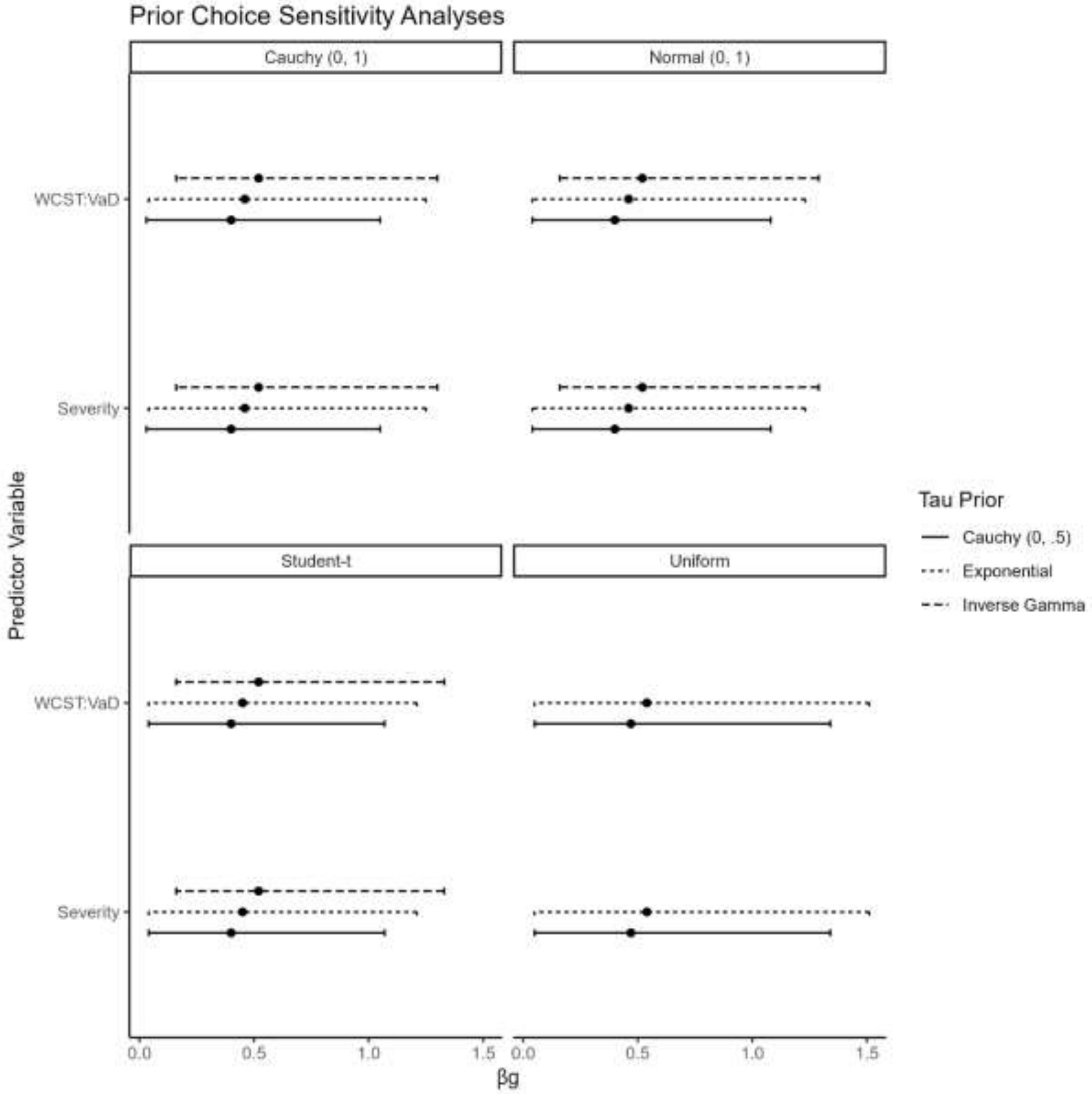

Figure 1. Study level standard deviation estimates with 95% confidence intervals for the Wisconsin Card Sorting Test model. VaD: vascular dementia, Severity: difference in dementia severity between dementia groups.

Raven’s Progressive Matrices

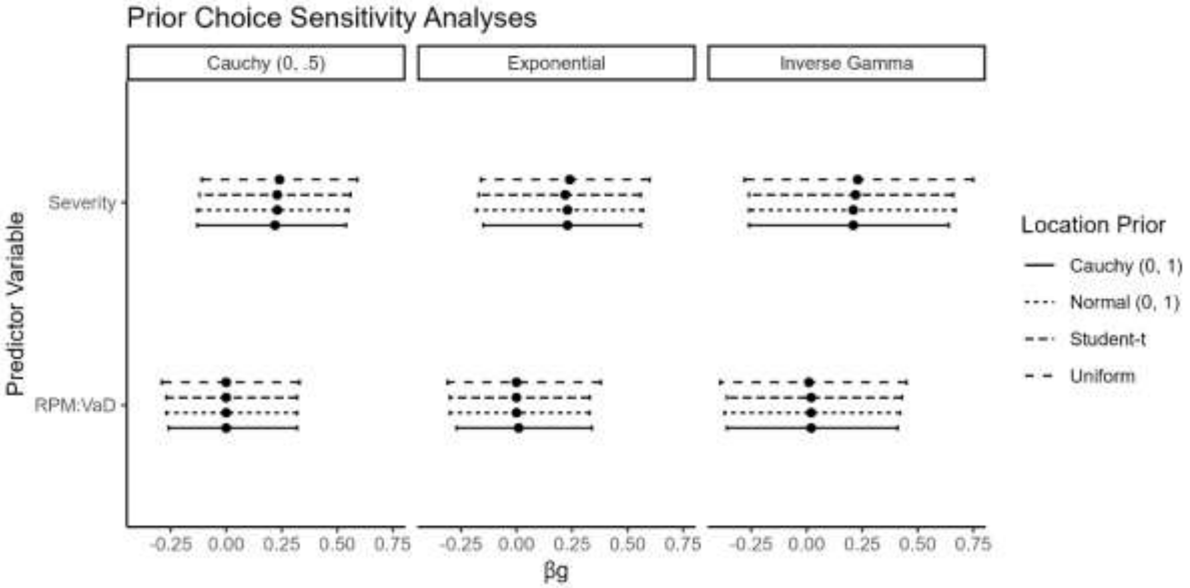

Figure 1. Regression coefficients with 95% confidence intervals for the Raven’s Progressive Matrices model. VaD: vascular dementia, Severity: difference in dementia severity between dementia groups.

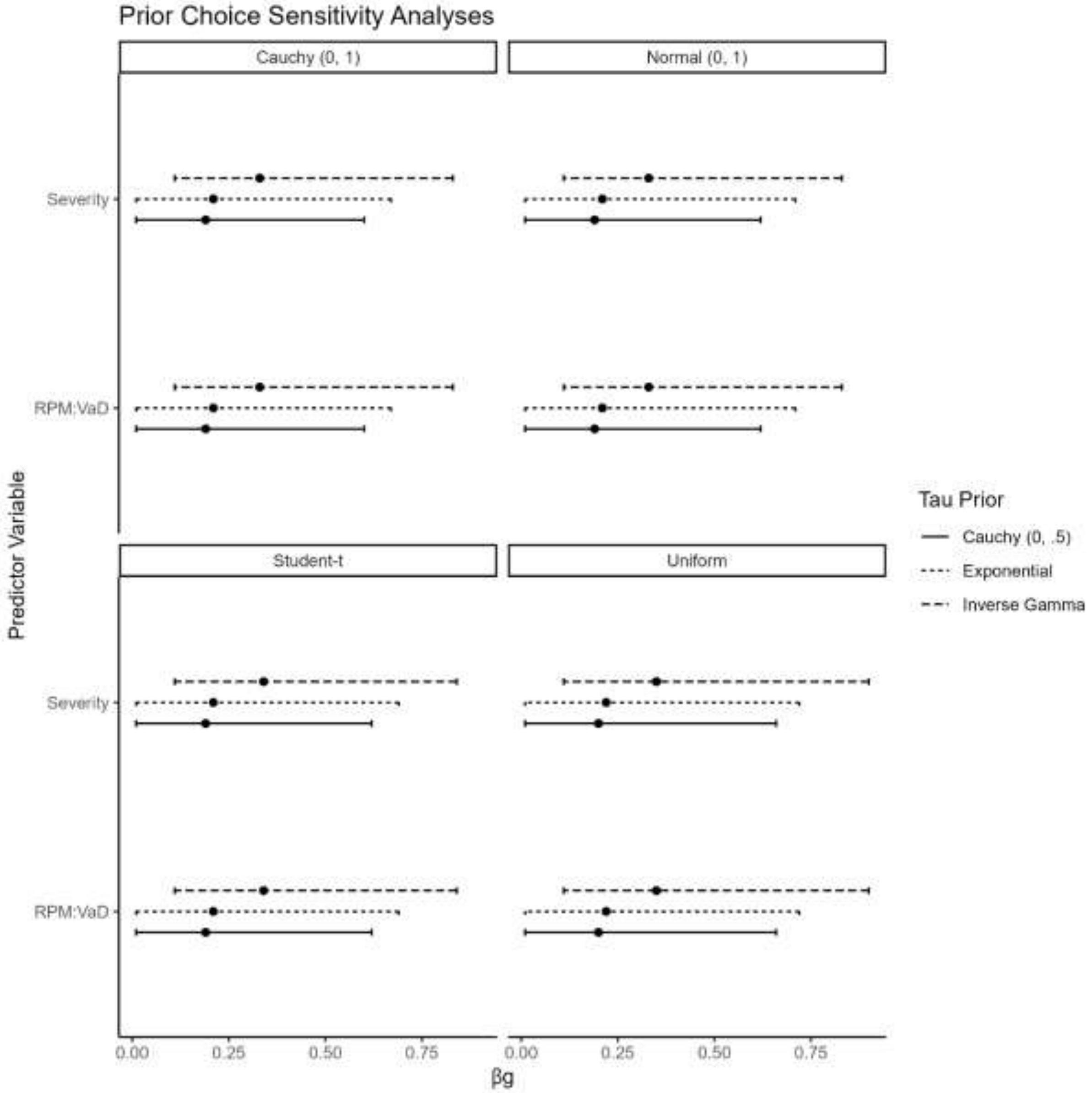

Figure 1. Study level standard deviation estimates with 95% confidence intervals for the Raven's Progressive Matrices model. VaD: vascular dementia, Severity: difference in dementia severity between dementia groups.

Raven's Coloured Progressive Matrices

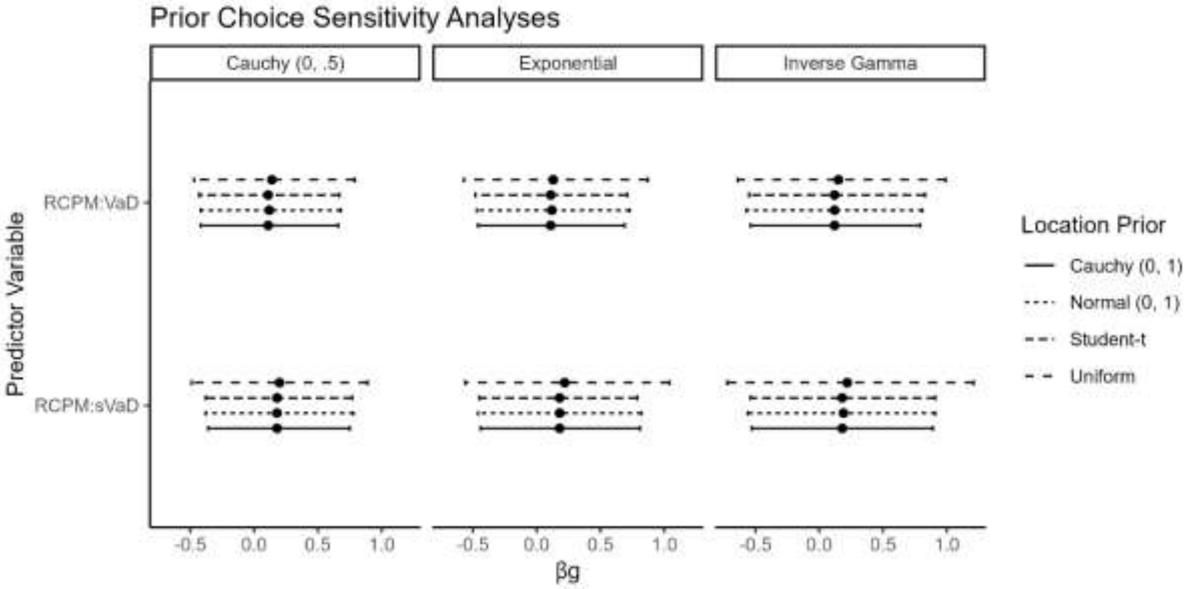

Figure 1. Regression coefficients with 95% confidence intervals for the Raven's Coloured Progressive Matrices model. sVaD: subcortical vascular dementia, VaD: vascular dementia.

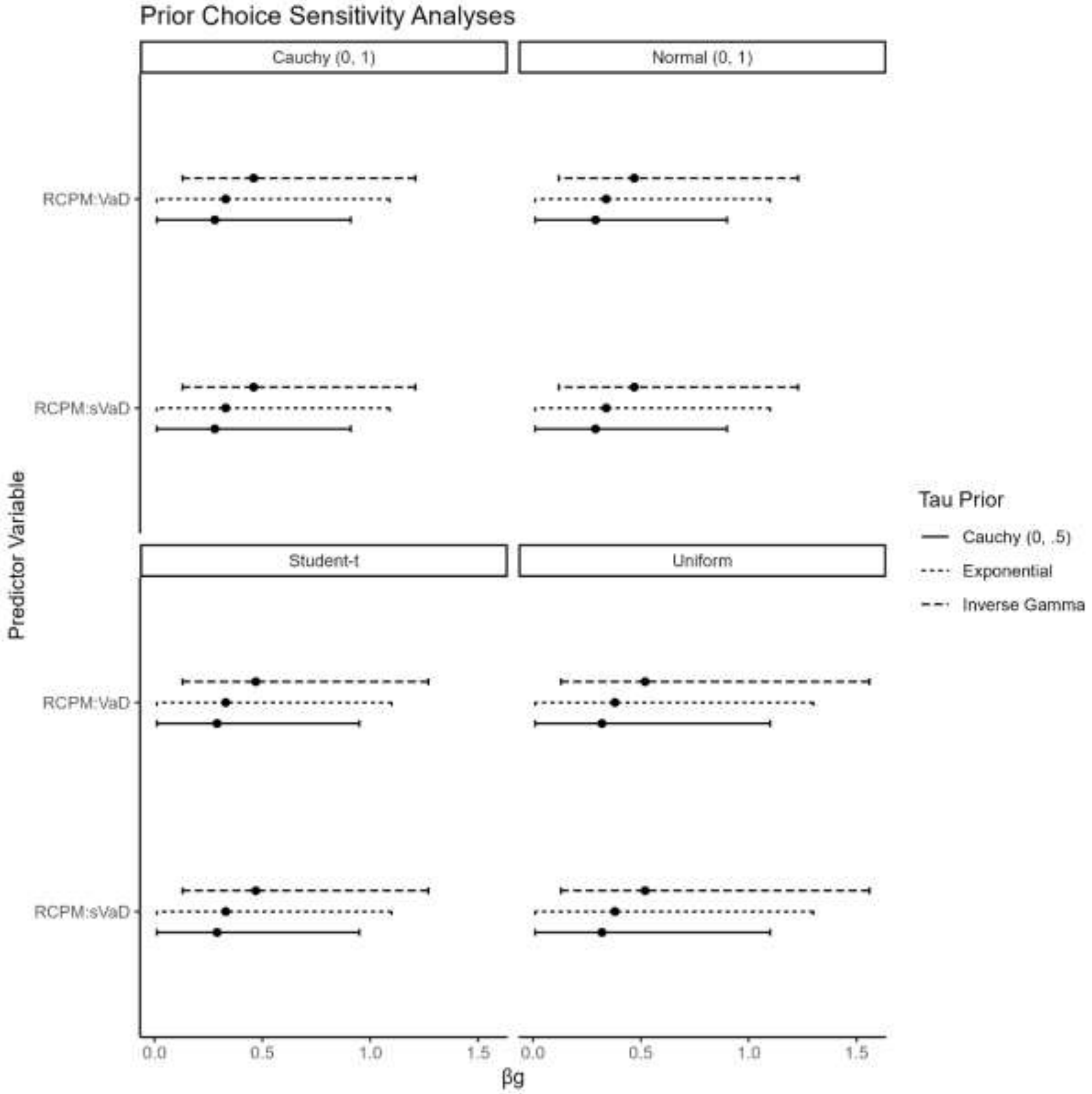

Figure 1. Study level standard deviation estimates with 95% confidence intervals for the Raven’s Coloured Progressive Matrices model. sVaD: subcortical vascular dementia, VaD: vascular dementia.

Attentional Matrices

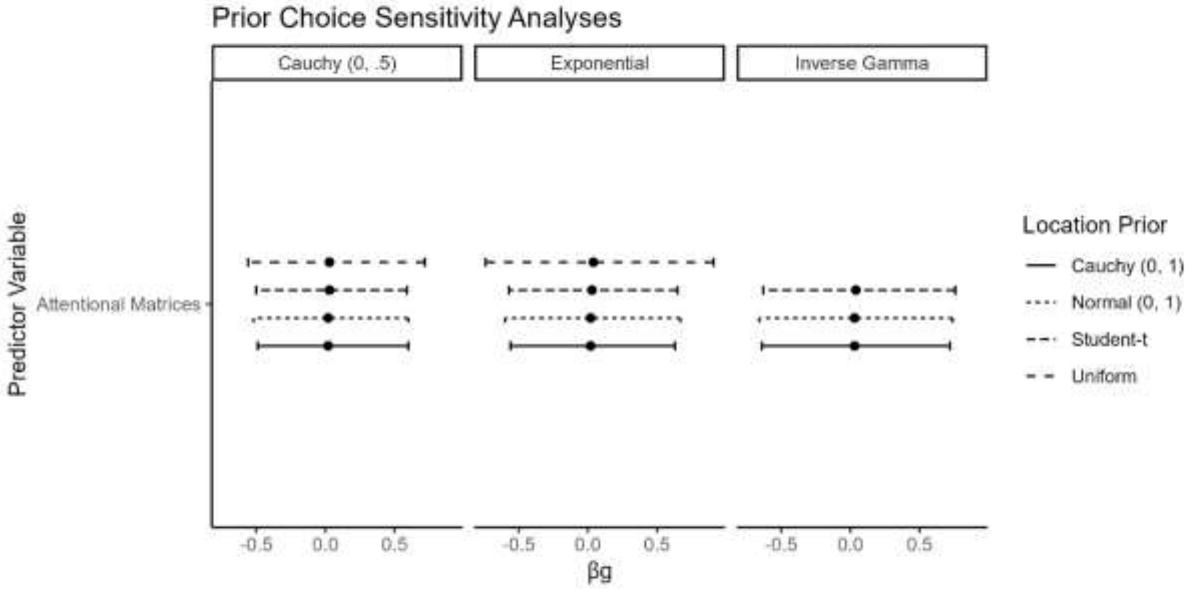

Figure 1. Regression coefficients with 95% confidence intervals for the Attentional Matrices model.

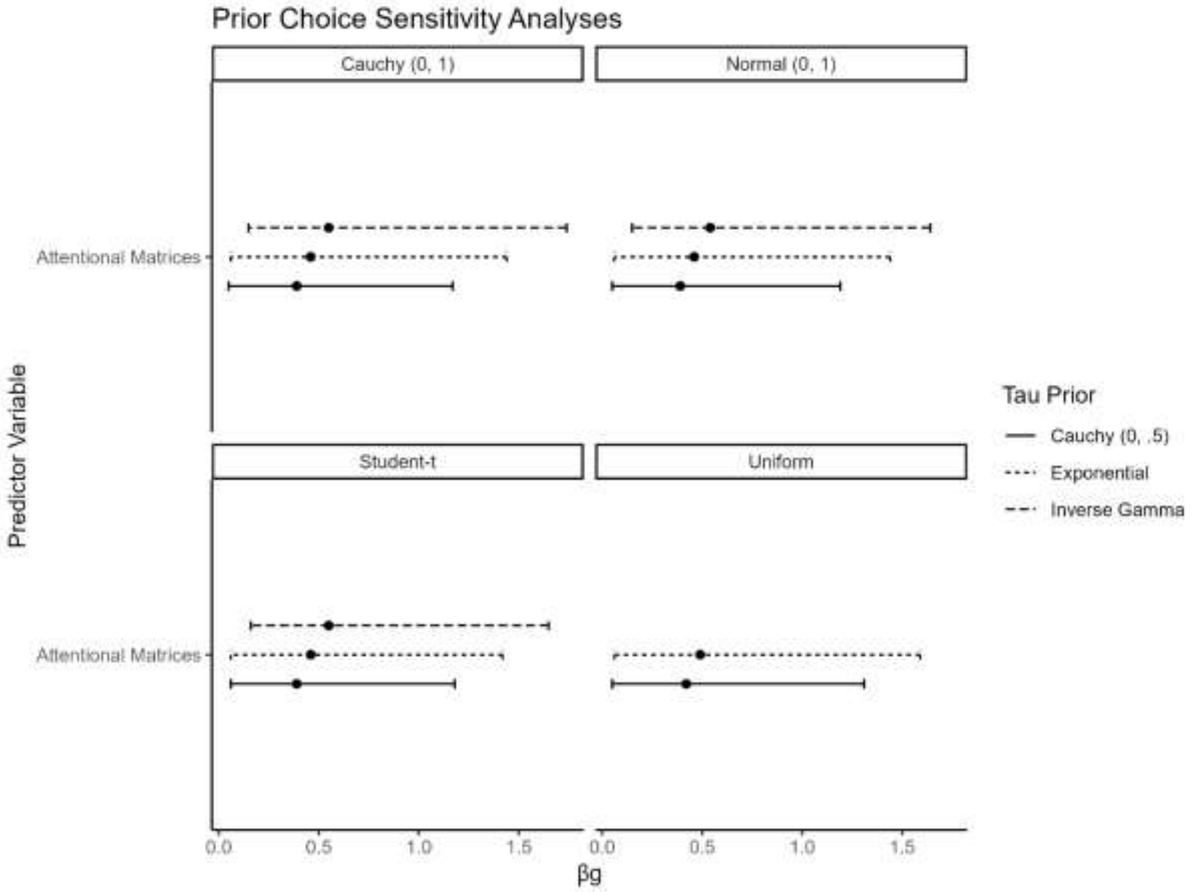

Figure 1. Study level standard deviation estimates with 95% confidence intervals for the Attentional Matrices model.

Frontal Assessment Battery: Abstraction

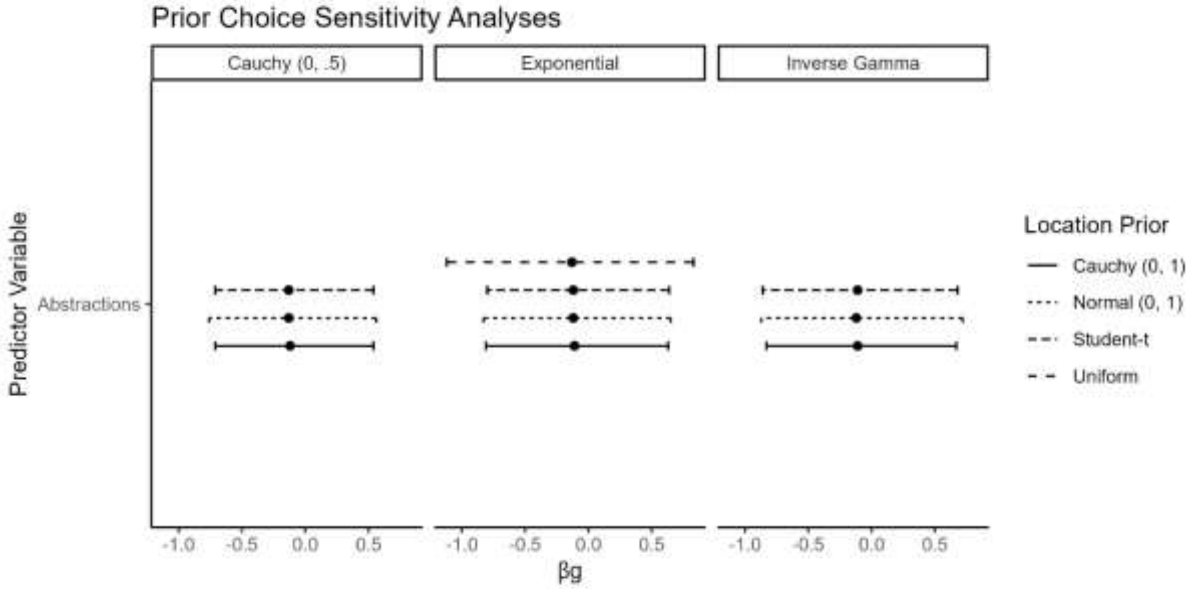

Figure 1. Regression coefficients with 95% confidence intervals for the Frontal Assessment Battery: Abstractions model.

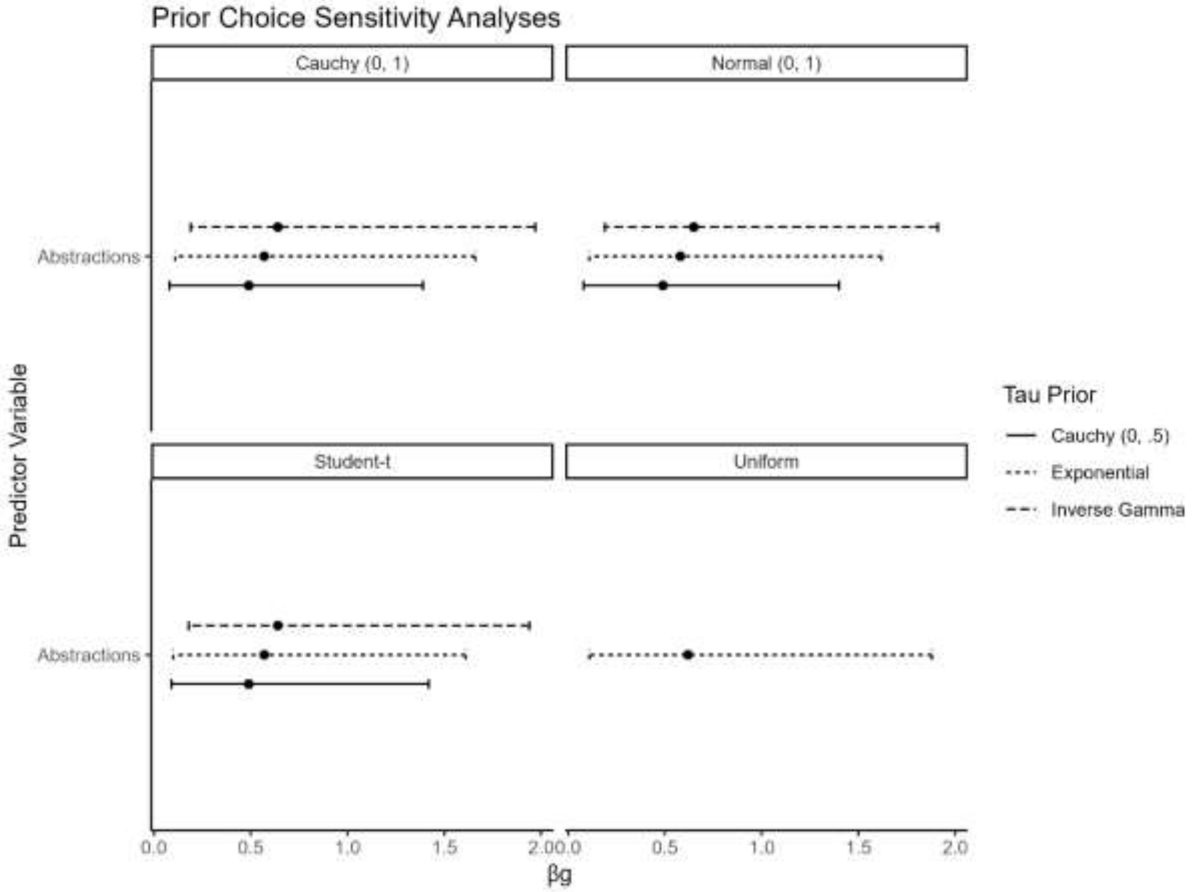

Figure 1. Study level standard deviation estimates with 95% confidence intervals for the Frontal Assessment Battery: Abstractions model.

Other Measures of Reasoning and Abstraction

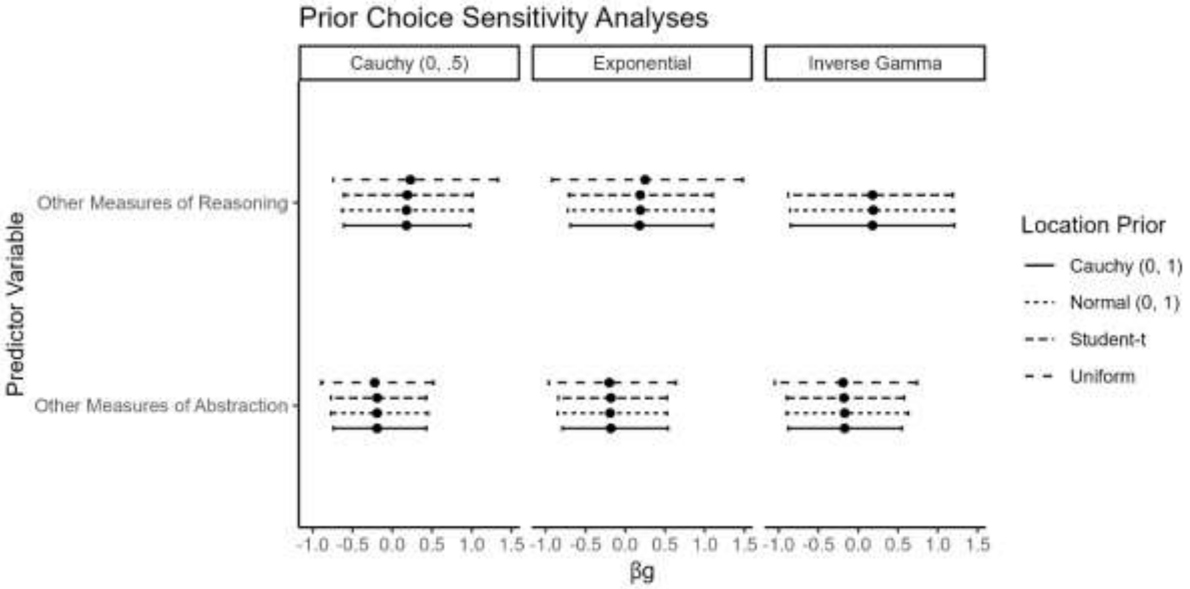

Figure 1. Regression coefficients with 95% confidence intervals for the models of Other Measures of Reasoning and Other Measures of Abstraction. For tests included in the analysis see Supplementary Materials 1.

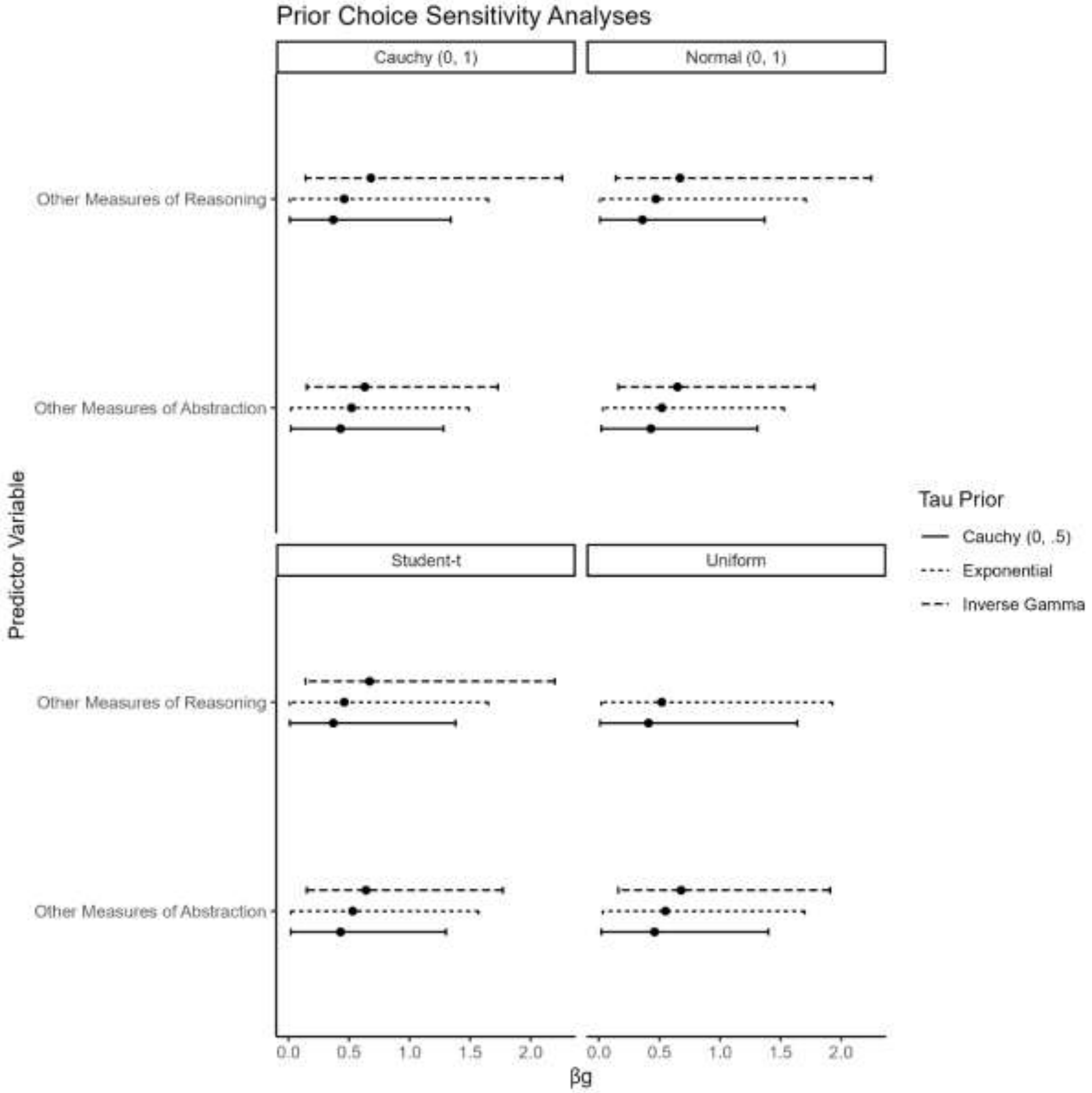

Figure 1. Study level standard deviation estimates with 95% confidence intervals for the models of Other Measures of Reasoning and Other Measures of Abstraction. For tests included in the analysis see Supplementary Materials 1.

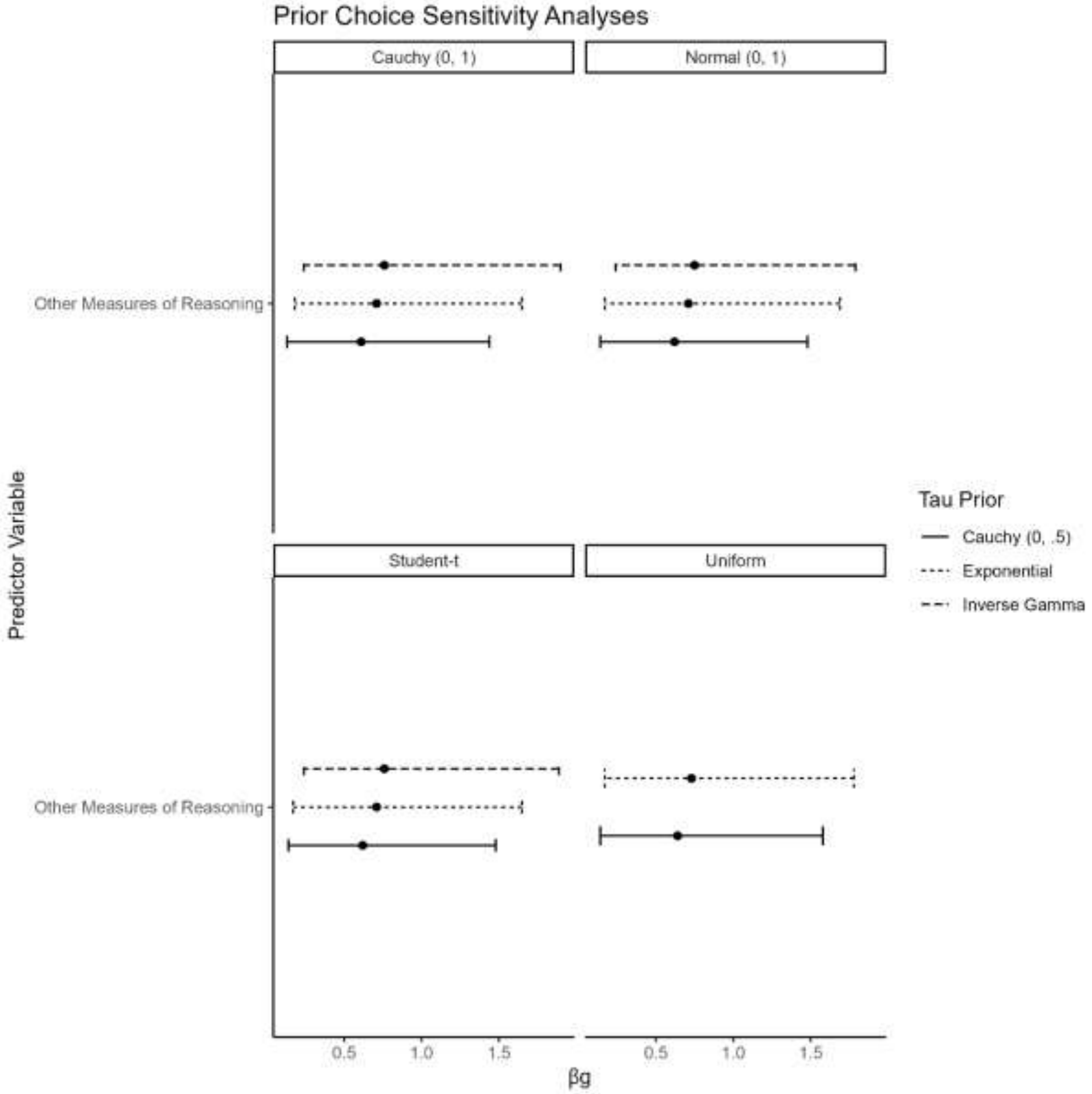

Figure 1. Effect size level standard deviation estimates with 95% confidence intervals for the models of Other Measures of Reasoning and Other Measures of Abstraction. For tests included in the analysis see Supplementary Materials 1.

Executive Functioning

Wechsler Adult Intelligence Scale: Arithmetic & Total Digits

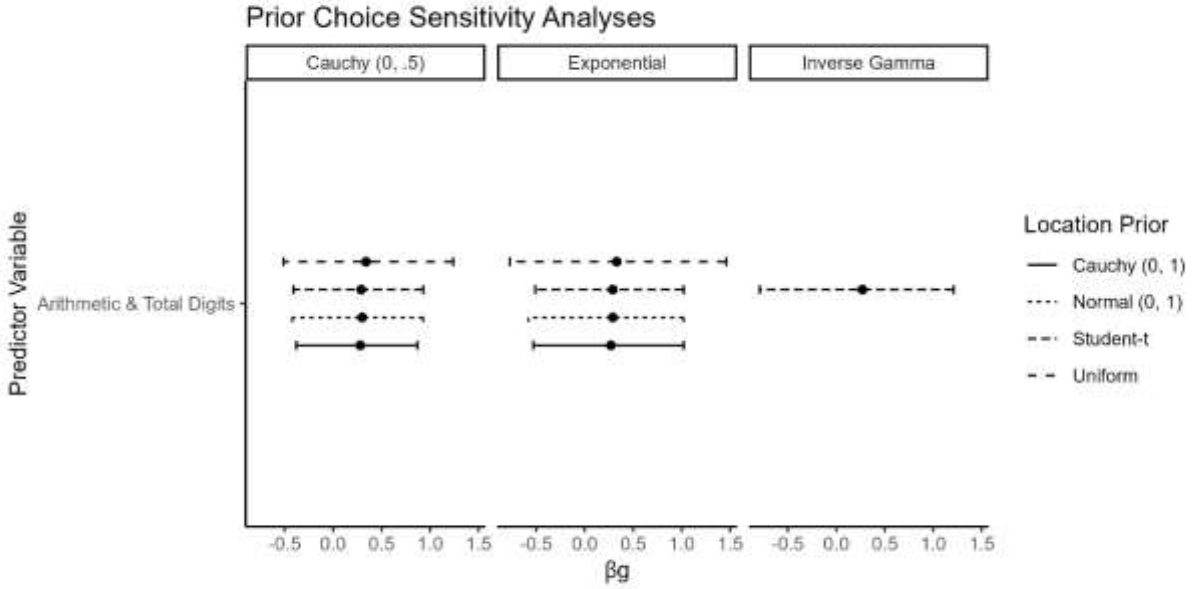

Figure 1. Regression coefficients with 95% confidence intervals for the Wechsler Adult Intelligence Scale: Arithmetic and Total Digits model.

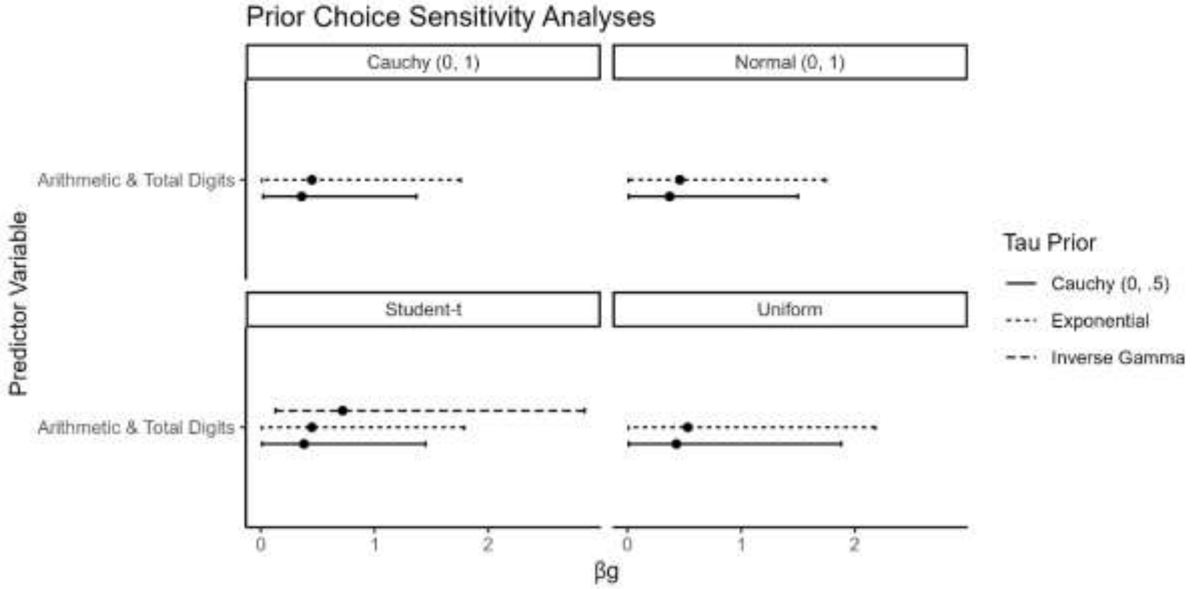

Figure 1. Study level standard deviation estimates with 95% confidence intervals for the Wechsler Adult Intelligence Scale: Arithmetic and Total Digits model.

Trail Making Test

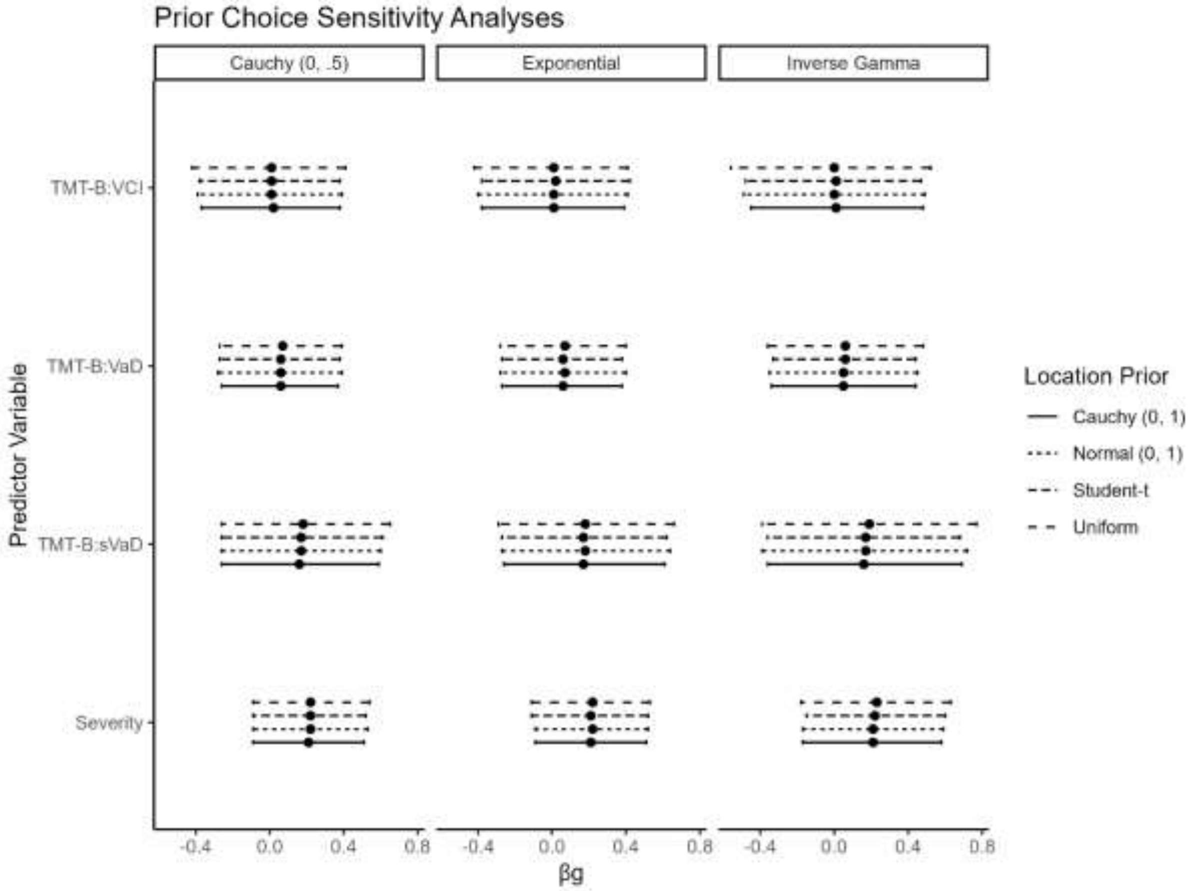

Figure 1. Regression coefficients with 95% confidence intervals for the Trail Making Test: B model. sVaD: subcortical vascular dementia, VaD: vascular dementia, VCI: vascular cognitive impairment, Severity: difference in dementia severity between dementia groups.

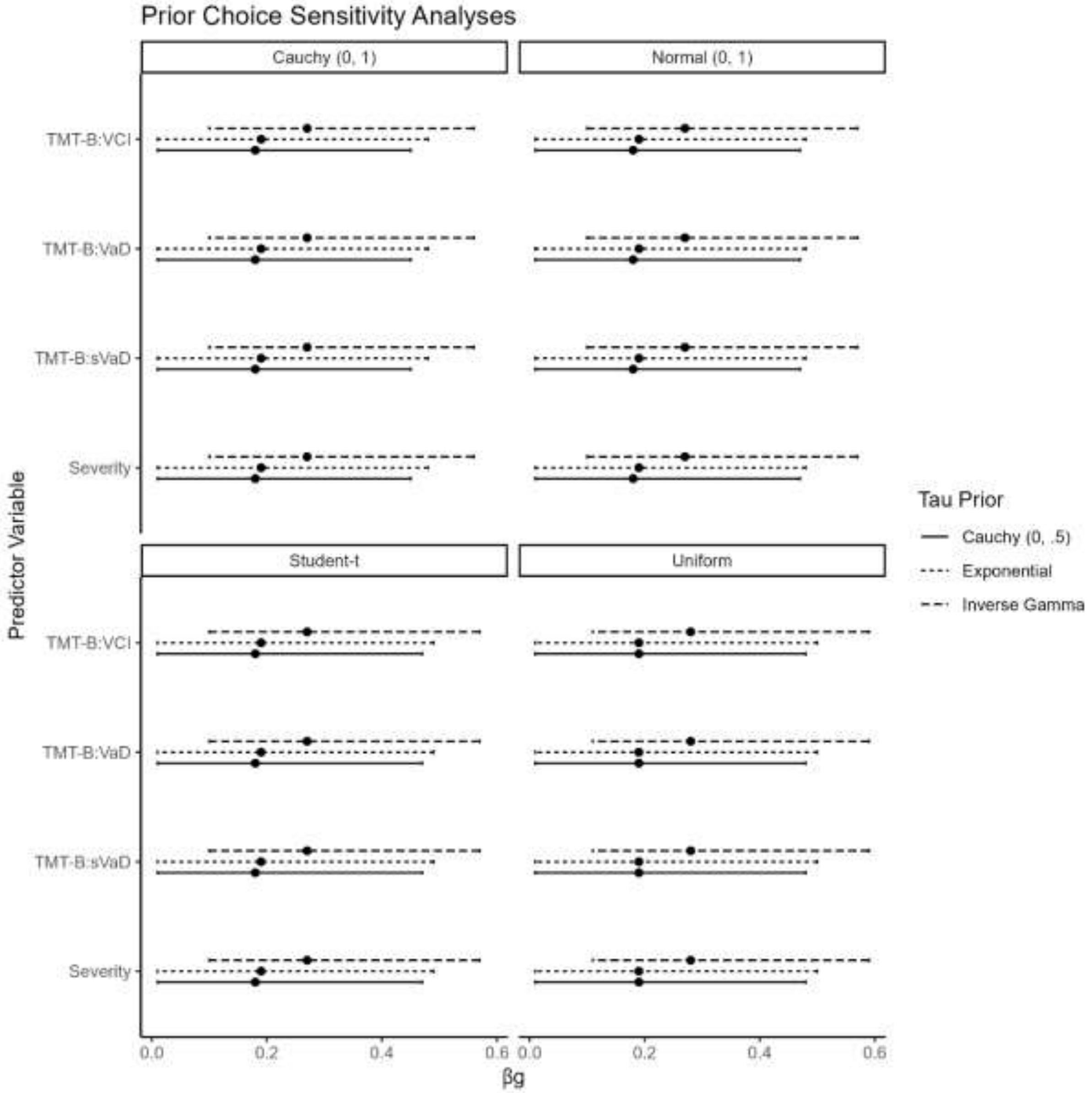

Figure 1. Study level standard deviation estimates with 95% confidence intervals for the Trail Making Test: B model. sVaD: subcortical vascular dementia, VaD: vascular dementia, VCI: vascular cognitive impairment, Severity: difference in dementia severity between dementia groups.

Frontal Assessment Battery Total Score

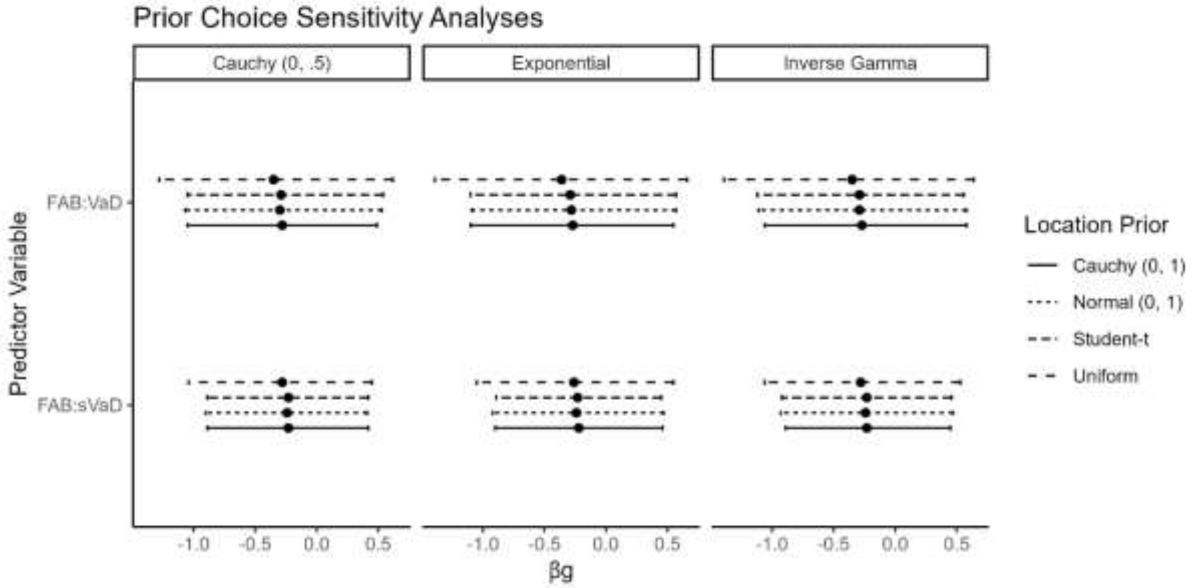

Figure 1. Regression coefficients with 95% confidence intervals for the Frontal Assessment Battery (FAB) Total Score model. sVaD: subcortical vascular dementia, VaD: vascular dementia.

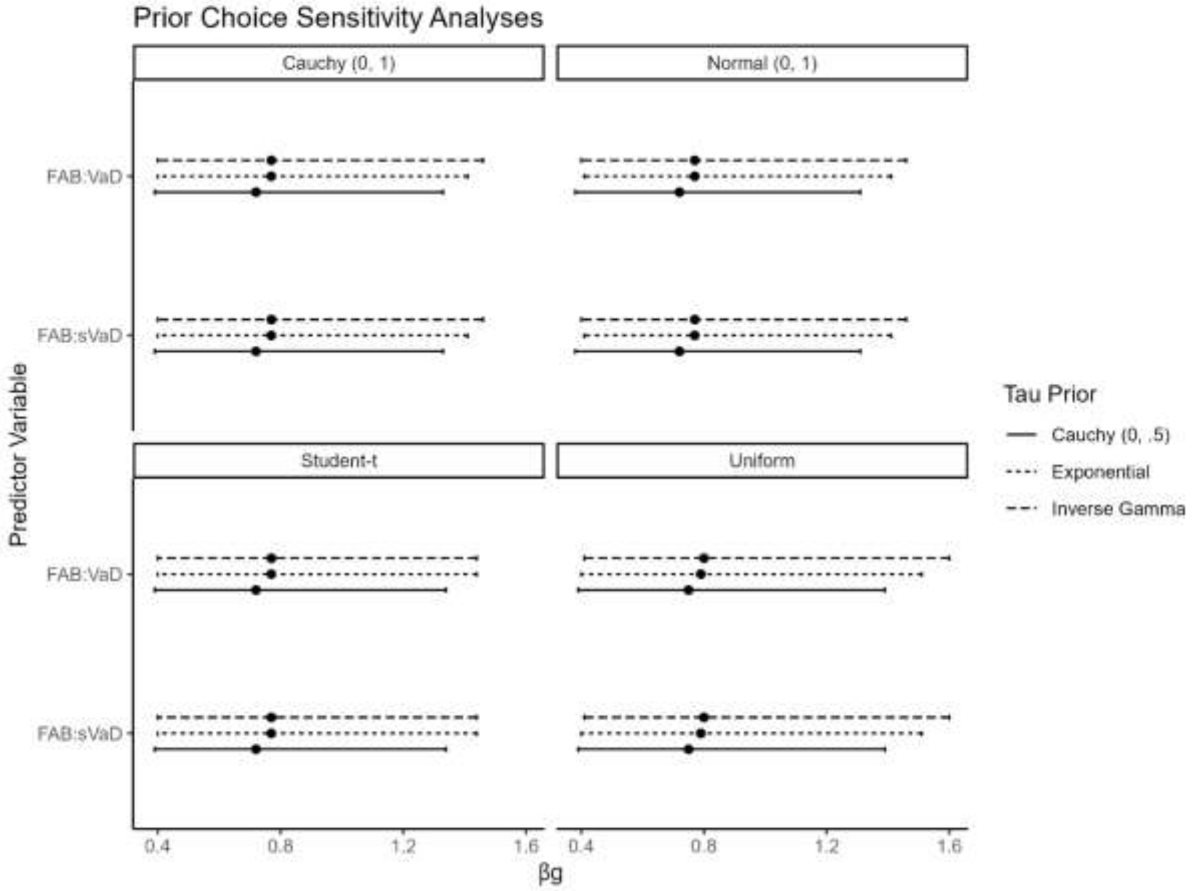

Figure 1. Study level standard deviation estimates with 95% confidence intervals for the Frontal Assessment Battery (FAB) Total Score model. sVaD: subcortical vascular dementia, VaD: vascular dementia.

Stroop Interference Condition

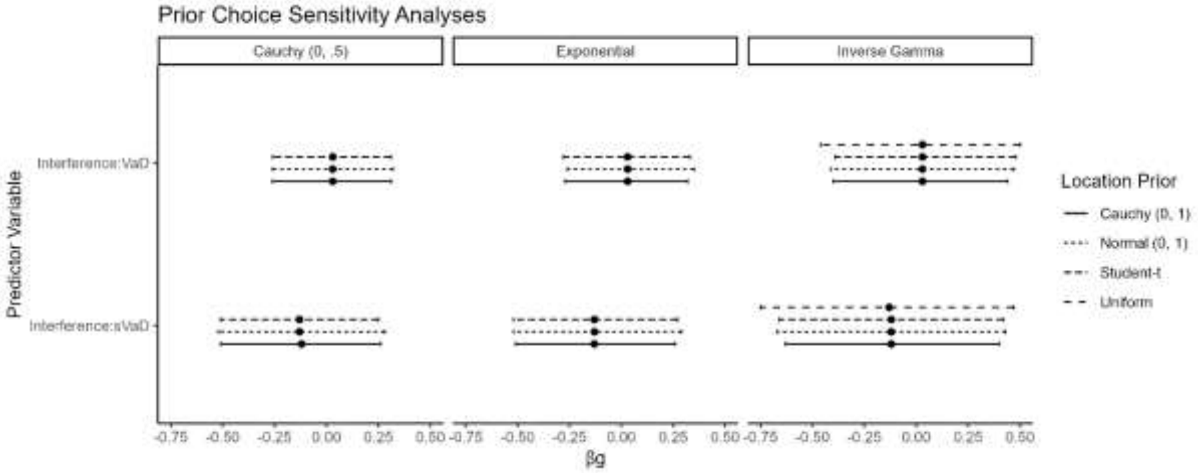

Figure 1. Regression coefficients with 95% confidence intervals for the Stroop Task Interference Condition model. sVaD: subcortical vascular dementia, VaD: vascular dementia.

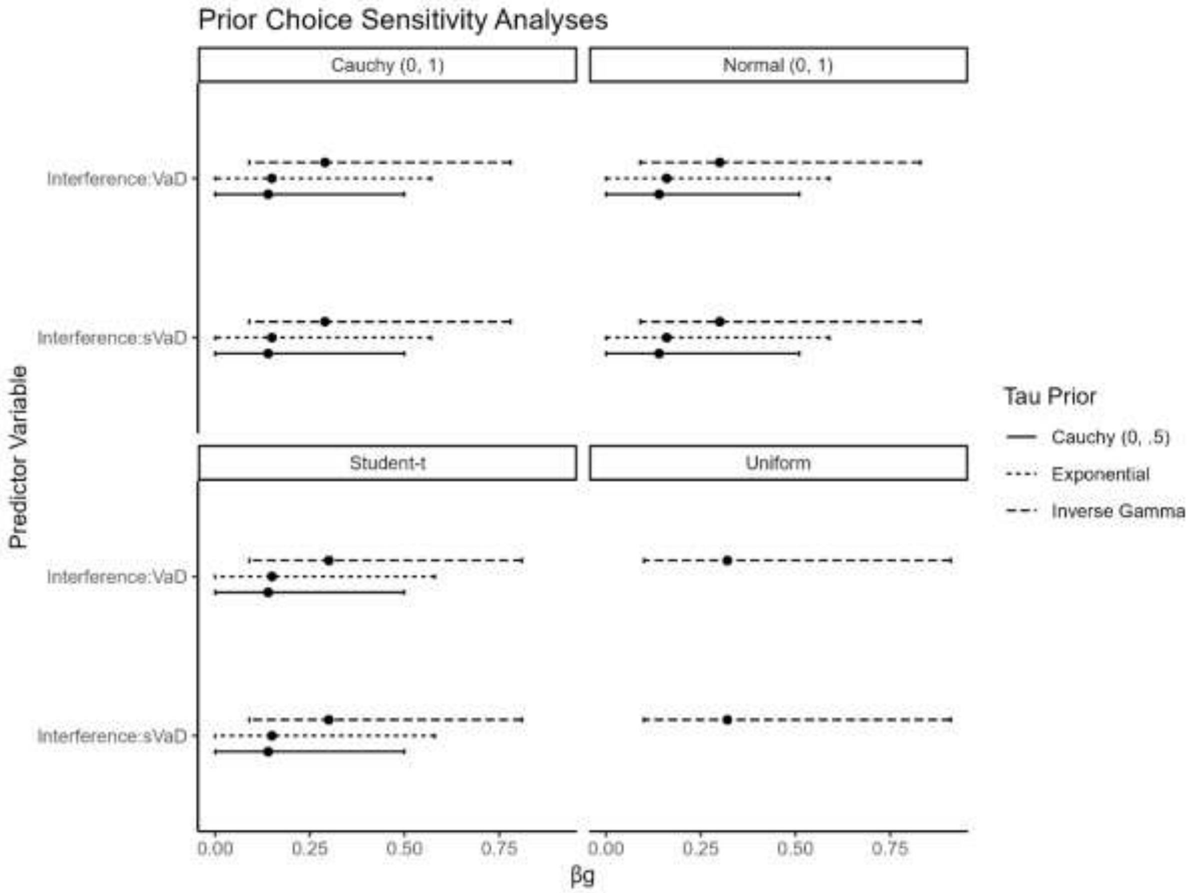

Figure 1. Study level standard deviation estimates with 95% confidence intervals for the Stroop Task Interference Condition model. sVaD: subcortical vascular dementia, VaD: vascular dementia.

Wechsler Memory Scale

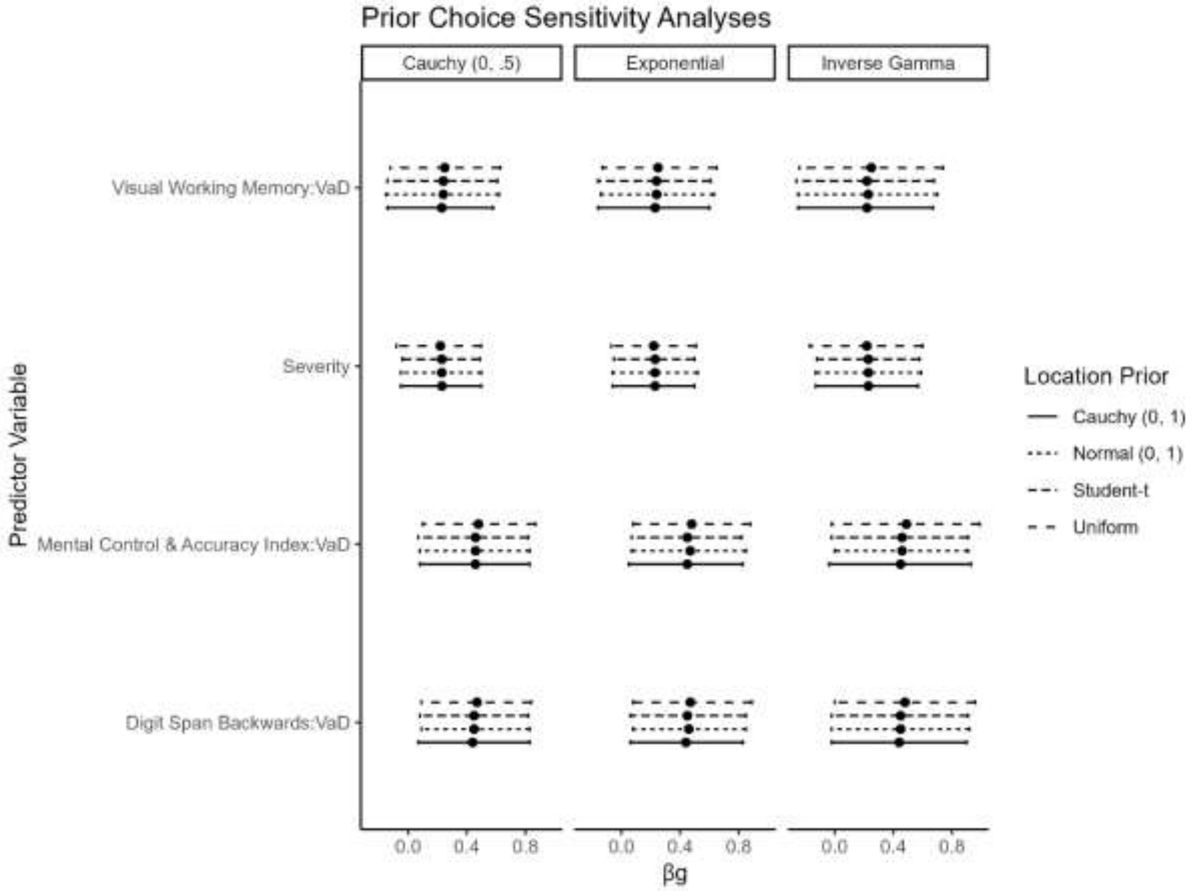

Figure 1. Regression coefficients with 95% confidence intervals for the Wechsler Memory Scale Visual Working Memory, Mental Control & Accuracy Index and Digit Span Backwards models. VaD: vascular dementia, Severity: difference in dementia severity between dementia groups.

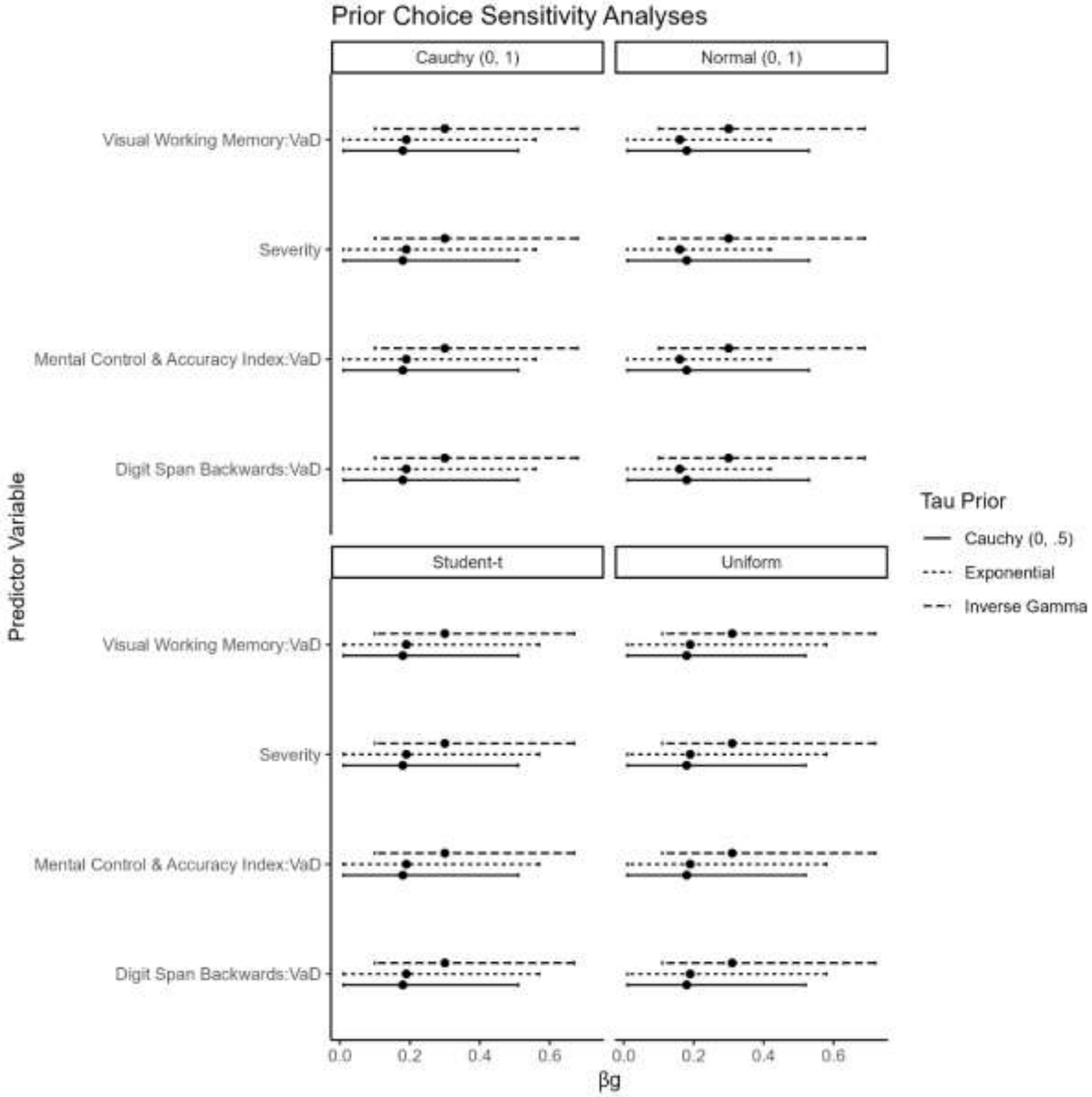

Figure 1. Study level standard deviation estimates with 95% confidence intervals for the Wechsler Memory Scale Visual Working Memory, Mental Control & Accuracy Index and Digit Span Backwards models. VaD: vascular dementia, Severity: difference in dementia severity between dementia groups.

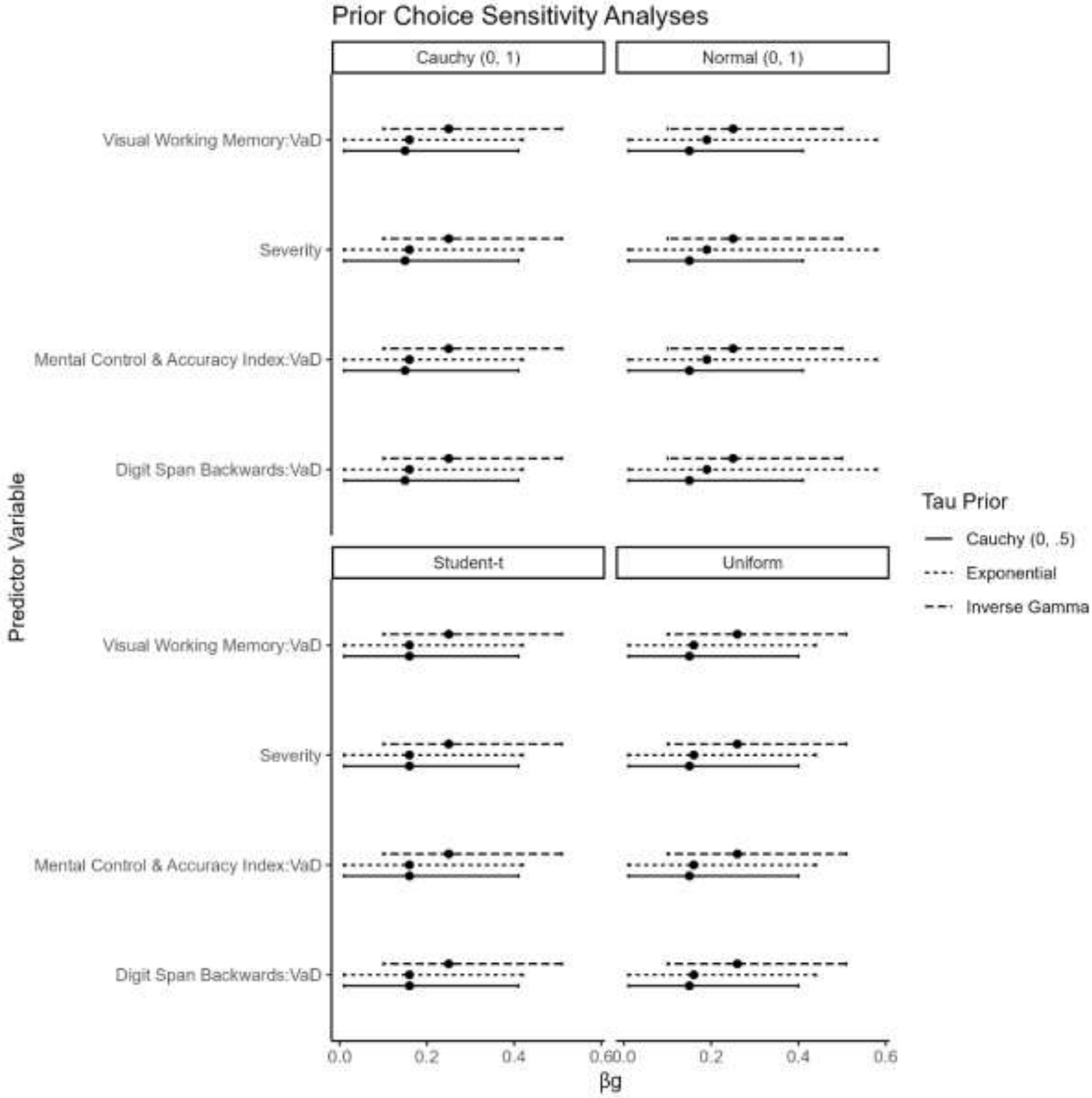

Figure 1. Effect size level standard deviation estimates with 95% confidence intervals for the Wechsler Memory Scale Visual Working Memory, Mental Control & Accuracy Index and Digit Span Backwards models. VaD: vascular dementia, Severity: difference in dementia severity between dementia groups.

Digit Span Backward

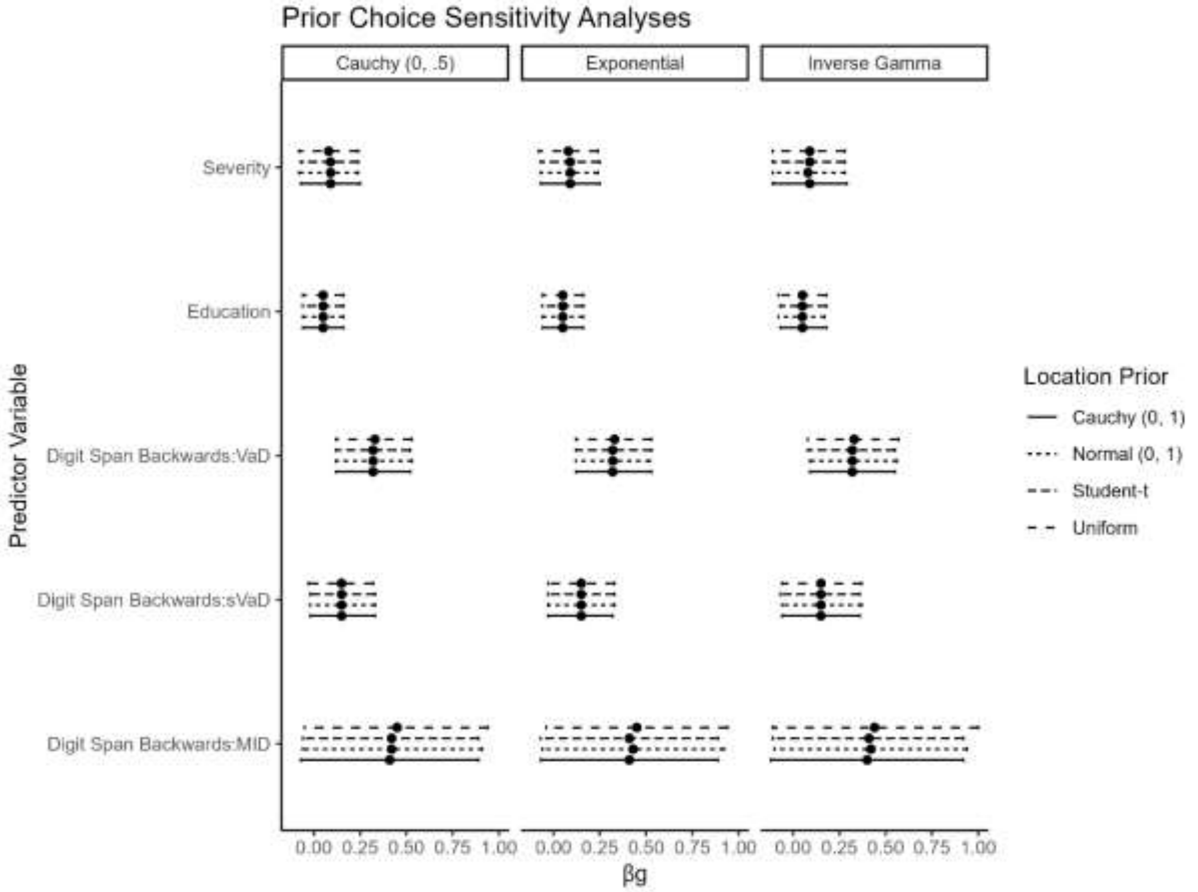

Figure 1. Regression coefficients with 95% confidence intervals for the other measures of the Digit Span Backwards model. sVaD: subcortical vascular dementia, VaD: vascular dementia, MID: multi-infarct dementia, Severity: difference in dementia severity between dementia groups, Education: difference in average years of education between dementia groups.

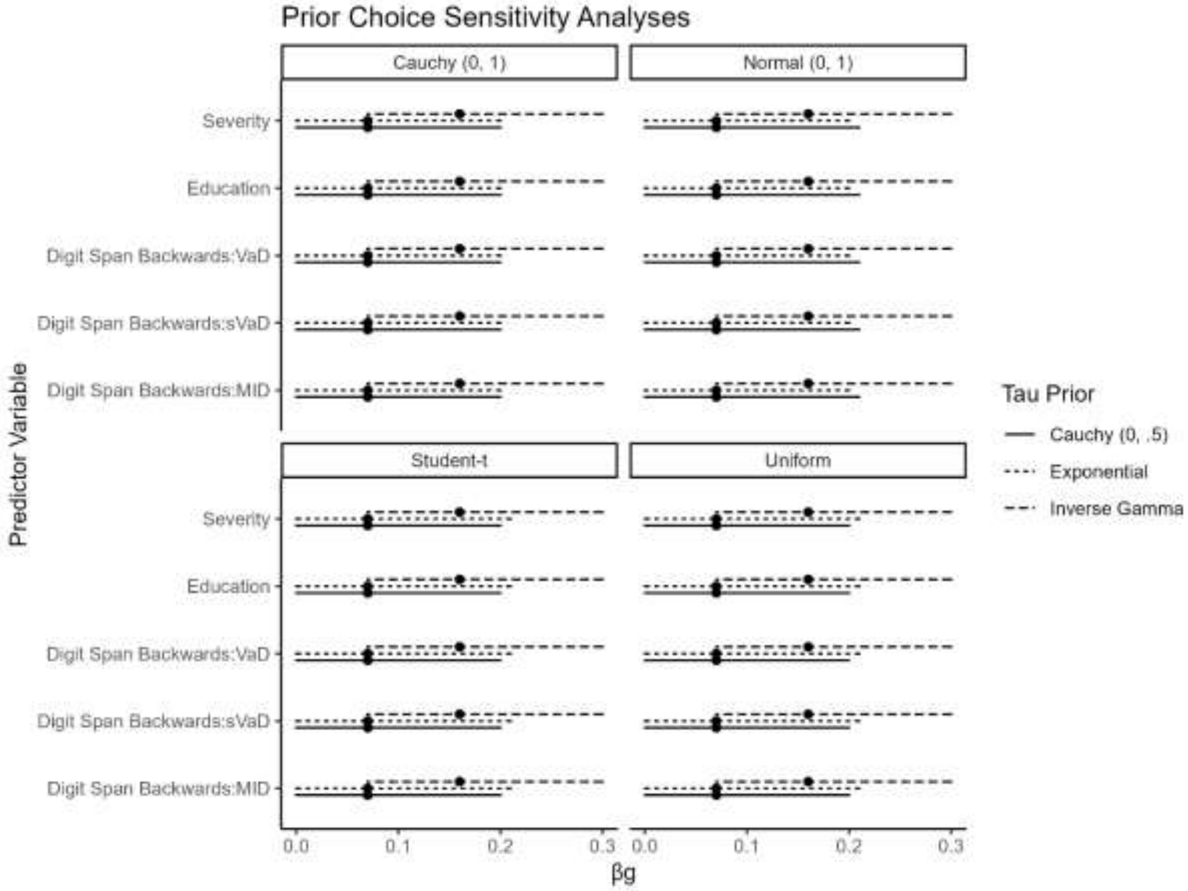

Figure 1. Study level standard deviation estimates coefficients with 95% confidence intervals for the other measures of the Digit Span Backwards model. sVaD: subcortical vascular dementia, VaD: vascular dementia, MID: multi-infarct dementia, Severity: difference in dementia severity between dementia groups, Education: difference in average years of education between dementia groups.

Visual Span

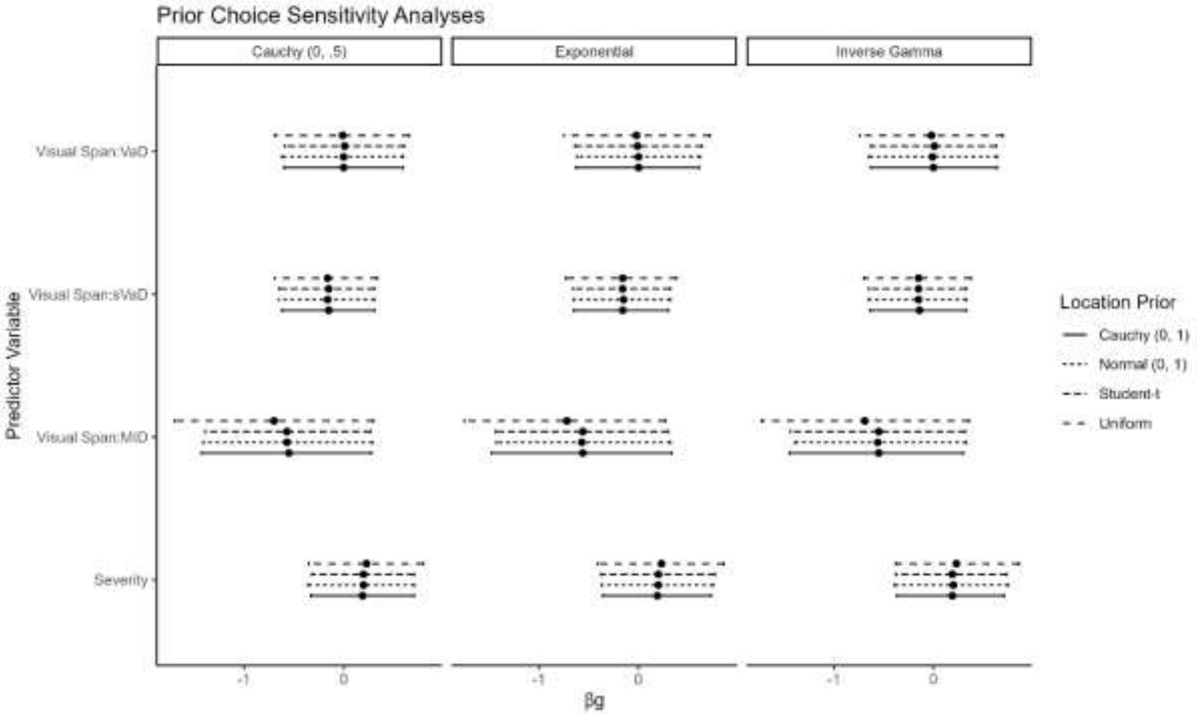

Figure 1. Regression coefficients with 95% confidence intervals for the measures of Visual Span model. For tests included in the analysis see Supplementary Materials 1. sVaD: subcortical vascular dementia, VaD: vascular dementia, MID: multi-infarct dementia, Severity: difference in dementia severity between dementia groups.

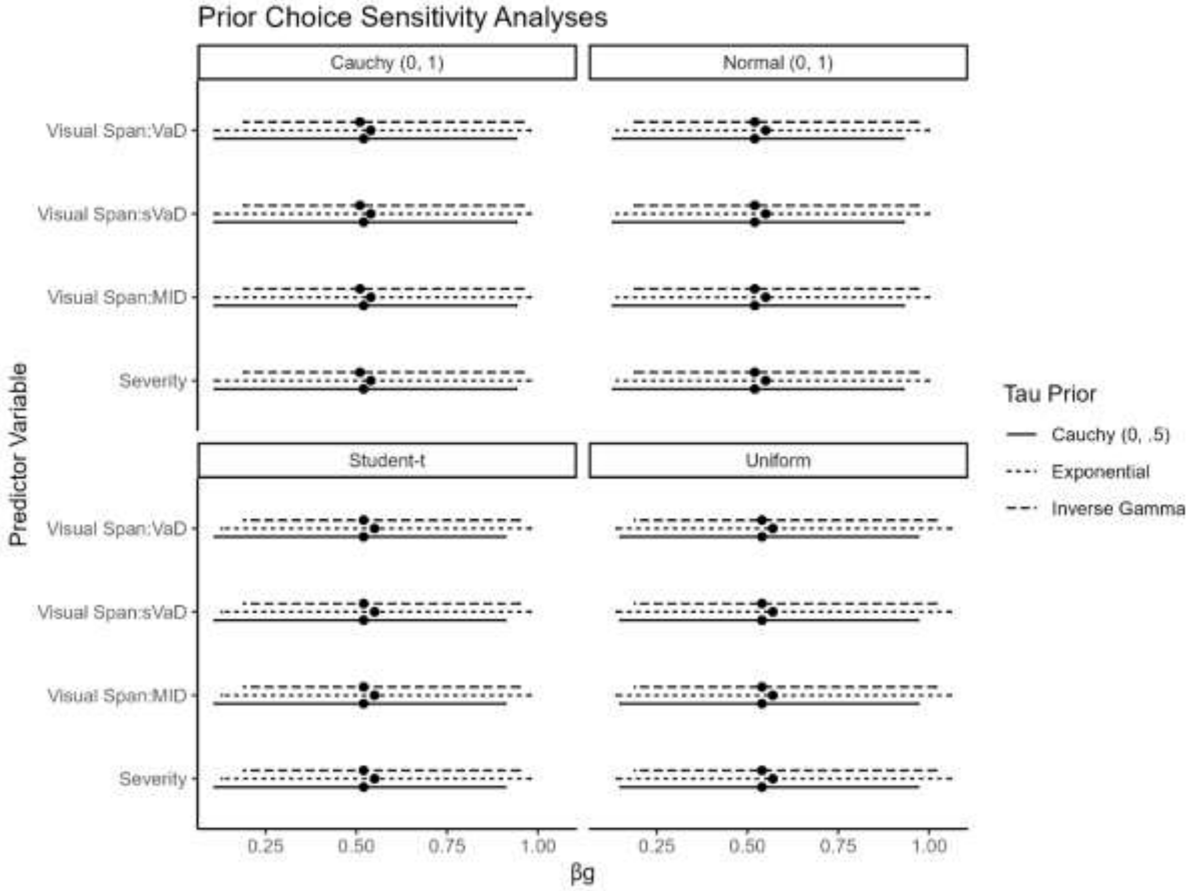

Figure 1. Study level standard deviation estimates with 95% confidence intervals for the measures of Visual Span model. For tests included in the analysis see Supplementary Materials 1. sVaD: subcortical vascular dementia, VaD: vascular dementia, MID: multi-infarct dementia, Severity: difference in dementia severity between dementia groups.

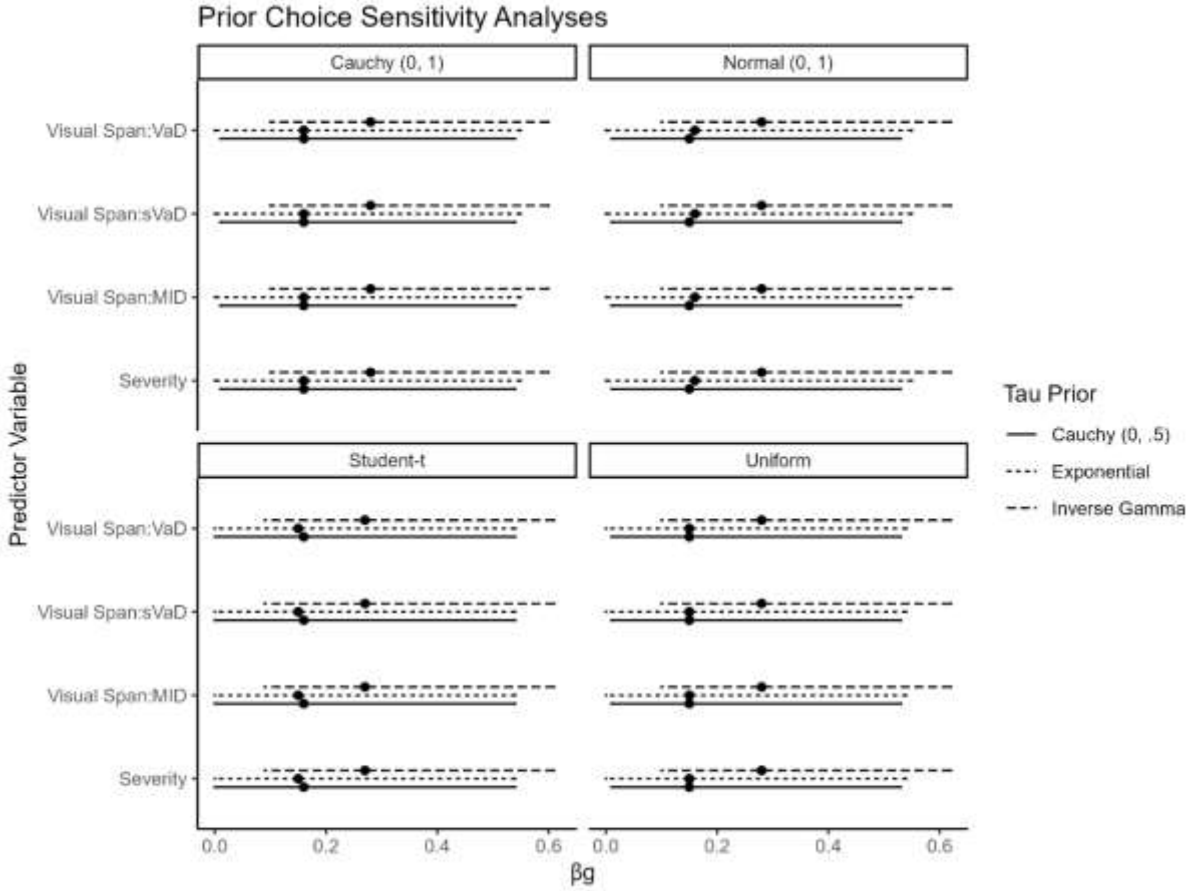

Figure 1. Effect size level standard deviation estimates with 95% confidence intervals for the measures of Visual Span model. For tests included in the analysis see Supplementary Materials 1. sVaD: subcortical vascular dementia, VaD: vascular dementia, MID: multi-infarct dementia, Severity: difference in dementia severity between dementia groups.

Maze Tasks

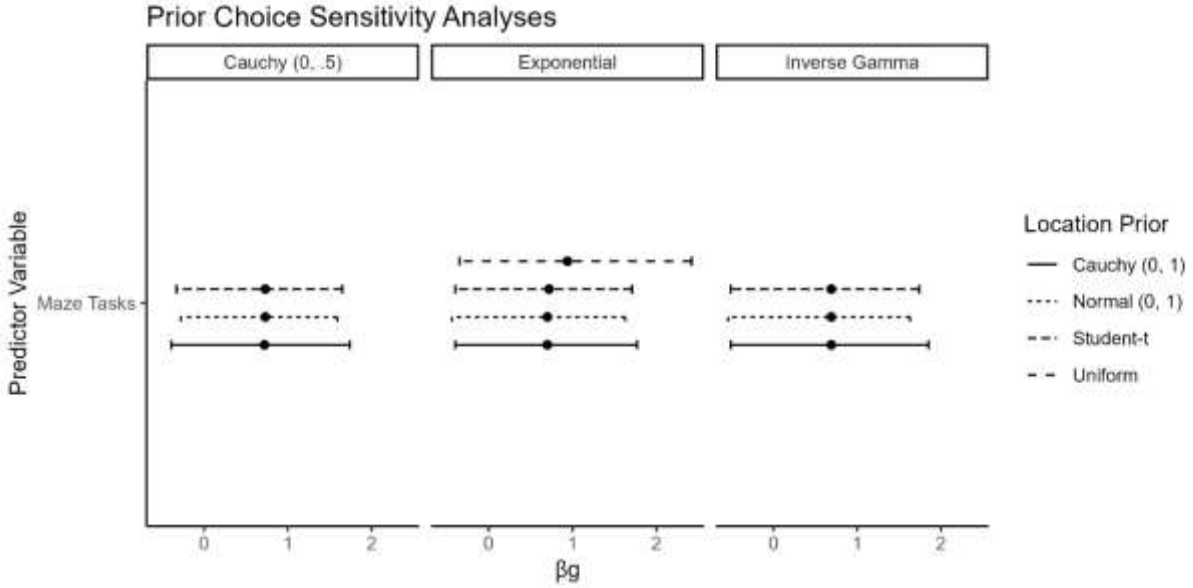

Figure 1. Regression coefficients with 95% confidence intervals for the Maze Tasks model. For tests included in the analysis see Supplementary Materials 1.

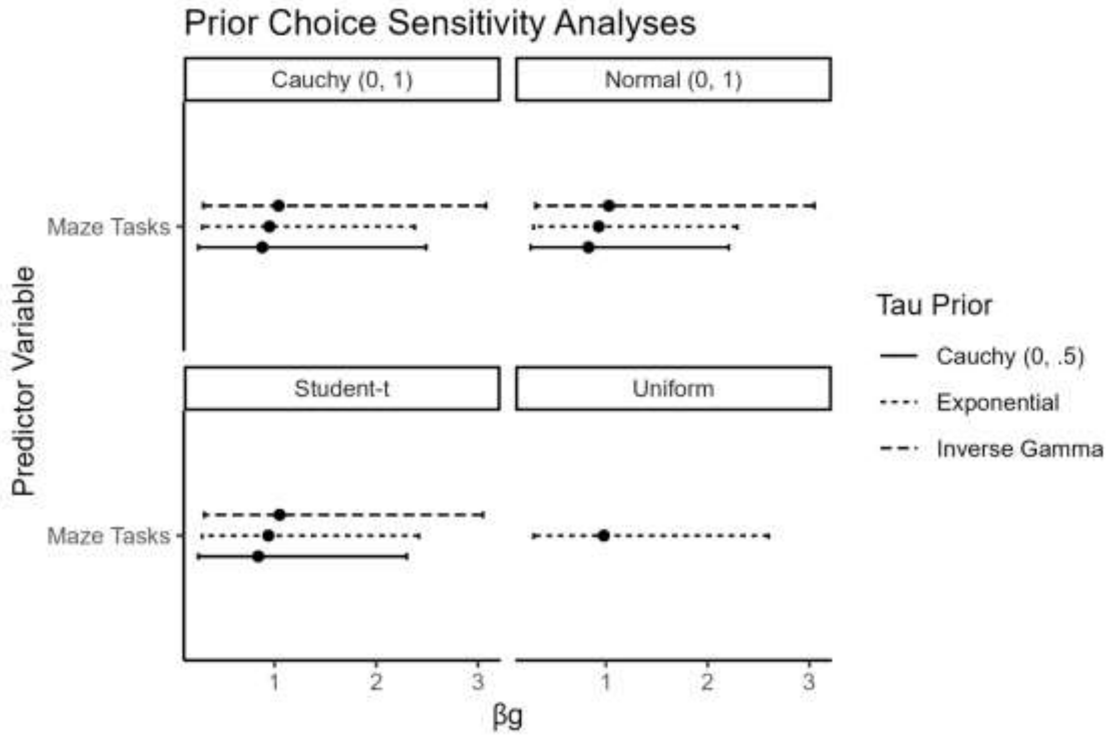

Figure 1. Study level standard deviation estimates with 95% confidence intervals for the Maze Tasks model. For tests included in the analysis see Supplementary Materials 1.

Graphical Sequence Test

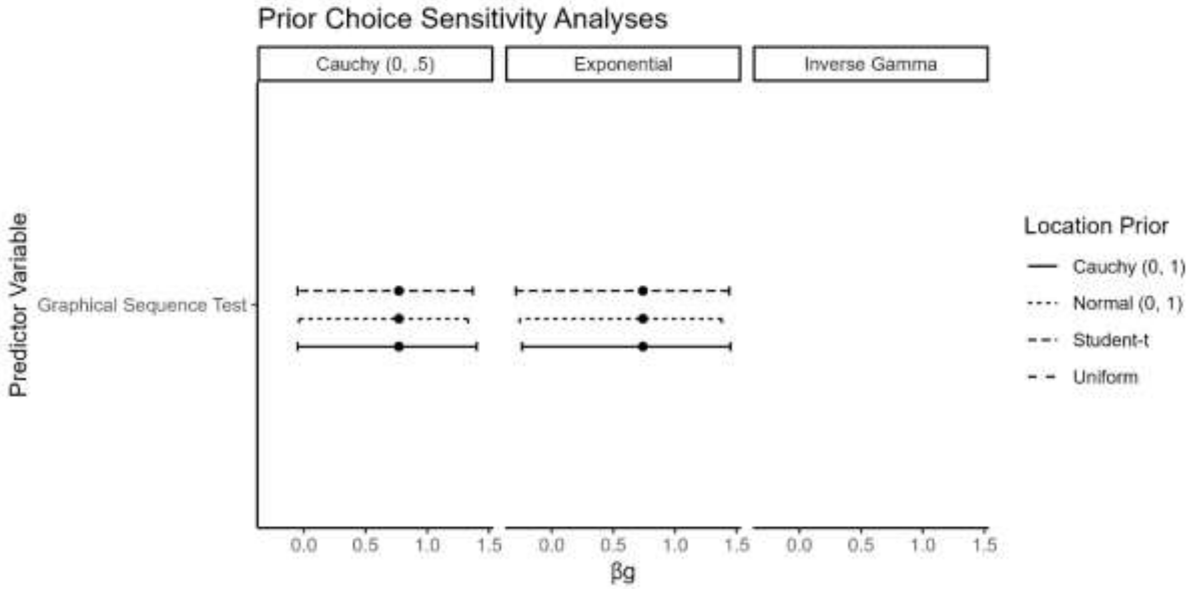

Figure 1. Regression coefficients with 95% confidence intervals for the Graphical Sequence Test model.

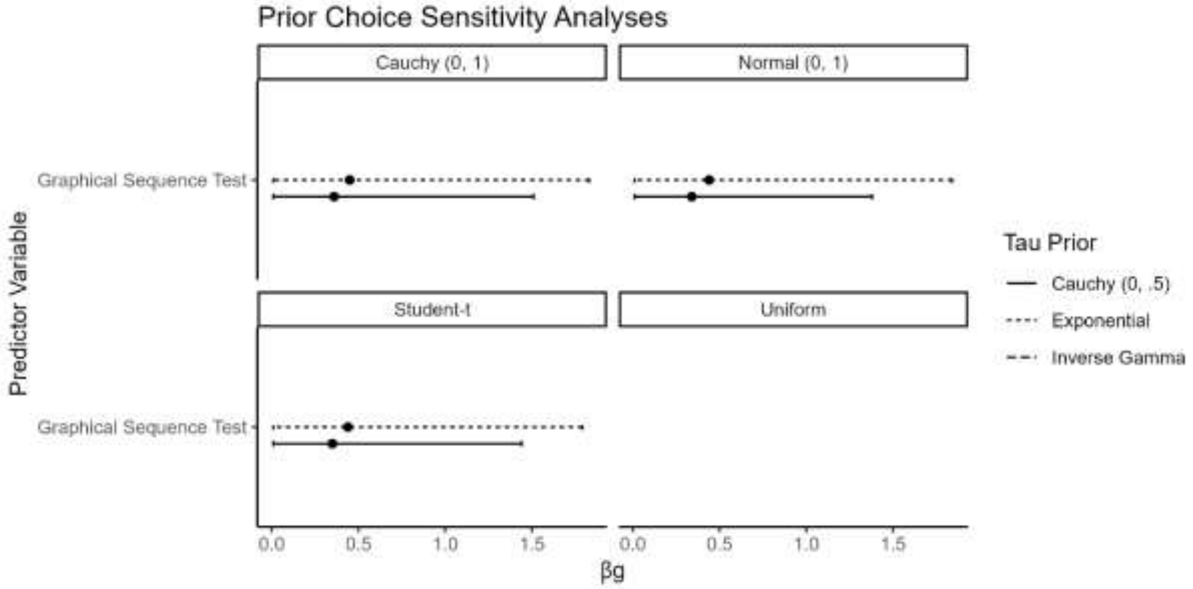

Figure 1. Study level standard deviation estimatess with 95% confidence intervals for the Graphical Sequence Test model.

Repetition of Words and Sentences

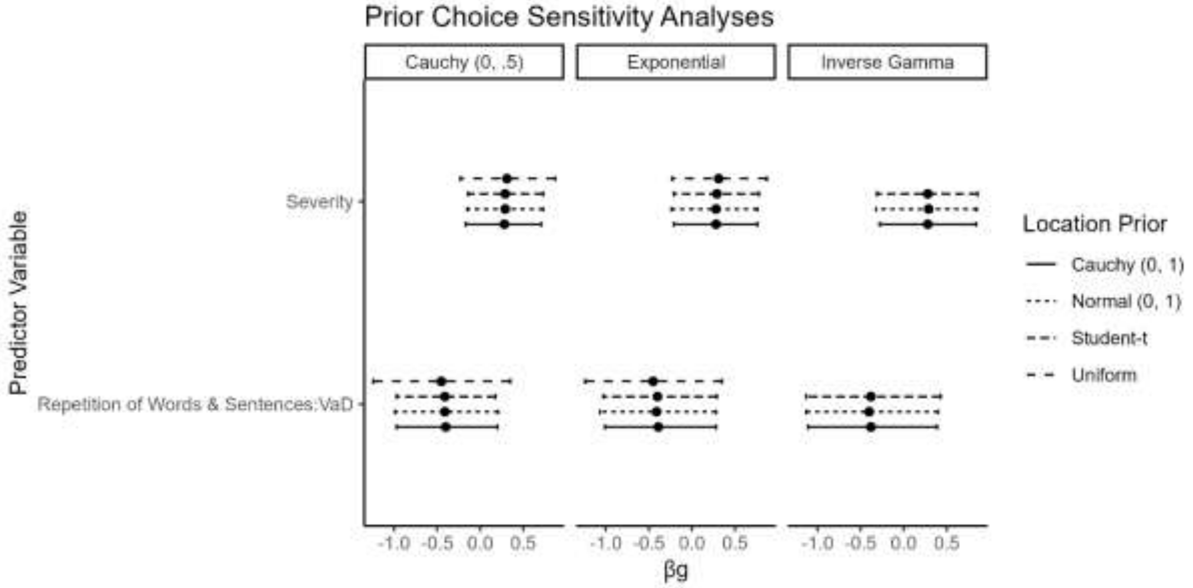

Figure 1. Regression coefficients with 95% confidence intervals for the Repetition of Words and Sentences Measures model. VaD: vascular dementia, Severity: difference in dementia severity between dementia groups.

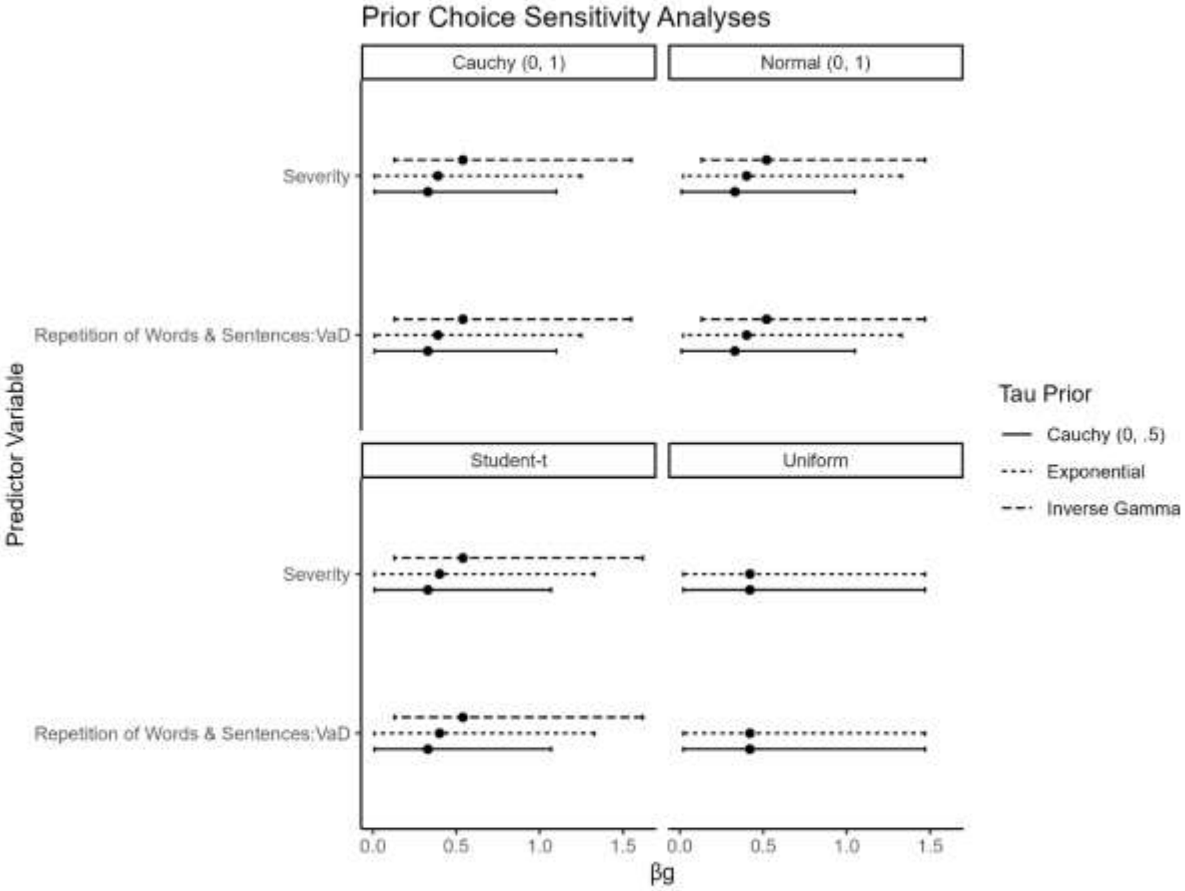

Figure 1. Study level standard deviation estimates with 95% confidence intervals for the Repetition of Words and Sentences Measures model. VaD: vascular dementia, Severity: difference in dementia severity between dementia groups.

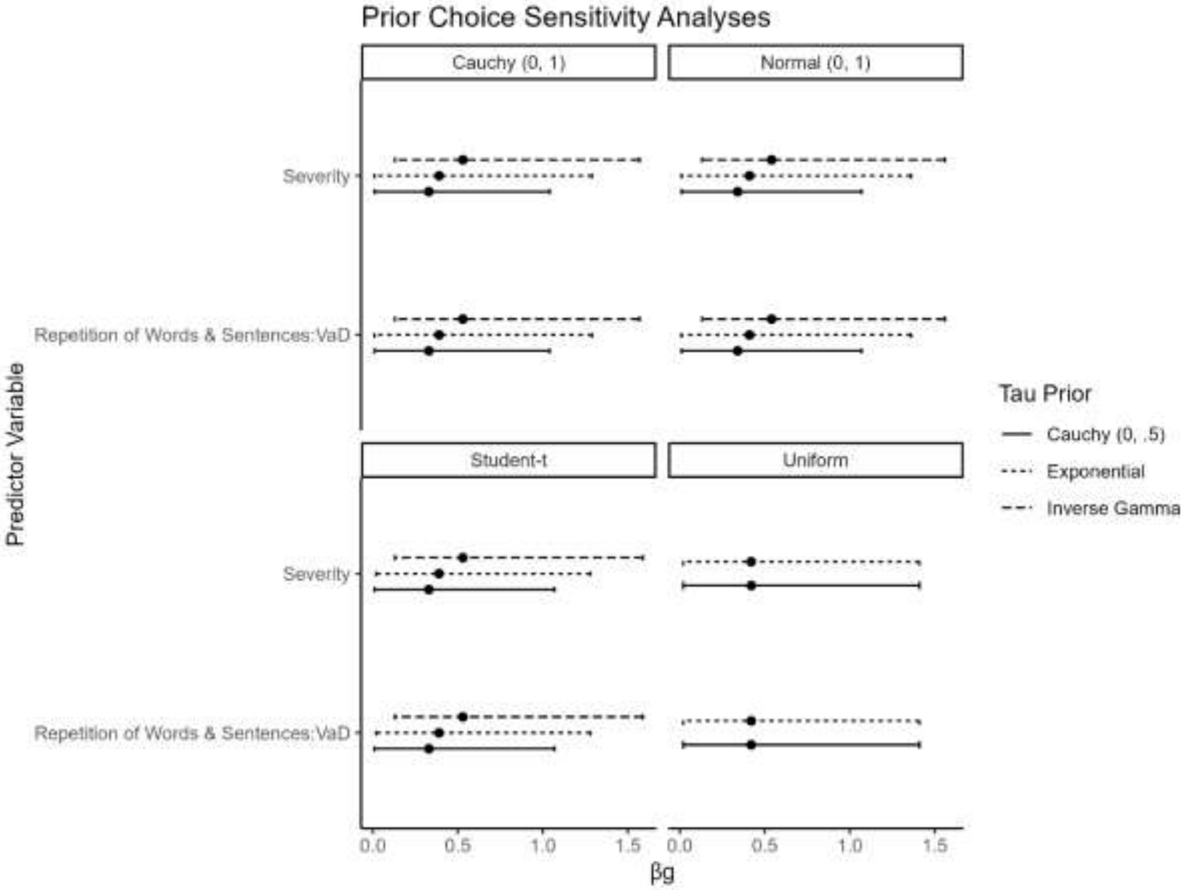

Figure 1. Effect size level standard deviation estimates with 95% confidence intervals for the Repetition of Words and Sentences Measures model. VaD: vascular dementia, Severity: difference in dementia severity between dementia groups.

Arithmetic

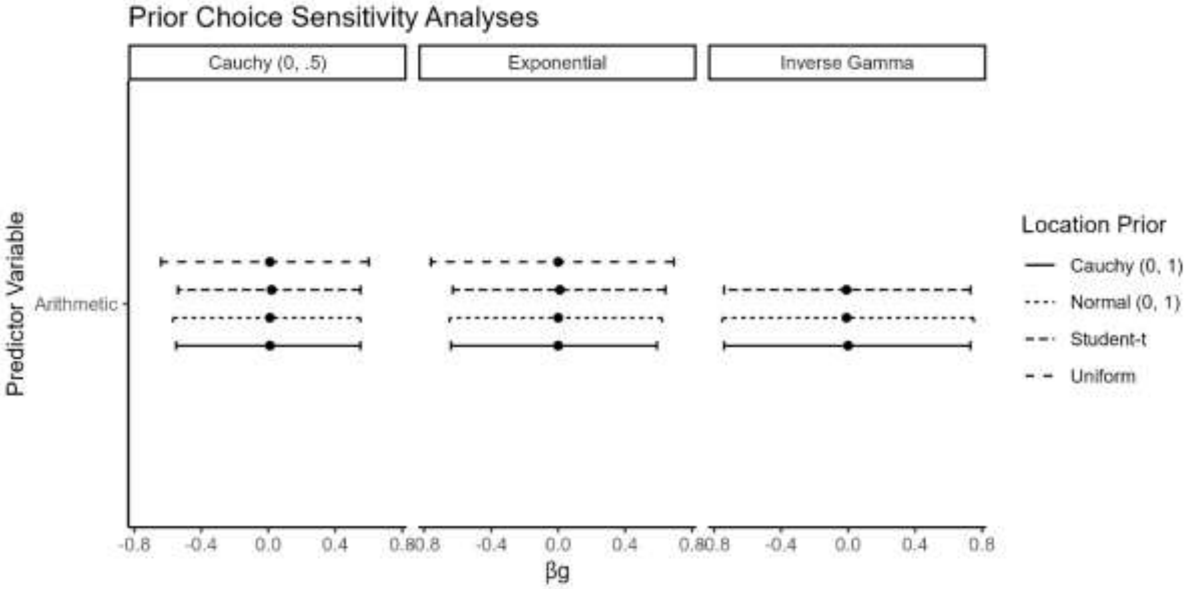

Figure 1. Regression coefficients with 95% confidence intervals for the other Arithmetic tests model. For tests included in the analysis see Supplementary Materials 1.

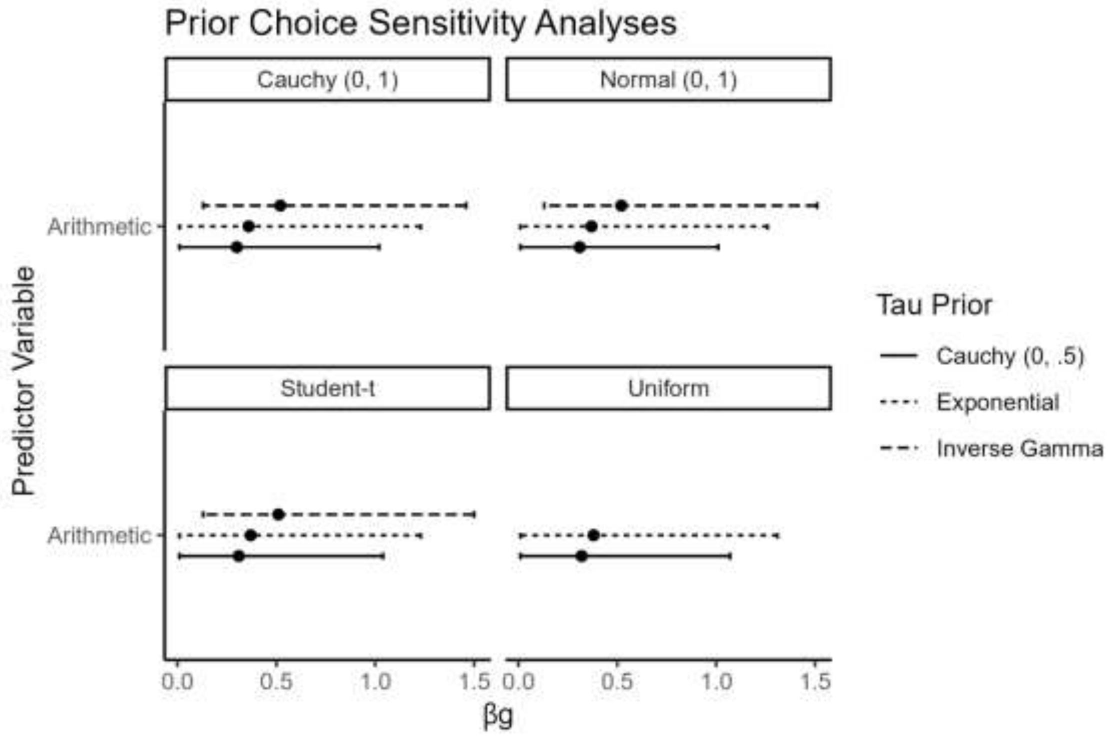

Figure 1. Study level standard deviation estimates with 95% confidence intervals for the other Arithmetic tests model. For tests included in the analysis see Supplementary Materials 1.

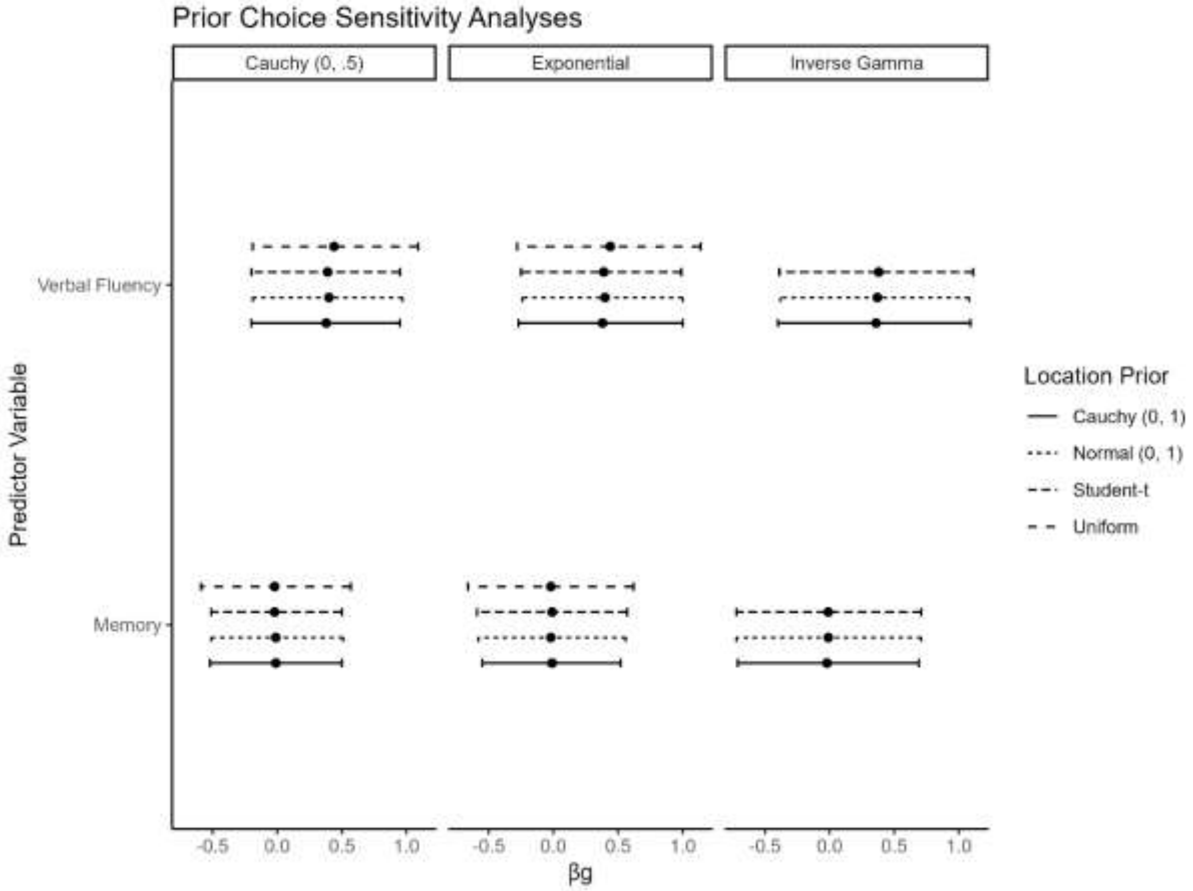

Figure 1. Regression coefficients with 95% confidence intervals for the Measures of Cognitive Control of Memory in verbal Fluency (i.e., recall from semantic memory) and in recall in episodic memory (Memory) model. For studies and measures included in the analysis see Supplementary Materials 1.

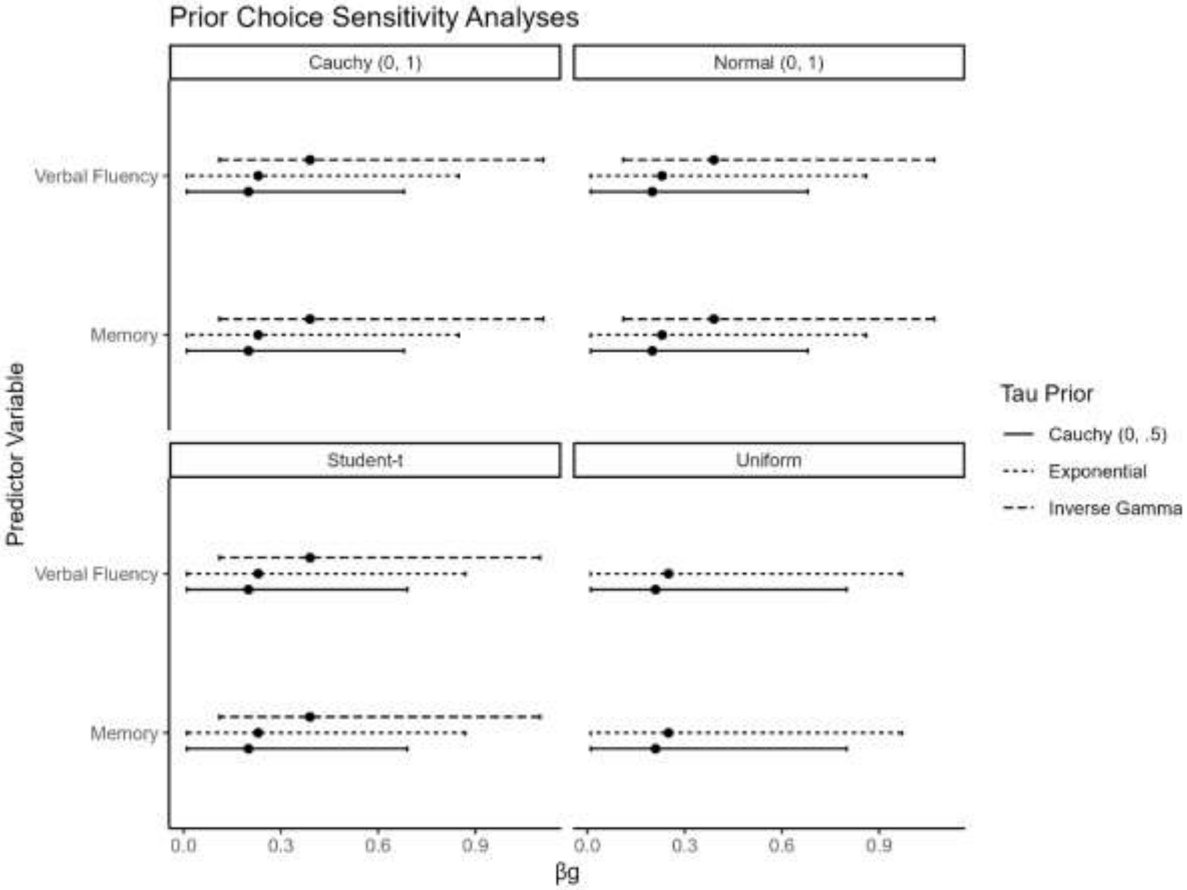

Figure 1. Study level standard deviation estimates with 95% confidence intervals for the Measures of Cognitive Control of Memory in verbal Fluency (i.e., recall from semantic memory) and in recall in episodic memory (Memory) model. For studies and measures included in the analysis see Supplementary Materials 1.

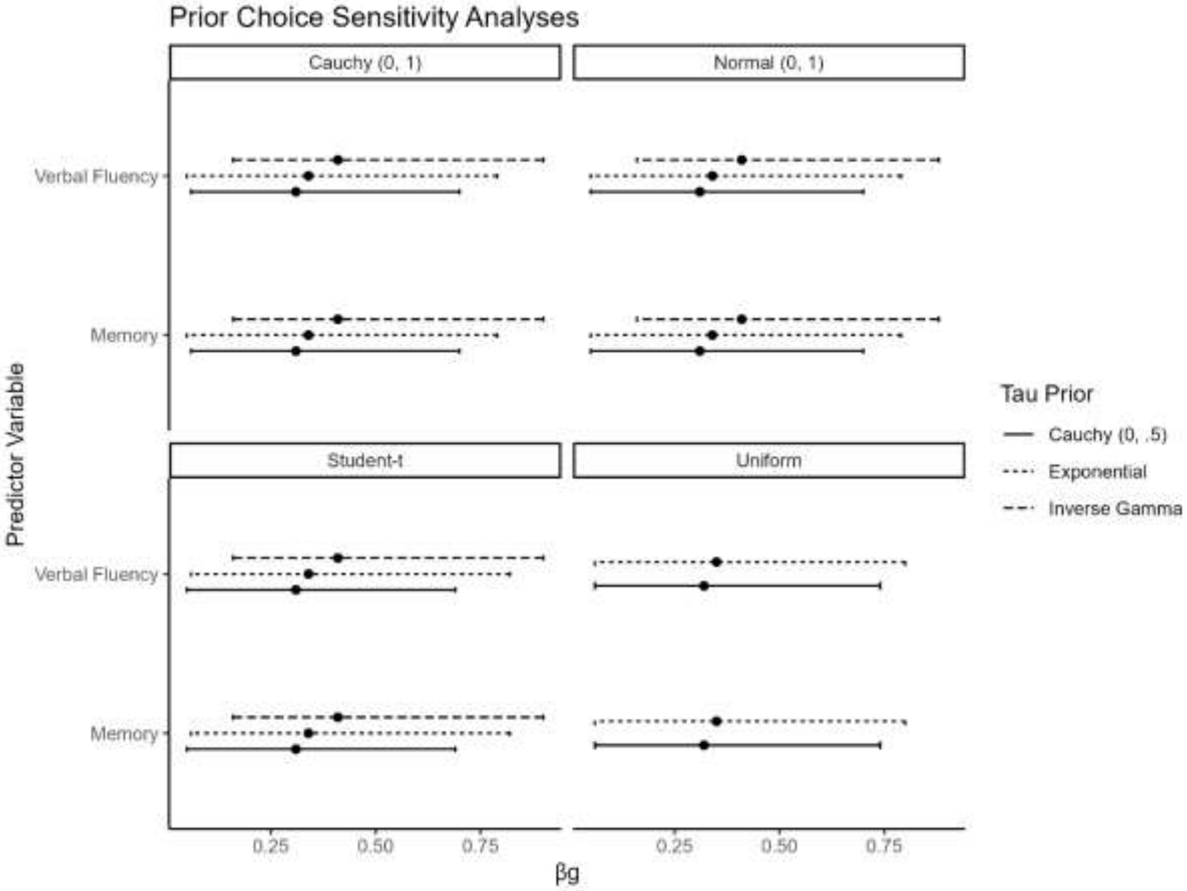

Figure 1. Effect size level standard deviation estimates with 95% confidence intervals for the Measures of Cognitive Control of Memory in verbal Fluency (i.e., recall from semantic memory) and in recall in episodic memory (Memory) model. For studies and measures included in the analysis see Supplementary Materials 1.

Other Measures of Cognitive Flexibility

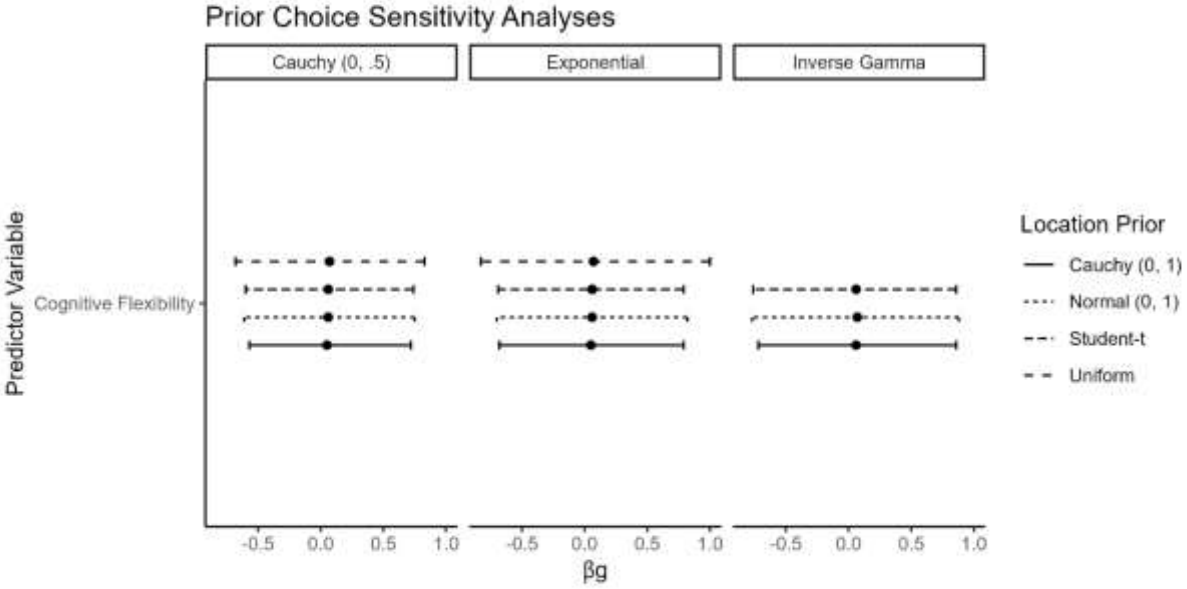

Figure 1. Regression coefficients with 95% confidence intervals for the Cognitive Flexibility model. For tests included in the analysis see Supplementary Materials 1.

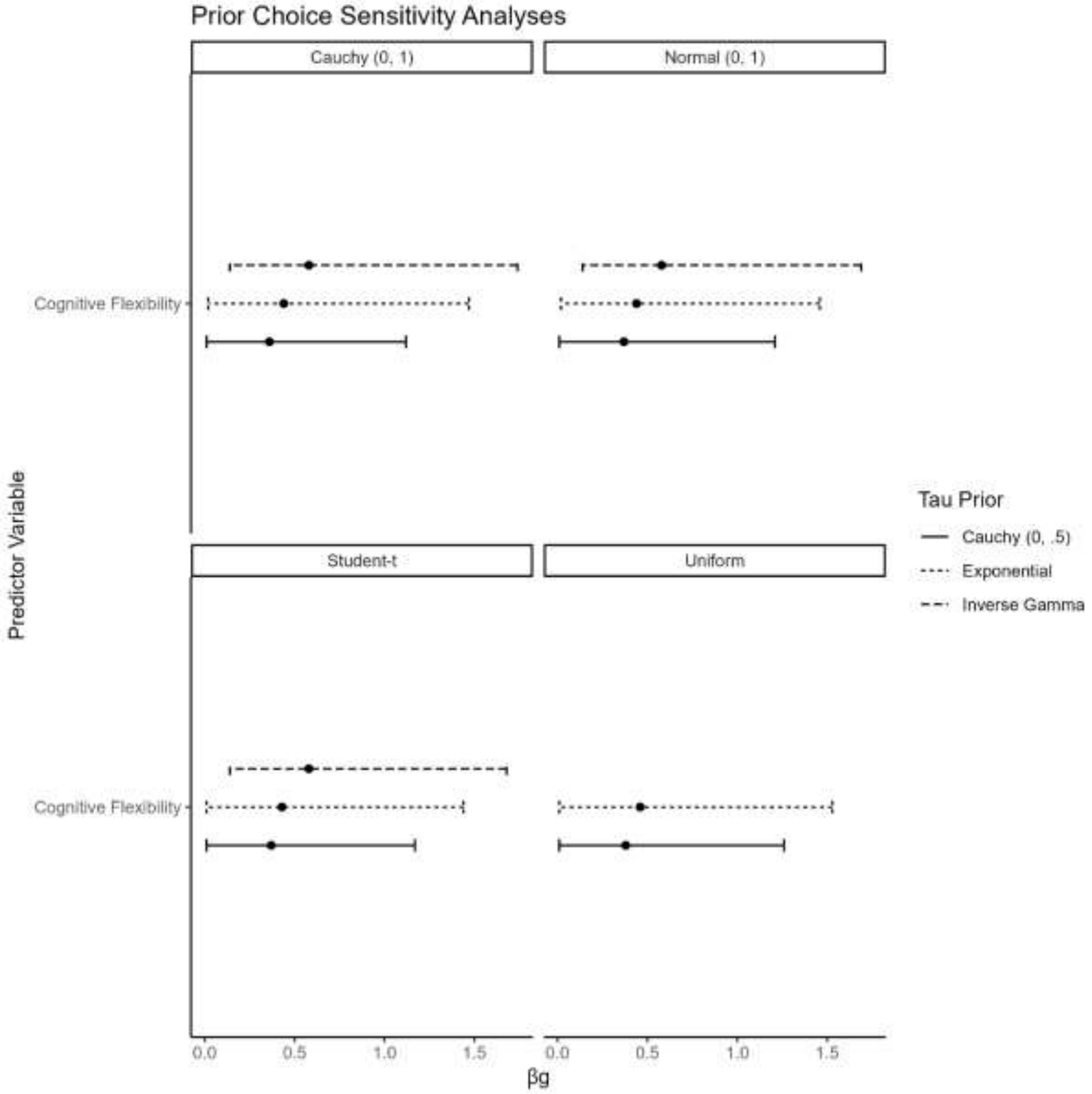

Figure 1. Study level standard deviation estimates with 95% confidence intervals for the Cognitive Flexibility model. For tests included in the analysis see Supplementary Materials 1.

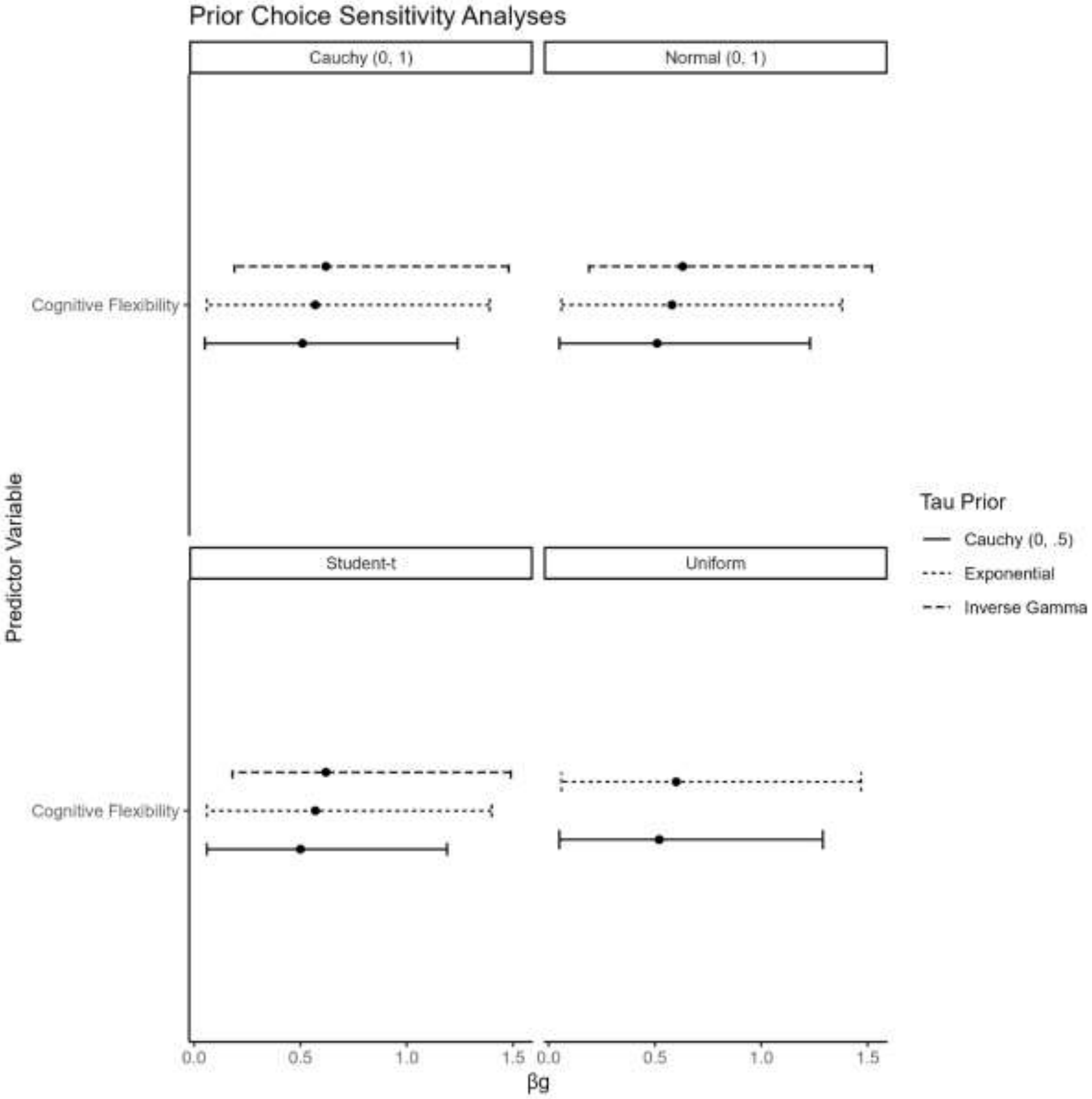

Figure 1. Effect size level standard deviation estimates with 95% confidence intervals for the Cognitive Flexibility model. For tests included in the analysis see Supplementary Materials 1.

Cognitive Estimation

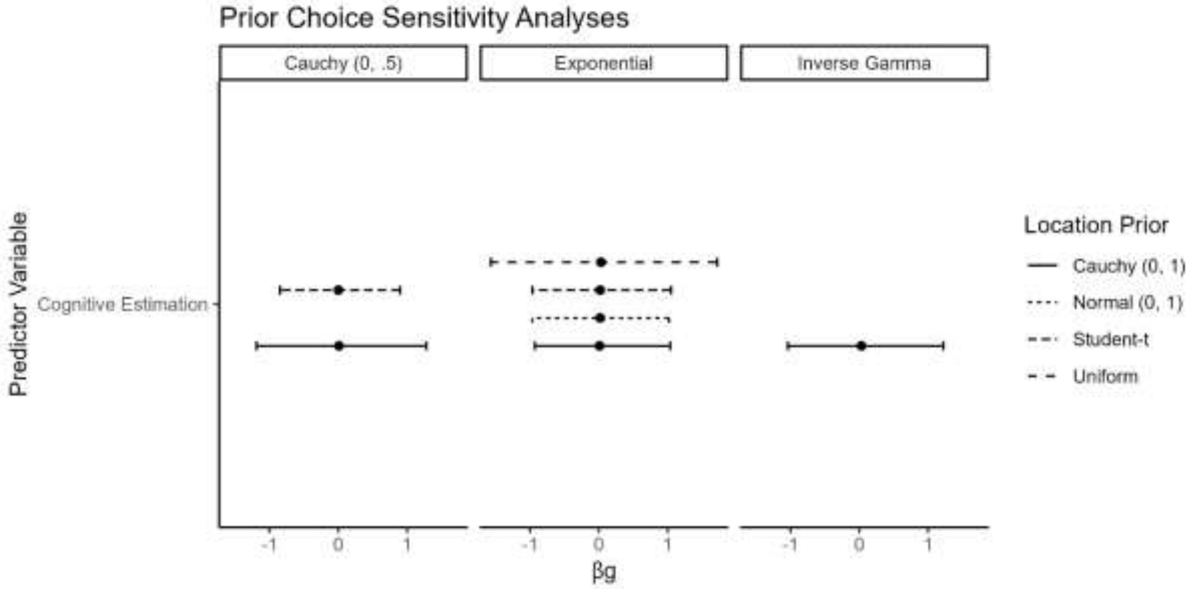

Figure 1. Regression coefficients with 95% confidence intervals for the Cognitive Estimation model. For tests included in the analysis see Supplementary Materials 1.

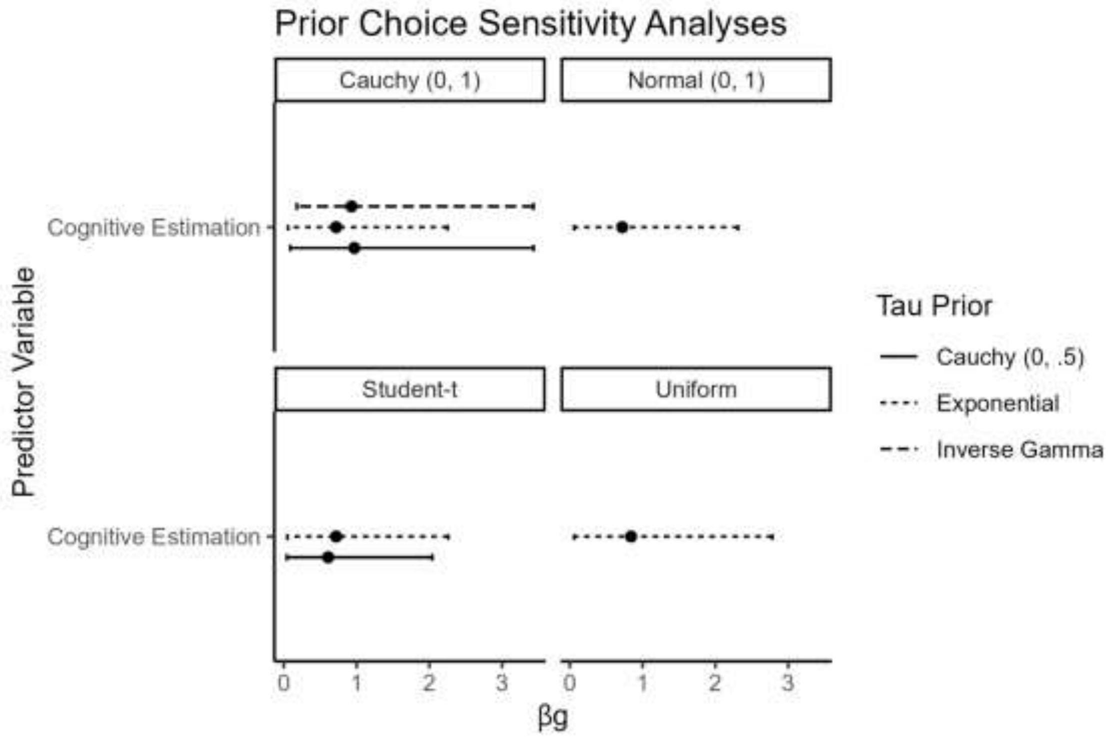

Figure 1. Study level standard deviation estimates with 95% confidence intervals for the Cognitive Estimation model. For tests included in the analysis see Supplementary Materials 1.

Global Measures of Executive Functioning

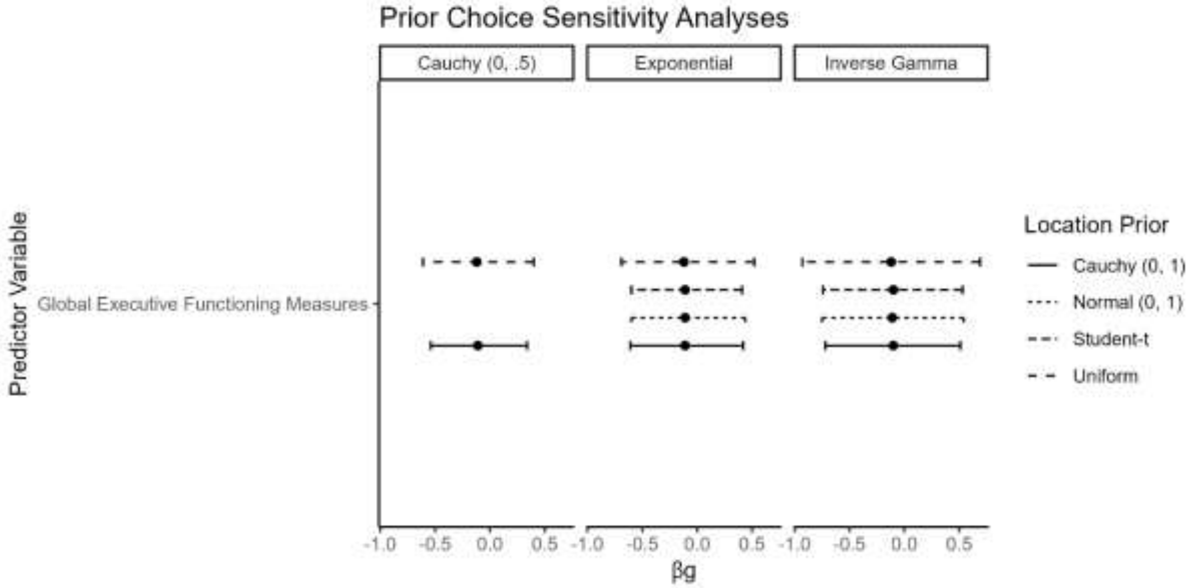

Figure 1. Regression coefficients with 95% confidence intervals for the Global Measures of Executive Functioning model. For tests included in the analysis see Supplementary Materials 1.

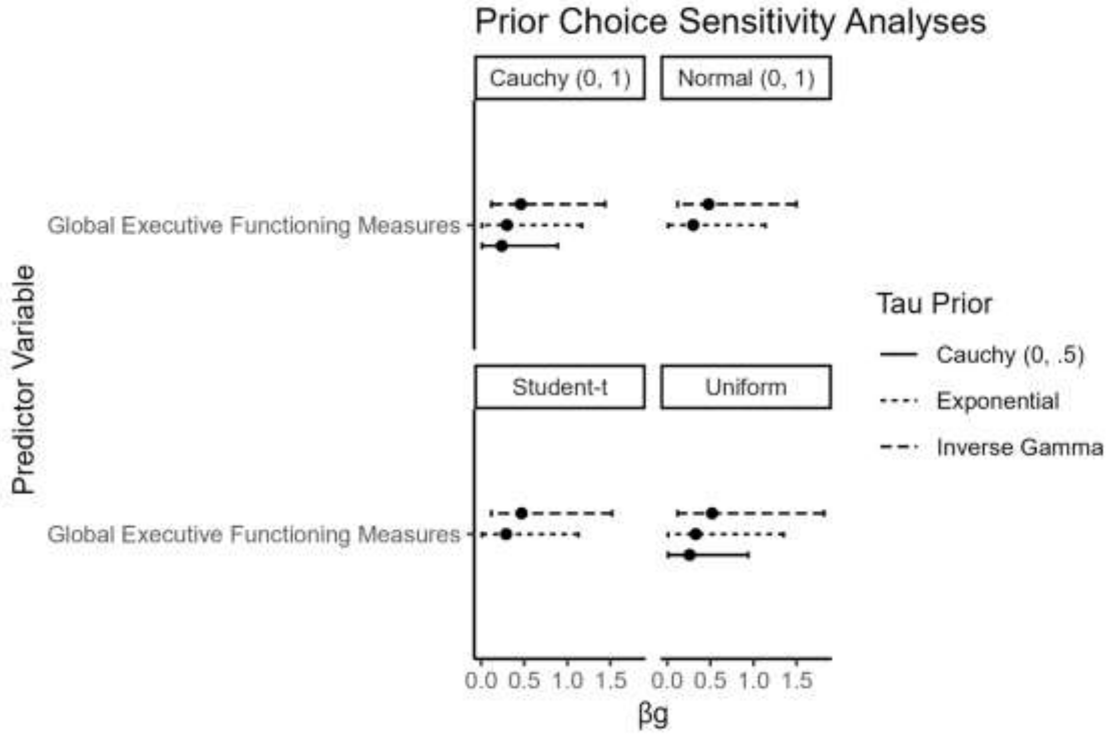

Figure 1. Study level standard deviation estimates with 95% confidence intervals for the Global Measures of Executive Functioning model. For tests included in the analysis see Supplementary Materials 1.

Set Maintenance

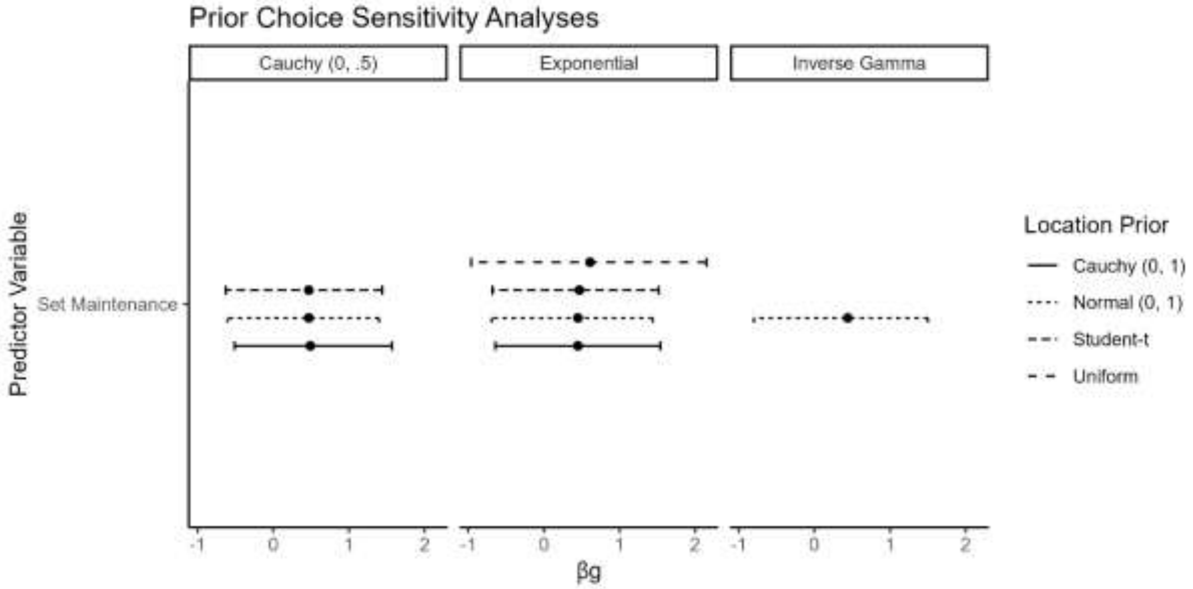

Figure 1. Regression coefficients with 95% confidence intervals for the Set Maintenance model. For tests and studies included in the analysis see Supplementary Materials 1.

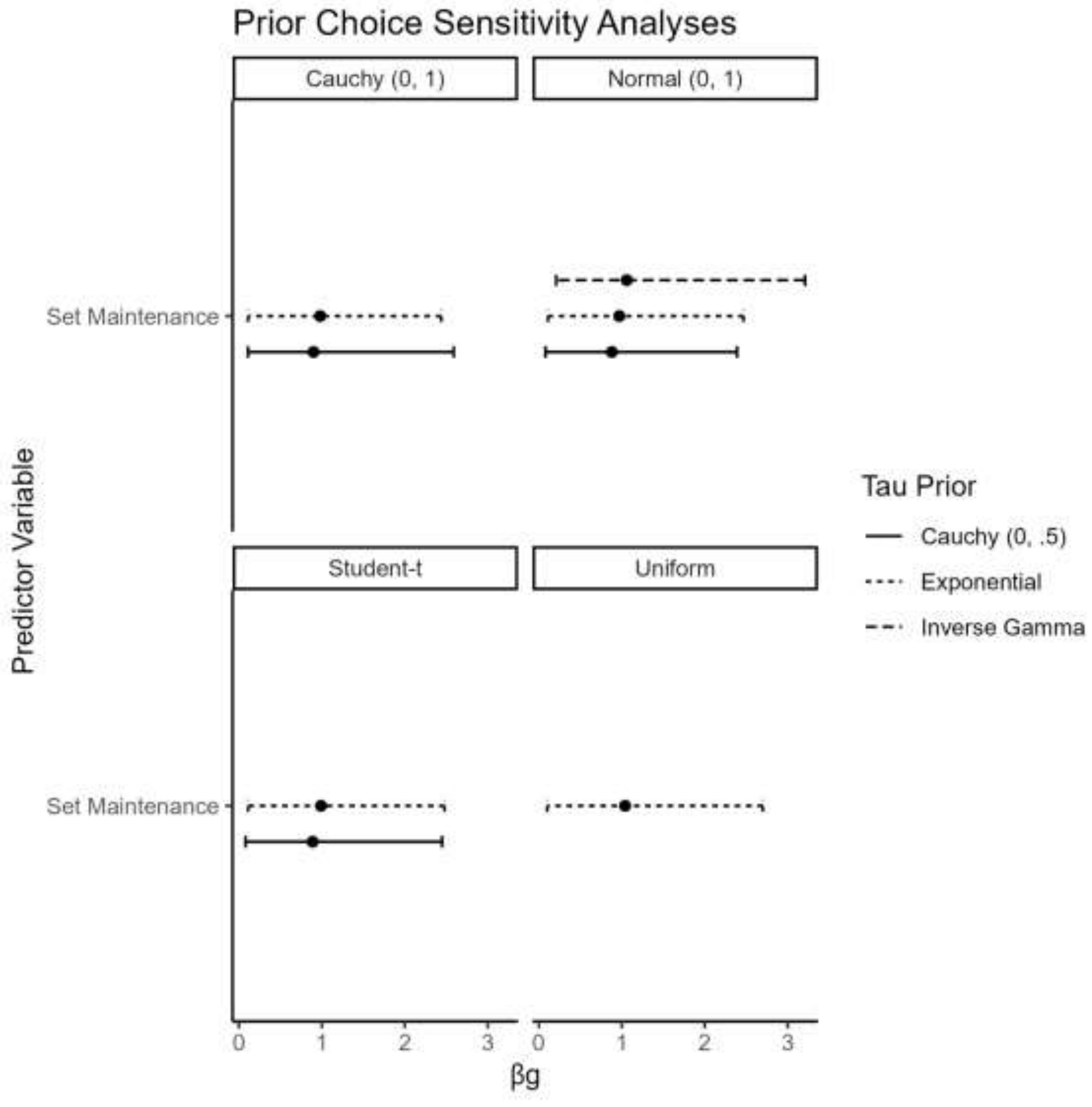

Figure 1. Study level standard deviation estimates with 95% confidence intervals for the Set Maintenance model. For tests and studies included in the analysis see Supplementary Materials 1.

Other Measures of Verbal Working Memory

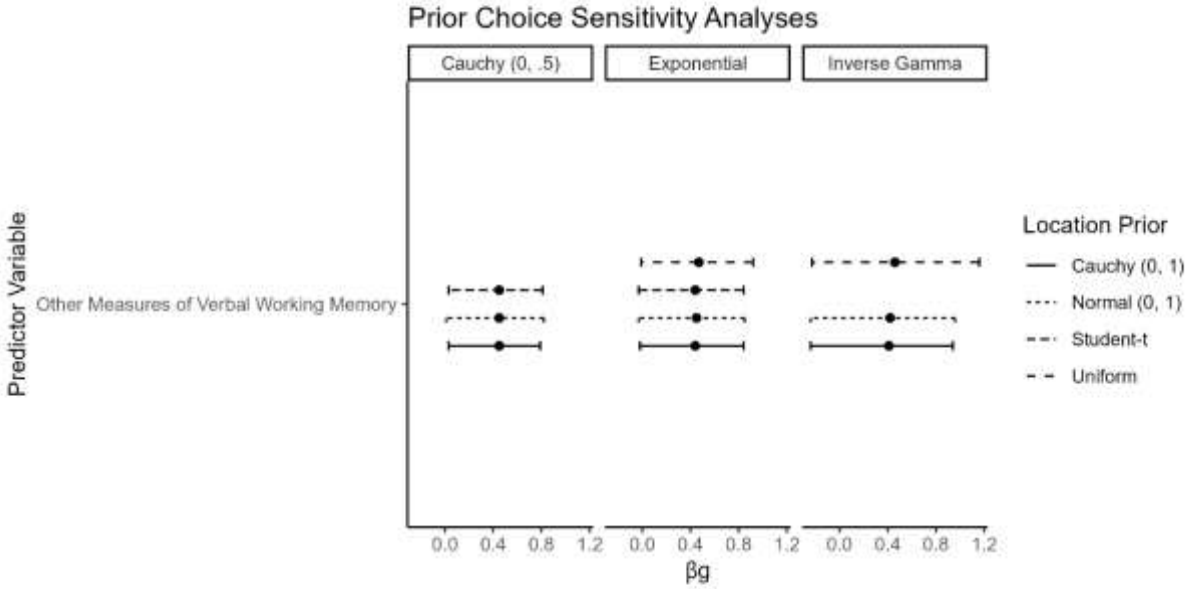

Figure 1. Regression coefficients with 95% confidence intervals for the Other Measures of Verbal Working Memory model. For tests included in the analysis see Supplementary Materials 1.

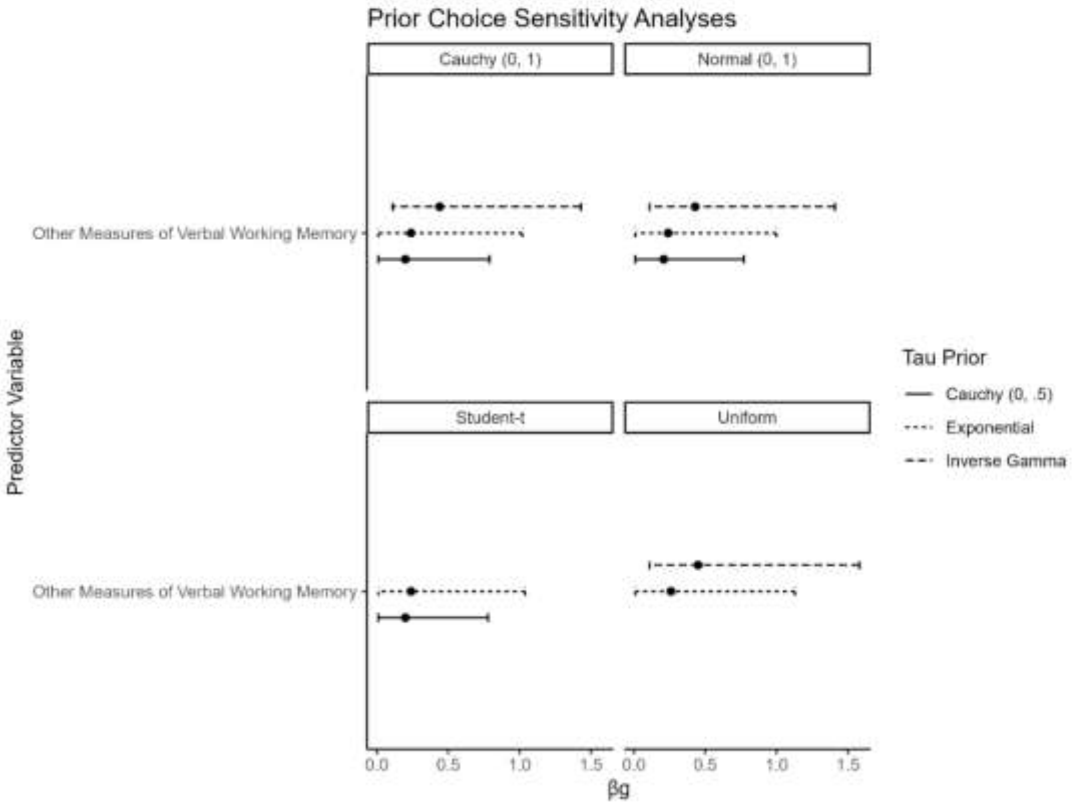

Figure 1. Study level standard deviation estimates with 95% confidence intervals for the Other Measures of Verbal Working Memory model. For tests included in the analysis see Supplementary Materials 1.

Other Measures of Visual Working Memory

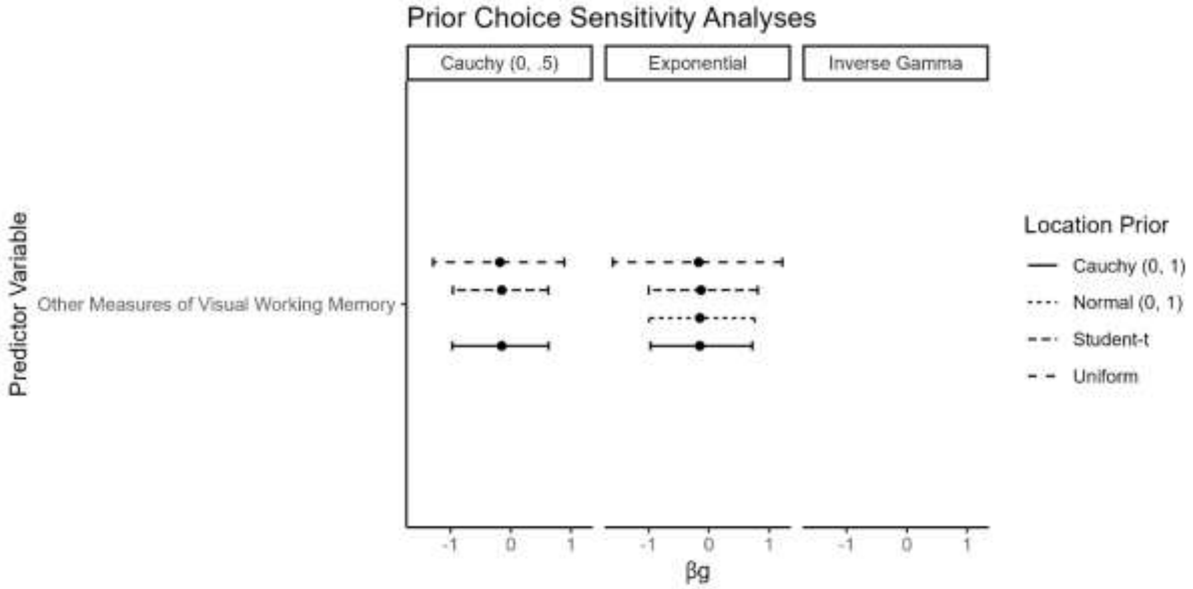

Figure 1. Regression coefficients with 95% confidence intervals for the Other Measures of Visual Working Memory model. For tests and studies included in the analysis see Supplementary Materials 1.

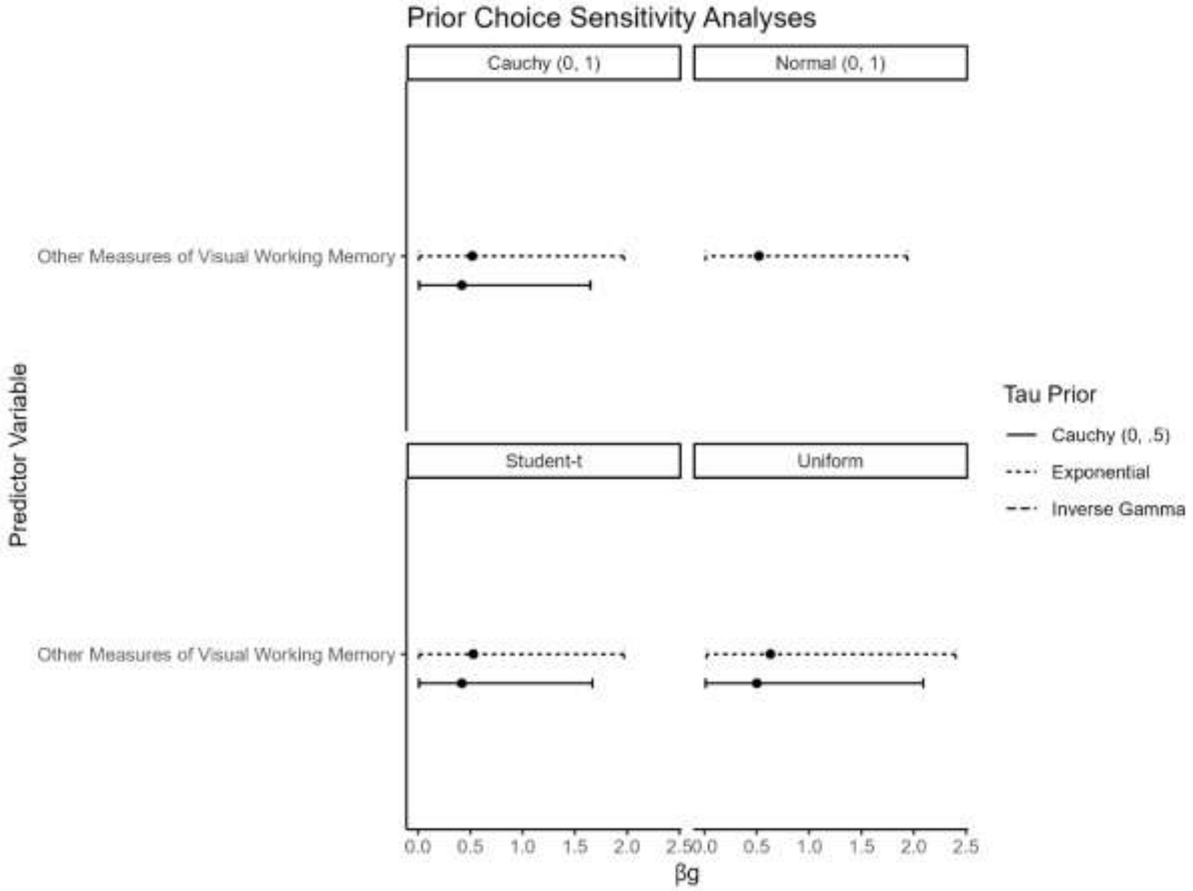

Figure 1. Study level standard deviation estimates with 95% confidence intervals for the Other Measures of Visual Working Memory model. For tests and studies included in the analysis see Supplementary Materials 1.

Sequencing

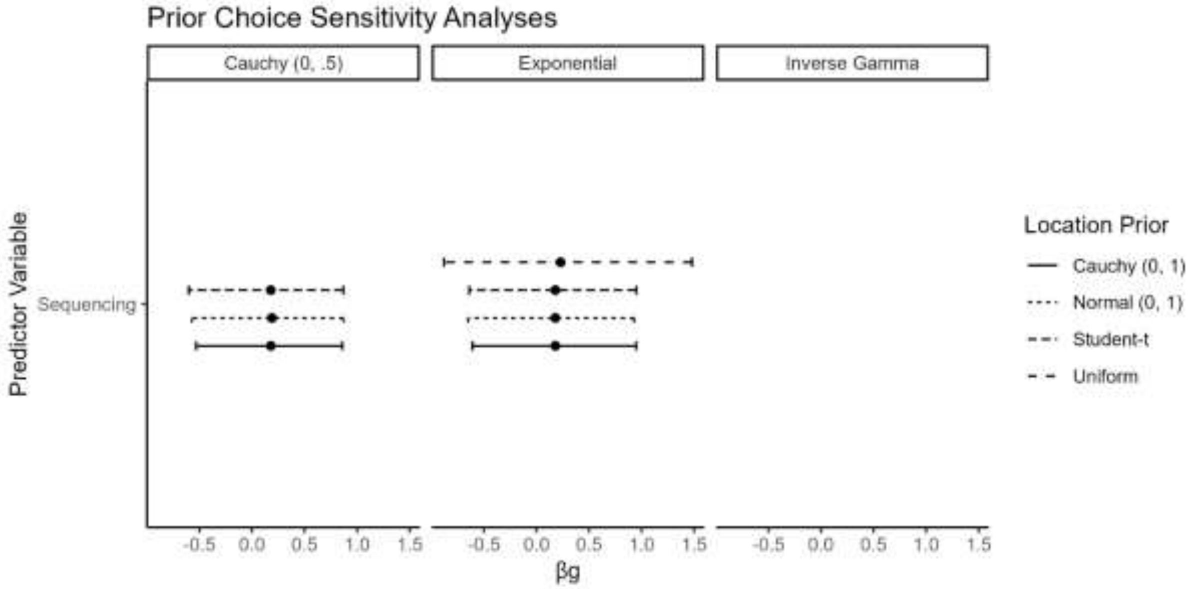

Figure 1. Regression coefficients with 95% confidence intervals for the Sequencing model. For tests and studies included in the analysis see Supplementary Materials 1.

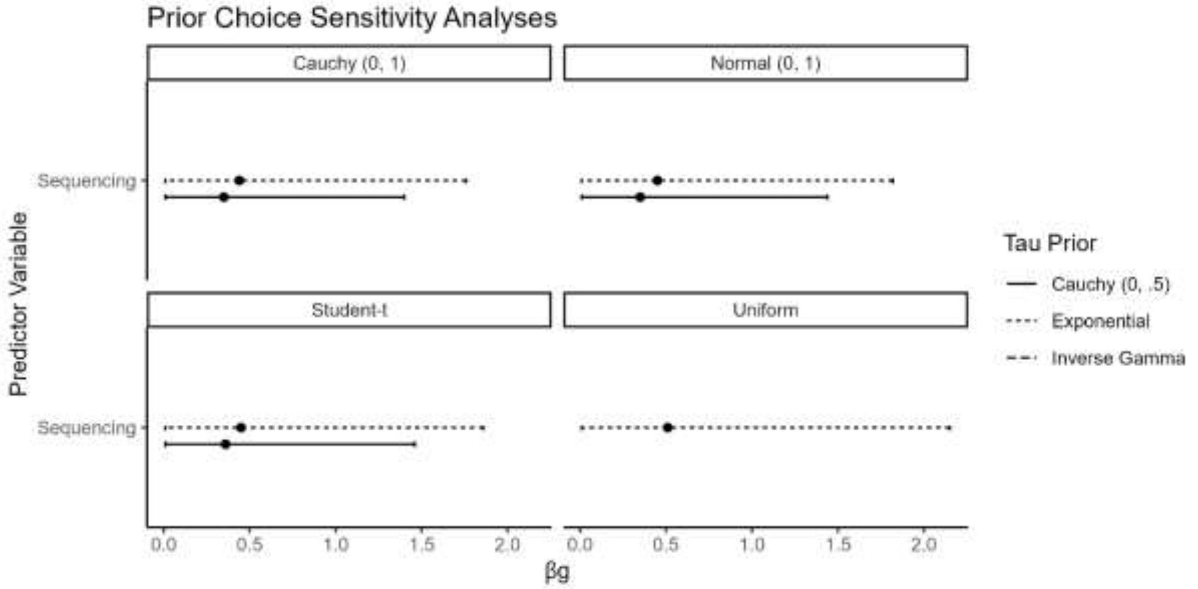

Figure 1. Study level standard deviation estimates with 95% confidence intervals for the Sequencing model. For tests and studies included in the analysis see Supplementary Materials 1.

Benton Visual Retention Test

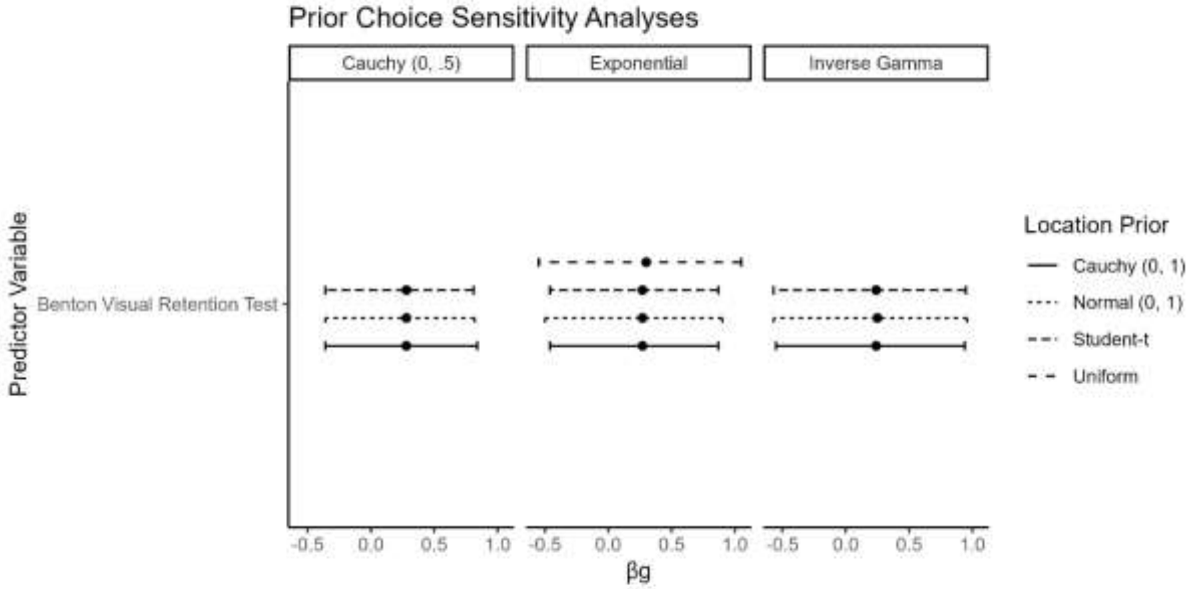

Figure 1. Regression coefficients with 95% confidence intervals for the Benton Visual Retention Test model.

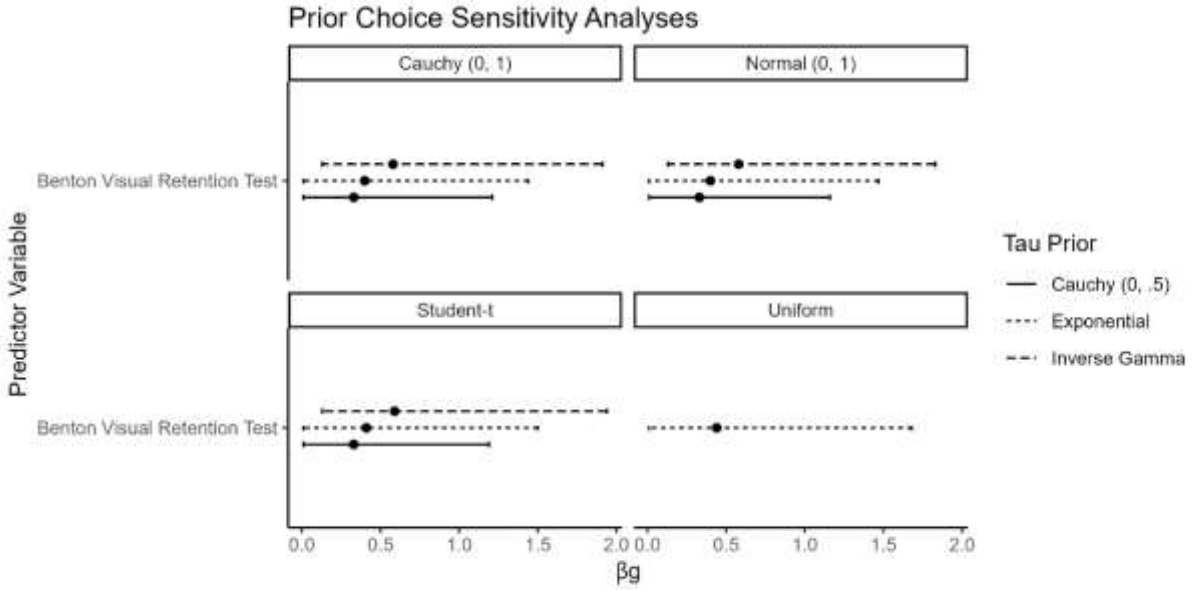

Figure 1. Study level standard deviation estimates with 95% confidence intervals for the Benton Visual Retention Test model.

Quality Sensitivity Analysis

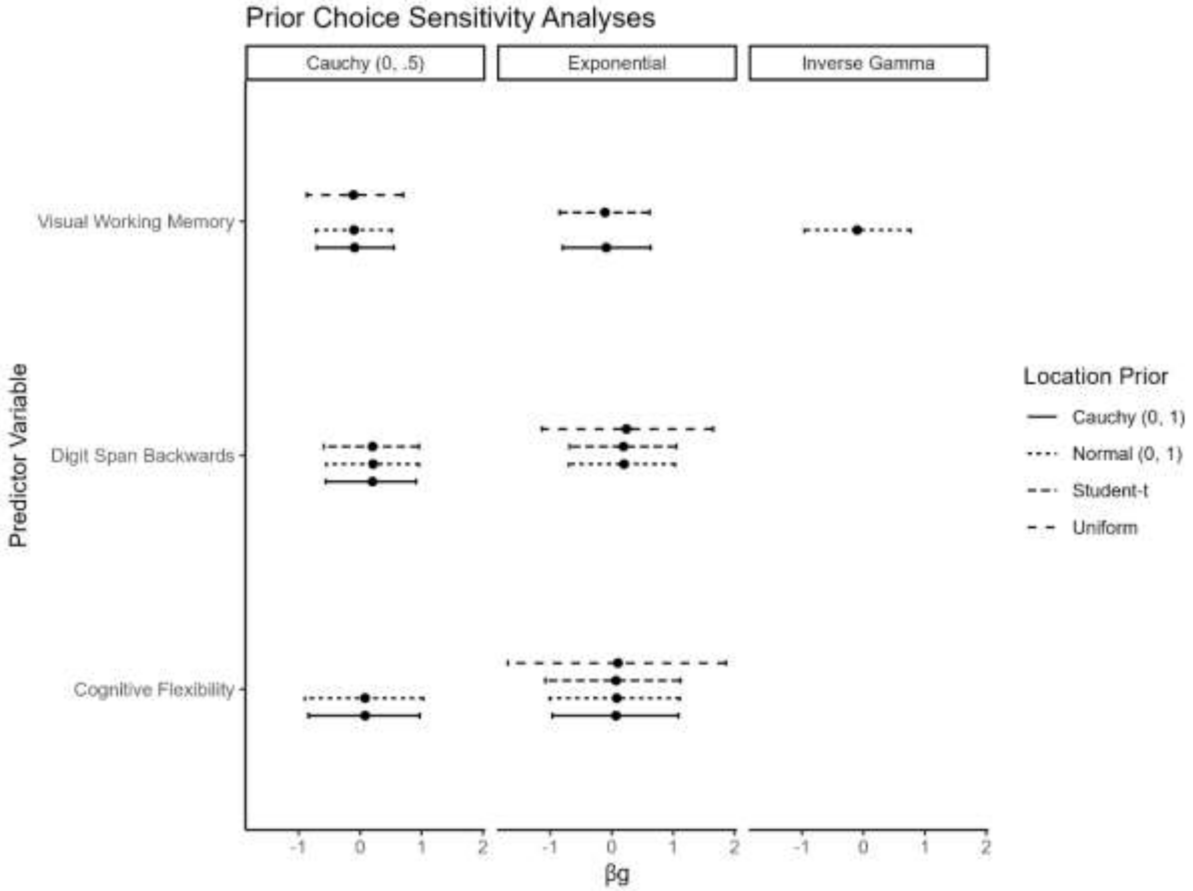

Figure 1. Regression coefficients with 95% confidence intervals for the study quality sensitivity analyses for measures of visual working memory, digit span backwards and cognitive flexibility. For tests and studies included in the analysis see Supplementary Materials 1.

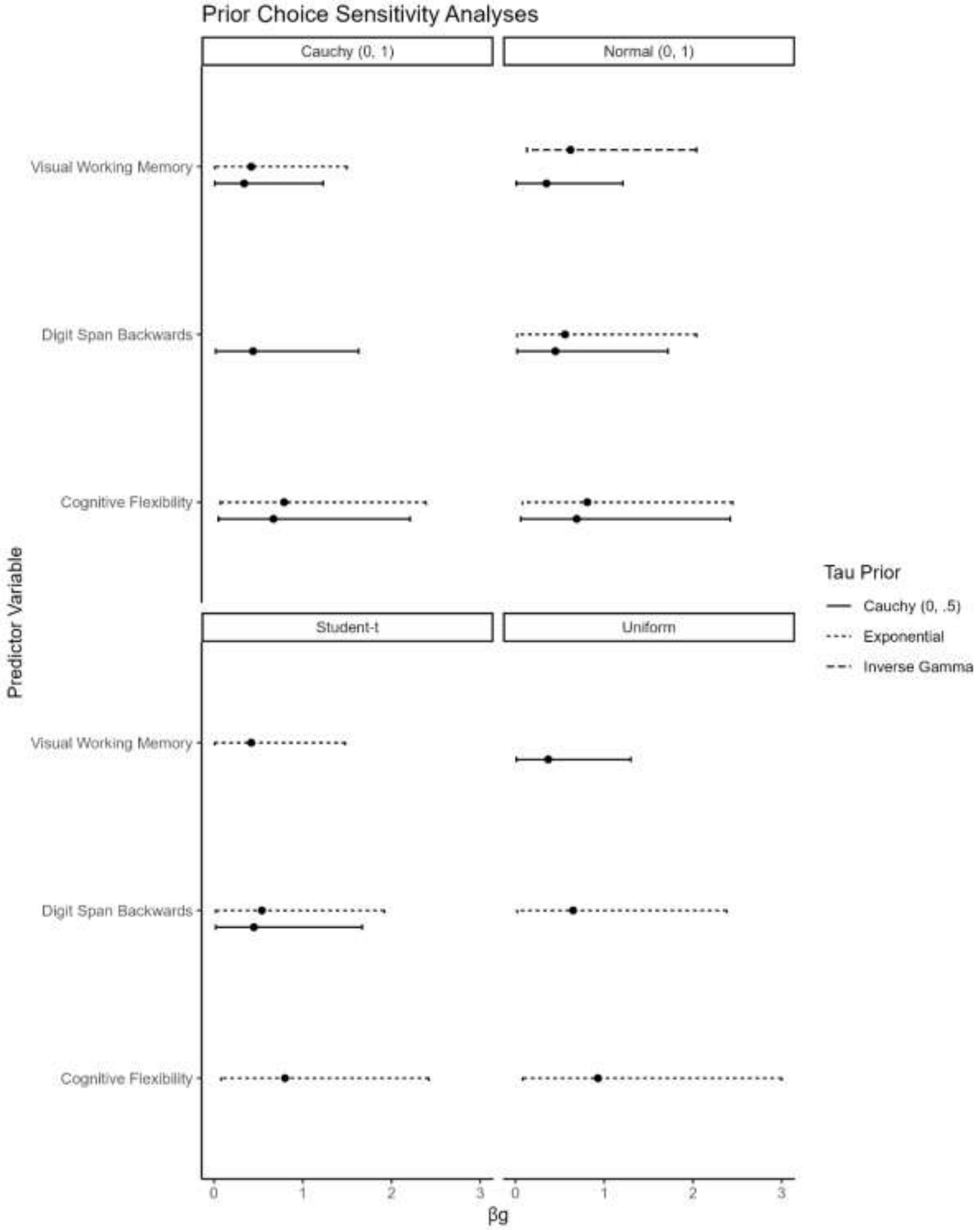

Figure 1. Study level standard deviation estiamtes with 95% confidence intervals for the study quality sensitivity analyses for measures of visual working memory, digit span backwards and cognitive flexibility. For tests and studies included in the analysis see Supplementary Materials 1.

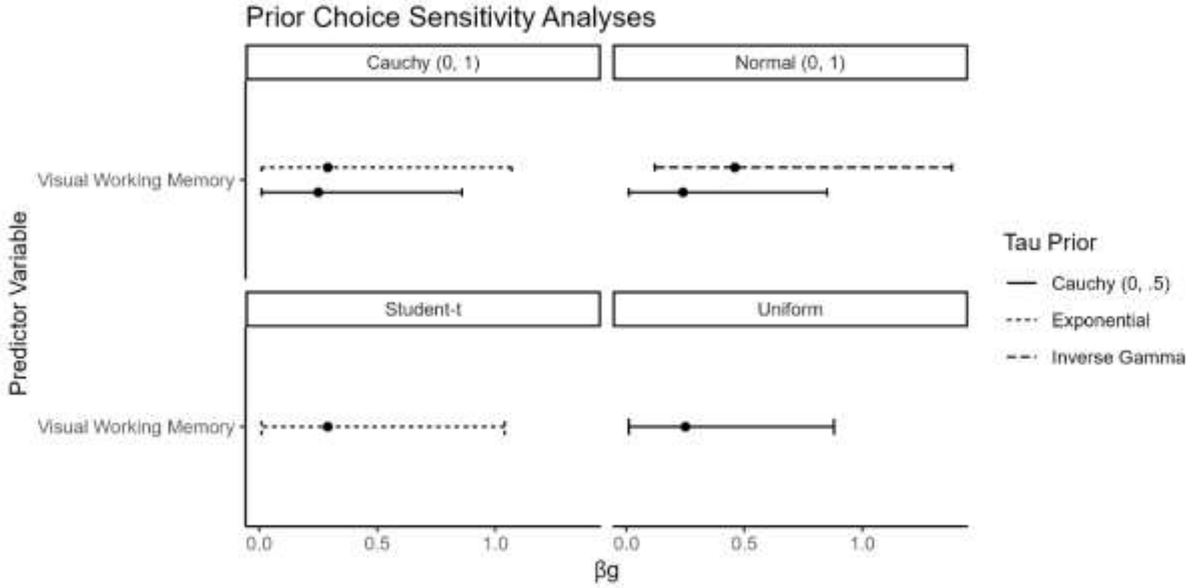

Figure 1. Effect size standard deviation estimates with 95% confidence intervals for the study quality sensitivity analyses for measures of visual working memory, digit span backwards and cognitive flexibility. For tests and studies included in the analysis see Supplementary Materials 1.

Memory

Wechsler Memory Scale: Verbal Episodic Memory

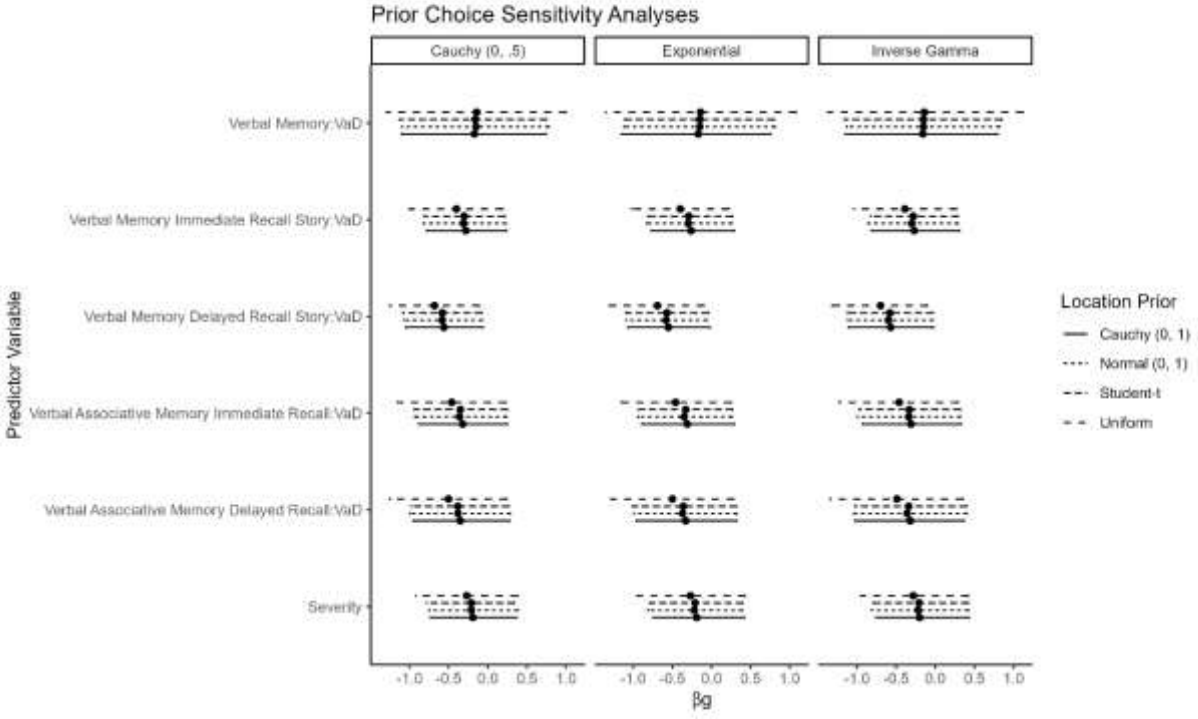

Figure 1. Regression coefficients with 95% confidence intervals for the Wechsler Memory Scale: Verbal Episodic Memory Subtests model. VaD: vascular dementia, Severity: difference in dementia severity between dementia groups.

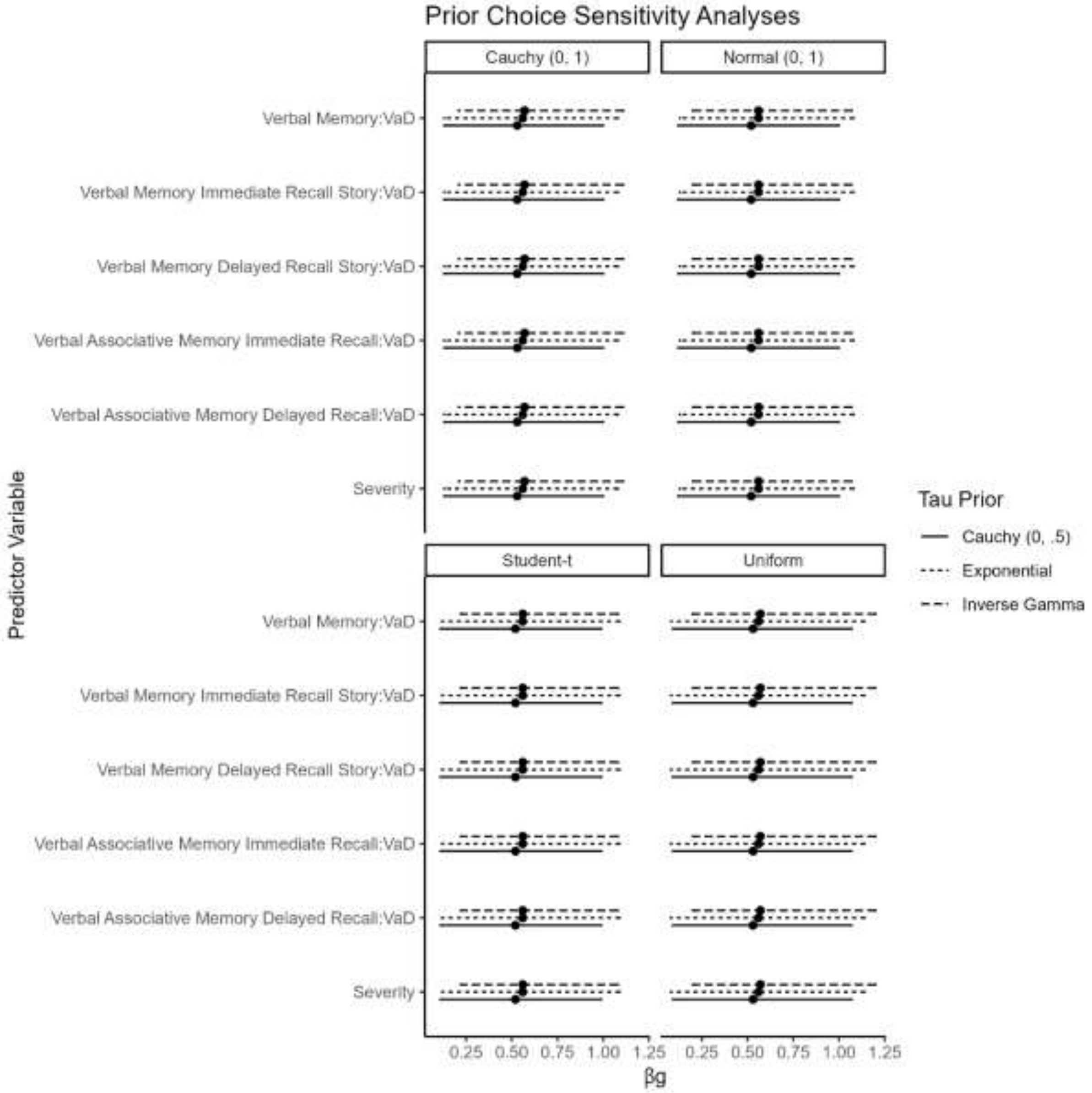

Figure 1. Study level standard deviation estimates with 95% confidence intervals for the Wechsler Memory Scale: Verbal Episodic Memory Subtests model. VaD: vascular dementia, Severity: difference in dementia severity between dementia groups.

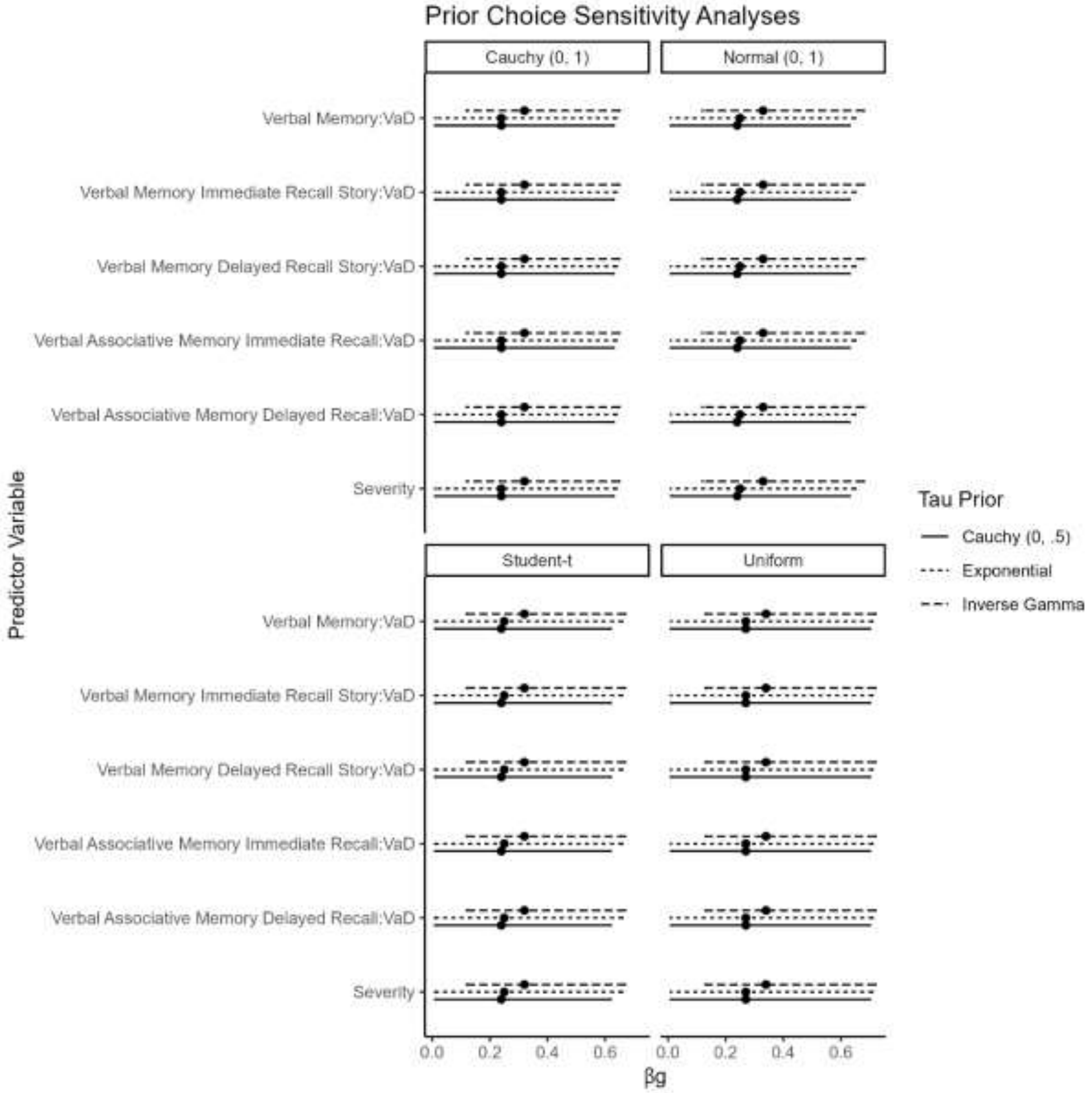

Figure 1. Effect size level standard deviation estimates with 95% confidence intervals for the Wechsler Memory Scale: Verbal Episodic Memory Subtests model. VaD: vascular dementia, Severity: difference in dementia severity between dementia groups.

Rey's Auditory Verbal Learning Test

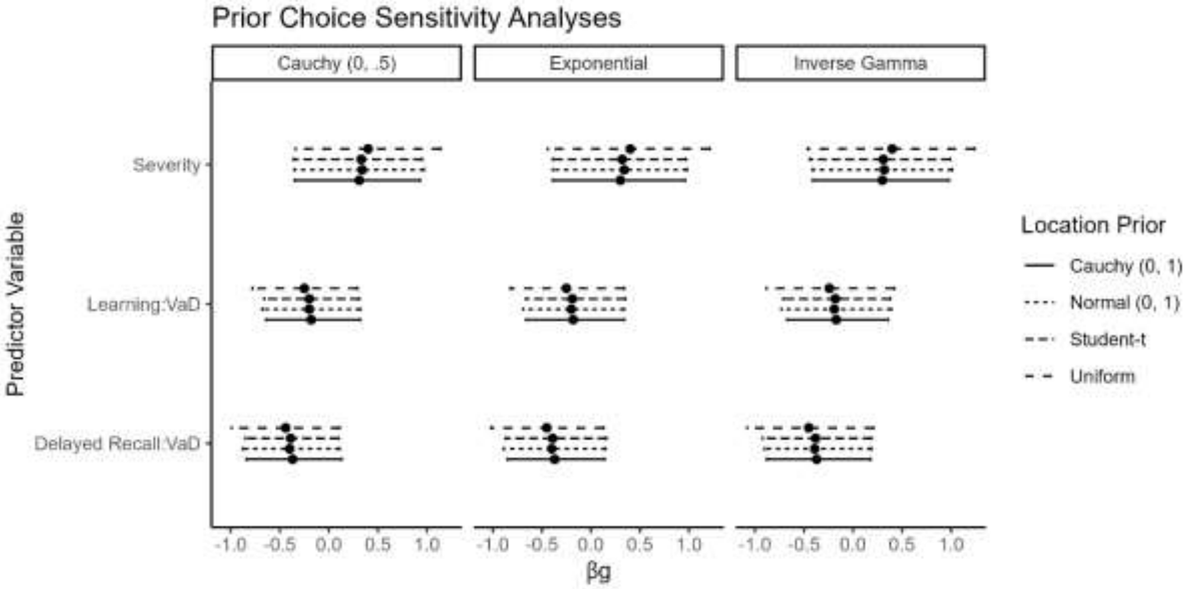

Figure 1. Regression coefficients with 95% confidence intervals for the Rey's Auditory Verbal Learning Test Learning and Delayed Recall model. VaD: vascular dementia, Severity: difference in dementia severity between dementia groups.

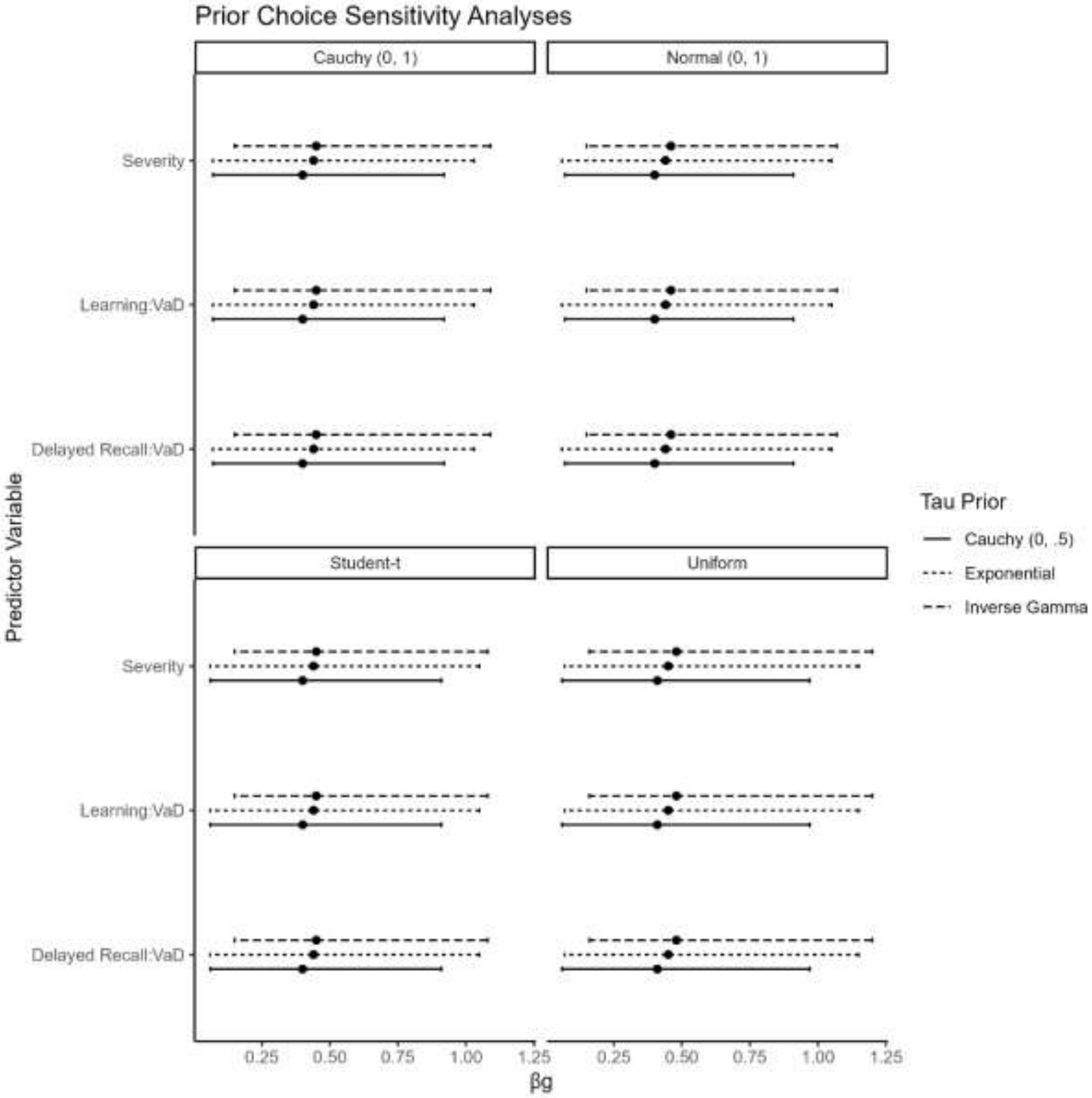

Figure 1. Study level standard deviation estimates with 95% confidence intervals for the Rey's Auditory Verbal Learning Test Learning and Delayed Recall model. VaD: vascular dementia, Severity: difference in dementia severity between dementia groups.

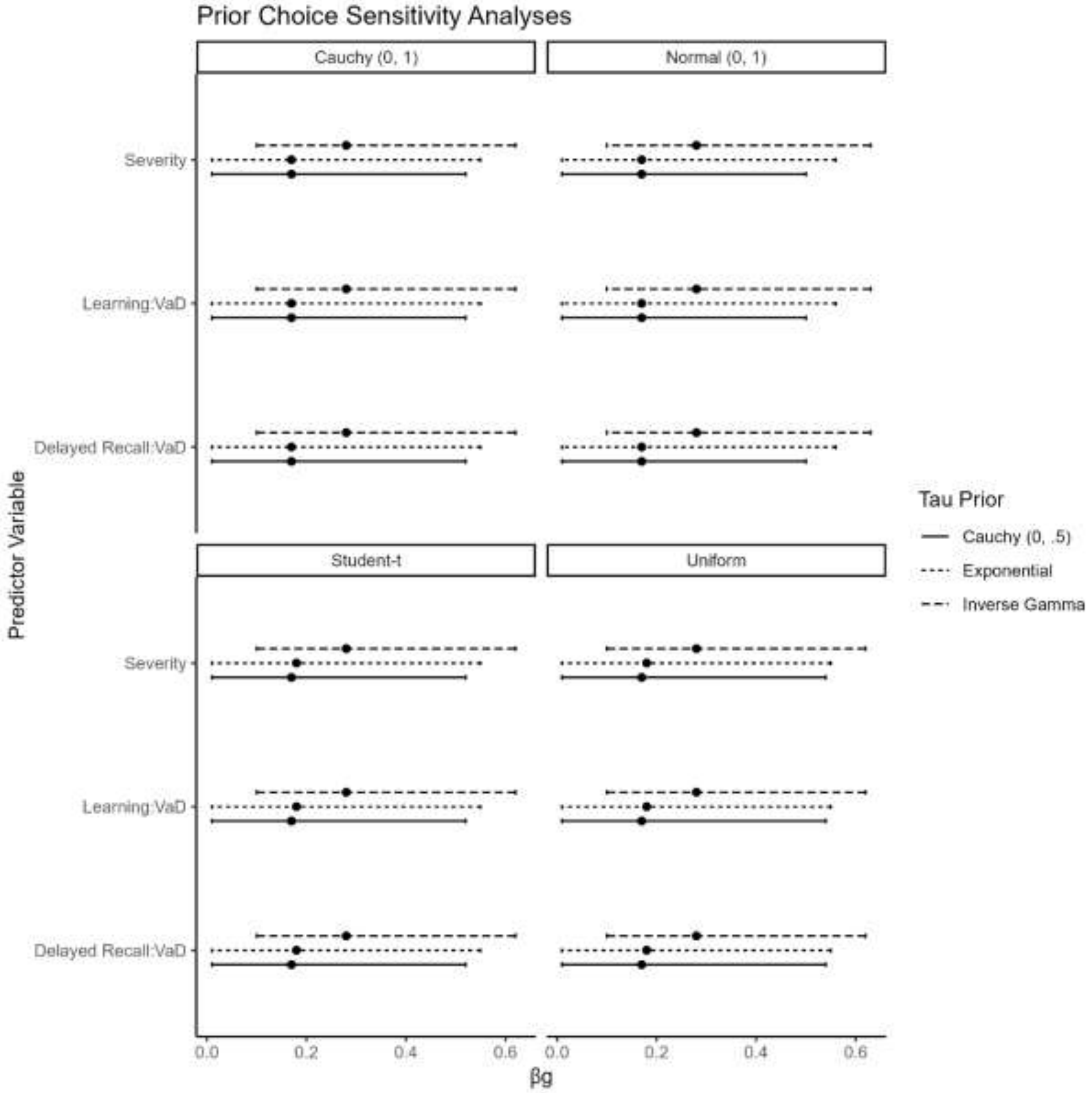

Figure 1. Effect size level standard deviation estimates with 95% confidence intervals for the Rey's Auditory Verbal Learning Test Learning and Delayed Recall model. VaD: vascular dementia, Severity: difference in dementia severity between dementia groups.

California Verbal Learning Test

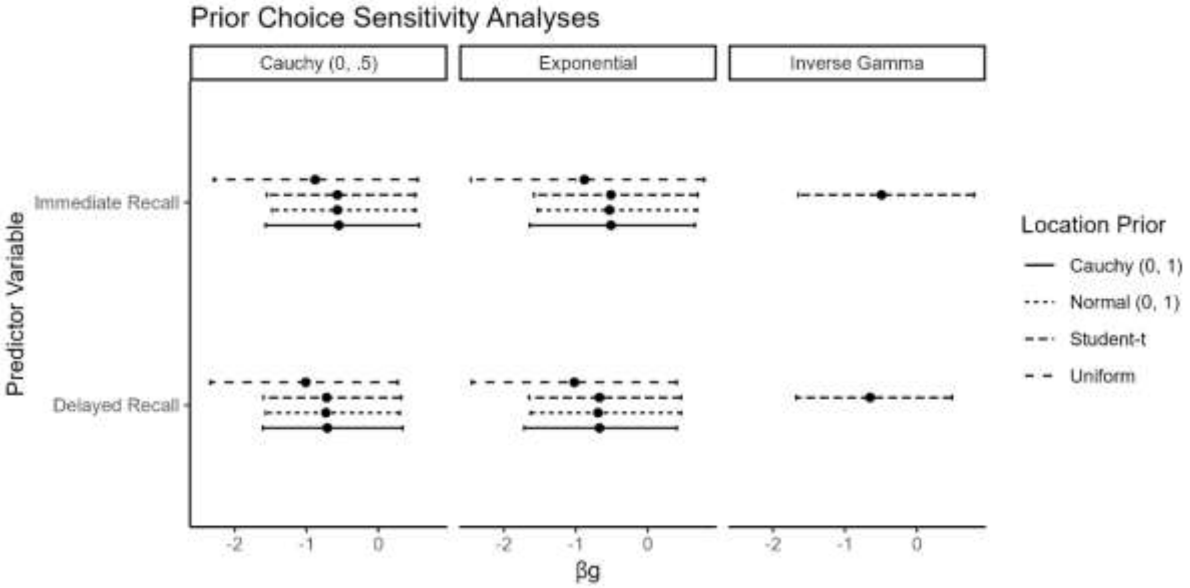

Figure 1. Regression coefficients with 95% confidence intervals for the California Verbal Learning Test Immediate and Delayed Free Recall model.

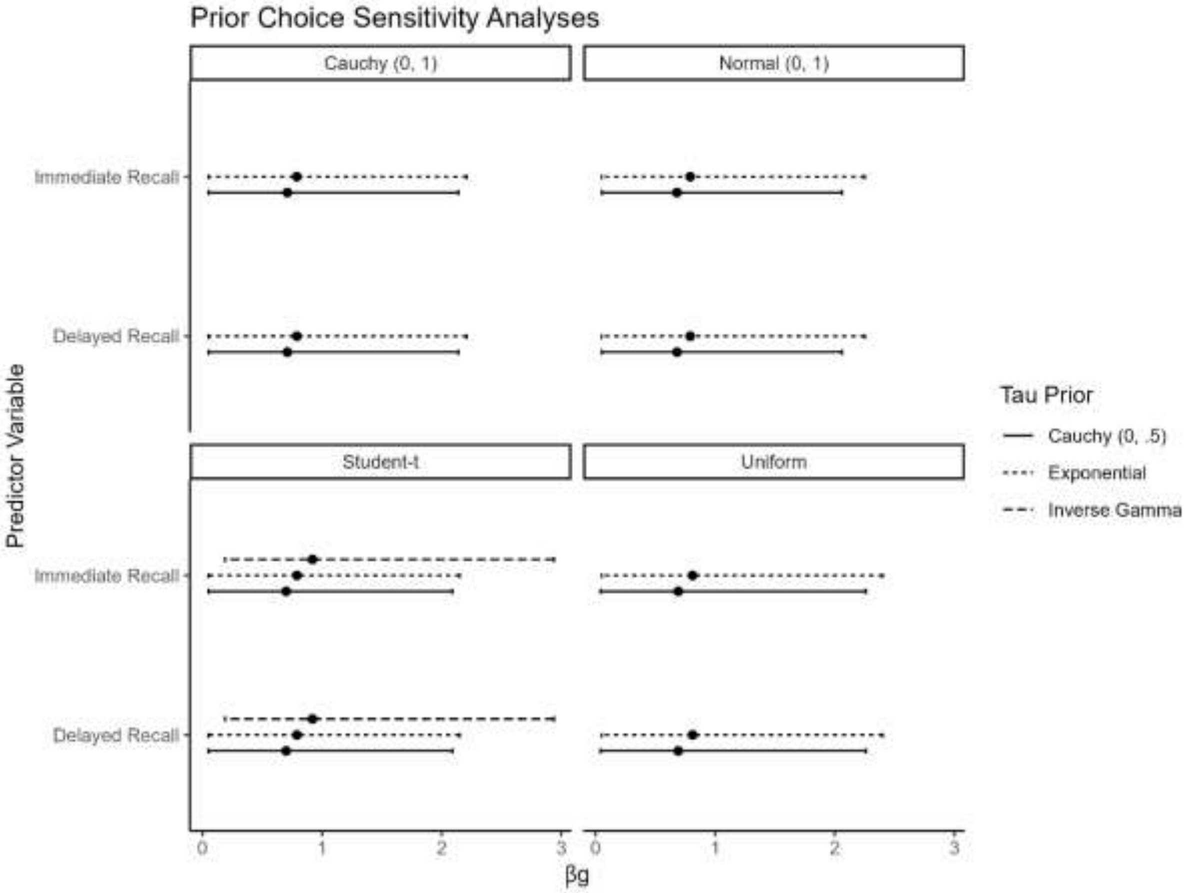

Figure 1. Study level standard deviation estimates with 95% confidence intervals for the California Verbal Learning Test Immediate and Delayed Free Recall model.

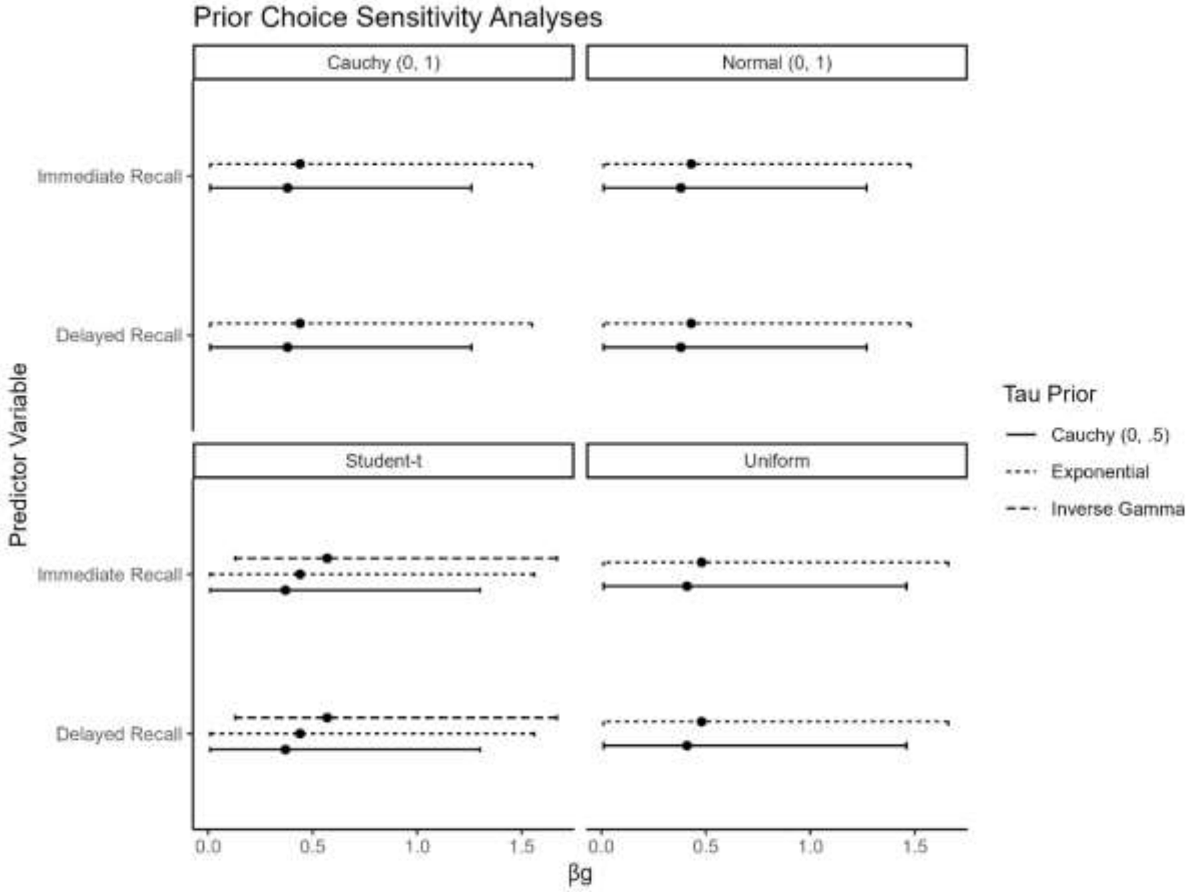

Figure 1. Effect size level standard deviation estimates with 95% confidence intervals for the California Verbal Learning Test Immediate and Delayed Free Recall model.

CERAD Word List

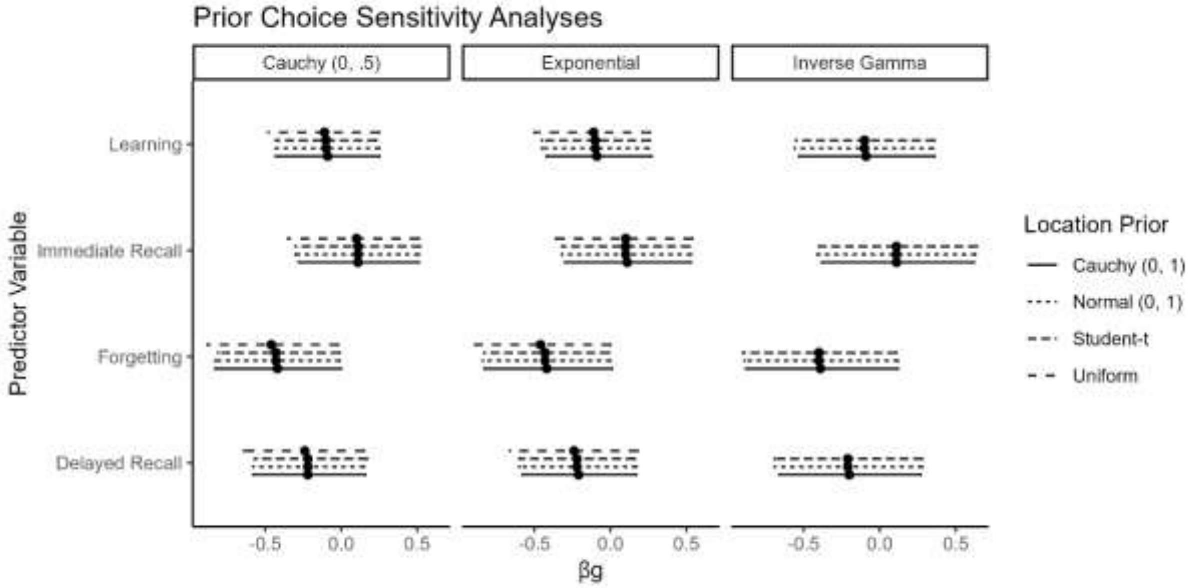

Figure 1. Regression coefficients with 95% confidence intervals for the Consortium to Establish a Registry for Alzheimer's Disease (CERAD) Word List Recall Measures model.

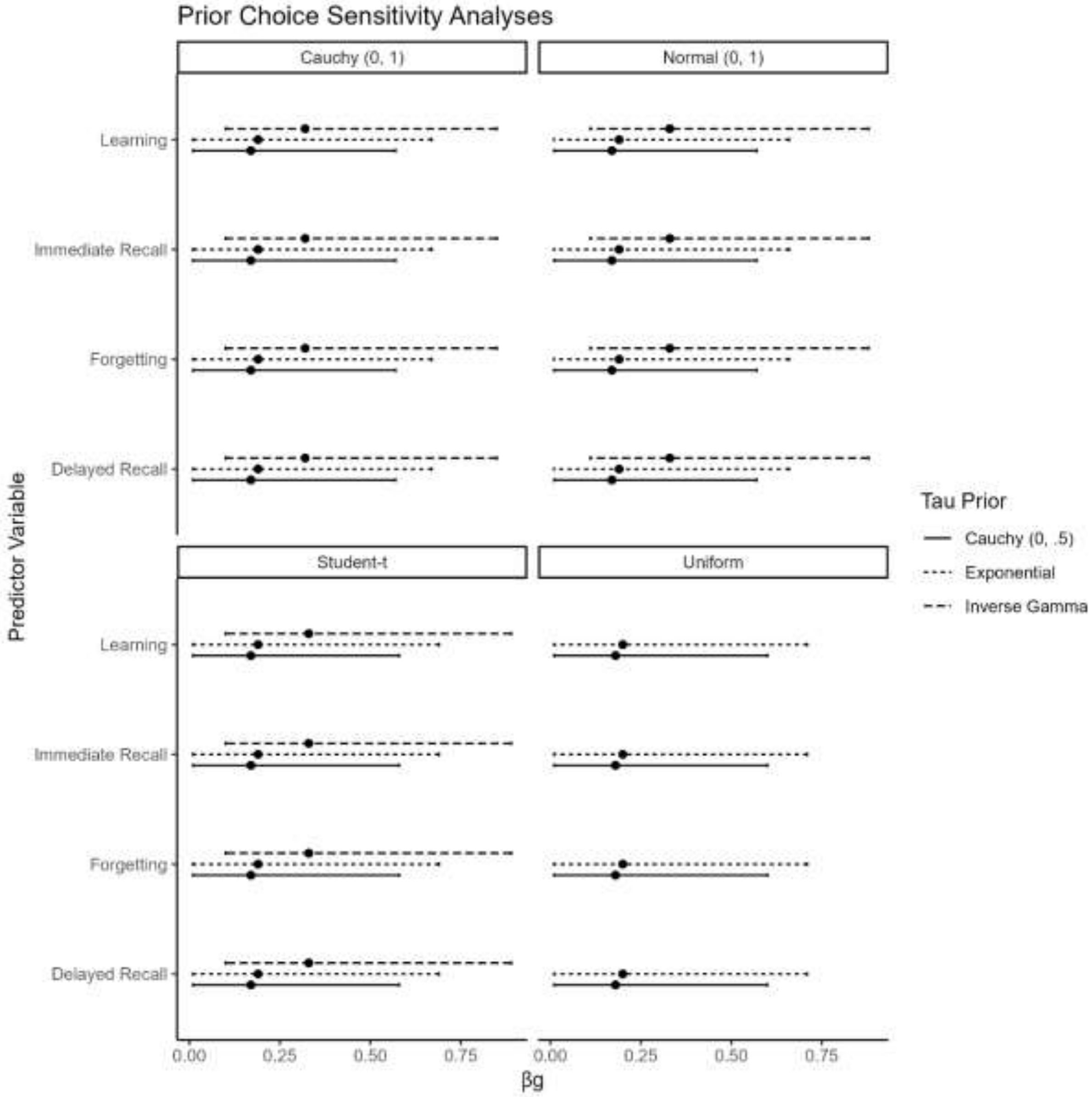

Figure 1. Study level standard deviation estimates with 95% confidence intervals for the Consortium to Establish a Registry for Alzheimer's Disease (CERAD) Word List Recall Measures model.

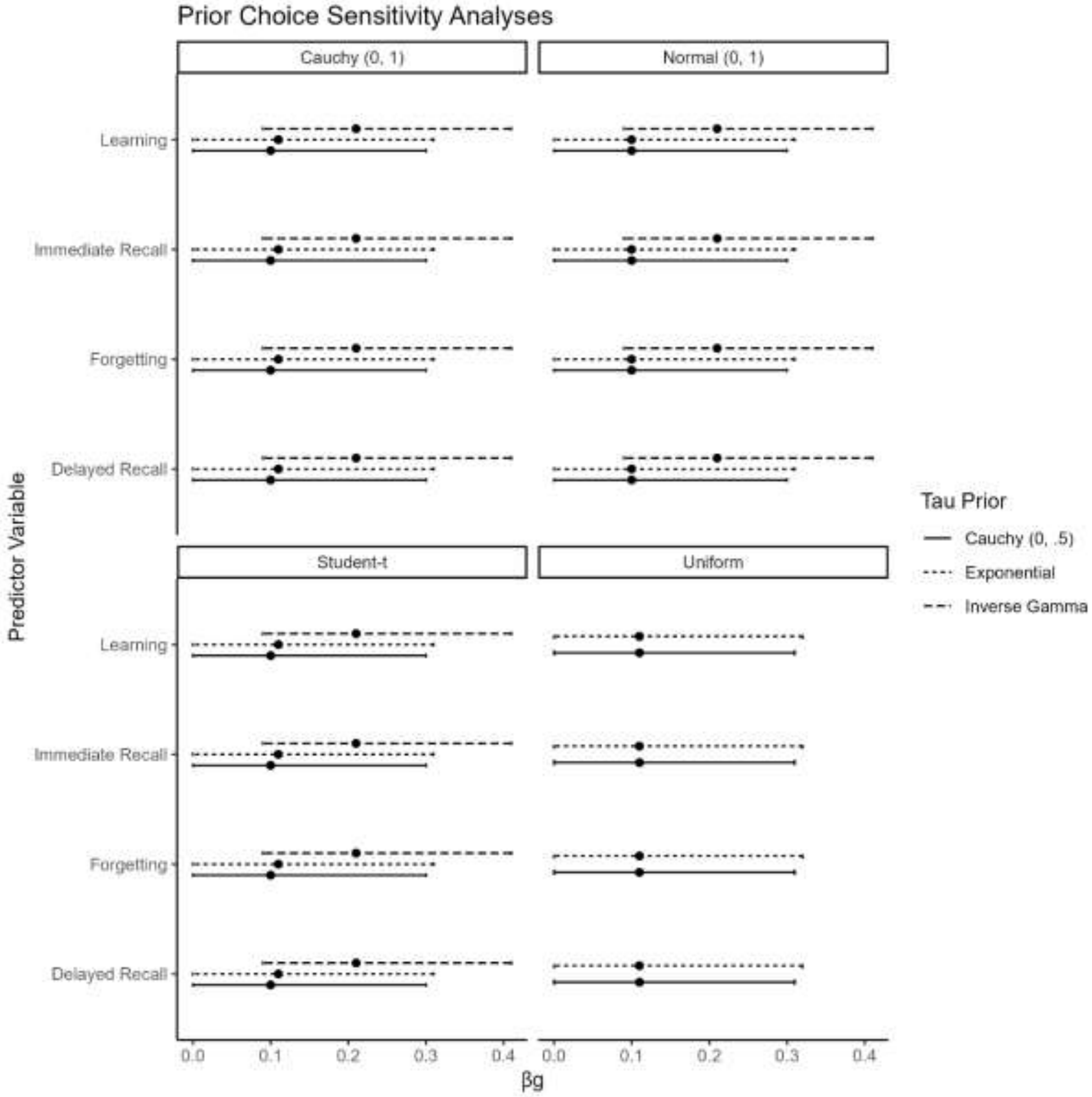

Figure 1. Effect size level standard deviation estimates with 95% confidence intervals for the Consortium to Establish a Registry for Alzheimer's Disease (CERAD) Word List Recall Measures model.

Hopkin's Verbal Learning Test: Learning

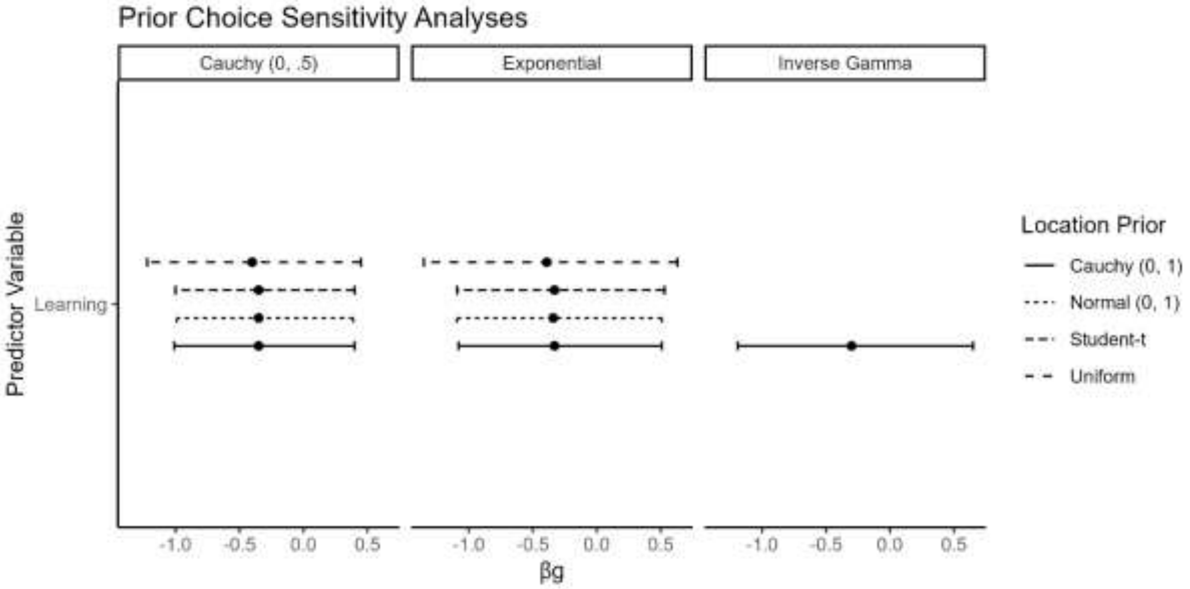

Figure 1. Regression coefficients with 95% confidence intervals for the Hopkin's Verbal Learning Test: Learning model.

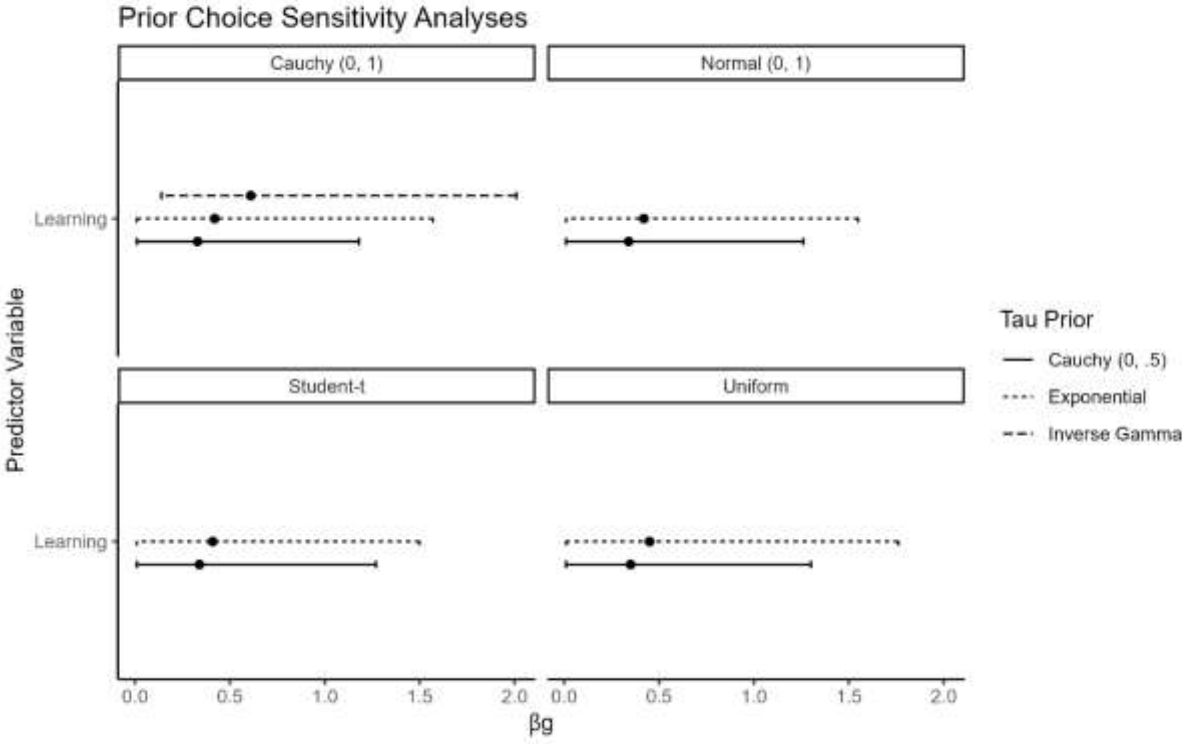

Figure 1. Study level standard deviation estimates with 95% confidence intervals for the Hopkin's Verbal Learning Test: Learning model.

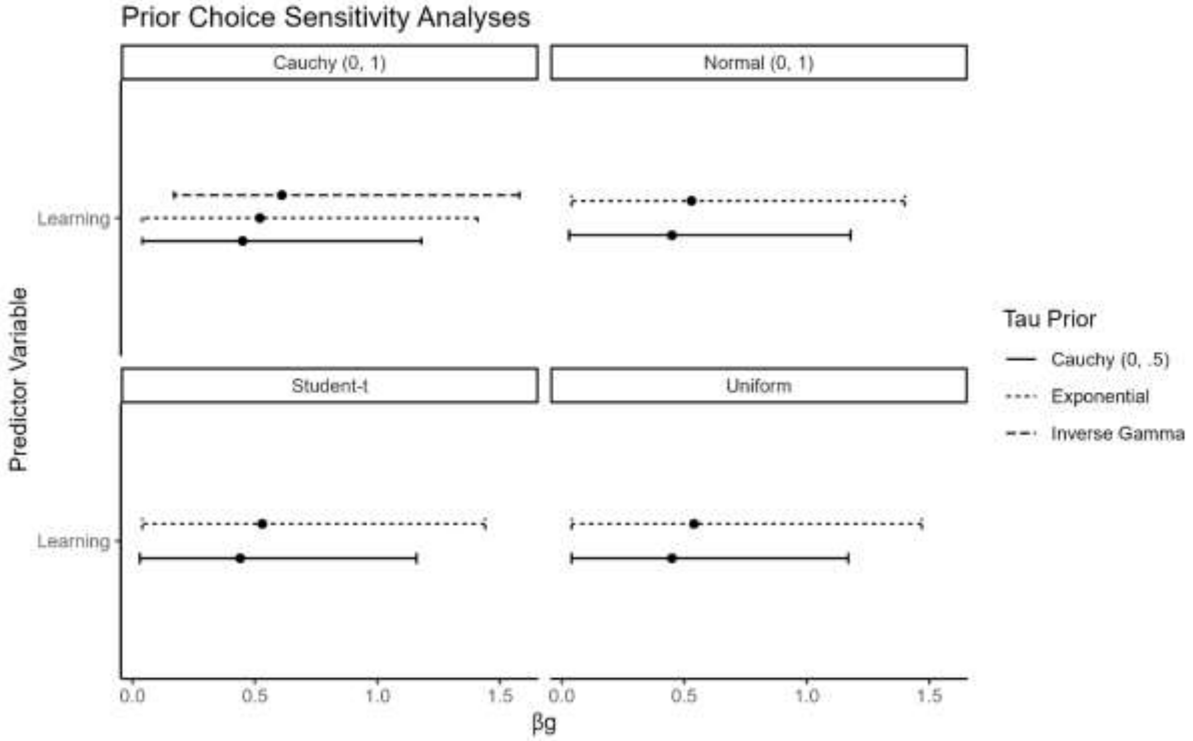

Figure 1. Effect size level standard deviation estimates with 95% confidence intervals for the Hopkin's Verbal Learning Test: Learning model.

Addenbrooke's Cognitive Examination

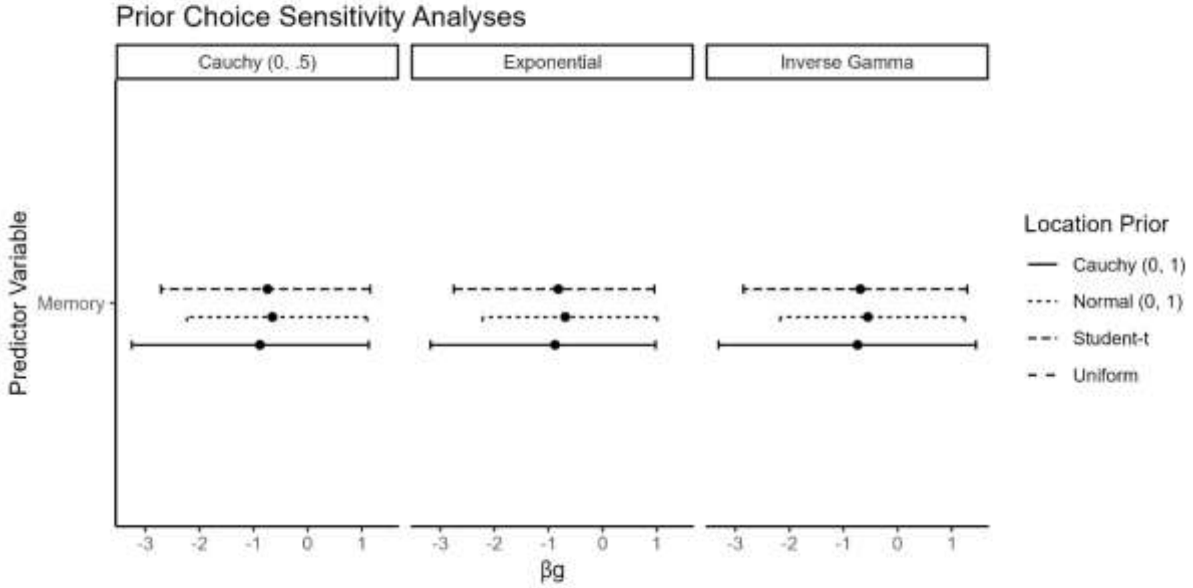

Figure 1. Regression coefficients with 95% confidence intervals for the Addenbrooke's Cognitive Examination: Memory subscale model.

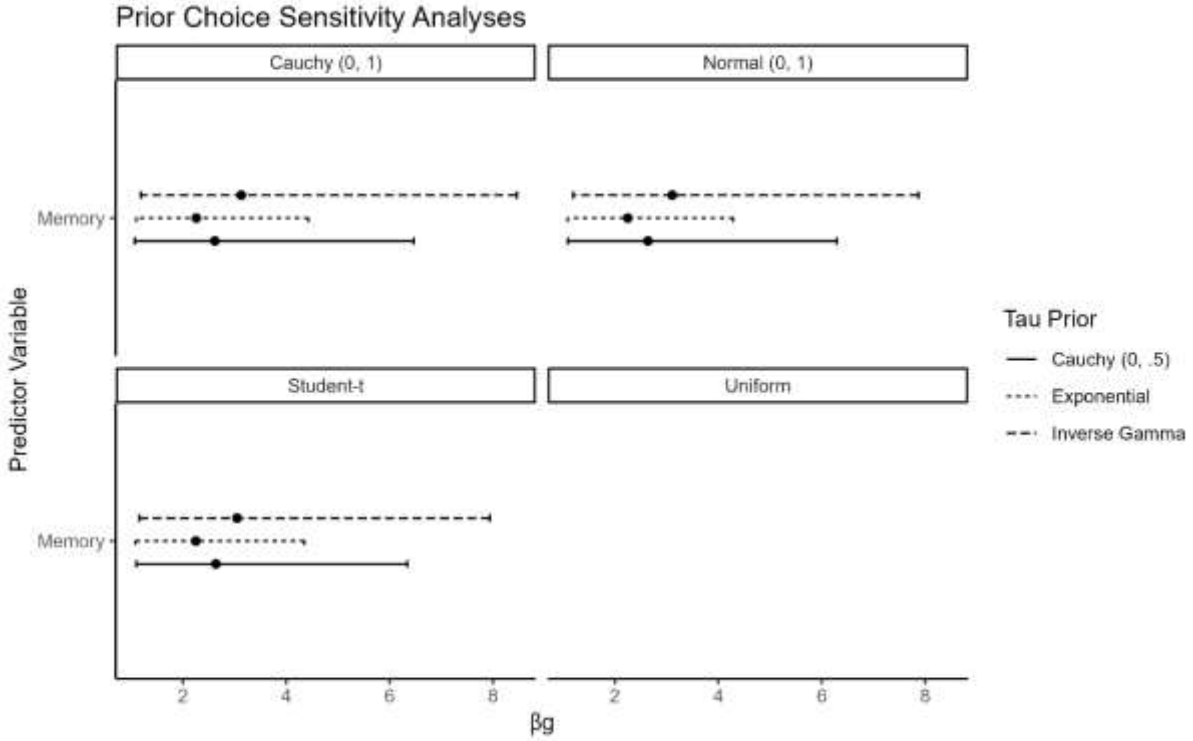

Figure 1. Study level standard deviation estimates with 95% confidence intervals for the Addenbrooke's Cognitive Examination: Memory subscale model.

Fuld Object Memory Examination: Learning

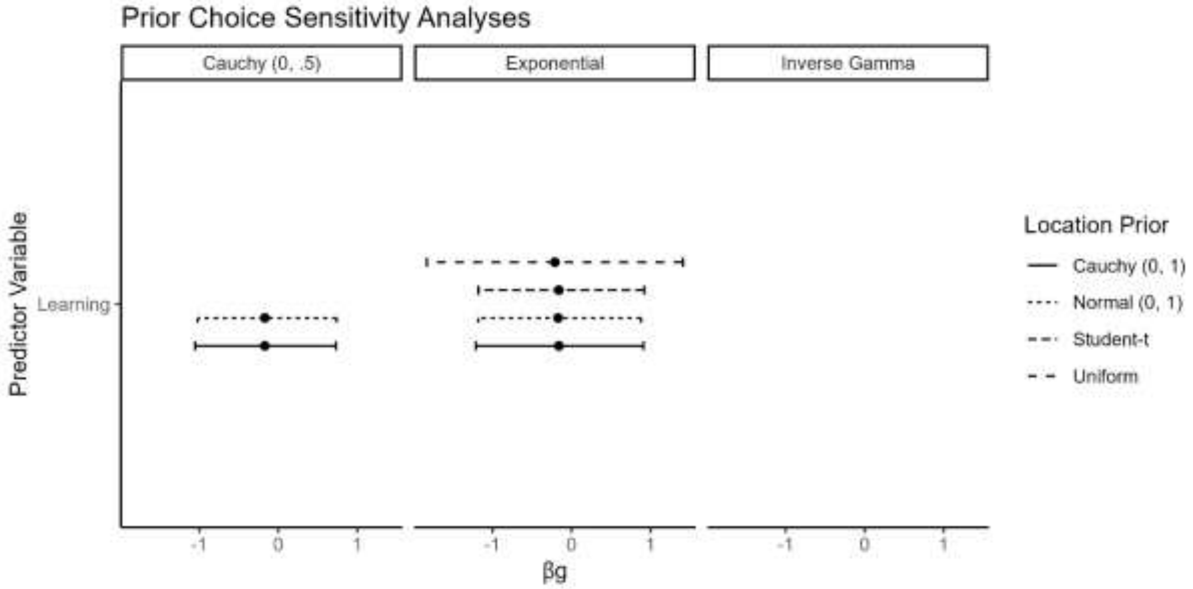

Figure 1. Regression coefficients with 95% confidence intervals for the Fuld Object Memory Examination: Learning model.

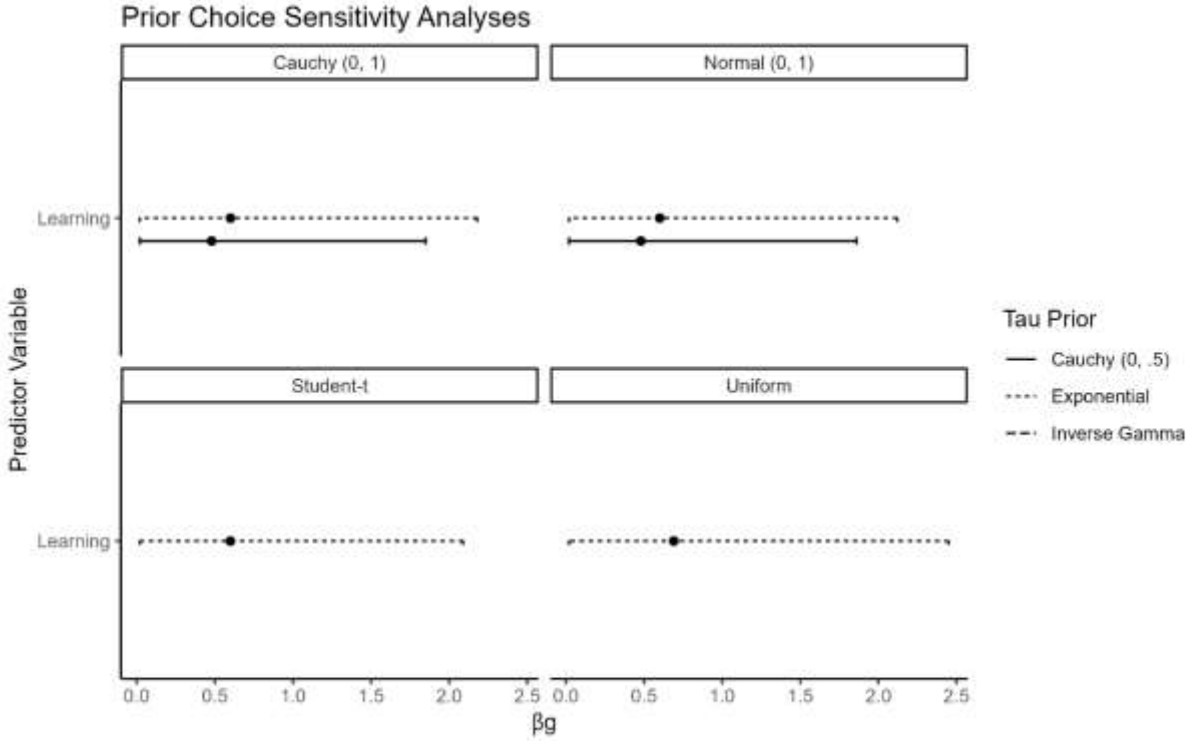

Figure 1. Study level standard deviation estimates with 95% confidence intervals for the Fuld Object Memory Examination: Learning model.

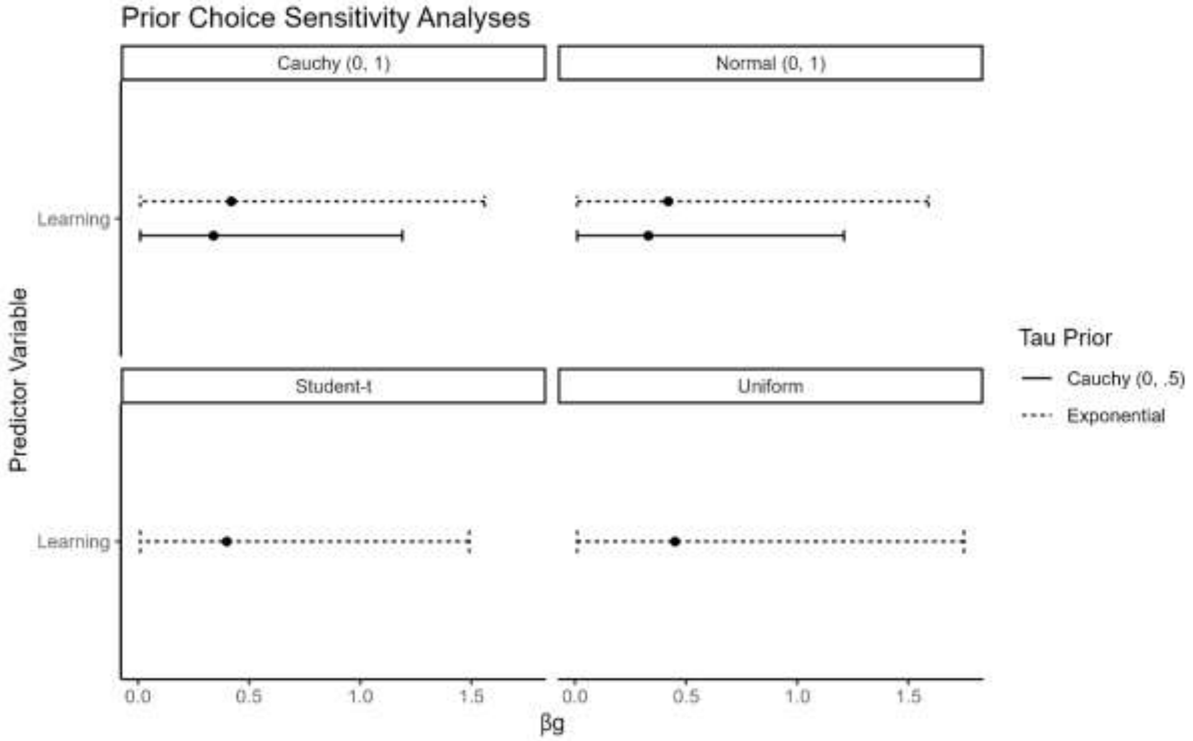

Figure 1. Effect size level standard deviation estimates with 95% confidence intervals for the Fuld Object Memory Examination: Learning model.

General Measures of Verbal Memory

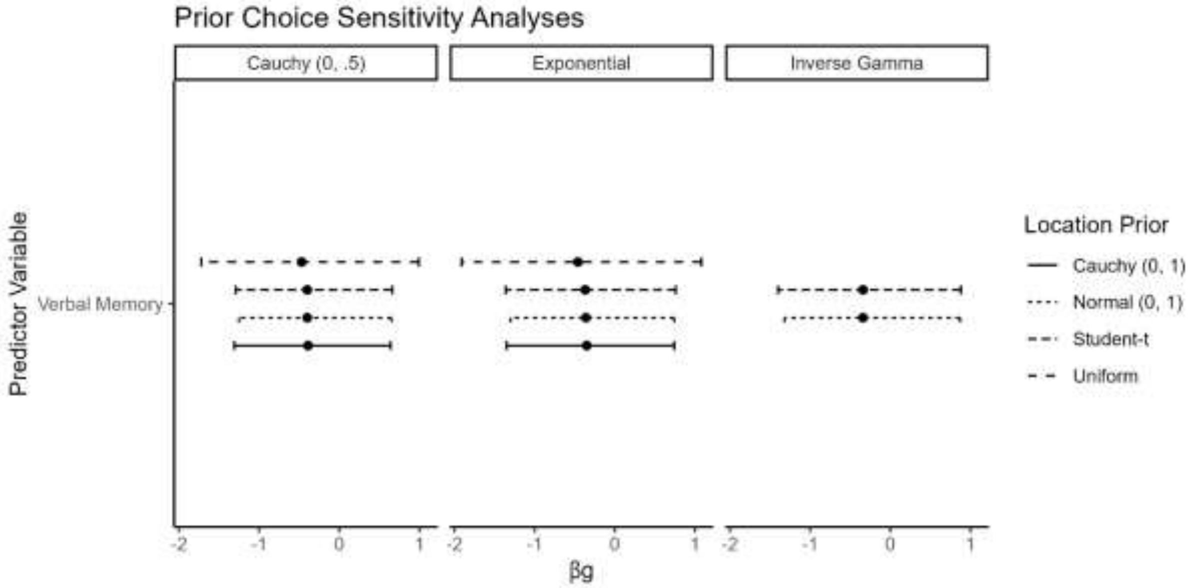

Figure 1. Regression coefficients with 95% confidence intervals for the General Measures of Verbal Memory model. For tests and studies included in the analysis see Supplementary Materials 1.

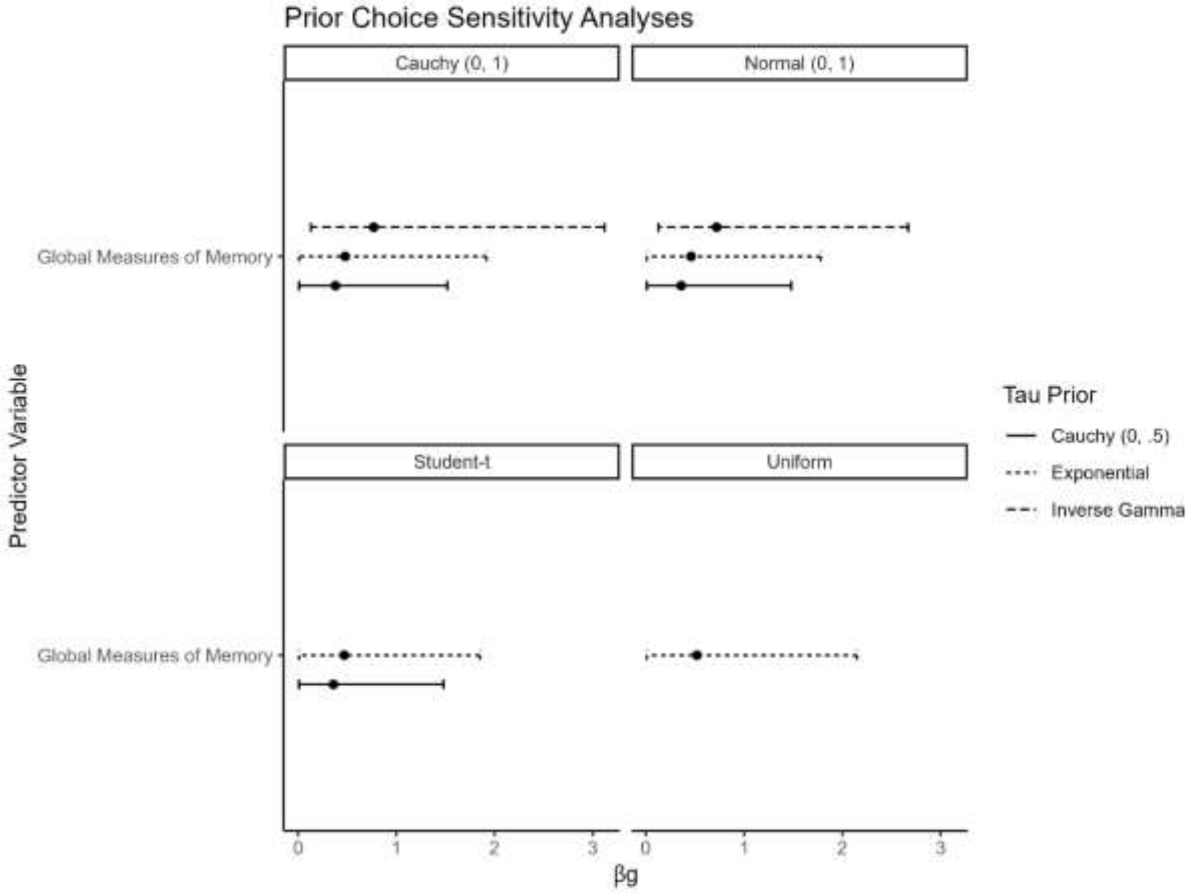

Figure 1. Study level standard deviation estimates with 95% confidence intervals for the General Measures of Verbal Memory model. For tests and studies included in the analysis see Supplementary Materials 1.

Other Measures of Verbal Learning

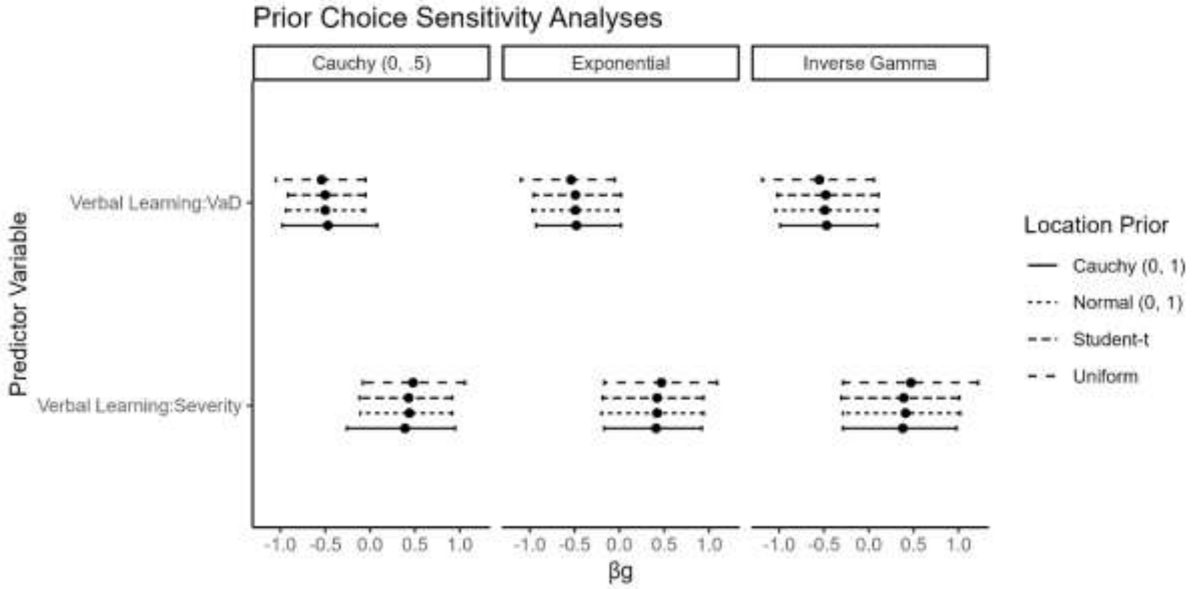

Figure 1. Regression coefficients with 95% confidence intervals for the Other Measures of Verbal Learning model. For tests and studies included in the analysis see Supplementary Materials 1. VaD: vascular dementia, Severity: difference in dementia severity between dementia groups.

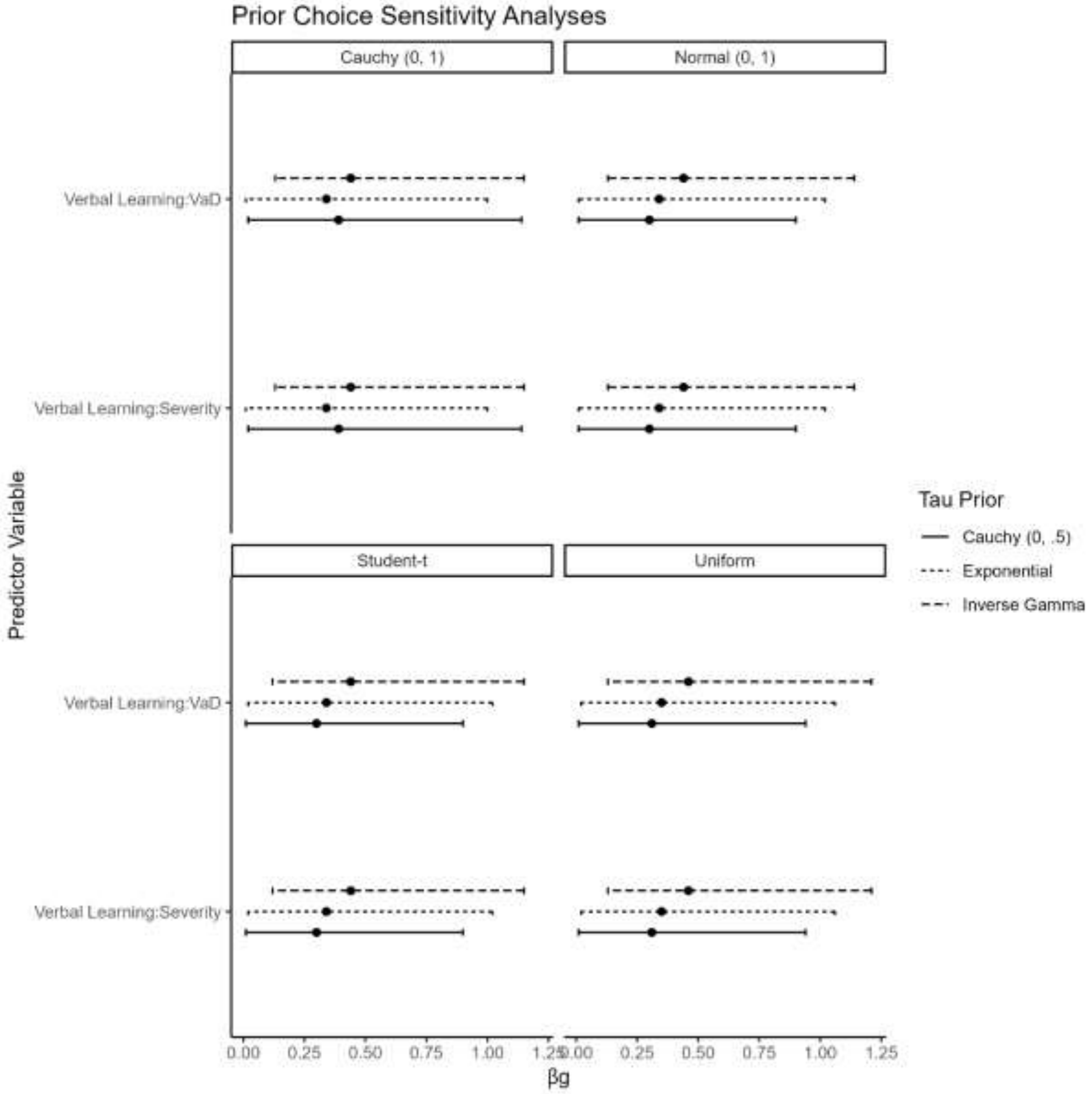

Figure 1. Study level standard deviation estimates with 95% confidence intervals for the Other Measures of Verbal Learning model. For tests and studies included in the analysis see Supplementary Materials 1. VaD: vascular dementia, Severity: difference in dementia severity between dementia groups.

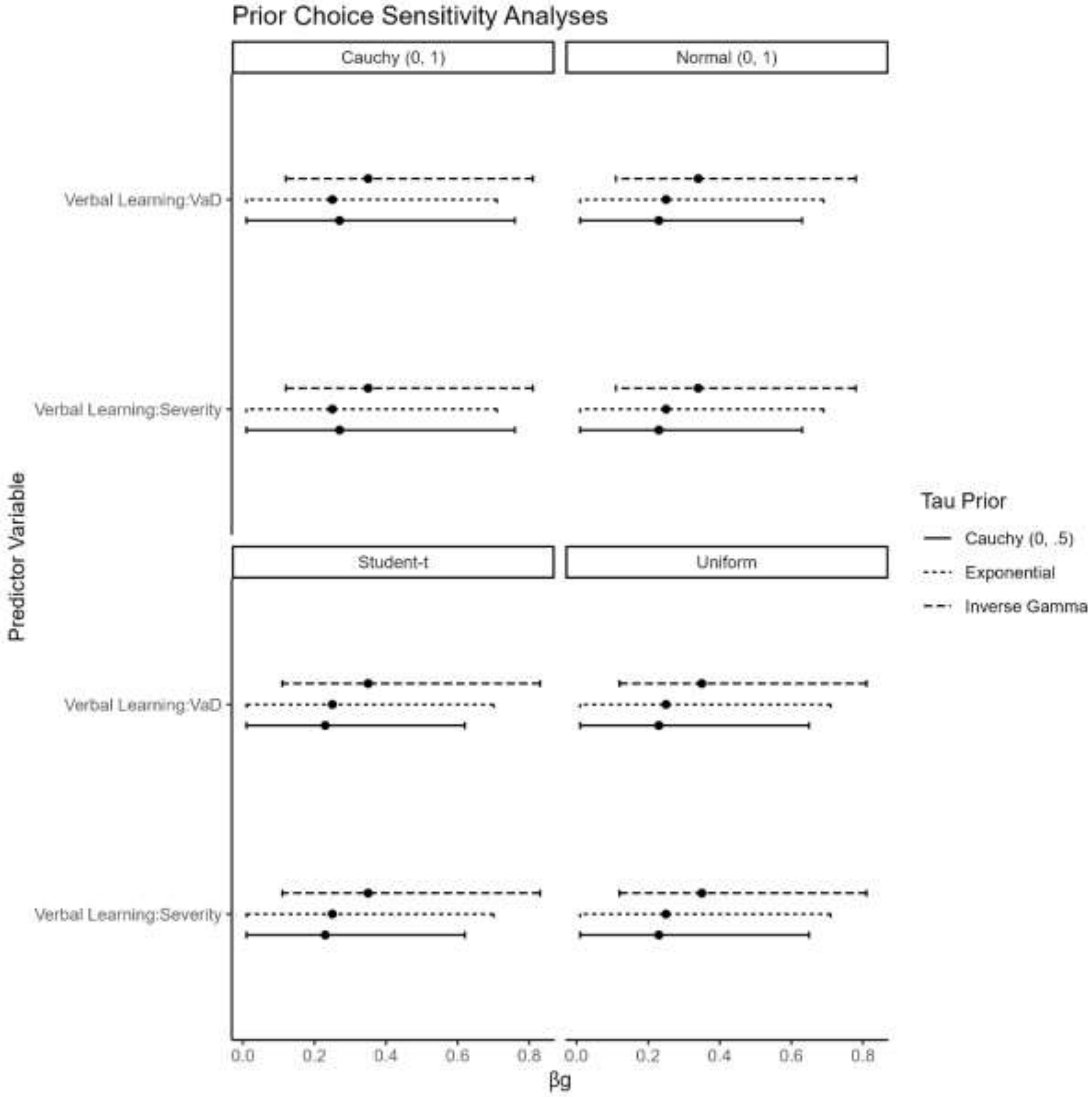

Figure 1. Effect size level standard deviation estimates with 95% confidence intervals for the Other Measures of Verbal Learning model. For tests and studies included in the analysis see Supplementary Materials 1. VaD: vascular dementia, Severity: difference in dementia severity between dementia groups.

Global Measures of Associative Memory

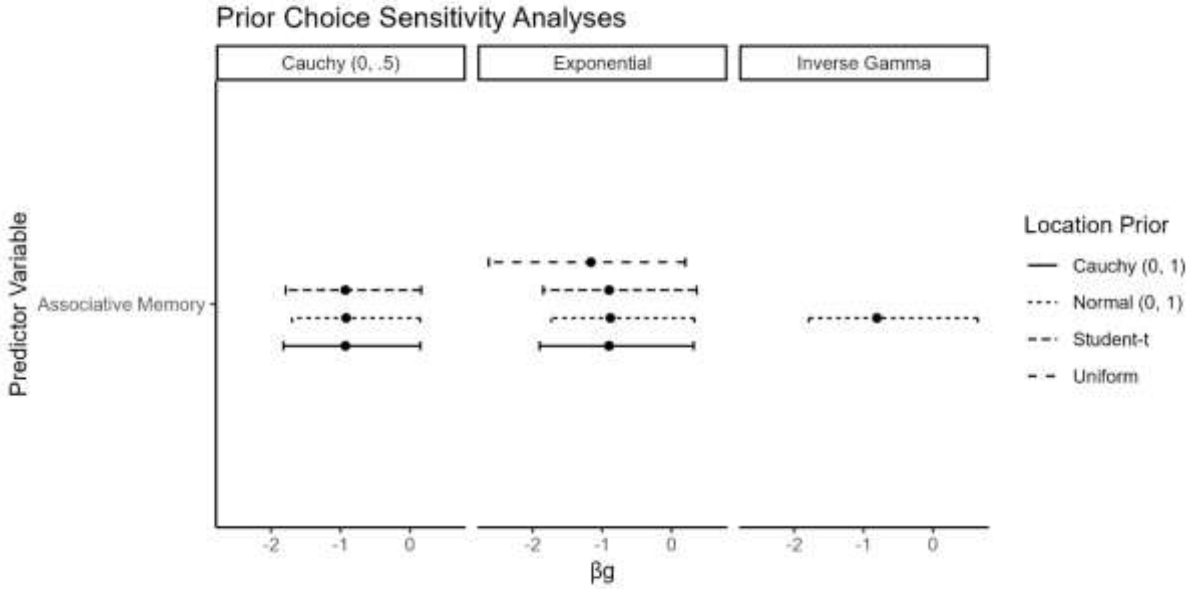

Figure 1. Regression coefficients with 95% confidence intervals for the Global Measures of Associative Memory model. For tests included in the analysis see Supplementary Materials 1.

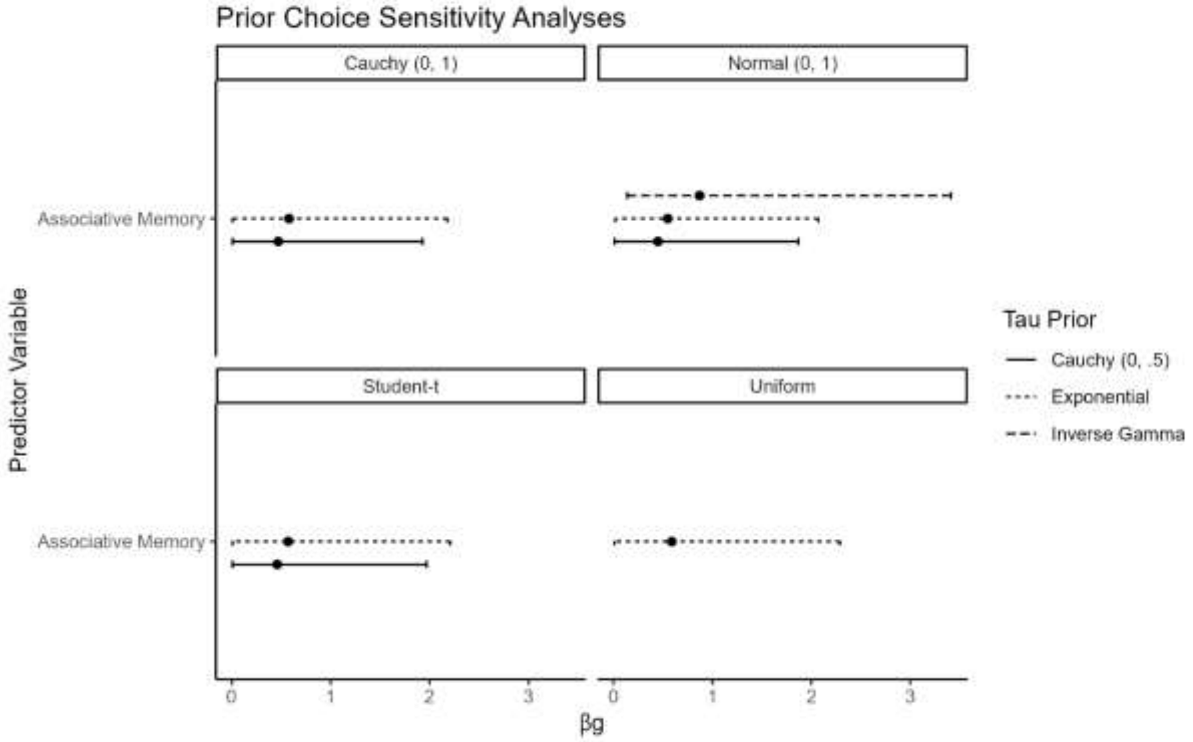

Figure 1. Study level standard deviation estimates with 95% confidence intervals for the Global Measures of Associative Memory model. For tests included in the analysis see Supplementary Materials 1.

Other Measures of Episodic Memory: Prose

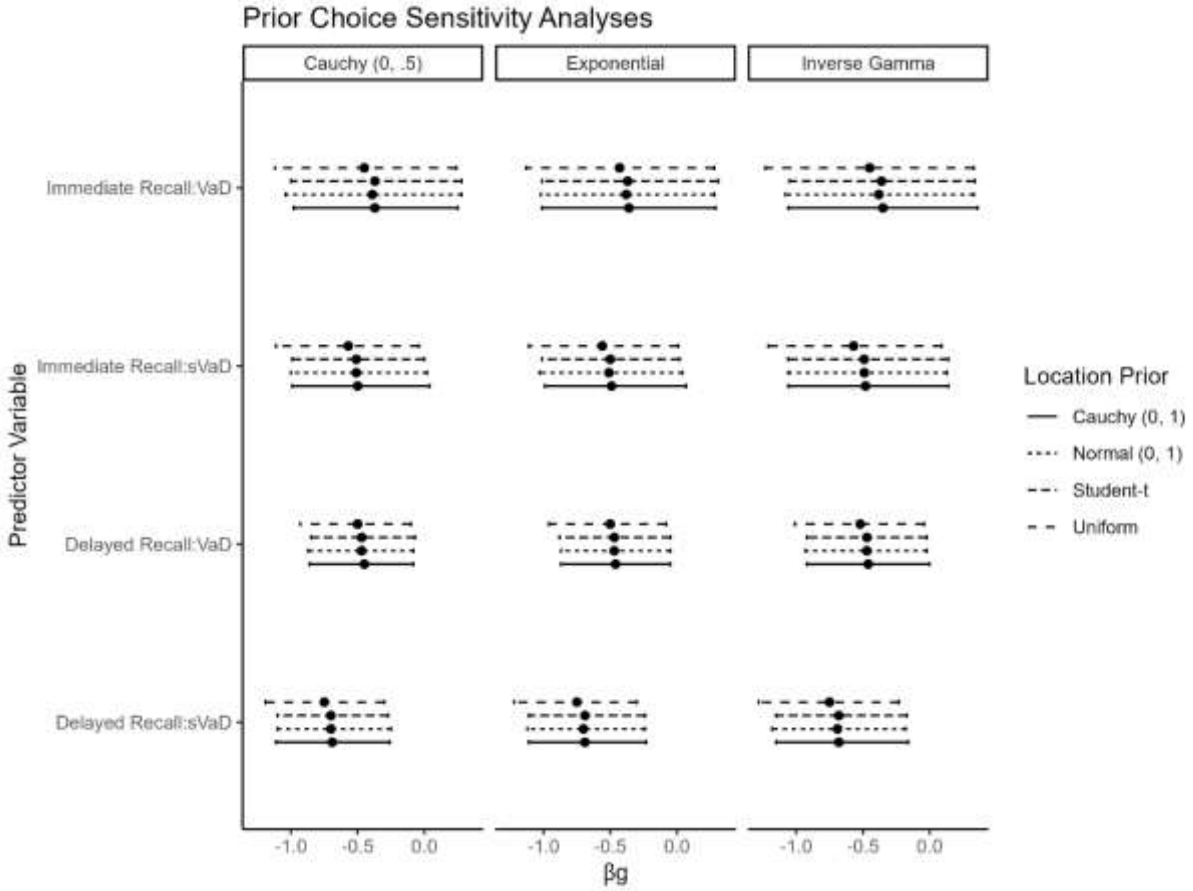

Figure 1. Regression coefficients with 95% confidence intervals for the Other Measures of Episodic Memory: Prose model. For tests and studies included in the analysis see Supplementary Materials 1. sVaD: subcortical vascular dementia, VaD: vascular dementia.

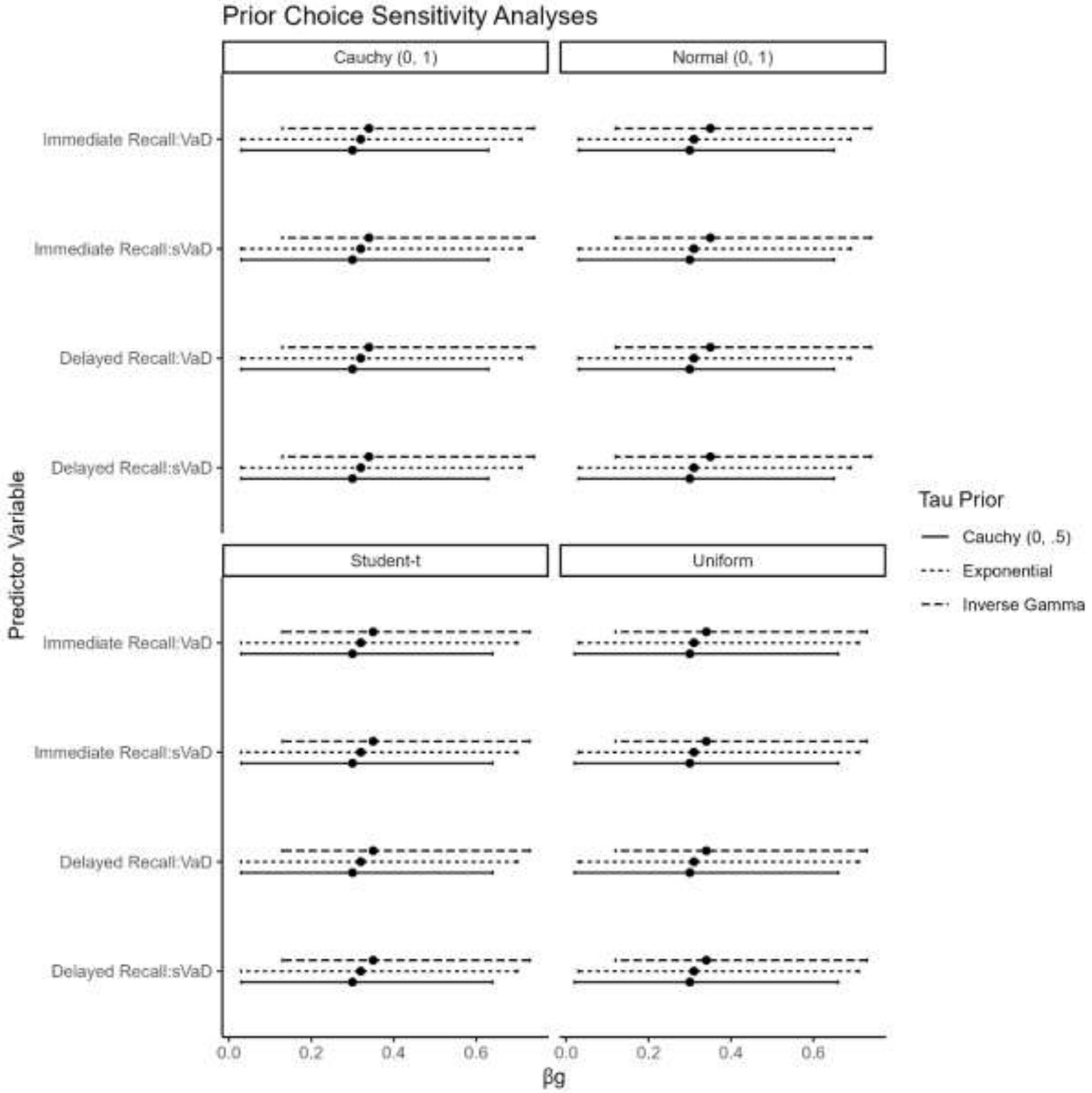

Figure 1. Study level standard deviation estimates with 95% confidence intervals for the Other Measures of Episodic Memory: Prose model. For tests and studies included in the analysis see Supplementary Materials 1. sVaD: subcortical vascular dementia, VaD: vascular dementia.

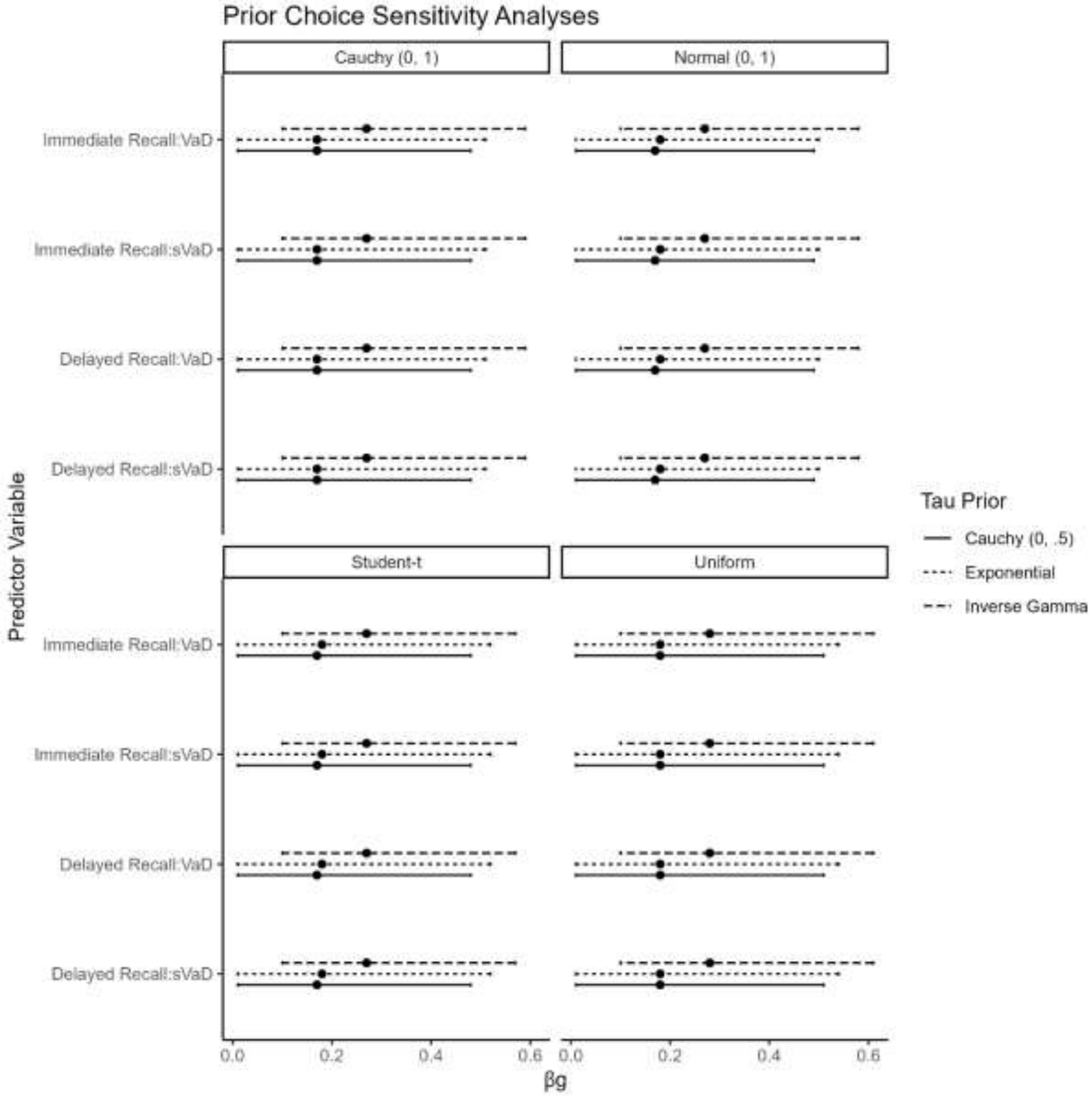

Figure 1. Effect size level standard deviation estimates with 95% confidence intervals for the Other Measures of Episodic Memory: Prose model. For tests and studies included in the analysis see Supplementary Materials 1. sVaD: subcortical vascular dementia, VaD: vascular dementia.

Other Measures of Episodic Memory: Word Lists

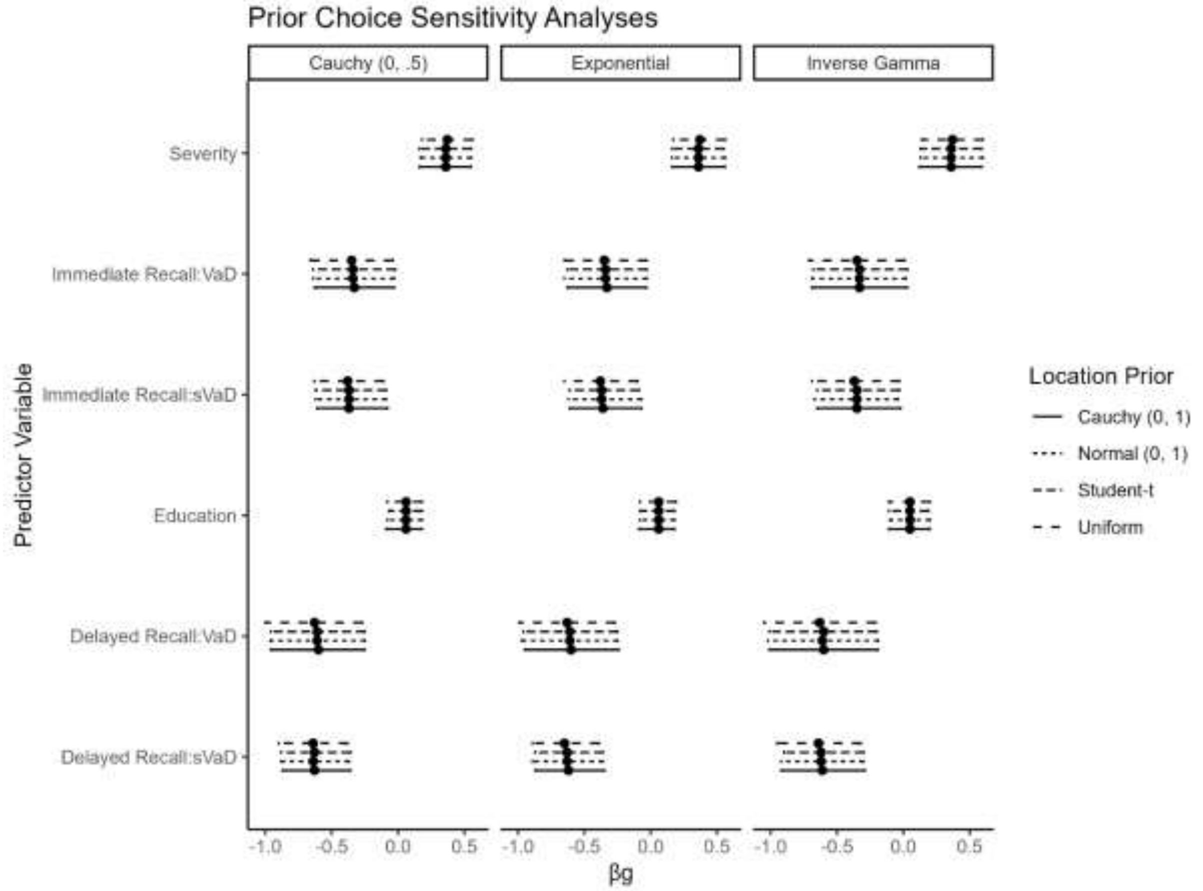

Figure 1. Regression coefficients with 95% confidence intervals for the Other Measures of Episodic Memory: Word Lists model. For tests and studies included in the analysis see Supplementary Materials 1. sVaD: subcortical vascular dementia, VaD: vascular dementia, Severity: difference in dementia severity between dementia groups, Education: difference in average years of education between dementia groups.

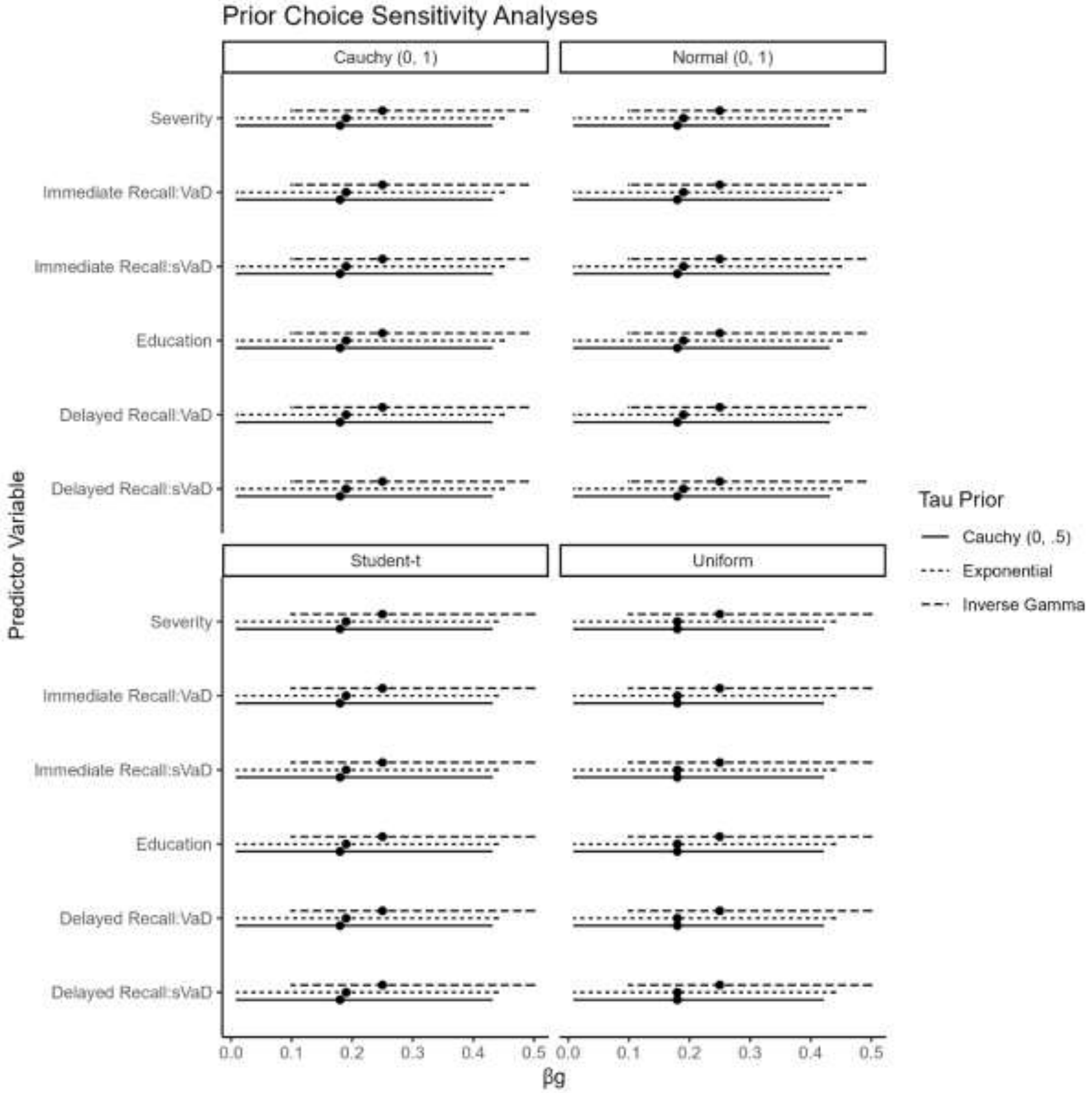

Figure 1. Study level standard deviation estimates with 95% confidence intervals for the Other Measures of Episodic Memory: Word Lists model. For tests and studies included in the analysis see Supplementary Materials 1. sVaD: subcortical vascular dementia, VaD: vascular dementia, Severity: difference in dementia severity between dementia groups, Education: difference in average years of education between dementia groups.

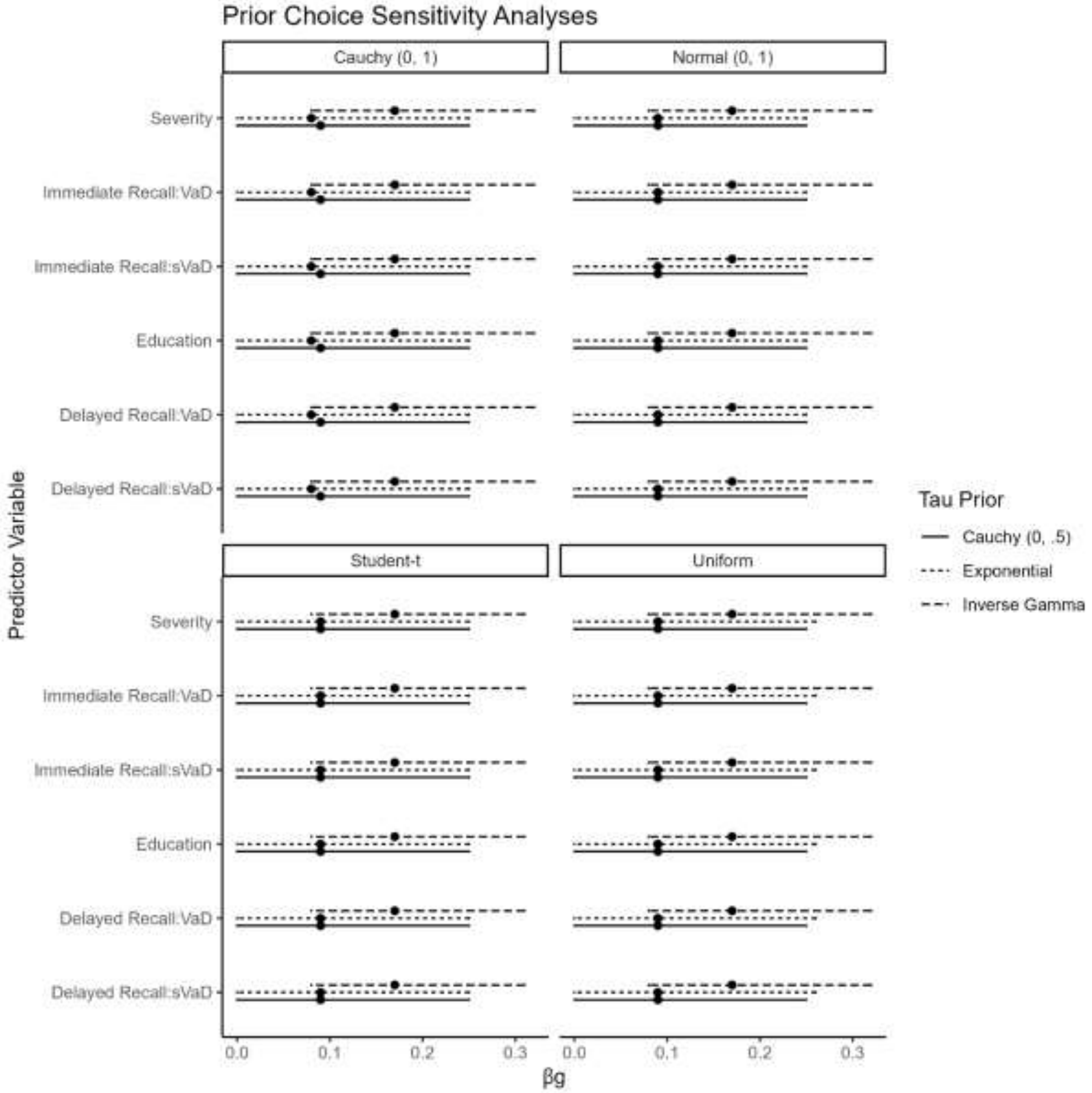

Figure 1. Effect size level standard deviation estimates with 95% confidence intervals for the Other Measures of Episodic Memory: Word Lists model. For tests and studies included in the analysis see Supplementary Materials 1. sVaD: subcortical vascular dementia, VaD: vascular dementia, Severity: difference in dementia severity between dementia groups, Education: difference in average years of education between dementia groups.

Other Measures of Cued Recall of Word Lists

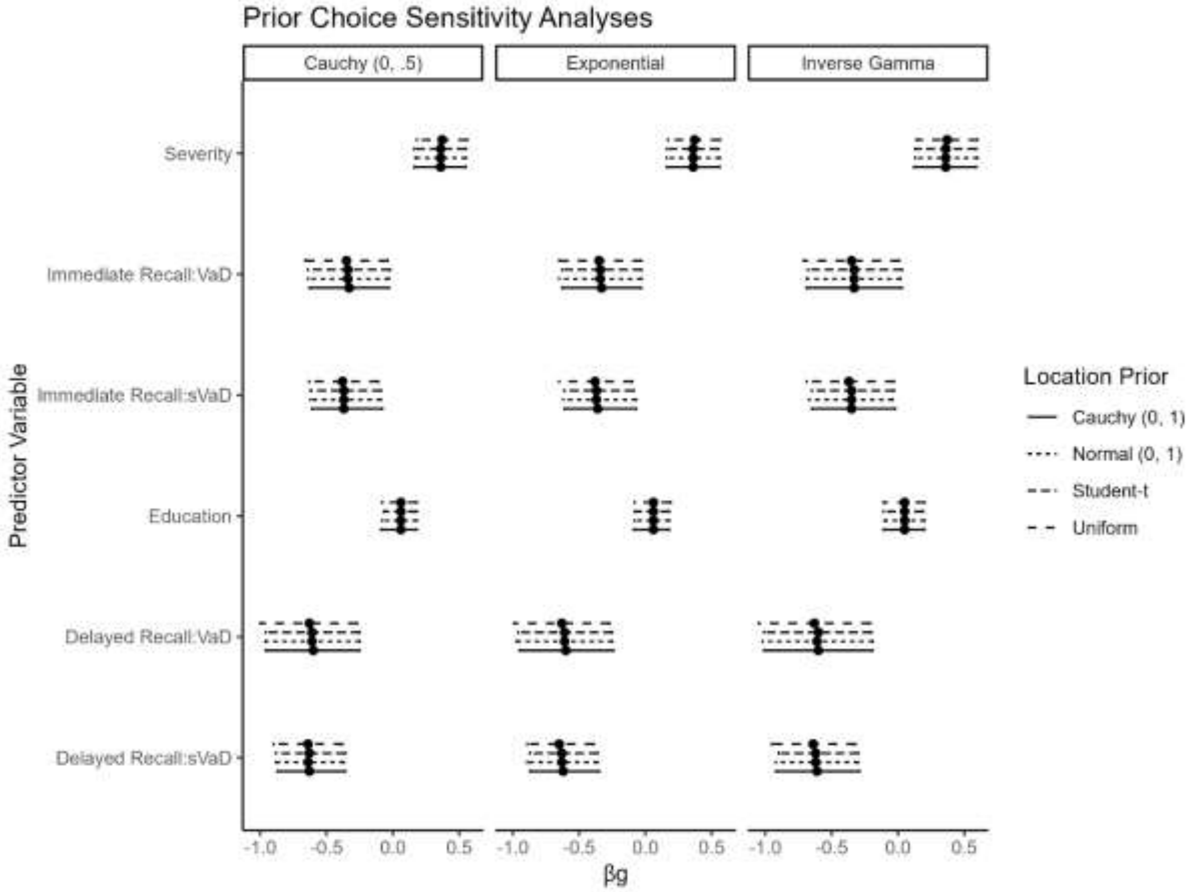

Figure 1. Regression coefficients with 95% confidence intervals for the Other Measures of Cued Recall of Word Lists model. For tests and studies included in the analysis see Supplementary Materials 1. VaD: vascular dementia, Severity: difference in dementia severity between dementia groups.

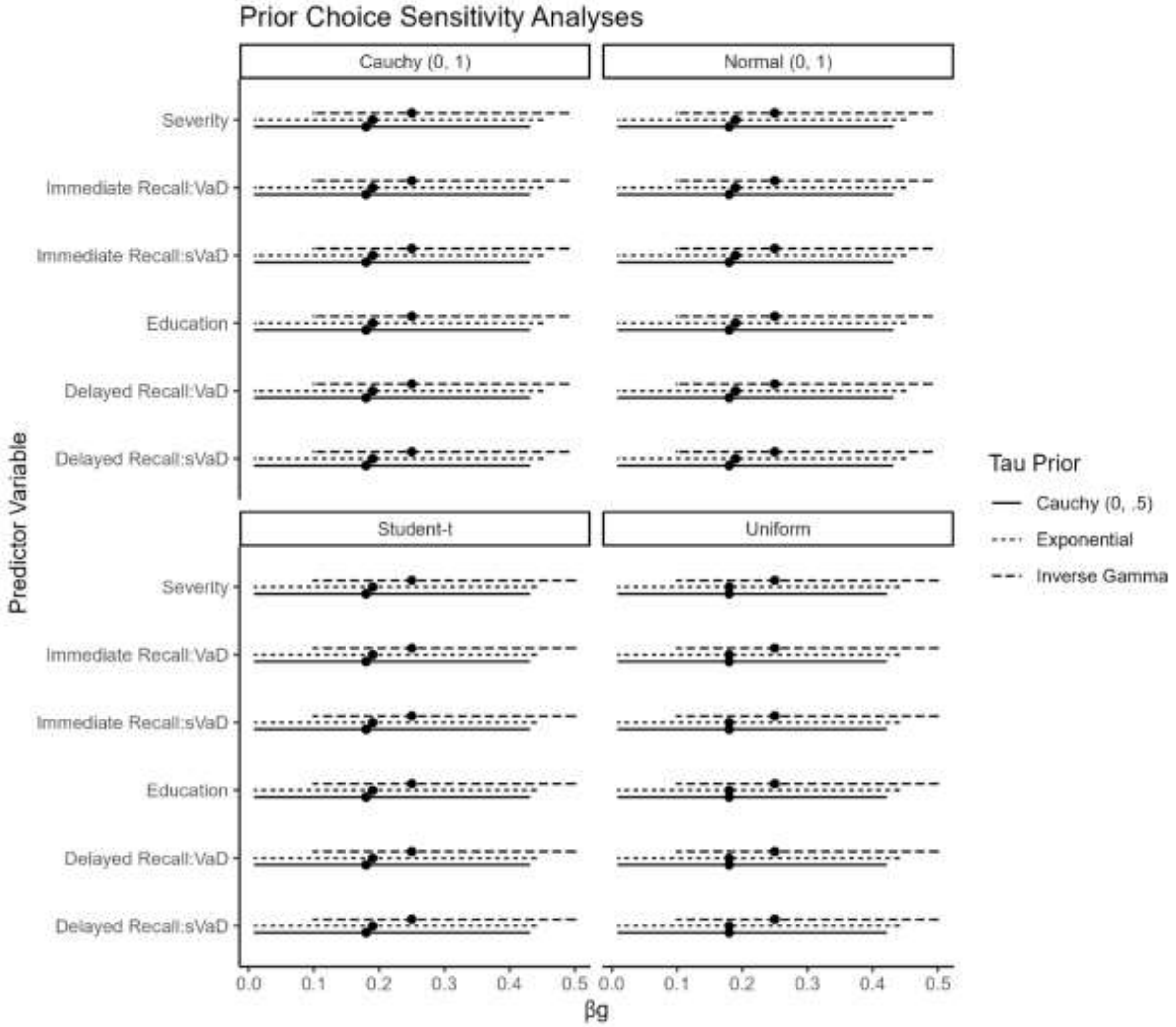

Figure 1. Study level standard deviation estimates with 95% confidence intervals for the Other Measures of Cued Recall of Word Lists model. For tests and studies included in the analysis see Supplementary Materials 1. VaD: vascular dementia, Severity: difference in dementia severity between dementia groups.

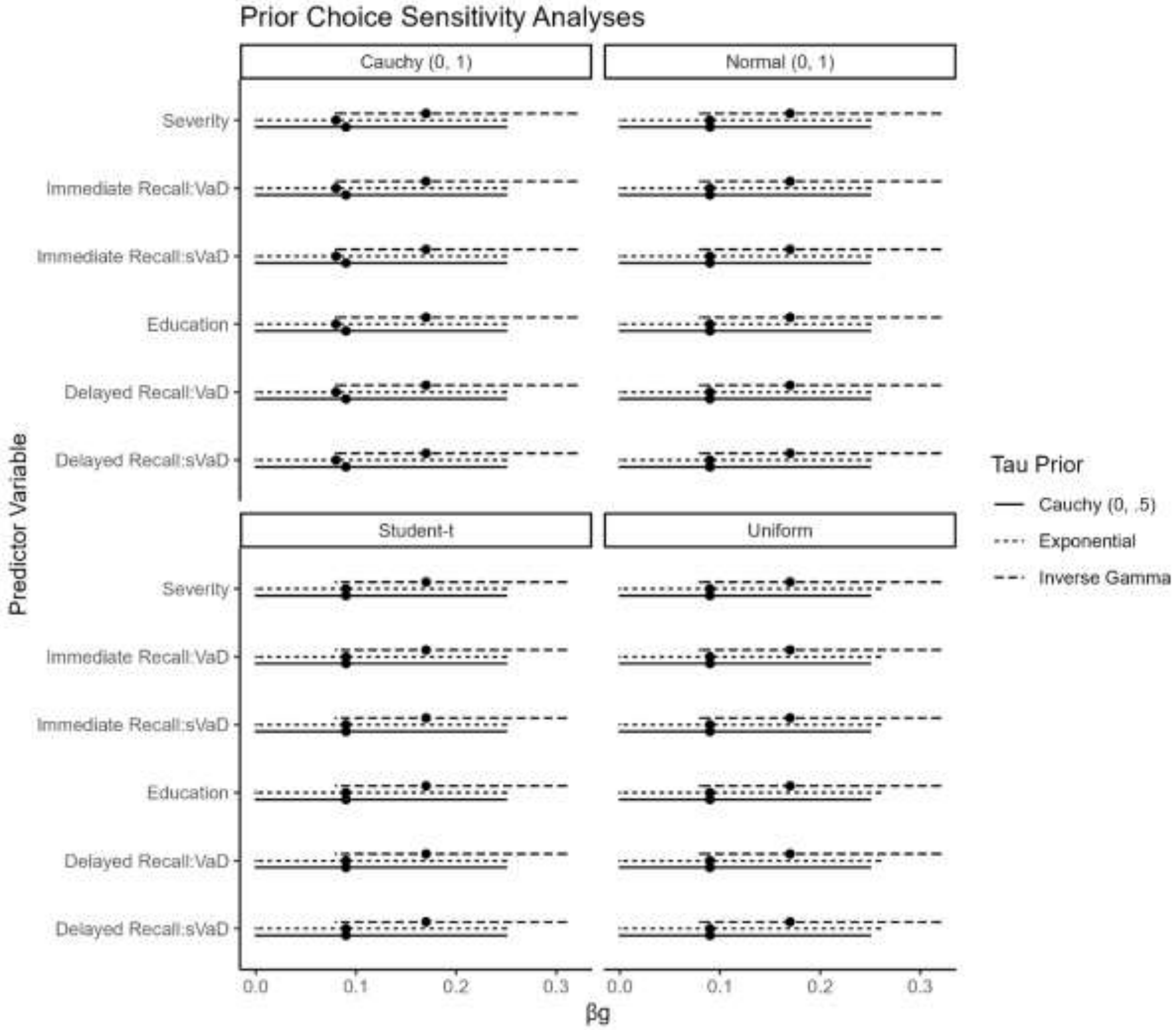

Figure 1. Effect size level standard deviation estimates with 95% confidence intervals for the Other Measures of Cued Recall of Word Lists model. For tests and studies included in the analysis see Supplementary Materials 1. VaD: vascular dementia, Severity: difference in dementia severity between dementia groups.

Memory Intrusions

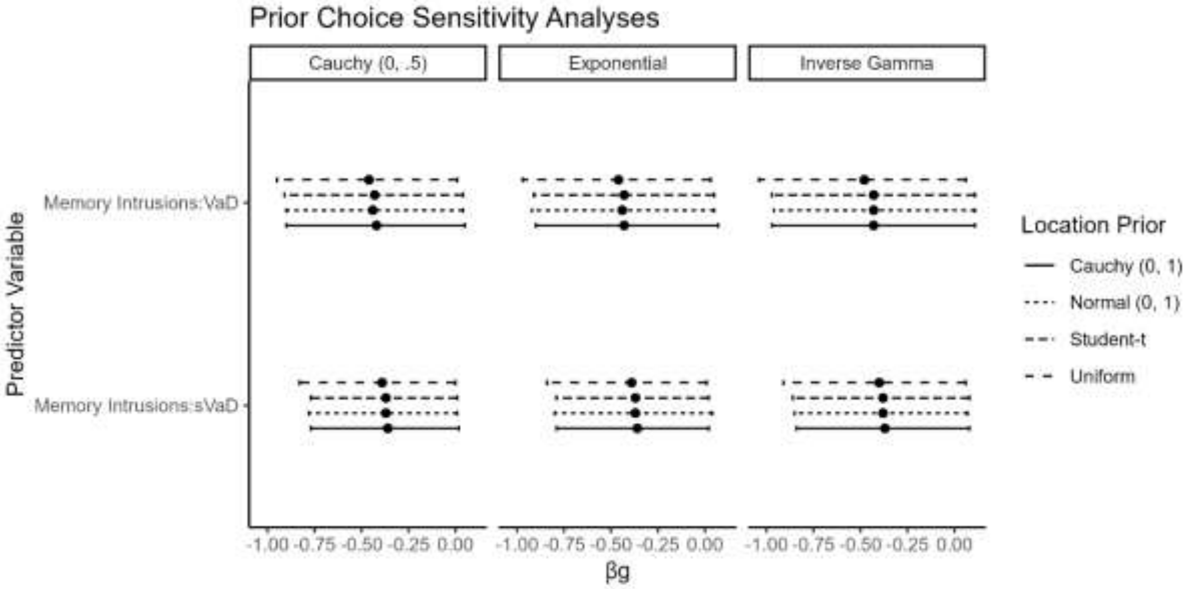

Figure 1. Regression coefficients with 95% confidence intervals for the Memory Intrusions model. For tests and studies included in the analysis see Supplementary Materials 1. sVaD: subcortical vascular dementia, VaD: vascular dementia.

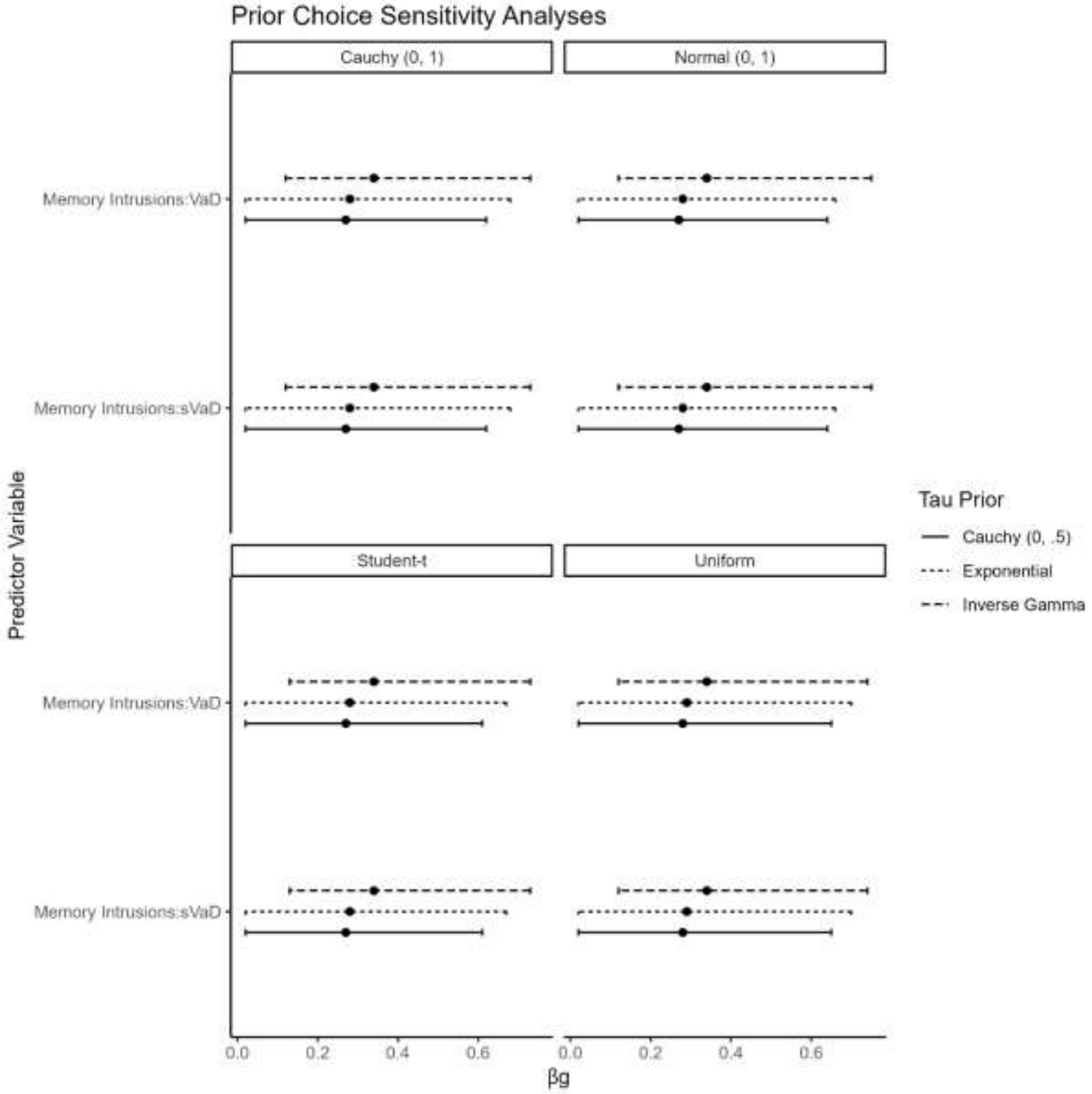

Figure 1. Study level standard deviation estimates with 95% confidence intervals for the Memory Intrusions model. For tests and studies included in the analysis see Supplementary Materials 1. sVaD: subcortical vascular dementia, VaD: vascular dementia.

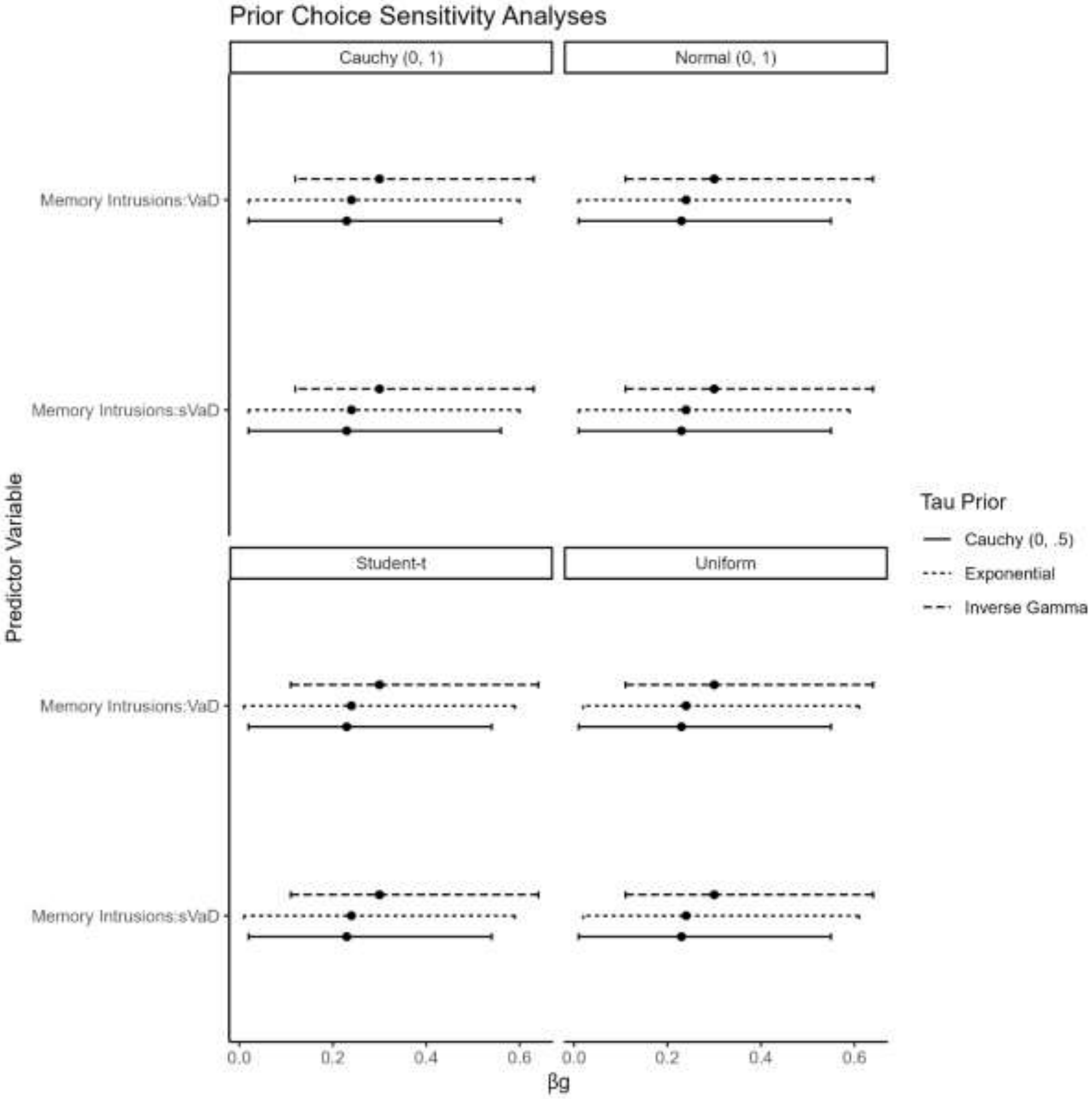

Figure 1. Effect size level standard deviation estimates with 95% confidence intervals for the Memory Intrusions model. For tests and studies included in the analysis see Supplementary Materials 1. sVaD: subcortical vascular dementia, VaD: vascular dementia.

Semantic Memory

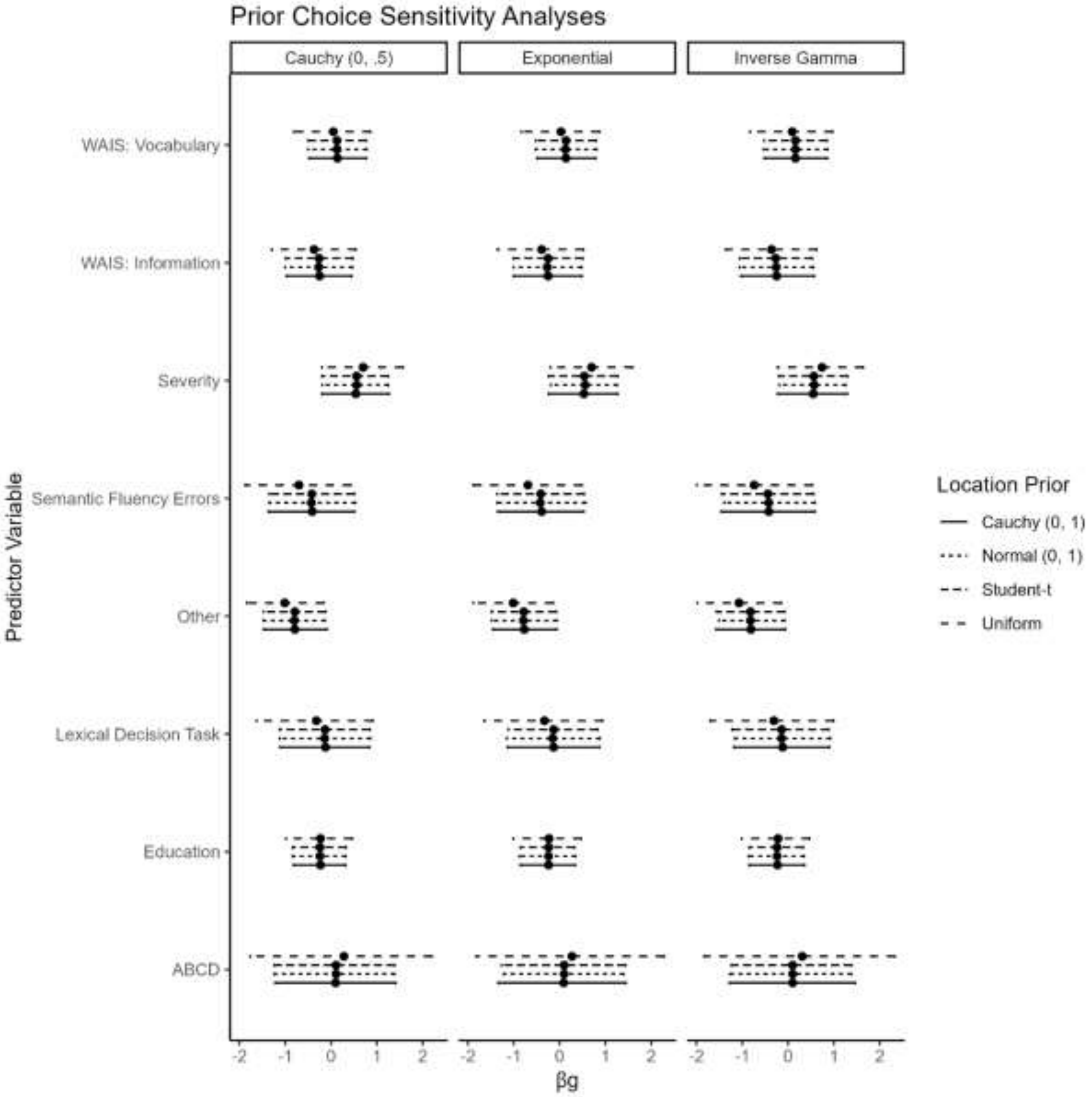

Figure 1. Regression coefficients with 95% confidence intervals for the Semantic Memory Measures model. For tests and studies included in the analysis see Supplementary Materials 1. WAIS: Wechsler Adult Intelligence Scale, ABCD: Arizona Battery for Communication Disorders of Dementia, Other: Other Measures of Semantic Memory, Severity: difference in dementia severity between dementia groups, Education: difference in average years of education between dementia groups.

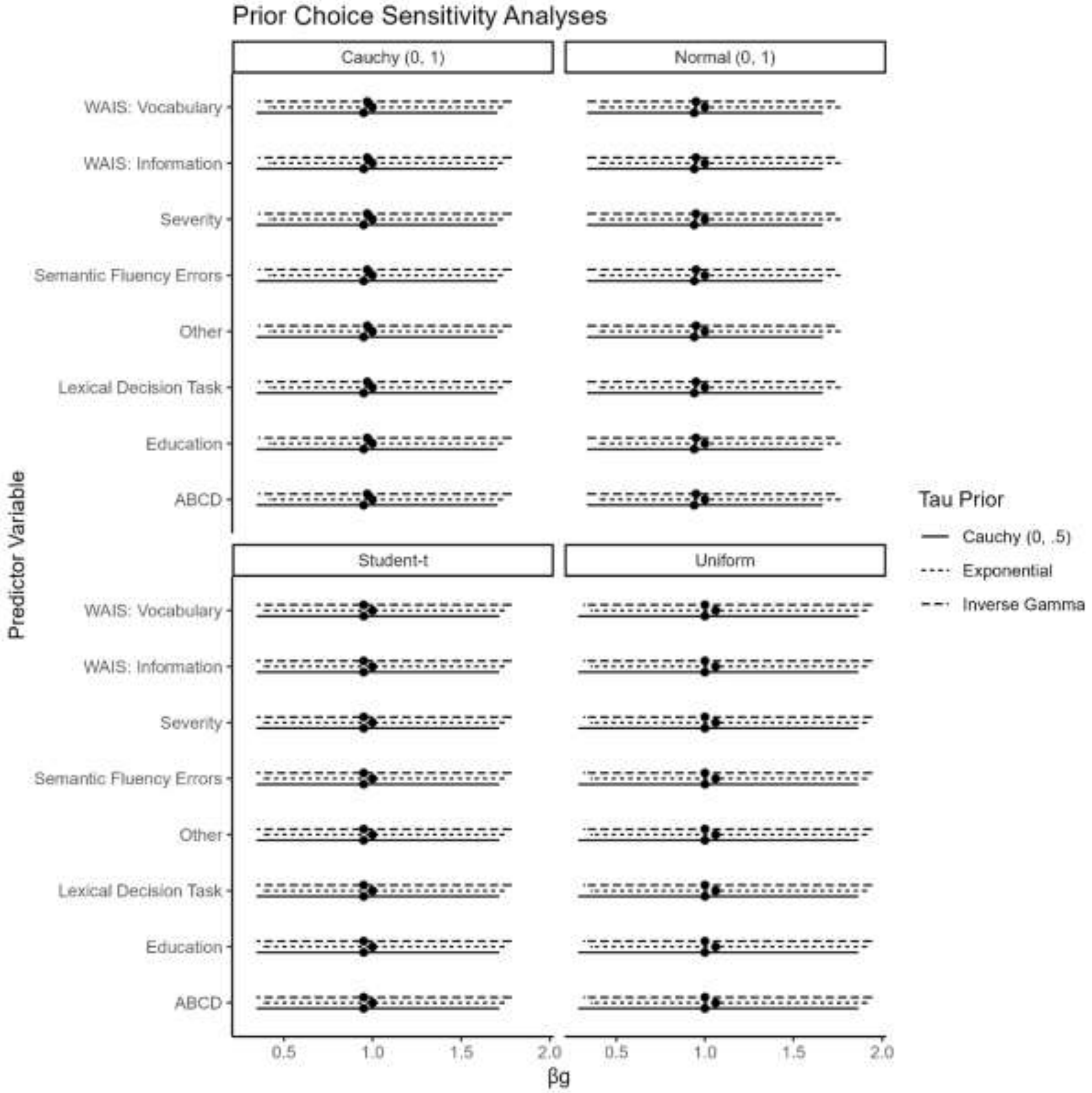

Figure 1. Study level standard deviation estimates with 95% confidence intervals for the Semantic Memory Measures model. For tests and studies included in the analysis see Supplementary Materials 1. WAIS: Wechsler Adult Intelligence Scale, ABCD: Arizona Battery for Communication Disorders of Dementia, Other: Other Measures of Semantic Memory, Severity: difference in dementia severity between dementia groups, Education: difference in average years of education between dementia groups.

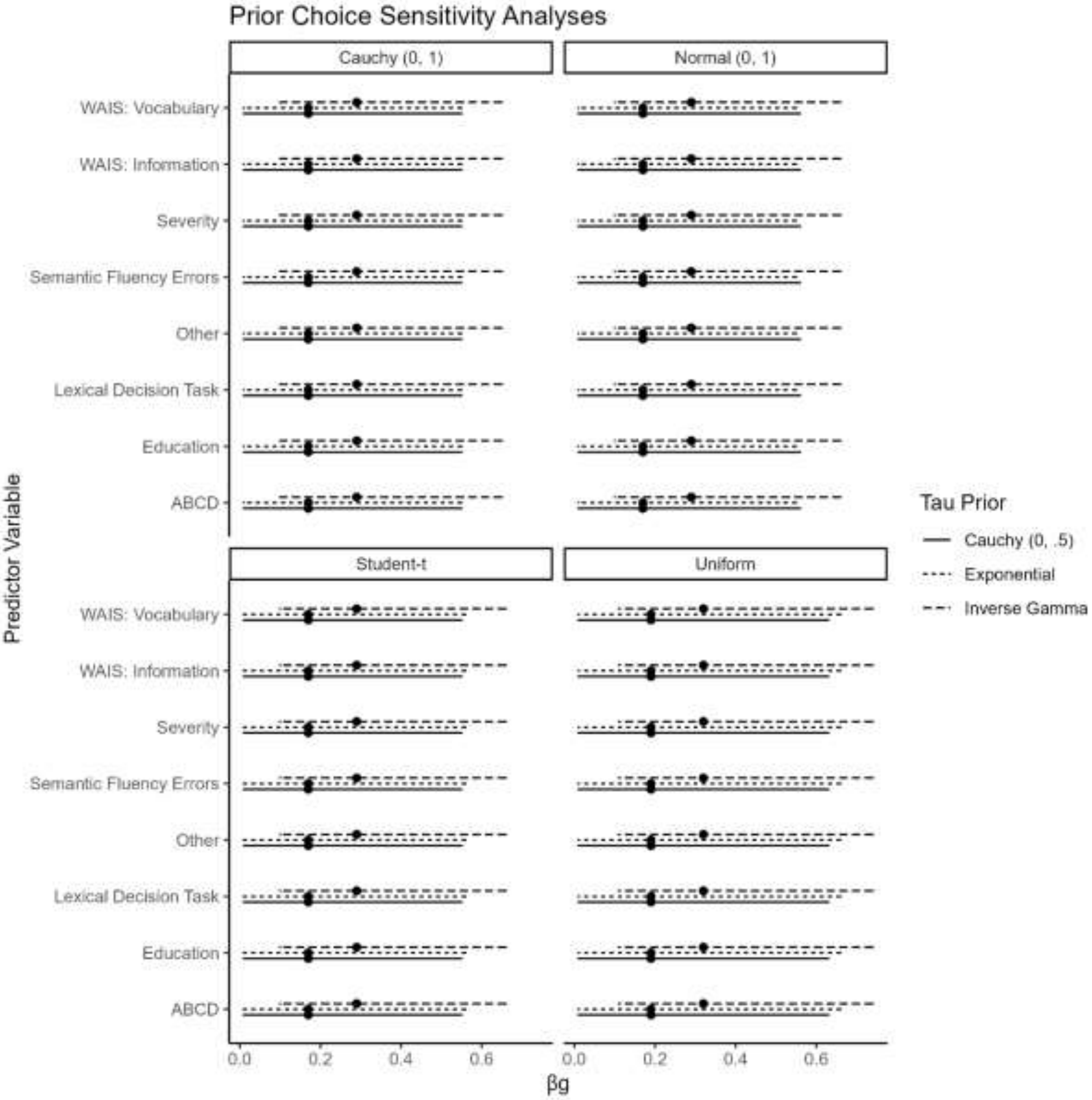

Figure 1. Effect size level standard deviation estimates with 95% confidence intervals for the Semantic Memory Measures model. For tests and studies included in the analysis see Supplementary Materials 1. WAIS: Wechsler Adult Intelligence Scale, ABCD: Arizona Battery for Communication Disorders of Dementia, Other: Other Measures of Semantic Memory, Severity: difference in dementia severity between dementia groups, Education: difference in average years of education between dementia groups.

Global Measures of Memory

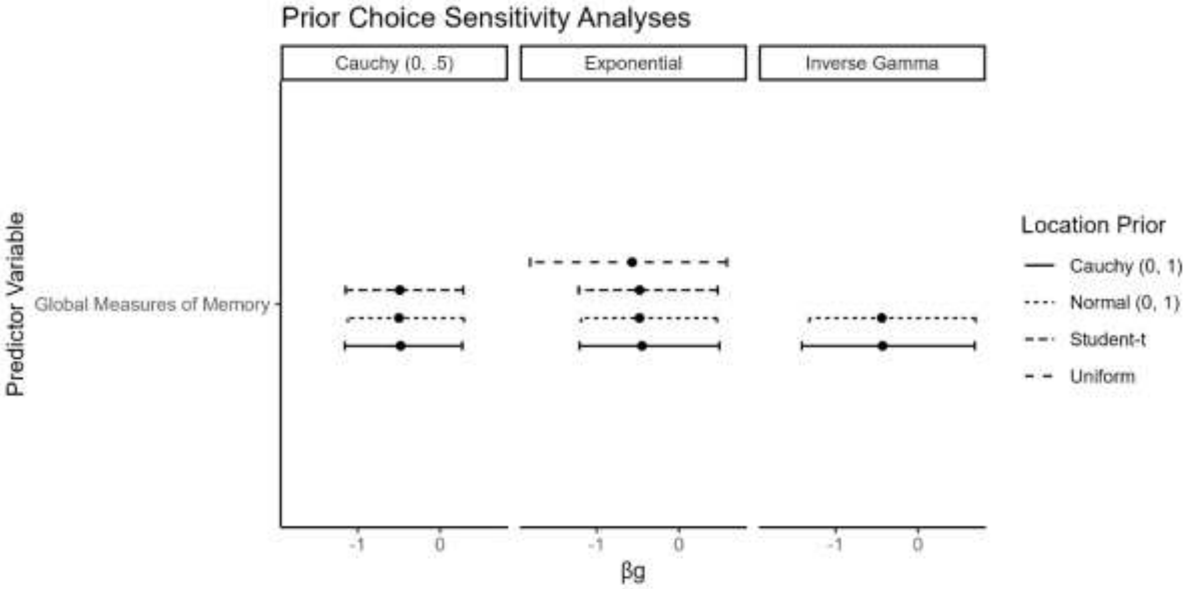

Figure 1. Regression coefficients with 95% confidence intervals for the Global Measures of Memory model. For tests and studies included in the analysis see Supplementary Materials 1.

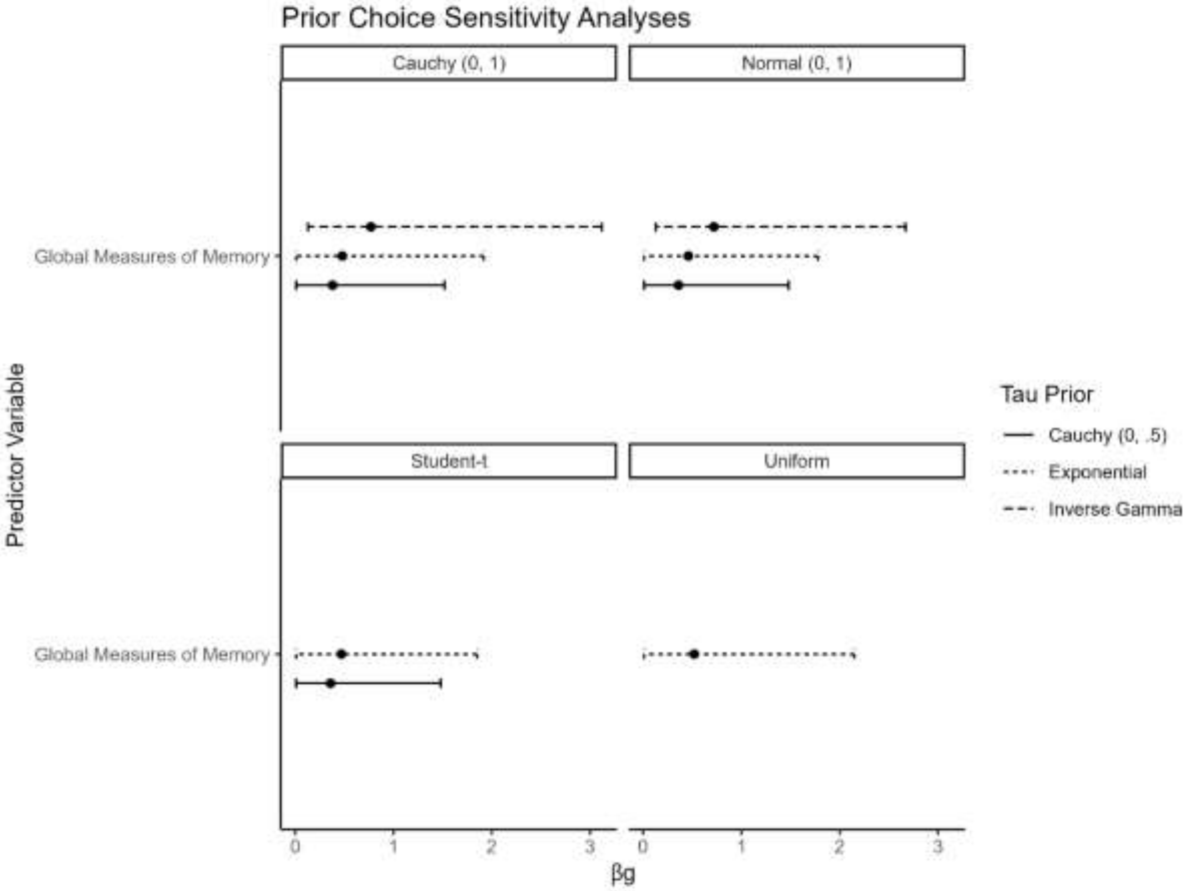

Figure 1. Study level standard deviation estimates with 95% confidence intervals for the Global Measures of Memory model. For tests and studies included in the analysis see Supplementary Materials 1.

Quality Sensitivity Analysis

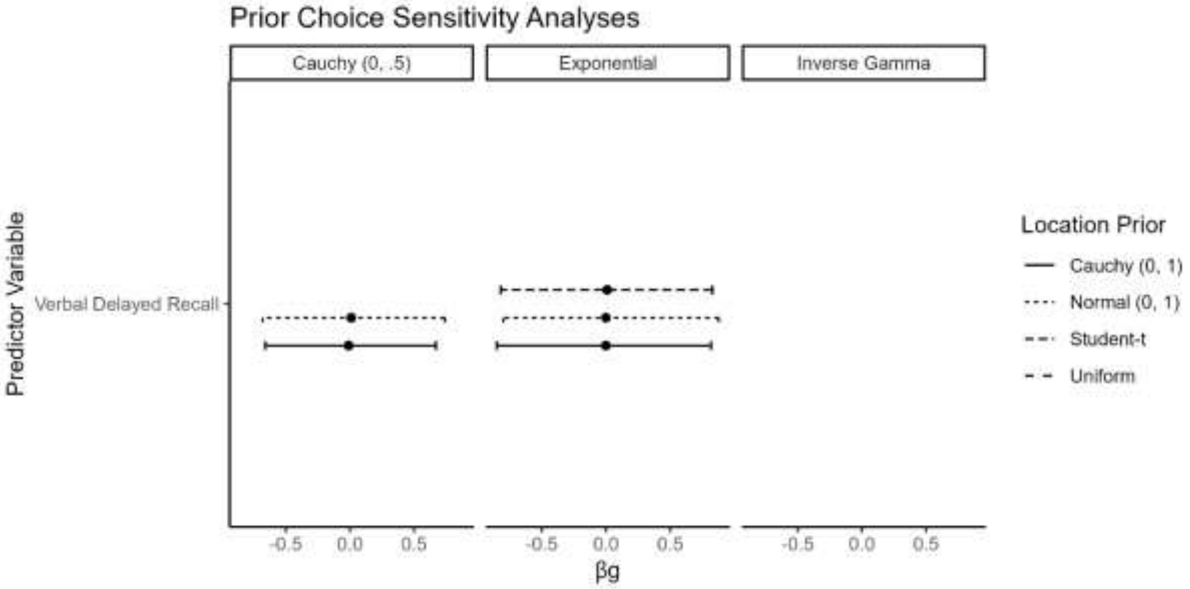

Figure 1. Regression coefficients with 95% confidence intervals for the study quality sensitivity analysis model for verbal delayed recall. For tests and studies included in the analysis see Supplementary Materials 1.

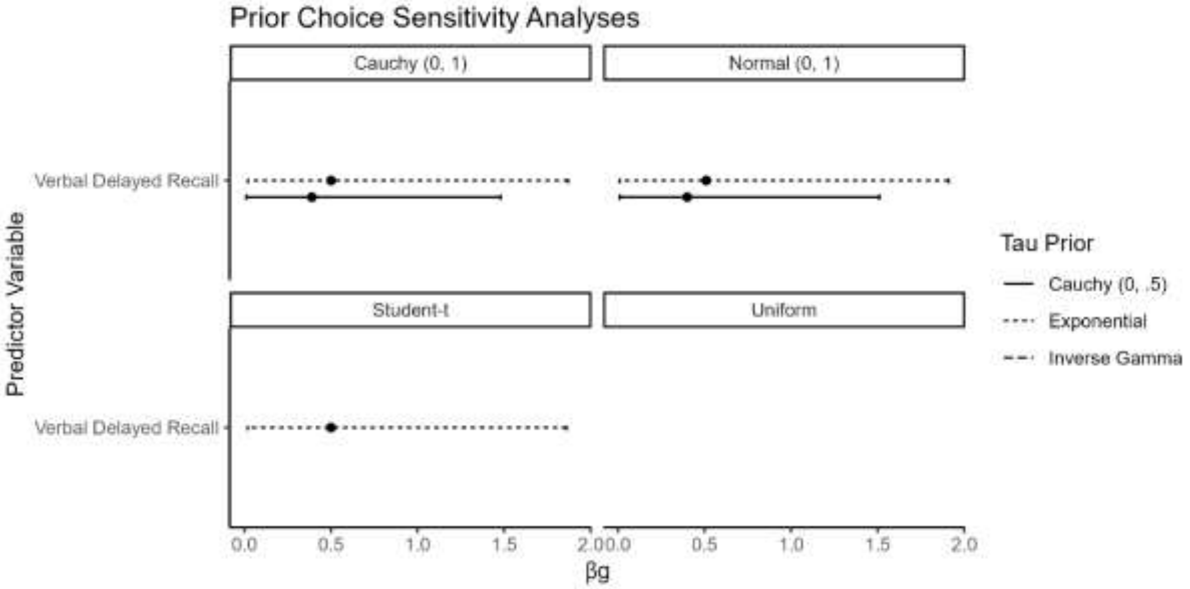

Figure 1. Study level standard deviation estimates with 95% confidence intervals for the study quality sensitivity analysis model for verbal delayed recall. For tests and studies included in the analysis see Supplementary Materials 1.

Visual Episodic Memory

Wechsler Memory Scale

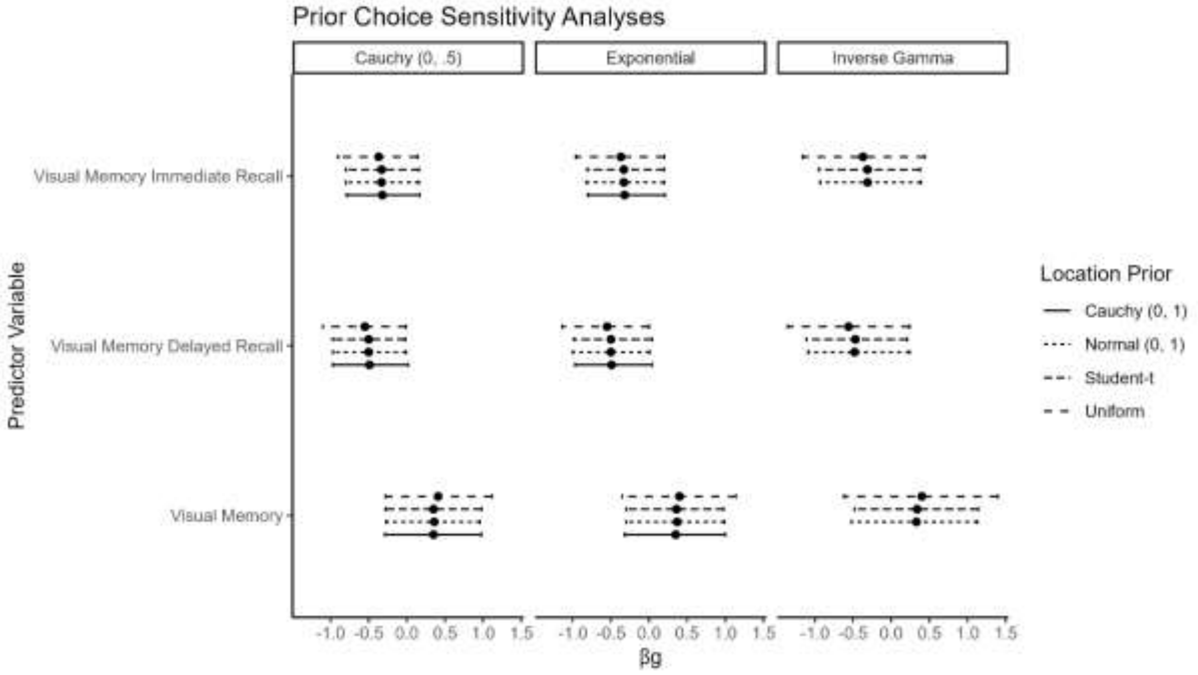

Figure 1. Regression coefficients with 95% confidence intervals for the Wechsler Memory Scale:Visual Episodic Memory subtests model.

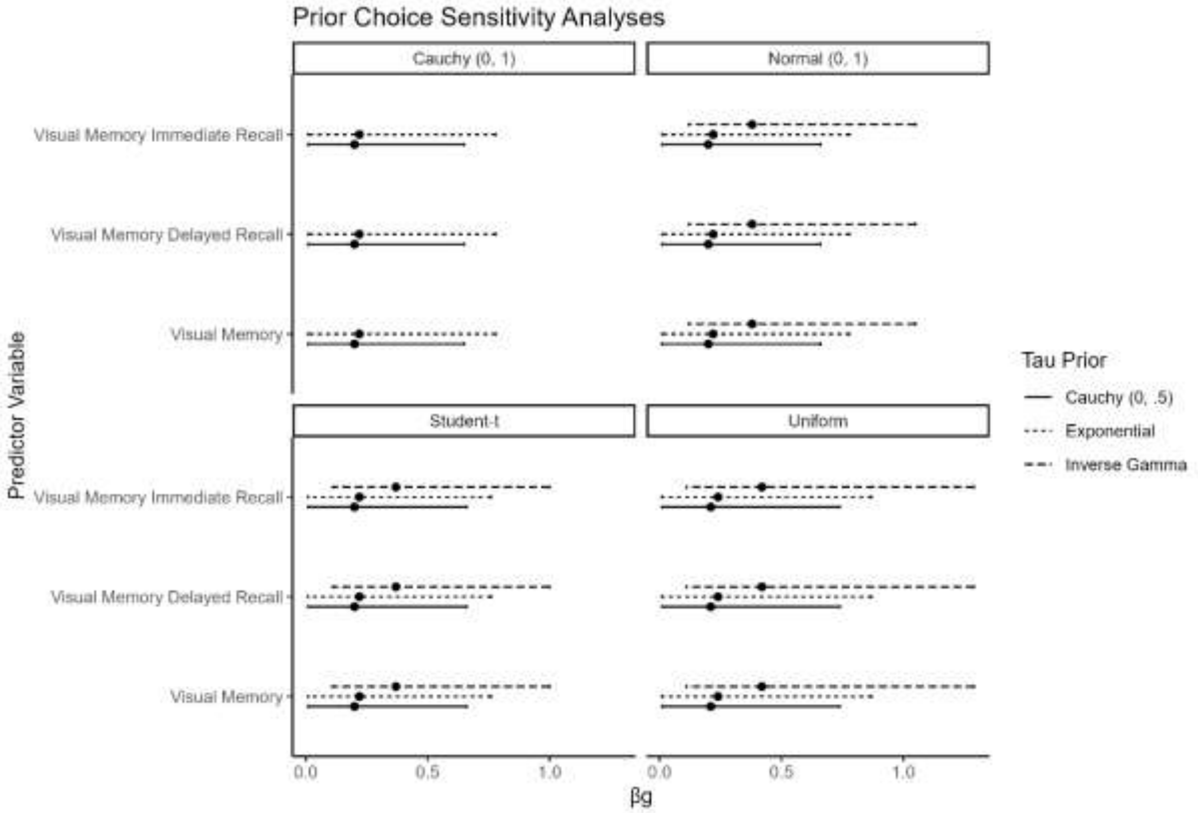

Figure 1. Study level standard deviation estimates with 95% confidence intervals for the Wechsler Memory Scale:Visual Episodic Memory subtests model.

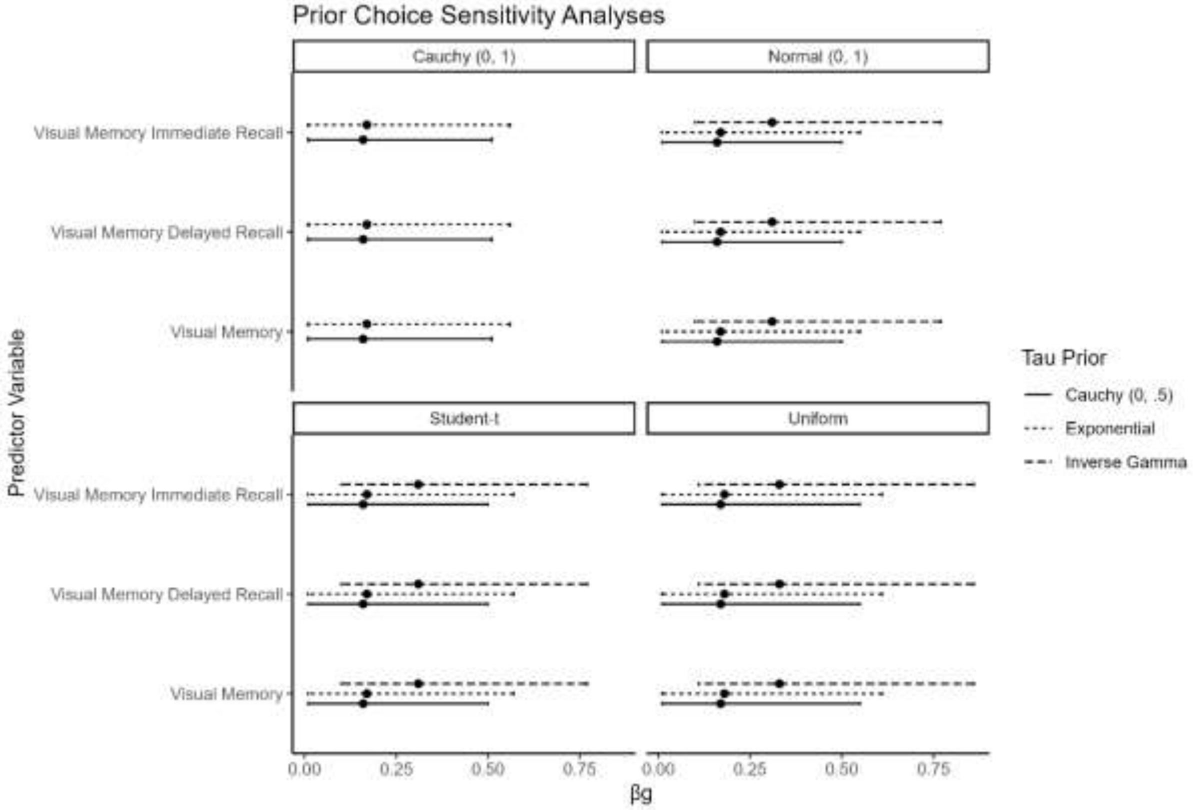

Figure 1. Effect size level standard deviation estimates with 95% confidence intervals for the Wechsler Memory Scale:Visual Episodic Memory subtests model.

Rey-Osterrieth Complex Figure Test

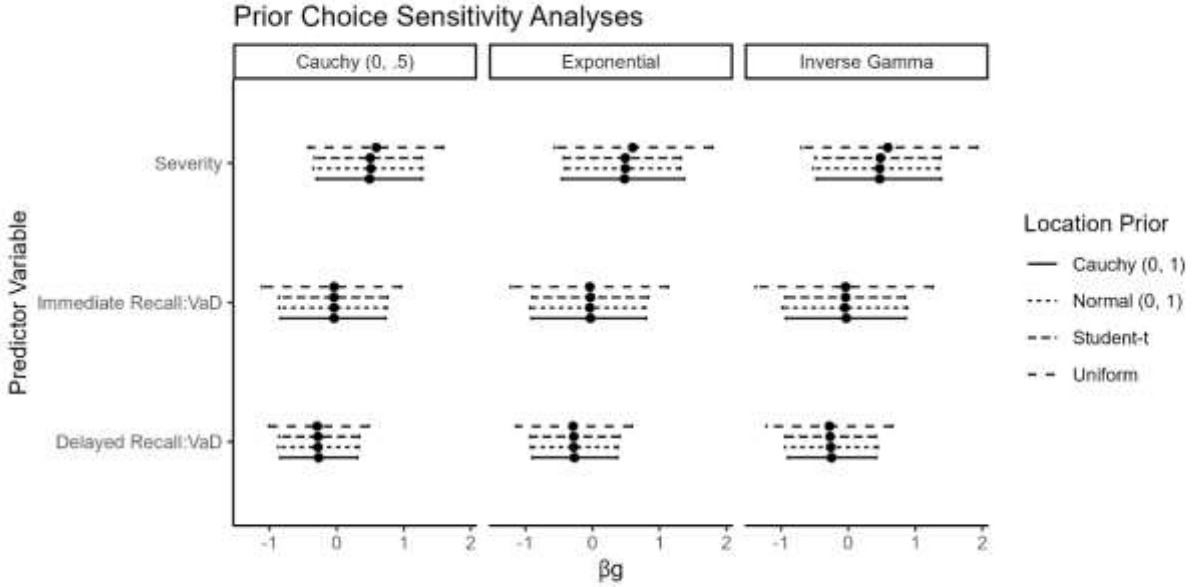

Figure 1. Regression coefficients with 95% confidence intervals for the Rey-Osterrieth Complex Figure Test model. VaD: vascular dementia, Severity: difference in dementia severity between dementia groups.

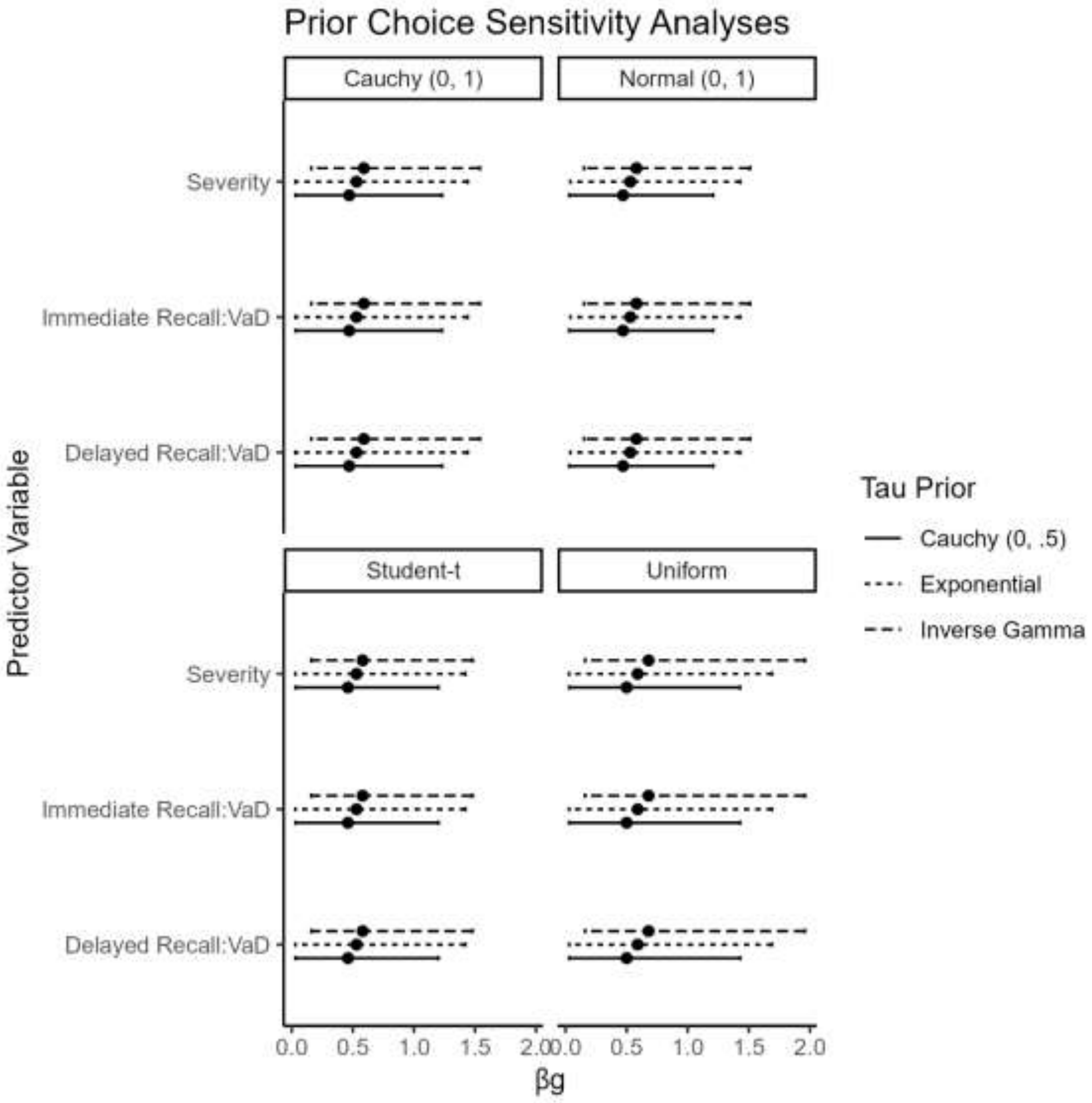

Figure 1. Study level standard deviation estimates with 95% confidence intervals for the Rey-Osterrieth Complex Figure Test model. VaD: vascular dementia, Severity: difference in dementia severity between dementia groups.

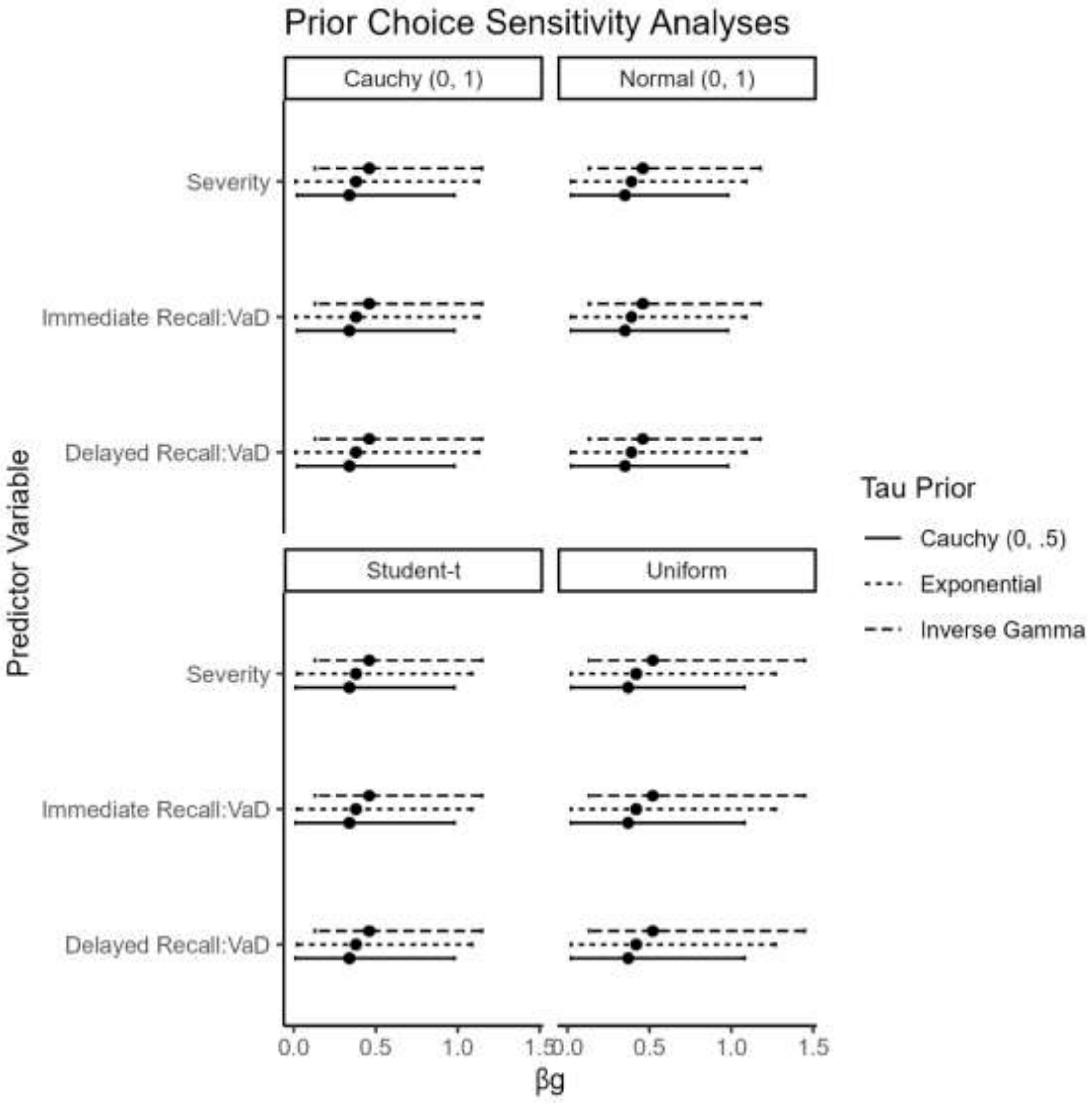

Figure 1. Effect size level standard deviation estimates with 95% confidence intervals for the Rey-Osterrieth Complex Figure Test model. VaD: vascular dementia, Severity: difference in dementia severity between dementia groups.

Visual Associative Memory

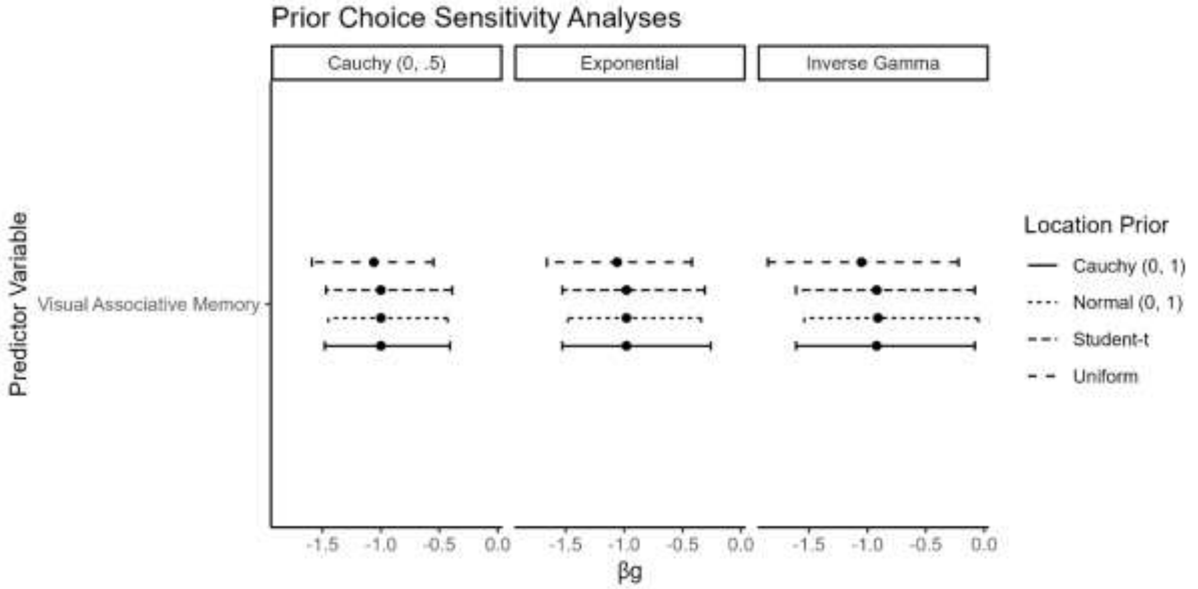

Figure 1. Regression coefficients with 95% confidence intervals for the Visual Associative Memory measures model. For tests and studies included in the analysis see Supplementary Materials 1.

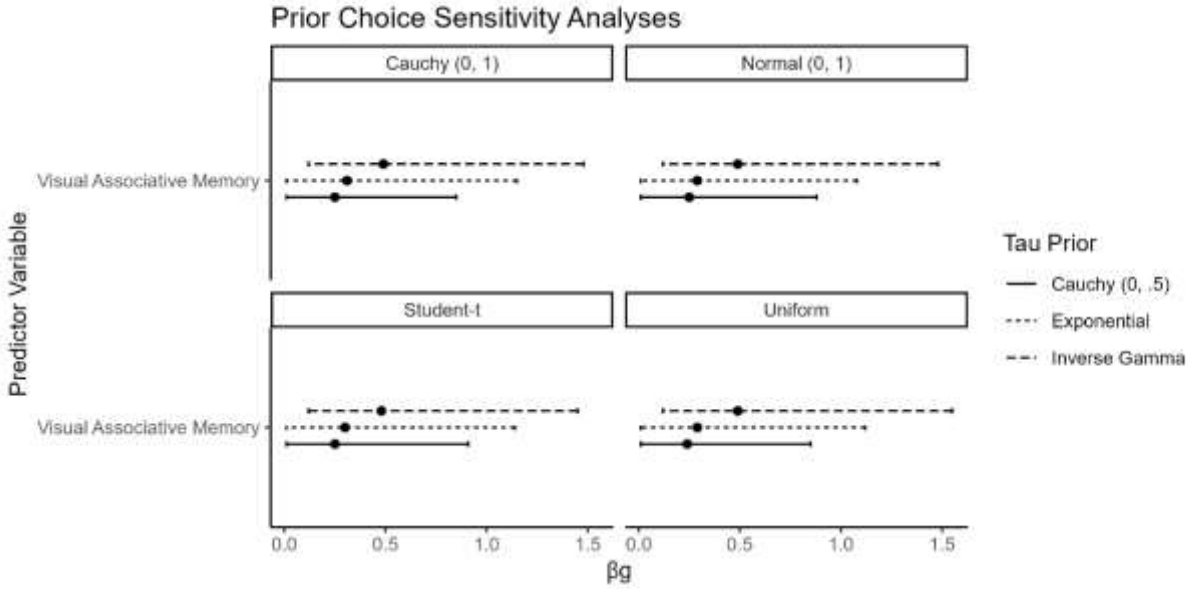

Figure 1. Study level standard deviation estimates with 95% confidence intervals for the Visual Associative Memory measures model. For tests and studies included in the analysis see Supplementary Materials 1.

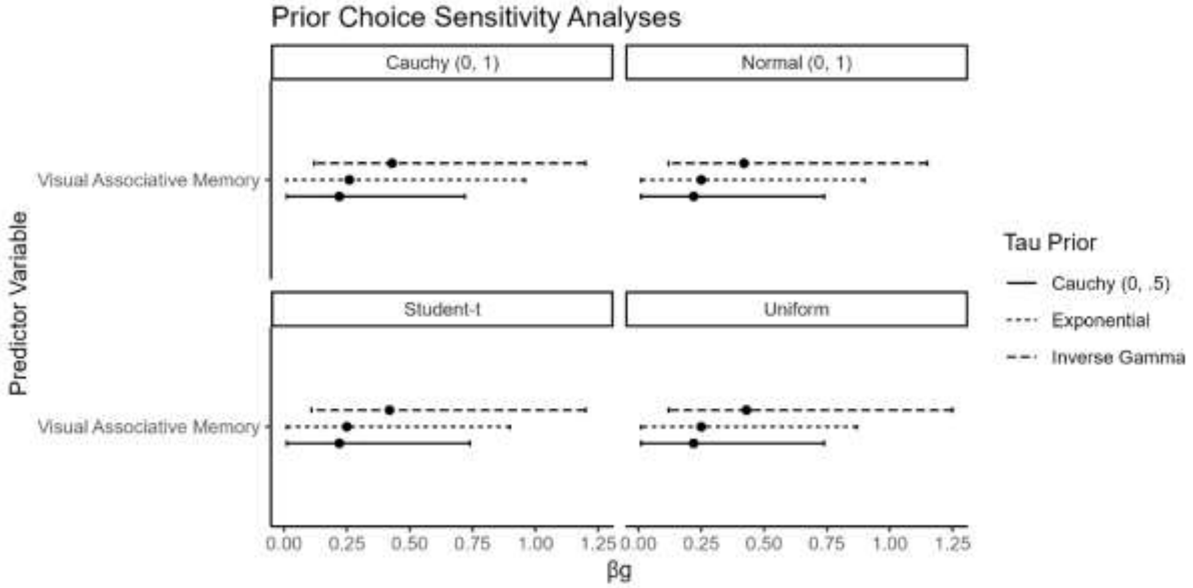

Figure 1. Effect size level standard deviation estimates with 95% confidence intervals for the Visual Associative Memory measures model. For tests and studies included in the analysis see Supplementary Materials 1.

Other Measures of Visual Memory

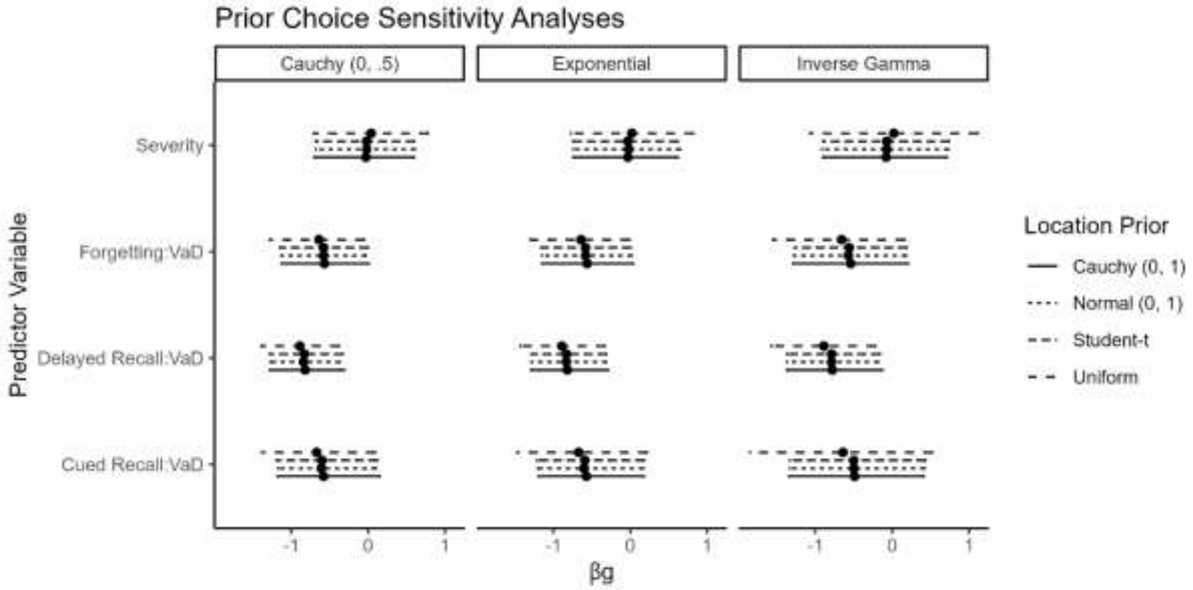

Figure 1. Regression coefficients with 95% confidence intervals for the Other Measures of Visual Memory model. For tests and studies included in the analysis see Supplementary Materials 1. VaD: vascular dementia, Severity: difference in dementia severity between dementia groups.

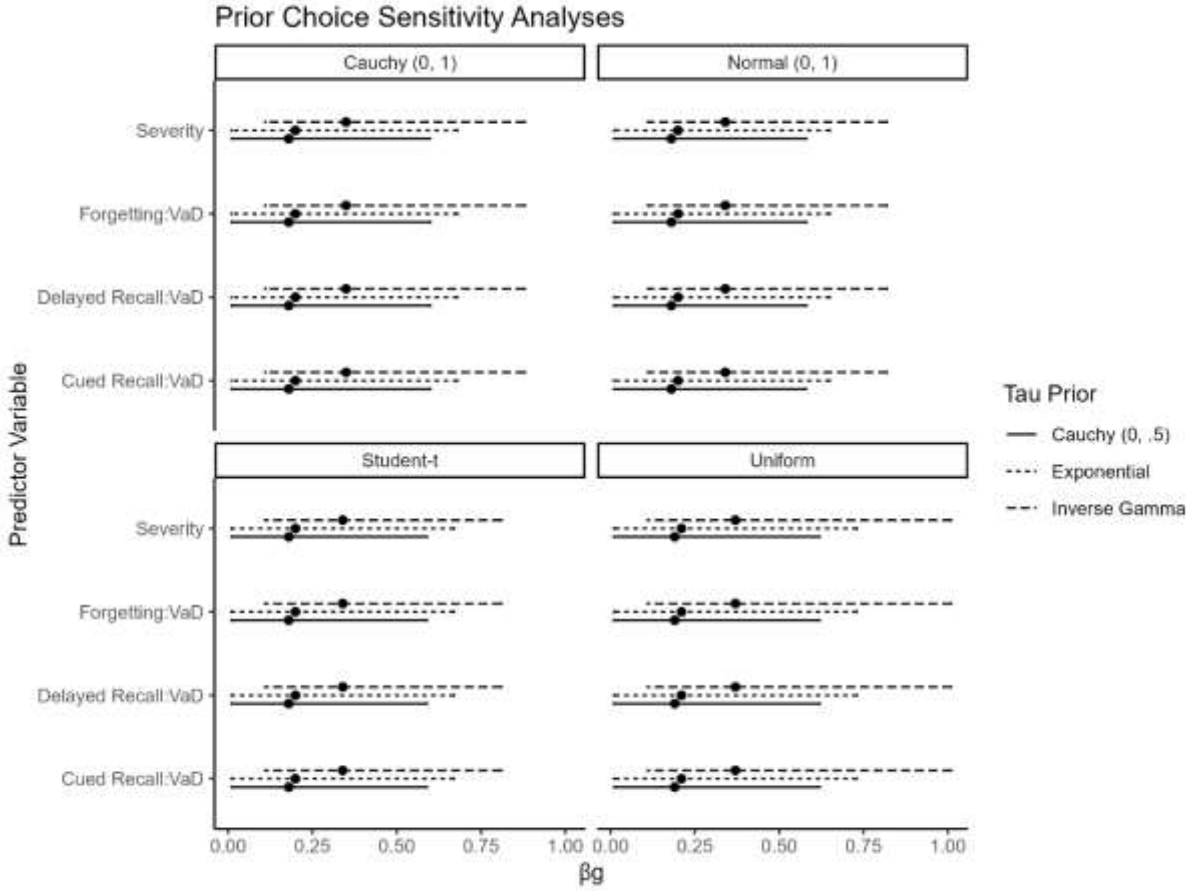

Figure 1. Study level standard deviation estimates with 95% confidence intervals for the Other Measures of Visual Memory model. For tests and studies included in the analysis see Supplementary Materials 1. VaD: vascular dementia, Severity: difference in dementia severity between dementia groups.

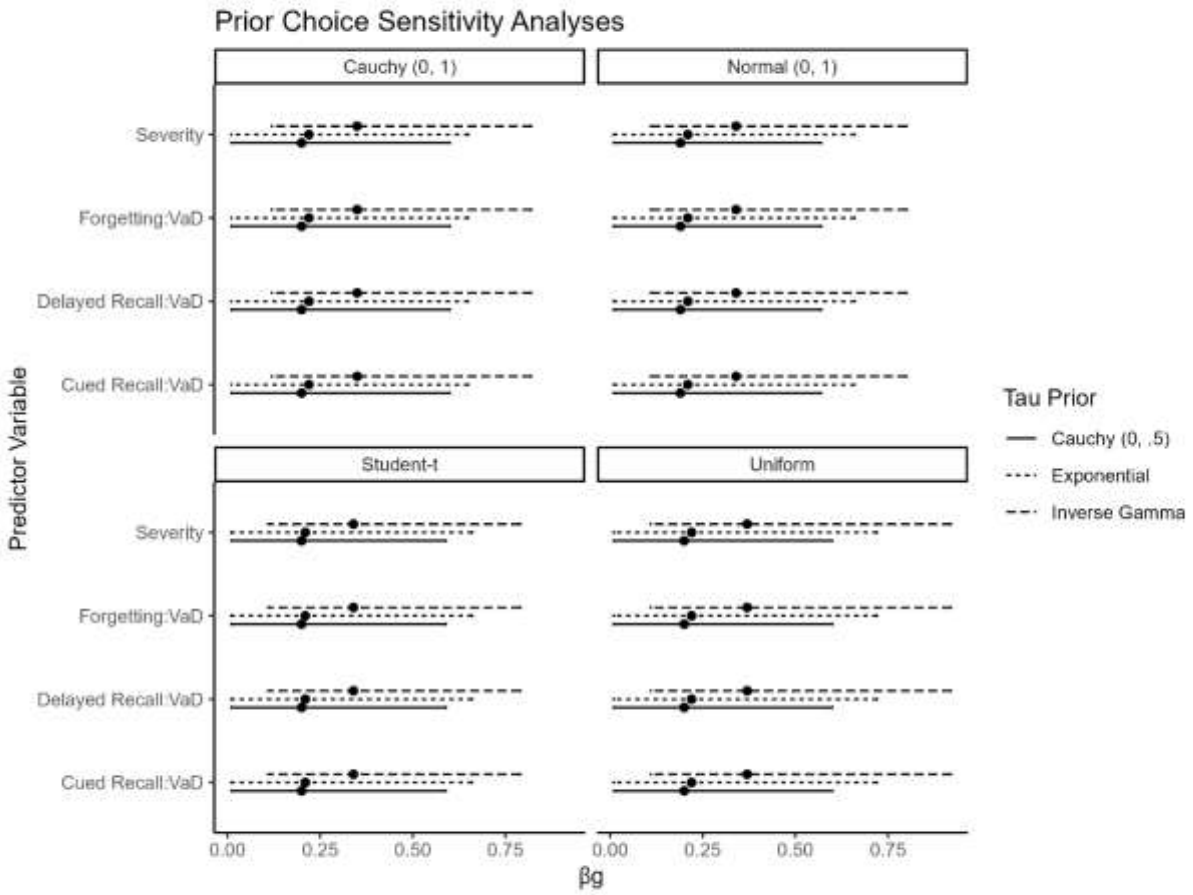

Figure 1. Effect size level standard deviation estimates with 95% confidence intervals for the Other Measures of Visual Memory model. For tests and studies included in the analysis see Supplementary Materials 1. VaD: vascular dementia, Severity: difference in dementia severity between dementia groups.

Recognition Memory

Wechsler Memory Scale

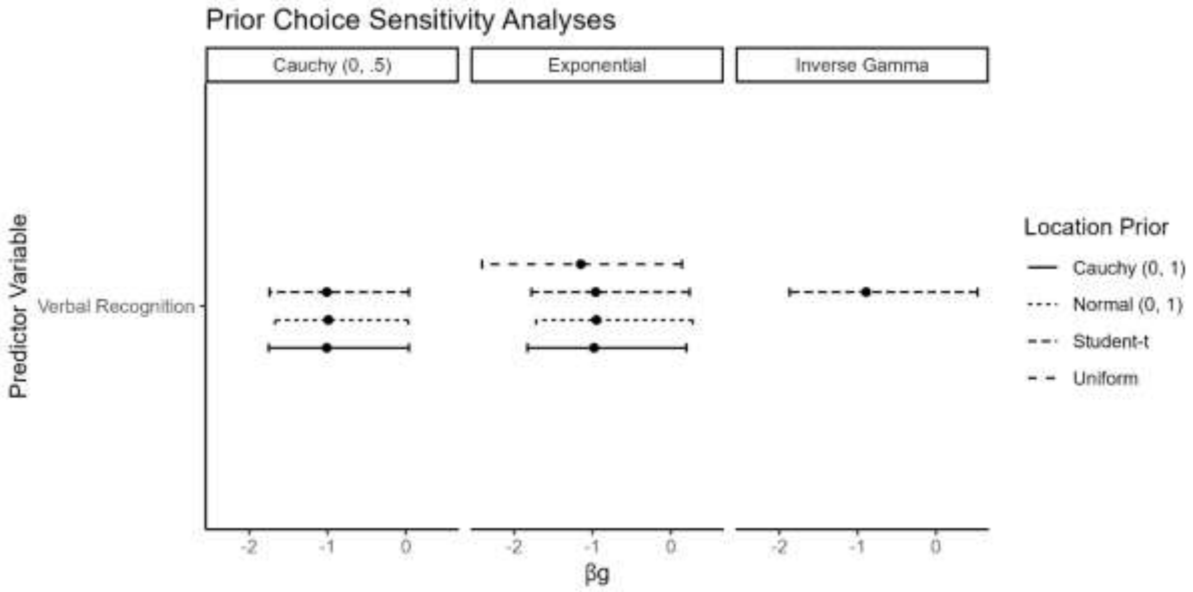

Figure 1. Regression coefficients with 95% confidence intervals for the Wechsler Memory Scale: Verbal Recognition model. For tests included in the analysis see Supplementary Materials 1.

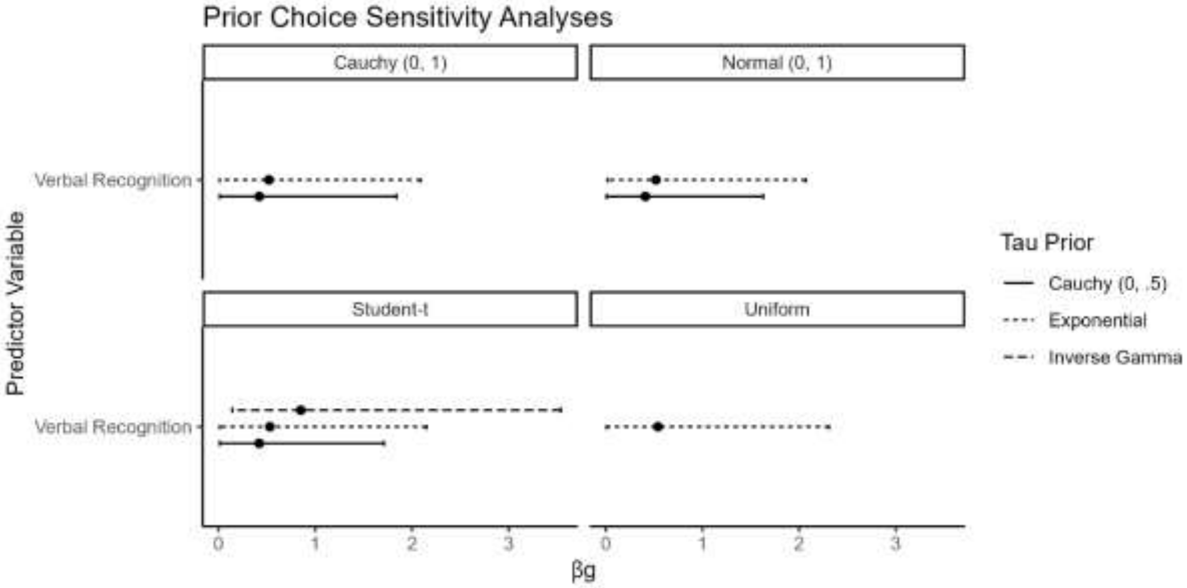

Figure 1. Study level standard deviation estimates with 95% confidence intervals for the Wechsler Memory Scale: Verbal Recognition model. For tests included in the analysis see Supplementary Materials 1.

Rey's Auditory Verbal Learning Test

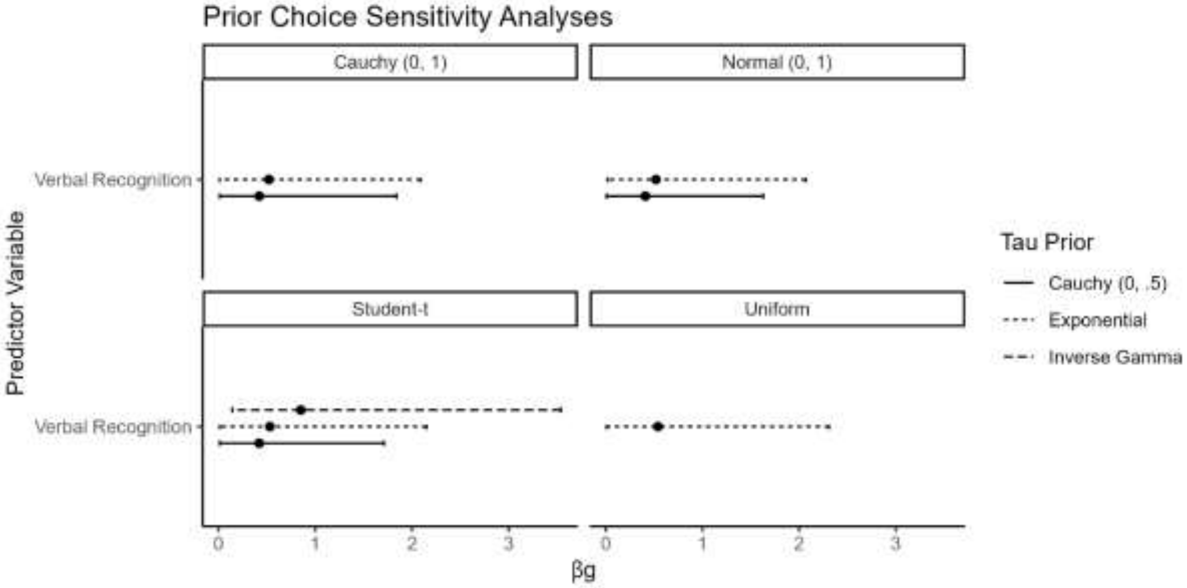

Figure 1. Regression coefficients with 95% confidence intervals for the Rey's Auditory Verbal Learning Test: Recognition model.

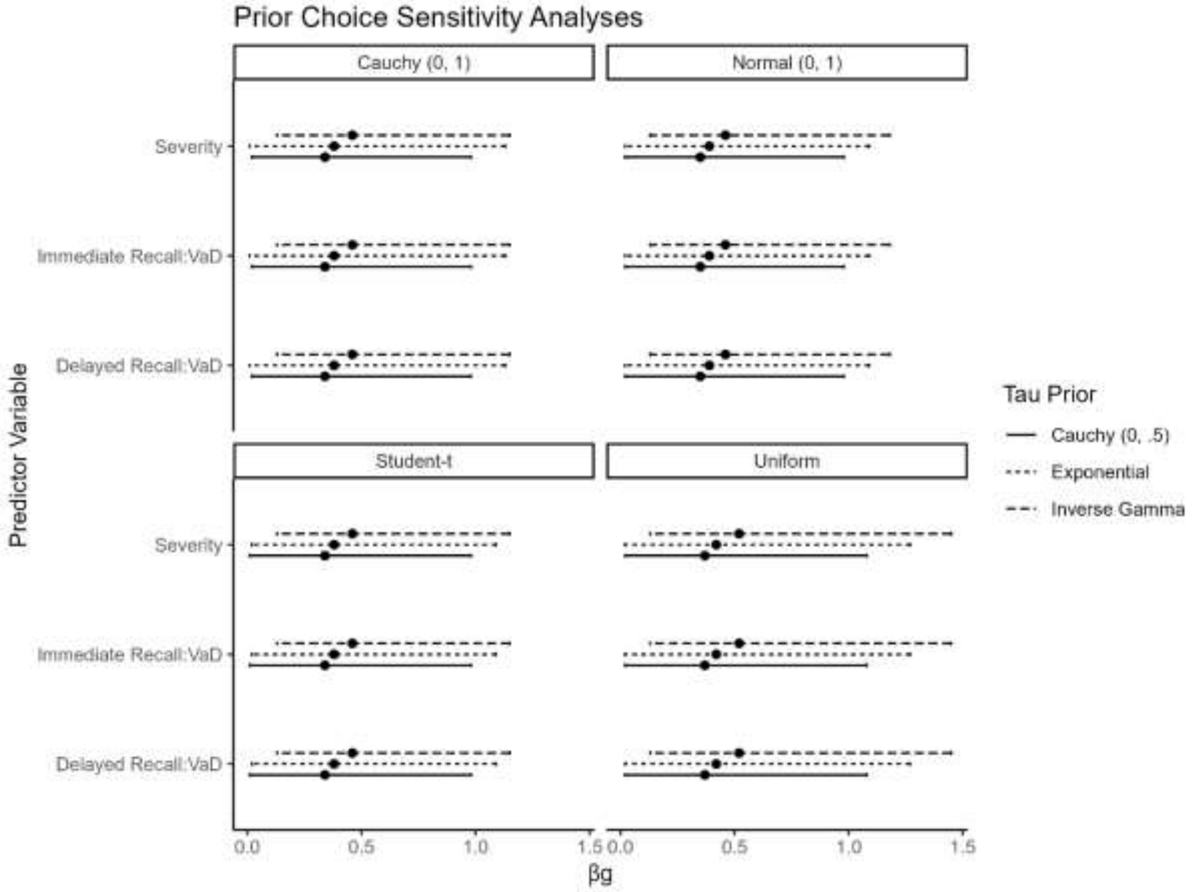

Figure 1. Study level standard deviation estimates with 95% confidence intervals for the Rey's Auditory Verbal Learning Test: Recognition model.

California Verbal Learning Test

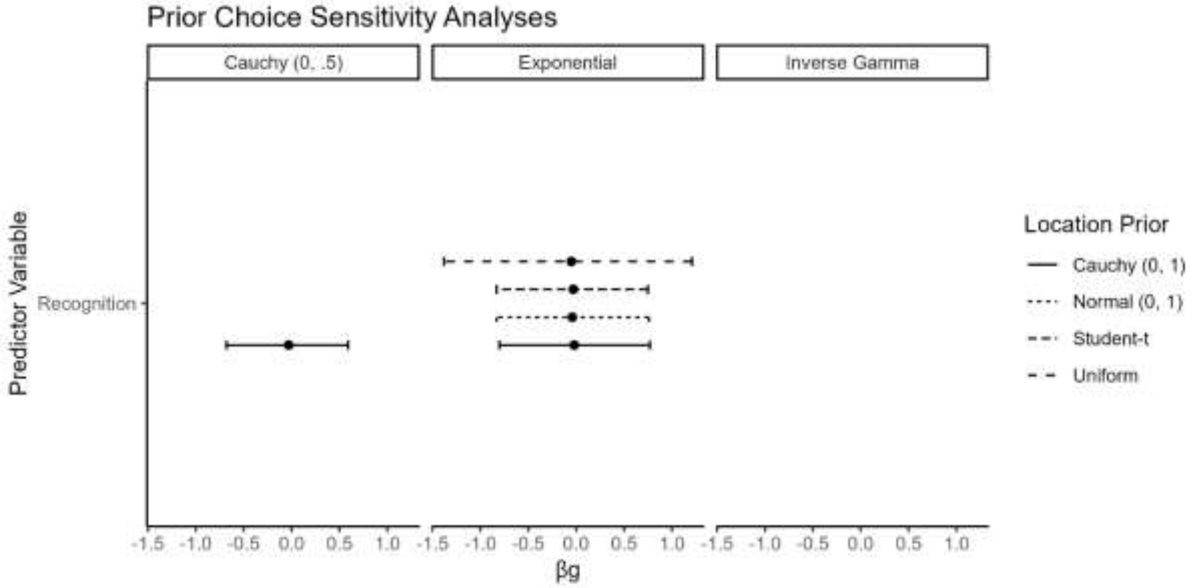

Figure 1. Regression coefficients with 95% confidence intervals for the California Verbal Learning Test: Recognition model.

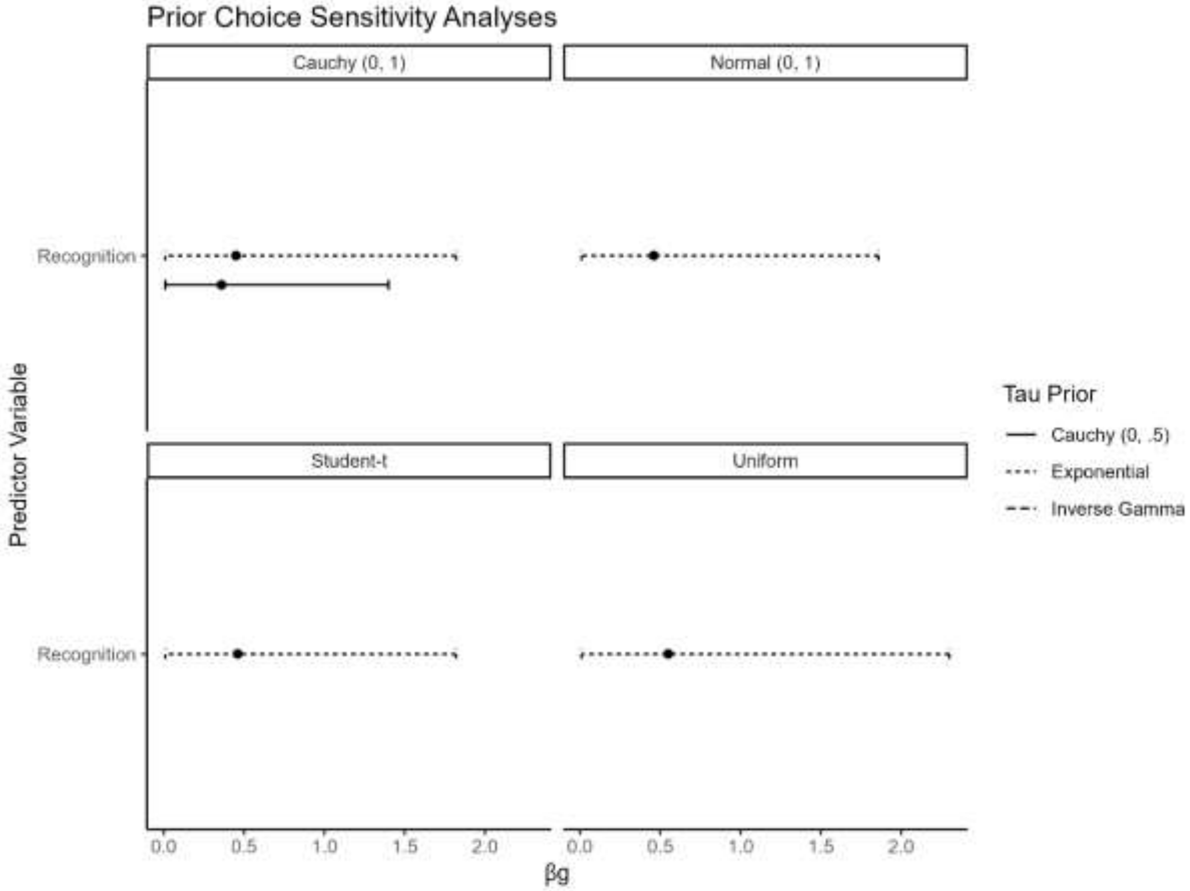

Figure 1. Study level standard deviation estimates with 95% confidence intervals for the California Verbal Learning Test: Recognition model.

CERAD Word List

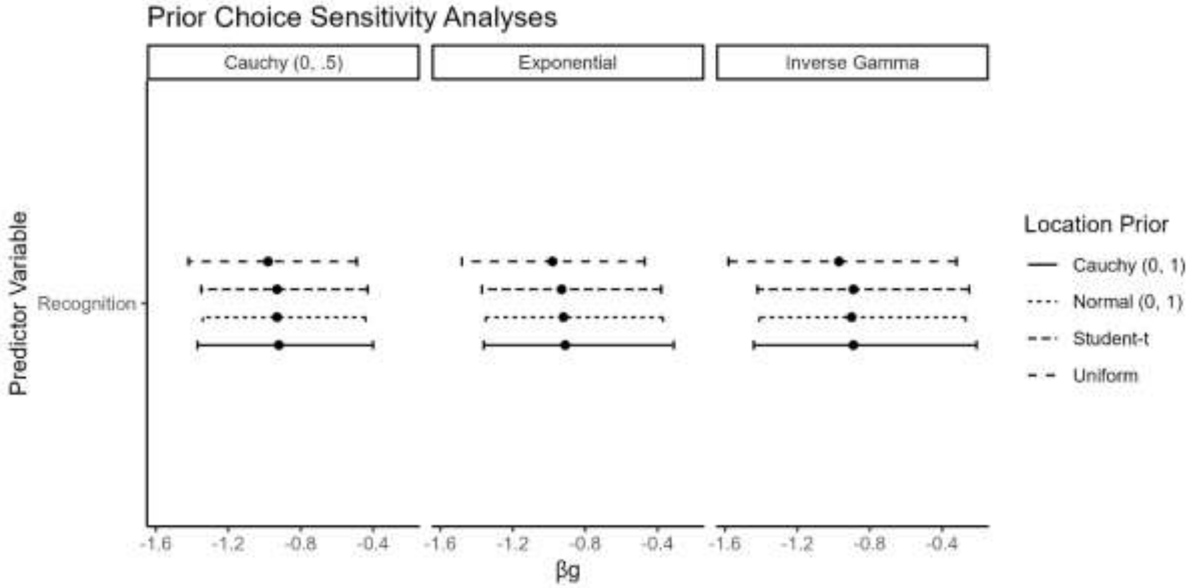

Figure 1. Regression coefficients with 95% confidence intervals for the Consortium to Establish a Registry for Alzheimer's Disease: Word List Recognition model.

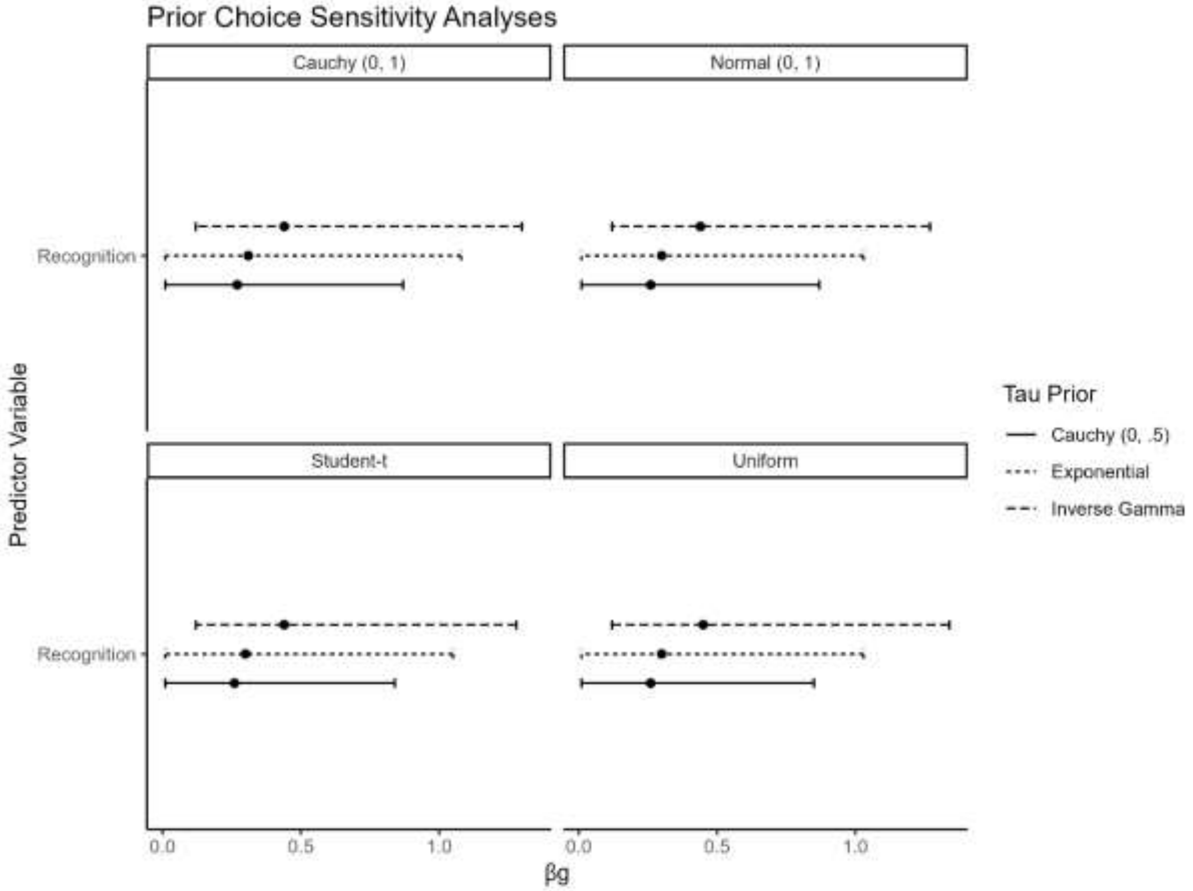

Figure 1. Study level standard deviation estimates with 95% confidence intervals for the Consortium to Establish a Registry for Alzheimer's Disease: Word List Recognition model.

Discriminability – *d’*

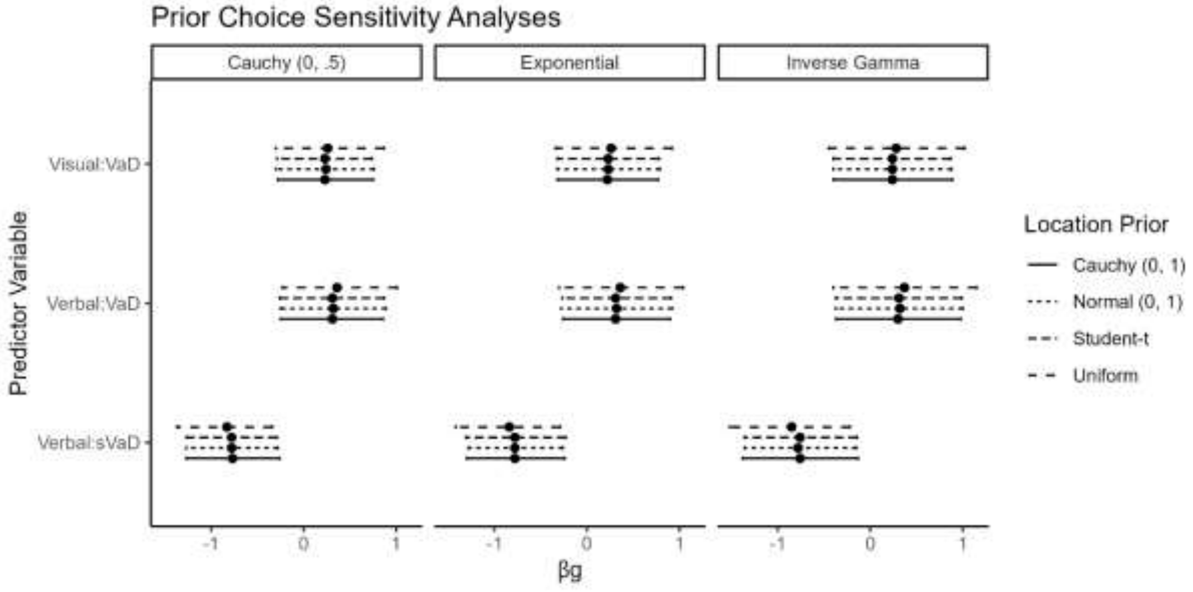

Figure 1. Regression coefficients with 95% confidence intervals for the Discriminability (*d’*) in visual (Visual) and verbal (Verbal) episodic memory model. For tests and studies included in the analysis see Supplementary Materials 1. sVaD: subcortical vascular dementia, VaD: vascular dementia.

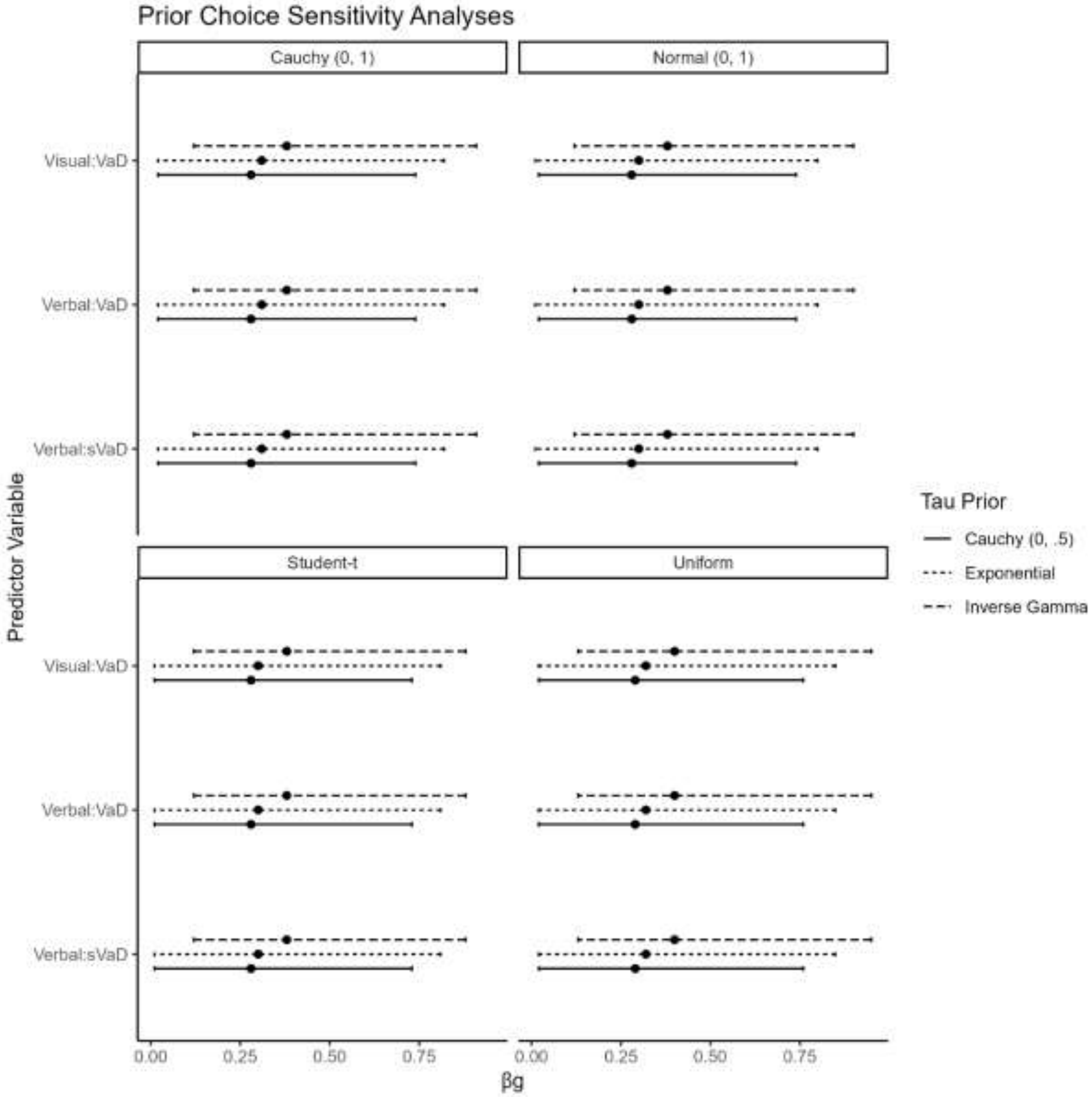

Figure 1. Study level standard deviation estimates with 95% confidence intervals for the Discriminability (*d'*) in visual (Visual) and verbal (Verbal) episodic memory model. For tests and studies included in the analysis see Supplementary Materials 1. sVaD: subcortical vascular dementia, VaD: vascular dementia.

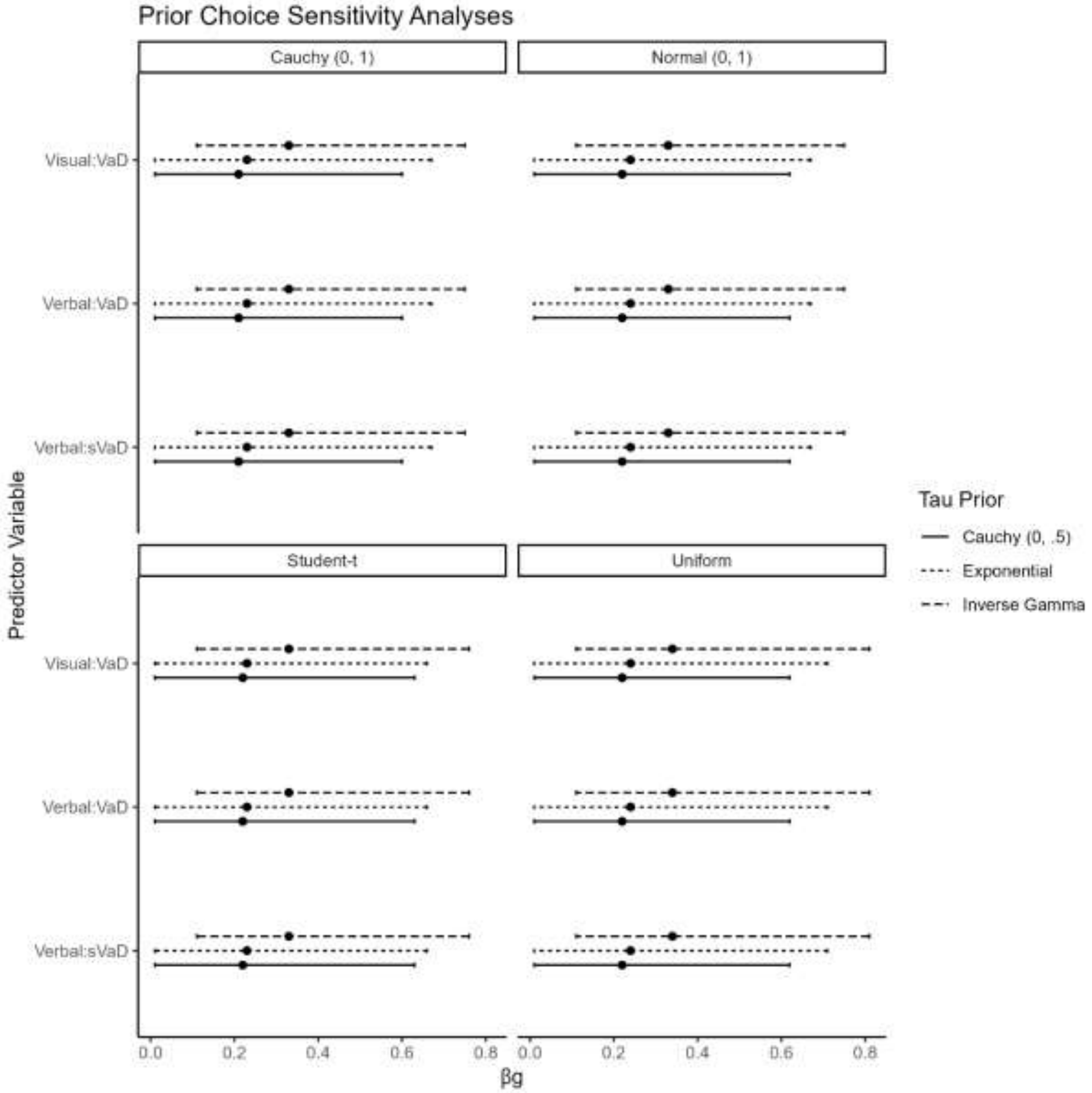

Figure 1. Effect size level standard deviation estimates with 95% confidence intervals for the Discriminability (*d'*) in visual (Visual) and verbal (Verbal) episodic memory model. For tests and studies included in the analysis see Supplementary Materials 1. sVaD: subcortical vascular dementia, VaD: vascular dementia.

Recognition Hits and False Alarms

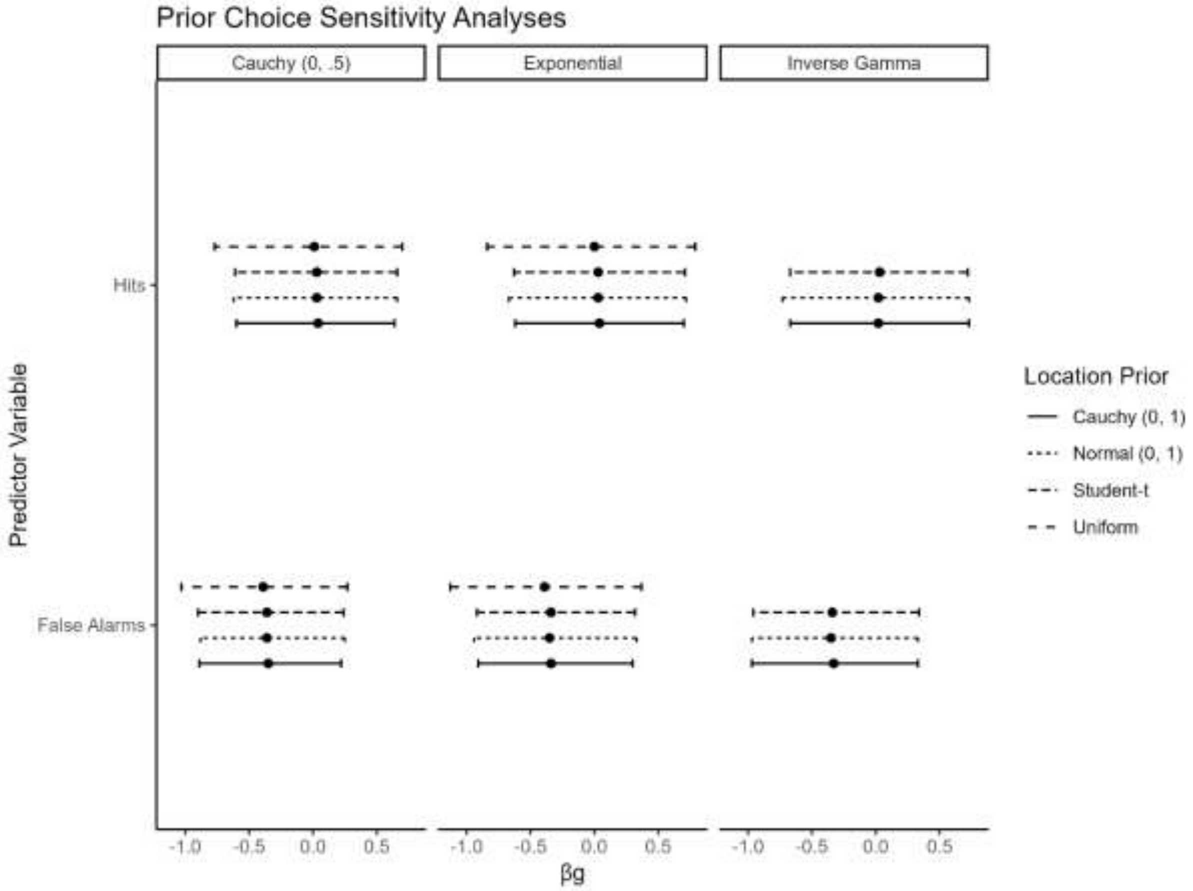

Figure 1. Regression coefficients with 95% confidence intervals for the Recognition Hits and False Alarms model. For tests and studies included in the analysis see Supplementary Materials 1.

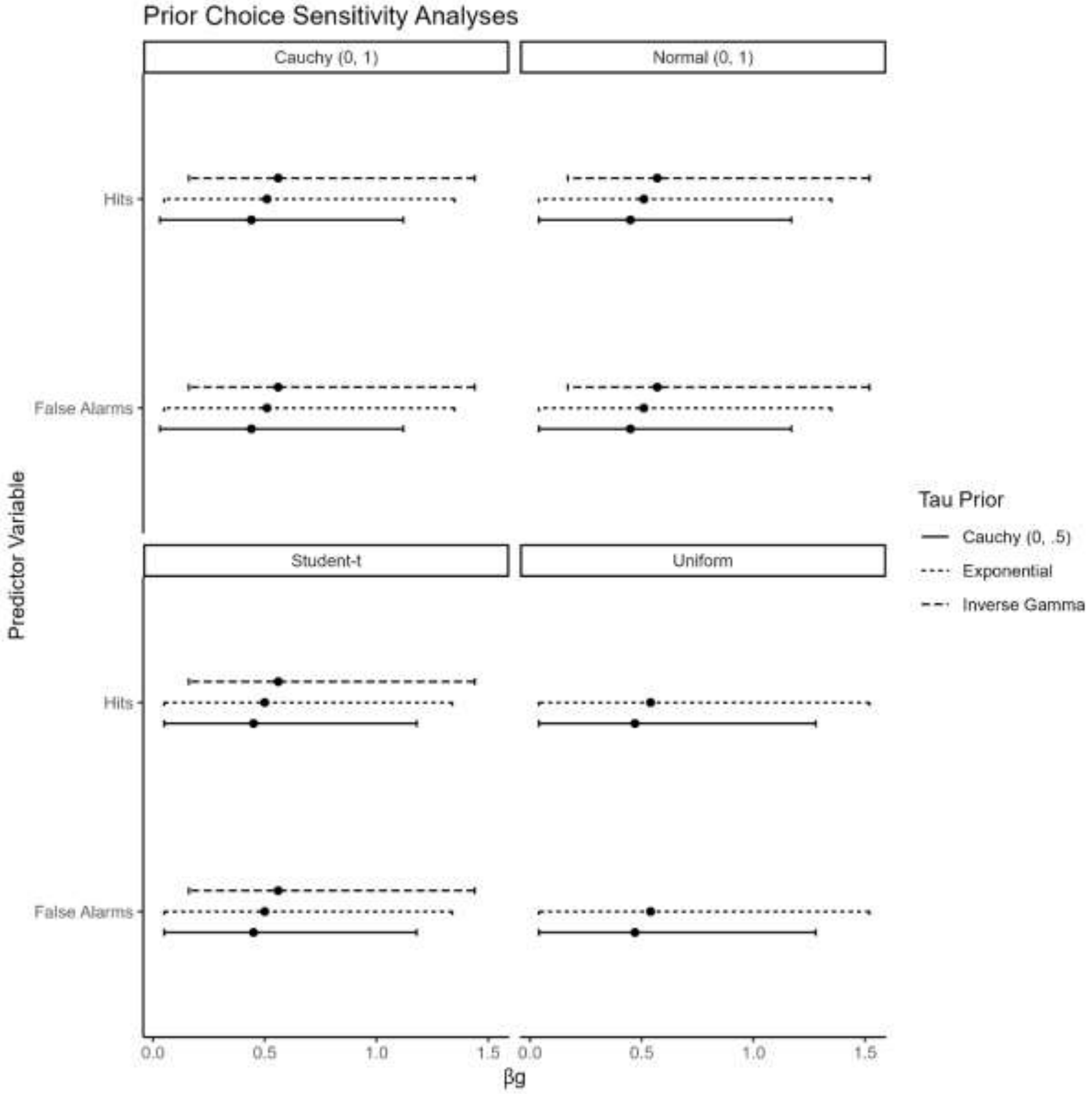

Figure 1. Study level standard deviation estimates with 95% confidence intervals for the Recognition Hits and False Alarms model. For tests and studies included in the analysis see Supplementary Materials 1.

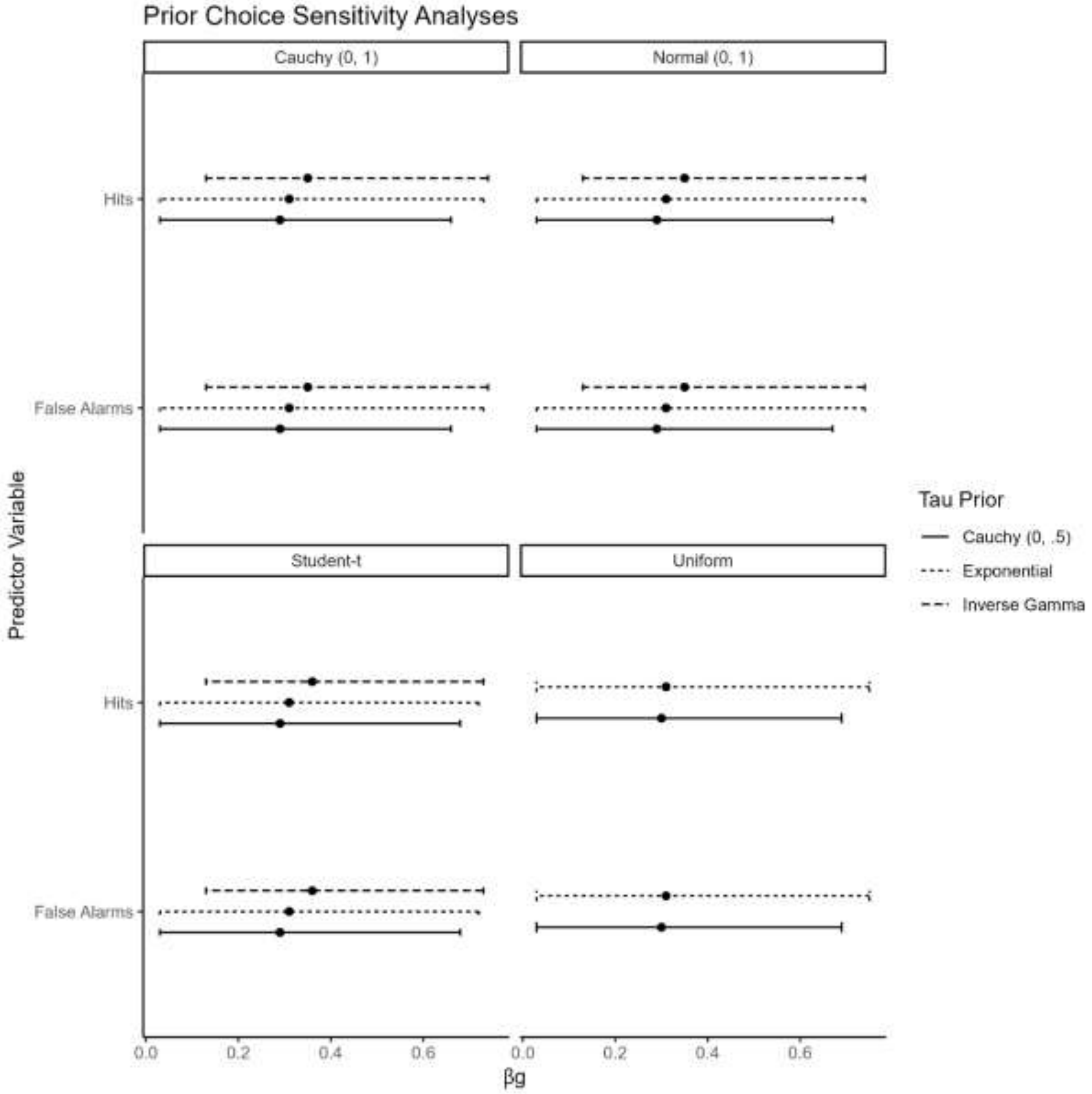

Figure 1. Effect size level standard deviation estimates with 95% confidence intervals for the Recognition Hits and False Alarms model. For tests and studies included in the analysis see Supplementary Materials 1.

Other Measures of Visual Recognition Memory

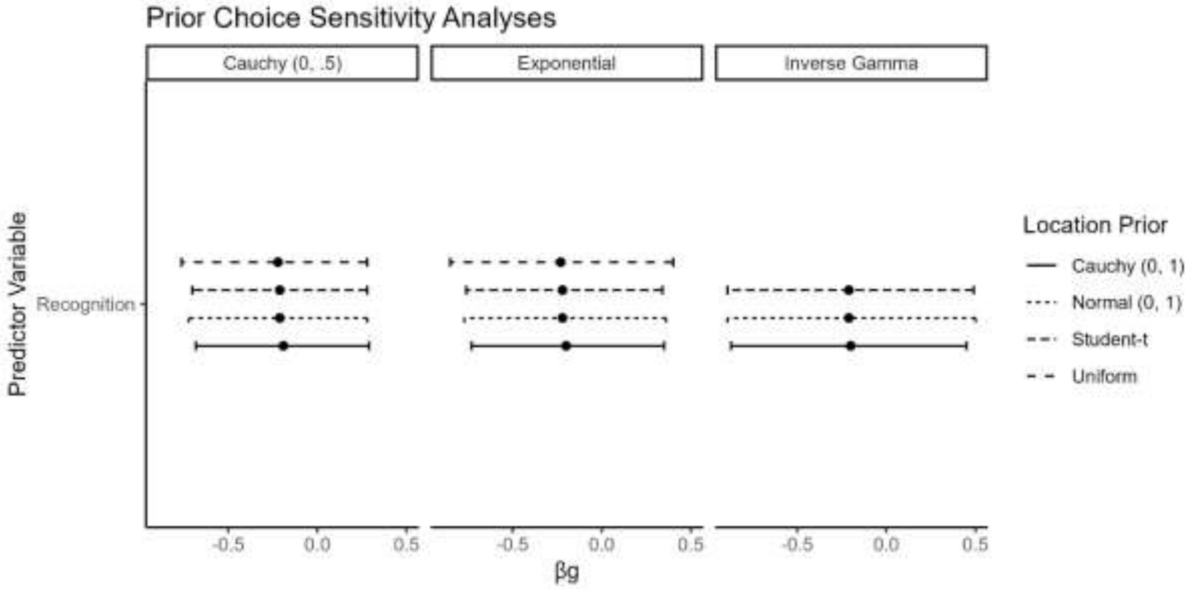

Figure 1. Regression coefficients with 95% confidence intervals for the Other Measures of Visual Recognition Memory model. For tests and studies included in the analysis see Supplementary Materials 1.

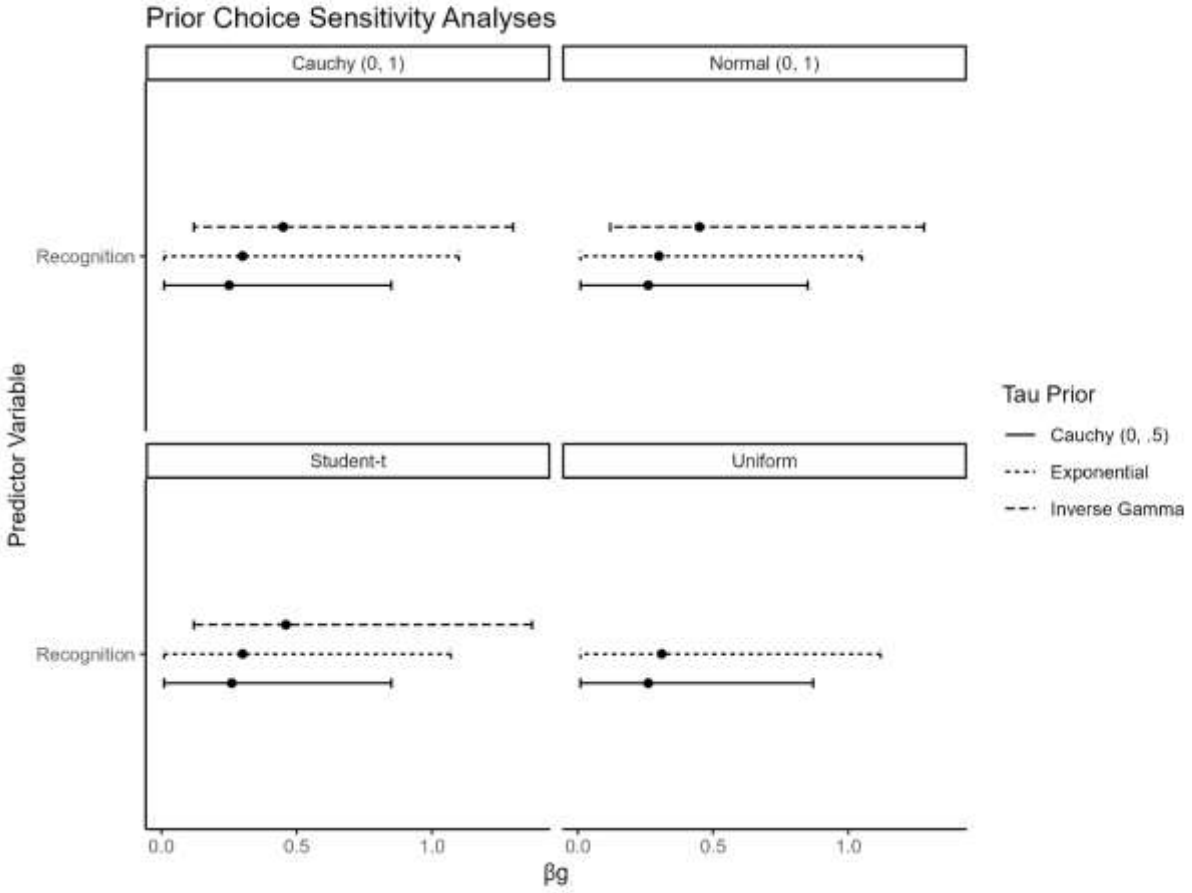

Figure 1. Study level standard deviation estimates with 95% confidence intervals for the Other Measures of Visual Recognition Memory model. For tests and studies included in the analysis see Supplementary Materials 1.

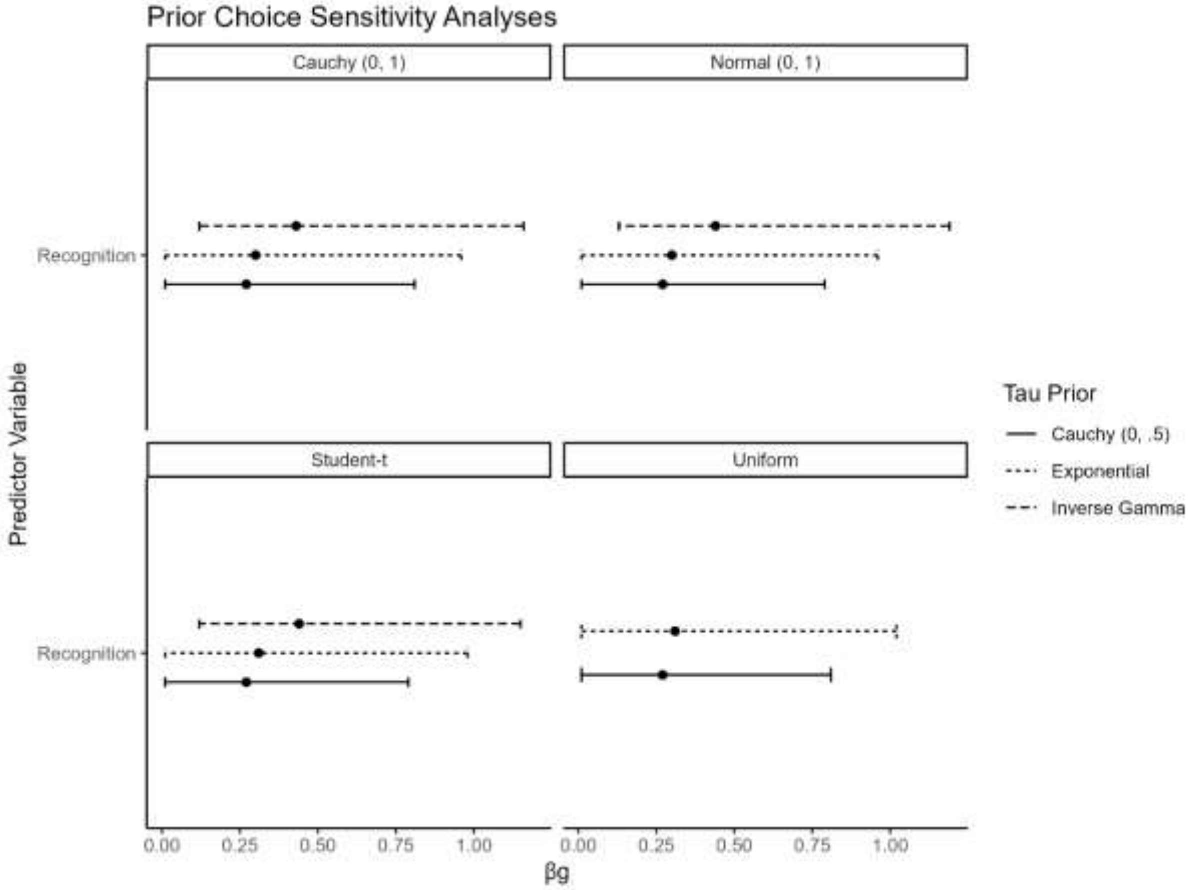

Figure 1. Effect size level standard deviation estimates with 95% confidence intervals for the Other Measures of Visual Recognition Memory model. For tests and studies included in the analysis see Supplementary Materials 1.

Other Measures of Verbal Recognition Memory

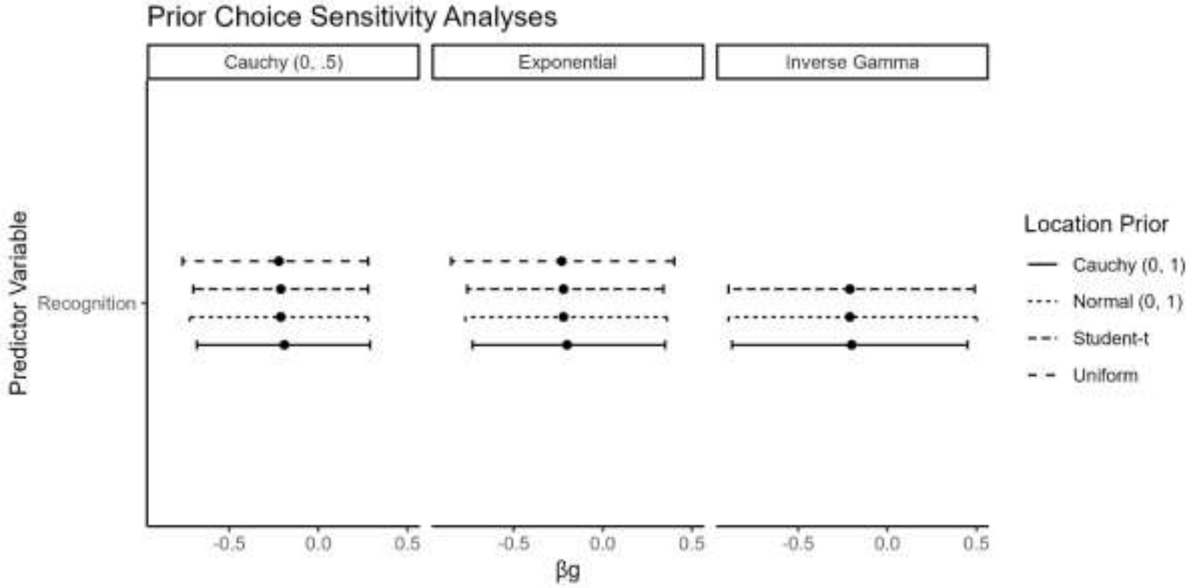

Figure 1. Regression coefficients with 95% confidence intervals for the Other Measures of Verbal Recognition Memory model. For tests and studies included in the analysis see Supplementary Materials 1.

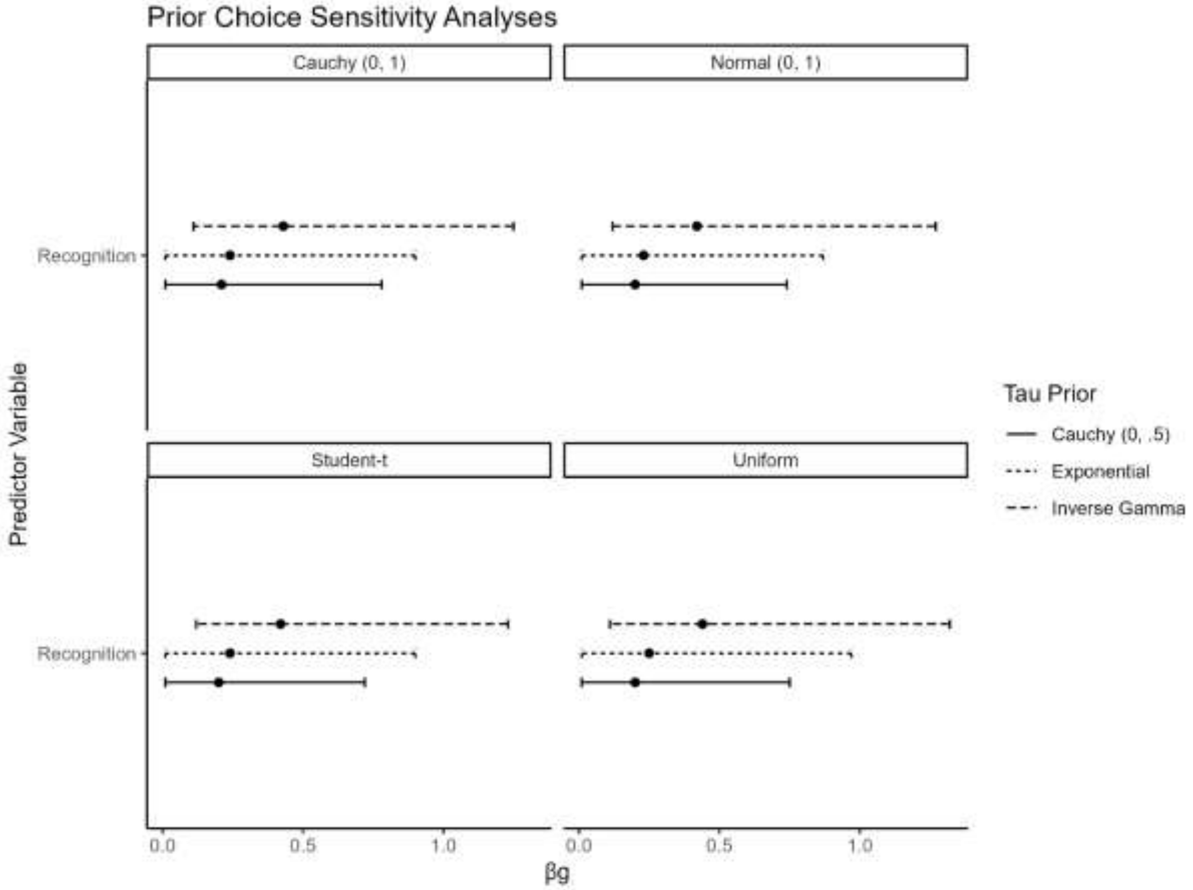

Figure 1. Study level standard deviation estimates with 95% confidence intervals for the Other Measures of Verbal Recognition Memory model. For tests and studies included in the analysis see Supplementary Materials 1.

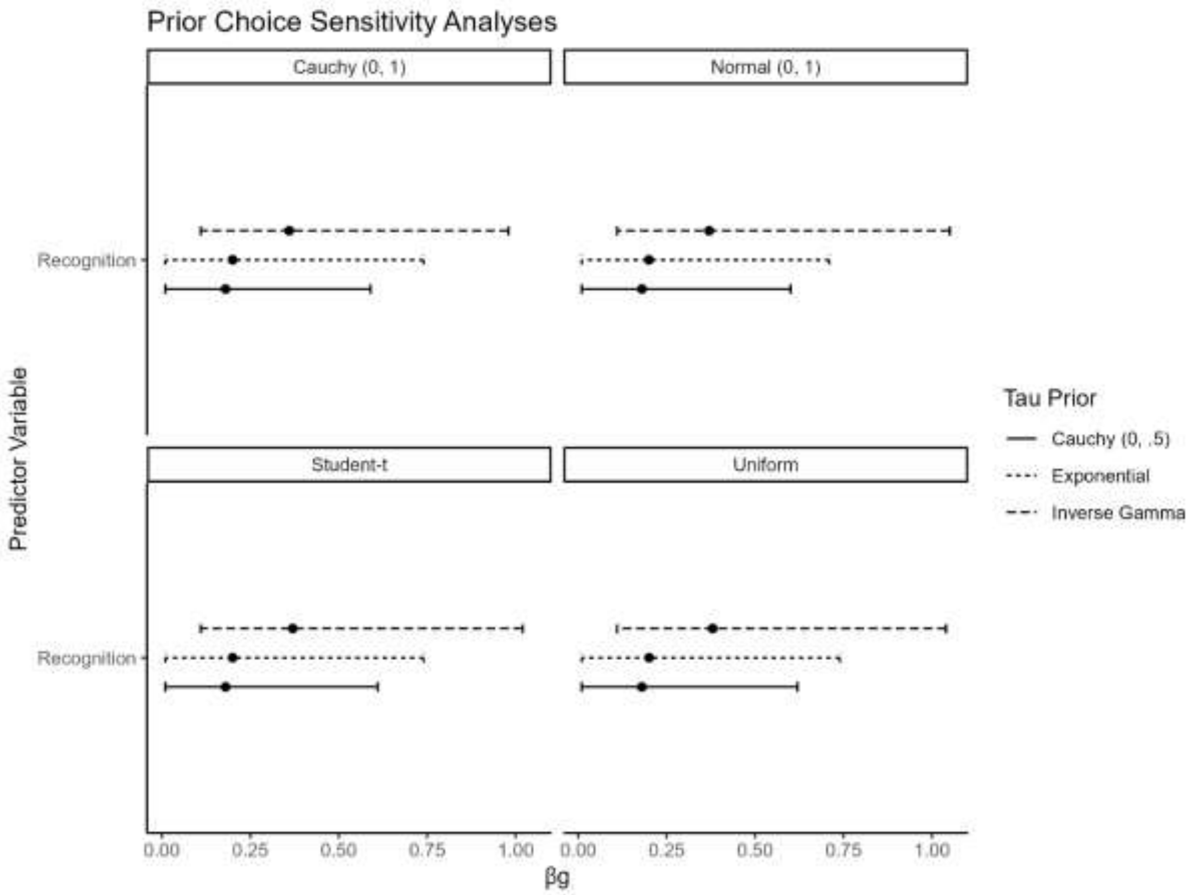

Figure 280. Effect size level standard deviation estimates with 95% confidence intervals for the Other Measures of Verbal Recognition Memory model. For tests and studies included in the analysis see Supplementary Materials 1.
