# Supplementary material for: Neuropsychological differential diagnosis of Alzheimer’s disease and vascular dementia: a systematic review with meta-regressions
Source: Front Aging Neurosci. 2023 Nov 6;15:1267434. doi: 10.3389/fnagi.2023.1267434 (PMC10657839; doi:10.3389/fnagi.2023.1267434)
Supplement: Supplementary file 4 [file Data_Sheet_2.docx]

**Supplementary Materials 4: Diagnostic Criteria**

| **Table 1. Diagnostic and Statistical Manual 5 diagnostic criteria** | |
| --- | --- |
| **Major or mild vascular neurocognitive disorder** | **Major or mild neurocognitive disorder due to Alzheimer’s disease** |
| A. The criteria are met for major or mild neurocognitive disorder.  B. The clinical features are consistent with a vascular etiology, as suggested by either of the following:   1. Onset of the cognitive deficits is temporally related to one or more cerebrovascular events. 2. *Evidence for decline is prominent in complex attention (including processing speed) and frontal-executive function*.   C. There is evidence of the presence of cerebrovascular disease from history, physical examination, and/or neuroimaging considered sufficient to account for the neurocognitive deficits.  D. The symptoms are not better explained by another brain disease or systemic disorder.  **Probable vascular neurocognitive disorder is diagnosed if one of the following is present; otherwise possible vascular neurocognitive disorder should be diagnosed:**   1. Clinical criteria are supported by neuroimaging evidence of significant parenchymal injury attributed to cerebrovascular disease (neuroimaging-supported). 2. The neurocognitive syndrome is temporally related to one or more documented cerebrovascular events. 3. Both clinical and genetic (e.g., cerebral autosomal dominant arteriopathy with subcortical infarcts and leukoencephalopathy) evidence of cerebrovascular disease is present.   **Possible vascular neurocognitive disorder is diagnosed if the clinical criteria are met but neuroimaging is not available and the temporal relationship of the neurocognitive syndrome with one or more cerebrovascular events is not established.** | A. The criteria are met for major or mild neurocognitive disorder.  B. There is insidious onset and gradual progression of impairment in one or more cognitive domains (for major neurocognitive disorder, at least two domains must be impaired).  C. Criteria are met for either probable or possible Alzheimer’s disease as follows:  **For major neurocognitive disorder:**  Probable Alzheimer’s disease is diagnosed if either of the following is present; otherwise, possible Alzheimer’s disease should be diagnosed.   1. Evidence of a causative Alzheimer’s disease genetic mutation from family history or genetic testing. 2. All three of the following are present:    1. Clear evidence of decline in memory and learning and at least one other cognitive domain (based on detailed history or serial neuropsychological testing).    2. Steadily progressive, gradual decline in cognition, without extended plateaus.    3. No evidence of mixed etiology (i.e., absence of other neurodegenerative or cerebrovascular disease, or another neurological, mental, or systemic disease or condition likely contributing to cognitive decline).   **For mild neurocognitive disorder:**  Probable Alzheimer’s disease is diagnosed if there is evidence of a causative Alzheimer’s disease genetic mutation from either genetic testing or family history.  Possible Alzheimer’s disease is diagnosed if there is no evidence of a causative Alzheimer’s disease genetic mutation from either genetic testing or family history, and all three of the following are present:   1. Clear evidence of *decline in memory and learning*. 2. Steadily progressive, gradual decline in cognition, without extended plateaus. 3. No evidence of mixed etiology (i.e., absence of other neurodegenerative or cerebrovascular disease, or another neurological or systemic disease or condition likely contributing to cognitive decline).   D. The disturbance is not better explained by cerebrovascular disease, another neurodegenerative disease, the effects of a substance, or another mental, neurological, or systemic disorder. |
| The cognitive deficits in these cases can be attributed to disruption of cortical-subcortical circuits, and complex attention, particularly speed of information processing, and executive ability are likely to be affected. A history of memory deficit early in the course, and progressive worsening of memory, language, executive function, and perceptual-motor abilities in the absence of corresponding focal lesions on brain imaging, are suggestive of Alzheimer’s disease as the primary diagnosis. | The typical presentation is amnestic (i.e., with impairment in memory and learning). Unusual nonamnestic presentations, particularly visuospatial and logopenic aphasic variants, also exist. At the mild NCD phase, Alzheimer’s disease manifests typically with impairment in memory and learning, sometimes accompanied by deficits in executive function. |

| **Table 2. International Classification of Diseases 11 diagnostic criteria** | |
| --- | --- |
| **Dementia due to cerebrovascular disease** | **Dementia due to Alzheimer’s disease** |
| **Description:**  Dementia due to brain parenchyma injury resulting from cerebrovascular disease (ischemic or hemorrhagic). The onset of the cognitive deficits is temporally related to one or more vascular events. Cognitive decline is typically *most prominent in speed of information processing, complex attention, and frontal-executive functioning*. There is evidence of the presence of cerebrovascular disease considered to be sufficient to account for the neurocognitive deficits from history, physical examination and neuroimaging.   - All diagnostic requirements for Dementia are met. - Dementia is presumed to be attributable to underlying cerebrovascular disease as demonstrated by neuroimaging, medical tests, and/or clinical history of cerebrovascular disease. - Diagnostic requirements for Alzheimer Disease Dementia, Mixed Type, with Cerebrovascular Disease are not met. - Neurocognitive symptoms often follow cerebrovascular compromise.   - In stroke, the type of neurocognitive impairment varies depending on the brain region(s) in which the stroke occurred. Stroke-related neurocognitive impairment typically begins abruptly after a stroke. Improvement in initial neurocognitive deficits is typically seen, with recovery reaching a plateau over time. Residual neurocognitive deficits often remain chronic over time.   - In contrast, in microvascular events, neurocognitive impairment typically affects so-called subcortical neurocognitive functions (e.g., attention, processing speed, and executive/frontal lobe-related functions). If microvascular events are attributed to progressing chronic conditions (e.g., hypertension, diabetes), as is common, clinical course of neurocognitive impairment may be slowly progressive. | **Description:**  Dementia due to Alzheimer disease is the most common form of dementia. Onset is insidious with *memory impairment typically reported as the initial presenting complaint*. The characteristic course is a slow but steady decline from a previous level of cognitive functioning with *impairment in additional cognitive domains* (such as executive functions, attention, language, social cognition and judgment, psychomotor speed, visuoperceptual or visuospatial abilities) *emerging with disease progression.* Dementia due to Alzheimer disease may be accompanied by mental and behavioral symptoms such as depressed mood and apathy in the initial stages of the disease and may be accompanied by psychotic symptoms, irritability, aggression, confusion, abnormalities of gait and mobility, and seizures at later stages. Positive genetic testing, family history and gradual cognitive decline are suggestive of Dementia due to Alzheimer disease.   - All diagnostic requirements for Dementia are met. - Dementia is presumed to be attributable to underlying Alzheimer Disease (8A20) based on quantified clinical assessment or standardized neuropsychological/cognitive testing, neuroimaging data, genetic testing, medical tests, family history, and/or clinical history.   Early clinical history is typically characterized by gradual onset, *progressive memory problems and word finding difficulties*, as well as mild functional impairment.  The most common form of Alzheimer Disease begins with neuronal impairment in the medial temporal lobes (the brain regions involved in memory formation). As Alzheimer Disease progresses and affects other brain regions, neurocognitive symptoms worsen.  Atypical forms of Alzheimer Disease are also characterized by progressive neurocognitive and functional impairment, with initial neurocognitive symptoms often corresponding to the brain region(s) initially affected (e.g., visual processing impairment in posterior cortical atrophy, etc.). |
